# Parallel Evolution of C-Type Lectin Domain Gene Family Sizes in Insect-Vectored Nematodes

**DOI:** 10.3389/fpls.2022.856826

**Published:** 2022-04-25

**Authors:** Jing Ning, Jiao Zhou, Haixiang Wang, Yaning Liu, Faheem Ahmad, Xiaohui Feng, Yu Fu, Xiaoting Gu, Lilin Zhao

**Affiliations:** ^1^State Key Laboratory of Integrated Management of Pest Insects and Rodents, Institute of Zoology, Chinese Academy of Sciences, Beijing, China; ^2^CAS Center for Excellence in Biotic Interactions, University of Chinese Academy of Sciences, Beijing, China; ^3^College of Forestry, Shanxi Agricultural University, Taigu, China; ^4^Department of Biosciences, COMSATS University Islamabad (CUI), Islamabad, Pakistan; ^5^School of Life Sciences, Division of Life Sciences and Medicine, University of Science and Technology of China, Hefei, China

**Keywords:** C-type lectin, gene family, insect vector, co-evolution, parallel evolution

## Abstract

The dispersal stage of pathogens is crucial for the successful spread and infection of their hosts. Some plant-parasitic nematodes (PPNs) have evolved specialized dispersal stages to reach healthy hosts by being carried out by insect vectors. Because gene gain and loss is a major factor contributing to the evolution of novel characteristics, it is essential to clarify the gene family characteristics among nematodes with different dispersal modes to disentangle the evolution of insect-mediated dispersal. Here, the size of the C-type lectin (CTL) family genes of insect-vectored nematodes was found to be drastically reduced compared with those of self-dispersing nematodes, whereas the diversity of their functional domains was significantly higher. The gene family sizes of vector-dispersed nematodes were only a twentieth of the size of that of a self-dispersing (i.e., without a biotic vector) nematode model *Caenorhabditis elegans*, and these genes were inactive during the dispersal stage. Phylogenetic analysis showed that some CTL genes of vector-borne PPNs shared higher homology to the animal parasitic nematodes compared with other PPNs. Moreover, homology modeling predicted that the CTLs of insect-vectored nematodes bear remarkable structural similarity to the lectin genes of their vector's immune system. Because CTL genes are important sugar-binding proteins for the innate immune response of *C. elegans*, the loss of some CTL genes of vector-transmitted PPNs might be responsible for their parallel adaptations to a mutualistic relationship with their vector. These results expand our understanding of the evolutionary benefits of vector-mediated transmission for the nematode and vector-nematode co-evolution.

## Introduction

Due to globalization, insect-vectored plant and animal diseases have become one of the major threats for biosafety, ecosystem function and stability as well as human health (Blaxter et al., 1998; Jones et al., 2014). One of the major pests threatening the safety of horticultural and forest ecosystem is plant-parasitic nematodes (PPNs), which have caused extensive damage to agricultural and cash crops worldwide (Jones et al., 2014). Over 4,100 species of PPNs have been described to date, which collectively pose a serious threat to global food and ecological security (Demangeat et al., 2010). Global agricultural economic losses caused by PPNs have been estimated to cost more than US$ 157 billion per year (Abad et al., 2008). Due to accelerated globalization and, hence, increased spread of these pests, this estimate is expected to increase in the future.

The PPNs have evolved a variety of dispersal strategies that help them expand their distribution. Some of the PPNs are self-dispersed (i.e., not using plant or animal vectors), such as some root-knot nematodes, which have a very short free-living stage in the soil. These PPNs can be transported to new uninfested areas by water or by clinging to soil particles (Dutta et al., 2012). However, the vast majority of PPN species have a wide parasitic range (i.e., number of suitable hosts) and are transmitted vertically from mother to offspring *via* seeds and/or horizontally *via* intermediate hosts. The stem and bulb nematode, *Ditylenchus dipsaci*, e.g., is known to infect more than 500 plant species and can use weeds as an intermediate, yet non-preferred, hosts and as vectors until a preferred host is available (Trankner, 1992; Vovlas et al., 2011). However, a few species have become more specialized with more limited host repertoire and have secondarily lost the ability for wide-range dispersal. For example, the hosts of *Bursaphelenchus* spp., such as *Bursaphelenchus xylophilus* (pinewood nematode) and *Bursaphelenchus cocophilus*, are limited to a few species of *Pinus* and *Palmae*, respectively. These PPNs have significantly improved the efficiency of their transmission by evolving special dispersal stages that are carried out to the healthy hosts by insect vectors (Jordaan et al., 1987; Moens and Perry, 2009). However, evidence of evolution behind the transition from self-dispersing to vector-mediated dispersal of nematodes is lacking.

Changes in the number of gene copies in a specific gene family represent a raw source of evolutionary innovations (Kondrashov et al., 2002; Chen et al., 2010). More copies of a specific gene could increase the content of the associated protein, which may improve the performance of the animal in a task in which the protein is needed (Ollivier et al., 2016; Cheng et al., 2017). However, a reduction in gene family size does not necessarily mean the loss of its biological function. Growing body of research shows that gene loss can be beneficial by providing an evolutionary mechanism for phenotypic adaptations (Chen et al., 2010; Sharma et al., 2019). For example, sperm whales, *Physeter macrocephalus*, have lost the gene AMPD3 which encodes an erythrocyte-specific enzyme related to O_2_ affinity of hemoglobin. This is probably an adaptation to sustained diving because lower affinity facilitates O_2_ release from hemoglobin to O_2_-depleted tissues (O'Brien et al., 2015; Sharma et al., 2019). In addition, a reduction in the size of hair- and epidermis-related gene families has likely facilitated the adaptation of cetaceans to the aquatic environment, resulting in a thicker and smoother epidermis that can better cope with water pressure and drag force (Sharma et al., 2019). This “less is more” principle can shed light on possible molecular and cellular mechanisms underlying various adaptations and, therefore, investigating gene losses has a great potential to reveal the genomic basis of evolutionary processes.

Despite the genomes of numerous nematode species have been sequenced and are available in repositories, the genomic basis of the evolutionary transition from self-dispersing to insect-vectored dispersal is not clear. However, the availability of these genomes enables the use of comparative genomics to associate genomic differences with the different nematode dispersal modes. We suggest three approaches that are most suitable for this task, namely, first, comparison of the genomes of nematode species differing in their dispersal strategies (e.g., self-dispersed without a biotic vector vs. insect-vectored) and lifestyles (insect- and/or plant-parasitic and free-living); second, comparison of gene expression of dispersal and propagative stages within insect-vectored nematode species; and finally, analysis of molecules that parasitic nematodes excrete and secrete when encountering the vector and host. Following these approaches, we explored genetic factors that influence the successful loading of nematodes to their insect vector. We focused on the C-type lectin (CTL) family because these genes play important roles in avoiding vector immunity. We found that the size of the CTL family was drastically reduced among insect-vectored nematodes compared with that of self-dispersing nematodes, while the high diversity of its functional domains was retained. The CTL genes of insect-vectored nematodes were not expressed during the dispersal stage, i.e., when being transported to a new host tree *via* an insect. Moreover, the CTL protein structures of insect-vectored parasitic nematodes were similar to their vector's homologous protein. Our results suggest that gene family reduction and lower expression of CTLs are mechanism that has likely contributed to the adaptation of nematodes from self-dispersing to insect-vectored dispersal strategy.

## Materials and Methods

### Collection of Genomic Data

We collected the protein sequence of 12 nematode species from NCBI (https://www.ncbi.nlm.nih.gov/) and WormBase (https://www.wormbase.org/), including two PPNs vectored by *Monochamus alternatus* (i.e., *B. xylophilus* and *Bursaphelenchus mucronatus*), two animal-parasitic nematodes vectored by blood-feeding insects (i.e., *Brugia malayi* and *Loa loa*), and self-dispersing nematodes spreading without a biotic vector, including two PPNs (i.e., *Meloidogyne arenaria* and *Meloidogyne javanica*), two animal-parasitic nematodes (i.e., *Steinernema carpocapsae* and *Enterobius vermicularis*), four free-living nematodes (i.e., *Caenorhabditis elegans, Caenorhabditis brenneri, Caenorhabditis remanei*, and *Pristionchus pacificus*), and one outgroup *Echinococcus granulosus* (Supplementary File 1).

### Phylogenetic Analysis

A phylogenetic tree was constructed with program RAxML using the maximum-likelihood (ML) algorithm and estimation of multiple sequence alignment (MSA) with a self-expanding bootstrap of 200 and substitution model from ProtTest using *E. granulosus* as an outgroup (Stamatakis, 2006). This ML tree was converted into an ultrametric time-scaled phylogenetic tree by r8s using the calibrated times from the TimeTree (http://timetree.org/) website. Finally, Evolview was used to display the phylogenetic tree (Subramanian et al., 2019). The Café (version 3.0) program was used to analyze the expansion and contraction of gene families (De Bie et al., 2006).

### Gene Family Clustering and Orthology Prediction

To understand the evolutionary relationships among insect-vectored and self-dispersing nematodes, we performed systematic gene comparisons among these groups. Proteins shorter than 30aa or with frameshifts were removed. The All-vs.-All similarity alignment of protein sequences was constructed using Diamond (1e−5) (Buchfink et al., 2015). Orthofinder was used to infer the orthologous genes within the species and obtain the gene family based on the similarity of protein sequence alignments (Emms and Kelly, 2019). Based on the gene family clustering results, the candidate-contracted gene families of the four insect-vectored nematodes were identified by comparing them with corresponding candidate genes from self-dispersing nematodes (using a *t*-test in SPSS version 20.0 to compare the gene numbers among the two groups of species).

### Identification of CTLs

Blastp and HMMScan (version 3.0) were used to search for CTL genes of *B. xylophilus, B. mucronatus, B. malayi, L. loa, S. carpocapsae, E. vermicularis, M. arenaria, and M. javanica* based on those form of *C. elegans, C. remanei, C. brenneri*, and *P. pacificus* (Johnson et al., 2010). In addition, domain analyses of these proteins were carried out with NCBI Conserve-Domain Tool (https://www.ncbi.nlm.nih.gov/cdd) and Pfam (http://www/sanger.82 ac.uk/Software/Pfam/). Signal peptides (SPs) were analyzed using SignalP version 3.0 (http://www.cbs.dtu.dk/services/SignalP), and transmembrane domain was searched by TMHMM server version 2.0 (http://www.cbs.dtu.dk/services/TMHMM/). Domain pattern was re-drawn using TBtools version 0.53 (Chen et al., 2020). The 3D structure prediction for the CTLs was carried out in the SWISS-MODEL workspace (http://swissmodel.expasy.org/workspace/) (Waterhouse et al., 2018). The subcellular location of nematode CTL proteins was predicted using WoLF PSORT (http://www.genscript.com/psort/wolf_psort.html). MSAs were executed using MUSCLE software (Edgar, 2004). Phylogenetic trees were constructed by the neighbor-joining (NJ) method in MEGA 6, 1,000 repetitions of bootstrap (Tamura et al., 2013). All CTL domain (CTLD) sequences from other model species used in this study are provided as Supplementary File 2.

### Transcriptomic Data Collection

The PPNs (*B. xylophilus*) were collected from infected trees in the Zhashui area of Shangluo City in Shaanxi Province (33°35′31″N, 108°49′25″E). The propagative (i.e., non-dispersal) stages (L_1_-L_4_) of the nematodes were cultured on *Botrytis cinerea* plates and retrieved using a modified Baermann funnel technique (Baermann, 1917). The third-stage dispersal juveniles (L_III_) were collected from infected pine trees. The chunks were cut into wood chips, and the nematodes were extracted using the Baermann's funnel technique. In addition, the fourth-stage dispersal juveniles (L_IV_) were collected from the trachea of newly emerged vector beetles (*M. alternatus*). Impurities were removed from the nematodes by sucrose flotation, and then they were washed using phosphate-buffered saline, flash-frozen with Trizol, and stored at −80°C until used in experiments.

Total RNA samples were extracted from propagative (L_2_, L_3_, L_4_, both female and male) and dispersal (L_III_ and L_IV_) stages of *B. xylophilus* using Trizol reagent (Thermo Fisher Scientific) according to the manufacturer's instructions. The integrity of RNA was assessed using a NanoDrop ND-1000 spectrophotometer (NanoDrop Technologies, Inc., Wilmington, DE 19810, USA). The A260/A280 ratio of the RNA was between 1.8 and 2.0. A dynabeads messenger RNA (mRNA) purification kit was used to purify mRNA (RiboPureTM Kit Family Ambion, Life Technology, Waltham, MA, USA). Then, RNA-seq libraries were constructed and sequenced on the Illumina Hiseq 2000 platform (Illumina, San Diego, CA, USA) in the Beijing Institutes of Biological Sciences (Chinese Academy of Sciences) (Hou et al., 2015). Raw data were processed to trim short or terminal low-quality bases and adapter sequences. Microbial contaminants with identity 95% and coverage 90% were removed by Blast. The clean data were assembled by Trinity (version 2.6.5) (Grabherr et al., 2011) to obtain reference transcripts due to the reference transcriptome (PRJNA381109). The differentially expressed transcripts (DETs) were analyzed using DEG-seq2, and differentially expressed mRNAs were finally selected with fold change > 2 and *p-adjusted* < 0.05 (Wang et al., 2010).

The RNA-seq data of *M. alternatus* with different life stages and with/without *B. xylophilus* were collected from the SRA database of the NCBI (Supplementary File 4) and processed by the same methods with *B. xylophilus*. The Illumina paired-end transcriptomic data of *B. malayi* were collected from the SRA database of the NCBI (Supplementary File 4). The RNA samples from the abovementioned sequences were extracted from different life stages. All the transcriptomic sequencing reads were trimmed to remove low-quality data and adaptor sequences using the IlluQC_PRLL.pl of NGS QC Toolkit and an in-house Perl script “QC_remove_low_quality.pl” (https://github.com/jianbone/L_vannamei_genome). Reads were mapped to the annotated *B. malayi* genome assemblies with Bowtie2 (Langdon, 2015). The relative change in gene expression was analyzed using the pheatmap package in R environment.

### Quantitative Real-Time Polymerase Chain Reaction

Quantitative real-time polymerase chain reaction (qPCR) was performed in triplicate for each sample using SYBR PrimeScript RT-PCR Kit (TaKaRa, Dalian, China) on an MX3000P Thermal cycler (Stratagene, La Jolla, California, USA) with designed specific primers (Supplementary Table 1). Thermal cycling was performed at 95°C for 30 s, followed by 40 cycles of 95°C for 5 s, 60°C for 20 s, and 95°C for 1 min, 55°C for 30 s, and 95°C for 30 s. β-actin of *B. xylophilus* was used as an internal control. The expression values were calculated using the 2^−ΔΔCT^ method and normalized to β-actin expression levels (Livak and Schmittgen, 2001). One-way ANOVA (SPSS version 20.0) with Tukey's multiple comparison test was used to compare the number of relative fold changes in gene expression. Data were considered significant when *p* < 0.05.

## Results

### Phylogeny of the Nematodes

**Figure 1:**
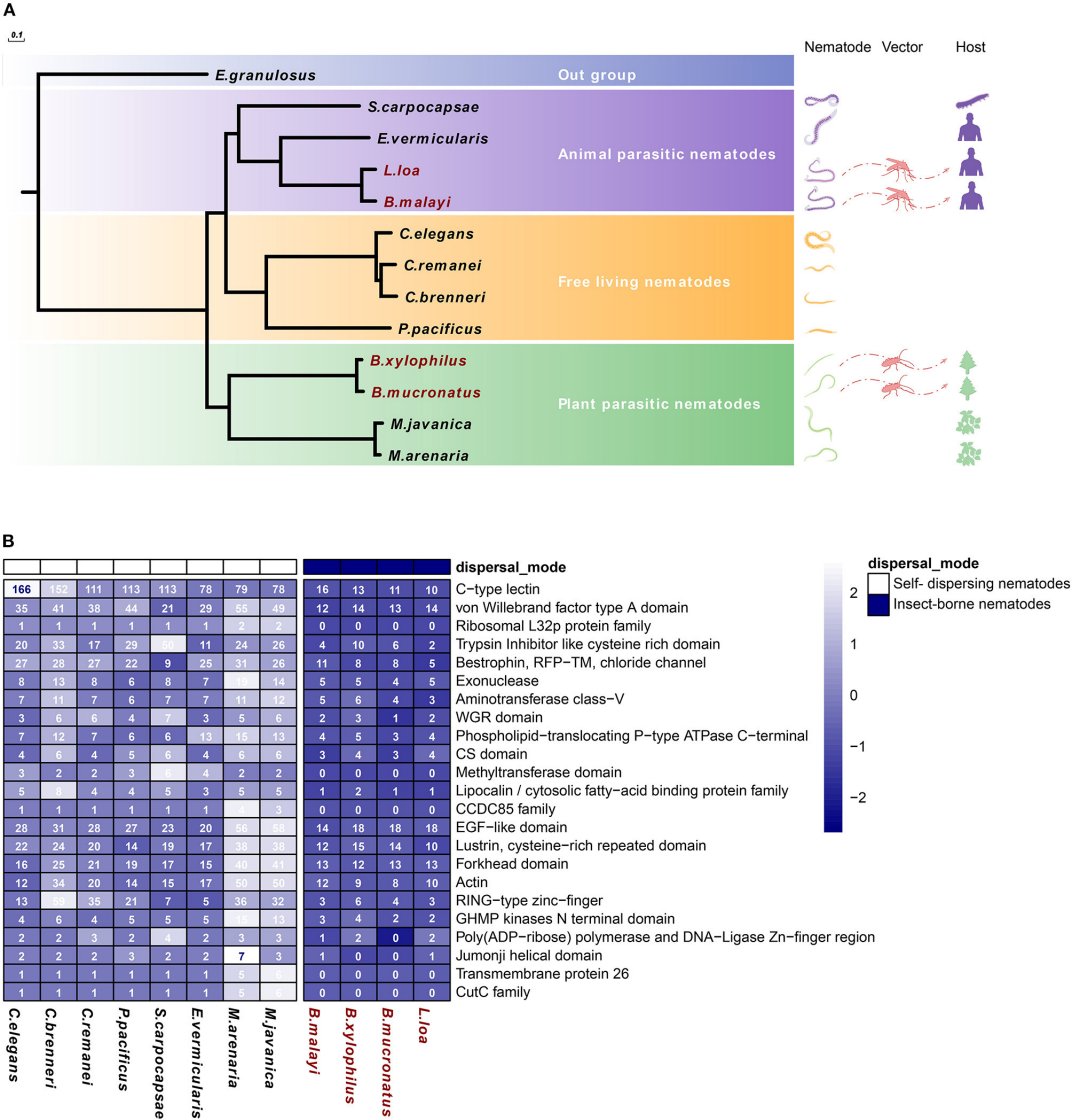
Significantly reduced gene families in insect-vectored nematodes compared with self-dispersing nematodes. **(A)** Phylogenetic tree and gene families of different nematode species in Nematoda phylum. The following nematode species were used: insect-vectored nematodes include two PPNs (i.e., *B. xylophilus* and *B. mucronatus*), two animal-parasitic nematodes (i.e., *B. malayi* and *L. loa*), and self-dispersing nematodes, including two PPNs (i.e., *M. arenaria* and *M. javanica*), two animal-parasitic nematodes (i.e., *S. carpocapsae* and *E. vermicularis*), and four free-living species (i.e., *C. elegans, C. remanei, C. brenneri*, and *P. pacificus*). *E. granulosus* in Taeniidae was used as an outgroup, 200 repetitions of bootstrap. **(B)** Distribution pattern of the shortened gene families in 12 nematode species. The gene number of each row was normalized in the heatmap, and the actual gene number is shown in the plot.

Compared with other nematodes, insect-vectored parasites were expected to show adaptations, i.e., genetic changes to this new method of transmission compared with self-dispersing (ancestral trait) nematodes. Therefore, comparing the genomes of insect-vectored nematodes with other nematodes could help to reveal the molecular basis of nematode-vector transmission. We compared the genome among nematodes differing in their dispersal strategy and lifestyle (plant- or animal-parasitic or free-living), including two PPNs vectored by *M. alternatus* (i.e., *B. xylophilus* and *B. mucronatus*), two animal-parasitic nematodes vectored by blood-feeding insects (i.e., *B. malayi* and *L. loa*), and self-dispersing nematodes spreading without a biotic vector, including two plant-parasitic nematodes (i.e., *M. arenaria* and *M. javanica*), two animal-parasitic nematodes (i.e., *S. carpocapsae* and *E. vermicularis*), and four free-living nematodes (i.e., *C. elegans, C. remanei, C. brenneri*, and *P. pacificus*). A total of 104,444 gene families were clustered among these species using OrthoFinder. The species with similar lifestyle were clustered into monophyletic groups, and the four vector-dispersed nematode species were also clustered with their corresponding suborders. This observation suggests that the four insect-vectored nematodes do not originate from the same common ancestor and that their ancestors may have been different from those of the self-dispersing nematodes (Figure 1A).

A genome-wide comparison revealed large and frequent changes in the size of the gene families. These changes resulted from the high rate of gene gain (through duplication) and loss (through deletion or pseudogenization) and the evolution of completely new genes. Based on the gene family clustering resulting, we identified 23 gene families whose sizes were significantly reduced among the four insect-vectored nematodes. Many of the reduced gene families were found to be involved in nucleotide repair, gene expression regulation, and immune system (Figure 1B). Exonuclease and DNA ligase and phospholipid-translocating ATPase were two reduced gene families that participate in nucleotide repair. Fork head domain is a common type of protein domain that is often found in transcription factors. Its purpose is to bind DNA, while the Zinc-finger domain-containing proteins function in gene transcription, translation, and other functions (Klug and Rhodes, 1987; Kaufmann and Knochel, 1996). CTLD and epidermal growth factor (EGF)-like domain are two major reduced gene families involved in the immune system. The newly completed *C. elegans* genome sequence encodes 278 predicted proteins containing the CTLD domain, which is the seventh most abundant domain (Zelensky and Gready, 2005; Brown et al., 2018). In addition, the EGF-like domain plays an important role in the immune system and apoptosis. Both EGF and the von Willebrand factor type A (VWA) domains are common additional domains of CTLs (Stetak et al., 2006; Lenting et al., 2015).

### C-Type Lectin Gene Family Contraction

**Figure 2:**
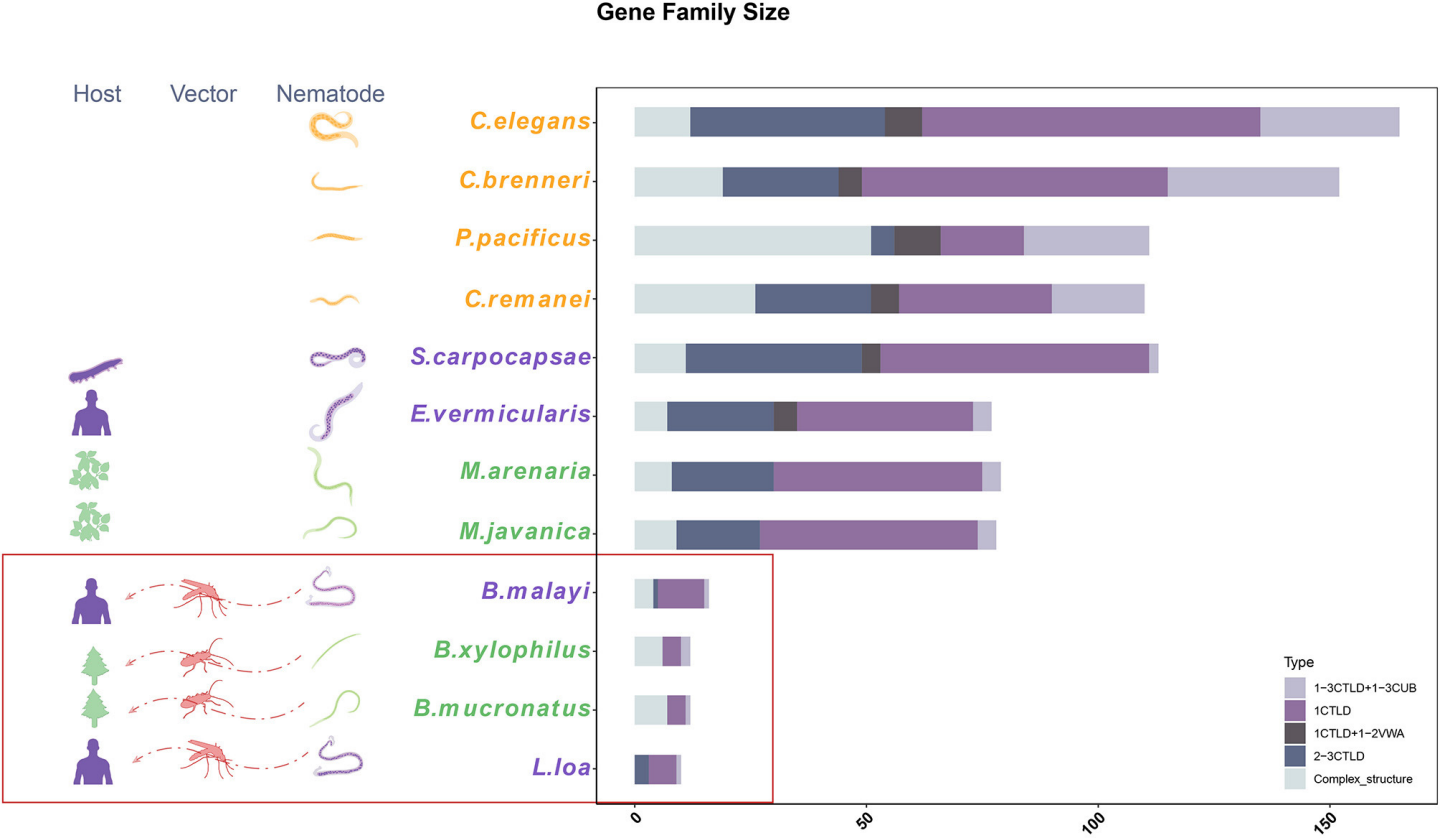
The gene family size and classification of C-type lectin (CTL) genes. The CTL gene family was classified into five types based on the domains.

To estimate the gene family contraction of the CTL accurately, we identified all CTL genes and classified them according to their domain characteristics. The most common type of CTL contains one CTLD domain, whereas other types also contain additional CTLDs, complement C1r/C1s Uegf Bmp1 (CUB), or VWA domains or some uncommon domains. Among the 12 nematodes with whole-genome sequence-based data used here, the size of the CTL family was highly reduced among insect-vectored nematodes compared with the self-dispersing nematodes. The order of species based on the number of CTLs from highest to lowest was *C. elegans, C. brenneri, S. carpocapsae, P. pacificus, C. remanei, M. arenaria, M. javanica, E. vermicularis, B. malayi, B. xylophilus, B. mucronatus*, and *L. loa* with 166, 152, 113, 111, 110, 79, 78, 77, 16, 12, 12, and 10 CTLs, respectively. Despite this, the species with the highest ratio of complex structure of CTLs were *B. xylophilus* and *B. mucronatus* (6/12) (Figure 2). The subcellular localizations of nematode CTL proteins were predicted using WoLF PSORT (Table 1). Most of them had a high probability to be located in the extracellular matrix based on signalP results. Except for extracellular CTLs, most proteins are located on the surface of the plasma membrane, which is common for CTL receptors (CLRs) (Geijtenbeek and Gringhuis, 2016). Transmembrane CLRs can use various intracellular signaling pathways to directly modulate cellular, developmental, homeostatic, and immunological responses (Osorio and Sousa, 2011). However, the rest of the proteins were presumably located in mitochondria, cytoplasm, endoplasmic, cytoskeleton, nucleus, and peroxisome.

**Table 1:** Subcellular localization of C-type lectins.

	**Cel**	**Cbn**	**Cre**	**Ppa**	**Sca**	**Mar**	**Mja**	**Eve**	**Bma**	**Bxy**	**Bmu**	**Llo**
Extracell	98	88	57	34	81	25	33	31	6	6	5	3
Plasma membrane	64	46	38	66	15	31	25	14	7	3	4	2
Mitochondrion	3	1	2	1	3	0	1	1	0	1	0	0
Cytoplasm	0	11	4	4	5	14	8	18	1	1	2	4
Endoplasmic	0	0	2	1	0	3	0	0	0	1	0	0
Cytoskeleton	0	0	0	0	0	0	0	1	1	0	0	1
Nucleus	1	5	5	6	7	4	8	7	1	0	1	0
Cytosolic or nuclear	0	1	3	1	2	2	3	4	0	0	0	0
Endoplasmic or Mitochondrion	0	0	0	0	0	0	0	1	0	0	0	0
Peroxisome	0	0	0	0	0	0	0	1	0	0	0	0

### Phylogenetic Analysis of CTL Genes

**Figure 3:**
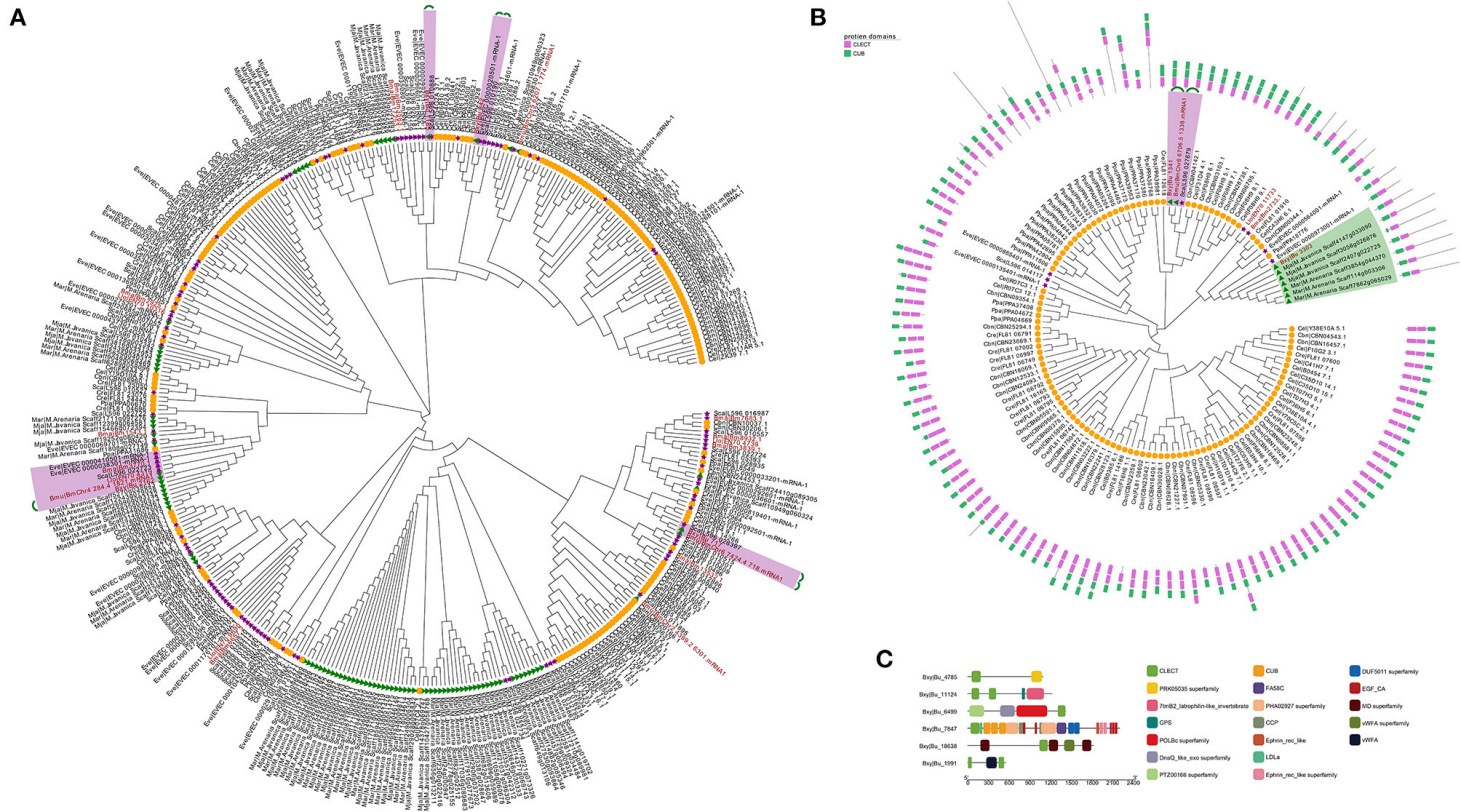
**(A)** Phylogenetic analysis of type I CTLs. The amino acid sequences from 12 nematodes. The putative 1:1 or 1:1:1 orthologs are connected by green lines. **(B)** Phylogenetic analysis of type IV CTLs. The amino acid sequences from 12 nematodes. The putative 1:1 or 1:1:1 orthologs are connected by green lines. **(C)** Schematic structure of CTLs in *B.xylophilus*. The putative domains are indicated in different color boxes.

To elucidate the evolutionary history of nematode CTLs, phylogeny analysis of five protein types from 12 sequenced species was performed using maximum likelihood (ML) methods. Among them, the members from type I contained one CTLD domain, which identified 401 sequences in this study. In the phylogenetic analysis, Bxy|Bu_1183 shared 1:1 orthology with Sca|L596_010888 and Bxy|Bu_842 shared 1:1:1 orthology with Eve|EVEC_0000020501-mRNA-1 and Sca|L596_010190. In addition, Bxy|Bu_2162 and Bxy|Bu_717 shared 1:1 orthology with *B. mucronatus* while being in the same clade with several CTL genes of animal parasitic nematodes (Figure 3A). Type III members contain 1–3 CTLD domains and 1–3 CUB domains. CUB domains can mediate protein–protein interactions in various extracellular proteins and are involved in a variety of major biological functions, including immunity and development, as well as in various cancer types (Gaboriaud et al., 2011). Such CTLs are very rare in parasitic nematodes. The phylogenetic tree showed that Bxy|Bu_1341, Bmu|BmChr6_6706.5_1338.mRNA1, and Sca|L596_027679 were 1:1:1 orthologs, whereas Bxy|Bu_3303 was in the same clade with CTL genes of PPNs (Figure 3B). The phylogenetic analysis of type II and type IV members is attached in Supplementary Figure 1. Phylogenetic analysis of CTL among nematodes shows that these genes are evolutionarily conserved. Interestingly, these CTL genes were not species-specifically expanded or contracted, and these genes from the same species were not clustered together but nested with the genes from other nematode species. Therefore, these results suggested that the reduction of these genes is a common phenomenon shared by insect-vectored nematodes investigated here, and may be associated with parallel adaptation to live inside the insect vector environment rather than adaptation among specific nematode-host species pairs.

To get a better overview of the characteristics of the complicated structure of CTLs, we further analyzed their sequence features in *B. xylophilus*. The CTLs show surprisingly high diversity in protein secondary structure and may have the potential to generate immune specificity. These proteins contain additional CTLDs, extracellular domains (CUB), low-density lipoprotein (LDL) receptor motifs, EGF-like domains, or VWA. Nine proteins contained a signal sequence and were thus secreted, and three proteins contained a transmembrane-spanning region (Table 2). Previous studies have considered that these secreted proteins may contribute to both recognition and antimicrobial activity (Nicholas and Hodgkin, 2004; Schulenburg and Ewbank, 2004; O'Rourke et al., 2006). In addition, Bxy|Bu_11124 had two CTLDs, one G Protein-Coupled Receptor (GPCR) proteolysis site (GPS), and one 7tmB2_latrophilin-like_invertebrate (GPCR), indicating that Bxy|Bu_11124 might be anchored to the cell membrane. Latrophilin, also called lectomedins, belongs to adhesion GPCRs. GPCRs are important regulators that couple cellular adhesion toward intracellular events (Langenhan et al., 2013, 2016). Bxy|Bu_7847 had a very complex structure, which contains CTLD, CUB, LDL, EGF, complement control protein (CCP) domain, and ephrin-receptor-like domain (Figure 3C).

**Table 2:** Subcellular localization of *B.xylophilus* C-type lectins.

	**Genomic**		**Proteomic**
	**SignalP**	**Transmembrane**	**Secreted protein**
Bxy|Bu_1185	√		
Bxy|Bu_842			
Bxy|Bu_2162	√		
Bxy|Bu_717	√		
Bxy|Bu_3303	√	√	
Bxy|Bu_1341	√		
Bxy|Bu_11124	√	√	
Bxy|Bu_1991			
Bxy|Bu_6499		√	
Bxy|Bu_7847	√		
Bxy|Bu_18638	√		√
Bxy|Bu_4785	√		

### Structural Conservation Among Nematode and Insect-Vector Lectins

**Figure 4:**
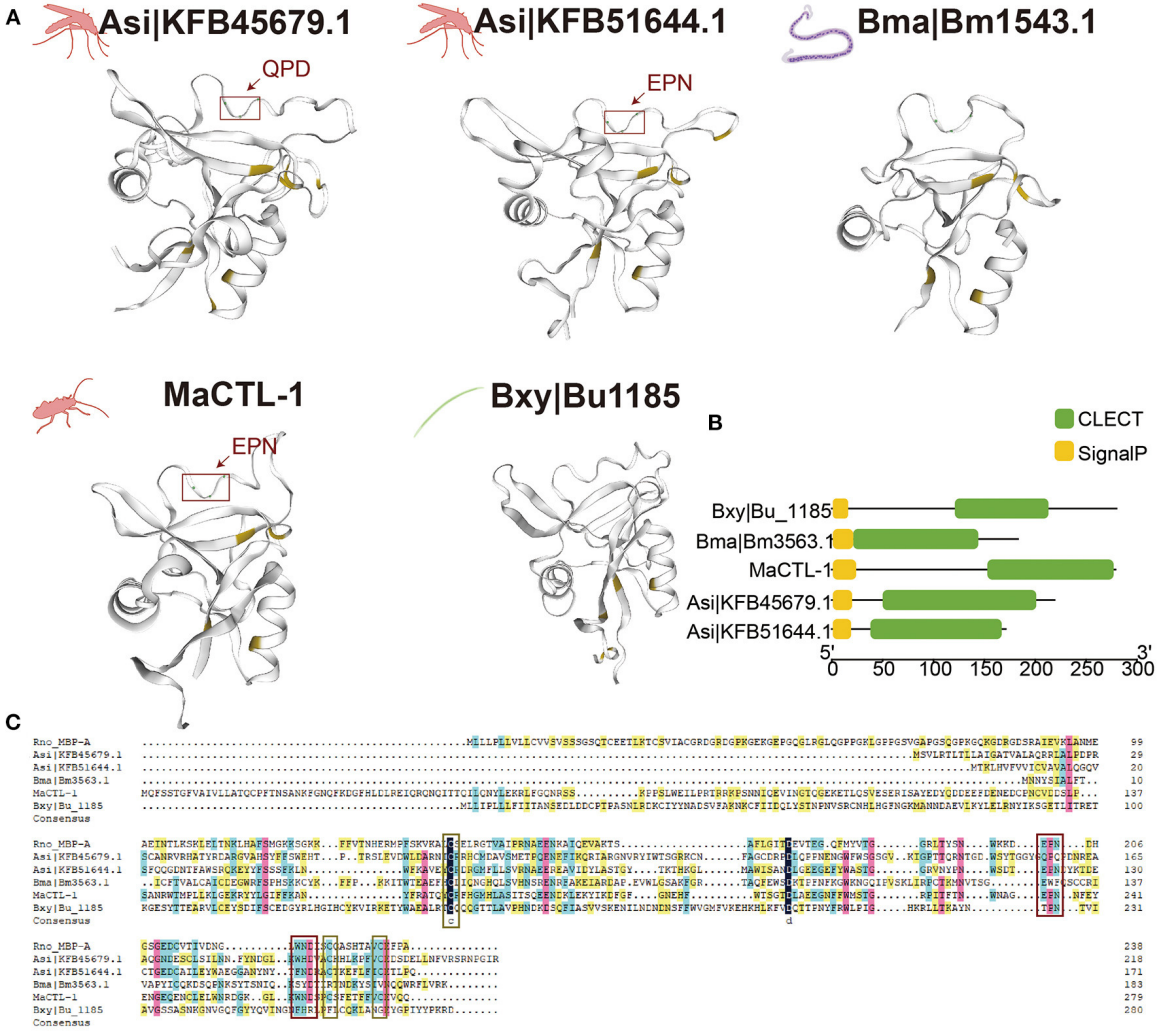
Similarity of nematode and their insect-vector lectins. **(A)** Structural model of the carbohydrate-recognition domain (CRD) of Bxy|Bu_1185, MaCTL-1, Bma|Bm1543.1, Asi|KFB45679.1, and Asi|KFB51644.1. Cysteine is shaded in yellow, and carbohydrate-binding motif is underlined in the red box. **(B)** Schematic structure of CTLs. The putative signal peptide (SP), CTL, are indicated in different color boxes. **(C)** Alignment of the amino acid sequences of Bxy|Bu_1185 in *B. xylophilus*, Bma|Bm1543.1 in *B. malayi*, MaCTL-1in *M. alternatus*, and Asi|KFB45679.1 and Asi|KFB51644.1 in *A. sinensis*. Domain abundant in cysteine is underlined in the yellow box and carbohydrate-binding motifs are underlined in the red box.

We aligned the CTLs of two representative insect-borne nematodes, namely, *B. xylophilus* and *B. malayi*, and their insect vector, namely, *M. alternatus* and *Anopheles sinensis*. The structures were aligned with the known structure of these proteins and modeled accordingly (Figure 4A). Based on these predictions, Bxy|Bu_1185 and Bma|Bm1543.1 showed a remarkable structural similarity to MaCTL-1, Asi|KFB45679.1, and Asi|KFB51644.1, with the major beta sheets and alpha helices being in corresponding positions. All of these lectins have four cysteine residues, and the three lectins of vector contain a motif QPD (Gln-Pro-Asp) and EPN (Glu-Pro-Asn) which confer specificity for carbohydrates for these proteins (Brown et al., 2018). In addition, all of these five lectins had a SP but no transmembrane domains which were probably secreted (Figure 4B). The carboxy-terminal domain had strong similarities to nematode and insect vector and mammalian CTLs, including the Bxy|Bu_1185, Bma|Bm1543.1, MaCTL-1, Asi|KFB45679.1, Asi|KFB51644.1, and rat mannose-binding protein-A (Rno-MBP-A) (Weis et al., 1991). Maximal amino-acid identity was 20.35% among these CTLs (Figure 4C).

### Comparison of CTLs Between Dispersal and Propagative Life Stages of Insect-Vectored Nematodes

This approach exploits the fact that many species of parasitic nematodes have life cycles where some stages are propagative and some are dispersive (which are transmitted to a new host by the insect vectors) (Sommer and Streit, 2011). Comparing the gene expression in dispersive and propagative life cycle stages might, therefore, identify the genes and proteins used or not used by the dispersal stage. A characteristic component of the innate immune system is that some of the genes are transcriptionally upregulated after microbial invasion (Zou et al., 2007). For the model free-living nematode *C. elegans*, 104 clec genes were only upregulated, and 103 clec genes were both upregulated and downregulated following an infection with different pathogens (Pees et al., 2016). Each life stage had a specific upregulated expression of CTL genes (Supplementary Figure 2). However, our transcriptomic analysis of *B. xylophilus* and *B. malayi* with different life histories did not find the same gene expression pattern.

**Figure 5:**
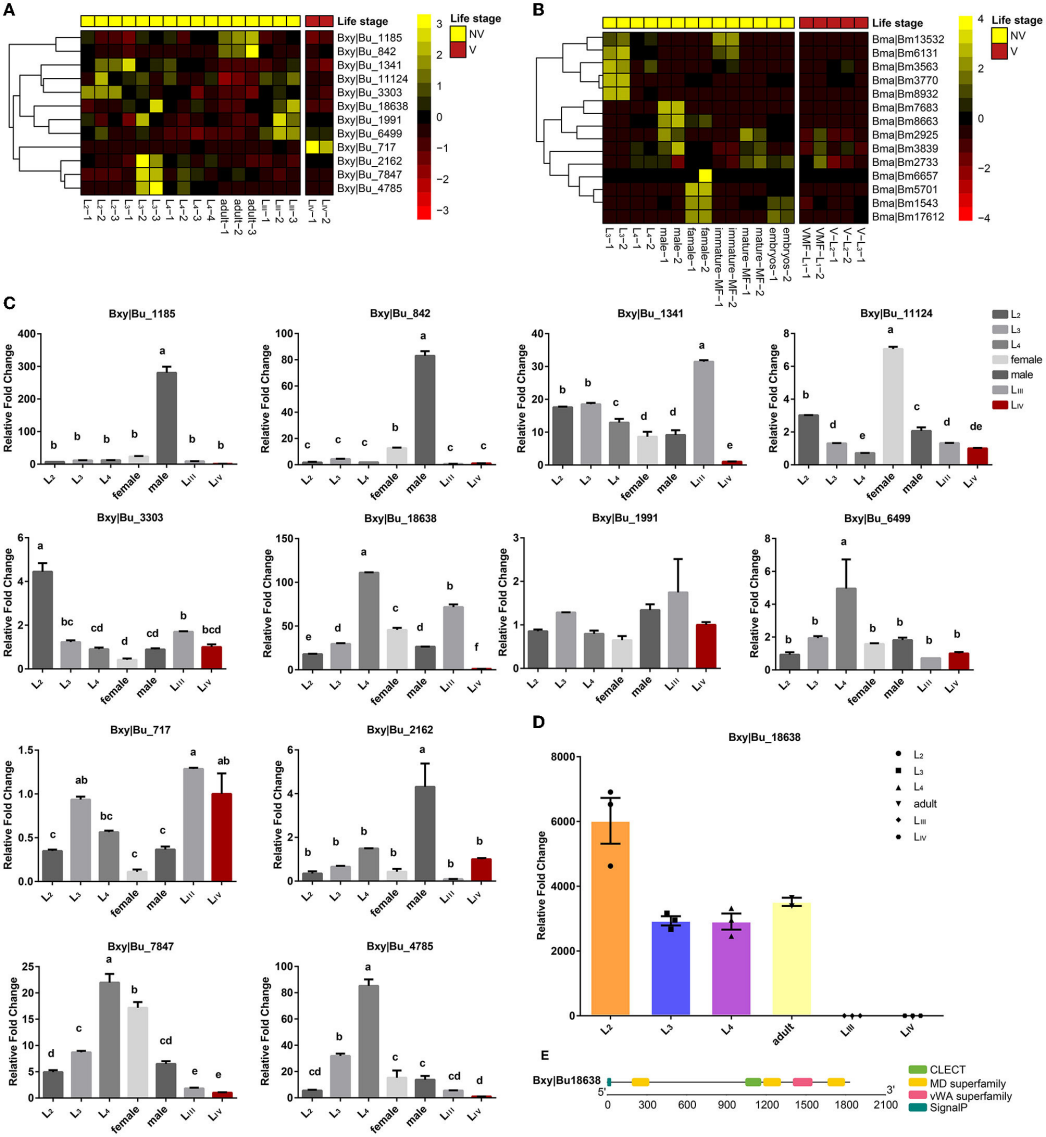
**(A)** Transcriptomic data of *B. xylophilus* CTL genes in different life stages, including vector-dispersal stage (V) and propagative, non-vectored stage (NV). **(B)** Transcriptomic data of *B. malayi* CTL genes in different life stages, including vector-dispersal stage (V) and non-vectored stage (NV). **(C)** Quantitative real-time polymerase chain reaction analysis of the *B. xylophilus* CTL genes expression in different life stages. *B. xylophilus* β-actin was used as an internal standard to normalize the templates. The relative messenger RNA (mRNA) levels are represented as the mean ± S.D. (*n* = 3). **(D)** Relative fold change of the *B. xylophilus* CTL Bxy|Bu_18638 protein expression in different life stages. The relative protein levels are represented as the mean ± S.D. (*n* = 3). **(E)** Schematic presentation of the structure of Bxy|Bu_18638. The putative SP, CTL, or CRD, MD superfamily, and vWA superfamily are indicated by different colored boxes. Different letters indicate statistically significant differences.

Besides the propagative stages (L_1_-L_4_), *B. xylophilus* has two dispersal life stages, namely, the third- and fourth-stage dispersal juveniles (L_III_ and L_IV_) (Futai, 2013). L_III_ larvae are attracted to mature vector-beetle larvae and aggregate around their pupal chambers, whereas in the next stage, L_IV_ larvae are only attracted to the newly enclosed adults which carry the nematodes in their trachea to new hosts trees (Zhao et al., 2007). Comparison of the transcriptome of these dispersive and propagative life stages of *B. xylophilus* showed that CTL genes are not activated in the L_IV_ stage except for Bxy|Bu_717 (Figure 5A). However, most of these lectin genes are activated by *Stenotrophomonas maltophilia*, which is one of the most dominant bacteria in *B. xylophilus* (Supplementary Figure 3), indicating that these genes may be related to the pathogen recognition in *B. xylophilus* (Cheng et al., 2013). The CTL genes of *B. xylophilus* were taken to perform qPCR experiments to validate the transcription level, and the results were generally in agreement with that from transcriptomic analysis (Figure 5C). *B. malayi* is a filarial (arthropod-vectored) nematode and one of the three causative agents of lymphatic filariasis in humans. The development and replication of *B. malayi* occur in the two discrete phases, i.e., in the mosquito vector and in the human main host. Both stages are essential to the life cycle of the parasite (Edeson and Wilson, 1964). The mosquito serves as both a biological vector and an intermediate host, and is required for the developmental cycle and transmission of *B. malayi*. Once the mosquito feeds on human blood and ingests microfilariae (MF) of *B. malayi*, the MF develop into infective larvae (L_1_ to L_3_), which is then transmitted into humans when the mosquito takes another blood meal (Sommer and Streit, 2011). Comparison of the transcriptome of these dispersive and propagative life stages of *B. malayi* showed that the expression of CTLs is not activated in MF and infective larval (L_1_-L_3_) stages (Figure 5B). In other words, the immune response of a vector may not always be effective because of the ability of the nematode to survive or overcome the immune defense.

Identification of the molecules that nematode parasites secrete into their vectors can help us to understand the interactive interface between parasites and their vectors. Among these CTL genes of *B. xylophilus*, we identified 6 secreted proteins (Table 2). By using the secretory proteomics analysis of nematodes of different stages (unpublished data), we found one of the secreted proteins, Bxy|Bu_18638. In addition to a CTLD, this secreted protein also has 3 MD domains and one VWA domain. This protein can only be detected during the propagative stage and may be related to the complex environment of the host pine tree during the corresponding propagative stage.

### The Vector Responses to Nematodes

**Figure 6:**
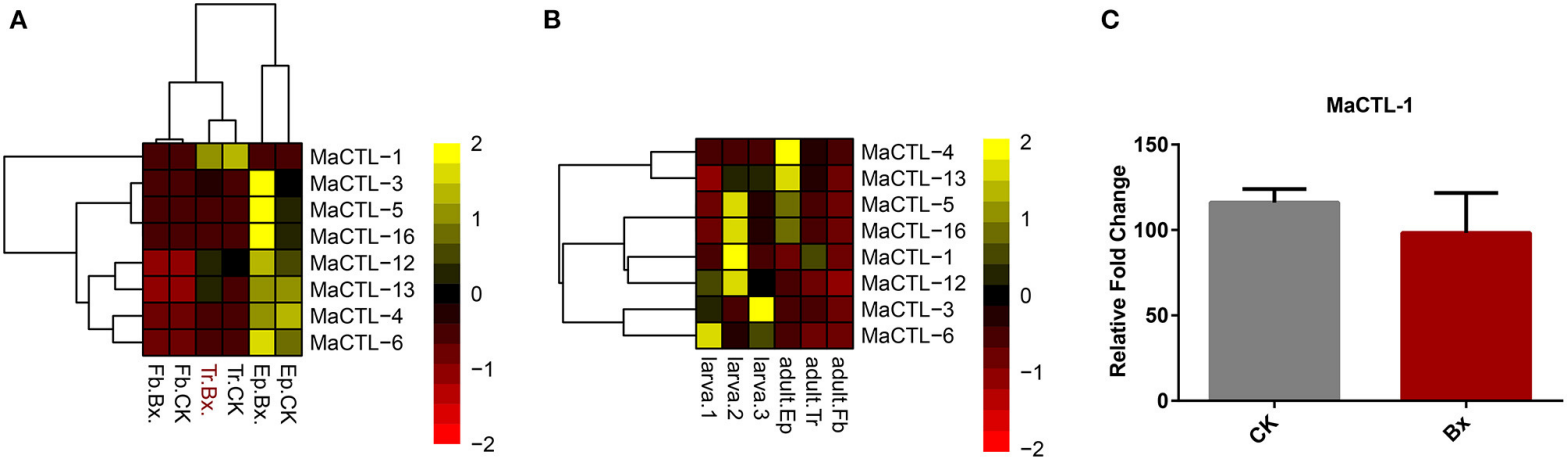
**(A)** Transcriptomic data of *M. alternatus* CTL genes expression in epidermis, trachea, and fat body of *M. alternatus* infested with numerous *B. xylophilus* compared with non-infested *M. alternatus*. **(B)** Comparison of CTL gene transcriptomic data between *M. alternatus* larva and adults. **(C)** Relative fold change in the CTL MaCTL-1 protein expression in *M. alternatus* vector beetles infested with pinewood nematode *B. xylophilus* and without the nematode. Relative protein levels are represented as the mean ± S.D. (*n* = 3).

The insect-vectored nematodes showed high specificity with their insect vectors (Kariuki et al., 2010; Zhou et al., 2018b). The successful boarding and transmission of parasitic nematodes depend on the immune recognition of the insect vector during parasitic invasion. This step is often mediated by pattern recognition proteins (PRPs), including peptidoglycan recognition proteins (PGRPs), β-glucan recognition proteins (βGRPs), CTLs, and galectins (Brown et al., 2018). Previous studies have shown that the vector beetle, i.e., *M. alternatus*, has fewer CTL genes compared with five other insect species not vectoring nematodes (Zhou et al., 2018a), but whether these genes trigger a response during nematode loading has not been studied. Therefore, to analyze whether CTL genes play a role in carrying *B. xylophilus*, a transcriptomic comparison of the vector beetle was performed with or without *B. xylophilus*. We found that *B. xylophilus* loading barely affected the transcription of CTLs of the insect vector, neither in their tracheal system, nor in epidermis and midgut (Figure 6A). In addition, we compared the CTL transcription between the larvae and adults of this vector beetle and found that the gene expression in the adult, especially the trachea, was not activated (Figure 6B). Similarly, the relative content of MaCTL-1 protein, which has high homology to *B. xylophilus* CTL, was not significantly different in the trachea with or without *B. xylophilus* (Figure 6C).

## Discussion

This study found that CTL family contraction has likely contributed to the evolutionary transition from self-dispersing to vector-mediated dispersal mode in insect-vectored nematodes. CTLs are the most abundant lectins in *C. elegans* and are important for nematodes to recognize hosts or pathogens (Loukas et al., 1999; Loukas and Maizels, 2000; Zelensky and Gready, 2005; Bauters et al., 2017; Brown et al., 2018; Zhang et al., 2018; Zhuo et al., 2019). The size of this gene family was drastically reduced in insect-vectored nematodes compared with those of self-dispersing nematodes (Figure 2). The activity of these genes was also downregulated in the dispersive life stages, i.e., stages that are transmitted to a new host by the insect vectors (Figure 5). Such changes may contribute to the insensitiveness of the nematode to their vector and vice versa: lower expression or gene number of CTL genes may potentially prevent or decrease the immune response of the nematode when entering the vector and, consequently, reduce the likelihood that the vector mounts an immune response against the nematode. Desensitization toward the vector may, therefore, be the first step in the transition from self-dispersing to vector-mediated dispersal. Interestingly, however, insect-vectored nematodes retained major genes with complex domains, which may be a strategy to compensate for the gene loss by increasing their functional diversity. This “less-is-more” molecular evolution strategy may, therefore, play an important role in the establishment of new interspecific relationships.

Our results also demonstrate a parallel evolution in CTL gene family sizes among insect-vectored nematodes. Gene family expansion and contraction can be used as a reliable index of adaptive evolution (Temperley et al., 2008). Similar evolutionary pressures, such as environmental changes and establishment of new interspecific relationships, can lead to the development of similar biological traits among phylogenetically distinct species. For example, previous studies have found contraction in the MMP12 gene family among both cetaceans and manatees. This gene family codes a potent protease-degrading elastin, a key protein necessary for the elasticity of vertebrate lung, indicating that the shared loss of MMP12 among these two lineages is likely a result of adaptation to aquatic environment (Sharma et al., 2019). Similarly, the reduction of the CTL gene family may be a driving molecular force behind the parallel evolution among nematodes adapted to vector-mediated dispersal.

Nematodes have developed a range of morphological, behavioral, physiological, and molecular defense reactions to survive and complete their life cycles within an insect vector. Correspondingly, vector insects have also evolved a variety of strategies to tolerate nematode invasion. Studies have compared CTL genes in nematodes and their vectors and found that these genes are largely identical, which may be due to the adaptation of the nematode to its vector hosts. First, gene loss events have not only occurred in the insect-vectored nematode, but also in many vector insects. For example, the size of the CTL gene family in insect vectors of *B. malayi* and *B. xylophilus, A. sinensis*, and *M. alternatus*, respectively, is also reduced (Zhou et al., 2014, 2018a). Second, we found that the CTL genes were not activated in the dispersive stages of these nematodes or in their insect vectors. However, these genes immediately activate once the vector is carried with intolerable nematode or the nematode loading into unmatched vector (Kariuki et al., 2010; Zhou et al., 2018b). Third, we found that these nematodes and their insect vectors had conserved lectin structures, suggesting a presence of a co-evolved nematode-vector immune-recognition strategy. In fact, many biological characteristics of insect-vectored nematodes have been degraded due to the establishment of a mutualistic relationship between nematodes and their vectors. For example, in order to reduce the likelihood of triggering its vector's immune response, pinewood nematode *B. xylophilus* molts before entering into the vector trachea to remove most of the symbiotic microbes adhering to its body surface (Zhang et al., 2021). In addition, the nematode's oral orifice is degenerated and forms a closed body cavity, which may prevent the bacteria and/or secretions from flowing out from its intestine into the insect vector (Zhang et al., 2021). These results suggest that both nematodes and their vectors reduce immune recognition and defense against each other to balance their mutualistic relationship. This “retreat in order to advance” strategy can reveal insights into possible mechanisms underlying adaptation and deepen our understanding of coevolution.

## Data Availability Statement

The datasets presented in this study can be found in online repositories. The names of the repository/repositories and accession number(s) can be found below: National Center for Biotechnology Information (NCBI) BioProject database under Accession Number PRJNA798902.

## Author Contributions

LZ designed the research. JN performed the experiments. JN, JZ, HW, and YL analyzed the data. XF, YF, and XG collected samples. JN, JZ, FA, and LZ wrote the manuscript. All authors critically reviewed and approved the manuscript.

## Funding

This study was supported by the Basic Frontier Scientific Research Program of the Chinese Academy of Sciences from 0 to 1 (ZDBS-LY-SM027-03), the National Key Plan for Scientific Research and Development of China (2021YFC2600100 and 2019YFC1200504), the Natural Science Foundation of China (NSFC 31970466), the Strategic Priority Research Program of Chinese Academy of Sciences (Grant No. XDPB16), the National Youth Talent Support Program of China (Ten Thousand People Plan), and the State Key Laboratory of Integrated Management of Pest Insects and Rodents (Grant No. Chinese IPM2111).

## Conflict of Interest

The authors declare that the research was conducted in the absence of any commercial or financial relationships that could be construed as a potential conflict of interest.

## Publisher's Note

All claims expressed in this article are solely those of the authors and do not necessarily represent those of their affiliated organizations, or those of the publisher, the editors and the reviewers. Any product that may be evaluated in this article, or claim that may be made by its manufacturer, is not guaranteed or endorsed by the publisher.

## Supplementary Material

Supplementary File 1 Genome datasets used in comparative analyses.

Supplementary File 2 Amino acid sequences of CTL genes in nematodes.

Supplementary File 3 Amino acid sequences of CTL genes in vectors.

Supplementary File 4 Transcriptomic datasets used in comparative analyses.

Supplementary Table 1 Primers used in quantitative real-time PCR analysis.

Supplementary Figure 1 Phylogenetic analysis of CTLs. **(A)** Phylogenetic analysis of type II CTLs. **(B)** Phylogenetic analysis of type IV CTLs.

Supplementary Figure 2 Specific upregulated expression of CTL genes in *C. elegans*.

Supplementary Figure 3 Transcriptomic data of *B. xylophilus* CTL genes under *S. maltophilia* compared with aseptic nematodes.
