# Supplementary material for: Parallel Evolution of C-Type Lectin Domain Gene Family Sizes in Insect-Vectored Nematodes
Source: Front Plant Sci. 2022 Apr 25;13:856826. doi: 10.3389/fpls.2022.856826 (PMC9085898; doi:10.3389/fpls.2022.856826)
Supplement: Supplementary File 1 — Genome datasets used in comparative analyses. [file Table_1.DOCX]

Supporting file 1. Genome datasets used in comparative analyses.

| **Species** | **Source** | **Data version** |
| --- | --- | --- |
| *Caenorhabditis elegans* | ftp://ftp.wormbase.org/pub/wormbase/parasite/releases/ | PRJNA13758 |
| *Caenorhabditis brenneri* | ftp://ftp.wormbase.org/pub/wormbase/parasite/releases/ | PRJNA20035 |
| *Caenorhabditis remanei* | ftp://ftp.wormbase.org/pub/wormbase/parasite/releases/ | PRJNA248909 |
| *Pristionchus pacificus* | ftp://ftp.wormbase.org/pub/wormbase/parasite/releases/ | PRJNA12644 |
| *Brugia malayi* | ftp://ftp.wormbase.org/pub/wormbase/parasite/releases/ | PRJNA10729 |
| *Loa loa* | ftp://ftp.wormbase.org/pub/wormbase/parasite/releases/ | PRJNA246086 |
| *Steinernema Carpocapsae* | ftp://ftp.wormbase.org/pub/wormbase/parasite/releases/ | PRJNA202318 |
| *Enterobius vermicularis* | ftp://ftp.wormbase.org/pub/wormbase/parasite/releases/ | PRJEB503 |
| *Meloidogyne arenaria* | ftp://ftp.wormbase.org/pub/wormbase/parasite/releases/ | PRJEB8714 |
| *Meloidogyne javanica* | ftp://ftp.wormbase.org/pub/wormbase/parasite/releases/ | PRJEB8714 |
| *Echinococcus granulosus* | ftp://ftp.wormbase.org/pub/wormbase/parasite/releases/ | PRJEB121 |
| *Bursaphelenchus xylophilus* | https://www.ncbi.nlm.nih.gov/genome/ | PRJNA604491 |
| *Bursaphelenchus mucronatus* | https://www.ncbi.nlm.nih.gov/genome/ | PRJNA562094 |
