# Supplementary material for: Parallel Evolution of C-Type Lectin Domain Gene Family Sizes in Insect-Vectored Nematodes
Source: Front Plant Sci. 2022 Apr 25;13:856826. doi: 10.3389/fpls.2022.856826 (PMC9085898; doi:10.3389/fpls.2022.856826)
Supplement: Supplementary File 2 — Amino acid sequences of CTL genes in nematodes. [file Table_2.DOCX]

>Bxy|Bu_scaffold100_cov122_len1285729_144.3_1185

MLLIPLLLFIITANSEDLDDCPTPASNLRDKCIYYNADSVFAKNKCFIIDQLYSTNPNVSRCNHLHGFNGKMANNDAEVLKYLELRNYIKSGETLITRETKGESYTTEARVLCEYSDIFSCEDGYRLHGIHCYKVIRKETYWAEALRTCQQQGTTLAVPHNDKDSQFIASVVSKENILNDNDNSFFWVGMFVKEHKHLKFVDQTTPNYFRWLPIGHKRLLTKAYNTPNTVIAVGSSASNKGNVGQFGYYQVINGNFHRLPFLCQKLANGEYGPIYYPKRD*

>Bxy|Bu_scaffold100_cov122_len1285729_217.5_842

MKVLLCSAWFDALPITITWKLRCSQIPYRSAYISGYCFVYKDAGHYNCSTVKETASGLYAPKLVSLSSVEERIVSHLKKINIFSSVNEQDLCVLNMGFRCPSGYDLFEDFCYKAFKDKKTFAEARSVCQSDGGDLPEVHSDTVNARLAEYVYYNTKSPIWLGIHIIGSYWNYDRKRVNNNHNHEQPYYFRWMFQDYGNTHAVLDYGIYAGYWRSLKPTESHSFVCQIRAY*

>Bxy|Bu_scaffold11_cov92_len918884_265_11124

MRVVLRLTLFLHLAYSDLVRRCEDKDLCQHGGECFLTAARQSSAKCPDGRYSEVDESCLHYSPIKKTFNDAYNFCRTRYDGHLVWLESKEKRAFVEEWIGKSAWIGLQRDGQSKARWHPGGEATMMDVEDGTAEKSGTHCGVLYHNHGQPKILTEFCDESHGFVCESNISRFKGADDWIAQCSCPSGYGGRQCETKLAAEKTENSSDKELCGIRRFEESCQENEVLMIDYAIYGYSPLLKYDGLCPLTSNLDHDCIESAGLLKLAQRCQYRQKCVIEDLAKFFENPTCSNGARLKYRVRCVEKKELACPHGMKILGDRCIAISDNKEKKTYADARAECTQKGGDLAYFDPSIDFTSILKGITPLPSSEYWIHNSSEKTNHFGRRKSECLTASWWSPNVGQVPCFAMLGWICEFVPNGIADRVLVDKLNQRKHIEEDTKDRSGEKDKENPRFCKDFKFHGQKIPETKACYDFEAECPQGLSGKIILTCDCASADWTGLEDHSRCMSPSLSALKDLVRTDDPFITVGKYEHVIYNLMGNSKLTSGDIFGFLESGDSMMAMALQRLEKEANAERKKIYSKKFAKSIGNTGDYLMDKRAEKAWYNMTDDHRVARAARLMQMLQNVLVLQSHGMANGSEDFKFSHFASKLEVKPEVQYAPGTPSIRIAQDVPEPETVSFDGFQKAASMEMPSSDLLSFAVKWSHDSKQMSGGLIPRMRTLNPSSNPPQADNTLKVGHFMFSNLGNLMSSTTSKRRVNSYIMGAFVNDPAISVDLMNEHVSFTFYHLVKDGVKNPKCVFWNLTTSSWDDKGCEMSFTNDEYTKCTCSHLTSFAILMDFTNSLGELRGREDGFLPAKFVDETLDWLTIIGCALSIISLTFCVLIFTIFRSLYNSRTTIHRNLCLSLLMAEIFFLFGIEETEPAILCSGVAIALHYFFLCAFAWMLLEGYQLYLMLIKVFDARSRTLWYYCCGYLIPAIIVLLSVLVAAENYGTANYCWIDVTSPTIWTFVGPIGLIVLFNSGVLVLALRTVLAVRNRDRSLAAKVLGWLKGSAMLLFLLGITWVFGFLSAIHVLQPYVAYIFTILNSLQGTFIFFLHVVMNDKARQCVLRCLKEGVCCQSPVTSSSHSKNGIRPDSSSTFNKTNNFFALRKDKILQWWPLKSSASNSKNSNSNEENRRSSEVASPPPSLKTPKEVESPRESDSKEEEDETVTRRRLLSPVREATVTVERF*

>Bxy|Bu_scaffold825_cov96_len158809_16.1_18638

MWPPGAGTSILALICLFAACSTQEIDPAVAKFAEQQQLLLKQFKADKSLRPDAFHGTPPNNTVTRRKREIKEEHPEGPEHCPPGYTGHLCESPICWNQTRLDHHSGDFPGELIELDFFDQCIGGLVVHVDRHIKDLVVVVTTEQAGYPSGKLFNAHGRLLFPNFEEIHPHLRQMHYENVQDEGTGKFYFTADSDDNEDCVVAVRAHTDLVLDGGFVESPHDDRVQEFVGEYHELQRDPTEFVPGYLAFKPSRLEFPETVTTVTFYRSQHEQQYAPMLVGTRYNCNANVFAGPYVCLEYGIYFVKIRGTDRGGNVWQRVYQFACNANPGPGPGPGPTPEPTPAPDHCLNGGTFVNNETHSYCYCGHHYEGRICETKILLGIDETLEIGAANFYRNSPIIVFTDALPDDPPSTRFDILQRLNEQSNPIYIIAYGGLGFKCNPDIHTEAYEQFRVLAQFSGGLVVKATETVDEHLSWIIWRVAYTLSVGLYNEHLLAANDLLDSCQYAPNEQVFFVDDSIEELIVAFVGRADYDVNVVNTIEEEVDAFYIMKGGDLTNKVYRNLEFGNYRLVIKKGQNSSETELPCHYRVYAKSKYEVFTGASIDIDHDLTLDQPRYNEEMHIVARVNNIEFPDPENVFAEALIWHNSIFQHDKREVLYASNGVYRDGCDYNLYFGTWKCERPNQFFYVEVYVEDATGLTVKRTSVGHCTVRDPNPPDQGCRNGGVRSGDRCLCVPGYEGKNCENIVCYNEGRSHSTHCECYAPWSGPHCTEPKCGYKNQWVDFRPRGVGLSFVIQGSSDVQSTLVELNRLAPEIVRDFKFNDPYWISHYSIHSFDDKQVKLYLQTDHPDDLIHGFEALQQDTQNTESVCKDLLIYEAIIEALTSSRATAHEVVYLVVSGGIKLNEHKEKETFQLIDALGAKINIIQYPINKCRIEIDQEASTAISTLAHYSGGSLYQTNRGTAFITLPLQYDEATVFDEIRDNCDKEQTFYFPISGNAQTVSITSLGDLRRGYPKISAAENTYVDARKVYDSAMSRLNIVYKDCPDGWQPYEQNCYLLEVREIPWDDAKATCMMQGGHLLHIANKGLDDLMSLYIGGTPTWIGLNDQNSHDWGWDQGAHDDLPLGNAFKHWAAGEPKSGKHCAILTSGGWVSEACNATKPFICQKHAYDLGYNPGGKEHGHLARGIWSVKLRTYNNEFTTCGIRVNVQSATQVFRQFTKKLDDDFGNGALVWKSKNNRIIAHLESHVNGHSSEARLEYAHLYPSETANLGDVVAFHKRDQCSYDYISNPFTASNLGYYVGFTGFDAYGYPFQRVLPAVSEDSIPTCKNGGVLSRRTEECVCPPEFRGRECQIPNCKFGYAAPNGVSCKCFHDYEGPLCQYAICLRNDTDHPPPPMDVYGKSFIIVLDGSNTANNAKVAQNFNKIIAEILDRLGNTEHQMYHNYIGILAYDKAQGEVEPVSKLVVETDRSKFLNELQTLINDNYQSRGQKRDYLFALAKVISSKDVIPGSAVYVIGDSGVEDAKTWNDQLYNHIAEKHVTIHTIILNDQVPPGNASNYRDPSIQHLLGLPFVTDGFIYQVDPDRFKDLFYIQLGSRFRGYSLTHQMYRECSDKIEYFQTGGDYGLLVVDLFTAHMDLEFDVIDPNGNSTDKAEHLFLTGTNVLFTIETNIPGIWTIQIHHKNKPSACFVSVREVANTAPAIGFNTDIRGDNGLHSRSAQYYPQDGLNAIIANSDTDHLTYAQVYTHDAHTLAFASPLVRRHDCAWNYISSEPFKCPASTFTVAVVGYDRGGHPYRRTYKTHCVGYKGPRTPTLTAFSDEGTSLDFPIGLAQF*

>Bxy|Bu_scaffold2034_cov104_len350705_270.3_2162

MGSPISNILLFLCVSVYADYENKLIIDFNNPPPSRRNSRGPVRDDAGVLNEVKEIVFRMEHVFNERIKNLSDRVSRLENRQILHDGDPHIDWKPLGTNGKKIKVFSTEATWSDAKRTCTSHGGKLLEIQSDNENRKITDYLQNFETNLFWIDAQVSVSVRLPAGRYQNFQNNRTEKDCTALNVLGKWVRMDCTERHGFVCQF*

>Bxy|Bu_scaffold26_cov103_len1649090_1086_3303

MQHVFLLDSCPYLLLFVVTLSLTAFAQNGPQPCPENAIVLTGSDYPQFVYSPYDELRQYPPNTDCRFVLVARSMQHRIHLSVIESKLEEPLFTECEDYVSIRDGGLPTSDEVLRWCGSYFPPSLTSSGDSLYIHFHSDNIVQSRGFNLSFVDYLIPGCPADWIRSDDNMYCYKMFSSAHGYTFIDAQRNCFYERSNLLTIENAREYQFIVGKYSDSHKFPWIGYNDANKEGEFDAIDPNVPIWPEDLSSSLRGDHKDKDCLFLDWNSIKDGPAHTVDDCRTRHSYICKKRQDGTTVPIQLSATLLRHGFEDPVFNYTLILLILLLLLVLLIILWIIYQKCKKRNVVAASEGSRLVQSAPHPDTTARAATAAATINLAPVPEEKKKKERAKTAATDAVKNVLAISASRTTEARDLALQKTDDDQTFQLDDTLFEKSKPEDNAVAATNYQQVSAESPRIRTSPNQAAPPVQDSEFARAQRRDSNPVLSDESEAELGVGETPIPFSPVKGDKASPAVHSPPDRSHLYESGPVTVTTKDETKDAKDSGVTSKTLSSTLPPVGEPSGPSREATIQTGITAPSSHRSRGTQPSHGFDRPHVGTLENVSAISLDEFWQESK*

>Bxy|Bu_scaffold301_cov119_len910634_867.3_1991

MNALLCPFGWHYYQDKCYFLTISNSMTYDNAEHECNTRYGGNLATIQSEGEDQFVHFLKESSHKHPAFLNKDEIRINLCFQSIENTLKISTETIILNNIPSSSLCKQECVRLRFDLGFECNSVVWISNENECLLNIGNITKNVIVSEELQYYEDSCFDKPSEEPSDIPLAMPVSQVRNCFAKTRFATLQGVVGKVYDRISLNDCLGECWNCEDCFEENKCQTAIYYEEDAMCIITNVSAKITRERVVKEDMSSLYEKRVKCLENNCEDLEVHLVFGIDPAVYNETITKQIMIEIFSSVSRITEFVRVTAVLLSSSPSNLFDDLQYENENDFKFKLETLKVSADSSDFEAGLIEFLNGVDEHWTTTDDVDVTWVLVLTDTAFQSRLGLFLDLKEGFRRFPLFSIGVGENLDFEKLEMIASSVDNIVHIQDPVTIGKALAPRICHQKPLRRFVTSRKLKKTKTDHSFTWLGLHRNDYGKMEWSDKSVFGEYENEILKYLSVTQEDGDCILRKGTTNWAFANCTEKHNFMCTLEPRNKLEDIIKRRLNIKKLY*

>Bxy|Bu_scaffold30_cov118_len1940649_1786_6499

MKLDDGGAIIDVAFSSCFLICILTFICSFMLSEYRICNVSCDYYLSEQTPYSAGNRSELPVIRVFGIDKNGQKCCAHIHGTSPCIWMRTNCQLSSSQRLLLSQTLSQRLQNAGIQLRADPIIEISECKARSMYGYYDNKDYFIRISLRNPVHTRTLTKILQDEALHDFKLQPYMSHIPFILQFFIEYSIFGMGQIGFRKVLFRKAPNSSSGYNLSSLPPASNMSVEFDAHVEDILNPWEEVNCKYINSGLEYIWNDEEHRRKLLKIPMFRPGHPSINTFTIAKNERMNLAKLRDKVTDAATNVPDTQKIKLSDLPPFYDNLSFDPDAVRTDIEDAEADDESQHDESIHDELSEMSSTSLSDDETGYSEMIGPFKDVDDFVGWETEGINTQFFEVSNSYEKKVTLQKIKKAREKKAKDPGDIKQNDISQIRSFEYCGNLRDVTIYTRLIKRHNHNVSPVKGSVKIQSSSTVDNLCVMAVELIADPVSNSRVPDPASDPVMVLSCALCTDISNWTSKTPFSYIRSIVVRGGVSCGPTRDVDYVDSEMELFQKFAEIVQRFDPDILVGYDLHRESWTYLERRTSFTKHPLCKMISRVKDVSIFEKSGAGIDAGRIKLEVWKTARRDSPMRSYEFGYVVGQVLDLPFMELDFEMLKELSNSTNPDCKLLLVDICMKRAQYDIQILEKLDIFVKTSQMARVYGIQFIEVLTRGSQFRVESMLLRLARKRGMIPPSVGVKQRSLMGSPETIPLNLEPESGIYRDPVVVLDFQSLYPSVCIAYNYCFTTCLGKMSRLLNADLGDQIKLGALSYTSLTSDKLKELIDNDNIHISPTGGVFVKANVQKSMLAEMLMELLDTRVMVKDSMKNFKGDAILRRTLDAQQLALKLVANVTYGYTAANWSGRMPCEEVADAIVSKGREALEGVIHMVDGNAERYKHAEVIYGDTDSVFVLFRGCSRNEAFKLGRLIAEDVTKMNPNPMKLKFEKVMQPCILVTKKRYVGRSYEKEEDAGVFDAKGIETVRRDGSLFVSQMLEKCLNILFDFGQTAVIRYLQSKLANPESFPLSSFIINAEYRGEYAEKAQVPAKKLAMQRKEMCERFEPIRGERLPFVIIRPEPHSKTKMIDCVVGWEEFLKDSCLQLHYNYYMGKQLMSALTRFFDLTPYGLRYHSLYFDICQSCGKSTNDSEFCEDCSEDDNVDQIRLAKYFVLQRRLLRTYRSCKNCIKMDTVDIDVRNLKCFNFNCNINNTRLKLERNLENEDDIELNDDTATPSWEEKMRTLDEKWQIRLGQQVAYLRERIEKLETTMNSLNHVMESDWNHFGDQKFKFFDRNVDWNEAQVFCRLHNAQLINVDDSQKNKIISEFLTRADPAITEVWIDLKTQTQMETDSYKYDNFSEKGLIEGCTVVDIRGKWKIRPCSRQRPFICERK*

>Bxy|Bu_scaffold37_cov107_len2094627_1113.4_7847

MGQPNMALFWAIVLYSILVQAEKSQNVTEIPYILTGVSMNCPAGWDLVGDKCFRAFNSEKSWPQALIFCERYGALLAKVESAAENEFLGRIISRPTKSSALSKHWIGLITEKSTEDEGSFVWSDGSVVSRFVGFWAKQQPDFGNGNCVQAGVNSEKLAEWQMEMCNLMSPFVCQLNACVSGSFHCGNGKCVSEKAHCNGHNECGDYSDELNCPAPHGQVACLHYEKSENGKIESPNFPSSYGPNLDCRWVIEGPINSRIMVTFDQFETELNQDLVTVLDGGPSENSTLVLDHLSGSPQSSRLSLITSTNMLTVQFRSDALVQARGFKASWRAVPFSCGGSLKAHLFSQTFSSPRPYPAGGECVWKIEAPEDQVISLNIEEFDIKPGKDQLIIYDGPHPSSPILSRLAGNLSNQLVISTQNFLYIYFYSSQADSTNGFTIHYKKGCNNTIKYNHGNLLSPGNGIIPYPASQICRYVIDLGEGMSDRPISIDFNTFDVQNDDVLKMFDGDVNATVLHEGKGFTEGRNPPKKLHSTKGKVAIVFETNAMKQANGWNLTFSANCPPLKAPKTVFLTTQSTSFGTKVTVSCQTGYEFTNGIGHRFDISCDLGGKWSNDYIPNCQPVYCSAVPQIANGYAVAATNVSYGGIAKFKCYDGFHFKSGKDTEEIYCKTEGWTETPKCLAEICPALPTFLNGERTLKFGDATGYGTVYEFSCAPGYRHNGPNTILCQSDGKWSAQQPVCKKRICPNIPQIENGRIVVPPTGALEFGDSARVECNPGFRSTGADSVKCLANQTLSEVPECRDVDECVEAIASCVTKSTKCLNLEGGYTCQCLDGFKPQLTCPSFHVLSPSLVKTSSSAIFNMSSVTFCAEKSDDKRYLEFEFPAPKVLERIRFEKTEDGVPLAISVSYSPDLKSRLRSIEDLLEIPLKNVDMAGSQVVVLKTPVEAQRVRVRLDRFKDSACARIELMGCQKTNCVDIDECEVANGYCDHQCHNTVGGFKCSCRDGYDLFVENGQGGAMLKEGETGLDNLDMIRYNKTCVPRKCPKVEAPAHGKVINLGKEFAHPNVLEFRCDHGYQIRGSPFLQCTSDGVWNGTVPTCTPAVCEGVQNNTSIGMFVTEDKPYVPYGDNVTVVCTQQNRPIKQSPLGGLRQCIYDPRPDGMDYWLSGPEATCPLVECGPPPALAGAFYEGEDANFKVGSSLAFNCRPPYSLLGKSSYDDRIARCNVDGSWDLGDMRCEGPVCVDPGHPEAGQTFLNSVEEGAVAKFTCDRPGYKPYAYDAINCTLGTSCLLSEDVGISNGYIPDGAFSDSDTKTMWGYEPHKARMSSTGWCGSKDAFIFLSVDLQRIYTLNTLRIAGVAGSGYLRGHVTKLQLFYKVQFSQNYDTYPVEFETAPGNHNKMYQFHLEPPIKARYVLLGVTEYEENPCLKFDMHGCLAPLSTAHEIPSHLQVGWNASVPQCIDAEPPQFINCPANPIFAQTDEFGQILPIKYNVPDVHDNSGVVAYVKVEPKDVQPPFHIHAPLDVKYTAYDEAGNFAECTVQLRVPDTQPPAMKCPESYSVYTSGNETSKHVVFNESTVHVVMHDSSNITEFTISPSETEINLFSAVDVEAVATDSHNNRASCKFQVALLPEPCTEASLRTDDHTIKKCSTENNQTSCIIKCKSGYRFVNSSIEEVKYSCSPSLEWSPKSHPPACVRKSEEPARYEFQVSVDYSSSIPARDECMKPYADAVANAFETLDASLTQKCSSSVQVYVRFLGARFMVDPSNNKMVKANFTVQILPTVLQEVFYELCGLTLSTIFDLKIPGATLPIKTLLQLPNGGLNAPTCMPMNAVSTKIHQGFACSKGEILNKEGTDLPECIPCAKGSVFAGSSCVLCPPGSYQDKDGQQECKKCPEGTFTRTSGTTSRSGCLAVCGNGMFSESGLIPCQLCPRHTFAGPPLPGGYKTCDACPEGTYTANLGATGPSHCKQPCKPGFFSESGLEPCSPCPLNFYQTSLGQQRCIECANNTITHEVGRAIEEECKPLECTGVKCQNKGVCAIANHKTICECRPGFTGEFCEEQVPICDTNPCLNGATCENVAGTFRCICPQNFTGSRCQFGPDECIGVNCPNGGVCQDLPGVGTYKCVCRTGFIGHDCSEIADLCQSDNPCKNGADCVPLQLGRYKCKCLPGWEGPTCERNIGGLFLVPLRDVFEIWGRNVMLGVVKSKFFLQ*

>Bxy|Bu_scaffold40_cov119_len2195569_814.4_717

MNTSLVLCVLALVLGYCSANTSPCPDGWRYSPFTSKCYKLFEDKTSWTTAEYKCAFQGAHHISVHDAADNQFVSELARQANIVWLGAVQYGTDRDYTWADHTAYGAFENWENGRRPSYHKGKKCSKFDGLSGKWIQSCCKVPAAFICSKPAAVLPAAIIEDDTGINEDFRRTRLAFRRRV*

>Bxy|Bu_scaffold40_cov119_len2195569_1517.1_1341

MTSSWLSRLTLLIGCVVAQQAPCPPGWTYKSHTNQCYLLSSINLPYNEAVEYCKKDGGSLVSLKTQDEYDYVRELYNNNTKGFYPPWIGLKRDSRWYSPVWRWSDGFVAYFTKWLKEEPTKTADNACVAWRTIENHGWKTLGCKYAQRFICKQASANCPTRNYERPEGVLSSPNFPTNYPNNLNCFYHIKVKPGYRIKLEFHDFYTENYFDKVVLYDDYNGNLTNKIDTIYGTYLFKKIYESSENVLTLNFITDHIITRQGWNATYTSVHKEPTQELHDPTGTITSPNYPKNYPNDIDQLYLISTLPDKVLNITITDFNLEKNYDYLAIQDGKDIIKSKQLTRLTGTDVKTPLSFLTSQNYALVRFFTDPSLNYRGFSLNYEAVEKY*

>Bxy|Bu_scaffold62_cov110_len994864_936.5_4785

MLRSTALAVFVLGCFCLSNAITQESLEDEDSQQTRRGYKFHNPVPATPHSPWLSGPNEKKYSFHIGQQSWVAAREICLTNNADLATISSEEELEWILSHYKPQLRHSKDRQIQVGLYAEIEEDEPMREWKWVTGEAVNSSILSWASGEPFDHANGKERCGLLNINSKTLDDVDCELGGSPLRFYRFICERTFNQHLKHEELNNPMWKKLEDILVFFGISDRTKAKNSNSTDSNESLKATPLEDEDYDEKKDLKKVNATEIKKESKVERAPVKPDEKPLKDDKIVEDKDKKLQTHSQAFAAKTIETAPTRDDSDLLVITNLEPLRDAGKFDGVTESSIRTTRPDPLAHLADTLEQAVESDAPGDDDAKMKNLERIINAVQKMVGDDDKDKVGWETVRARIHSEKRPKFGPTIESENENSNSERSNSNSERSDDPTYKSDNGNSKSENPNSKTENSHFPSENRNSKSVDPSDSSEVHHISHHDSHRHPHFSFPDIFDEILGPMLPRPFRPRPFDRPEHRDTRKWQHPDKSHLQNREMSEINRQYGQKTVESTTLIPKTGEIEGSGQDIEIPIEVFTKAKAEVSTTRPNLSNIEPHWVVRKTFYAQPHQKRKIKVTEPHRNPDRETLPESFEFDRDEPEPQEQTTKTPAEKIQFVESKQKTEIEQNAVEATEPVNPTEQFQIALSLDEDFKQAQKEQQAHPGVNSGDLLIIPKSSEEIVGCGAKNLKKESTVSTTPSPAQIEERNLHINAFLNNLRDLLNRSDSTVLSNLFEANYTDSAENLADKLERASQLARTTQSVPPSPEELERLRKNIENDVLKSIEELGETEDREFKSMEVAEKDLEAIGSGLEPPAEKLISLTPVALTAHQQIIVEKKTESPEEANVSGYTPKTTVELAKEKKTKKFFVLSSAEAAENAAETEGFLAKNEEEKPVEIEASTKQTATSIIPESEEDSAEAIEGSGQAPVGTRRTEVKEQGKQHKKPRQSLFPDLDFSLLNNKKLAEEAAESSKKQKAQADEAYKALAKLDQNSLAARIAASEAQRKKAEQKTDEIKDPIQVKKIQVQPALMKNKKPEDVAKQLEKMFFDWSNGAFGVLNG*

>Bma|Bm13532.1

MICIIKCFITILLFKGIIGRLCPEGWLYSKQTKYCYHLVTIPTTFNNAQFACLLQNAYLLSIHSEAENRFISAMFAKNGHSIWLGAAIFGNSRKYEYVDSTPFDYTNWQNGTQPIYKKSRKCVKISSENWKWFDDCCYYRPVSYVCKKLAGKYEQLYWKTIADKTSNIKKNHNHRHLKLFNYKFSNEKNNIVMKENERRNKVSNMVTSSANKIYDNKMRDKLRTRK*

>Bma|Bm1543.1

MPRFAIRTVCNLLDSEIVKIRNKEEAHTAYGLAGGKSFWLGLRRGKRDWYWDTSTYGTRAHFFFWRTGQPDITDNKNCVYAAYAGEWMTADCDNFRSPKMFVCQSPSYKN*

>Bma|Bm17253.1

EFHCLIQNGHQLSVHNSRENRFAKEIARDAPEVWLGSAKFGRTAQFEWSDKTPFNFKGWKNGQIPVSKLIRPCTKMNVTSGEWFQSCCRIVAPYICQKDSQPNKSYTSNIQKSYDTIRTNDKYSIVNQQWRFLVRK*

>Bma|Bm17612.1

MFSVPAFYLLQFNSAKYLGFTNFMPRFAIRTVCNLLDSEIVKIRNKEEAHTAYGLAGGKSFWLGLRRGKRDWYWDTSTYGTRTHFFFWRTGQPDITDNKNCVYAAYAGEWMAADCDNFRSPKMVGSRVAQNPEHRTISDVLVYLEREPVMLRALQEYMREREQIEDLLLTCLEDTERRSEVEGQDGEEPVPQEQAFYEEEAEWAPLPETNDADEAENWPTEAEWAPLPESDEETEAPVAPNAVAEPVESVRELVLRTRTINIPVLATHTSVAAAPEEFHPADLGDLPEQLRRELLVPQAEPYTVVQANEERNIVCGICGRQFATLKGWRIHASRMHKQDGKFLRSLWPLPPAATRIHGRAKESGDRSSRSRLVPESVCGRDQRKAGQAPQT*

>Bma|Bm18080.1

MKNSKMLYGKFFVKFYELYCLFTLLLLDENVESAYIGLQSVPYDVTQRYFCTGETIDYTNWLSGKPDNKNLKDNGCVILNAKNGKWIDYSCDANDELGPWATICQIKDTREEYDELKCPKGFEYCSNTKNCYKVIIPLLIFIGNNKGKGMNQTEAQNECKTYNAHLVSIDSEEENQFVSDLSMKHPLSAMRIFIGLELHQTDDISEQHWLDGTTIDYTKWDYMKPDKERVRPEHECVIMIPERHGVWSNWSCADSCCAKHAIYGAVCEIDHDDKII*

>Bma|Bm2733.1

MAVHAQACPAEPIILQAKSNAQQVYFHTIGYETGRYANNMRCSWILMGSSSRKRIMLTVHDSDIDDALFSECDDYCSIHDGDTSKSPEVVRWCGDKHPNAVISTTDSLYVYFHSDEAFQGKGINMSFIEFDIPGCPPSWIFSSVGYCYTLKRPVHGLTWLEAQKECNLVRSNLLTLTSAIEYSFVAAIYKKSKTLPWIGYMEDSSEGKFMPVDPNSGPWLEDLPKLTGSSPGQNCAYIDWRNRNGYILAMDDCRNRHEYICKRHQDGNTIPYLPQEEMMRSSVAKSPIRFTVWLFIILLILLMLILLYLCYRKCRKQHALSRIGSSDVNQRLVAGIDVQRAATSTTVSHIESSEVQRIDGANSDARVTTVTENAFNAVLDGGSSRQTIEQNTPNQNSHSAQQLNLQRIEPTVLTIEPCFNARQTSEERLSVPSRSKQNLPFGLRENTMGSTTREEALLKIRRGELFERPRVSVLDHTSAISLDEFWNKNEMSTINRN*

>Bma|Bm2925.1

MFYQLSSIWLHILLSTIWFHILELIQRCNGCLATSAVTKPKSHPSTYNPRTPTTTIAPNIQCFPVARQKRNSLSSTSSASNNLLVNNLPANCLPIDYLKTAMAEMKAGLVQQSDSFNFEHKLEESSLFPSTISKHPAIAIEHHTINDNSDNHQLLSSQHQTEKLCRDFEWDGPIYLYNQSLKIKVPYCYKYVASIDDDTDELTMLTQKSAREKCRSYSDINGGSSDLVSIHSNDENYDLQSTLAFFGWQSAWIGLAYDDVHTTWHWTDGTTLSFSKLSLRNPSQHCAILLTNGSWISEDCKSNTVSHFVCKKQAFA*

>Bma|Bm3563.1

MNNYSIALFTICFTVALCAICDEGWRFSPHSKKCYKFFPKKITWTEAEFHCLIQNGHQLSVHNSRENRFAKEIARDAPEVWLGSAKFGRTAQFEWSDKTPFNFKGWKNGQIPVSKLIRPCTKMNVTSGEWFQSCCRIVAPYICQKDSQPNKSYTSNIQKSYDTIRTNDKYSIVNQQWRFLVRK*

>Bma|Bm3770.1

MSRWWFVRLIVAIVILLAQIVGSHSTVKARQIFISPVQNYGTSWIADPEGHHYQFHLSEQSWNIAREICLALASDLVVIKSLQQINWLISHYPLRNSIMPERTIQIGLVLVDKENSAEKEWKWVDNTPLNATYLEWEILVRNTTEKALDPTERGRCALLSIDNRMLKAIPCDQRPDFDYTNRFICQRNDEQHREHERKNNPFYEFFAQWISELRKDTLVKSQPRTVQLQGVRTQIANVQGKVEDLSPISGKKENVVGEDKEDSFSGSQKIIAEKTLTTADPDVEGTDVDFATKQMEKEKQKVAKNETSTGNKHELQSKNFTAKRKSIVRKENIKNNEDVPLHNGKENGLISSLNPLKQKSQQSSELCDENETAQKDSRTWYEQFGDIFRNLNLFLKQEKSSDLRALLDNNDTNKTLVERLKETLHAKSSKEPSKHDVIENKRVLQKMNDQQQFDDSIHEVHDISNTLAHESDINSVVPYKKNTGIVLQKVDGRPVIDHTYGTAKLNRTEIYRKEELAKRSEVNEGEIYELLGKKLQSIFTERIQNVSNLDNNATIMSNEISKDIKSNISEEQINRNEIQIYYDVANEQKPGSPSLLPADQHNNITEETASVPRNFINNIQQIIKRPPESSNETETPLEFLNGNIILLEERKVDRTKTNIKLDIEKAISNMKQNLENASEEIRRLFSKSRSWL*

>Bma|Bm3839.1

MLYELIYLFFASTIQAATSPCSDEFSATKCLNEMDDVICQETFPINEAFPVVNAHCTNFKSHKVEKCRKQCRSCCQHPDFMCTDKKDSALNCRTIAEKGHCTTTDVTFKAILAVECAGSCGLCRHAGCMDNNYLLCSMLQKLCDLSDYYELMVKKCKRTCNSCAIDMQYPGWVYNKETKAHYKFIDAISQSENISAVKYERMCVEEDAHLVSIHSETENAFVYGLAGCERVFIGLRGQTSDQFGWLDGSRVDFKNFFPGFPLGTQYCAYMTGDSMFWYTDSCFTKGCAVCKKVN*

>Bma|Bm5701.1

MEKMNSLILMLLFAGSTAWTRLKFVNSGSERLQQNVMDRTACNHHNCLKNRKFLLTMMQSPLRIINIAVDPPVNILIRNSDECKYNEDCKDENFCWNGYCMPRGNPGDSCLSDDQCSSGLMCSNQTGSNDGICISDLTEKNIKNVSDKSGMNWIPKDQVIKTNAKMGKKLETIFTHKDEGDIKNRTSSELTSNFMDGTMIVGKNNMTEYLLPPITGINTFGGMMGEFSSLSITTDNTLNTTKAPSSPETEITTEGTLSTFPSFSAAEITTEDSLSMSLPSPETEITTEGTLSTFPSFSAAEITTEDSLSMSLPSPETEITTEGTLSTFPSFSAAEITTEDSLSMSLSSPETEIITEGTLSTFPSFSAAEITTEDSLSMSLSSPETEIITEGTLSTFSSIEITAQNSLSRVTEIITDNALATKSSSSPMSEDSIEDAVMGPSFFSPENSGEGKTTVKFAFTATIGKSNISEAEKDIPTVPSNQSDVTNMNTVDSHRGILFEVENNSKSYSEIADMISSYSSHPSNDSTTTGLNTTDISSYLLPQPQYWCDDGWKLFRGRCYIQMNDGNYTYHEAKKKCMNMGALLVAIVNEDVTGFLYSNFYFKHPFWIGLFNDGKGWKWSDGTKLKYQLWGKNEPNTIYSCVFAETEKNGRNWYTANCDDTILHDSVIGCVCQQ*

>Bma|Bm6131.1

MIIFQQYHLFVTPQKQRLRLSLSLILLLLLQQNVVTATIFTDNNTTTITDTAVSISSIISATIISTTTTTITTTTATITITTQQENGLKNVTDAKYTVSDIGLECAQGWEKCRSKCFRVYTIERSWPQALLFCSRYGSQLARIESFGENSFLHRLVNRQQKNLPINRNEFWIGVVAQQTEDENAFFLWSDGTVISRYVGFWNDGQPDYRTGTCAKVSITTTNELRWSLEMCNTLLPFICVLPACIKGSFFCQNGKCVPHSAHCDGINDCGDYSDEFNCPASPKVITCLKYEKGESGKIQSPNFPSPYNANANCRWVVEGPINSRIYITFDAFETEEYEDFVTILDGGPAENSSVVMAILSGSKKPETLISSTNVMVVRFSSDTQIQARGFEANWRATSISCGGILKAQPYGQIFTSPDYPKNYPSGVECVWKIDADPGQLISLDIEELDLERANDFLQIYDGGTPLAPILARLTGTFSNPQLIISTQSQLYIYFYSNFARNGRGFSITYKRGCSNRIRLDKGIITSPGYTRISYPNSQRCIYTVELPDRNSEQPTAFAINSFDVAEDDRLMMFEEVEGGRALHPGDGFSAISRPPKSIFAQTGIVQIVFTTNSIRNGLGWNITFSTNCPPLQTPKLVSLSTKASAFGTKVTASCPRGYEFRTGRGQMFDITCQLGGKWTEDHIPDCQPVYCSTVPQIANGFASSATNVSYGGSAKYTCYDGFDFSTGKDSGEIYCTDEGRWTLTPSCKAMTCPALAPFLNGERILEFGDGTGYGTVFRFECTAGFRRIGAATLLCLSTGEWSFAQPYCKKLTCTNVPLITNGVVVTGERFEFGDLARVECQPGFRTVGADSLKCLANQTLSDVPECQDIDECAEGSAICSIQSTKCINMPGGYHCQCLSGFQAQLSCNTASVLNSLSAEGSSEMDGFRAEDYATTGWCANPNDSNRKITFVFAVPKVIERIRIEKTTNGAYPIVISLKYSNRTGVPLIPFVAANITKLITRNVAIVGGELLVLPQAIEVRVLELTIEEFFNNACMKLDILGCHKTNCFDVNECEQNNGNCEQICINSQGSYRCACEIGFDLLTEDGQGGVHIKDGETGLNALDVIRYNQTCVPRLCANLSSPKNGLLLSTAKTFHYPMIIQFQCDFAYQMMGASHLKCMQDGSWNGTAPLCLPATCQGVRNNSAIGLFVAPENSTIAYGRNVSIVCSQQNRPASSSLLSSFRQCIYDPQEDGRDYWLSGPEIDCPLVDCGPPPSLAGAIYEGDDYSYKVGSAFTFSCRPPYSLIGKSSYDDRTIRCNVDGNWDLGDLRCEGPVCVDPGFPDDGQIQLESVEEGAQAKFTCNRAGYKPFPSDTINCTLGTACVLAEDVGISSGFIPDGAFADNSDSTTWGYEPHKARLSSTGWCGSKDAFIFLSVDLQRIYTLTTLRMAGVAGSGHLRGHITKMQLFYKVQYSQNYDTYPIEFETPSGNHNAMHQFELNPPLRARYILLGVTEYEQNPCIRFDMQGCLAPLSIAHEIPSHLQVGWNASVPQCVDSESPTFHNCPTNPIYILTDDNGQLLPATYEIPTAADNSGSVAYIRVTPDGFEPPKMITNDMDIIYVAFDDAGNAAECTVQLRIPDTQPPVMKCPDSYIVPANDGEFEKLIRFNESTVHMVIQDTSNITDVTFEPSEALLTLSSHVTVEVIATDSASNRNKCKFQVSLQPKPCSSWSLIGEENVEKECQIKGATTICSAKCARKFTFVNGKNGTRQFTCTNGIWSPSNVIPACVPIALEPARYELTVSIDYATLTPVGNDCLKGYSEYVGTFFNNLDATLSQRCSSSIEVFVRFLDVKFINTVNGVTANYTIQILPTVLQNVFYELCGLTLRTIFDLRIPGVTVPVQNLLYVNGETIATQSVGCPSMNATKTVVVQGFGCADGEVLREGNAETLPECLQCPKGTVHINNTCELCPAGSYQDEVAQITCKPCPEQTFTQFPGSQTFNACLPICGNGMYSETGLIPCQLCPRHTFAGPPIFGGYKQCEQCPQGSYTAKLGSTGPSQCKLPCPAGHFSLTGLEPCSPCPINWYQPVLGQQRCIECHNDTITRDVGTIEGTDCMPVDCSAVKCENKGTCMVDNHKALCFCRPGFTGKYCEEQMPLCNTQPCFNEGICETAAGTFRCICAQNYTGSRCQFGPDECIGMSCPNGGVCHDLPGLGTTKCICRTGFTGPDCSQIVDPCFMDNPCKHGADCVPLQLGRFKCKCLPGWTGPTCSININDCAENPCAMNATCTDLVNDFRCECPPGFTGKRCHEKINLCAQNPCINGLCVDMLHTQRCICEPGWTGEICDIKIDQCASHPCLNGATCKDQIDGFICQCAPGFHGFLCQHMTDHCASSPCRNHATCINQGAQYLCECSLGFEGAHCEHNRNECDLLHKCSQEGTELCEDLINGYKCNCRHGYTGELCEIHIDQCASEPCLNNGTCVDTGSQFRCDCPRGWKGNRCEEEDGLCALNPCHNDAHCVNLVADYFCVCPEGVSGKDCEIAPNRCLGEPCHNGGVCGDFGSHLECTCPKDFIGVGCQYELDACQEGVCQNDAICELLEGGNYRCICEPGFTGQNCETNINDCSPSPCPLAAICIDQVDGFFCQCPFNMTGLNCDKVIDEDYDFHFYDPILSAAAALSVPFKFTSSAFTISLWVKFDVPLTRGTVLTLYSSRESNYPSKISELLRISADNIHLNLLHDETPLNLHFPPTQRLNDGNWNNLVITWQSIGGSYSLIWNAVRIYADIGYGTGKILDINAWISLGEPINEFSSEPKFVGSITRVNIWKRAIDFEAEIPSIVHQCQQQQVIYDDLTLRFAGYTRLSGKVEKVVRSTCGRDYTQQPAKKIDIFGCPSDIFVVSYQKEVNITWQEPVFTSVHGYVEVKRNLKPGQVFTWGEYLVVYLAKDNYSIAECIFKIYVSREFCPTLQDPFHGVQACESWGPQLRYKACSVECENGYEFSIEPPVFYTCSSDGQWRPRPINAYTFRYPQCTKAHQAIRVAEVSINYPTVSICNAAGRNTLAEKLSQRIELLNSKWNIYSTSNISDHSVFNISVQCFAGNEETTVTAIDMTVRLRREAQNFFNVKISIPITNDILENSKTGQRAKVSDVLENEILLEDIFGLEQVIPNGRPDLNSFELKERHLCEIGTVSVRNLCVPCAPGSFYDLTTHTCKLCMTDEYQPRAAQTSCLPCPRGYITTAPGSALLTDCKNACDAGSMFNISSGICEPCGFGFYQPAPGAFSCIPCGVGKTTLKETSIAEDECRDECPDGEHLTQVGVCLPCPQGTYRTRGVHKSCVDCPPGTTTEGIASVRRMQCNTPKCSAGQFLVTTTKQCQFCPRGTFQDEEIQTVCKLCPPDHTTASQGATQASQCYSTNQCATGEDNCSWHAVCIDLPDDNDIPSYQCKCKPGYKGNGTHCQDACNNFCLNDGTCKKNPIGYVECICKENFSGDRCEVRFQARTQKVALITAGIGGVVTILVIIVIIIWMISYRFNRVEESSEPEKCPVEENTHTNFLYGRIPSEQPRPIGYYYEDDDEYDMKTMFVGEEEKEMAERVRHAQAHMYTPSNNRLD*

>Bma|Bm6657.2

MSPEIILVNYYFLLLIFNLVPLRYVSTKIITANVNSRYHNVEQLSNKHETECPRGWTRYERTKSCFHVIERRMRWSEAERACANFGGHLASITDEYENMFAFNLAKDANLSTPTLWLGRLVKLTRTGAYEWNDGAIGRHTDGFRGELPSGTDLCLTMWLDFDRPEGSWNEWDCNYVSGYSALCKRSLKRTPITTTATSNTRSGSHSGLSYYSRRCCLISSLCHNTSQSCTPEERCIPDDLDCWAKICPNGGIGWCLPLPKI*

>Bma|Bm8663.1

MLILFLLSTFDIIDKINGKKENIAEAKIQIERNKLSCHNGWVPYASTGSVKYTHCLKILTVFRMTWQQAEESCRQVGKDAHLVSIQTMEQISWLTEAIDREMQIKGKSLYTLFPKNSPWFDSGWWIGLGQLCPKSQSDQMPSFRWTDGKLFANDKLIIGDVKLTVPKSNKWDNHYCVFYDFQKSPTGLATHRTFNITTCSDRRRFICQMPAFSQDQLSSGGSAIVTEEEVEITESANSELTADFPCGVDPLFCPLKTSQGTVCYRLQTEPFYWEQAMKECDAKHQSDLTSIHSKEEANFIYEMVQLQPSISGETKYWIGLHRRNAQSQYEWSDGSSFDYLLERLRNDDPDEERGICVAIQFNVSTKLLQKQPLYNLFGVPIQVTKNTPAYFWTHQRCDNRHLAICRKPGFDYHSRFELSAKEERRLKKQSNWKCSNGYKLFRGMCYKLYGTNNNGVTFSEAIQRCKNEKANLVSLTDMYENGFVTSMLQNLNSNAWIGMDMTDGRVRWLDGEPLKLIRFGPDNRVIRIGGDRHIFQNVGEPGFSNEACVALDATNMVGYWNIIFNKTNYVFPSGSTKADNKSNQCDTMELPYICETYADESANVIQNKKTCNEIDDITYCFLLNDPNKHINGHFNFVDASEICATLPTKYTNYGRKYGQLAQADNIFEWLFLTAMALENDYDEFFMGIRFRKSVGFERTDNLRLRLAPWDIGEPNLKNGNCVVLKIGRNGPAWYIDDCMKRKPIVCRLTNEEPMSMVPQTVRCPDGKEDWILGETHCYHLVSNTSMFSSGFKADHDCFKYFNATLASFETQKDFELFKSHILHNELSVSNALIGLIRQQHGSYAWKDLSPVTFLNWAEDEPADTKGRIIQQCVKMQLNGNYTWHSLSCWQSTHFLCSTPVVNVNYNPSARENDKNEGFSSQQLSYDKERNSRKKLPNDYDSYSSKTDQSLLPVPSTQSISAISTFGEICLVLIVALSIVGGIQLWKRKRRLRLSSQRIVQFDQLQNEEENTM*

>Bma|Bm8932.1

MNGICVQSFEHLATTNDLETLIVPPFKLKPIKFDAKEKINNQPTNTTEFQTLKMAKSDEQSESEELEETETEELSETVAEATDVTASQIPNEEITEVMNVTEFETIIETGTVSDYHTSNMETDSWTLEKCDSSADCKKSQICQDGLCHNSHSYTKLCDSLWWQFRQKCYLPIHGRFTHSEGSDYCAKFGAHLVTIRDTQQANYVNYIYDIGGSGTYWIGLIKDINGTLKWQSDESMIYTNWSEGEPEPRIGCVIINTVIHNGKWSITDCSDLQYPDQGFVCEMDIRNN*

>Bmu|BmChr1_8922.9_8095.mRNA1

MVGKVVVLLGMILRVGCASKVKYDENFIKEVSSRSDNEIEYGFDNMVPAKPYDQRLELNVSKILDSLIASHDRRIRPNYGGAPTEVNVTAHIITISAISEVTMDYTIDLYLRQFWRDTRLAFTSVRDDDTTKLTVGIDMVKSIWTPDTFFPNEKKSFFHDTTSHNSFLRIDNRGNVMRSIRLTVTANCPMNLHTFPLDEQLCALEIESYGYSTADIIYHWHHHKAVIIDENVHLAHFTIEDHFHVERTISLSTGNYSRLTTYFKFKRNIGFYLIQVYLPSSLIVVISWVSFWLNREATQARVAIGVTTVLTQTTLMTSTNASLPKVSYVKSLDIFLGVCFFIVFASLLEYAAVGYLMKRQRSSACRRANSQLVYYYEFEDQSQVRLGSAREKRRGASFRRTHSPQVHNGGVVEQGLPLMGLSPTHSHPSRCYAPCCPLMQHVFLLDSCPYFLLFVVILSFTAFAQNGPQPCPENAIVLTGSDYPQFVYSPYDEWRQYPPNTDCRFVLIARSMQHRIHLSVIESKLEEPLFTECEDYVSIRDGGLPTSDEVLRWCGSYFPPSLTSSGDSLYIHFHSDNIVQSRGFNLSFVDYLIPGCPADWIRSDDSMYCYKMFSSAHGYTFLDAQRNCFYERSNLLTIENAREYQFIVGKYSDSHKFPWIGYNDANKEGEFDAIDPNVPIWPEDLSSSLRGDHKDKDCVFLDWNSIKEGPAHTVDDCRNRHAFICKKRQDGTTVPIQLSATLLRHGFEDPVFNYTLILLILLVLLVLLIILWIIYQKCKKRNVVAASEGSRLVQSAPHPDTAARAATAAATITLAPVPEEKRKKERAKTAATDAVKNVLAISASRTTEARDLALQKTDDDQTFQLDDSLFVKSKPDVNSVTPTEYQPISAESPRIRTSPNQRAPQAIDSEFARAQRRDSNPVLSDESEAELGLGETPVPFSPVKGDNASPAMHSPPDRSHLYESGPVTVTTKDETKDAKDSGVASKTLSSTLPPVGEPSGPSREATIQTGITGPSSHRSRGTQPSHGFDRPHVGTLENVSAISLDEFWQESK*

>Bmu|BmChr2_4399.2_6301.mRNA1

MGKIERSSTLIQRCHGGGGEEQGSMDGRRAFARLSNYLSRRSFSTAKGDGKRKTMARYQRPIDGQQERWNDRINSTRWNKMEEGWINEMLRETALVGFVLGCICLSNAITQESLEDEDSQQTKRGYRFHNPVPATPHSPWLSGPNEKKYSFHLGQQSWVAAREICLTNNADLATISSEEELEWILSHYKPQLRHSKDRQIQIGLYAEIEENEPMREWKWVTGEAVNSSVLSWASGEPFDHANGKERCGLLNINSKTLDDVDCELGGSPLRFYRFICERTFNQHLKHEELNNPMWKKLEDILVFFGISDRTKAKNSNSTDSNESLKATPLEDEDYDEKKDLKKVNTTEIKEESKVERATVRLDEKSLKDEKIVEDKDKKLQKAHSHAFAAKIVETAPTRDDSDLLVITNLEPLRDAGKFDVVTESNVRTTRPDPLAHLADTLEQAVESDAPGDDDAKMKNLERIINAVQKMVGDDDKDKVGWETVRARVHSEKRPKFGPTIESENVNSNLERPTSKAEKSDNPNSKSGNLNSKTENSHFPSENRNAKSIDASDSSEIPHIPHHDSHRHPHFSFPDIFDEILSPMLPRPFRPRPFDKPEHRDTRKWQNPDKSYLQNREMNEINRQYGQKTVESTTLISKIGEIEGSGQDIAIPIEVFTKAKAEVSTAKPNLSNIEPHWVVRKTFYAQPHQKRKIKVAEPHRNPDRKTLPESFEFDQDEPEPQEQTTKTPVEKIQYVESKQKTEIEQNAVEATERLNPTEQFQIALSLDEDFKQAQKEQQAHLGVNSGDLLIIPKSSEEVVGCGAKNLKKESTISTTPSPAQIEERNLKINAFLNNLRDLLNGSDSTVLSNLFEANYTDLAENLADKLERASQLARTTQSVPPSPEELERLRKNIEKDVLKSIEELGETEDREFKSMEVTEKDLEPIGSGLEPPTEKLISLTPVALTAHQQIVVEKKTESPEEANVSGYTPKTTMELAKERKTKKFFVLSSAEAAENAAETEGFLAKSEEERPVKIETSTKLTLTSIIPESEEDSAEVIEGSGETPLVAKINEQKEQAKKQKKPRQSLFPDLDFSLLNNKKLAKEAAESSQKQKAQADEAYEALAKLDQNSLAARIAASEAQRKKTEQKADEVKDTIKVKKIQVQPALMKNKKPEDVAKQLEKMFYDWSNGAFGVFNG*

>Bmu|BmChr2_4653.1_7908.mRNA1

MGHPNMALFWAIVFYSILVQAEKSQNVTEIPYILTGVSMNCPAGWDLVGDKCFRAFNSEKSWPQALIFCERYGALLAKVESAAENEFFGRIISRPTKSSALSKHWIGLITEKSTEDEGSFVWSDGSVVSRFVGFWAKQQPDFGNGNCVQAGVNLEKSVEWQMEMCNLMSPFVCQLNACVSGLFHCGNGKCVPEKAHCNGHNECGDFSDELNCPVPHGQVACLHYEKSENGKIESPNFPSSYGPNLDCRWVIEGPINSRVMITFDQFETELNQDLVTVLDGGPSENSTVVLDHLSGSPQSSRLSLITSTNMLTVQFRSDALVQARGFKASWRAVPFACGGSLKAHLFSQTFSSPRPYPAGGECVWKIEAPEDQVISLNIEEFDIKPGKDQLIIFDGPHPSSPILSRLAGNLSNQLVVSTQNFLYIYFYSSQADSTNGFTIHYKKGCNNTIKYSHGDILSPGNGIIPYPASQKCRYVIDLGEGMSDRPISIDFNTFDVQNDDLLKMFDGDVNTTALHEGKGFTEGRNPPKKLHSTKGKVSIVFETNAMKQANGWNLTFSANCPPLKAPKTVFLTTQSTSFGTKVTVSCQTGYEFTNGIGHRFDISCDLGGKWSNDYIPNCQPVYCSAVPQIANGYAFTASNVSYGGIAKFKCYDGFHFNSGKDTEEIYCKTEGWTETPKCIAEICPALPTFLNGERTLKFGDATGYGTVYEFSCAPGYRHNGPNTILCKSDGKWSAQQPVCKKRICPNIPQIENGKIVLPPTGGLEFGDSARVECNPGFRSTGADSVKCLANQTLSEVPECRDVDECSEAIASCVTKSTKCLNLEGGYTCQCLDGFKPQLTCPSFHVLSASLVKTSSSAIFNMSSVTFCAERFDDQRYLEFEFPAPKVLERIRFEKTEDGVPLAISVSYSPDLKSRLRSIQDLLEIPLKNVDMAGSQVVVLKMPVEAQRVRVRLDRFKDSACARIELMGCQKTNCVDIDECEVANGYCDHQCHNTVGGFKCLCRDGYHLFAENGQGDVMLKEGETGFDNLDMIRYNKTCVPRKCPKVEAPANGKVINLNKEFAHPNVLEFRCDHGYQIRGSPFLQCTSDGVWNGTVPTCTPAVCEGVQNNTSIGMFVTEDKPYVPYGDNITVVCTQQNRPIKQSPLGGLRQCIYDPRPDGMDYWLSGPEATCPLVECGPPPALAGAFYEGEDANFKVGSSLAFNCRPPYSLLGKSSYDDRIARCNVDGSWDLGDMRCEGPVCVDPGHPEAGQTHLNSVEEGAVAKFTCDRPGYKPYAYDAINCTLGTSCLLSEDVGISNGYIPDGAFSDSDTKTMWGYEPHKARMSSTGWCGSKDAFIFLSVDLQRIYTLNTLRIAGVAGSGYLRGHVTKLQLFYKVQFSQNYDTYPVEFEAAPGNHNKMYQFHLEPPIKARYVLLGVTEYEENPCLKFDMHGCLAPLSTAHEIPSHLQVGWNASVPQCIDAEPPQFINCPANPIFAQTDEFGQILPVKYNVPDVQDNSGVVAYVKVEPKDLQPPFHIHAPLDVKYTAYDEAGNFAECTVQLRVPDTQPPAMKCPESYSVYTSGNETSKHVVFNDSSVHVVMHDLSNITEFSISPSETEINLFSAVDVEAVATDSHNNRASCKFQVALLPEPCTEASLRTDDHAIKKCSAVNNQTICIIKCKTGYRFVNSSIDEVKYSCTPSLEWSPKSHPPACVHKSEEPARYEFQVSVDYSSSIPARDECMKPYADAVANAFETLDASLTQKCSSSVQVYVRFLGARFMVDPSNNKMVKANFTVQILPTVLQEVFYELCGLTLSTIFDLKIPGATLPIKTLLQLPNGGLNAPTCMPMNAVSTKIHQGFACSKGEILNKDGIDLPECIPCAKGSVFAGSSCVLCPPGSYQDKDGQQECKKCPEGTFTRTPGTISRSECLTVCGNGMFSESGLIPCQLCPRHTFAGPPLPGGYKTCDACPEGTYTANLGATGPSHCKQPCKPGFFSESGLEPCSPCPINFYQTSLGQQRCIECDNNTITHEVGRATEEECKPLECTGVKCQNKGVCAIANHKTICECRPGFTGEFCEEQVPICDTNPCLNGATCENVAGTFRCICPQNFTGSRCQFGPDECIGVNCPNGGVCQDLPGVGTYKCVCRTGFIGHDCSEIADFCQSDNPCKNGADCVPLQLGRYKCKCLPGWEGLTCERNIGGLFLLLLRDIAHNIRKFEGGMSYQP*

>Bmu|BmChr3_5799.8_4634.mRNA2

MFSQPPGPPQFPQQPGFPPIQPQYPVPTSQSQPPNGYFPQNAGPPQNFGPPSMYGPPGFPSGASGGPGIAPGSPGMPPTGVMPPGMGPPSGMPGIPGPTGMPLGLPGMGPPSGMPGPAGMPPGPPGMGPPSGMPGIHGPTGVPPGMGSPSGMPGIPGPVGMPPGPPGMQRPQQRLDPNAMPSIVQVVQDDRALYSDTAFQTGFTEAALPPLVTTEFTAYDTGNANPKFIRSSVYTMPVSNDMIKSYQLPITLSLTPFAELNPNELQPPIVDLGELGPIRCQRCKAYISSFMEFIDGGRRFRCPFCHATTPVEDAYFAHLDHTGKRTDVQQRPELMYGSYEFVATKAYCSNSLLPLEPAFVFLLDVSYNSVRSGLLETFCSNIVDILKHLPKDTNAEKPTIKIALATYDQQIHFYDLSSSNQTNMCVVSDIEDVFVPFVEGFFVDYETAAENLERCLRDIRQTFSDTRVTETILGPAIKVGLESLKAARRSGKLLVFHTNLPTVEAPGKLKNREDRKLLGTDKEKQVLSPGTEYYGRLGEECVKNGVGIDLFFFPNAHIDLASIAPVATITGGSIYKYQYFDAHRDGKRFLSDLKRDVSRLIAFDVMMRVRTSTGIRPTGFFGSFFMQNVTAIEMGTLDADKSLQVEIKYDDKLNENERAYVQVATLFTSPNGQRRLRVQNLTLKVVSDHAELFRNVDYTAVVTHMFKLCEKIVREQSPKDMRDELINASAKILAGYREKCSDGAPIGQLILPEALKLLPLFACCISKSDALVAGGEITVDDRTWMMNLVRSMRPDDVIRYLYPSVYPLTQVNVESIRESLPVSVRASFQYFQQEEAYLVDNGMIAFIWIGPQVNKDWLQNVFGVHAVENLDSEKSEVPLRENPDSQALHALLERLNRQRPRVLKVVLVKAGDGLESWMKKFLVEDRYAITWKLRCSQIPYRSAYISGYCFVYKDTGHYNCSTVKETASGLYAPKLVSLSSVEERIVNHLKKINIFSSVNEQDLCVLNMGFRCPSGYDLFEDFCYKAFKEKKTFAKARSVCQSDGGDLPEVHSDTVNARLGEYVYYNTKSPIWLGIRIIGSYWNYDRKRVNNDHNHEQPYYFRWMFQDYGNTHAVLDHGIYAGYWRSLKPTESHSFVCQVRAY*

>Bmu|BmChr3_5879.8_10386.mRNA1

MTSNLRTKCIYYNADSVFAKNKCFIIDQLYSRNPNASQCNHLHGFNGKMAKNDAEVLKYLKLRNYIKSGETLTTSETKGESYTTEARVLCEYSDIFSCENGYRLHGIHCYKVIRKERYWAEALRTCQQQDSTLAVPHNDRDTQFIASVVSKENILNDNDNSFFWVGMFVKDHKHLKFVDQTTPDYFRWLPIGHKRFMTKAYNTPNTVIAVGSSASNKGNVGQFGYYQSVMGHWYSPVQALGISICFAVLDVVICILGLAWDGKFFTFDNIVHWFDFQSYNFTRNPIDFLAVCVIRICLLLGGAIGVYANPTSGAQACSKYSNLTFAAVLLMIAFSPSKLLAFYEHPNPLAVGDWILMIWSMTACLFVQGIWVSVLGKVRDDAYRRLDDRNFYESDNEYYAELQRREAKEQEEKRETFTMLLRLFSYMAKEWMFYMVAFTFLLLYSFSRVFAPYYTGEVVGAVFGEGASYAQLHKKVLIMAGLCISSAIFGGLRGGFFTYSQARVDRRIRNDLFRSLVRQEIGFYDANKTGEIVSRLNADCQTMSNTLSLYVNVLTRNTTMLFGSLIFMFTLSWRLCMVTLIAVPIIFLVSKVYGVYYDRLAEESQASIAKANDVAEEVLSAVRTVKSFACENFESLRFLGFLNVTLGIGARRAIAHIGFLWTTEFLQMGILTVVLFYGGHLVIQGEISSGLLVSFLLYQFQLGENLRELGEVWNGLMQAVGASRKVFELIDREPKVINDGRIAPDGRNAQIEGRIEFRHVNFSYPTRPDLPIMNDLSFTVNPGEVVALVGPSGGGKSSCIAMLEHFYEPDSGEVLLDGVPIRDYDHKFLHNKIALVGQEPVLYARSVHENITYGYEPAYNNTEMVQKSAKMANAHNFIMETTEGYETDVGEKGSQMSGGQKQRIAIARALVRDPVVLLLDEATSALDSESEHSVQEAIQKNLNGRTVIMIAHRLSTVENADKIVVIAGGKVVQQGVHKDLIQQDGLYKQLVQRQMLNGNIQEAPPKPVPAPARSRQIRQSRESTSQAQSFLGTSLAKTEMSVEIDNVDGVDAHYAYFRSVYGQLSDSTVGMRLRNIYEYTKNSNQIVQKSLDGVETRLYDVSDNFLAPLYERYCYPTTERLAKASVTGYDKSQNLIHSAKDLAQAGTALGLGVAVVSAQVGLIISASMTNLLLDGVILTKDVGVGIVAKTKAYEKQIEAKIFSTVENCKSIAGVPVNKAAEYTNSLLDVMNAFLDNFLHLPVEEIENPESSIPQRIRHLTSRVSQAIVSRTVQPAQQQIAVLLENLRAKLTLMDTLKKQGGNVKSQMLSTSLSDLYGRIEKEANEFKVHPSEALLKSIQNCAKRLNASLNDIAQKSSKTLPSAASDRLQSAISYIENLDRTFEDAESIHAIRDEVIVEAKQKLSDLLQIVRLSPRNPDDKIDLNEELSD*

>Bmu|BmChr4_284.4_1821.mRNA1

MMIPQLSNILLLLCVCVYADYENKLIIDLNNPPPSRRNSRGPVRDDAGVLNEVKEIVYRMEHVFNERIKNLSDRVSRIENRQILHDGNPHIDWKPLGTSGKKIKVFSSEATWSDARRTCSSNGGKLLEIQSDNENRKITDYLQNFETNLFWIDAQVSVSVRIPTGRYQNFQNNRTEKDCTALNVLGKWIRMDCAERHGFVCQF*

>Bmu|BmChr4_1934_14178.mRNA1

MWPPGAGTSILALICLFAAYSTQEIDPAVAQFAEHQQLLLKQFKADKNSRPDAFHGTPPNNTVTRTKREITDEHPKAQNIALQAIRDISAKAVSLEYCDKKAICWNQTQLDHHSGEFPGELLELDFFDQCIGGLVVHVDRHVQDLVVVITTERSGYPSGKLFNAHGRRLFPNFEVIHPHIRELHFENVQDEGTGRFFFVAESNDNEDCVVAVRATTDLVLDGGFVESPHDDRVQEFVGEYHELQRDPTELVPGYLAFKPSRLEFPETVTTVTFYRSQHEQQYAPLLVGTRYNCNANVFAGPYVCLEYGIYFVKIRGTDRGGNVWQRVYQFACNAKPGPGPGPGPTPEPTPQPDHCLNGGTFVNNETHRYCYCGHHFEGPICENKLCDHGGDIGSDGKCVCYGSWDGEFCEHLTCKERSGRTFDTTDETALNFVIRSHEDGRIRSQVLESISLIINTFEAFSENVVKAYTATFILDGRTKLYFESDDPTVFLEEISEIEDHWLPSDKCTDKIFLGIDEALEIGAASYYRNSPIIVFTDALPDDPASTRFDILQRLNKQNNPIYIISYGGLGFKCNPDIHTEAYEGFRILAQFSGGLAVKATETEDEHLSWAIGRVAYTVAVGLYQEHLLAANDLLDSCQYAPNEQLFFVDDSAEELTVAFVGREDYEVNVVNTIEEEVPAFYVMKGGDLTSKVYKNLEFGNYRLVIKKGRNSTETELPCHYRVYAKSKYEVFTGASIDIDHDVTLDQPRFNEEMHIVARVNNIEFPDPENVFAEALIWHNSVIEHDKREVLYASNGVYRDGCDYNLYFGTWKCERANQFFYIEVYVEDATGLTVKRTTVGHCTVRDPNPPDQGCRNGGVRSGDRCLCVPGYEGEKCENIVCYNGGRSHVTHCECYAPWSGPHCSEPKCGYQNRWASFKPQGVGLSFVIQGSSDVQSTLVELNRLAPEIVRDVKFNDPYWISHYSIHSFDDKEVKLYLRTDHPDDLIHGFEALQQDTQNTESVCKDLLIYEAIIEALTSSRATEHEVVYLVVSGGIKLNEKREKETFELIDALGAKINIIQYPINKCRIEMDQEASTAIIQLAHYSGGSLYQTNRGTAFITLPLQYDEATVFDEVRDNCEKEQTFYFPISGNAQTVSITSLGDLRKEYPKIAGAQKQFVDDRKIYDSAMSRLNIVYKDCPDGWVPYEQNCYLLEVREATWDDAKATCMVQGGHLLHIPNKGLDDLMSLYTGSRATWMGLNDQNSHQWGWDQGTHDDLPLGNAYKNWATGEPKSGKHCATLQVSGWATANCNETYPIICQKHAYDLGYNPGGREHGHLSRGIWSVKLRTYNDGFTTCGIRVNVQSATQVFRKFTKKLDDDFGNGALVWKSKNNRIIAHLESHVNGHSSEARLEYAHLYPSETANLGDVVAFHKRDQCSYDYISNTFTASNLGYYVGFTGFDAYGYPFQRILPAVSEDSIPTCKNGGVLSKRTEECVCPPAFRGRECQIPNCKYGYPAPNGVSCRCFPDYEGPLCQYAVCLRNDTDHPPPPMDVYGKSFIVVLDGSNTANNAKVAQDFTKIINEILDRLGNTEHQMYHNYIGILAYDDAQDQPSSPLVVETARPKFLSELQSLINKNYQSKGQKRDYLYALAKLIRHKDVIPGSAVYVIGDSGVEDVKTWDAELYNRIAEKHVTIHTIILNDQVPPGNASNYRDPSIQHLIGLPFVTDGFIYQVNPDHFKDLFYIQLGSRFRGYSLTHQMYRECSDKIEYFQTGGDYGLLVVDLFTAHMDLDFDVIDPNGNSTDKAEHLFITGTNVLFTIETNIPGIWTIQIHHKEKPSACFVSVREVANSAPAIGFNTDIQGDNGIHSRSAQYYPQDGLNAIIANSDSDPLTYAQVYTHDAHTLVFASPLVRRHDCAWNYISSEPFKCPASTFTVAVEGFDQGGHPYRRTYKTHCVGYKGPRAPISTAFSDEETSIDLPIGLAQF*

>Bmu|BmChr5_6207.1_774.mRNA1

MLSTPKQMKLEGDLKVSTLDDEKNSFFCGISLNRKNFEDDIEISYYTTTPSWEENMRTLDEKWQIRLGQQVAYLRERIEKLETTMNSLNHIMESDWNHFGDQKFKFFDRNVDWNEAQVICRLHNAQLINVDDSEKNKIISEFLTRSEQAITEVWIGLKTQTQMETDSYKYDNFSEKGLIEGCTVVNIRGKWKIRPCSRPRPFICERK*

>Bmu|BmChr6_6706.5_1338.mRNA1

MAFSLLTRLAVLIGCTVAQQAPCPTGWTYKSHTNQCYLLSSINLPYNEAVEYCKKDGGSLVSLKNQDEYDYVKELYNNNTRGFYPPWIGLKRDSRWYSPVWRWPDGFVAYFTKWLKEEPSKTEDNACVAWRTIENHGWKTLGCKYAQRFICKQASANCPTRNYERPEGVLSSPNFPTNYPNNLNCFYHIKVKPGYRIRLVFHNFYTENYFDKVVLYDDYNGNLTNQIDTVYGTYLFKKIYESSENVLTLNFITDHIITRQGWNATYTSVHKEPTQELNDPTGTITSPNYPKNYPNDIDQLFLISTLPDKVLNITITDFNLERNYDYLAIQDGKDIIKSRQLTRLTGAEVTTPLSFLTSQNYALIRFFTDPSLNYRGFSLNYEAVEKY*

>Bmu|BmChr6_7424.4_718.mRNA1

MNSSIVLCVLVLFLGYCSATTSPCPEGWRFSPFTSKCYKLFNDQTSWTTAEYKCAFQGAHHISVHDAADNQFVSELARQANIVWLGAVQYGTDRDYTWADHTAYGAFENWENGRRPSYHKGKKCSKFDGLSGKWIQSCCKVPAAFICSKPAAVLPSAIIEDDTGINEDFRRTRLAFRRRV*

>Cbn|CBN00312.1

MWTVLIIAIVTMAYGLTETTLYFLSTGACGSQEPRKPPMKYGDCDSDWVTVNRTKTGQKYCHKFFSDRLTYEEAEKKCQEYEAHLSGFTDQNELDILDKMLDKAKSEGVRFDNGDNVWIGARRRTACSTREGIGAEEGGFNPDPTHPCSRLQAFEWVNGVAQNPPDFEDRWIAEVEPLFYDNAENCVELLKGVKNWELWPKDVVGDKKLNDVHCGRDLYYLCGKEAPIVKTDK*

>Cbn|CBN00344.1

MRTRPWPYWFSILYVFCSSEVVAQDSNTCSGTVVLNATKQIQYLTTPNYGTSYKYPPFLDCKFLIKAPDKARISIEIIDMEMEPRIFDDCSDYVGFSEENVEKNISTLTTLCEDSHKRQFLSASNAITVIFHTDQLVENRGARLSYRYYDITSCPPDWTELSNHTCVRIETNQKLDWIGAQKRCLEQQSNLITFSNIEKANELQEKYESINMKLWIGNNDALVEGKLVDFTNKEAPRLSKVGHAVLIDNNEDNDCMVLQFQEADEFRMDQCQNYNGYICEMEKNGSSVLYPPPVKEIQEGTNMRAGQYTLWLLLFLIGILFLTVLAFLCFMCWKHKEGARIHTESTTIQQNAFMSDSSQRVEHSRSSAGASNNIPVSNDVNRTSRSSPVTVPIENNTRAPPKKFPMAPVPNRLPMNEEISGSEPAVETAILGGDEDIETVSAAPKPQPRTLPPMPAREGTFHSINTREGSTMRTRRNKELFERPVMNVLDNVSAISLDEFWSNKKS*

>Cbn|CBN00378.1

MKNQILFFLFSLFLLGCLADSDLTCPAGFKLVNQSKCLKIFPTSLKHLEAETTCRGFGGTLVTVKNAIDNRNIVNMAANSGASYTWLGIFCFGNDTSSCYHDDGTGALTYNSFQSGYPKSGLGGCVYMPTIGKTAGQWVNGQCEVSSHAFICEVPTTFIDPLCTHNFDGYCYLPSHEFSSDTLQNTTFEEAMVICEEQGGAKLVSIHSQKENDFIKSIYKYINFPQIFIGAKVYIGFSWVDDTRWDYDYTNPVASSDGKCLKMDTSGKIENGGMWSPVDCKEANFFLCKRPMFGVSEKEINPKFKRQLTVPASTDFPDFSHCNTTLYLAPGIITSFGYPEKSVPTYCTWNLAVLGPYRLGLFFNDFSTASELNITDEYGKVLSNPMGNLSPFQVLAPTNIVSMSYNSKMDAPDGYHGFSVTILPF*

>Cbn|CBN00416.1

MKQEMETTLPKDTCPFSLQSIDLTYSPDPSFYSYTWTPTETGWSFPGCRDGWHLSIRSSDLSVCIKVFLLDSNQNEAKDFCKKQDSVLIRVQTVEESHWMKKTVQSLVSSNQFFWIDGIRNCTGREEYCNNFDYSDGLTTGNAALTSDGNALLSFTSNGYIENCLIIMSNWESNATINDVQCVTEYSGMFCGYQLA*

>Cbn|CBN00560.1

MIFFSIVCYFLAAFGVVNADCAPGDIKNTQENCVHVENLASTWQEAENFCVAHNGHLASVHNAFDMTALRKVGSMCVNFWLGGQCQKGSNCNWVDGTTFDYTNFRNGNPGTDNCVVADTKSGTWTTQPCTATSCIACEIKGKMQDCQDWMKAGFTDSGKYTILVNGKETEVWCDMQTYGGGWVLFQNRLDETESYWDRNWNEYKNGFGDTDENVNFWLGNEALYQLTNGKHVTLRVELYGDRTPNSKNATDFWFEHYFEFKVGSESENYPLLNVEMDWAHPVGNASTAWYDLTCSIGSPFSTVDNIHDPVKECVTKFQMGGWWLKNCALSTLNGAYIPKDWNNGYGMFWIWDGSETILHPRKTRMLLRNTV*

>Cbn|CBN00683.1

MSNNQNSNQSLFQRHWFSANPDQIDQADNSKPSDLLSKHWFSATSDQLRLANPVESSQNGEEFAEVRYRDPSVSRRARIVRVQNDNVPPPPLYENTPIEVLETPEPPSGVPTPRALSAVESSAPRRPPQRPQPETSFLEVSINFFLDHWKIILSLGGSLILFLVAFFLVLFFVIIPPTAKTTVVSNTTTTAPNTTTVTVTTRLPATLPTTVITTRPTTVVTTTRITTTEQTFTMKPTTLAAGTTCTNNFVLVNTKCLKLITGLSNRVLADNQCRNLAGGAALVSIKSSDENEMLYGFLRYQTEKRIWIGLQCDRNKCAWDYGRGNIALYNNFAEGSPSVDVGKCVYYLVSDQKWVSGDCEQDFMGYVCELPPTVPDDCTYNYNNNCYAKIGNVISVGEAEQICSNACSHVVSIHSELENQFLKSIVNETVLLGGVAPANNLILWEDESHGGFLNFHETGYDPNHVCIFMKHTTGHWKTESDLLIDFENVPEISEKSVPTIVAVSSDSEPKTEFEPVERQRHFGILQYVIRTRLQRVMLIGLVNIILIAALFVFIVTLVTGQHNSPDTDVTTLETTATTITTKTKTSCAYEFQGNCYLKIGYSTSVPEAEQICISACAHVISILSEQENQFLMSIVQETTLLGGAAPSKHWIFWEDRSFGNFSNFQETGYNPNHVCILMDSQSGNWRTDSVCTGLTWCKRGVEEICNRF*

>Cbn|CBN00859.1

MSNTSILLFFLFLVTVSANVCDDLREKEGETNGCGKGWKRFQRPSGGWCIKVFFEELVTQAEAEKRCQAEKATLTGFQNQTEVFHVTTTATTHLKPATGSLWVGLKRNQKCLKLKITKNCTTMNSFDWTDKLTTGTHGFIWNGGEPLNYGWNQDCVVLTVGNTGYVETLFQVGTFDDVGCEMTYKNSARAIKGFVCGKKSEK*

>Cbn|CBN01197.1

MTHLKLLVGFSILLTVSAADFGDSSCDSSEEHGGGGHGGRPPRPPGPRPPRPPGPPGPAPPSGPDNSNRPRCEQGWYTSYRPQGIWCFRVGIGKMDYNGAISECATYGGVLSGIQNEWERWRMAEEALRQTAAYNVQYAGIWLGAQRVNGAFQWTDGHTTGTGGMVFGPGQPDNNNMGGRGPENCLQMIALAPGYWNNPGTWAAWTNGMIDDQWCNQVDDPPTRMYVCGKR*GPQG

>Cbn|CBN01228.1

MDYFRQIILFTLLAVGLAADNTFDPLILARSNEEPHQRPTFYNWDGRDLGTTAFENLPHQTSQSTPLPINQSEHCPDGWLKFFDSCYFFETEALGFAKAEKNCQDKQATLFVANSLDEWETVRAHTKKSQFSWIGLVRFSQYERYEKLPRWQTTGSINPSKINWLINPYQPLGNGWSTVANCVALFEFPSTIESSSYAFFYPCTQHFNSICERNSTILNARN*

>Cbn|CBN01396.1

MQRRTTIWFLLLFQPLLVFTQNRTEPYDCKIGSLNPLAATSLNACFKLYDSPKSYQAARRHCVSLGGQLADKINKDDSSLYSANADLEVTNGTKFWVGASNLKCNIAWENGGEIEYNEMWAPDSRYYGVAIEKMSLGGLWHTIPVGQKLPFVCTFQGKSKDAPAPVHAMRAPAKKRTAKVEKAKEDDIDESLNAALADEKKKQEANSNNKQDSKKDEEDINESLNAALSDEKKKSEAMALSASDKKESSSKDEDSNTEEASMSAQQALNASAAMSASMAASSSSSNSSSDESLDETYDSAEMAMRKEIGKTVVAMKSQEMASQSEDYDKYTDEDLVSAAASLIGGYTVNANWADSRTRNSSSFDSDTDEESMSMSMTVADQMAMGMQSSNKRESSSESSDSMSESSSDQMSEQASMAMNAAMQASEKSSKKSESSSKDESEDSASMSMEQKAEANAKMAAASASASKSESNQNSQDETVEEADVASAASIFGKEKQKSGDPKPTTKVPTTTEEPDIDESLNAALANQRSTTTNSIDLSTVLTTVNPINPLIVAAKPSEKLPGCPAEWTQFSTNATAPSLCFKRFEKPMNFEDARLFCVSKGGHLASIHNERQLLLLSALLHNNGPDALSDQTWIGLNRIHQKYYVYEDETPMDFTRWLPGAPNINDCTVFTGNELPNYPHKGTQYKFGDFPCEETQKSVLCEVTLGKDKTKTQPTCEDGWSYYSHDGTAKSGKCYKRIDQSKTFTEAREVCKQENSYVASVQNEGEARFVSALVQTEKNYTVDEQTWIGYVKYDRDFGWEDGNQGLHFDPWTEKMPREKKCTVFTGNEIHEDCRSQYRFVSVECNKKQRSILCSKPPMVNGVPTVFKVTDNSSKKLSTQGTTWPEAQNACSKIRNNSASYLHSEFSREKRDFVRRQASDIEYYIGLHKVGGVWRWDQPNGSFCPGLSGFESWDENYPTTYSDSIAVVNIVKNGESKWQNTRRYEEFYYFCETIAFDTDTKL*

>Cbn|CBN01470.1

MTSTTIPTSKTVSTAVYQSTHGSTRLVSTKLSTSTTSTPALNFTTPSPTTTISTVICTEGFTLINNKCWMLVNTKDYRGNANKVCFSNKGSTLVTIKSYDENEALAIFVKVKNISKIWLGLKCEGQGGSTCKWDYGQGPLGAYSNFGSGSPNVTVGDCIYYAVSTKQWISHSCSDKNVNIVCELPTNKDDEKNRQTDGQILYL*

>Cbn|CBN01630.1

MRFSIIVASALTALLLVHAAPTPSNNDFESSGEAPVTLPQNSEEEPHRRLRFYNWDFKDLGTTAFEDIPYPARQPPSAVNQTEKCADGWLRFADSCYYIESALMGFAKAERNCFQKQSTLFVANSIEEWDAISSHAKEAYFSWIGLVRFTHYEKTEQLPRWQTEGAINPAKINWLIKPFKPVSNGWTQFANCAASYKSPAKLESASYTFFYPCTYLLYSICERNSTIVNVMQ*

>Cbn|CBN02556.1

MPFLNLSSRIRRNNHSQQNEPLLLSRRFEEFLQSQNPKRQFIVLILFLAVVAYLLTKGVFELYGFLENREKNGVLIRDPLLEYGLCGADWVTVNRTKTGHKYCHRFFSEKLAHDDAEKKCQEHGAHLSGFSDQSELDILDKMLDDAKRNGTRFDNLDAVWIGARRRKRCWTRKGIGERKGFNPNDKHPCSRRLVFEWENRAAQNPPDFEGRWITEMEPSFSGEAEMCVELL*KGVKYRDN

>Cbn|CBN02609.1

MRLLLLLLVFGMTATLGHFIEGKSSISFGNPSFHSSSSSESSSNEGRSSHRHRHSGRHGRRPPRPIQNDHSHSHEYDGGEDGGENRGCGRGWKRFDRPTGGWCIKVAQGQFSQAQAESKCQVEGGTLSGLQDTNEISYITSTAIRLFPERTGSLWVGARRSPACSRAPLSSSCNRMTSFTWTDGSTTGTAGFQWAANQPDNSHFKTQHCAVLLASSPSSDPTWNWLTNKLDDEACVNPTGSYQRIVRGYVCGKRANERRRK*

>Cbn|CBN02629.1

MRTWVLVAALAVICLAAERELSHGERAKAVLKSWNPRNEIKYFEPIREQKYRTTDEADLNLETHHISKRSVGQPHFLAGMATRGCNLPGYTGDTCQYPLCSARNPYIPNNQNTDDVSIDATNLANCSTPYVIVVDETMWDIKIQLETDSPLNPTFYLQAENGDLVYPDSDSQRPTSYVAQYQFLAPGQYQLGPRADTGSEFCTMLMSSRTQIQVTGGFASGAQAERNDYPTLKYVYFDTESVVAVHAQGLDFPGQIQAIGFTGAENHISRYIPIGTRYNCTYPYILERYTCNKNTNNDRGENFLQVEGMSNSGYRFRRILSYTCILPPVPTSTVPVPITTPAPLTTCKNGGQLLKDTDGSSYCYCFGLFAGNDCSQMICANGGFLPEPTSERCQCPEGFTGFHCQNIVCSDTSGFDFNAENPTLTLVLRSRTELSAVIEQATTSVQGMVDLLSSEPGYLTNFIVVLFDNGKLLINKRYDSWDLAMVDLTKAINSAPSNGGCSDVVFSSVAAALSLYPTNKSPIYVITDASPNDSTEKDAIFQLESYWRAPIYFIYVTPLPNENCNSSPDNTGYRDMVDVAARSGGNTFYFSNRTIVSNFFYQHMLNTLFRSQLILSGDYSQCSSQNVYKSAAIDLTADMVIVVATGSDLSLIVTNPAGGTPDFDVVYTDGTNYIWSYNKQVSGQWFFNLKSSQPNSACTFKVYQKKYNFGGMSQYNPDYDVFWSFATTLSSAAGVLRQPVAGFDAAPVFHISNYPAFISMDRVHANLQIYAIRDGVQTEVYGSSGMWRDACEFNFYFPPFTCRVPDEVLYFNFFARDNNDMTLQRAGTMYCANVHPNPPPDHQCQNGGVMNPSNTTCFCTPEFTGTYCQNVVCYNGGTPNGASCLCPPGYAGESCEMAKCLETGPDPEYIRYGVDMVFAVEITSQSLASLVMLDTNFQEILRDVLMQDRMWIRNFVLVGFNSTWGGTIASSPANNLTAITAALHNLATSVPADTGCRVKLWEALNDAIFGRQIVPGSFIEIFQTTPEDEMDQRSLGLFYDMSRAMNINIYGFLSARPQALPNGFACNANLTDYYILFGIVTGSTGTTYILQAPEISNAVRLIPLQFSNGQVNINAMTDCRHDSGMITYFPVDAYTQTIQLTVFGYGTQIQVYNGNGVLADALELFYDDFTGQSVYEVRKTCDDGWESFGQYCVKFLATTDELMAFPQARNFCATAGGYLVDDLGDDKNNFLSSIAAKTQFWIGLAKGNDGQFYWDRGQGITPDLLNQPNTYWAPNEPSNDPTRQCVYFDGRGADTHKAWTTDSCSTPRAFMCQKHRYDADHKPNVIGNADLPAGHWYATVKTNPTNGNSPSCSLSVRVQSSLQIVTGFTASIGDDNPQIDPIQDSSNNRLIAYIHSADNENRVPILTDAMLWDFYNGTFYNGMKYQPRYGCQYGWVSQNFPCPNSDNQNNEFGVLHVGEDEFGNTFQRLTFGHCSPAQITCGNGGIRQGGQCVCTDYWTGSRCTVPFCVNGGTRNDDQASCSCPSGYTGPNCQYEVCQPNIPQLFSDDRKTLLLVVETTRQNSATVNQLIANLKAIVTSATSSTPLWFTNFGLVTFDTTGRTFEKFNYTTIDALITDLTAQSNAISTDGACSMPYLGVLAHLLEHDDVIAMPNSEIFLITPAGPSDLNKYGETMDTLFNTQAHLHYILSKTANCPTFDGVNNVKDMTWLGYGSSGNILFTDSDNLVSLLNVYLPSLYGASVLQDPTGAANYTCSDGSLPWFVPVDANTTFIYVSTSAEFGSLSVKDPLGLAHNIAPAYSVGDQKIYKIEVDRIGGIWTLQLVNPPGLCLAHVYSTGGAKVYTKFSLPMPVGSNTDLTGAHQDGRYVQPVAGFENVAVFHLSGNAFHAGQLQYVEIFDIDNNGGATNILRSELYRREQCSYEYYSDLFTCNGDMIIAFIHGVDENNQKFRRQEVVICNGRNPTTGQPVTGTMSPVTQGTVTQGPATQATNTQGPVTQQPPATTQTPPQTMPTATSPQNGLAFDIVFLIDGSQSAQNSFDQFTKFIQTMMVSFDVGMAGARVGLVVVAPDLDDQAPPAAQLNSITSQTILNSNLALLKDNYADFDHAGQVLTYNLQVVTSAEYMDPTAGYRPDISNHVLVYITTTTSFYTDPTPSAQSIIAKKQYGIITVGYGAGFDNGKLQTISGGAACSFTATDFTTLNNQIKPIQQLISNAATNGGVYCKNN*

>Cbn|CBN02677.1

MGSIQFFKTTKLAFEGSFPGFCGEENLVRFLLVFYFFGLFCLRLLEDKIMAYFDSNINSVKKVGPSNSDYYGAMTRCTALGAALTGLQSNEERFWIDQEVNRQTMSSTIRDAGTWLGAQKIAGTNQFQWTDGHTTGLVTMGWGPKQPDNSNMRGRGSQDCVHIISLNTGYTRGWTLLGFVTGQLDDINCGYNDDPPQNFYACGKKPS*

>Cbn|CBN03026.1

MLLRFSIFSLLLLHLVSSIFIKEPYGSDSESRERSYSGRRRHKHKYYDGGYYDDDDNRPPRPPPRPTCEDGWTFFQRPSGGWCMKVYPGTLNQYNSETMCGSQGAVLSGLQNIAEVNWLVSSALTVIAPQTSGGVWIGTRRRPECIGSGQTATCTKTNTFYWTDNSTTGIEGMIFQQGEPNNGNSPNGQQDCALMTVATTPTINAVSTYWSGQMDDIMCIASGTINWPSGTLPRANRAYVCGKKAGR*

>Cbn|CBN03092.1

MSTSKDELPAFQENTEPLLTSAAQRKRRNDFMLGFFVSSIIFLILIGVGAAAFVLYGLNNKIDQLINCTATFPVETMTTTVPTTVETTTENSINREMEEMEKSMEQAFKMMGMIIKGLQGLSVEENTTKSTVSTTSSENSSSELRVPELKYGDCDSEWVTVNRTETGKKYCHRFFPHKLTYEEAEKKCQVHGAHLSGFTGQSELDVLDKMLDKAKSEGVQFDNGDSVWIGARRRAACSTRQGIGEKEHGFNSDPTHPCSRRQVFEWVNGVAQNPPEFQKLWLTDFEPNFAGDVENCVVLLKGVKNIGYWPRDILGDKKLNDVPCYLKYYYFCGKEAPIVKTDE*

>Cbn|CBN03103.1

MIRLPIFLFFAGLIGLTVGAECPLGWTFNPVSSECYLISTRLFTFDESVQYCNSIGGKSVSIGSYSEKDAIVAFTNSTLLQPWIGSRRNTTTTKFYNLNGSYFYTLMWTTNEPSVNGDCVTFKGATPSGLQVTQCYQLQPAFCKQTPALCNSAVIGGSTTWSGTFQSPGYPTQYYNNLDCRYLIIAPNNTYITIEFYPYVVEEWYDYVDLYEGNSTSFADHIAEVSSTGWARGYESEGNVMNVRFKTNYAITDKGWLATWKAKKDMPVISQSGTNGTMVSPNYPNNYDSNDEQKYQISVGFGAQVNLTIDDFRTESRFDYLNIYNSSTQSNSTLVATLSGASVAPFNYISPKSYMSMKFVSDGSLQYKGWHAYWSIC*

>Cbn|CBN03222.1

MKLFLLFTFLTTAYADIACPSGFTLVLDKCLKVIPNPLTHTKAEVDCTYLGGTLAAVYSAINKTWLGLFCFSEQNSSSCYHDDNSGTASVYNNFAPGYPLINGIYGGCVYMSTEGSLAGKWVSMNCKAETLPFVCEVPSTIYDPTCTHNYDGYCYYPSTELKTTQAKFSNAQSICQQEFNGNLASIHSKRENDYFVSLFRKIRYADNILLGAQELLPDTFSWNDGSNYADFDNRNPLDTGSKGCLVLVSSTGLWERVDCSFERVFLCKRPLSETTTPTPIAELVTSNPSDFSNCNTTFLMAPGVITSYGYLSLSADTTYCTWRIVTLGAYRIRLSFVDFNSPNPVYVYDEYGKRTDRISSGTCVISPTNIMNVTFQNSGIAGYRGFRAVAQPY*

>Cbn|CBN03938.1

MKLLIFFTAIGGLYAQTCNTGGIYSAQFNRCYQYFTAPAQFEFAEEQCALLGGHLVSIQNGAENALIQSNGANSFKKSNYSDYWIGANDLETSGQWKWTDPSVTWNYQNWALGEPQSGSDCAIQDKGDGTWSAIGCTSYRPFVCVTPVIITATCPPITTPKPAICPTYAPCPVKNCVPSCDQGWTYFAPTDFCYRVYHGAKFDDAEAACVLLGGHLASIHSLTENTFVNNLASCGIKEGKYQNLAWIGMHQVGKDWVWTDGTTTDYINWAPKQPDNPGKELCVQTAPDVSHDSWYENWNNLACNTIMRAYICKKQSVHS*

>Cbn|CBN04142.1

MMPRVLFYIFLLTTFAYSKAPCGSSWLHIAHLDSCFLSAPQPAEFSEAEEYCNNLGSSLVVINSEEEGSIVREFFARENPSFFNWIGMRWNERKSEFRWIDDKKRNYTYFLPDEPNASGECIAWVLDQNVDGWQSISCHYSQFFMCQKAAEGIITTWHRNDEGIITSPNFPNQYDNLEYDTHIIKSEQGTRILMYFEHVETELNCDIITVSDDYGISGRTLFRLSGSYHNYSVISNRNYVMINFKSDEDGTGKGFFMRYKIIRPLPTKVFSSNSYGTVTSNNYPNSPDSFLIQYYLVQCPLETHVTLTVKAINLDRNDRVKVYDGGDETSKKLRIFRQFSSKSTVPIRTSQNNMFISYDTGEQFNGSNHWAFEYNCEPDGNLGDEIII*

>Cbn|CBN04543.1

MRSILLIVSLACFIAVESASTPVCTNGFTLISNKCLKLFPSAVTHKVAERSCMDWGATLVTVKNANDNHAITAIAASSSLVWMGLFCLDNDPARCLWDDATGSAEMYSTFASGFPHIDVGKCVYYSVKGALAGQWLSGDCEKDTKAYICELPVTVADNCPNNYNGHCYTFHPPSSFVQAQTTCEKECGNLASINSANENRYINTLSNRQASGNLYIGATWPTKDVFNWIDGSLWTYNNIDLSYSHGATCVAMINGANIGSSGMWFSIDCNMQYSSVCKRPAGIKCNGAAPAVTVTPVPKNPSYCNAGVQLTPGVVTSPDYPYQYDNNQNCVYQLATLGSYNVLLRFSDFGTESKKDIVTVYDGDSVNSPVIGTYSGELNAFSLVSTGNTMTLSFKSDAKNGGRGFSARFTSYAYGK*

>Cbn|CBN04672.1

MKLFLLTTLLTSAYAHLACPSGFTLVLDECLKIIPNPLTHTQAEVDCTYLGGTLVNVHSAIKNRAVSEFASKSGLNKIWLGLFCFSNQNSSSCYHDDNSGTASVYNNFAPGYPLINGIYGGCVYMSTEGSLAGKWVSVECEAESMPFVCEVPSTRYDPTCTHNYGGHCYYPSTELNTTSATFSNAQSICQQEFNGNLASIHSKRENDYVVSLFRGKSDADYVLLGAQDLLPNTFSWIDGSNYAAFDYRDPLDPGTADCLGMEMGTGLWKRVGCTAKIVFLCKRSISKTVISTPKAHLVTSNPSDFSNCNTTFLMAPGVITSYGYLSTTPSATSCTWKIVTLGAYHIRLSFVDYSSPDPFSSNRAVVSPTNIMNVTFQDRGIAGFRGFKAVAQPY*

>Cbn|CBN04817.1

MRVTLVSALVLLLGGPSLQGPIVAQKPRKVSPGYNVFERPDGTVVTHSYHDTLATFDEAEKICDAEGASLSGIGSMEEANFLKGLVKPVNSEGQYWIGGRRNPSCFHVKGYVNKSGDPCSRENVVTWQNNVAKTIKEDWWRDHLCDSKDCVKIPNPTSEVYDEEKGNKGFQACLGFVAGNPSWADKNSSQFLDDKECDSTFGFFCQRPLVASYRLFTDPVTPIEAEENCEATGGLLAGINTEKDAVDITELAKEEGSVNGLFWLGATRNPSCFNVSGYIDQVGHPCSRLNVMQWSRGVGTRVKEDWWRDHYISTVKNPSSNAGQQDGDHGFQRCLVFVHGSHQWAKDNSSQFLDDSECNQKRGYVCAYM*

>Cbn|CBN05279.1

MRLTLVIALISYHTVTNALECYDSSERLIGDACFSLVNERKNFYDAQRHCQHNMKTWSNLAVENNKSQMIFLTRYAKTDFDETTGWFWLGVLRMETNENFKTVTGLPLYWQNFKDINPSMNYVAARLADGSWDTKPLEEKLPFACSYVPLPRKQAREAESTSSNWLRESVYRSIDS*

>Cbn|CBN05294.1

MRLLLLLLVFGMTATLGHVIEGKSSISFDNPSFHSSSSSESSSNEGRGTNRHRHLGRHGRRPPRPIQNDHSHSHGSHSHEHGGGDNDGGGENRGCGRGWKRFDRPTGGWCIKVAQGQFSQAQAESKCQSEGGTLSGLQDTNEISYITSAALLLFPERTGSLWVGARRSPACSSAPLSSSCNRMTSFTWTDGSTTGTAGFQWAANQPDNSHFKTQHCAVLLASSPSSDPTWNWLTDKLDDEACVNPTGSYQRIVRGYVCGKKANERRRK*

>Cbn|CBN05330.1

MWIPLLILCFWTMGISTLAPPREPDYSRIAGDPKTSYFCTNNFTLVFAKCLRHFPEPTTRMTAMKTCRRYSGNLVTIKTRDEQASVKHHLGAEPRKYWIGLYCFTDNVSQCVWDNGNATAQALRHFPPFSPSVKLGNCVHSQINGDGKEVWLSTNCEEEKLEFYCEIPVTLEDHCELNFNQHCYFPSVLAEKGIQNARNSCKQPFGADLVSIGSREEHLFIKNYYKTLNVSSIFIGARAWLGRYFKWMDGTSWTYTASDRYNIILGDCMVMNLKSDGNRTEGYWYGEDCMATRHFMCKRKAGQRFLIQKPLYKPPRWSLRLVSSYMNYGTITSPDYPEGYGPNVTAVYKLWTLGSQRIQLHFLEIQTEKDHDFVRVYDGMSEESKLIGNLSGQHRDLFLESSSNRMHVVFKTDLQGNDLGFQANFKSIV*

>Cbn|CBN05589.1

MFNRISQIYQARQDRLSRQFENQESLASNRNSQHSEEIQSASLENAAATSTEGTQDAEPKTEFEPVERQRHFGILHYTMNNRFKKIMLIGLANLLIILAFFLFMFFFMMRFRCGTLDISTTTTIAPTTTYTTRAPSSTNAPQLKTCMNGFQLVGSKCWWINVRVYNRDTAVETCRTYGSILFTIDNEAEDLAWKAFMNNQHHRCWMGLLCKNSNKETCEWDNSVKWPFAYSAFAEDSPSDTLPCIYYNVSDSLGAPWTSSDCSLNLSSVCELPPTGESDCLYNYKGFCYYYYATPVTFSEAQDACKTKCGNLASVGSHLEYIFLSSIEGVQDISIYLGGVVVNQNLFYWLDGSPTNYNNIQKYDEKSSCLVLNMGQKANNGNWSTVPCTSKNPYVCKVPNDVICN*

>Cbn|CBN05591.1

MLRALLLLLFFLISPSFSLVCPPEYILVNEQKCLKIVWTPATHWEATQNCTAVSGTLVTIKNAIDNRAIVNLLSGTEIQEIWLGLSCFGNTSSTCFWDNGSGSPILYNNFMGPNITSLYGRCVYMPVANWYGSNQGKWRNIECFNGITPSVLTVLPYICEAPPTTPSQMFPYSCMNNYNGYCYYTQNYRQTYNIAFTSCVMGAVPNLISIHSKMEIDYIRNVYKGTNTTMIYIGAQVDSNGRFYWADGSVWDFDYIDPLDFGEGKCLVMDVQGFGLWSRVDCDQQFEFMCKQKIMVPPTSAPVVSDANVFDEKENAEHPKQLYDASNCNSTLYLAPGSISSFGYPAYTASYCTWQLVALGPYRLGIYFDFWATYGTLTIYDEYGNNIGQFMGTYDRTPFPRYTPFNIATVTFEPMPKTGSGVDKGFHAVILPL*

>Cbn|CBN06047.1

MITLEDAQAKCKTEGAVVAGTQNLDEINWMADTVKALQPSSVSIWIGATRTNACKDVRITAECTALSSFYWTDGSVVGVSGFKWQLGEPNNTGLGQACVQLYMADKTMDDVV*

>Cbn|CBN06110.1

MLRNLLILLWLSLPILGQNYTCPPNYTMVLNKCLQVFPTTAWGNHSAAEQICRKDGGTLVTIRSAIENRAVATFAAGNNRIWIGTFCFGNDTSQCVYDDYSGPLSGYSGFERGYPLVTNHNDGCVLMVTQGSTAGNWLSSWCNPYEPVSPVCEVPPFIDYQIPTPSCVNKYNGFCYALLQGTYEYGTGCGSSFLSIHSNRENDYIQNNLFPPGQNLILLGAKRKYLVNYDWSDTTNWDFDNRDPMDFNLNHLDCLAMNRSTGLWNYVACNVSQPLICKYPIPDNHPIESHCNANSLLMAPLTITSNGYPRNNNGSPCSYKIGTTGAYRVKISIVDIWGIQLTIKDFDGNLIVQTGIQGTYVSPSSYISVTQTGTGVFRAIVIPY*

>Cbn|CBN06265.1

MAKIYGKVESGPNATFTQGGITLEDCIDRGFTHTNIILVTWIDGVCSGYNSGPITTPILVVETEEDSGNYVAIKTFNANTCPATFNDIDLTFVQVNGDTYPWVKTADGVSFSGCRSGWTRFDRANGRSVCLQAFKLSFRVTKATAISACQQASADIVGVETKEELTWMFEQKNSLVGRDNSFWVDGERVPECLPPINQKELGCNNFKWSNDLTSNVANLSDKNLADMSYYDPYESKAENCLVLFSFHGQLNDASCTQFNSAEGVICGYKLF*

>Cbn|CBN06522.1

MHNFLFSVFLLPLCFNSLLAATCRGEEKLDPSGKYCYVIHKGFASFHDAEKACYDYGGYHLATVPTMIDNRFVYNLSSHANVFSNYYWLGLTDMTADGSWEWIDGSDLNFMNWASDSINGYCGAMRAYDGKWQAQDCAKQYPFICYGAAQGAPTDSPNPPKTTIRPTRKEENMIKFMADSESVGNPNTDPNAVNFYNKEREYIRAVTDVLFANPSSNGNICKFYMSVSFYGYTKYDQQFDHSAAWSQGQFDNLLESNVWDDGKTDQAYNITDAITGAQKFQWSPSMDDIGYTTLVFLTARKDFTNIPSLFNPFPQFDEVVVVSLDGANMPGVPAGVANIPVSKEFNNVDINNLVSVLKCH*

>Cbn|CBN06550.1

MTKELMAHEEAEAHCAKQEQKSTIAFMHREALPFRIYKHFTGVYSLWLEASVAITRDLIYDVKGGHLLLAIDGYPYNLPNIALARVAPTEKAMVLCEYTPRMNQAESNYLLRRYGEIYYPTIFTSDSAYVRTASVLQRNDKDPSMDHKYCKHVLRPFLQTDDAQAAVPTREFLDRSSPAGLEAMSDARAAPIYCQTTFDVMEYGDCPENYKPFYRKETGQKFCHDFIDKPRFYDEAEADCAGDGAHISGFTGREELDFLDSLLDAAQEEDDVPFGHENSIWLGARRREACNRKGIQGKEGGFNSDPTHPCSRLRVFEWVHGVAQNPPDFESRWIGPLEPNFVSDDEKCVELLKGEQTRWDDKDGDKKLNDVPCDPDRGKYYFCGKEAPIVSKKNNL*

>Cbn|CBN06705.1

MFAALLTTLFLALTLKDVTSANLCPTGWTFANETSYCYLISDRYMTYPETASYCQSLGASQMFISVSKELTFLNYFTVGLFAQPWLAITRNVTSNKWYNSDGTTPFSTWWSTGEPGLNGDCATFKSVDPQGMKATPCYSIQPAMCKQMPALCPTTTSYGGLYTRSGTIKSPGYPDQYYNNLDCWYSITAPNNTYITLEFNPYLVEKTFDYCMIYDGANGTGTYLGKTDEYTNPRYEFESSSNHVSFKFHTDRTITSNGWLLTWNAKVYSAPVIQNGTSGNFTSPNYPNNYDPYTEQLYYINAPDGFHVNVTIDDFLTEAKYDVLEVYNTSSVIANNLVANLSGNSTAPWSWVSPSNVVTMRFKTDGSVQKRGFDVLCGSIIAGHGDKISKIDQKTGQIETIRSEKNVKLLTTLGSRWISIDLSNAISVFDADMKRSAIFEYKTVNFTSLLQAESYRNKVCVATDDETIRIINTINGDFISVSEPFGSEIRKVQQSSTTDVLAIGLSSGDIIFHRLNGHTEIGRVKMGAPVAAQEFK*

>Cbn|CBN07064.1

MILRSLLLILIAQVTWCQIGQPGQAPGQVPLDPGYRQPLEPIKNILQGSYGNNVTLYYRNSPYRVSGDLTVEYGVTMDIETGTRIYFDTGIGLIIKGTLRAIGNEFAHIEMLPYQQQINYDDEMPKFRLVDGPTVRQGRLQVQFRERWRSVCTMVTNWTSIDTGTACRSMGYSDGGFWKWYLRNNDTYPFVMPKPDCHGSAKNLWDCPSFSDPNRIRLSENLCQGEDDIGIYCWGPPTFTGWARHWKGIQILNSPFHYVNSDPDLVAVNRESNSRLEFVDILYAGYDGVNKNTTSALYIEGVPPIMNGLRIEHSARDGLQLLDTNGPAIIANSTFSHNRGHGIFVVNTTDARIFVNNSRIQGNWGDGIWYKQKTGINLIDYGLREKRGIGSGRLEEEKPRIDMCSEHRIDDNHFFPHLILVNLKNRTFLDPTQPPICWMTVSLPSRLPYTYSIQWLHVRDRNPRTSRTTLLVCDSNNPDDNTCSTPRFRIPIKNEVYPQSISLKSSGKPLYLAVEHVLDGEQAGYVQGDVNLLFNIHASVLDKAYYGLNVTNCIIEKNTGNGVFANDIRERTALTNVTLDENQGYAGFLVRDGAADIWLNETRIFRNWGDGMNISYAGGSIIVNGTRIEKNRWRGAAIHYNQTLPFLPLYNEVIFKGRPSNNKFYLPTVISENEWGGLLVGNFCAYGNESWWGYRNPPLNMVYPRIEPKILISWVEFLKNQYHPAMEIFSCRDPSVTQNIVDITGNRVDGNMGFGMRIAPAVNMHMLISSNQFLHNNDTTLYVRNAQWPELGKLPGEVTISKNVFKFNFAKYIISIGMNEDAQRQFLTFNQQNEIRANTVFDPFPSLPPRSTPYAALVVSSSNVKIHRNCFNNERAKYEIATELERHAKWIDARENNWGFQEIPRFIDKFFDQFNRYSLASIDIDPYMAACNQRMPYISLLNGAFRQFKKASEPNRLGGIIYENHDLLKGRYTVTKDLQVVPGAKLTIASGSILEFQHGIGMIVQGDLIRNEYDQDEKVIFTSTPFTLEKRPQIRLVNPDGNDEVTEGRLEVLVDDQWGTVCNRSWTPQLTILACNQLGLVADVQYFENWRIFPEEGDLPMVMDNIRCEEQEVDLTKCRHDGVERNCAAGCRRTEVVGLRCLEPRWAGVRYSLLANPPTVTGQTTMDNWRIEKGGLFNFRTSEFCAAFKIDWNYHTFHRLEIRNNFWDGIDVVYNDLVKKPAIRNSLIYNNRRNGFHIRSAGITVENVTISHSAQSGMRYNPSVSALEQNDIVSWLSLKEQPELEANNIFRIPDQRLDLIEVMESNLNQRKFLVAAETEDCPEDPLQECVYNLMIRSVGYQYGLASKMAIQIVNPPSNISDEDAIFTEVSTGKSWSARKDQIYFPVVSTENAMRMRYTRSYGTPKLVVLVLFLDTQEYVDRFIHLYQSRVEDNQYGFSAVHYSNLTFSDGRLSNRWNNERIWLQKVNFTRNTEAVLWLHSPQHVVPQGTPLAEITYHFDNCSVVDNTGPIIESHRDLYASANIFHWILWSNTFANNSRSGIAVALPDTYDLLAKQQHSFWLTENRFERNDDFKILLDGYYAFANISSNNFTSNIAPHRFGMLEIRGMEKKLVCERNRFFFNWGHWMIKIDATSQYLRQIDVPSFIQYNYIEKNKFIRQRADYVDMWPRSYAVGVFGAQKIDVHFNRFYNELMDFELVSGSRYTDIYDTMNATFNWWGTGNEAVISQRVFDFDDWNTYTRAQWSPFYVSNDLSINFWWKPYRDGQLANATYVEPTVHDLHGRVYEDKNLTLITERWYEFPHYYRPFRPYRITRDVTIMPGATLYIQENVEVHIWPNVRILVLGNLVARGSYWQPIRFKPINTTELAEIKGRIPTEFRKKRGIVWVSREEESERAGPEGAPRLNNRLDQPSQPDQPDQPKKALHPEEPNLPNQLTQPDKPKKALHPEEPTRPKRASDRSKPDLVYRDFPTLHRDDPYYQRFTVSLTANGSDYGRSGFLQIYNKTIGETVPSCDRQFTIRNAQVVCRELGLETQNVYHWLTPRWDYNPQLRLIKTYMEPRECRGDEPSLDRCNLRLSGNDSQWMCMDSEHFNYIYCGSNRSLSNEYIGNWGGVTFAQASLEHEFGEKRGSRKEKSILQNVEIVGGGAGHNDSWQSAGLQIFHRSPILDHVNVTNCSVNGVQVISPNDRITLANLNVTFNQGQGVSIMTTFVQAPSTSQDAMRKPMSIPYYSQGMLDMCAAVKRFDVKNRIMLYYKYDSYPVDCVKIFTSSGRRVAFRIVQYHLYSSPTDLGRSDALRLYSSESFAPMSLLAEFRSDYHSVDPSQAVSSEEIAVHLRATAADGVYGFIAEVSALPSNSEQHTVGEVIIRGSRMDNNDRGAIEYSNLGEMSPNLVIESSSFSFNGIHLFGNISTSSQAIQLHLHNTVFFLFRSNSIAHNRGGLYISATSSSPVVRLGALVKNCVFVYNSNSTTLALSGNNYQSISLLNNIISHNFALYHDTIVAHDVAINMTRNTLFSNTGLHTLDIHANSKTSADKNVFFYNHFYDNLALGHGHQYMEKFGYQPLRENNEFLNRPRREARDRRQVLTQQGVSFDWWTHVDNETTRYRSTIIAGSSQEIFKFNTFNDPLNDYELTTGQQSQYEIGSIDAKENYWGYPGTVGVATGKIRDQEDYPALVKVDFMPVLESNTSLIEGDCPAGWFQAGHNEFKSCFLFVAAAVTYPRAVEYCKDLGAFVPYLRVEDILQTQLAQRVEKFSIDLITDQERLKAYGVEDDIHLWISSVNIPNTQCGWLGARSKRIGEVNCNILLPFICEKGTHPYSEPILWRPGIIIPIIIAGVIVFVLFTLVVCWCWKSRKRNEVMIERKNIVRASLKLQKREQEYQKRKMQQMQSGSEHTHTSAHASLDGGSTLSAYDWRSGRIPPRPMLRTPSDTLSTATSDHTFSYTGYTPHGGPTTGTTPSSRYRSRYVSTNPNGGYSDLTTTVPTTVTTSTETTVVRMRPHEMRSETSATESCSTCPTSDSERTSTATDISSYTSDAGSESTVRSTVVNNRRSLLPPARSPIPALRRGGSNGFLNMTNNFQPLPTEPPPRLPPTNHQPSLNRFVELHAPPRTGGGGSLNRTKKPVIETSM*

>Cbn|CBN07304.1

MATPYHFVFLFLVGAPLVTPTVHDRSCGNDTNNYWLDVVLVIDNSENMINTNVYETISSIFGPKSQIGTGYKDPRSSRVGIITYNYNATMVANLKAIKSLQQLQNQLTTLKNAGNQVFEDSDQTNLDTYAMTSPDAIARALRSNFVNIITVNTGKDKTVSKQLATLASNKMAYIMSENITAELQNALAAINCFCPDGWVPYKHYGVCLYTSSQTKHRANANQVCQSLGVPASLPTEFNQKKREFNYELLNSGNLRVNSYWNGLTNQNGSWYWDQPKGKPLIPLDPSCGTVPPNSVCVADVEFSDKTVHWSSVNCANTFYFLCEAATCDTDNYCD*

>Cbn|CBN07397.1

MDNVVGDRPPSKFSFGYARLHWKCYLVGMSFIVLLIIAFFLILFLAILKHPSTPPPITPTVLPNVASTVTSRMTTATTHTVAPRKTTVTKTTQTLGTRTTTRIRTIPTRTSPTSTKTVPILTKSSPIPTKTLPTSTHTLPISTKTLSSSTGTVPLSTNTLPLSTKTLPFLTETGPTSTVTPVPSTTVTMTPAIATSASTKSEISTIMSTRESTAENPCIYQYNDHCYFPVNTSMKFGDAQKYCSKNCANLPSINSALENQYINTLFKTETVVMLGAVATSKTNIIWTEKMDHGYNNIQNFTKENCVFTTLNSNDGHTGHNNDNTSSLHSVSKESLPDEIQNERTVVEVVTTEGAPDFSPREQQGFFGLLHSTVNNRFKRILLVGVANVMLILSFLLFMLFFVLKIKCAKPEVIFETIQCPTSSVFVSTTVATTKKPTVLLICPDGFTLVGQICWKIFPDYLDRTSAAEMCKTYNESYLFGIYSAEQNQQFIDFMTIKDHPCWLGLMCTNGNQGNCSFDNGLHAPFPYTNFATGSPDASTECLYYDSFHVTETLWISEKCERTLPFVCEFRPKVVTVNAICDHTYHDYCYSVHDTRLSYSDAQDACRNECGNLPSVIDENENKFLISIKDDSEIWLGGTVMSQNSVIWGDGTSMAYTNLTYYDSKQSCIMMESSGSWSTSPCGALYRFVCKVPIDANC*

>Cbn|CBN07565.1

MLLRFSIILLLLLPLVASIFYGGDSGSDSESRERSYSGRRRHKHKYYDGGYYDDDNNRPPRPPPRPTCEEGWTFFQRPSGGWCMKVYPGTLNQYNSETMCVSHGAVLSGLQNTAEINWLVSSALSVIAPQTSGGVWIGTRRRPECVGYGQTATCTKTNTFYWTDNSTTGIEGMIFQQGEPNNGNSANGQQDCALMTVATTPTINAVSTYWSGQMDDIMCIASGTINWPSGTLPRANRAYVCGKKARQ*

>Cbn|CBN07604.1

MYTLVLLFTLFQNALGQCVLGDDAYVGGLCYTASTYKATYSEAEFTCNGKAQSLAIIRNTAQANVLATLVNKITKEPNGLFWIGLKRKTMGSRWQWEDGTPFSWSNFDSNYLQNNLYVAESTMNGKWRTLDGEESHYFVCSYDPNLVNPTTSQSETTYYPSGSTDYPYETTPYPYTDSTYYPGETTSYVYPDGTTSYEDPGTTSYVFETTSYEDPGTTSYADPGTTSYEDPGTTSYVYETTDYPDYDSTVPDVDDSTPNPFDSTTDFPRLKKQLNKLPEGIKQLLRKYNF*

>Cbn|CBN07748.1

MADLNKIQSINDLKNTLFNGTISETYESNLAKGLEAAEDMLYYGSVDKKRDYYQRAIIVYASVFNSQFDPTGVANRLKADGINIITIAFDEENQVSGELDWELSKVASPRYNFSNFEDENILDQIRGALLESNCFCPNEWLQYRNSFIDEYSKPFGTCLHYLSLTASWTAARFACRHIHNNSHLATEFDKAKHDYIFQMVQNSEQPEIYHIGLNMVKGVWKWDQPPGLDQPNLQGWEGWDKGYPLTSSTKNAVMNKIGAAGTVWQNVQAMSVGGYVCEVAACDTDNFCDRDDVN*

>Cbn|CBN07901.1

MKSLVLFLLTLAIAYGYDNTICKNGFNLVNNKCWKLFQDPANHTTAENTCTQYGGSLFMAKNAIDNRAILSFVGGYQIDSLWMGIFCIGSDKSQCYWDDQTGTTMMYDNFATGFPNSGTGRCVYYSLSGYPRGQWLNEDCTEKMQYICELPTTHSDICDLNFNDNCYFRLDAQPFNVAQKQCEQMCGDMVSIHSAEENRFIASIYQDLPYDYIRIGGVATSPDFIVWTDGSTMDYNNLEKIGVNGICLYMALKNTYYSTRGAWYAFDCSTPYNYVCKRPVGAPNCRSTPAPPPPPQPTATATCTSGVHVAPGVISSPGYPHYYTSGCQYTLTTFGPNKIRLTFDYCNISPNDYIYIYDGESEYAPLISKAYTQAGKDPCLHQYDGHCYFPVNKSMNFGNAQDYCAKNCANLPSISSALENQYLNSIFKTETVTVLGAVAMSKTNIIWADLSEQRYNNIQNFGKENCVFTTLNSYNGQTGQWFTDDCTRETFFVCKRSIGVECNGGDF*

>Cbn|CBN08074.1

MKLLILLIPLFQQISTEDICTSGFSYFPTFNKCWKAMTEELAQPDADEACRKLNGATLAMINSQIEYNALKSFLNNVVDNYNWIGLMCNGTTTASCHWTHDRGAAEDYYFKTGSLDPKTGECVYFDKSNRKLASMMCSYKTQFVCELPPTTTDSCNNNYNRNCYYVIQNSANYTDAQATCEESCGNLVSIHSELENRYVLSLFSSAGVVSLGGMAASKNYIIWNDFSVQLYNKIGEFEDGGCLFMNVGDDNSGVWHIDVCRPPSKFSFGYVRLHWKYFLAGAIFIALLITAFFLILFLVILKPSSSPSPVASTILPDVFSTVTNLLTVSTISTGTPVTVAQTTQTVVASTAKNPLPTVPTSTRTAVPLTTTVNKGSPGATTTVVPTASTQSVLSTVTSTLAPTVQSTSTKSIGNSTTAMGSTQHILSTLIPDSPTVTSTISMNISTTPGPTPDPNKVCATGFKLVNNKCWFLGPYTVYRDLGDSLCFNQAGSSLLSIKSAEENEKVAEFVAGQGIDKIWTGLKCFTNDTSSCVWDYGQGDVSGYSNFAVGSPNITIGTCVYYLAADKKWYSGVCDSDNWRYQMFCELPSTMRDTCKNNWNNNCYIENGQALFFSDAEDYCKQQCGTLLSIHTPLENRYVSSIYQLYYESRGGSLILGCVAPSQDMIIYNDYTPINTYNNLKNFTMDQNCVFFDIPSSNWYSGDYAEEECRGYNGATLVKVDSITKLKGIQQYLGDMLYRETWIGLYCNGTQPTDCYWTHDRGILDFDVFGKWSPNATTGNCVVMNVKDGIWSSRDCNQRTDSFCELPATTQDDCANNYDHHCYTLNQTSLSFSDAQRTCQQSCGNLVSIHSELENRYVSSVISQENGTVSLGGVAPSSRTILWSDSSMPAYNNINQYGHGYCLFTNVSSSYDGYGYWFSDLCSKKSWFVCKRSTGIKC*

>Cbn|CBN08581.1

MRPFLLVIGAFLHFTSAATPVCTNGFTLVNDKCLRLFTSTVTHKVAERTCMNYGATLVTIKNAIDNRGIQTVTGTFSYTLWMGLYCFGNDVTKCLWDDASGDASLYSSFMSGYPQVQTGKCVFYSSQSSTMGKWYSSDCENTTRSFICELPTTYQDDCGNNYNGFCYTHNSAAPFITAQETCEASCGNLVSIHSANENRYLNSLFLQSGSGSILIGATRTSRNSYSWFDGSLWSYNNFDVTALQTGNCVAMSFGTSNNVSAGSWYTVDCGSSYGYMCKRPAGVQCPANPPPVTVTPVPSNPSYCNSTLLMAPGTITTPNYPRYYDNNVYCTYQLATLGAYNILLKFTSFVTEAKFDYVAVYDGDNFNSHLFGNYSGTPGPFSLVSTGNTMLVTFRSDKTNTFAGFSASFSSYVFHS*

>Cbn|CBN08626.1

MKSLIVFLVLCFVSTHASNNTICAKGYNLVNNKCWKLFQDAAIHADAERNCSADGGTLFMAKNAIDNRAIASFVSTHGIDHLWMGVFCIGNGRNLCYYDDQSGSTLLYDNFAAGFPSSAAGRCVYYSVPGHPAGQWLNGYCNQKLSYVYICDLNFNDNCYFTIDAFSFSDGQQQCEQLCANMASIHSSEENRYITSLYSKTSYDFILIGGMAVNGFVLWNDGSVMDYSNLQTSDSTGCLCMSVNNSTRGSWYLADCSTPKHILCKRPIGVPNCRGAPVPITSPPVTPPTCNSGVHVAPGWITSPMYPSNYNAGCQYTLTTYGSNRIHVYFPIAVLEGGYDYVYIYDGDSKDAPRLDYWTGTNQAKGYYSTGNTMFIDFVYRSGSTKNQGFNATFISIF*

>Cbn|CBN08960.1

MVTSMFLLISALILMVNAQCRFEDSPIGDLIFFIFRCYSFSKTPKTYTDASNYCHSINQNLAVVHNSQQNNFLFLKVFNSPRFPDNVRARSESSNGKFWIGLSRSSINSPFVWDDGTSLGWTNFDQNLPKDSLFVFESTVNGKWATLDGTQVLDTVCSYQLNSTTKPSVTCLFMVDMQSSGITQTAISTYQAYYIFAQTVAGKLNDQSNFSGYLDNFGYSGGLADHQEYPADLYTDFSNIAFPIDGIDDDIDLDLKDVDASLNTAQWTPHVDDQTCMIFFSAAPEAMYGGSSIQHTYDSFTTVIGVLFGAATSIPGLTDPIVASTLSDADAEAVVQKLLESLPSL*

>Cbn|CBN09305.1

MKILQLALLFCFTIYAYGSTKVCAPGYKLVNGNKCWKLMNNGKTRDDAHNDCGVNQHGGLLAMPENSGDNEALKNLVKNSGHSNLWFGLYCWSGDKSQCQWDNIDSMGYDNFNSGQPNGNTNCVNFLSATGRWGSRDCGHTLPYVCELPTTTQDCGANCDTNFHNHCYKLVHSSKNFNDAESQCKSYGMHLVSIHSTLELEFVAHMYADKGTYWLGGKMPVDNQITWLDGTPQDFSPGTRVLDGACMQINIDSSHNDINWLGKNCGYTPSKFLCKRSVQAC*

>Cbn|CBN09354.1

MIRLIVLLQLVGLALAGPISVTTVAPARCPDGFSLLNDITCVKLLETPRLYQDALNSCKNFDGGNLASVKSEMVASLFFELCLTFIFEENSFLVNLAAVKNSVHPIWLGLICYSSANSSCNWADATGTTAGYSNFARGNPKSSSGNNVYLLTAGSSSGKWVSADGNVVTLNYFCEVPATGVETPCPNEFNESCYIYNQTLLSEADARISCQNQFFGELVSIHSQEENDHVQALIRNTSLVRIGVRTNGVGMYWDDGSYFDFNNFASFRPDTENCSAMQILQGSVDPGKWLSDSCDEKIGFVCKRLKGHSSRSPNPGPTFNPNNCYGTHYFSGQGEIVSPNYPNSYFGMGTPCTYIFTVPSNQIAQIKFRVLKLDDQSTISLYTGIEGTVPIIELTGSTSAGSGSYSSGSNVLKMVFKNEKRKYDATTKWSANYGWNL*

>Cbn|CBN09565.1

MKRFFTLIFVFLALLPVILTTPSCPSGFKLVNKNKCLKVFSEYDKHFEAESVCRGYGGTFATAHNAIDNRAIVSFAGNAALAYIWMGMFCFGNQSSTCYHDDGSGVVKYNNFAPGMPKVDSTIRDGGCVYTPAFGNDAGKWYSGPCEWTGIEYVCEVPVTKNDTCTYNYNGNCYFPSNLLPIEDVDRNFYDALNICHTHSMELLSIHSRREIDFIKSIYKNSGIVELTLGAQAIQKNLFDWTDGTNFDYDNFDPLSTTNGSCLQMDLSERDDIGMWYQTDCLTVKYFMCVRPAGSTTGAIEISTKEKRVNPKFQATRFTPPKILDLSDSSNCNTTLLLAPGVITSLGYSDNKPLIAYCSWKVAVLGPYRLGLYFTDFSVKNPVNVYSDNNTLLDSFDDNKTPFSLLAPSNLVTLTHDSVNDGVNNYHGFSATVLPY*

>Cbn|CBN09662.1

MSTVPKDINVGRWLSGPCEVAGFAFVCEVPNAVADPTCTHNYNGYCYLPSHEIPNITATATFSQAQVICQSLSSNLSSVHSQRENDFIKSLYKNSGFNSSYLGGASFSSLGAKWLDETNFDYNYTDPIGYSNGTCLVMDISSRVNNGMWSGMDCQTPNHFVCKRQIQDTTANVKTKRLELTEGLVATASPDLSHCNSTIYLAPGVVTSFEYPNSNVPATYCTWKLAVLGPYCAGLYFTDFSFF*

>Cbn|CBN10037.1

MKTDPTTIATTTIPTTTKPVVFSIVDPGALLIDCPDACATGWQYYNSKCYKKFNTAGTYAQAISACQAQGAELVTINDFDENDALRKAFDTNALVTEAQETWIGLKATSGVYAWSDGSSASYTNWAQNQPAAGSGQCVQMITDALSNKTYQYQRGGWKTYGCGKTSASYICEAPAGGPSISLVGR*

>Cbn|CBN10379.1

MKSLIILFSLLLLHVSAQSLKCPDGYTMVLKKCLQAYPAPTRGNHSAAEEKCRKDGGTLANIRSVIENRATSVFASNNNLDKFWIGTFCFGNDTSKCVYDDDSGSVSDYNSFAVGNPQVTGVSDGCVYMLATGPQAGRWISSKCKTDEAVGSVCELPAYVDNPGYLQRSGCSYFNGYCYIRKSGPVSTMASTCSQIKGFPLSIRSKRENDFVYGLNSHNYDLLLGATRIAYDENKSISKYAWMDGAGWNEFENIDVTAHNYNGNACLGMDRWTGLWKSVYCGTSYFYYCKVPLPPNPAVDTSNCNSTVLMAPAVITSFGYPDNNSPPPCTWKIATVGAYQLRIAFKKGYDSIVTVYDGYGNNIGTASPDSSIVAPTNYITVVQTGGGPFYADILPY*

>Cbn|CBN10724.1

MLRLFFFVALCIFGSVAPNCLPGDVSFEQYCFSFNRVFGTFNDTNALCNKMVGGSLVKINNMIENNWIQKWAVVNLDADYNLFWIGASDEGHTKDWRWRDNSALNFNNWNRGEPLEDRHCGAMALSSGKWFSALCTEKHQFLCQYPNSNYPTGAPYTCPPCPSFSK*

>Cbn|CBN10901.1

MPTGFTYSWTANTTSWSFNGCRDGWTRFDREDSVSVCMQIFLQNNIAYNTSKEVCSAQSAVLTGLASLEECKWVQARVFQITTEDWMGFWIDGVRNCTDGQTCQTFAWTDGYTTNYDVLYDSTNADLAVSPAGPAYECCLTVGYTSPQTINDDKYCLMAYLDESVECYHYRYTSRPKTIVVKELKDERVVAIKAIIPPDLLSTCPSTHDSLDFSMTIPSGDTYSWTKSLDGYSLNPCRDTWKSFSRTDGNMVCLQIFQVNNIDQPAAKKFCMDREANLTGVASVEETSWMHETLEEIPPVNEFDSFWIDGERQCAEDYQCDVSGV*

>Cbn|CBN11122.1

MKSLLLLLFFIAVSSAQNCLSSADRLINKRCFTFVNQQLSYQDARDWCHFRNPVTFSYLATVPDANTANFLASIAHTLFNNNGGYFWIGLSRYNNKFQWDDGTPVSWTNFQLQNSQNYVAESITNAKWTTYPNDNKYNFICSYDPNGSQTGRPTYPPDTSVAPWGTTAETETPPGPTPTGKPR*

>Cbn|CBN11239.1

MMNKVLLATVLFGAVAAFDFSSSSESCEDDSHGHGGGRPPRPPGGGNNGGGNGGCPAGWRRFNRPNGGWCMKAFGGALTQANADAQCRSYGGVLSGLQNMEEARFVSRSALSVISRPSGSIWIGARRRSACLRQRITSSCTALNSFQWTDGSASGTAGFVWSDRQPDNAYDLTQECVVLTAAADTLKVKNVDWPVAMLDDTPCVLPLTDPSPRTIAGYVCGKAASRR*

>Cbn|CBN11518.1

MKLFLLSTLLTTAYANIACPSGFTLVLDKCLKVMPNPLTHTEAEVDCTYFGGTLVNIHSAIENRAVSEFASKSGLNKTWLGLFCFSEQNSSSCYHDDNSGTASVYNNFAPGYPLINGIYGGCVYMSTGGSLAGKWVSVKCEAESMPYVCEVPSTIYDPTCTHNYGGYCYYPSTELSTTSATFSDAQSICQQEFNGNLASIHSKRENDYVVSLFRGNPNANYVLLGAQELLPKTFSWNDGSNYADFDNRDPMDASNMNCLAMVTGTGLWTRMGCTGRIVFLCKRPISETVTPTPKAELVISNPSDFSNCNTTFLMAPGAITSYGYLSLTPSTTSCTWRIVTLGAYRIRLSFVDVDTKNPVYVYDEYGREIRRVSYNTAAYSPTNIMNVTFQDSGLAGYRGFRAVAQPY*

>Cbn|CBN11562.1

MLQHVFLASLSFLLVCSYPYTMIVTYGKPSNYTGYNRVLNVLSWEKCVMHCYNLPFCLFAYSPSSTPLECYSFYAKHMLFEVEKTTSANNLLVAFKVVDDKGYCPVGDNAPTFGGAPNYGSVSLGKSNTSYVITPEPGPSWKIRYNATLACQYDWGMFTRPTTKWCIRVFGNAWSESYTQEAALAFCKTQNATLTGLDSIEERDFVAKVALIRITSYNRFAGFWVSGIRKPECYFDGWENIPYCTGANPQQFNHTDNYLTSYSGYTWDPSQPDRSGNCVQMWIRDPALNPDHDYPHGNSDDYDCSTLNRGFACGKLPELTFN*

>Cbn|CBN12021.1

MQNKDQIFKQLPTTQKQRTRLGKHICNIKTRVKEGSEDEYGMTFYNWDGRDLGTTAFEDLPHQASQSTPLPINQSEHCPDGWLKFFDSCYFFETEALGFAKAQKNCQDKQATLFVANSLDEWETVRAHTKKSQFSWIGLVRFSQYERYEKLPRWQTTGSINPSKINWLINPYQPLGNGWSTVANCVALFEFPSTIESSSYTFFYPCTQHFNSICERNSTILNARN*

>Cbn|CBN12109.1

MKCYQFFLLLLSVSVGSALYDPSSYTDRPCGNDISNLWLDVVAVVDNSIGMTTEHLNNIAANIGTLFADTRIGTRPSEPRTTRLGLVTYNSLATTNADLNKFQSLDDVYNGLFSALDRTSNTNTAYLATGLQAAGQLFATQSIGSLRNHYKRVIIVYTSKYQGSGELDPVPMAARFRADGIYIITVAYGDDSKALSDNLQKIATPGFNFTYTSQSLVRDIQGALLQSNCFCPNDWIHYRQSYLDMNSYRYGSCLKPVNLPGSWKQAQLSCHSRHSNSYLVTEFNSNKHDFILTAVKNTFDQPISYHIGLNYINGIWVWDQPTGLPPVPMNGSSWDNWIPGYPKNPHANSGVQNIQTGFGVDWQNIPVYIAMSNYICETYSCDSDNFCDASDVKH*

>Cbn|CBN12236.1

MQKSTLILVLCAVSAMADCPAAYRALLNSQMEESTSNGCAADWKFVNRPSGGWCMKVFTGYYESKAEAEKACQAVDATLSGLQTADEALYIQAAILAQVSESSGSLWTGIQRTKKCIGQKLTATCTNLTSFEWTDGATTGTGGMVFQVGQPDNQGLNQNCALFLASKTPTISARGTYYAATYEDVGCALTTFTPENVGRKTLGYVCGKKANE*

>Cbn|CBN12378.1

MKSLIILFSLILLHVSGQSLKCPDGYTMVLNKCLQAYPALTRGNHSAAEETCRQDGGTLASIRSVIENRATSVFASNNNLDKFWIGTFCFGNDTSKCVYDDNSGSVSDYNSFAAGMPLVTGIIDGCVYMLATGPQAGRWVSSKCKTDEAVGSVCELPAFVDNPGYLQRSGCSYFDGYCYIRRSGPVSTMASTCSQIKGFPLSIRSKRENDFIYGLNSNFNDILLGATRIAYDENKSISKYAWMDGAG*

>Cbn|CBN12459.1

MQTVDPRMILIHNFLDINKETIKNAKTDQVLPQAIQSNLIQNSQSSALQDDSANSLVLMDSTAACDSGWTKSSVNGMCYKIATSDTTWYAAEDWCYNQRYGSHLTSVHSQAEAKWIAATYVTGGSWFPFMDNWVGLRRSCDNTSYVWTDGTPVDFLWWQPGYPGSKDPEQSCVTIWVNTLLKLNPQYVMGQFDDIWDCGTNLATPTCRYDPTSTAPHIKYDTSYTCRDSTTEAAKITTTTMPLTTTTTTETTTTTETTTTPTTTTTTTTTPTTTTETTTTRTTTTETTTTATTTTTTPTTTTTTKTTTTPITTTTTPTTTTTTQITSTTTPIVQGTANFNTAKRGCQAFGGEMATISDSEMNEALRLAFSTNNDSTIAHQAWVGASSYSNWAPGKPNQAQGSEYTEYCNVMTLSVVNSGLDFGFSRGVWSDYPCNFTQEFVICKQS*

>Cbn|CBN12487.1

MMQVPDTVIDEPSGSSMDILTCVTGCLLDSECFLVYSKADGECIRLDVLTPYQVNVTSDGRFVAFKTNLTTCPTTYLGLSFSVIMPSGFTYSWTANSTSWSFTGCRDGWTRFDRADSVSVCMKIFLQNYIAYNTSREMCANQSAVLTGLASSEECKWVQARVFQLTATAWVGFWIDGVRNCTEGQTCQTFAWTDGYTTNYDVLYNSTNADLSISPATSANECCLTVGYISPQTINDINCAASLNLILGYVCGYKV*

>Cbn|CBN12528.1

MKQLIALLCLSSPLVFSFSNIPICTNGFTLINNKCLKLFDYSVPHGVAEKICRHSGATLVTAKSLSDNQAISTIAAKSVALIWIGLYCADSDSSKCLWDDASGAANMYNSFAADDCTFNYNGNCYTFNGTNATFIDAQTTCEQECGNLVSINSELENRYISAMAERLWTPRAAYIGATITAASVSWLYSWTDGSRWTYSNIDNMMYKPCFTMMIGNNYLQASGAWFGYVCEWETSFVCKRPAGLSCDRSQPVVPVTDPPARSSFCNSSLLFAPGVISSPNYPDNYENNVDCEYHLSTIGSYNIALSFLGDFVTEECCDKVYVYDGDSTSSPLIGVYSGTLDTFNLVSTGNTMTVVFKTNSEGVARGFNARFVSYSG*

>Cbn|CBN12533.1

MAASAGVTNIWIGVYCFATGNTTTCYHDDGSGTISYKSFASGNPAVQSGGCVYMSVTGKSAGQWVSAPCEVIALPFVCESPPTVADPSCNLNYNGYCYMPSHAIPYSPSITNYTNAQSICQANGGFLASLHSKPEIDYVRGIYRNAEYSALFLGAQAFLPDTFDWDDGSHWDYDYTDPLSKTKGNCLLMNLPVNDPVRGLWSETNRTFEYPFLCKKKILEQTPVSTMSPVQVKERNLEETLHINKNNLPHHQKLIDFSNCNTTLYMAPGTITSFGYPNSKPPVSYCTWTVATLGPYRLGVYFTDFSTCLNVYLYDELGNQLSNPSGNQRPFRVLGSSNIVKITHDSSYDAMYGYHGFSATLLSF*

>Cbn|CBN12739.1

MIFKLFLLSTLVGLASSACFDNGDKEIGGSCYKFVAQKLSFEDARNWCHYKNPVTASYLAYVPNQFTSNFLASYARSAFGTNDGSFWIGLSRKSGGSWSWDAGFPIGYTNFGDQVGQNYVAESIVNAKWNTFGASDTNFFVCGYDPAAPPTFAPQTAGPSGPTPTMRATTQPVPTTTD*

>Cbn|CBN12850.1

MRFCLLLLICTAVPITHQLLSYDWSLKKACEKMNGQFNQREKDTSTTGDTCSLKFKVATRDDDDSRAFCELYAPWRLLKVSRETVSGRHFTTCHVEATLTCEKGWWQMFGHCLMIPNKGMTYTRQEAEELCKITNPNAGIAFMHHKYIVGIWRRYFKGTGQIWVDATETWDQYIQKTKTVDGTALALAFTGKHYDFSVQSNSLIKIDPNVKLEVLCQYKPEMNPAEINYLGRRYSEIYYPTIPVNNGILVRTTSSYTRTSSNFEVCQKITKPFMFKPIAPFVPDENALEELGMHPKHLIYLTRSGAELPYEKAKMDETICDISKDPLTVKHPNASIGSFRIKNFKEKSQCQNMLSTAIVHGKSPAELRFMSDSRSLPIWCKLGYAQAFIKDNFTTVDGFESFARENGEVIAHKLFTTRMKYWDAKEACKKNGAMLSGINSKEEADKLGELVKKAGTNEDQFWLGGKRRPECQNIDGYDKDPKSICSRNKVIVWEDNVAQEFFDDWWKDGDYHTNPSYWKGEQDCLTYVYGTPGWASSKSEGFLDDVRCDINSMFFCAKKLKFDEKAPDGWV*

>Cbn|CBN13087.1

MRLLLALFLLVTAVAAAPSYDSAFQHLPNYYPELKHQDAIHIRAKRAIFAALGIANSCEDGWTGDGCKNPICTDPRPVPTTGQTALIELLFLKGGCGGSYYIPVDSDVGKTTQTIQIHISAAGIPYVNLTDSKGVVFTPTYANNGDGYSLSIYGDLPSGGYSLTIDNQGVPTTECIVEVNSDTVLKVTEGFVNSPQSDDTPYGESAVDGVPMYVVAHVDSQQAPAQVHSITIRQGNSLTPVYRAPLTRRYQCGYEYYAGQWQCQLGNSYYYHVDGIDSNGFAFRRTGKFACMQHLTSPAPPTTTRAPLTTCFNNGTLLNVVNQGQTCFCTELFTGTQCEKVNCMNAGFPDPDGNECACAAGYHGTNCQDVTCPLNWEPYLTDYKTLVVVIRSVTSMNQNLAAISNAVYKELTSNAANNYEVYKGFVLVKFGNGVYTNTYYPAYDQTKFLTDINLASAAAGQCSDATFDSIASIFTEVAIYQKSPIYFFTDAIASDVEKWQTVIEMNTRQKFPIYTHFFVQDNCLFDDMSQGFQAIEYASYYSGGLILRPTPDTLQHIFQNVIKATAYKMNSVLIDDLGSCATPTRVFFVDTSTTEIMILAIGESLIVSVTDPNGATTTALQIVNSGTTQMYEIANPVVGEHLITVVSNVKNTPCSYRVQARSEYDLFIGTSTGVNDDASDSEPVVGQSAHIVAQLTGLKKNVADPFRLFSEISITSNVNLDNTYQKPMYYSSGKYRDGCGFHMYFGASDFCDFMSQPFYATVYADDGKGFTIQRTTTGFCSGTPTTPYPPNTCQNGGVSDPTNNATCICPPGFYGKYCENIQCVNGGTARGGSCVCPVGTAGTFCEQYMCTTFNNNPDVSFDGQSIAFVISTRSTMKNAVATIATNVQTMTRDMQQASDKWIDKWILIAVNSNTSYLLVNSNRPADFVAGVNNLNGNFTNYAADETSCQIQIEQAMLGAALLSERRSSVWVFTDSDGPNDLNYIQLFDTAQEYQISLNLVGVGSSICTTPENNGQFPYYLKSLSETTLGEVYMTDKLDQIMFFIISLYKSAVSHRYYVPDCKAGTSYYMPVDGWTQSLTLAVTGTDLRSVQITFPDGTLGQNSDYELVAINDPELKLNQYVAACEGSFWNHRKQNCFEFTAEKYDWLDGWNMCHSQKAYLLHIDSQDINDFVFSQITGYRVWIGLAFLNGKWYWDVPDNNFEQPLSGYTNWADGVDPANPKFNHAVMNANGKWEPADPAEMNFGGCMKHRYGQGYYPGEGANIVPAGLWKVTVQSNSGSCEIQARSQSEIQVFFGFVTDPRVDKPNTYANIQSSNNYLIAYPTGVYPYTPDTKPSMEGKLNYAVLSSNRTITNSLPLGNRVCTYATISAPFSCPDTDGSISDFSIKFTGLDQYGYAFERYADALCTKTVISCGNGGFINNGVCVCRAGWVGTTCNTPVCQNGGIEKNGKCDCSSVPQFSGQFCELAHCEPPYPTAFNDKDRTLAIVLETSYNMGSSIFQLRKNLKASLDSINNDPTLQGWFTNFVLYPFDSTSNQASWYPVTVSRNSDDIVAAVKNISTMSCPGNAPCSSQCPRPIVSVLQNVLSMDALASPNSVVLVITRSSPEDYEQVGRIAQVLQDKKAYINFVFPAIDSPCGEGWNNPNVDALYRIISYSQGNTFTMNPVELSKNFLTQYIPTLYSSGGIAASSGNCMNDEIIFQVEHEMYEFSIDFYHPLMETIKVFDPSGDQITIPDNIITSDSNYIGIFPVNETGATRAGTYRILLTGTGGNNCFATVRGRSNLELFLGFVDSNSDSNNGATNDAAHHAPVNLQQNTVVVHATGLGQGIVRYVQIVMPGFGLLHTTEMRKRDTECSYEWYATTPFQFDYDSYWIVIYGSSEFGSNWKRNFYVSTVGSRPPLPPPPANCDLQSVKQDTLFLIDSSLKDTNVTFTILKQFAVTAMQPYNYVNSLAQVAAGSVADKGYWGFSYNAGENSFDRVSELLYDMQYIGVNGQNVTAGLQLVLDFFDLPAQGYRSDPDVRHLLVYVTQTNPTDADPSELVRAIKRSGRYEIIVVALDMQPSDQLTNMVAPRCFYYAQDFHDLMNYGVNFVQGQSCLRWNFCNY*

>Cbn|CBN13470.1

MFQLLLLSILLPLISAQCGPGAVYQASGSRCLTFFRTATDFDSAESICATLNGHLVSIHNTIDNNWVSSQAQKYFSGSSWLGAKTTAPDVTNPLNWYWTDGSNFDYQNYRVGEPSAQGATACMQMQTATTKWLTANCTMQLPFICSYSSSVTPTCPTVTIPSHCPSGYTWYDETDFCYKSTVRFTNFNDARSACQADGGDLASIHSLNENTFLVELSKAGITNKDKSHNDDVFIGLIYQNSKWQWTDGTPVTFLNWGDGEPNNMEKEWWTSLVADPHEDKNTENTRWNNVAQIDMRAFICKRAPLH*

>Cbn|CBN13670.1

MCEFWGGGSTYKPREGQKSLRGDTCSFTLPFASDTKESARMICEEYVPYHINEAVPGVPDTVCKAEATLVCKNGWIQNFGRCYKMWKEMMTRDDAGKHCEKEGATVAFMHREALPFRIKEYFTNVYQFWIDASIAITKDLIHDVENGNLLLAIDGYPYNLPNIALARVAPDVKAMVLCEYTPPMTQSESSYLLRKYGQVYFPTIETSQATYVRTASSLNRNINNPTADDDYCTRVLKPFIPDGKAQSAIPTQEFMNKLVEMRAGGAAIVRTSAFSGNANDKDRKSKSCVNYDKNSLFRMYSKDMKGKYLTIDSSNWKKNEPNEQCDAATYSSGIVLSREGKPGLEAMSDARYAPLYCQSVVSEYKYGGCPDGFKEFHRKQLGQKWCHMFIGNNMKNYADAQAHCQTMGAWLSGYSSQEELDFMDQLITVDGDTLIGARRRDQCSRFGTDYSRGYDEDPSSPCSRKWVFEWKNGVAPNPPDFEPNWAHAVEPNHGTYDEECLIVMKGIRPYIGSNQFNGDKKLNDHRCSDEFNFFCGMEALIVVVN*

>Cbn|CBN13677.1

MLQHVFLASLSFSLVYSYPYTMIVTYGKPTNYTGYNRVLNVLSWEKCVMHCYNLPFCLFAYSPSSTPLECYSFYAKHMLFEVEKTTSANNSLVAFKVVDDKRYCPIGDNAPTFGNAPNYGSVSFGKSNTSYVITPEPGPSWKIHYNATLGCQYDWGMFTRPTTKWCIRVFGNAWSESYTQEAALAFCKTQNATLTGLDSIEERDFVAKVALTRITGYNKFAGFWVSGTRRPECYFDGWENIPYCTGANPQQFNHTDPYLTNYSGYTWDPSQPDWSGNCVQMWIRDPALNPEYDYPHGNSDDYDCSTLNRGFACGKLPELTFN*

>Cbn|CBN14296.1

MSQKDAEAKCKTEGATLSGVQNKEEMKWMGDELIAISGKNGRIWIGLHRTDQCMSGGLTATCTPLSSFYWTDGSTVGVSGFKWGAGEPNNMNGQACGIVWAAAQTMDDMGCDQTVTEALVCGKIATF*

>Cbn|CBN14384.1

MNGQFNLREKDTSTTGDTCALKFKVATRDDDDSRAFCELYAPWRLLKVSRETVSGRHFTTCHVEATLTCEKGWWQMFGHCLMIPNKGMTYTRQEAEELCKITNPNAGIAFMHHKYIVGIWRRYFKGTGQIWVDATETWDQYIQKTKTVDGTALALAFTGKHYDFSVQSNSLIKIDPNVKLEVLCQYKPEMNPAEINYLGRRYSEIYYPTIPVNNGILVRTTSSYTRTSSNFEVCQKITKPFMFKPIAPFVPDENALEELGMHPKHLIYLTRSGAELPYDQAKMDETMCDISKDPLTVKHPNASIGSFKIKNFKEKSQCQNMLSTAIVHGKSPAKLRFMSDSRSLPIWCKLGYAQTFIIDSFTTKDGFESFARENGEVIAHKLFTTKVNYWSARRTCQQNGAMLSGVNSKEEADKLGELAGKAGVKDDQIWLGGIRRQECRHINGYVKDRNSICSRNKVIVWEDNVAQEFFDNWWKDGDSHTNPNYFAGKQDCLTYVHGFPSWASAKSQGFLDDVGCDSWYMFFCAKKLKYDEKAPDGWV*

>Cbn|CBN14703.1

MKQNGRCRKRRLLPEVVFILLLAVFVKPTLSSSKAAPEAQLTDVELKCPEDWIRLGTKCYLPFNIHQSWPFALTTCQRYGSTLAKIQTGNENQFIASLLSKPGKSTDIKEYWIGLTVEVLDDDELYVWSDGTPTSRYVGFWRQDQPNHLNGTCALGKVERKDLEWRLETCNLLRRFVCERAACVQGSYFCSSGSCISESKKCNGYNDCDDGSDEQNCPQAFQPNCRTYEKGESGVLSSPNYPNSYDPNLNCRHVLEGPINSRIELTIEHFETEPDFDVMTILDGGPAENSTIVIKRLSGSLDTAQTITSSTNMMIVQFRTDAQSNARGWQLKWRAVPFTCGGHYTAQAYIQSFVSPGYPKTFSNGAECVWTVETTPGQVVSLVFGHSSIV*

>Cbn|CBN15049.1

MLLRASIFLLLLPSLVSSIFIKEPYGASDSESRERSYSAGRRYKAYGGGGRPPTCEEGWTFFQRPSGGWCMKVYPGNLNQYNSETMCVSKGAVLSGLQNIAEINWLISSALTAIAPQTSGGVWVGARRRPECIGHGITATCTKTNTFYWTDNSTTGIEGMIFQEGEPNNRPAPQNCALMTVATTPRIHALSYYYSGQMDDMICERADASWWPSSTLSRSNRAYVCGKKARR*

>Cbn|CBN15687.1

MDYNGAISECATYGGVLSGIQNEWERWRMAEEALRQTAAYNVQYAGVWLGAQRVNGAFQWTDGHTTGTGGMVFGPGQPDNNNMGGRGPENCLQMIALAPGYWNNPGTWAAWTNGMIDDQWCNQVDDPPTRMYVCGKRGPQG*

>Cbn|CBN15729.1

MNESSLIPTSSLSDSSSADLSVLSDAVGTAACASGWKRYSVNGMCYKESSTSMSWYAAEDWCWGQRAGAHLVSVHSQAEAQWLNSQYKVWYAPMDDWIGLKKECDMGKYFWTDGSPVDFTWWQPGYPKAQYAEQSYLEYSIVASCFWIHTGAIR*

>Cbn|CBN15864.1

MCEYYKGEYKARTGGSKSMVGDVCKFKFSYATDNKESARTYCEEYVPYHIQVASFGPETTCLAEATLICASGWVQFFGRCYKMTKELMTHEQAVNHCATQDKKSTIAFMHREALPFRIYDYFTGVYSLWLEASIAITKDLIYDVKGGHLLLAFDGYPYNLPNIALARVAPTEKAMVLCEYTPRMNQAESNYLLKRYGEIYYPTIFTSDSAYVRTASDLQRNDGKPLRDHKYCKHVLRPFLQTDDAQAAVPTREFLDALKKERVAEIIRTSVYSGDSKVSSRSNEACSASDSRNYGMSFPTSGGSPLFKSMKDDKIWRKDEPKEVCDGASWSTGIVLSRSFSELGLEAMSDARAAPLYCQTSFDVFEYGRCDSDWNTAYRKETGQKYCHRFFSEKVTYDEAEKKCQEHGAHVSGFTGQSELDILDKMLDEAKRKGVSFEEYENVWIGAQRRAICTRLGIEGKVGGFNPDPTHPCSRRRVFEWVHGVAQNPPDFENRWVADLEPNFVGDDEKCVELLKGVKNRWNWPKDPADKKLNDVPCDLKLYYFCGREAPIVKIDA*

>Cbn|CBN15890.1

MRFLISLFLFSTLISSAVGIVCPRGFSAVNNQKCLRAFTNNLKHLEAETDCSYLGGTLVTIKNAVENRGVANLAGLAGANNVWIGMFCFATGNTTTCYHDDNSGVVAYKSFASGNPAVQGNGGCIYMSTSGKTSGQWYSAPCEVIGMPFICEVPPTVYDATCTHNYNGYCYYPSFELTSGTTSTTYQKAQAICQSMGANMVSIHSKPEVDYIRALFLNSGVTNVFLGAQAFLPDTFDWVDGSHWDFDYTNPLAYVKGSCLQMDLSNRPNNGMWSETNCQLTNSFLCKKKISGAVAASTPQPMAGTVPAVVEARVNPKFHNALPHRQELLDFSNCNTTLYMAPGLITSFGYPNTKPPATYCTWRVVSLGPYRVALYFTDFSVYNPVYIYDEFGNLITSPGYNQSPFAILGPTNIVTLKHDSSYDGAYNYHGFSATVLPY*

>Cbn|CBN15927.1

MNLKYILVLAFLCSLWILAIESRKVTQKPKKADPPPPPPLKKDKSWVRSKIDSAKEKYEAGKQKIKSKISSFGSKHGETKVKNAPAPIPQKTTTARRADWNHHMKTTKSYGWNPNIQGQGGVQNSLKTTKQHYGWNSNIQGSGGIQNPPQTGWNSNTNIDPAIGNPFQTTKKPLFDRIKEKAKNSEKYVGKAVGWAKKDLGIGVEGPKKPSKILKYGKKAVEYVFKKKSPKSPNIPVRIAKHPIPGYDSGSLNSNGYSGNVGSGNLGYSGSNYGQTYPESYAPSSERSSDLHQKVEFLRNRLEKMQETIQGTWNTSEAGTKYKLFEEKKNWNDAQLHCEELGSNLAYLDSESKNNYAKFLIDSQNVTMVWFGLRTEVGSEQESQDQFSNFSNLDGCGVVDRNGTWSISTCTMELPYLCQAFRFDVLVEIP*

>Cbn|CBN15937.1

MACFHEETCIMAFEAETTCQLFYYTSKPTYLIVLDSSAGGIEGTGVVAIKANVPSCTATFNFSFIFIRSEIENEYYFWYKTFSGWSFQSCRYGWQRFDRNRGASIVCMKAVKSETLETAKEQCESLNSTLIGVASTQESDWMKSTVLQNTNDEAAMFWISGVRNVSNNEGSELVWTDGITTDNTTIALLSDITGESENCLAIQATETSSIIKVDCYSSVPTGAFCGYKFI*

>Cbn|CBN16409.1

MKSLIVFLILCSASTCAFNITICAKGYNLVNNKCWKLFQNAATHTDAEKSCTADGGTLLMVKNETDNKAVASYLTNTGIEHLWMGVFCIGNSRNLCYYDDQTGSTLLYDNFAAGFPNSAIGRCVYYSVPGHPAGQWLNGDCTQKLSYVCELPTTHPDVCELNFNNNCYFMLDALSFSDGQQQCEQLCANMVSVHSSEENRYITSLYSKSSYDFIRLGGMAPSSSFVTWNDGSIMDYNNMETFGSTGCLYLSLKSTVTYSRGSWYTADCTTPKHILCKRPIGAPTCRSTPFPITPPPITPPTCDSGVHVAPGWIASPRYPSAYNAACQYTLTTFGSNRIRIHFNPVSTANNNNDVVNIYDGDSTDAPRLGSLSGNNFARYYTSTGNTMFIDFVYRSGSGYTGFNATFYSIF*

>Cbn|CBN16457.1

MLLLPLALIPLLSVLTSATTPVCTNGFTLVGSKCLRLYTYPSTHQVAETTCASYGATLATPKNAVDNRAIATFVGSNPSLVWIGVYCFGSSLSKCMWDDATGSVEPYNSFASGFPLVDIGKCVYYSTQGALAGKWLSGDCDNEPRAFVCELPHTYTDICDHNYNGYCYIPIAITGFVAAQTQCERNCGNLATINSPNENRYINAFVSYDYNDGRNLMIGAIQARRSFYTWADGSIWGYNNVDPSSGYYDSCMFMSISASNVVPNGYWYSANCTSPSYFSLCKIPAGIKCNGAPPPVTVTPIPSSPSYCNSSVLIAPGVITSSHYPDNYDNNLYCVYQLTTIGSYRVQLTFTDFYTENGNDVVSVYNGDTVTSSALLGQYSGTLSTFSVKSYGNKMTVTFKTDGSVVYRGFSARFTSV*

>Cbn|CBN16652.1

MSPTLISAFLLIATVFHSSLAASCPTGTKLYNSKCYAALGMDMTRDKALAYCKKTYGSYAVLATPLTYAENNFVTEQVQLHANWHTWLEFVADGTYIVGDDGKPPVYTNFAVGEPFSVSVGYCITIGMNGYWYAQPCADSHSALCEFALYPPTVKPTTTTTVIPSGYPSCVQKYLNFVPCQSGWEYYPPTCACFRIISNTTYYNAMNTCRSIGGTLASVHNSGEAAFINRLGVQTNSYWISTASARDNIIVGLTYNSDRGIFQWDDSTTFDYAQGFAPAEPADYTSQGQIILSNPMGYATYMRMGNCAFQTCRYAACKRYVY*

>Cbn|CBN16661.1

MKLVVFTCFLLFGIVSARFDLSSSESCEDSHSHSHGSHSHEHGGGGGGGANRGCGRGWRRFNRPSGGWCIKVAQGQFSQAQAESKCQEEGGTLSGLQDTNEISYITSTALRLFPERTGSLWVGARRTPACSSAPISASCNAMNSFTWTDGSTSGTAGFQWNRKQPDNSHGQTQQCVVLLATGTQVVRDAWTWNTNQLDDVRCVNPGGSQQRIVRGYVCGKRATERRRK*

>Cbn|CBN16694.1

MTNTNVLQPWIGARRNTTTNKFYNIDGSYLTTLAWTTNEPSANGDCVTLKGAAPSGLQVTQCYQMQPAFCKQTPALCNSAVLGGPTTWSGTFQSPGYPTQYYNNLDCRYLINAPNNTYITIDFYPFVIEEWYDSVELFEESPHMTGYVAMSQMEM*

>Cbn|CBN17504.1

MKLFLLTTLLTSAYAHLACPSGFTLVLDECLKIIPNPLTHTQAEVDCTYLGGTLVNVHSAIKNRAVSEFASKSRLNKIWLGLFCFSNQNSSSCYHDDNSGTASVYNNFAPGYPLINGIYGGCVYMSTEGSLAGKWVSVECEAESMPFVCEVPSTHYDPTCTHNYGGYCYYPSTELNTTFATFPNAQSICQQEFNGNLASIHSKRENDYVISLFRGNIDAEYVLLGAQDLLPNTFSWIDGSNYAAFDYRDPLDPGTADCLGMEMGTGLWKRVGCTARIVFLCKRSISKTVTSTPKTHLVTSNPSDFSNCNTTFLMAPGVITSYGYLSTTPSTTFCTWKIVTLGAYRIRLSFVDYSSPDPVYLYDEYGREITYVVSSNRAVVSPTNIMNVTFQDRGIAGFRGFKAVAQPY*

>Cbn|CBN17513.1

MQKSTLILVLCAVSAMADCPAAYRALLNSQMKESTANGCAADWKFVNRPSGGWCMKVFTGYYESKAEAEKACQTVDATLSGLQTADEALYIQAAILAQVPESSGSLWTGIQRTKKCIGQKLTATCTNLTSFEWTDGATTGTGGMVFQVGQPDNQGLNQNCALFLASKTPTISARGTYYAATYEDVGCALTTFTPENIGRKTLGYVCGKKADK*

>Cbn|CBN17994.1

MRTWVLIAALAVICLTAEQELSQKDIIKNVLKTWDHSSEKKFFEPIRQQKYRTLDDADFNLETHHISKRSIAQPHVFAGQATRGCNLPGYTGATCEIPLCAARNPYVPNDQSSDDVAIDAVNLANCSETYIIVVDETMWDIKIELETQSPLNPTFFLQSENGNLIYPDSDRQTPSTYQATYIGMPPGQYQLGPRADTGNEFCTMLMSSRSYIKVSGGFVSGSQAERSDYPTLKFAYFDTESVVAVHAQGLEFPGQIQAIGFTGAENHISRYVPIATRYNCTYPYILERYTCRRSADTDRGYNMMQVEGISNAGYLFRRIISYQCVLPPVTTTMAPAPTTPVTPITSCQNGGQLLTDANGAAYCYCFGLYSGNDCSQMLCANGGFLPTPTSERCQCPEGFTGFHCQNIVCTDTSGFDFNAENPTITLVIRSRTQLSAVIEQATDAVTSIVDLLASEPGYLTNFIVVLFDNGKLLVNKRYDSWGAAMVDLTKAIHSAPSDGGCDDVVFSSVAAALSQYPTNKSPVYIITDANPNDSIEKETVFHLESYWRAPLYFVYVQPAPADGCNTSPDNAGYRDMMDMAARSSGNTFYFSNRTNINSFFYQHMLNTLFRSQLILSGDYSHCSNQNVYKSAAIDLTADLVVVVATGTDLTLLVTSPTGDRPTFDVSYNDGVNYIWTYNRHVSGQWFFNIISSSSSSSCTFKVYQKKFNFGGMTKYNPDYDIFWSFATTLTSAAGLLRQPVLGFDASPVFHVSNYPAFVSMDRVHANLQIYAIRDGVQTEVYGSSGMWRDACEFNFYFTPFTCRRPDELLYFNFFARDNNDMALQRAGTMFCASVHPTPPPDHQCQNGGVMNPSNTTCFCTPEWSGTYCTDIVCYNGGTPAGDHCVCSPGYAGESCELARCIETGPNPEFIRYGVDMVFAVEITQNSLASLVMLDVNFQEILRDVLMQDRGWIRNFVLVGYNSTWGGPIAESPASNLTAITNALHSLASTVPSDTGCTVKLWDALNHAIFARELPPGSFVEIFQTTPEDDTDTRSLGLFYDMSRSMDLVLYGFLAAVPRLQPSGFVCNATIENYYTLLGIVSGSTGTTYTLQAAEISNAVRLIPLQFSNGQVNFNYLDDCSHDNGMITYFPVDAYTQTIQIGTFGYGTNIQVYTGDGVLAEALELFYDDFTGQSVYEVRKACPDGWDPNGQYCMKFVSANDDILPMPQARAFCASAGGYLVDDVSEDKHNFLISAAHRTQFWIGLFRNTDDGQFYWDRGLGNDPSLLNQPQTYWGVNQPSADPVDQCVYFDGQQGDNTKVWITDSCSTPRPFACQRHRYDADHRPNEIGDDDLPAGHWYAFLKSNPSATQSPSCYMDIRVQSSLQIVTGYTTNIGSDMPQPDPIQDSPSNRLITYVHSKDNENRVPILTDAYLWDAYNGTFYNGLKYQARFACQYAWVSQDFPCPNGDNQANEFGVLHVGEDEFGNTFQRLTYGHCSRAEISCGNGGIRQNGVCVCTDYWTGSRCTVPICVNGGTRNADQATCNCTDGYTGQNCQFELCYTNSQELFSNDRKTMLLVVETTRQNSATVTQLIANLKAIVTSATSSAPLWFTNFGLVTFDTTGRTFEKFDYANIDALITDLTTQSNAISTDGACSLPYLGVLAHLLEHDDLIAKPNSEIFLVTAAGPSDLASYVETMDNLFNTQVHLHYVVSKSASCPTFDGLNNVQDMTWLGYGSSGNILFTDSDNIVNLLNNYLPTLYGASVLQDPTGNTGYDCSDGSLPWFVPVDINTTYIYVTTSAEFGSLSIKDPLGLSHNVAPIYNVQNQKLYQIEVARLGGIWTLQLVNPPGLCLAHVYSTGGAKVYTKFALPNPTGGKADPLGSHQDGMMAQPVAGFDNVAVFHIAGDPFHRGQLQYVEIFDVGQNSVTNILRSELYRRYECSYEYYSDLFTCNGDMVVVFVHGVDENNQKFRRQEVVVCNGRNPSTGLPVTGTAGPVTQPTQGPNTQPQTQGPVTTTQVPQTALQFDVVFLIDGSSTAQTSFDSFTKFIQTFMVSFNVGLAGARVGLVVVAPDLDDQAPPAAQLITITSQTILNSYLALLKDNYADFDHAGQVLTYNLQVVTSTDFMQASAGYRSNINNHVLIYLTTTTNFYTDPTPSAQTILAQKQYGIITIGYGAGFDNAKLQSISGGAACSFTATDFAGLNNQIRTIQQLIINANSNGGVYCTGN*

>Cbn|CBN18069.1

MILVFLLFALFPHATGIACQRGLTAVNDQKCLMMNKMELKHLWAENSCAQYGGTLVPIKNAVDNRAVANMAASAGATNIWIGIFCFATGNTTTCYHDDGSGTISYNNFASGNPAVQNGGCVYMSVTGQSAGQWVSGNCEVSLPFVCESPTTVADPSCKLNYNGYCYLRSHEIPSTSNITTFTKAQAICQANGGFLLSLHSKPEIDYVRNIYKNTRYSSIFLGAQKFLPNTFDWDDESNWDYDYTDPLSKTKGDCLLMTLPSFKDQTAGLWSRTNCSFKYPFMCKRKILEPTAVRSLKPVQLKETSIEKTVYHSQKNLPHRQELVDFSNCNTTLYMAPGTITSYGYPNIKPPVTYCTWTVATLGPYQLGVYFTDFSVSKSVYLYDEHGNQLGNPSGNQSPFRVLGPSNIVKITHDSSYDAVYGYHGFSATLLPF*

>Cbn|CBN18172.1

MKEWNTTESGSKYRLFEERKNWDNAERHCQGFGAHLAIIDNEAKNTFVTNLINSSETSDFAWIGMKTKTTTQTSTPFTNFDAESPIDGCAVMDAKGVWSIRSCIQLRPFICQIIKSDISI*

>Cbn|CBN18283.1

MMNFNQFFIFSLLPLVINAQCDPGAIYSPERNKCYQYHTIDEDFKTAESICENSNGHLISVHTAIDNTYFAQQAQKNLYNGLVWLGANADSPVLSNPDNWKWMDKTPFNYQNYQSGQPSVLGSSACMVFNASTGKWLTPSCQNSYPFICASDPVNTVQTTCAPQKANKCPSRYAWLEETNACYKLVSSKKNFTEANAACKQEGAELASIHSKLENDFLVEMSTTSANSSIPSGHSFVYIGLVYLQNKWQWTDGSESEHYRNWAVGEPNYLNREFWTVLMPDPCRADYNPTGTQWNNIENMEQRAFICKKPAN*

>Cbn|CBN18295.1

MLRLFFFVALCFFGSVAPNCLPGDVSFEQYCFSFNRVFGAFNDTNALCYKTVGGSLVKINNMIENNWIQKWAVVNLDADYNLFWIGASDEGHTKDWRWRDNSALNFNNWNRGEPLEDRHCGAMALSSGKWFSAVCTEKHQFLCQYPNSNYPTGASYTCPPCPSFSK*

>Cbn|CBN18469.1

MQLLLTFLFTYVSLVASTSSTPVCTNGFTLINNKCLKLFPDPVTHRVAERTCMNLGATLATVKNANDNQAITTVAGSTTSLMWLGLYCFDNDDTKCIWDDGSGVTNKYNNFANGFPLAIIGKCVYYTTQGVFAGQWLSGDCDTDLHAFVCELPFTFADTCDYNYDGNCYTFHNTLTPFVQAQQACEQECGNLVSITSEMENRYIAIITNRLWGVFGIYLGATWSMYDMFTWVDGSRWNYNRIDRSFIDRPCVALLSGPGQTPSADSWYGVSCTSARYFICKRPAGSQCNSPQPTVTVTPISSSPSYCNSSMLLAPGVITSPNFPNNYDNNVYCTYHLSTIGSYYISLQFLAFSTEADFDRVFVYDGDSTSNTLLATYSGTSAPLLVSSGNSMTVVFKTDRSNVAQGFKARFMSYAR*

>Cbn|CBN18475.1

MHLLLSLLIFLPSISYSQTAICTNGFTLVNNKCLKLFTEPATRKDASKTCGKYGGTLATIKTSQDNSDVVTIVANTKRRVWIGLYCLMNDPPTCFWDNDRVSVAKYNNFAPNFPYVEIGRCVTFKTDGKLAGKWLNVECEEDLRPFVCEIPSTFEDDCALNYNGYCYIPSEGEAIGFDNARRSCQAVCADLISLHSDNEVRFIESYYESSNVSSIQIGGRAAPSKYYKWVDQSEFDFNNYDRLAGVHGDCLFLALSTDGKLSKGSWYGDNCLESRSFMCKLKIGTQCDGTNTTTPATVDPVKLQSVNCKYQWTS*

>Cbn|CBN18529.1

MSTSKDELPTHQETAEPLLNPAAQRKRRNDFMLGFLVSCIIFLILIGVGVAAFMLFGEKNKTDQLISCTPISPVEGMTTTIPTTAATTTKASIDREMEEMGKSMEQAFSIMGMMIKGLQGLAVGDLQKPAMEHIDKDSDWVTVNRTESGRKFLHRFFPEKLTYEEAEKKCQEHGAHLSGFTGQSELDVLDKMLDEAISKGVRYDGSDNVWIGARRRDACSTREGIGAEKGGFNPDPTHPCSRSRVFEWVNGVAQNPPDFQNLWITDFEPSFAGDSESCVVLMKGPKNIPGWPRNVAGDKKLNDVPCELEYYFFCGKEIVKTDE*

>Cbn|CBN18842.1

MFKPEGPKCLDVVGHFMVDYDDSHGGEDDENDFFNVENDDTPEAETPVVNAPAASNPVVVSKVETADVACRKMEVGNCEEGWKSFKRPNGEWCMKVFYENWVNQIESEKRCNEQNATLSGFHDQLEKQFVTTTVTDHLYPSTGSVWIGLKRTPNCLNSKLTKNCTTLNSFEWSDKFTKGSDGMLWTWGQPANVGGKQDCAMLTTGYNGYIQYFQTGTFDDVGCDIRYKDTTRGVRGFVCGKQAKH*

>Cbn|CBN19186.1

MACFYEETCIMAFEAETTCQLFYYTSKPTYLIVLDSSADGIEETGVVAIKSNVSSCSTTFNFSFIFIPSEIENENYFWYKTFTGWAFQSCRFGWQRFDRNNGASIVCMKTVVSGTLEAAKQQCESLNSTLIGVASTQENDWMRNTVLQITNDETAMFWISGLRNVSKDEGSELIWTDGITTDNTTIALLNDITGESENCLAIQATETSSIINMNCGSGAPTGAFCGYKFI*

>Cbn|CBN19332.1

MKSISTLLLAVVIGSVVSHRDNYGFGGGRPGSGGRPGSGGRPGGSRPGNSNRGCGSGWERFNRPSGGWCIKVFRGEHSQANAEARCQNIGARLSGVQNQAEINYITRSALSLISQSSGSVWLGAKRTSACSTAPLSNTCNSMNSFQWTDRATTGTAGLIWNTNQPDNAHAQTQQCMVALASRSAMVQDSWGWQANRLDDVGCGVVAGENGPRTVRAYACGKRA*

>Cbn|CBN19745.1

MLKNLLILSWLSLPILGQNYICPPNYTMVLNKCLQVFPTTAWGNHSAAEQICRKDGGTLVTIRSAIENRAVATFAAGNNRIWIGTFCFGNDTSQCVYDDYSGPLSGFSGFERGYPLVTNHNDGCVLMVTQGSTAGNWLSSWCNPYEPVSPVCEVPRFVDYQIPTPSCVNKYDGFCYFLWQGTYQDGTSCGSSFLSIHSKRENDYIQNNLFPPGQNQILIGTKRQYLVYYAWTDTTNWDFDNRDPMDFNLNRLDCLAINRSTGLWNSVACNVSLPLVCKYPIPDNHPIESHCNANSLLMAPLTITSNGYPHNNNGSSCSYKIGTTGAYRVKISIVDIWGVQLTIRDFDGNLIAQTGLQGTYVSPSSYISVTQTGTGGFKAMVIPY*

>Cbn|CBN19898.1

MIFLILSFFTSVVVGTTPAPPTCPDGWTVLNDDTCVRDNRILTNLAISNGQTHPVWLGLTCPGSSWGTCAWDDQSGYTSDYNCFASGSPSSDIGPNAYILTTGNSIGRWVSADGDILSLAYFCEIPTNNQPAFCPNEFNGSCYTMHISLANETIARETCHQECGELASIHSQDENDHIFALYPRTTNQYQYRIGGRTTPDGTGRYWVDGTDFDYQNFDYFNEFNGQCVSVALVFDIIDAEGWMSNPCDEEIPFVCKRPKSAVSCGAVPPTVSPKVVTVPTVAPPPTVTPYPEMCRHTMFDVGNGTVFSPYYPESFNPIMSWDCKYVFTTPPGTKAQMRFSPDSNLIDTPLFYQDALEYCKAYPGGNLVSIHNAIDNRALVTIANDQGYTKPVWLGMTCMDWEYVTCVWEDQTGYSTSYSSFASGYPSLDIGVNSYMITTGNSIGKWVSADGTVQTLSFFCEVPTNGAKMFCPNEVNSFCYSFIAPSPFLPFQQARDYCTSQCGDLASLHSDYENFFIKNLVPYTFYQKNQFWIGAQTDGNALYWTDNSQFDYNNFGFLNPDIGNCSSLSIYTDTVASGQWVSSSCDQKLPFVCKRPKNVTSCTGISPTVIPNGVPLPTVAPRPTVSPNDPEQCHRGAQFFSGSGTIYSPFYPNNYNSLDGGPCVYVITVPHGQIAQVQFTQDTFIKNSWIEFYAQLEDGKQFGNFSGNAPVVPFYSSTNVIKMVFHLFWEYRDANEHWVANYGVKV*

>Cbn|CBN19996.1

MPDNSCPELFTDVDMKVPNTDVAWKKSGRRWTLMKCMDGWELFQRPGDIFVCMKFFQTEFGANRSTAQNTCTKNEAKLSGVANRKKSEWMEKQAEYLNTGAWDGAWLDGVRSCDASEPNCHNYIWSDGFTSGNDAFGGNLWSPNHTATVQSCLTALSILSGYQRLNAVSCEKYDRAVGVICGYQLK*

>Cbn|CBN20166.1

MRHSVLVGVLLSLATVETVSVAKSTDSDIVLKVSTDKQSTRDGHHFSGEWLESPWGDLYQFRAGDQNWLTAREHCLSLNADLAAIRNVEQLDWILSHYAPLSSRFAQRLVQIGLYAPEGQTHEWKWLNGEEINKTLQWSSGEPYDHSMEGRERCGLLNVEKRLLDDVDCESTIPDHRAQRYICQRTSENHKQQQRSNNYIWQKIENLFSFFGIGGSPTPHNATAPATNDYEDEVLKNETSTAKTVAKFSDSNEETSSEEEESVAKTLAALPKIEGSGESTSLKELQEPEGSGQIVDKKAIEAADLIPGVDEKKLDKMIDKMEEMIKSIDDLTVQPSSLERTTVSTVVLKKEKKDEEVTEEKKVEEKVKEKVKEKDQELNDNKISESIEGDFDHAQSKDMPKADIEPPKEEEEDVDCDEEGSGSGEEEQNEEETTEKLELAPEKEDKIKEFLGVLRLFLDRAEHGDLRKLLDDQSGKTLLERMKNAVREANKREFEMLEKLESSKKTGDKDELVKIMKKDQMSTEEQKDLYKKISSAVMKAAKLHKIEEKEEKSEEKVQEEQAMEKFAIAKEKADSEESEGTLEVLKSAREGKAEKKEKVGDDDYYGDYLDDNNVIKVQNREKSDVVEGKKEEKKETKKEEKKEEEKVAEKADEVKKETAAHDEPKKEEDAKKEESNEVKEEVEKDSKEEKEKTGETTTIAKVESDEAENDDKLKEKKDDESTTVASESENSSESTNSTTEKSETVVSIKPAEAEENDAELEASGQEEISTTTESTTVALKQIPAEEIEKIAKLEAKQLTEDEPVTEETKVETTTTPTSSSTTEINKESTSSSSATTTTTQATSSSKTTTTEAVTTTKAVTVPVKTVSPEEMEKLSKMEATEKVTLLPPLPTFTFPTLAPFTFPTLPTHATAKPTPAPKVPTLEEILGNLNDQFKKLLSPPKPLPPK*

>Cbn|CBN20395.1

MRFSIIVASALTALLLVHAAPTPSNNDIESSGEAPVTLPQNSEEEPHRRLRFYNWDFKDLGTTAFEDIPYPARQPPSAVNQTEKCADGWLRFADSCYFIESEPMGFAKAERNCFQKQSTLFVANSIEEWDAISSHAKEAYFSWIGLVRFTHYEKTEQLPRWQTEGAINPAKINWLIKPFKPVSNGWTQFANCAASYKSPAKLESASYTFFYPCTYLLYSICERNSTIVNVMQ*

>Cbn|CBN20633.1

MKLIVFTCLFLLGLVAARFDSSSSESCEDSHSHSHGSHSHEHGGGGGRGANRGCERGWRRFNRPSGGWCIKVAQGQFSQAQAESKCQVEGGTLSGLQDTNEISYITSAALRLFPERTGSLWVGAKRTPACSSSPISASCNAMNSFTWTDGSTSGTAGFQWNKNQPDNAHSKTQQCVVLLASSTQVVTDSWTWNTNQLDDVRCVNPGGSQQRIVRGYVCGKRATERRRK*

>Cbn|CBN21163.1

MAPRVVFLTILALVGGAAAQTCNTGGIYSAQFNRCYQYFTAPAQFEFAEEQCALLGGHLVSIQNGAENALIQSNGANSFKKSNYSDYWIGANDLETSGQWKWTDPSVTWNYQNWALGEPQSGSDCAIQDKGDGTWSAIGCTSYRPFVCVTPVIITATCPPITTPKPTVCPTPTPCPVKNCVPSCDQGWTYFAPTDFCYRVYHNAKWDDAEAACVLLGAHLASVHSETENTFVNNLASCGIKEGNPKDLAWIGLHKVGQDWVWTDGSKSDYINWAPKQPDNPGKENCVETAPDLSHDKWYENWNNEACSTEMRAYVCKKGSIHV*

>Cbn|CBN21227.1

MKSLVLLLLISSIAYGYDNTICKNGFNLVNNKCWKLFQDPANHTTAENTCTQYGGSLFMAKNAIDNRAILSFVGGYQIDSLWMGIFCIGSDKSQCYWDDQTGTTVIYDNFATGFPNSGIGRCVYYSLSGSPRGQWLNEDCTEKMPYICELPTTHTDVCDLNFNDNCYFRLDAQPFNVAQKQCEQMCGDMVSIHSAEENRFIASIYQDLPYDYIRIGGVATFSDFIVWTDGTTMDYNNLERIGVNGICLYMALKNTYNSTRGAWYAFDCSTPYNYVCKRPVGEPNCRSTPAPPPPPQPIATATCTSGVHVAPGVISSPGYPHYYTSGCQYTLTTFGPNKIRLFFNYCNISPNDYIYMYDGESAYAPLIPGKGTWTFTSTGNTMFINFKYGSNKPAPGFNATFYSVF*

>Cbn|CBN21611.1

MVLAIIALVVSAFFIPDVLADPCGDSNWRYFPQTNSCYKLIDENLPWTIAEFKCLFQGAHHVSIDSPEENQFVHELSNWSEIWTGAAFFGKDMHYVNSDGSRYGNFENWKDGRKPPMNRARRCIKMDQNGEWFQSCCKKKTYTICEKKAAYSASSYSGSTNTVNGFRFMRHRS*

>Cbn|CBN21668.1

MVLQMLLYTWSFFFIYSILPAIISANECYVTPCTRDIIIVVDGSSSMQTSTYVSQEINMITKLTYSWTLDESKVRLALVGAYFGNEFNGLDYFTDSSLVEKRLQSFRLAAMQYGLFSGNFNTTVRFLDERYVGKRANFGPRINVQKRIVIFSSHSGTSDISSTKNTLEQFSQQGYEVTIVGIGVDESVYKGTYYHKFVSVQWFELGVVAQSIIDTITEEGICFLDKGWTTPKPQTCTTTTTTTTTRAPTTTTAKGVTAKTVTSKPTTSPKPVPPTHPPFPVGDYQECSCTTQSLYIDIVFVVDTSAGMGLGGLMMVKAEINTLTVFKPSDYDNEDEFTEDLWTDPRLEDVDETIDEVNLHAGLQQAAKMLGSMRNGVKKVVVVYAASYNDEGNDDPRQIAANIRGSGHKIITVAFVEPESSSLVLKMAEIASPRMNFTSYRDDLLVEELEDAFCQVRKKEFILFLFHKFQVNCYCPRGWRQLTLENRTYGECFFPTKIDASWTATKYECPILSKEHTGNGHLVYVNSALKNTFLNNFYMENWDKENQEKPNYDIGYYYDKTTQKYIWVNGVTNNPYANWAENHPDLSKGECVMAKQVEGTTTDFKWISINCQVDSGRGLCQEAACDSDFYCPPEN*

>Cbn|CBN21835.1

MFKLVTLALAYLAFASAQTGYTDWFDSTGTVVPTPYPTPGGTNVDRECGGDLSNLWLDVVVVVDNSKGMTNEGITEIAANIVTVFGNGTKIGNQYSDPRSTRLGIVTYNKQATIVADLNLLQSIDDLYQTIFSVLNTVSNTDDSFLAKGIGAAESVLQNGRANGVRSNYKRLVVVYASAYKGEGENDPIPVSDRLKSSGVILSTVAFDQDGDESLLAGLAVIASPNYAFTSKDLNLVGELQGVALQSEFSSKYKPPNCFIPANCFCPNLWTQYKANFDDEYSYKYGVCIRAATISSSWTAAKFACQNLAKNGFLATEYDGQKHNFLFRVAQNNTAFTAPYIYHIGLSYVNGGWNWQQPAGYPLKPMSGYSTWNPSYPKSFSSNIGVLEQQFSSDLTVGWQNINAYSVAEYYMCEVASCDTEKYCS*

>Cbn|CBN21895.1

MFFLPRSFSLPSFLIFLNFLVIFAYSKKTNGHCPKDWKNVTRPSGEWCMKIIYENYLIHSEAEKRCQEEGATLSGFQNQMESMWVTTTVASKIYPNTGSVWVGYRRIKDCLKSGVTANCTRLNSFEWTDKVTTGSHGVVWTCGQPSNYAYAQECVTLTVGYTGYTEDGYQVGTLDDAYCGTRFKGTPRDVRAIVCGKTPEC*

>Cbn|CBN22240.1

MRQSSVFILLLCVSSALATVEVATKCPDGWKWFKRSRGGWCMKVFAEMITLEDAQAKCKTEGAVVAGTQNLDELNWMADTVQTLQPSSVSIWIGATRTNACKDVRITAECTALSSFYWTDGSVVGVSGFKWQPGEPNNTGLGQACVQLYIDKKLMDDVVCSGHKLGGYACGKIASF*

>Cbn|CBN22241.1

MILNKCLQAFPAPTKGTHSAAEELCRNDGGTLVNIRSVIDNRATSVFASNHNLDKFWIGTFCFGNDTSKCVYDDYSGSVSDYNSFAAGMPLVTGISDGCVYMLATGSQAGRWISSKCKTDESIGSVCEVPAYMYDPEYSNKNQCSYFDGYCYIKQGGEISTMVRTCSQIGGFPLSIRSKRENDFVYGLNSNNYEDLLLGATRIAYNREESVSKYAWMDGAGWNEFENIDISADNSDNDARACLAMDRWTGLWKSVYCGTVYNYYCKVPLPPNPAVDISNCNSTVLMAPAVITSFGYPDNSSPPPCTWKIATVGAYKLRIAFKNGFSSGVTVYDGDGNNIGTAIYKSIVAPTNYITVVQTGRGQFYADILPY*

>Cbn|CBN22337.1

MFPLFLSVLFGVLLTCDGLSTTEAPYSCQQGYTLLNNDVCVKLFESPLRLDDAIRQCGNEGLFYYGYGKLVSIHTKDELDTLLSIAKAQNVVSPVWIGLTCPSTQAMDCLWVDRSSVYYHPFSYGNPKPENGDNVYMLTSGSSAGQWVSVNGAVASFGYFCAIHSITSKTFCPNQWNDYCYTYHNESLNEADSRSVCQKECGDLVSVHSDAENNHVSLVVPYDPLLTEIRLGGRVDGNQKYWVDGSEFSFSKFGYLDTNIGNCFGLQVKNDLVYRDWWLSNNCADEMPFVCKRLINATSCPYVPSTTPNSPYVNFDCGVVKHLTGTGTLYAPPYLYSGEYGPCVYVISQPKGSIAKVQFLPNSEIKNSRFTLYSQLEGGQPFANLTGTLPLTVFSSPTNVLKMIYTGEPTYPTQLLNYSANYWST*

>Cbn|CBN22358.1

MKLLVFLSLVVYTVSDTPVCNNGFSLVNSAKCLKLVTTPTKHRTAEANCASYGGTLVTIRNAIDNRAVSTFVGNIGLSFWIGVYCVTNDPTTCYFDDDLGTASAYNNFARGFPNTDLGGCVYSASSGSLAGQWISSECEDVEMAYVCEAPTTKTDSCAHNYNGYCYLTSHENATSPALPFTAAQDACRQNCGELVSIHSRRENNYVQNLYTNTNISAFLIGALTTAPLTPYWIDQSRWDYGHVNPRSGSTGSCFQMTTGTDGTWYQVDCKASQYFLCKRPAGTVCNSTPPPPVIVTPAPTNPSGCNSTSLFSSGTFTSPNYPSAYGVTWCVYKLTTLGAYRISLYFTDFYVYQYTFVNVYDSNGARLAQLTGTSLPSPYYSSSNTMTVSFSSTPGTPGYRGFNAKFLSF*

>Cbn|CBN22634.1

MEKIQSKLRWMVYESELNPLLFSSEYSSKVFSGTNIEYTDAASACANNNAVLSGIQDSNEMRYLARTASSNLASSTGSLWIGAKRTAACAHSKLTAICSKTTSFYWTDGATTGTAGFSWLDGSQPDNALGGNQDCIAFFFAPSNTRIATVNWFPGAMDDVNCDASQFNSIPQRKIQGYVCGKDASS*

>Cbn|CBN22819.1

MLILELSFFALLDCYFLLITLWTQAKVMPKTSFSLLLFSSFVIICSAQVQNGTCEPGWTWFSRPNGGWCMKVFYGSHTWWHAAGRCQSHGARLSGISGEVVFPLPADIERNFIKQSILNLTAGMNSDIQPNVWLGARRSALCMYKQYFVGGPPCLPQFAFQWTDGFTTGVNGFGFGTGEPNNIGGLEDCAAYRPVGGANDFNCDDETATSGYVCGKEDIDP*

>Cbn|CBN23042.1

MKTLIVFLVLCLALTHASNNTICAKGYNLVNNKCWKLFQDAASHANAERSCSADGGTLFMAKNAIDNRAVASFVSSSGVDHLWMGVFCIGNSRNVCYYDDQTGSTLLYDNFAAGFPNSATGRCVYYSVPGYPTGQWLNGDCLQKLSYVCELPTTHPDVCELNFNDNCYFTLDAFPFSDGQQQCEQLCANMVSVHSSEENRYITSLYSTSSYDFIRLGGMAPSSNFVTWNDGSIMDYNNMERFDSTGCLYMSLKNDYYHSTGSWYTADCTTPKHILCKRPIGAPNCRSTPVPITPPPITPPTCNSGVHVAPGWIATPRYPSAYNAACQYTLTTFGSNRIRIYFFYGVYTPNSNDQVNIYDGDSTDAPRLATLYGKVGDQLYTSTGNTMFIDFVYRSGSTSTYSGFNATFFSIF*

>Cbn|CBN23248.1

MQLFIALIATCVSLVFTSNTPVCTDEFRLVSGKCLRLFTSEVIHSVAERTCMNYGATLVTIKNAVDNRGIQTVAKNFNNTVWMGLYCFGDDVTKCLWDDASGDSSLYSSFMAGNPQVQTGKCVYYSNVNESSTLGKWYSSDCENTARPFICELPSTYEDDCGNNYNGFCYTHNPAATFTVAQATCEASCGNLVSIHSANENRYLNSLFLQSGSGIFYIGAIRTSRNSYSWFDGSLWSYNNFDVTALRPAGVQCPANPPPVIVTPVPSNPSYCNSALLMAPGTITSPNFLNNYDNYLNCTYQLATLGSYNILLKFTSFYTEGCCDIVRVYDDDNVNSPLLGTYSGSLGSFSVVSTGNSMLITFKTDGSITFYGFSASFSSYVFHT*

>Cbn|CBN23527.1

MRIKIFIAFTFLILTTVAQDFDWSFKKMCAFWGGKDTFKPREGGSKAMAGDKCSFTFPMATDTELSARTYCETYVPYHIISVVPGPNTVCNAEATLICASGWVQMFGRCYKMTKELMTHEQAEEHCAKQEQKSTIAFMHREALPFRIYDYFTGVYSLWLEASIAITQDLIYDVAGGHLLLAIDGYPYNLPNVALARVAPTEKAMVLCEYTPRMNQAESNYLLKRYGEIYYPTIFTSDSAYVRTASVLQRNDKDPSMDHEYCKHVLRPFLQTDDAQAAVPTREFLDALKKERVAEIIRTSVYSGDSKVASRSNPACSVSDSRNYGMEFPGDGKNFFKTMKDEPIWSEDQPKEVCDGASWSTGIVLSRSSPAGLEVMSDARAAPIYCQTTFDVMEYGDCPENYKPFYRKETGQKFCHDFIDKPRSYDEAEADCAGDGAHISGFTGQEELDFLDSLLDAAQEEDDVPFGHENSIWLGAKRREACNRKGIQGKEGGFNPEPTHPCSRLRVFEWVHGVAQNPPDFENRWIGPLEPNFVSDDEKCVELLKGEQTVWDDKNGDKKLNDIPCNDRRLYYFCGKEAPIVSKKNIL*

>Cbn|CBN23542.1

MKLLLLAGILFTISFYAYADIICAPGYTLVNESKCWQLFNNQTAESDADESCRQNGGGILATLTTAIDNRALLTMFNGTDVSRVWIGLKCTGLSLSTCQWDDQSQVKYSSFASGFPNDRFGQCVYYSADGSPQGQWANGPCEDKLPYVCELPTTSSDFLNCTNNYNNNCYTADNTTIEFSWAQKDCQTQLGNLASVHSYLENRYLTSLFDFDGSVWLGGLAPGAGLIVWTDGSNNNYYNLRTTGNGTCVSMDLHTDSSNGNWTSVDCNSQLGYLCKRYTCSSDGHCP*

>Cbn|CBN23669.1

MKHFVSILLLIALTGSAIGITCPRGFSAVNNQKCLRLFNNNMKHLEAENDCSYLGGTLVTIKTAIDNRAVTNMAASASAQNVWIGVYCFAVGNTTTCYHDDNSGVLNYTSFAAGNPAVQANGGCVYMATTGKNAGQWFSAPCEVIGMPFVCEVPPTFADKNCTRNYYGYCYMPSHEIKSSSTNTTSYDEAESICEANGGTLASIHSKPELDYIRAIYRNTNIQKIFLGAQAFLPDTFDWQDGTNWDYDYTNPLATTSGDCLQMDLTAGKPNNGMWSETDCGSNLYFLCKRRITPTPGVMAQEVKKQGRVNPKFRKSRKALPHRQELLDFSNCNSTLYMSPGVITSFGYPNSKPPVAYCTWTVAALGPYRVGIYFTDFSVYQDVYIYDEFGNVVSAPSGTQRPFQVLGDTNLLRLVHDSRYDANYGYTGFSATVLPF*

>Cbn|CBN24093.1

MNKFLLISIFSLLFSGVASVACPRGFSLVKSNKCLKLVTSKLKHLEAELECSYLGGTLATVKTAIDNRAITNLAASASATTVWLGMFCYTNGTTIPCYHDDDSGPVIYNNFASGNPKIQGAGGCVYMTTTGKTAGQWTSSICENVGMPYICEVPTTVADSTCTRNYNGYCYLPSHELKLPLSTYPNAQAACKANGGNLASIHSKTEIDYIRAMYKGTGISDLFIGAQSVKANTFKWEDGSAWDFDYRDPLSTTLGTCLLMDMYPNIINNGLWGATDCQNAFNFLCKRKIVAKSVEENEERYMDHPKNLAIPHELLDTSNCNTTLFMAPGVITSYGYPSTKPPKTSCTWTIVTLGPYRVGLYFTKFSTGSAVEIYDEYGGIINSPSGDQSPFYNLAPTNWVKIVHNSKSDTGHAGFSTTILPY*

>Cbn|CBN24189.1

MKSISTLLLAIIIGSVVSHRDNYGFGGGRPGSGGRPGSGRPGNSNRGCDSGWERFNRPSGGWCIKVFRGEHSQANAEARCQNIGARLSGVQNQAEINYITRSALSLISQSSGSVWLGAKRTSACSTAPLSNTCNSMNSFQWTDRATTGTAGLIWNTNQPDNAHAQTQQCMVALASRSAMVQDSWGWQANRLDDVGCGVVAGENGPRTVRAYACGKRA*

>Cbn|CBN24248.1

MSSVLLRFKLFNWSYKDFGTSAFTDISLDQRQRFADFPTGLCPDGWVRFSDSCYWIEQHKQSFAEAEKRCYEKNATLFVVNSQDEWDAVREHFPQVGFTWIGLVRFTHRERSEDFPTWQTEGAVNPTRLNWLIRPYKPVSNGWSILANCAAHYSAALNWEASAYTYYQPCSFKHFSICERNSTILDFLNRKFDIQS*

>Cbn|CBN24249.1

MKLILLALLCFITYAYGSTKICAPGYTMIDGHKCWKLMNGAKNRQDAHNDCGLNQHGALLAMTESSDDKEALKKFVASTGQSNFWFGLKCSSDDKSNCKWDNGDHLEYDNFNSGQPSGNFGCVNFLSATGRWGSRDCGHTLPYVCELPTTTKAPQQNIPDCGNNCDTNFQNECYKLVHSSKNFNDAESQCKSFGMHLVSVHNALESGFVAHMYADKGTYWLGGKMPSDNQINWLDGSSVDYKAKMRVLDGQCMQISVNDNHSTDMWQGKNCGYTPSKFLCKRSVQAC*

>Cbn|CBN24453.1

MKTDPTTTPTTTVPTTTTTVVDTTTTVKRYITFRRTSVGARLTDCAAACPDGWKYWNSKCYKKFNEWGTYYDATLSCSTIGAQLVSISSESENGALWRAFDSNVLVDESEVSWIGLKYVSGNWMNTDGYISGYFNWAPTQPAMGSGQCVQMITDSLKNATYLWQRGGWKTFDCTSVSASYICEMDANI*

>Cbn|CBN24497.1

MISKAVAVFLIFLVKFGVSDDPCPLNATLLHDNSCVKLFESPLRYQNASDVCKSLNGSLVSIHDSINDNALLLLAQSRYVVSPIWIGLSCIGIHREDCNWDDGTGSSAEYFHFASGNPSLQAGNNVYMLTSSYRAGQWVSVDGRLASFGYFCQFPPIVPLKFCPNLFNGYCYTLHTDELNEADARKVCQNDCGDLASIHSHEENVHINSLSPYGANAIDTARLGGRADQKVKTWFDGTPFNYTHFGHLESSRTCFAMNMVTNANLDAGQWESFDCDAKVPFVCKRAMSSTQC*

>Cbn|CBN24657.1

MSNTSILLFFFFLVTVSANVCDDLREKEEEINGCGKGWKRFQRPSGGWCIKVFFEELLTQAEAEKRCQAEKATLTGFQNQTEVFHVTTTATTHLKPATGSLWVGLKRNQKCLKLKITKNCTTMNTFDWTDKFTTGTHGFIWNGGEPLNYGWNQDCVVLTVGNTGYVETLFQVGTFDDVGCEMTYKNSARAIKGFVCGKKSEK*

>Cbn|CBN24871.1

MSFGYILLVLCAILFFGNAVNGHPKIKSCPKGWLQFEKTCYFRQPDILNFEDASKSCERRESKLFDYDDAFEFEAIRKLFPNYYYTWIRANVEEELEWLYEPYEEIINGKNSVATCIAFYSSPTRSYSYYHPCSALFHSICKKSLDTFHQWMD*

>Cbn|CBN25294.1

MKSFISLFLLFVLFSNVAAMVCPRGFSVVNNQKCLRVFSNKLKHLEAEIDCSYLGGTLVTVKTAIDNRGVANLASMSGATQVWIGMFCYPTGNTTTCYHDDNSGPLTYTSFAAGNPLYEGNGGCVYMSTSGKTSGQWFSAPCEVIGMPFVCEVPPTVYDATCTRNYNGYCYMPSHEMDIDTANTDYAKAQATCQDNGANLVSIHSKPEIDYIRAIYQSSSITRVYLGAQAFLPDTFDWQDGTQYDYDYTDPLATTKGDCLMMDLSKKDNNGMWSEISCTNIEYFLCKKKLVEPTAAPVVTTAGTMNTSPAAGDVEQKEVEKVNPKFHRVRSALPHRQELLDFSNCNTTIYMAPGVITSFGYPNTKPPATYCTWNVATLGPYRVGIYFTDFSVYNAVNVYDEYGNLITTAAYNKNPFQVLGTSNIVKITHDSRYDAIYSYTGFSATILPY*

>Cbn|CBN25378.1

MRLKILFFAIFLFVVNAQDFDWSFHKMCEYYKGDYKARTGGSKSMVGDVCKFKFPYATDNKESARTYCEEYVPYHIQVASFGPETTCLAEATLICASGWVQFFGRCYKMTKELMTHEQAVNHCATQDKKSTIAFMHREALPFRIYDYFTGVYSLWLEASIAITKDLIYDVAGGHLLLAIDGYPYNLPNIALARVAPTVKAMVLCEYTPRMNQAESNYLLKRYGEIYYPTIFTSDSAFVRTASDLQRNDGKPLRDHEYCKHVLRPFLQTDDAQAAVPTREFLDALKKERVAEIIRTSVYSGDSKVSSRSNEACSASDSRNYGMSFPTSGGSPLFKSMKDDKIWRKDEPKEVCDGASWSTGIVLSRSSPELGLEAMSDARAAPLYCQTSFDVFEYGRCDSDWNTAYRKETGQKYCHRFFSEKVTYDEAEKKCQEHGAHVSGFTGQSELDILDKMLDEAKRKGVTFGNDETVWIGARRRPTCTRLGIEGKEGGFNKDPTHPCSRRRVFEWVHGVAQNPPDFENRWVADLEPNFVGDDEKCVELLKGVKNRWNWPKDPADKKLNDVPCDLKLYYFCGREAPIVKIDA*

>Cbn|CBN25828.1

MSQKDAEAKCKTEGATLSGVQNKEEMKWMGDELIAISGKNGRIWIGLHRTDQCMSGGLTATCTALSSFYWTDGSTVGVSGFKWGAGEPNNMNGQACGIVWAQAQTMDDMGCDQTVTEALVCGKIATF*

>Cbn|CBN25876.1

MLEGKFEKKFYEMKMKTEYDIEKEVGKLRDQFNTDLRDYERITTKDIIQIKREESWYTASEKCIGYGAHLASIHSRLENGFVSKLIPANETVWIGVNDIQKENVFKNPDGTAVDFLRWAQHQPNNEEHNENCVEVDHNGYWHDKLCIITRPFVCKKKIK*

>Cbn|CBN26332.1

INGKCIAVTTGRYSGSSQQCKSISCNPKQYVFTFLSIDSKAENDHVMDGLSTNNVAAAYIGLAQSSSGWGWQNGDTSIYNNWAKPPVTQPPTIGPTTTPFRSTLPPSEQRRSTAVQVDFITSLSHYIADRGPAEFAFFAYGCTQTSPYIYQYPNFVSTFEDTDKMIETLKTTINENCVRSSPLDFRSMFRDQTVYYNRYSTSYRPFKSKFLSMIYFTSSTDADNIKAASALYPMANSSVITINVGTTSIDVSRLSIPIGKYGLRVNTPEDITNLVPKVDSMIFGATGVSSAGVKVDVMKEDQWDFDVRKSEPSVFASSDYTDCAYMNTTDGLWYSDGTCATKRQVLCQYVLPLPPQPPTPNPTIAWQDPCYNEYTNFTYFEILSNAKCYRMSSSKKQFAEAENVCLSDHPLFLHVPKLVSIETREEEEQLKTLITGIPNEGMFWFGLKRDPTNSSNWYFINGDTYDASYSNWRDGYPRDADGCDCVALVVNDSSWINTDCNKPLYSICSYRFNGTEPTVKPF*

>Cbn|CBN28123.1

MWFHSTFILALLVYVSADTSDTCPKGFSILGSTNKCVKLITDVARHADASANCSSYGGHLISVHTAIDNRAYVNLASSSSTPFWLGLKCSASGVPASCLWDDQSGNAGSYNAFANGYPVVEVGNCVYVSTTGSFAGKWLSGDCNTMSLNFICEAPANTPVTDTCSFQYNGNCYYPSLSSLSEQEAEFACEQECANLVSIHSIEENNYVQSLFTNNAPTYIRIGATSNNQNVKSWIDGSNWDYSNIGYENSNLGLCWSMALLNDIVSTGKWISSKCDTSVPFVCKRKVGSQCGSTAGPTLAPGQCNGPQFYDNSGTFYSPSWPYSYIGQQNPCSYVLDTPVGSLAQIQFPIMVLDSQASIALYSRIEDSTPLVVLQGNSASNQWYSSTTNTMKVVFRPCVSNCAPVNGVVYRWQANFQPSNQVTQAPPVTITPNPNNPSGCNATILAAPGYITSPNYPNLYPNFSECLYHLSTNGGYRIRVDFGQIDTEQCCDNIVIRDGPLLSSPVLGSISGSWPAHSKIYQSSTNSMLVTFTSDASGQATGFSATFTAY*

>Cbn|CBN28656.1

MKIQILIILLPVVTSAQLDLNSIRPCNVGCQDGWVPYSGNCYKKMFNVLTQSTAEQECLNLGSHLASFETTEEATAIRSLVLIAPLFSTNLLSYSSTSQDSWIGLSKTSNGAWKWTDSSEVEFTNLPDGTSVTGASCVSMNISGVWQPNECSSTVSSFICKRASASSA*

>Cbn|CBN28738.1

MIAVVLTALLLASASQIVTADDNNHCPSGWTFSTNTSFCYIQSPQYLTYAEADPFCQSIGGSQVFVFTARELTWLTDFTKASFALPWLATTRNVTSGLWYNSDKTTPTSSWWTTGEPSANGDCATFKGTGQSGLKATPCYSIQPALCRQMPALCPTTTNYGGTSTRSGTIQSPGYPVQYYNNLNCLYSITSPVNTYITLLFSPYLLEDYFDYIDVFDGPNSSYPYLGTTDDWWYTRDSFESSSNSVSFIFHTDSIITDKGWLLTWNAKSNTPPISQSGQNGSFTSPNYPNNYDPYTEQLYYITAPIGFQVNVTIPDFNTEMNYDVLEVYNSSYVSSYNLVANLTGSAVAPWNWLSPSRYVTMRFKSDPIVQKKGFYVVWTIQ*

>Cbn|CBN28838.1

MVNVDNAIARQFLFSNFVSRNQESLKHSDGSDERFLFKLMNQNSLTLPEPVNFIGVQDFDQSSPTIISDAIGTAKCASGWVRWEGNGCCYKEMGSPMLSWYASEDWCWSQRAGAHLASVHSQAEATWLNYQYKLWWSKMDDWIGLRRNCDNTAWEWTDGSPVDFLWWQPNYPIYGGIEDSCTAVCYFLIFILFIPS

>Cbn|CBN29294.1

MKFYSFILFTTTVITIFGQLEEDWGLKKACEKTGGKYTKRVSRQSSTGDTCEMPFKVATRDEQDSRDFCELYAPWRLLKTERKKDAGRHTTICHVEATLMCEQDWWQMFGNCFRMPDKHMVFTRGEAENLCKTSAPTGTEGTIAFMHHKYIVGVWRRYFRGTPQIWVSATESWNEYIQKTKTVDGDALALAFTGKHYDFSVTANSLIRIDSSIRLNTLCQYKPPITPAEINYLGRRYSQIYYPSIPVDNGIMVRSASSYTRTAKNWDVCKKVLAPFMIEKIGDFVPDPSVLQSMRYARVERSFTYLTRSGAVRELDDNGILQRTSNGTCSSYLPEYKIKVENEKYADFLVKDIPIDQTQDCDNMLSAAINHKEKTNLQIMSDSRSLPIWCKLGRPERFQHKPPPPEYETFRRTDGKWVAHKLHKDPKTYDEAKAICESEGARLTGIDARKEADQLRDIAEKAGVKDNQLWIGGRRKPECVDQEKWNTDVNDKCYRSRVIYWEHATAREFEESWWKDGKTPETRNPDYSKKEQDCLSLVVGNPSWADPKSGGFLDDIPCKDAYGFFCSMPLEMTKTLVED*

>Cbn|CBN30028.1

DNRAIASFVSTHGIDHLWMGVFCIGNGRNLCYYDDQSGSTLLYDNFAAGFPSSASGRCVYYSVPGHPAGQWLNGYCNQKLSYVCELPTTHPDICDLNFNDNCYFTIDAFSFNDGQQQCEQLCANMVSIHSSEENRYITSLYPKTSYDFILIGGMAVNGFVMWNDGTVMDYSNLQTSDSTGCLCMSVNNYTRGSWYLADCSTPKHILCKRPIGVPNCRGTPVPITSPPVTPPTCNSGVHVAPGWITSPRYPSNYNAGCQYTLTTYGSNRIHVYFPIAVLEGGYDYVYIYDGDSKDAPRLDYWTGTNQAKGYLSTGNTMFIDFIYRSGSTKNQGFNATFISIF*

>Cbn|CBN30206.1

MKTDPTTIATTTIPTTTKSVVFSIVDPGALLIDCPDACATGWQYYNSKCYKKFDTAGTYAQAISACQAQGAELVTIDDFDENDALRKAFDTNALVTEAQETWIGLKATSGVYAWSDGSSASYTNWAQNQPAAGSGQCVQMITDALSNKTYQYQRGGWKTYGCGKTSASYICEAPAGGPSISLVGR*

>Cbn|CBN30616.1

MTKVLVFFLLGFFITEIRAQCGIGSIVDEHNNKCYHFHKAEVDFKAAESVCSTRNSHLVSVHNNLIIPYLTEKSTQHLFENGYAWLGGQRGFPIESNQQWEWTDGTPFDYQNFTNEKTDSSEIYGCMKFSVETGKWLTASCTEKHQFVCASDRKQKVTALLDSCPTGTANRCPSRYVYFEATQSCYKIIIGLFYNTTSNSFENTDGTPVDYTNWSQGQPNPSNFNTYTTIMSDAHGSINAGEWFNVVNENERGFICKKAATLY*

>Cbn|CBN31237.1

MEGDKCTFKFPMATETEESARTYCEEYVPYHIITAVPGSPTTECRAEATLICHSGWVQMFGRCYRMVKQMMTKDEGKQRCEKERKGSKIGFMHREALPFRIAEYYTAVYQYWIDASEAITGNLIYDVPDGNLLIANDGYPYSLPNIALARVDKSTKAMVLCEYTPEMTQSESNWLLEKYGQIYYPTVRTSEGSYVRTASSLNRNENDNTADNKYCERVMRPFVPGGMAQSAFPTREFMNKVKEVNKNDRSLIVRTSAFSKNSQKAERQNKRCVSRAAADFHMIVTKPSGGSIAKTVEQSSWKYSKRQEVCDAATWSSAIVLSHEGSPGLELMSDARYAPIYCQSITNTYRYGPCPSGFAQYDRKSTGQRWCHKFVKSSKNYDSAEDNCQTMGAHLSAFTEGEEVDFLDKLMAGASLDTWIGAKRRKQCMKKGSFLEDGFDRDVTSPCSRVRVFEWLNEVAPNPPAVEDDYWRTPTEPNYIAEGEDCLSIVPGSPRRQLNENACPRRFYSFCGLEAPIVKV*

>Cbn|CBN31606.1

MRSIFVVLLMAAVVQTQFNFHGMSSFFSTDEEFPTQLQNFEGRTQTEIRTLKEKVEKLEKLVEGLQSILMKEWNTTESGSKYRLFEERKNWDNAERHCQGFGAHLAIIDNEAKNTFVTNLINSSETSDFAWIGMKTKTTTQTSTPFTNFDAESPIDGCAVMDAKGVWSIRSCIQLRPFICQIIKSDISI*

>Cbn|CBN31672.1

MRLLLLLLYSTTINTIFGQLEEDWGLRKACEKTGGKYTKRASRQSSTGDTCEMPFKVATRDEQDSRDFCELYAPWRLLKTERKKDAGRHTTICHVEATLMCEENWWQMFGHCFRMPDKHMVFTRGEAENLCKTSAPTGTEGTIAFMHHKYIVGVWRRYFRGTPQIWVSATESWNEYIQKTKTVDGDALALAFTGKHYDFSVTANSLIRIDASIRLNTLCQYKPPITPAEINYLGRRYSQIYYPSIPVDNGIMVRSASSYTRTAKNWDVCKKVLAPFMIEKIGDFVPEDYVLERMSRVNLGLTYLTRSGAIRELDGNGEHQQKNDATCHAYLPEYKIKVQEDRRADFPIKNITIDQNQDCDNMLSAAINHKDKAKLQIMSDSRSLPIWCKLGRPERFQHKPPPPGFIPFLRSDGKYMAHKLHKELKKYDEAKTICEAEGARLTGIDARKEAEHLIKIAQNEGINHKQMWVGGRRKKECMDQERWNDNKDHICYRSRVIYWEHAMAREFEESWWKDGPTEGTKNPDYASGGQDCLTLVVGTPGWADPKSGGFLDDISCRDVHAFFCSIPLEMTKVLVED*

>Cbn|CBN31834.1

MGKYRYLLIFFALFDVMYSIAELLTPIAVINTGYGFVTFITDGPFFDDVMSGQHGVASRCTFISLSYAILIIHFVYRYLLLFNPDHVRRFFEPAGVLILTLYFIFHGVSWTYICEHCLAPNEEIRDIIRPAFQEVYHVDADTVPCLTGQYMNASDYVVYKSWIGISSLTLFSSYCMTLYFVLGYKILTKINGLSTNNVAAAYIGLAQSSSGWGWQNGDTSTYNNWAKPPVTQPPTIGPTTTPFRSTLPPSEQRRSTAVVIGVDASLFSTNQLTFAQVDFITSLSHYIADRGPAEFAFFAYGCTQTSPYIYQYPNFVSTFEDTDKMIQTLKTTINQNCVRSSPLDFRSDADNIKAASALYPMANSSVITINVGATSIDVSRLSIPIGKYGLRVNTPEDITNLVPKVDSMIFGATGVSSAGVKVDVMKEDQWDFDVRKSEPSVFASSDYTDCAYMNTTDGLWYSDGTCATKRQVLCQYVLPLPPQPPTPNPTIAWQDPCYNEYTNFTYFEILSNAKCYRMSSSKKQFAEAENVCLSDHPLFLHVPKLVSIETREEEEQLKTLITGIPNEGMFWFGLKRDPTNSTNWYFINGDTYDASYSNWRDGYPRDADGCDCVALVVNDSSWINTDCNKPLYSICSYRFNGTEPTVKPF*

>Cbn|CBN32010.1

MKFVVFILAVTLCSAQVDRDWSFQAFCGHWQGNATYKPREGYSSLSGDSCSLKFPMATADEESARQYCEEQVPYHINEAVPGSETICKAEATLICKAEWVQMFGRCYKMKKVMMTREEADAHCKKEQASIAYLHRHALPFRFYEYFTHVSQIWLEASEAITSDLIYDTPGGNLILAFDGYPYNLPNIALARVDPTQKAMVLCEYTPAMTQAESTYLLRRYGEIYYPTVFTSEGAYVRTASSLNRNENDNMADNKYCSRVLAPFVPGGKAQSAIPTREFLDEVKRVNRNDKALIIRTSAFSQKADKSKRVQSSCVNDQSSVFHIYSLEKAGSIKIGKSEWMAGEPTGMCDAASWSSAVVMSREGSPGLEAMSDARYAPIYCQSITDVYAYGDCPAGFSVYERKELGQRWCHKLVVDLMTYDDAERNCQTMGAHLSGFSSEDEYKFLAQLMGPRYSKHYGTMEVFLGAKRREQCMKLGTGKSRGYEKDVNHTCSRNRVFEWKNNVAPNPPVTDNFWMSSAEPNYYHIDEICLVMIKRKQGDKGTLNDIGCAYKLAHFCGKEAPIVKVSS*

>Cbn|CBN32585.1

MKIQILIILLPVVTSAQLDLNSIRPCNVGCQDGWVPYSGNCYKKMFDVLTQSTAEQECVNLGSHLASFETTEEATAIRNLVLIAPLFSTDLLSYSSTSQDSWIGLSKTSNGAWKWTDSSEVEFTNLPDGTSVTGASCVSMNISGVWQPNECSSTVSSFICKRASATTA*

>Cbn|CBN32741.1

MRLAEAHSYCHKLIGDGSAHVLRVECGGENDYISGMVKGHSDKVWIDARARFDIVDGAAGLFGPGFVYRWPNGKIVRYSNWADGNGLEEIGTSNKCVTIKNDGHWINANCSSTAAVICEKKLHRPYSKFCPKHWVYNKETQSCYRTISKTNMTILEADNKCFDYGFEHRQDAMLTSIGSEDENQFVMDLAKEKDANFEFIYLGGYGRSRNGNKWHWMDGSDFTYMNWDRGMPFGRRALAVLVMNKRGKWINHYADKILSQYNAVAVCKFKS*

>Cre|FL81_00190

RDMFPMRVIVETVRSQHCLTCSHEGHMITDTYAIVAGTTTLNQLVDTVLAALGHSSMANSARGLIQVNNWKPLPFDQITENLDETVENLFKDISSHVVLKILSKPTTDTNAVQCISEVKNKLLKAAVNKTPNVLTNVENQQVKDVINTIISGDETLLNSEQLGAVNEWLDTLDTAEDRRSPTQVQRFNTLFEIPRLEKWFKQDANPSKQKMNNYLSQLNQSPFRRNNSKISYQQMCNWFSQKRSSNRSAAPTDPVQVTPAQTTASQLLPGLQLNLLQSLFGVGVEPRPKFDFSLMNEKLDDNRILGGSDSPSPADDEIHSNSDETIQDNMFVMNIKPEPETFQESTASSPDMSNSMRESLSSTSPKLISGLDLGSFSNHSSSTNSMPNVNSSAARSRLMFDPLTELPVLEKWFEENPHPTWMQIDQYTQCLNNCAYRENYPHISQHNVKIWFKNRRAKCKRLLNGMQEKMEQKLFVKMLKFAFLALVLLGLVSGLDVDSQNKISERLSSKLLQYNSFGLPEVSQRFQPEFAAKSAVAATNAPISCYNRSIDILPHDGTAQYGDLVESDFSSTCSDTFTFYVDNFIDEFFVIITTLDALNPRATVYNPIGGEVSSCKDYASSATQSIHLICNGKNIHGTGAYTVKLAADMNKPCIFEIRAPTKLTVDGGFVEDIRDDDVQQIILSTANQGIFRFPLENQASYLAFKVESEEFPIHPEEVHLYTNGQFDIKMDLNLRYGCNTPHITQQSYNCTSQNLYHAKFRGYDSDGNRFQRIYDFNCDIGDVVFTTPSVDISTTPARFCENGGILYNDTGCYCGEFFAGVNCEIPLCLNGGTSTDGGCICDIGYSGDHCQHISCLQLSDDNFEEAQSALAFVVRASSSMKAQLNEIAEAADRIVKYYEVHYPIFFQKFVLTVVSNNGVTFSHDYDSGKDFVASIRNLTAPATETECDDALLAGVSATIDNNAFKKYPIVNTRTQIFFMITDSASGSCNVDVSTNTFDQLRSISRLSGGLLIQTTLLQLSDATFSVAQDIWQYDSILTNDLEDCRKAPQFQPFFVDQSIDYLTLRATGYNLSPILTLPNNTQLSLQLFYSNGDFYVWRTAKPPVGAYFLNINTNSTHNSICQYRLMGRSTYRLYTSITDNIGTDDTYHAPVFQTTSHLVARMDGLYLDDPLDTTIEAIVWYNDPQTSQRQILYASSGTYRDQCQYEMYFGMFSCPQPYLPLYINIYTNDGYGQVVLRTATSYCSATIPNPVDSNCLNGGVYYNNTCQCPTHFIGDKCQQILCENGGNTLFGACQCPSGFSGQFCEVTKCYEYNGYGFWGFNHRSLTVMIHDSLTTRSTLRTLNDAAPRVINDILYQHPKWISNYQLVEFNATGHTLLVDSESGQDLVTGISSLYDQNRNHTSYSCLGLNFYSTLLDTISHDNVQWGGIVYVFLYGQPVQDLDSYEKILQRIEINKIQINIVQSSLNPCGQDIALDGLISLTQFSGGAFITATTPNAGNVFNQLPTHYMSSLIYENTALDCTDTTFYVPIDVGTQTFTAYVQGYLNADPVYTPPTDSLSTVTNVFNDLGTNARIDHIVRVCDDGWTVDGSHCFKFSFEEKSWFAALADCHSEQAVLTGVFNQAEQDGLNYGTDGAQFWIGLNDVLGGQWEWDTLDDKLNLTLQDTQYTNWETGQPSADPTKKCVVDSNKGAKNPSSRGWFTEDCTKQYYYVCQKHAYSADYIASDPEINSNRLVVYTYGVKNPSGAEYAHFYYDNFTMLEAQTLRYRDNCMYNFISTPFKCPNFYFQMLITGIDDAGYLYQRVVPAACIGGVNEDSCTNGGVYYKGKCLCTPNFYGDACEYAYCQNGGYLSATLDQCSCPSNYGGQFCQTPICDRNQLNVPAIGDVQRTFIVILDGTNNDAMKTVNDNFEKTLNGVLNSIQTQDPSWFTTFVGVVFRDAESVKANPSVPSTSQVFTSKNSADFASMLATEVKNNPYTAQQQKRDVFTGIVKAIINSNVVPNSKVFVITAGNAEDTIDRQTVISALALSHSSVNFLFIGDTKPPGDANTYDDPTVTSLFEAAHITGGASYQLTSPDDLQTTWLSILASLHQSYYVAIVDIFAQQAPQIAVLDTDNKQVESFSIVQSKTNTVRAFKQNGEQPGVWTIDVDYGMTNSGPCTLNIRSQSKLEVDLGFTQDVASDGGYHDGGAVLFPRGGDFVNAVVADISTGGTLTYAQIFDLAETRIAWASPMIKRDGCAYPFVSEYTFQCYRNQFVVALDGFDFEGHPFRRTFTIHCDGDIRPPPTEPSFSTTPEVTTPAGPTPKACDATTATVDIIVAFDSSDTITEDMYYATIGALKSIGNAINIGQDRSRIIMGTYDATSHFNGDLNTIDSFDKYQEEIADLFSLGYTGINGNNIQSVFDYIVAENNTAPLRPAPARKLLVLLSSQGWDKGNVSDGKENGYPDPTASAKALQQIGFETFAIAVGPTANLSQLTAIAKCTSQVTTPTALDKTVAQIISSLCGTTPVC*

>Cre|FL81_01778

MRLLLALFLLVTAVAAAPNHDSAFQNLPNYYPELKHQEPIHIRAKRAIFAAIGISNTCEDGWTGDGCKNPICTDPRPAPSSGQSELIELLFLKGSCAGSYYIPVDSDVGKTTQTIQIHVSASGVPYVNLTDSKGMVFTPTYTNNGDGYALTVFGDLPSGGYSLTVDNQNVPTTECIIEVNAGTSLKVSQGFVYSPQSDYTPQGESGIDGVPMYAVVHIASQQAPAQVHSITIRQGNSLEPIYRAALTRRYQCGYEFYAGQWQCQLKNSYYYHIDGVDSNGFNFRRTGRIQCMEHLTSPVPPTTTQAPLTSCFNNGTLLNVADQGQTCFCPELFSGRQCESVNCMNAGFPDPDMNECVCAAGYHGTNCQDVTCPLNWESFLTNYKTLVVVIRNTVSMNKYLDAISTAIYKELTSQAGNNYEVYKGFVLVKFGNGVYTNTYYPAYSQDKLLSDIKIASTTVGQCSDATFDSIASIFTEVAIYQKSPIYFFTDAIASDVEKWQTVIEMNTRQKFPIYTHYFVQDNCGFDDMSQGFQAIEYASYYSGGLILQPAPDALEFIFQNVIKATAYKMNSVLIDDLASCSTPTRVFFVDSSTTELMILAIGQSLTVSVTDPNGNTNTALKIVDSGTTQLYEISNPVIGEHLITVVSNVQNTPCSYRVQARSEYDLFIGTSTGVNDDASDSEPVVGQSTHIVAQLTGLKGKVADPFRLFSEISITSNVNLDNTYQKPMYYSSGKYRDGCGFHMYFGAASFCDFMSQPFYATVYADDGNGYTIQRTTTGFCSGTPTTPYPPNSCQNGGVTDPTNNATCICPPGFYGKYCENIQCTNGGTARGGQCVCPVGTAGTFCEQYMCTTMNNNPDVSFNGQSIAFVISTRSTMKDSVAKIAANVQTMTRDMQIASDKWINTWILIAVNSNTSTLLVKSNRPEDFVAGVVNLSANFSNYAADETSCQIQIEQAMLGAALLSEKRSSVWIFADSDGPNDSSYIQLFDVAQEYQIVLNLVGVGSSICTAAENNGQFPYYLKSLTETTLGSVYMTDKLDQVMLFIVSMYKSAVSHRYYVPDCTKATSYYMPVDGWTQSLTLSVIGTDLYNVEVMFPDGTKGQNSDYELVSINDPELKLNQYVAACEGSFWNHQQQNCFEFTATKYAWLDGWDFCHSQKAYLIHIDNADVNKFVFSQVNGYRAWIGLAFNAGQWYWDVPDGNFPQPLTGYTNWAPDVDPANPKYNHAVMNSNGFWEPADPNEENFGACMKHRYGQGYYPGEGVNIVPAGLWKVTVQSNSGSCEIQARSQSDIQVFFGFVTDPRNDKPSTYANIQSSNNYLIAYPTGVLPYTPDTKPSMEGKLNYAVLSSNRTITNSLPLGNRVCTYATISAPFSCPVTDGSISEFSIKFTGIDQYGYAFERYGDALCTKTVISCANGGFVNNGVCVCRAGWVGTTCSTPVCQNGGIEKNGACDCSSVPQFTGQFCQLAHCEPPYPTSFNDKGRTLAIVLETSYNMGSSIFQLKRNLKASLDSINNDATLQGWFNNFVLYPFDSTSNQASWYPPTISRNSDDIVAAVKNISTMSCPGSAPCSSQCPRPIVSVLKNVLDMDALASPNSVILVITRSSPEDYLQVGQIAQKLQDKKAYINFAFPAIDSPCGEGWNTPNVDSLYQIISYSQGNTFTMNAVDLSKNFLTQYIPTLYSSGGIAASSGNCNNDEIIFQVEHEMYEFSIDFYHPLMENIRVFDPSGDELTKPDSVIASDTNYIGVFPVNETGATRAGTYRILLTGTGGNNCFATVRGRSNLEIFLGFVDSAADSNNGATIDAAHHAPINLENNTIVVHANGLGQGIVRYVQIVMPGFGLMHTTEMRRRDAECSYEWYATTPFSFDYDSYYVIVYGSSEFGSNWKRNFYVSTVGSRPPLPPPPASCDLQQVKQDTLFLIDSSLKDTNVTFTILKQFAVTAMQPYNYMNGLAQVASILVYGQAQGGFSFNAGENSYDRVSGLLNNLTYIAQPGQNVTAGLQYALDYYDMPSQGYRTDPDVKHLLVYVTNTNPTDADPSELIRTMKRSGLYDVVVVALDMQPSEQLTNMVSSRCYYYAQDYHDLMNYGVNLVQGQSCMRFNFCNY*

>Cre|FL81_01910

MRSRLWPFWFSLLYFLSLSEVVAQRNATCSGTVVLNATKDFQYLTTPNYETSYKYPAFLDCKFFIKAPDKSRVVIDIIDLEMEPRIFEECSDFVGLSEDVTEKNFSRIITLCESLRMRQFISTSNTITVIFQSDELFEFRGARLSYRYYELPSCPPGWTELSNETCVRLETDQKTDWVRAQNRCLDQRSNLLMLENDAKVNELEKLYKAVPTKLWTGNNDAAIEGKLVDISSREAPHLISQTFSSLTDNNDDNDCMTLQFGDTEMLRMDSCASLNGYICEMKKDGTSVLYDPPIEEIKGGSFSKTSQYTLWILLFLIGLLFLTILAFLCFICWKQKDARVHTENATIQQNAFMSDSAHRADQPRVAAPGQNTVHIANDTNRTARSSPVVVPIENNRPPPKKFPMAPVPNRLPMHEEPSGSEPAVETAILGGDNDIETVSAAPQPQPRTLPPMPTREGTFQSINTRDGSTMRTRRNKELFERPIMHVLDNVSAISLDEFWSNKKP*

>Cre|FL81_01945.1

MRTWVLIAALAVVCLAAEQELSHKERVRAVLKSWDPKADTKFFEPIRQQKYRTTDESDLFLDTHHISKRSIAQPHVFAGQATRGCNLPGYTGETCQYPLCSARNPYVPTNQASDDVQIDAVNIANCSVPYIIVVDETMWDIKIELESEVPLNPTFFLQYENGDIISPDSDRQLPTSYTATYAFLAPGQYQLGSMLPGSTGQEYCTMQMSARTPMKISGGFISGNQPERNDYPNLKVAYFDTESVVAVHVQALEFPGQIQGIGFTGAENHISRYIPIGTRYNCTYPYILERYTCKRTSNNDVGRNFLQVEGISNGGYLFRRILSYQCVLPPVSTTIAPSPTTPTPPLTTCQNGGQLLTDASGAPYCYCFGLWSGNDCSQMICANGGFLPTPTSDRCQCPEGFMGFHCQNIICTDTSGFDFNAENPTLTFVIRSRYQLSDVIEQVTNATDEIVQELASEPGYLSNFIVVLFDNGKLLLNKRYDSWDGAKVDLLKAIHSAPTDGGCDDVVFSAVASALSLYPTNKSPIYVITDANPNDNAEKETVFHLESYWRAPVYFIYVQPGPADGCNTSPDSSAYRDMVDIASRTGGNTFYFSNRTNISPFFYQHMLNTLFRSQLVLSGDYSHCVNQNIYKTAAIDLTADLIVVVATGTDLKLVATSPLGSYPDFNIAFTDGVNYIWTYNNAVNGQWFFTLLSGSPNAACTLKVYQKKFNFGGMTQYSPDYDIFWSFATTLTSAAGILRQPVLGFDASPVFHVSNYPAFISMDRVHANLQIYAIRDGLQTEVYGASGMWRDACEFNFYFPPFTCRVPDEVLYFNFFARDNNDMSLQRAGTMYCASIHPTPPPDHQCQNGGVMNPSNTTCFCTPEFSGTYCQDLVCYNGGTNKGDHCVCPPGYGGESCELARCIETGPTPEFIRYGVDMIFAVEITQNSQTTLMMLDTQFGNILRDIQMQDRRWIRNFVLVGFNSTWGGPIAEAPADNLTAITDAMDKIRSSGSTDTGCTVKLWDALNHAIFSRELIPGSFVEIFQTTPEDDTDTRSLGLFYDMSRRMDLVLYGFLVTNPLLKPAGFVCNAQIENYYTLLGIVGGSTGTTYVIQAFEILNAVKTIPIQFSSGQVTFNYDNDCRHDDGLITYFPIDAYSQTIQIDTFGYGTNIAIYTGDGVLYSAYQIMYDSFTGQGIYEVRKGCDKDWEEYGQYCLRFILGNDNVLPVPQAMKFCASAGGYLVDDMDAGQNEFLNGAAAYTQFWIGLYRNNDGQFVWDRGTGVTPAPLSQTETYWADNEPSNDPMNQCVYYDGRADPKKAWVTDSCSTPRPFACHKNRYDPEHRPNTIGEDDLPAGQWYVMVKSNPGDGYPSNCSVSVRVQSSLQIVTGYSTSIGDDIPQPDPIQDSPNNRLISYVHSLDNENRVPILTDAILWDAYTGNFYNGLKYQPRFACQYQWVSQDFPCPNGDNPANEFGVLHVGEDEFGNTFQRLTYGHCSKAQITCGNGGIRQNGVCVCSEYYVGSRCTVPICVNGGTRNSDEATCSCPDGYAGPNCQFELCQPTLPQLFSNDRKSLLFVVETTRQNSDTVNQLIANIKSIVSSATSSTPLWFTNFGLVTFDTTGRTFEEFDYTNIDDLIVDLKAQSNGINTDGACSMPYLGVLAHLLEHDDVIAMPNSEIFLVTAAGPADLDKYVEAMTTLFNAQAHLHYVVSKSAGCPTFNGVNNVEDMTWLGYGSSGNILFTNPTNLVNLLNNYLPTLYGASVLQDPTGNTNYTCSNSKWLVPVDMNTTFIYVTVSSEFGSLSANSPLSTGVTPEIIYNVDDQKVYRIEVDRLGGIWQLGVNSPGLCLAHIYSTGGAKVYTKFAQPNSAGGKPDPLGAHKDGSSVQPAAGFDNTAVFHIAGDPFHNGQLQYVEIFDIGSNTITNILRSELYRRAECSYEYYSDLFTCNGDMIAVFVHGVDEYNQKFRRQEIVICNGRAPVTNAPVTGTIVPVTQLTQGPVTQVTQGPVTSTTPVPHTALQFDIVFLIDGSQTAQDSFDWLTKFVQTFMVSFNVGLNGARVGLIVVAPDLDDQPPPAAQFNSITSQASLISNLNLLKENYADFDHAGQVLTYNLQIVTSNDYKSAGYRSNIDNHVLVYITTNTAFYTDPTPTAQTIIAQQQYGIITIGYGTGFDNNKLQTISGGAACSFTAPDFARMRTWVLIAALAVVCLAVEQELSHKDRARAVLKSWNPKNDLKYFEPIRERTYSISDDFIVETHRSKRSIAQPHVFAGLATRGCNKPGYTGDTCQYPLCAVRNPYIPNSQNSDDISIDATNLANCTQPYVIVVDETMWDIKIELETESPLKPIFFLQAENGDLIYPDSASERPTSYTAKYEMLPPGQYMLGPMAATGEEYCTMMMTARTNIQVTGGFISGDQAERSDYPTLKYTFFDTESVVALHAQGLDFPGQIQGIGFTGAENHISRYIPIGTRYNCTYPYILERYTCRRTSNNDVGHNFLQVEGTSNAGYRFRRILSYQCILPPVSTTTVPAPTTTAAPLTSCQNGGQLLKDASGSPYCYCFGLFTGNDCSQMLCANGGFLPTPTSERCQCPEGFTGYHCQNIVCTDTSGFDFNAENPTLTLVIRSRSELSSVIESAAGSVQSIVDLLSSEPGYLTNFIVVLFDSGKLLINKRYDSWDAAMVDLLKAINSAPSDGGCDDVVFSAVAAALSLYPTNKSPIYVITDGTPNDNGEKETVFHLESYWRAPIYFIYVQPTTAENCNSSPDNSAYRDMVDVASRTGGNTFYFSDRTTISTFFYQHMFNTLFRSQLVLSGDYSHCVNQNVYKSVAIDLTADMVVVVATGTNLTLQVTSPTSDRPTFNTAFTDGVNYIWTYNQQVAGQWFFNLVSGSPNAACTLKIYQKKFNFGGVSQYSPDYDIFWSFATTLTSAAGVLRQPVAGFDAAPVFHVSNYPAFVSMDRVHANLQIYAIRDGVQTEVYGSSGMWRDACEFNFYFPPFTCRVPDEVLYFNFFARDNNDMSLQRAGTMYCASVHPTPAPANQCQNGGVMNPTNTTCFCTPEFTGTYCQNLVCYNGGTNKGDHCVCPPGYAGESCELARCLETGPNPEFIRYGVDMIFAVEITQQSLASLVMLDSNFQEILRDVQMQNRGWIRNFVLVGFNSTWGGPIATSPANNLTAISAALHSLATTIPSDTGCRVQLWDALNHAVFARDVVPGSFIEIFQTTPEDVLDQRSLGIFYTMSRSMDLSIYGFLSARPQSQPVGFVCNATLPDYYVLFGIVTGSTGTTYILQSAEISNAVRLIPLQFSNGQVTINELDDCRHDNGMTTFFPVDAYTQTIQLTVFGYGTSIQVYNGNGVLAEALELFSDDFTGQSVYEVRKNCDNGFEPFGQYCIKFLAKSEDTMSMPQARNFCATAGGYLADDLGDDKNNFFKTGSANTQFWIGLFKGSDGQFYWDRGQGVAPDLLNPANTYWADNEPSNDPTRQCVFFNGQAGDVHKTWVTDSCATVRPFICQKHRYDADHRPNTIGDADLPAGNWYVNIITTPPANMPNYCTLSVRVQSSLQIVTGYTTSVSDDNPQIDPVQDSSANRLISYVHSTDNENRVPILTDAILWDAGNGTFYNGLKYQNRFGCEYSWVSQNFPCPNSDNANNEFGVLHVGEDEFGNTFQRITWGHCSPAEITCGNGGIRQGGQCICTDYWVGAQCTVPICVNGGTKNDDERSCSCPDGYTGLNCQYEVCTPALPQLFSDDRKTLLLVVETTRQNSATVTQLIANLKSIVTSATSFAPLWFANYGLVTFDSTGRTFENFNYTNIDDLITDLTAQSAAISTDGTCSLPYLGVLAHLLEHDNVIAMPNSEIFLFTAAGPSDLNKYGETMDTLFNTQAHLHYVVSQSANCPTFDGVNNVRDMTWLGYGSSGNILFTDSNNIVSLLNNYLPSLYGASVLQDPTGPTNYTCSDGSLPWFVPVDANTTFIYVTVSSEFGSLSVKDPLGLAHNVNPAYSVNNQKMYKIEVDRLGGIWTLQLVNPPGLCLAHVYSTGGAKVYTKFSLPMLVGKNTDPTGSHQDGRNPVPIAGFENVATFHLSGNTFHAGQLQYVEIFDIGNNGGATNILRSELYRREKCSFEYYSDLFTCNGDMIIVFIHGVDENNQKFRRQQVVICNGMNPSTGQPVTGTMAPITQATQQTQGPITQQTQGPNTQPTPQPGQSTTQPPVTASPSPSPSSALQFDIVFLIDGSQSAQSSFDSFTKFIQTMMVTFDVGLNGARVGLVVVAPDLEDQAPPAAQLNSISSQSSLNSNLALLKDNYADFDHPGQVLTYNLQVVTSNDYMSATAGYRSNINNHVLVYITTTTAFYTDPTPSAQTIIAQKQYGIITVGYGASFDNNKLQTISGGAACSFTATDFATLNNQIKPIQQLIINANTNGGNYCKSN*

>Cre|FL81_01976

MSPTLISVFLLVLIGLHSSVSAASCPAGTKIYNSKCYAALGMDMTRDNALAYCKKTYGSYARLTTPLTYAENNFVTEQVQLHANWHTWLEFVADGTYIVGDDGRPPVYTNFAVGEPFSVSLGYCITIGMNGYWYAQPCTDSHSALCEFDLVPPTVKPTIAPTLAPSGYPSCVQKYLNFVPCLSGWDYYPPTCSCFKIITNTTYYNAMNVCRSIGGTLASVHNAGEAAFITRLGAQANSYWTLTATARDNIIVGLTYNSDRKIFQWDDSTVFDYAQGFAPAEPADYTSQGQIVLSNPMGYATYMRMGNCAFQTCRYAACKRYVY*

>Cre|FL81_02592

MIFHFSESSNLHYSPQPLDLYPLILHLVIKLFNWSYKDFGTSAFSDISLEQRQRFADFPAETGTCPDGWIRFSDSCYWVEQHKQSFAEAEKRCYEKNATLFVVNSQDEWDAVREHFPQIGYTWIGLVRFTHRERSEDTPTWQTEGAVNPAKLNWLIRPYKPVSNGWSILSNCAAHYSASLNLDASAYTYYQPCSFKYYSICERNSTILDFLNRKFDIQA*

>Cre|FL81_02666

MLRVLCLTIACFLALVAGGCLPGDASFESYCFSFNRLQGRFDDAQAHCVKSVGGSLVTIYNMIENNWIQKLAVDNLDADYDLFWIGASDAIKTNDWRWRDNSKLNFTNWNRGQPLEDRHCAAMSVASGTWFTEVCTIQHQFMCQYPNGDYPTGAPYTCPPCQACRFF*

>Cre|FL81_04478

MNLLFLIASLFSSFLIVNSTVTCNYGDTEYDGYCYTFVDQQLQFPDAQAYCVSLGGVLVRFRGNADGRWLTSTAATKFHATYGNFWIGLHYVKNENNENKTLIWDDGHDVNYTNWSSGSPFSGYDYVGARLADTKWVSLLAETPLPFICYYQKGQNRNQPTTPVPSELPTGICDGAELLLGHRCFFFNPTLLNYDLAKQECEKARKTLAIFDDFSQINFVTSTAISKFSMTYGSFWISLRKNSNDENDKKFYWADGSVNTLNNWTPGYPFQNQFVVSLQVSNSKWKTSDNSTYMPSVCSGYVQ*

>Cre|FL81_04479

MIVSHFYVIINNEFESQFVINVTFFRFKMTSMDLIGRESGKDKTLIYLLSQTAIKNKIKCHTNIIKNRKQHQETTLQDHFNIMILSLSLFILGFIGLSETATCRYPHEKFIGKRCYSFVSKRHPYAAAEEYCHSHGYALATVDTAITANFLASTAATEFGSNNGQFWIGLSRKKDYSLFYWDDGTNVAYTNFEAGFPNTKDYVAENTLAEHKELEFVCSYDPLSTTTPAPTTTPTTVKTTTVTTTTTVKKTTATTMTTPKPTTTVSTTTTTTVPPTTTAKACPRGFTLFEPTQKCYIVLVYGNDSDYPVVPDDAPFLTKENQRCAKYGATVATVHSSGLNDILRSLIYERFNDTRYATIGLRNIDMKTIGGKWAWFDGSATDYLNFGNMFPKTGDFIAQTSSQGFWVTYTHTAPNEGVVCSVDF*

>Cre|FL81_04694

MTGSTDDSAPDTSGVSRIFWNYEMTPYYGAWCYEIGLHYLEEGRDDQLNQVLSTPYDLAIVDETYTSLQGAISLKLKENHGTKIIAFATTELMPVAANMRGYARNPVNVPNTFLLSSEVYENNRNGFFSRTRRIYEYLVDQIYSGPIASRNSEASGKLLGLESATKSRIFENSLLTVNDFPDTFSFIQSRGNDLIPVGEHCASSENLPSDFRNFVEDPISKGTIYVAFGSYLNLEDGPEGTVESFVEALNYFEDYRVIWSHKGNVTGAKCHVKFVNWAPQKELLAHENTVAFITHGGLKSAKEGVCSGVPMLFLPFYGDQPRNAHRFVTNGIAEALYKKSITAVDIKQKLEKLLEDPSYKENVVKTRSYYLDSPMSSLNLGAFHISRVLRRPESQFIRFKRRSISMTHLQYLNIDIIFYSFCYLMSPLISYNFPSSTTNDHGLGVSEKYHRAKYLETQQKLSDASQPSLVLAADGAILPCDANWHQYPDTGCCYRISDEKSDWYGGTNICKALNSDAQMASFHSQAESLFFANKYSSIHAWTGLSQTEVPNTWTYTDGTPDWHWFPALTSASSAADSSCVEMMDGLLGLLFALSLQKGQTNPYSCTEVNQIICKYCPKETTSSTTTTTTTTTATTTTKTTTKTTTPTTTTKATTKKTTTLPSASVTCTSNCPAQSVNFNGKCYKKCRGSVKFEDSCNECGGTMITISNKAEKDFVSRVFGENDGTVSQIWIGNTESNGYLDWEYGQPSKPDNSLDYCISMDLTAVDHVDEFLQNKTFNDILEMSQKFQRARYLETQQKLSDASQPDLVLAAEGAVLPCEAGWHQYSGTGCCYKKTDAISAWYGGTDLCKALHPEAQMASFHSQGESEFGTPGVWTYTDGTPDWHWFFAQSSTMKPESSCVEMLDGVLVYLFSWSAKKGQTQPYSCTEEIAILPERNNFNEYNYNYNNDNFNYHNNSHHYNRNYN*

>Cre|FL81_04696

MTNFQKCRGSVKFDESSDWCGGTMVTISNKEENDFISRVFGENDGTVSQIWIGNTESNGYLDWEYGQPSKPNSALDYCISMDLTAGPWRGKYKYLPCESTVVSSITSMNP*

>Cre|FL81_04698

MLFLTSILLLVFANLCSSVEVNPCDSTWHYYNKTGCCYKTSTDLGTWFDGSAICAKMHVGAHLASLRNEDESKFVAKTHRNGLDGIHAWTGLSQTQNANNWTFTDGSKPWSSFMTPYIFPNNHTSCVEIVDNWLVELFQNTGKTQPTFCYHYRKSLCKYCPVPVVPTIITTTTVRTPAARIEKAHPRSNQPELVSGCSSGLAANQVPVIPVSNATTPVPTTTKTTKAGIISAASLHTQNGNQSNKTRSI*

>Cre|FL81_04714

MRFYFLVATIFSGLLFSQAAPVDVVDGSGEAPDTLLQNTEEQPHQRLRFHNWDYKDLGTTAFEDIAFPARQPPAAVNQTEQCPDGWVRFADSCYWIETELLGFAKAERKCFEKQSTLFVANSLEEWDTVRSHSKEAYFSWLGLIRFTHYEKTEQNWLIKPYKPLSNGWTQFANCAASYKSPASLESASYTFFYPCTYLLYSICERNSTIVNALQ*

>Cre|FL81_04808

MSKILMKILIMFNILVFTNGQVDRDPSFQSMCEFWNGKDSYRPRDNGYKSMSGDKCSFVFEVATDTEASARRYCEVNVPYHINDAIPGERTTCKAEATLICKNQWVQMFGRCYKITKELMTRKDAGEHCEKEKATIAFLHREDLAFRINDYFKFVSRLWIDASEAITKDVIQNVQGGNLLLALDGFMYNLPNIALAIVDSSEMAMVLCEYTPPMNQAESNYLLKKYGEIYHPTIVTSHSSYIRTTSSLNRNVDDETANNRYCTNVLKPFIPDGKAQAAIPTRDFLNELARKKVGGIVRTSAFSARTTKTDRQNRQCVRNSNSIFHTYVSGLNNKGGYEPVESSEWRQNEPNEMCDGATSSTAIVLSVVDAYEYTNCPNGYQMFYRKELGQRWCHKYYNGPGVPMLNYDEAQAKCASEGAALTGYTSPEELAFLDDLLTKGNNLNRDTLIGAKRRDDCPQYGNKYSGGFSPDVTHRCSRKNVFEWKNGVAPNPPNIEADWAYPDEPNHGYDDEKCLVLLKGANSDHFRADMTKKLNDHSCTKKYHYICGMEAPIVKQ*

>Cre|FL81_04978

MSNLVPVHKSLESSLVDVPLLSKEVDDSDVFETSHSSQFQMFQLKHLVTLSVLLIGVASRGCFDADDKEISGFCYKFVNQKLTFEDARDWCHYKDPVTQSYLAYIQNQFTANFLASYGKTIFGSTDATFWIGLSRERNWMPFTWDNGYQLGQSWSNFDGQIKQNYVAERVSNAKWTTFAENQTNYFVCSYDPTDPPTFAPKTPTRPTTTTTTTTTTIGPTTTGK*

>Cre|FL81_05261

MFQPMLLVSLLLLPLISAQCGPGAVFQSSSSRCFTFFRTGVDFQSAEAICATLNGHLVSIHNAIDNAFVSSQAQKYFDGSAWLGAKTTAPDVTNPLNWYWTDGSNFDYQNYRVGEPSAQGATACMQLQTGTAKWLTTNCSTQLPFICSYSSSVTPTCPTVTIPSHCPSGYTWYDETDFCYKARKSTVRFTNFNDARSACQADGGDLASIHSQAENQFLVELSKAGITNKDKGHNDDVFIGLIYQNSKWQWTDGTAVNYLNWGDGEPNNMEKEWWTSLVADPHEDRNTEDTRWNNVAQVDMRAFICKRAPLH*

>Cre|FL81_05393

MKTALILAVSLVFCHLIPSDGGQCQMGWSPLYPYCYKAFHQRAIFGEAEGICTANSAHLVSIHSLEENDLVKMLTKTGHLPVSYWENYVRIGLFYNTATNLWAWTDGSPTSYLNWAPRAPEYMYNMQYHAALMPDRSYNDSDYNKEGGQWYNIQNWPTRAFVCKKLNYEH*

>Cre|FL81_05692

MRPSILVGFFLLTLSGIGAVSVAKSTDNDIVLKVSTDKQTSRDGHHFTGEWLESPWGDLYQFRAGDQNWLVAREHCLSLNADLAAIRNVEQLDWILSHYAPLSSRFGQRLVQIGLYAPEGQTHEWKWLNGNEINKTLQWSSGEPYDHSMEGRERCGLLNVEKRLLDDVDCESTSPDHHAQRYICQRTSESHKQQQRSNNYIWQKIENLFSFFGIGGSPTPHNATVTNTNDYEDEVVKNETSTAKPAVKFSDSEETSSEEEESVAKTLAALPKIEGSGESTSLKELQEPEGSGQIVDKKAIEAADLIPGVDEKKLDKMIDKMEEMIKSIDDLTVPPAGLERTTVSTIVVKKEEKFEEKKKTDDKKEEEHKKDKELNDNKISESIEGDFDHEQSKDMPKADIEPPKEEDCDEEDGSGSGEEEAAEEEESGEKLELAPEKEDKIKEFLGVLRLFLDRAEHGDLRKLLDDQSGKTLLERMKNAVREANKREFEMLEKLEISKKKGEQDEFVTKKDQMSTEEQKDLYKKISSAVMKAAKLHKIEEKVQDEQAMEKFNIAKVKADSEEESEGTLEVLKSAREGKAEKKEKVGDDDYYGDYLDDNNVIKVQNREKKDAKKGSKEKNASEDKKESGEKKELKKKNEQSHDEPKKEEVEKKKKEKKVEEKVESEEKKKEEESMKEEKPTEELKKDQEVNAEEKNVLKQKEEEEKTTEETTTMTTEEQEKETTISVEQSSSVSSIKPAEAEEDEAELEASGQEDIVTTTESTTVAIKEVPSEEIEKIAKLEAKQLVEDEKVTEETKEEATTSIPSSSTESEKTSPSTTTSEATTPTSTTPVTTTKPVTVAIKTVSPEEMEKLSKIEATEKVTLLPPLPTFTFPTLAPFTFPTLPTLATAKPTPAPKVPTLEEILGNLNDQFKKLLSPPKPLPPK*

>Cre|FL81_06741

MVLFLLTIILILPELARAQLKCPPGYTLVNDKCLMIIETPLTHSRAEANCTFNGGTLVNIRNAITNRAVTQFAATAGIDKTWIGLFCFENKNTSMCYYDDNTGPILDYNSFASGYPMVDGIYGGCVYMPTSGSLAGKWVSVKCEAESIPVMCEVPVSVYDSNCAHNFNGYCYTPSSELPITTAKFADARKICQDKKSDIVSIHSKREVDYIKSLYRGSKSQVLIGAQQILPNTYTWLDGYDWNTFDYRDPLDQQRTDYNCLTMDSATAPQNPSDFSNCNTTLLMTPGTITSDGYLSTSPPEPAVYCTWRIVTTGPYRVRLLGIAVGVFICLRRRKGKSGGGGGKGKK*

>Cre|FL81_06742

DVNKTYMNLLLAIFLILPVLARPQDQLECPSGYTLVIDKCLMVIKTPMRHLEAESACTYNGGTLVNIKDAITNRAVTQFAATTGIDKTWIGLFCFENKNTSMCYYDDNTGSILDYNSFASGYPMVDGIYGGCVYMPTTGSLAGKWVSVKCEAESIPVMCEVPVSVYDPTCAHNFNGYCYTPSSELPITTAKFADARRICQDKNADLVSIHSKREVDYIKSLYRGSKSQVLIGAQQILPNTYTWLDGHDWNSFDYRDPLDQQRTDFNCLTMDSATGLWNRASCDYEYAFLCKRPIAWSTVKPPTETVLAQNPSDFSNCNTTLLMTPGTITSYGYLSTSPPEPAVYCTWRIVTTGPYRVRLSFTDISTYNDIYVYNEDGTTFARVRTTSNFEQGMSIPQELQAFMAYGFGKELPPSEVEKGHKFCNDLTSEQHDKLYEAFKGTCEKYLDTESYKSGKTNTAGYIICCDSFGMCGMSGWMIFLIILIVLLCLAGAAAAFWNLVEAKQYFANHDFIRPLSAAEMERGKKFYNELSSKEQNELYSLFDASCNTYLDSKSYNSAIREPDMYSTLCLAGAAAAFWFFYYKRKMGGRDEEKEIESTADTANTGHDISVETY*

>Cre|FL81_06749

MYLSRSSESTRAQWVTTWCNNDRNSMMSLYPYICEAPPTEEGTVAFSCEFIYNGNCYFRSAGLGFGYSPSNATEAAYACPHYNGILTSIHSKMEVDYIKNIYRGTNISRIYIGAQSALMTDRLSWIDGTDWDFDYMNPLDTNRGMCLVMDVQGDGFWSRVDCNLQFDFLCRQKIIPFTPEVSSKETHPEIVLDSSNCNSTFVLSPGSFSTFRWPQVPDVMSYCTWRVASLGPYRVGIFFDYWGTYGSLTIYDEFGTNIGEFSGIYEREPFASFTPFNYATVKYEPGNATGGNRDTGFHAVIRPV*

>Cre|FL81_06791

DNRAVSTFAASAGATTVWLGMFCFATGNTTTCYHDDNSGPLSYNSFAAGNPAIAGNGGCVYMQTSGKTAGQWLSAPCEVVGMPFICEVPLTNADPTCTHNYNGWCYMASHEMQLATVNTTYVRAQSICQSNGGNMVSFHSKPEVDYVRAIYRTSGIQQIFVGAMAFLPDTFDWSDGSVWDFDYTDPLATSKGNCMTMDLSSRPNNGMWSETNCQNINYFLCKRKAGAAQPASTAKSMVEDEDNSVVEQEKPVNPKFVRTLKGTITSFGYPNTRPPITSCTWNIAALGPYRVGIYFTDFSVYNAVYIYDEFGNLITSPNGNMRPFQVLGATNVVKITHDSRYDAAYNYHGFTATILPF*

>Cre|FL81_06792

ELSDAKIACPTGFALVNQQKCLKIFPNHLKHLEAELDCTHFGGTLATIHNAIDNRAVSNFAANAGVQNAWIGVFCFENQTTSCYYDDNSGRLSYNNFIPGHPRLDNGYGGCVYMTTSGKNAGQWSNPTCLHNFNGNCYLPSHELTGSPPNATYSDARGICHSNSAELASIHSHQEVDFIRTIYKDLDFYSVLIGGQVSADGNNVTWVDGSDFDYNYMNPIGQTDGNCLQMNTIKSQTNNGLWSKFKCERINYFLCKRKIGDAVNPKFKITTLGYPNSVEGIFCTWKIGVLGAYRVGIYFTDFSINGALNIYDEYGNLIEAPSISRYPFQALGPTNLVSMTHDSRYDKPGLYHGFSATILPY*

>Cre|FL81_06793

MNSHILFALFSLFCSTYAASTPVCRDGFKLVNRKKCLKSFPDYKLHDEAEADNRAVSDLAGGHSNPFVWLGVYCFSNVTTSCYLDDNSGPLTYSNFATGYPKRDAQYGGCVYMTTYGPDVGKWFSSPCEYTGLEYVCEVPSTVDDKACTHNYNGNCYLPSHELSINTPNTTYQTAQNICKSINARLVSIHSKSEIDYIKSLYTNSGIQQITLGAQAFMQDTFDWTDGSSFDFDNFDPLATTKGNCLQMDLSSRVDNGMWSQINCQTVNYFVCKRKAGVAVTVAKKEEQEEEEEEKDVNPKFKFQMVTEKRELPIQHLDLSDLCNSTLFIAPGVITSFGYPAAQPPITYCTWKVAVVGAYRLGIYFTDFSVMYPVNIYVEKNNLLASPKYEMKPFSVLAPYNMVTLTHDSAKDAELQFHGFSATILPY*

>Cre|FL81_06795

MPKLYSLFFLFFNLFSIIHASEIVCPSGFQLINQNKCLKLFSTNLKHLEAELTCTSYGGTLVNINNAIDNRAVSNLAASTSATSIWIGTFCFSSRDISTCYNDNGSGNLTYTNFAAGYPNVGEGYGGCVSMQTSGPTAGKWISAPCEVAGMPFVCEVPATVAEKICFNGGSKMVSIHSKREIDYIREIYKDSEVTNQITLGAFSYDSDVFNWVDSSRWNFDYFDPLDMSFGDCLGMDLSEEPNTPGQITSLGYPISKPPAAYCTWKLATVGAYRLGIYFTDISVAKSVYIYDEYGNLLADPSGNLSPFKVLAPTNIMTITHDGSSGDHGFSATFLSY*

>Cre|FL81_06952

MFQNSIIGINGTSGRQVYIEYTISYDQTSQSWNFQSMRPLRCFSAAYKMFKRALGPWCITMPVTTPRKYTESASICARTSNSVLSGLDTPEEFEYIKGPLEQVARKRWLNASYPLYASWLSGAFSFADPTLSANPTGYLFNPGKPDGTGEDCLAFRVNSDRTCGIDDIPCDQDRTSDNSTWMRGLISVDSVMIVTKGTPEVYINYSSLAMKWDQCLTWCTQNDLCTVKRSNDIESKFAYKVKNENASTCPADDTIGETNFIFGTNRTSGRQVYVDYTISFDQTSQSWNFQSTRPLLCPSAGYKMFKRPLGPWCIQLTSNSDCQNYTAIESICTKTPTMESIHLSGIDTPEKSDLPRIKCKESDSKKAFSFADPTLSANPTGYLFNTGKPNGTGADCLAFRVNSDRTCGIDNILWLKSRCDQGRTSDNSTCLDMNLLLPIILILPVIVNSQLSCPPNYTLVIDKCLMIIKTPLTHSQAETDCTYNGGTLVNIDSAIINRAVTQFASNAGINKTWIGLFCFENRNTSMCYYDDASGTLLDYNSFASGYPMVDGIYGGCVYMPTTGSLAGKWDNNSDLVSIHSKREVDYIKSLYRGTIEPVDILLGAQQILPNIYTWVDGYPWEKFDYRDPMDRGAQDIDCLTMNSVTGLWTRAVCDRPLLFVCKRYITGNPSQTEVAPTENPSDFSNCNTTFLMTPGTITSYGYLSTSPLEVNCTWRIVTLGPYRVRLFFTDVDTYNTIYVYNEYGIQIEAVRYSRSVISRSNIVTVSWQGIINGKPETYTNSRILPLSWDQCLSYCTQNASCLSVYMENNVCQKFEGGDLQTVKRSNNVLTKFAYKLSNVAACPKDDNVDGKTYIFTTNVTSGRQSYLEYTITYDPSLQSWKSAKQIGRFRALKPMATSWISGVRKPECIGNATCQGLSATVYCPMIVTNGVPEVYSTPNSLALNWDQCLTNCSGTVSCMAVYSNAEGNCQLFDVGQLQTVKRSIQSNSKFAFKIKDENLSTCPVDDRVEGKGSYIGYNATRSQRFYKGYTVNFDPASLSWIFNSTESLYCSDKTYQFFMRPLGPWCMKIVISTDCRISSDIADTCGMIPGGMLSGIESQLEIQYIEQRTGGWDVYKNPYNAIWISGIRKTECIGNTSCQGASAFSFSDPTLSSNLPGYQFKPNKPDGTGADCLAYSIEGDISRGIENFHSLDLGWEDCLNYCTENATCVAIHEEDTVCRMFEIGQLQTVGRSVSVESKFAYKINNDDLLSCPADDTIGGKGYFFGIDTSTGLSTSGRQIYENYTITYDSSTQTWNFLTNGPLMCPSAGHQMFLRPLGPWCMIFYPNSPCQDNAKIVSICASSVNKYLSGIESPEEFEWVRARAYGANWFDPSIRLNAAWVSGAHAPPPIPQMFVWAVKFVASGLHK*

>Cre|FL81_06997

MISKSVSIFLLLALFSIKYSEGQSCPTGFQLVNQNKCLRVFAQKLKHLEAETDCSYLGGTLVTIKTAIDNRVIANIAANAGASSIWIGIFCFATGNTTTCYHDDNSGELTYNSFASGNPAVTGNGGCVYMQTSGKTSGQWLSAPCAVVGMPFVCEVPMTVADPTCSHNYNGYCYTGSTEMHLATINTTYERAQSICQSKNSDLVSIHSKQELDFIRAIYRNSGIQQIFVGAQAFLPDTFDWSDGSNWDFDYTDPLATSKGNCLVMDLSDRPNNGMWSQTDCQNVNFFLCKRKIGDATVATTVATAETTEAVNPKFKRASRAAIRNELLDFSNCNSTLILAPGTITSFGYPNTKPPITTCTWNIGALGPYRVGVYFTDFSVYNAVYVYDEYGNIISSVTGNXRPFQVLGTSNVVTITHDSRFDAAYNYNGFSATILPY*

>Cre|FL81_07002

MPFVCEVPMTVADPTCSHNYNGYCYTGSTEMHLATINTTYXRAQSXCQSKNSDLVSIHSKQELDFIRAIYRNSGIQQIFVGAQAFLPDTFDWSDGSNWDFDYTDPLATSKGNCLVMDLSDRPNNGMWSQTDCQNVNFFLCKRKIGDATVATTVATTEAVNPKFKRASRAAIRNEFLDFSNCNSTLVLAPGTITSFGYPNTKPPITTCTWNIGTLGPYRVGVYFTDFSVYNAVYVYDEYGNIISSVTGNXRPXQVLGTSNVVTITHDSRFDAAYNYNGFSATILPY*

>Cre|FL81_07268

MKSSVLVFLLCISSALAYVEVAMKCPDGWDWFKRSRGGWCMKVFSGPVTQGEGETKCKAEGAVLAGVQNGDEVKWMGESLVKVAGGGSLWIGAKRSIPCIPIRGLTALCTPLTSFYWTDASTTGFLGFKKWLAGEPNNYGGNQGCVQLFSASGLMDDAACNIYSTGYVCGKSSTFVLLLCVVSALADCPAAWKTILNAQREESTSNGCEADWKFFKRPSGGWCMRVFPGYYEAQADAEKACKTVDATLSGLQNKKEGIYIQSAILAQVPQTSGSLWTGLQRTKKCMGQKLTATCNNLTSFEWTDNATTGTEGFLFQDAQPDNKNLDQNCALFLASKAASIVARGTYFAGTYEDVNCVTGFNSENIARKTFGYVCGKKASSLWNGGKRSKACIPIRGLTDVCTSLTTFYWTDASTTGCLGFKKWMVGEPNNAGQDCIQLFKNTGLMDDLGYFKK*

>Cre|FL81_07269

MKSSVLVFLLCISSALAYVEVAMNCTDGWDWFKRSRGGWCMKVFSEKINQGEAEAKCNEEGAVLAGVXNXDEVXWMAESLVKLVGKGNIWIGAKRSIPCRSVSGFTALCTDLTSFYWTDASTTGFLGFKKWLVGEPTNWGLYQHCIVLSSTARLMDDVACNIKATGYVCGKVASF*

>Cre|FL81_07448

MNFKFPTLFFLIFHHPKMPRHMPLTVFLLLNVLLIPVFSMFAPVNSGRSCIDAKSYFAVSEDESNEQNEESEDDFFHVENLEVQQDQQNNKNRLVSVVQSDVSQAVTCPKVANGNCEEGWKNFTRPSGEWCMKIFYENSVTQPAAKERCKAQGALLSGLQNQMESQFVFSTVTAHIYPETGSIWVGLERRAECRDVQWSWNCTQTTSFEWIDKSATGTDGLAWACNQPDNSRNRTQQCATLTASYQGSVLGFQTGQLDDVGCDFDYIKMNKKARDIKAYVCGKKPKA*

>Cre|FL81_07450

MSSFWSLLILLNFLVIAVSSGKTNGHCQKGWTKFTRPSGDWCIKIFYETLVTQPEAEAKCQAADATLSSFQNQVESLWVAATSVAHIYPKTGSIWIGAKRTKACLKSQLTEKCTRFNSFEWTDKSANGTDGFLWDAREPSNTRSIQDCLIMTIGSTGYVSGYDIQVGTLNDNKCDLKLNSKDPQQIQAMKSSVLVFLLCISSALTYVEVAMKCPDGWDWFKRSRGEAEAKCNEEGAVLAGVLNQDEVNWMAESLVKVSGGGSLWTGAKRSSACIPIRELTAF*

>Cre|FL81_07595

MLRQFIACLALLILPQLTLATAGTPICTNGFTLINGKCWRFFATGASHRSAERTCMNYGATLVTVKNAIDNRAVQTIVGTSTYSIWMGLYCFGNDVTKCLWDDASGSSELYDNFLSAYPQIETGKCVVYSLQSSMVGRWYSADCQNETRAFVCELPTSYAEHCGNLVSIRSANENRYINSLFLQSGTGSILIGATKTSKNSYSWFDGSLWSYNNFDTTALQTGNCVAMSFGTSNNVSAGTWYTVDCGASYGYMCKRPAGVQCPANPPAVTVTPVPSNPSYCNSTLLMAPGTITSPNYPRYYDNNVLCTYQLTTLGAYNILLKFTSFVTEAKFDYVTVYDGDNTNSYLFGNYSGPVPAFSLVSTGNSMLVTFRSDKTNNYQGFTASFSSYVYHS*

>Cre|FL81_07599

MQLAVIPFFLHSFFYTSFAASTPVCTNGFTLINNKCLKLYTTLASYSVAEESCRNVGATLMTAKNANENQAFTTIVGSTVSLVWMGLYCMDSDPSKCLWDDTTGAAGMYSNFASGFPLVDIGKCVYYSVQEALAGKWISGDCDNDPKAYITVLTIKTDFVIPFIARGLHLFKHKRYVNMNTGILFQ*

>Cre|FL81_07600

MQLFIVAIFSFCIAFVSCSTPVCMHGFTLANNKCLKLYTNETTYKLAEQSCKTFGATLVTPKNLNDNRAITTFLGSSASLVWMGLYCFDSNPSKCLWDDASGSADSYNSFASGFPLVDIGKCVYFSAQGALAGKWLSGDCEKETRAYVCELPHTYADSCQFNYNGHCYTIHDSTTFVQAQSICEQECGNLASITSANENRYLQTLTNKLVMDSNLIGGMWPSLNVFNWIDGSPTSYNNIDRSATYNANCMAVSNSASLSVPPGYWYSVSCNSPRNFICKRPAGVKCSGTPPPVTMTPVPSNPSLCNSTLLLAPGVITSPNYPQNYANNQSCSYQLATLGSYQILLKFDGFITEATYDVVNVYDGDFTNKPLLGSYSGNLGSFHVDSTGNAMYVTFKSDRSNVAQGFSASFQTESFDHASVYDGDSTSSRCVLNWKHNVCHIPVGRE*

>Cre|FL81_07703

MRSIFVVLLLAAVVQTQFNFHGMSRSFFSTDEEFPTQLQNFEGRTQTEIRTLKEKVEKLEKLIEGLQSILMKEWNQTESGSKYRLFEERKNWDNAEKHCQGFGAHLAIIDNEAKNTFVTNLINSSETSDFAWIGMKTKTTTQTSTPFTNFDSESPIDGCAVMDSKGVWAIRSCIQLRPFVCQIIRNDYSILFCFFLALWITQIETRKVTQKPKPPPPPPPPPKKDISWVRSKIDSAKEKYEAGKQKIKSKFSFGSKSDDTKPKETFAQKTTSRKPDLHLKTTKKYGWNSNVQANYAVPNTMFTTKKPLFDRVKEKVKGSEKYVGKAVGWAKKDLGIGVEGPKKPSKILTYGKKAAEYVFKKKSKKTAIAAYSTGRSNSNDYSRDFGNSGSYSGGGNSEVSSDLHQKVEYLKNRLEIMQNTIKGTWNTSEAGTKYKLFEEKKTWNDAQVAQKRESQLRHGFYSYIVKSWEVISHIWTANQRITSRNFCCILKISQWFGLDLEQKLELNQYPHLKINSQISHIWMVVELLIAMGHGAFHHVPWNSRIFVKLFVSMFWSRYHEIL*

>Cre|FL81_07917

MTLETIYRRIGKFGLADFEFQKPAFTETIAVSVALVLIAIYVYVSFFIYSGVFGSPIPQNSDVYADPLCPGNIKNLWLDVVVRNTISQVFGAVSNIGPTKWPADPRSTCVGVVTYDNSSTVNAEVDASKSFSDLYNVIQSSLVAVDTTNTSYLSSGLLAAEKAFKDSRQRTYRYKFQQVVIAFAADYQGSGTIKDAKPVANRLKNNGVSIITVSCTNDPNASNEIQSLASPGFNFIDEMNTAKLVKQLTNALLSVNCFCPSDYTQYRADYHDPTSTQYGICIKGYIETGGIIDPYQHAIDWCEYEATNGILVNEFTKQKHDFITYYMNDAFGVNKPQEYYIGLRYLKNQWVWEQPTGQERIPLDPNGWTNWAPGYPQANSTGQVIADQPWTKGSSEFVWAPPQTDDFYFVCQAVASSTENFTGQL*

>Cre|FL81_08198

MSQKITACVPSRNPLKSIAFQALFESKMVMNLLIIWSLLVYTNAQNFDKSFKEMCEFLDGKDSYRQRENISQPMEGDKCTVVFPISFDDKNSAQRYCEDNVPFHIHGSDFEREPGKLFCDAEASLICKDGWVQMFGRCYQITRQMMNHPDAVKHCGDKKSKIAFMHREALPFRINDYFSGVSQVWLNASEAITNDLIYNVDGGNLLLALDGYRYGLPNIALARVETNEKAMALCEYTPPMNQAESNHLLRRYGEIYYPTLFTEDKAYVRSFSSLQRSSDRMRDHNYCKKVLMPFLQTDSAQSAYPTPEFLELLTKHREPTIIRTSVYSADSSLLNRVSSNCTTSTSKNYGFDRTNSNGSALFTTLTSANIWRKDEPKEQCDGASWSTGIVLSREKGEARLEAMSDARYAPIYCQTNFDTFDYGKCPDKWRPYYRKERGQLWCHRLAVNEKYEPIQESFDGAEKWCKLQGAAVTGFTNAEELALLDDIINKGIWITDNVKDQFRTWLGAKRRPQCDKVLKGKKGFIPEKSHACSRLRVFEWLNGVAQNPPDFEDHWVAPSEPNNWQKNTEGCIELLKGDEMKRWKVKDASKMLNDNDCSQQKYFICGKEAPIKSNSNV*

>Cre|FL81_08596

MRHIALLFAILPAILASASPYCTNGFSMVNNKCLRLFTSPMVHKDAVATCSTQSVCESLCGNLVSIHSGNEMRYIKNYYAARSTESIYIGAITVSGKYNSWTDGTAWDYNNIDNSQSWQATSNCMMVSLSSNGTQTNDAWYHTSCDTARPFMCKRKVGTECSSDYSTTTQDSTSVDPSGPPITPTCNSFLMSRGTFFSPGYPGNYYNNLNCSFTLATLGAYRIRLSFNSFQTESCCDRVKIYDGDSADSRLVAQLSGAQNTPLYHESTGNMMYVTFTTDGSVVQQGFTANFLSLI*

>Cre|FL81_08597

MWRHVFLLLALLPSIQATQTITCTNGFTLVGNKCLKLFTAPTSRRNASKTCAEYAGNLVAIQNQEENDALASFVGDGVRSVWLGLYCFGTSNCLWDDASGSNDGFSSFVYIYDIYKCVTYKVTGSWSGRWFRGDCSYDLRPFVCQIPTTYEDECALNYNGNCYLPSENFFSSQVIGIDSARQWCLNNCAGLVSIHSANELRLIQNYYKTKDHESILVGAVTNSGKSFLWTDGSTWDYGSVVGSEQSSGTCVKLALKTSGSRVKGSFYVTKCEESNYYMCKRPAGISCVGEPAPSGETPPVTPNCNEFLMNSGSFSSPNYPETYSDGESCTYGLGTLGSQRIRVTFSNIVVKDHEDHIRIYDGDSVESPLAMIITGRHSEPVYYESSSNRVFVTYNTADNLDAEFARTGFKADFVTIENEALASFVGDGVSSVWLGLYCFEGGTRNCLWDDASGSIEGFSNFVYNSNAYNIYKCATYKVTGSWRGRWFCGDCSYDLRPFVCQIPTTYEDDCALNYNGSCYLPSENFTSPQTIGIDSARQWCLTNCADLVSIHSANELRLIQNYYKTKDHESILVGAVTNSGKSFLWTDGSTWDLGHFVGSGQSFGTCVKLALKTSGSRVKGSLYVTKCEESNYYMCKRPAGISCVGEPVAIVEALPVTSHCNESLMNSGSFSSPNYPETYSDGENCTYDLGTLRSDRIRVTFSNITVKDREDYIRIYDGDSVESPLALIITGNHSETVYFESSSNRLFVTFNSVTNNDTEPARTGFKANFVTIV*

>Cre|FL81_08599

MRHILLLLALLSYIPVTQSITCTNDFTLVGNKCLRLFNTPTSRTNASRTCAEYAGNLVTIRNHDDNFLLSAFVGNATDDDIWIGLYCFDKNVTNCLWDGGQGSAEVYNNFVSSRSEGNPFFGFYKCVYYKVGDPWNGHWLSADCKWLRYSFVCEIPTTFDDDCALNFNGSCYIPSEELINSTQSLGLDSVQKICQENDGDLISIHSANENRFILNYYEGLGLDSVLLGAVVTKHFFWMDDSHWDFSNIDHTNDEYGTCLKMALQNNITISKGSWYITPCDGSHYFMCKRPAGNSDLIHVPKVAKTPPSQCNKVLLMSGTFSSPTYPENCMYTLKTLDGQRIQITFQKFHVTNGYIEIVEEYQGAEVKQKFSGTNKNLRYKSLTNELKVLFITEDLENMDSEFVAKFFSVFENE*

>Cre|FL81_08602

MHSFQSFSITFLSFLVVFSVSDPICNNGFTLVNSAKCLRLFTGPVRHRAAEAIVDLGGCVYSAAAGTLAGQWISAECEDVEMAYVCEIPTTKTDPCAHNYNGYCYLMSHENSTFPTMPFTSAEDSCHQNCAELVSIHSRRELTYIQSLYSTPNISAVLIGALTTSPLTPYWVDQSRWDYGHVSPRSGSTGSCFQMAVKTDGTWYQVDCKTSQYFLCKRPTGVTCNSTPAPPVIVTPAPTNPTGCNSTSLFDSGRITSPNYPSAYPIPSLCNYRLSTLGAYRIGLYFTGVSTYTNYGYVYVYDSNGARLAALTGSAGANTYYSTANTMTVTFTSGTYGSGYSGFAANFLSF*

>Cre|FL81_09841

MHSLIFLFSLLALLSDGLLAAVVCPGEEKLDPSGRYCYVVHKGALSFHDAEKACYDYGGYHLATVPTMIDNRFLYNLSSHSNVYANYFWLGLTDMTADGTWQWIDGSDLNFVNWAPDSAQGYCGAMRESDGRWQAQDCAKAYPFFCYGSIGDPNTDTNALNFYNMERDFIRAVTDSLFANPSFNGNVCTFYMSVSFYGYTKYDQQFDHSAAWSQRQFDNFAITGAQRFQWSPSMDDIGYTTLVFLTARKDFTNVPSLFNPFPKFDEVVVVSLNGAQMPGIPDGVQNVPVSNKFTNVDINNLVSALKCH*

>Cre|FL81_10016

MLTVLSVLFSLLCVPVTSFLPTFPPNNKNPCERFGLDSEFFDLNTWFKKRKQLFNWAAEIRCLRDIINKCEDGEEKSRGGRSLQDPYSNNYPTEASTTDYGNPYSYPRPYSRTYYRHSISPIRPPIPQPITTAKPAGECSCQQELEKLEAKFEKKLYEVKMKAAYETETAVGELRKQFEQDLRSYERITTKDVVEIKRTLDYMQAPRVMNNDVEYFLIQREESWYTASEKCIGYGAHLASVHSRLDNGLLAKLIPANETVWIGVNDIQKEN*

>Cre|FL81_10194

MGQTQPVWIGLTCSLSGSPSSCYWADDSGSAYSYSNFASGNPFVDVGQKVYMLISGGSAGKWVSGDGDLISISSRSICESTCGDLVSIHSSAENNHILWIYNQYPSNYYSGDYLRIGGFSDGVGKYWLDGTAWDYSNLEYFNPQIGSCMTMAVKDDIVPRGTWMSNNCAVQTGFVCKRKMGATC*

>Cre|FL81_10290

MYAELQSRRGGTINTDSASGSSSPGINEESTTVEIINTNPPSEFGLVDKQRHFGILRYTVTNRFRKLMLIALVNLVVFAAFFLFVFFLIVHLDKKHEDILVSSTNNCQSSYDVYSYARLDVNSDLKNHGYSTSLDDIEDDIKSDLPDPKESYPSYYFGSNILTCIRTMFTSDMTLCGAKMYFLVKRLPNDTDVSDLVFLLRKFHISITFVVSEKPSGGMNQQVLYTLATQTNGFCVFAEDHKFHDTPAWVPSFWPLYLVYSANAGVRSTGSLTLPVFNAPLLGEYHICMTLQDHGAMQKFRMVHLKWNNSESSSSGFFKETLESHAVKFGETTYIIKGPYTLDAVPYNMTLGFEYSDDEINILQIRIYSVSAVDCWVPYSIMTSQIPESYDEPSHKPHSSGIVSIIMKNRLLILIVFLPRSHSGYLETTTPSKSSFTTTIPFGHIDCTPNQTSTFFFAYSNDLTPDQVLNTWHTLRNNSQGSYDIYSYARFDVNSDFKNHAYSSSIENIEDDIKSDLPDPKESYPRYYFGSNILTCIRDMFMSDINLCGAKMYFLVKRLPNDTDVSDLVDLLGTFHISVTFVVSEKPSGGMNQQVLYTLATKTNGICIFAEDYMLQETPTWLPSIWPLYLVYSVNAEVKSTGSLTLPVFNSSLAGDYHICMTLQDHGTIDILGLIDKWNCRSLGKIQNGAFDMGNTTYIIKGPYTLNAVPYNMTLGFEYSDYEINILLIRIYNGFDSVGNNCWKYVHKFMDQSGADTECLQYNGASLIKIYDKYENDHLQSYLILKNANDRVWLGMTCNESTVAASCNWSYDRGTAAEYNNFGNNYPNVTKGPCVYIIKYNKQWFSSDCEQRRAVMCEVPQTSEDPCPYNYNHNCYFPSDVNASFADAVRSCSNMCADLVSIHSALENRYLITIPDIQARWWLGGVGPAPDIIVWSDFSPVTYSTFQFYHTQISGCLYTDINYNTKVDRLWFTDYCYKTARFFCKRPTETPPIASVEKSPDRVFKYCKIATFVLLGLLILGVVVFLILFFVVPKHGDQSASSTVVPSISTIGSTTVTKLATTTKPSSELYDSSTVPLVTNVTAEHSTTSKHLPTNGPITNTTASGLSTTSPSNRCSLITNTTFLFAYSNDLQSSLIQKALGDIKTFIVNPKITTFANNRFDTIKEDPIHFHGSKDNFTDSVNALLPDSKLKLPNTKDGSNVLNVIEKFLEQPTCGAIVYILMKRLPDTVDVTDLIQELRLNHISVFPVIDSAYIGSKDQSIMCKLAHTTNGFCDYQIASRLENEVYETMWIVSRVHVFVSQSYQVSGKGSIKVPSFVRPPQGNENGSMGLVVTYQNHARDTNFKALNLTITDDQNKVVLTGQTKSTNGNALLKHPVLKNNVTYQIGIDYEYARTDWFEKIDVRMYSISVPSLYKAKNEANQGLEVIELEEEVPENDADELEEEELVRYCFWIWILLLDIAFGKCVFYVLEVGEESGQWGNGLCKDDRYPFICEFPPTFDDTCGFNYNNHCYTKLNFQFTFTDAQDACQRYCSILLSIHSELKNRLVVSMFNTTGSILLAGVAPSENLVIWADRTAQDYNNLKSFDMELSCIHMDFQSGDWSSALCNKASWIVCKRRVGINAFGNRPSVSRIVDVPTYNDDPTTEMEPINKQNYFTLLNQVGDSQFQKVILIGLLNIVIIGVFVAILFSLFFLNHKDPHASSPTKSSTSSYPTTNTKPTVTTTTSTQNATDCTPTTPATLLFAYSNDIFSGHIQDACDWITEYFTSLKIIYFANIRFDTTTDDPIYFHANKSNFTVSVNTYLPDSSLSFPSQRTESNVLNVIRKFLSNQEYPLCGSILYILIKRLPNTKDVSDLIQQLQQHHIFVYTVTDTNSFGGTDQGLMCEITHATNGFCDFQSTDMRNEYYQTAYEISRTYQFVSQSYQVSGQGVINVTSFVRPKQGNQAGTMSLVITFQNHGTDDNLKSVSFSILDDEDKVVKSDHYKSTNGNSFLEHPVLKNDVTYKIVINYEYGEQRKEIIEVRQQHHLALFHRVANSQLQKIVLIGLLNVIILSIFAGVLLVLVFTEDKPHINNKEGSSTTAAQSKSTDVAQLTSTTVKSFSPSYPDACTPRSNSTFLFAYSNDLPSDLIDKARDWIVNLLPNRRPVCYANRRFDSTDDIIHYHNSNQQVKSSLSQSVKAHMPNSTLKFPSRANGSDVLNLIRSFLTNEECPLCGAVVYIIMKRWPNYEDPSELIQQLRQHHVFVYTNTDTTPSGGIDPNFMCKITHATNGFCGLQSSAKMDDDIFHTVRLISRPFQIVSESYQVSGKGSKRTPKFQIPLQEYADDFISMVIVFQNHILDDNYKYINYTFLNDNQEVVIAHYLKPEQSNTDIEQRRLAVGVTYQVILDYDYGTAQEDLMEIRLSSSKTSPKSRSLLIKIGQPQFALHFCKTYKMINRISQIYAERQARKRQFQNDISRASVSKDSRYQSTTTNTRERTIVEVANQEPRPDFEPVERQRHFGILRYNMKNRFYKVMLIGLMNVILIVIFFLLMFFFAFKSKCSSDEGSTTVSPREHFITKAIGSGALLLTCPDGYDLVGKNCLQLYTTLLTWSDAKDRCHLSSSYLLTIKTKQEDKEFRAYMQEHHQYNRTWLGLTCTSSKLTSCEWEDQPFPYSAFANGSPNGGCMFYNVSDTLGTPWVSGDCDEKLVSVCKTRPTATCKNNFGSYCYYYYSQEASFSEAADFCKLQCGNLVSVLSEDENNFLLSMDEIKRGLIVSEDKIMWADGSAMLYNNIVEYRKQDLCLIMRMGSNAGIWTSTTCSKTYSYVCKVPAQ*

>Cre|FL81_10496

MARSAVFLAIVALAGGVAAQTCNTGATTCPTPAPCPAKTCVPSCDQGWTYFAPTDFCYKVFHGAKFNDAEAACVLLGAHLTSIHSLTENTFVNNIASCGIKESKYENLAWIGMHQENGKDWVWTDGTPTDYINWAPKQPDNPGKELCVQTAPDVSHDSWYENWNNLACNTVMRAYICKK*

>Cre|FL81_11286

MQLLLLLSLILSAFSITFDRGSDSSESCEDTSEYHPRPHRPRPHRPRPPPHKPRPPPTELTKCPDTWMLFRRPQGNWCVTLFHKSQTWQGADNMCKTYGGRLTGLQTGQERMRLAEAARRIVQPLTPRNASVWIGARRKPECPRAGVCQDQNSFYWTDGHTTGTAGFHWAASQPDGQVEGNFGVQSCARLAVFSGGSAASPRHGAYDDYTCEYLPSRVTLVACGKKATRY*

>Cre|FL81_11297

MSHLKLLLTISLLFLISTSSARDFGDDSSCESSEEHGGGHGGRPRPPRPPQPTPRPDDAHRPRCAQEWFTSYRPQGIWCIRVGIGKLDYNQAAAQCATYGGVLSGIQNDWERWLISNEANRQTLAYNIQYSGIWLGAQRNPGSNTFSWTDGHTTGVQGMVFGPGQPDNKNRDNRGPQNCLQLIALTPGFWNNPGSQVWVSYQSGQIDDYWCDQVEDPEQRMYACGKLGPRE*

>Cre|FL81_11313

MTKIIFLFFFFLFATVFAIFRPSDSDGSCEDESRHHGGHGHKHHHRDDSSDEGGYGGGGYQDPEPRCPSGWLKFNRPTGLWCVKVFSGVLTQPDAETACQAQGATLSGVQDENEITQITAQALPLLPSQSSFSIWIGATRTTACSPCTPLTSFQWTDGSTTGTSGFLWNLLQPDNNFGGTQSCVVLLASTSLVIRDQWTWNANRLDDESCVGERGGALRGVKAYVCGKRGE*

>Cre|FL81_11327

MVRGCGRGWHRFDRPSGGWCMRVFKGNVSQMEAESRCRREGAALSGLLNENEIGRVAGHALRTLRPLTSGSIWLGAKRTSQCSTSPISSTCTPLNSFLWTDGSTQGSSGFQWNTKQPDNNYAKTQQCVVLLASRSTVIQDQWTWNSNRLDDVACVNPGGSEQRAQKEMRTLLLLLALIIGSVVTQREYRGGRGDNGNSNNNNRGCESGWQRFNRPTGGYCIKVYRGEHTQPQAEARCQSVGAKLTGVVSQEEISWITKSALSLISQSSGSIWLGAKRTNTCNSSPLSSKCTSMNSFYWSDRITIGTSGLIWNTNQPDNSHAQSQQCVVLLAARSTVIQDKWTWYSNRLDDVACGLPSGDNNGPRAIRAYACGKGA*

>Cre|FL81_11543

IAVLITKIFGESRIGTSIPNQPKTTRLGLVTYNWNATIQAGLDKFQSQQDVFENIFNDLNSVSSSSESYLANGLVAAENVLARGPNRGNNYQKVIVLFAASYSSHSNPIAIADRLKQAGITIITMGYNNVGDPNFYQNLGNIASPNKSFTERSLSQIGDIQGALLDSNCFCPPNWLQYRTPFTKLGECISPVSLQAVWNAARLSCRNQRPNAYMVNEYSQAKHDFVLQLVKNTSGFQQPYTYFIGLAYSSSGNWQWDQPNGWPQPVLQNWTNWDHGYPVSSSSMGAVQNQQKGEGAVWRNVGVWNSAAPYVCEVAACDTDNYCVDEDS*

>Cre|FL81_11778

MIFHLILVYFLISFVQAQNKCINNNDDRYIDGACFTFFNIPLNFTAAQEYCQHHAPSAWSILAKETSDTQAIWLAKIAVTEFEMNSGYFWIGVYRETTYDNFKTMDGFYLRYQRFAVMNPSMNYVAARTSDGKWYTLPEERRLPFVCSYYPTNVVQAAANAPRVLKSLKELFYL*

>Cre|FL81_11779

MTTQSFFLLPVFLLSFVNAQCYNAEDSAIGDLCYSFPRFQMNFQDAQNYCHSQNQNLAVIHTSTQSSFLATIVRTRSNTTNAKFWIGLSRSSANSSYVWDDRTPLSWSNFNPNGRYVVESTANAKWQTETASSLLDFVCSYDPFTTGFYTTEEPMTTTGETMASVTCLFMVDLQSAGIDQSAITTYRSFYNFAQLVGSKLNDASDFSGYLDTFGYSSYLGNHDMFSSLNYDDFKNIPFPIDGTDDDIDMDLKDVDSTLANAVWSPPSQDQTCLIFFSAAPVAEFGGTTIKSSYNSFTTVVGVLLGGATSIPGLTNSISVSSMTDADAQASHMKHLYFLSTLLLAIATTAQNCADSSDHLIKGRCFKLVNKQLSYQDARNWCRYSNPVGFTYLATVADQYMDNFLASYARTTFNSNEGNFWIGLTRTSGKWQWEDETPVAWTNFRVQNSQNYAAESITDGKWTGYSPDTKMNFVCTYWPGVTPGPTPTPEVGTSDTPTWTTVSEAYPTPTGIFLMLLKQLVLTFTISIGLASSACFDNGDKEIQGGCYKFVPQKLTWDDARNWCHYQNPVTATYARSAFGTDDGNFWIGLSRSSNGSWTWDNGFPVGWTNFGPQNGQNYGAESIVNAKWNAYGSSDKYFFVCSYDPAAPPTFPPPQSTTTSSPVAVSTQAVPTTTGKKS*

>Cre|FL81_12618

MSSSLTSILLFLIFLIPSTSAKTPCSPSWLHIAHLDSCFLSAPQPAEFSEAQDYCSQMNSSLVVINSEDEGSIVREFFARENPSFFNWIGMRWDERRAEFEWVDGKKRNYTYFLPDEPGVSGECIAWVLDDNLDGWQAISCHYSQFFMCQKPAEGIVTTWHRDDEGVITSPNYPEPYENLEYDTHIIKSEPGTRILMYFENVETEQNCDVITVSDDYGISGRTLFSRPLPLKVFSSNSYGTVTSNNYPTSPDSFLIQYYLIQCPMEFHVALTAKAMQLDKNDRVKVHNGADEKAKKLRIFRQFSPQSADAIKSTQNSMFISYDTGEQYMPTNHWAFEYQCVPDGNLGAEIEI*

>Cre|FL81_12734

MVLFPLLFLLFFRRVAHTLTFPPSSSSTTTVLFCLQPFVPENAHVIFNEPGPYARDTVAKYSCALGFDLIGSEERTCLSDGSWSDEPPICAIDVAFNKPVTQSSGNVAIALGGNMCTMTNDESKSFWEVDLLGDYSIRSLSMRLGTKSSPIVSVEAIETGGAQCIVDSSLFTINTTTSISCLYDNISRLRITATRRLHLCQVNVYAVNAVFGGMCYAASRDEQTDWLGAQRKCLDRGSTLPLRIDDSTRRGLRASLSASSSAKAFYWIGASSSMTEWRWVDGEGVGDSADWPGQPSPIPSASEAVLLARPLDWKWVPASQTAWNSFLCQSKPKFCTSPGVGEATKVSFSSHSYAIGTLCFYSCDAGYDLHGIRQRELNTNAQTVGTLPTPRRPPTDHSVEFVNPTEHGAVLSQLVNLSIVEDRRLSRMEELTSNRRLTNRWPITQIKGSTSHEQQNGGGSDSTIAMAVLASVIVAILCYGITKFAKSTNHGANGDLSISRDKLNASNAAIYATPSIPPQRPDSVIYYAPTVAHMEVPPHLLQLQQLPNGNIHVTLPIGRQMGRPSLPSSMLFNSSMPPPSPTPSQILYSFDHEPIYDTPPDTHVYQS*

>Cre|FL81_12970

MTYFYFISLFITSLLLLLPTFGLSIPPTTFPNCPKGWHHDEPGRTCYHLARRKMRLPEAHAYCHKLIPDGSAHVLRVECGGENDYISGLVKVHSDKVWIDARARFDIVDGSAGLFGPGFVYRWPNGKMVRYSNWADGNGLEEIGTSDKCVFIKNDGHWINANCSSTAAVICEKKLHRPYSKFCPKHWVYNKETQSCYRTISKTNMTILEADNKCFDYGFEHRQDAMLTSIQSESENQFVMNLAKERDANFEFIYLGGYGRSRNGNKWHWMDGSEFNYLNWDRGMPFGRRALAVLVMNKRGKWINHYADKILSQYNAVAVCKFKS*

>Cre|FL81_13197

MIRYHSLLLLILPVFVNAQCDLGSVYNSDKNVCFTFYNASVDFKTAESICTISSGHLASVHNIIDNNYLAKQAQQYISANGIIWLGAKSTSPNVTDPNSWNWSDGTPFDYQNYQSGEPSSLQTAACMQFSAATAKWKTASCINYAPFICEYQPDNFPVTCPPTIIKSCPSGYYYLQETQYCYKLVIARGNFDDARSGCWSMGAELVSILSPTENGFIHDISQTGHDVWNKEQTNNIYIGLIYQNGHWQWTDGSSAYYLNWASGEPNFMKKEHWTTYMPDGHSDKYNPYGDEWNNIENDQQRVFPIPFVRFCRHSASQKCVAVDVWLGQHSDKRLFPMKCLNDTCPEGTFCDEDGKCWEVIKFGDGSRYYEMKLIDGKWK*

>Cre|FL81_13198

MILPFFLLSLPFLVNAQCGVGAIYNEKNNLCYRLYKAAVDFNTAEMICSTLNGHLASVHNLDDNNFLTQQASKLIYDNGPVWLGAQTSSPNVRDPNNWKWTDETPFDYQNYRIGQPSSLGTSACMQFLTSDGTWLTATCTDEFPFICASEPKKITTVSGVTCPPTKVYRCPSRYVWFQYTQSCYRVFIGSYSFESANQYCKNDGAELASVHSYTENQFLAQISTTGLGETSTGNNIWIGLIKNGTHWQWTDGSPVDFVNWESSYPQGYLATAMISDGDQRWYNTIYPERGFICKRPAIH*

>Cre|FL81_13257

MLLYSLLSFYFISITVDAYCEYGTEYKNATGCFQFFRTPLNFTNAVRFCRVNMKSTLVRPVSFIRNQQLQQAAMKLGIEEYWIGASNVDNDWEWLDGSMLTYSNFDVGSGYPKKTESQIGAVSMGSLSGLWYTKLDAMMLPFVCEFPISTQFDNGVLYRAPKLQSLIFPSAGTKAPMLLVESVDQSSLFNKIGPKKNEMETGEKVYLKPIDMSAAGMSGFSGQPIVIMNTFKRKVKVKPVVVSQTETEMARSLKEKEEIEDSTNAKSVLNVGGNAAARNGTSMTEEMSSNSKAKREREENESIRSKTIQISRG*

>Cre|FL81_13514

MLLTLSLVLLFAVPTSAQLGNGTCDEGWKWFHRTGGGWCMKLFTGSYSWFHAAGLCQTQGARLSGIGGDTGVNPQQRIEQQWIKDTLLNLTSHITDVQPNVWIGARRKPECMYKQLNVAALCIPVLAFEWTDGKTIGVNGFQWAPGEPNNVGGIEDCGALRTDMNKSNDFDCYSTHNTRGYVCGKPQIDP*

>Cre|FL81_14185

MILSNNYFLLTLVVFIICIIHVESSCPVGYDQVNSKCITITPQRFTHHKALLTCKEKNGHLVFVQNAIDNTAIVNYASNITSPMWIGAICKVNKQPKECTWDDGSTLDYSNFLPGYPVTNIGTCVYIDSPNQPLKGRWIILSVSAEKDPNAPSECLSKCLTPLAKLQRSFSYVFNNFEKVCDLLEDGAFCSRKCNQEDQTKFYQYTTFYRIHCIDYEEDIQEHLTCIAKASEDADLVCKDKCKQAHKVEKTASKETKMKKECLTLECSTLCYFDELAESCPEARNVLLKINIGQVHSMASGVHPITMEKMLPECRNLHNTEYMRAKLLASSSSLLMDHTPSEMETEEKPVITA*

>Cre|FL81_14186

MYWTLLLLTFLVSVSADTCPNGFTLLDATQKCVKLITTAAKHADASASCSQYGGHLISVHNAIDNRAYLALASASTTPYWLGIKCSLSGSPQSCLWDDQSGNAGTYNGFANGYPLVEVGSCVYSPTQGSFAGKWLSGDCDTMVLNFICEASTNTPVTDTCSFQYNGNCYFPTLSSLSEQEARFACQQECADLVSIHSVEENNYVQALFTNNAPSYIRIGAMTTDQNVNTWTDGTNWDYSNVGYSDTKLGYCWSMSLTNDIVSAGKWISSKCDTPIPFVCKRKVGQQCGSTAGPTLAPGQCNSPQFYDNSGTFYSPSWPYSYIGQLTPCSYILDTPVGSLAEIRFPVMNLDNQASIAIYSRIEDTTPLVVLQGNNAGNQWYTSTTNTMRVVFRPCVNNCPTDGGVYRWQANFQPSNQVTQPPPVTVTPNPNNPSGCNSTILVTPGYITSPNYPGLYPNFLECFYHLSTNGGYRIKLDFGAVDTEQCCDNIVVRDGPLLGSPVLGVVSGSWPAHAKIFQSTSNSMFVSFTTDASGQGMGFSATFNAY*

>Cre|FL81_14266

MVFAVIALVVSAILIPEALADPCGDSNWRYFPQTNSCYKLIDENLPWTIAEFKCLFQGAHHVSIDSPEENQFVHELSHWSEIWTGAAFFGKDMHYVNSDGSRYGNFENWKDGRKPPMNRARRCIKMDANGEWFQSCCKKKTYTICEKKAAYSASSYSGANNSVNGFRFMRHRS*

>Cre|FL81_14806

MIFFSIVCYFLATFGVVDGDCAPGDVKNTQENCVHVENLASTWQEAENFCVAHNGHLASVHNAFDMTSLRKVAGICTNFWLGGQCQAGSNCKWVDGTDFDYKNFRNGNQGTDNCVVADTKSGTWSTQPCTATSCIACEIKGAMQDCQDWMKAGYTDSGKYTILVNGKETEVWCDMQTYGGGWVLFQNRLDDSESYWDRKWDEYKNGFGDTDENVNFWLGNEALYTLTNDKHVTLRVEMYGDRTPNSKNATDFWFGHYFEFKVGPETQNYPLVNLEMDWAHPIGNASTAWYDLTCSIGSPFSTVDNIHDPVKECVTKFQMGGWWLKNCALSTLNGAYTPKDWNNGYGMFWIWDGSDTILHPRKTRMLLRNTVV*

>Cre|FL81_15127

MTTLRFIIFTLFVYGVTSDNTTDGPYILALSDEQPHQRLQFYNWDHKDLGTNAFEDLPPLAEQPTPLPINQTEKCPDGWVRFSDSCYFYETELLGFAKAERKCYDKQATLFVANSFEEWDLVRTRTEKSHFSWIGLVRFSHFERSEQLPRWQTTGSINPSKLNWIIKPFNPIANGWSSIANCAATYQSPSPVESTSYTYFYPCTLLLNSICERNSTIVNARN*

>Cre|FL81_15604

MSKNAMTQINIENDKSDSRERRRRLGRKRRKRRRLCDQIGEDKWIKRVIMRERKERITDCLMVFAPLPIGFIVANWLCCLLVHSANCNGKRTVEGEEDDEEAEAVTSDRSGKITELFSDLIIEEMKFTILVLCVCLLKIANSLNLNDIKKVRTNPTTIATTTTMKPTTTTKTTTTTTVYTSTTKPVEHSVVDPGPMLIECPDACPTGWQYYNSNCYKKFDSGVTYSQAVSACSGLGAQLVTIDNFDENDALRKAFDTNTLFYESQETWIGLKFVSGSWTWSGGSEASYINWAPTQPASGQCVQMITDALINETYKYQRGGWKTYDCSKISAIEMKCLTAVFCLQLLTFSSALDLNDIKKMRTDPTTLATTPTTTTPTTTTTKPTTTTKTTTTTPTTTTVTTTTTKPVEFSIVDPGAIMIDCPDACPKAFDTNALVDESRETWIGLKASVTGGSWIWTDGSTASYTNWAPTQPSSASQCVQMITDALSDATYLYQRGGWKTYDCSKTSASYICEQSASEAFDTNALVDEPKETWIGLRSTSGSWKWTDGSSASFTNWAQTQPSSSQCVQMITDALSNATYQYQRGGWKTYDCSKTSASYICQRWATI*

>Cre|FL81_16165

MDSETENVLFFSQKLHMNLYSLTTFFLLPAYLSAGPSCPAGFSLLNQNKCIKVFTSSAKRSDATTQCISLGGTLVTIKNAIDNRAISTIAASAGLQNIWIGIYCYNFHNVTCYHDDSTGVISYNSFRPGYPKEAYDIGQSVYMQTNGAEWHTGYRDQMSLPFLCELPTTVSDPTCTHNYNGYCYLPSHEIPGIDSSTTYSKAQAICKANSANLASIHSKQEIDYIKSIYTNPNEISQITLGAEARQLHVFNWVDGSNFDYNYFDPLVNSTGNCLQMDLSYRYDRGLWSEISCQSVNNFLCKRKIGATEQIMEPSQPINHFDLSDPSNCNTTLLMAPGVFTSFGYGTSPLPNTYCYWRLATVGAYKVGIYFTDFSVWFNMNIMDQYGETIARSTGNLQPFSVLASTAIATVTHLQTDDTPSYNRHGFRAVVLPY*

>Cre|FL81_16206

MFLLGLLLLNLFGNLSAAGTGPTCPDGFTLLNDSKCVKLYETAMTYAKAVKTCRSIIKGDIVSVHKNTDNQALLNLINSHHSVRPIWLGLTCVTSNPNSCSWDDNSGAASYYNNFAKSNPNLSAGKNVYMLVSGSSTGKWISADGNLVSLSFVCETPSSLVPDDESCSPASPTTFLFAYSNDLNPTDVLEVWSHFDQHREEISNKSVVFANVRFDLRKAEDIFYHTNFSDVMDSVEAHLPDSDLGFTDVGTGSDIISIIQKFMNDGQKAPICGSAMLILLKRYTNEQNIDDIVAKLRKHHIYIYVVTHEAPSGGLYSQTMYNVATRTNGYCSFGIDQNFLYAATNGGAYYSHYLFYSTNIPVSGKNGTVALPLMTVPDLETDYLIMTIQDHGPLTSFIRQEIDWNAVGTDLSGGEAENIWDFGWVKGNGTFYELSWQPRPNYVYNMTYSFAFTDRRSQVLQFRAFTEDENVINTWIPYDN*

>Cre|FL81_16497

MKMLMKILIIFGLLASITNAQVDRDWSFQRMCEFWDGGSSYRPRPNGFNSIEGDKCTFEYPVATDSRESAKRYCEENVPYHINDVTAGERTKCSAEATLICKNEWIQLFGRCYKMTKELMTRDNAVEHCKTQKXDATIAFLHRETLPFRIYDYFTRVSRLWIDASEAITXDLIDENVNGNLLLAIDGYMYNLPNVALTRVDSSETAMVLCEYTPPMNRAESNXLLKKYGEIYYPTVSTSEGAXIRTTXSLNRIDEDLFKDNRYCSRVMNPFIXNSNARSAIPTREFLDEVNKVQNGLIIRTAAFSKNSRKDERIGATCSAKKGATHRVLWRGSDGNDVSVPVDKSLWRSDEPNEICDAGSWSSALVSGRDGSPGLEAMSDARYAPIYCQNTVDSYSYGDCPAGFTEYYRKSIGQKFCHRFFSEKKTQPDAEAHCQTFGAHLTGYTDSEELKLIGTLNVLGIWETLIGGRRRSECITNGAKNEPGYSREESSPCSRKRVYEWKNGVAPNPPTIENDWSFDYEPNYVAYKEECLTIVRHKGVSLNDNECDWPFPFVCGMEAPIVKLLSS*

>Cre|FL81_16649

MHCSLLLILIIPVIVNAQCELGSVYNSDKNVCFTFYNASVDFQTAESICTVSSGHLASVHNIIDNNYLAKQAQQYISSNGMIWLGAKSISQNVTDWNWSDGTPFDWQYWQSGEPSSLETTACMQFSAATSKWRTASCINYAPFICEYPPGEVAVTCPPSIIKSCPSEYYYLEETQKCYKVVIDRGNYDNARSGCLNDGAELVSIHSYTENSFIHDISQTGHDVWYDTNEDQTNDIYIGLIYNGYWRWTDGTSFDYSSWASGEPNNMNREFWTTYMPDAHTDNLRPNYNPNGLQWNNVINEQQRGYICQRSMIS*

>Cre|FL81_17156

MADARATTCYLNGLSLRTINEVEKILRSLPREGDPCEIQCSQPSIGSLKIHEDPKPLVLSPPTKQEXPKKSKGKQTISEYSCVSLKSEEMKKREVIASSVSGTGSKIQCKVLTDQIQVNPIMSCNGVVLSLDVEVTIPDRQEKKKMRLEIECPDFMPARIKIGNKWKSIIIENSLLLNNALESCRTYTDGTLVSIHNAIDNRALINLAVAQQTVRPIWIGLTCLTTSCVWLDQSQTDYTNFVSGNPQPDVGINVYMLTSGNSAGKWVSADGSLVSLNYICEVPAIIVPDLCSNPFNDYCYTFNQDVRNEINARIYCQKDCGDLVSIHSEKENRHVLSLFNFTSPPPQVRIGAGTDGIGKYWVDGTVFDYSNFGYFNVDIGKCSTMQIPYGVLDAGQWLSNNCDDELPFVCKRLNGITSCPPSPTPTYNPNMCYGSHFFTGNGTIYSPNYPESYYGQFYPCTFIFTVPSGNIAQVMFTLLNIDDQSKISLYSGIEGTEPIIELTGQFFSPPPFNSSTNLVEIPSLISDDMESCRNLTFGSLVTIDNAIDNRALINLATAQNTTRPIWIGLECYGFPCYWLDQGGTNYSNFASGLGVNDKRAEDRKQWKGANKLGKILEKVRDELWENEEFRRDRELIEKEDEEKRCESLEKS*

>Cre|FL81_17187

MFNLTHSRTSADGICFLHNGATLVSIKSANENRALVDFVKNEHLDTVWTGLLCNNGTNITSCIWDIQAGTAARYSNFEAGYPDVTYGGCVYFTASGKAIGQWGSAQCSQSMAFVCELPATVLDETCIFNYNGNCYTRFDNYWNFSGAQRECESRCSNLVSIHSANEMRFIQTIYSDVQSAFIRIGGIAMSQDFLIWTDGSPGDFDNIQYFQPGSCIIMATGTSKSGYWYTHPCDQGYVMSFILDHWKHLLIGGISVLVLIVSVFLITFFAVRGSFHCEKENVMITTTASNIITETTNFKVKTTTTSTRTPFKATSTTLKPTTTATPQNLTCTNDFTMVHGKCWRWFNENSTRADADQKCKWHNGATLVSIRNYEENQALADFIKEKNIDSLWIGLYCTEWTNIDSCIWDIQAGSAANYNSTCDFNYNSHCYSPFPYRKYVTDAQNFCVSRCSNLVSIHSANENRFIQSMYPDVPYMYTMIGALAPSLDYIIWIDGSESDYNNMRQTTESPRGSCVAMATGSVDTGYWFVFPCTEYWYYTLLCKQDSASIPGDRDASKTRFQNIITFVMDHWKPLLIGGISILVLIVAVFLITFFAVRGSLDCESDYTSSTFSSSIGTESSVRTTASTAIPSTVSTQKQFPTSLNLSSTAVYSTTPDLETSVQLFDLVPQVPICTNNFTMVEGKCWKLFNHGEIRSDADKICKTYSGSTLVSIKNYDENKLLIDFVKDSHIDTLWTGLFCNNGTNSTSCFWDVQAGSAEQYSNFGTGYPNLNYGGCVYFTASGNTIGQWGSAQCSQNMPFVCELPPTIIESGCKYIYNNNCYIRTDYGYTTTQAQRYCVSNCANLVSIHSGNENRFIQAMYDMSYAYILIGALAPSRDYLIWLDGSPTDYNNLNVINPGVCVLMSLSNGSPAIWYTKECGYPSWFLCKRPVGAKC*

>Cre|FL81_17188

MEEKESVIKTHFEKFITFIRDHWKLISIGGVIILLLMISVFLITFFAVRGSYESGVHFGVREILIMHVSSLHNDSLDNGNDDYLEFPTTTVKTAKPSSTTTLKSLTNPVFTTTSAPLTCTNNFTMVNGKCWMLINNSSIRSDADKMCKTYNGATLVSIRNADENQALTDFLKDENINTLWTGLYCFGETNITACIWDIQAGSAANYSSFKVGYPDADYGRCVFYIGNGTDIGQWENEECGETMPFVCELPPTVHEGTSDFLYNSHCYFRIDMMKNITDSQHFCLSRCSNLVSIHSANENRFIQSIYDTNFTNIAIGGFASSKDYMIWMDGSPTDYNNIQFSYAGSCVIMAAGTTDTGYWYTRPCNETFWFFDDATTLEENDNGALQTHFENSVAFVMDHWKPLLIGGISVFVLLVSVFLITFFATSLLLRVHPKRRRKRVQNLKLRQKLVYFLFLNSVNFSFIHSENSTTITSVTPPDSYTTTLGINPVQITSHL*

>Cre|FL81_17189

MNAKTKILERLSQVFSRETSERAGTSTPSVSTISDEFEGPEQTIVDMYDAPPKTEFEPIERQRHFGILHYTVNNRFRKMMLIGVINVILIVSFFLFIFFFVIQPKTGGGGDGFQSSTLHRGAVTTSSPPALMCTNDFVLINEKCLKLNVTAYSKPTAETICNEVGATLLSINSLKENQEVVDYLKTQNVSSVWMRLVCNNNDKSSCHWNTGDNVTYSNFTKGNPTSDAKCVSLLLSGDTTGQWKSEYCNQQLSFVCELPTTSPDNCPYNYNHHCYLRFDQSLSFSDAQSTCQKHCANLPSVHSSSENLYLTSIYGFQNQNLFLGGFASSQNCIYWIDGSPTDYINLNSFHSNFACVISHMGDGGEWETVDCSQPISSFICKRPTDGNHKKLDPMLTEGDIQFSSQVGMYSKKRESAMKHG*

>Cre|FL81_17241

MIHLILLFLKCWMLVSQTADEPTADEYCRLNGGGVIATLKNAIDNRGLLTILNGTSVSRLWIGMICTGLSPSTCRWADTTTVQYTSFSSGFPNDRFGQCVYYSADGYPAGQWASGPYVTGDCLDYANYNNYCYMKEEDVQSFSWAQKDCARHNANLVSIHSYLENRFITSLFQEGKMDLWIGALAPGSALIVWTDGTPNNYYNLKNSGNGSCVSMGFDPNTNSTGDWSSDERCRENGGGILVTMKNAIDNRALLTVLNGTGIHRLWMGMTCRESSVSWCQWADKTDVQYNSFSSGYPNSQVGKCVYYNSYGDLTGQWTSEPCEEKLPFVCEILSSTPEIVGSDCYSQYNNFCYTRVRDKLSFKSAQQNCVLKNSSLISIHSYLENRYITTLFNIDGSVWLGGQITPYDTLEWTDMTPYDYSNLKHVGNGTCVSIGLVKYTNAMDWSTGNCESTYPSICKRPLTI*

>Cre|FL81_17267

MKILIIFGLLASFANAQVDRDWSFQRMYEFWDGGSSYRPRSNGFNSIEGDKCTFEYPVATDSQESAKRYCEENVPYHINDVTPGERTKCSAEATLICKNEWIQLFGRCYKMTKELMTRDNAVEHCKTQKXDATIAFLHRETLPFRIYDYFTRVSRLWIDASEAITXDLIDENVNGNLLLAIDGYMYNLPNVALTRVDSSETAMNGLIIRTAAFSKSSRKSERIGATCSANKGSIYHVLWMGSDGNAVSQPIDKSLWRSDEPNEICDAGSWSSALVSGRDGSPGLEAMSDARYAPIYCQNIVDSYSYGDCPAGFTEYYRKSLGQKWCHRFFPDKMVNEDAEAHCQKHGAHLTGYTNQEELKLLADLIGDNYEKLYHNLDTWIGARRRSECITTGTDNEPGYDRDPASKCSRVRVFEWINGVAPNPPDIEPDWNHDGEPNFAENREQCLAVMKGNKLTGTLNDVPCTMKYNFICGTEAPIIIKSP*

>Cre|FL81_17412

MKLLFVLFLKFVFISAQLDLNSIKKQQTYPATGPTTISTSAASTVGPCVGCQYGWVPYGGSCYKKMLDVLTQSTAEQECITLGAHLASFETNDKATAIKNLVLSAPLFSTDLFSYSSSSQESWIGLSKTSNGAWKWTDASEVEFANLPTGTSVTGASCVSMNTSGVWKPNDCTSTVTSFICKRLSSPL*

>Cre|FL81_17436

MKLIILYSCLFQLIVSQTLKCDPGFTMVLDKCLQIFSEPQNHTFAENKCRSNGGTLVIIQNAIQNRAISKFASDNNLSKVWIGVYCFGNSTSSCFYDDFSGPVSIGNNFAPGFPYSGCVSMVTTGSQSGKWYSYNCNDVDGINSYICEMPTTRNGTINGAESCVNYDGYCYYMTHDAPLEVAERICQENNGHVASINSQHENDFIKNTFASVYFLMLGAREYFPNVYAWSDGSKFEDFDNRYPFDIADNTRPCLVMATNTGLWQRAGCDIQQSFVCKVPLEPKTIEGNSHCNSTMLMAPTTITSYDYNIGSEVFTPCTWQIISPGPYLIQIYFVDIQNSTVEVFDENGKQIALVTSRITVYAPSNFVTVVHKNGGGFKAKIQKYESYFPTLVFYIIHPIAKSNMHCLRYYGSDNQIHSEIYECRLFGGTLVTINNAIENRAVVQYASDQGQERIWLGSYCFGNSSSSCYNDDYSDSSYSNFAPGNPLVNGSEGSCVSMITKGSHVGRWFSSNCNHNFYSYICQLPATLNGSMYKSDGKWLEVFNNSAYYVVEGSIDQAEIECQKKNGHVVSIHSKQENDFILNRMALVPENVRLGAKRVFNNSYAWADGTLWDFDFRDQLDDMPESLDCLEIFTVHQLWSRTDCDTTASNICKIPLPPIEDNSHCNTTLLMSPSTFTSYGYGMQGLQSPCTWKIVAPGPYQVNLQFLEMRNSSVTVLDASGKLIASVNSTVKVLAESNIVTVVHNGQGTFKASALAF*

>Cre|FL81_17908

MFVRTWILLLVFPVIVSTNECYVTPCTRDIIIVVDGSSSMQTSTYVSQEINMITKLTYSWTLDESKVRLALVGAYFGNEFNGLDYFTDGSLVEKRLQSFRLAAMQYGLFNGDFNTFFVSVQLFELGVVAQSIIDSITEDGICFLDQGWTTPKPEICTTSTTTTRAPTTTTKKTASVKPATTPKPVPPTQPPFPVGDYQDCSCTTQSLYIDIVFVIDVSEGMGLGGLMMVKAEINTLVGQMSLDPNIQKHVQVGLIKYSNEAEIVFKPSDYDDEDEFTEDLWSDPRLQDVDKVDEVNLHLGLQKAAKMIGSMRRGVRKVVVIYAASYNDEGNDDARQIAANIRESGYEIITVAFVEPESSNLVMKIGEIASPRMNFTSFRDDLLVEEMEDAFCQVNCYCPNGWKQLVLENRKYGECFFPTKIDASWTATKFECPILSKDHTGNGHLVYVNSALKNQFLNDFYMNHWDPENQEKPNYDIGYYYDKTTNKFIWVNGVTNNPYSNWAEGYPDITKGECVMAKRVDGTQEDFRWVSVNCQTDYGRGLCQEAACDSDFYCPPEYN*

>Cre|FL81_18659

MLKIARIQSPSKNNSLHLQIPCEVCKIQMSHGNHFGVNSCRACAAFFRRTVHSRWSQLKCQGGSCNKMTFFCKPCRLQRCFEMGMVTANFQYNRDGIVTPSVPTTISTVSHITPSLEQYLGRPHFVILSDKNPKKTIIDLHRLLGEATKILNLGPATPIFCDGSQLKKLSFGMKNEFDLRKLKIATRMTQTEIAGNWEFYVKKVATWLTHFDEFKKLPVGMKMKILQTIWHIWSRLEKLSTTAKYRRSSGRNKRSEIVVQSGVLIDISEVDFDSKWMSDYPTDQVRRFMMHSSCDQFTVTDHLTEMELSDVELTFMLAQLCFQYAGNRYQGEIQEVCDRLMTILSDDLHDYYVNELNMPRYFKRLAKMMQINNEIQVAQHPSRSRSNGSDPHVSNSEVGVHTSRNDNRAVGTYVSSYGIDRIWMGVFCIGNSKSQCYFDDQVGSTAIYDNFATGFPNAENGGCVYYSVPGSPSGQWMNFYCREVLPYVCELPITHNDNCDYNFNDHCYFPFVELPFSSAQLQCQNLCSNLVSIHSPEENRYITSIYSQSSYDFIWIGGIATSSDFVVWADGSVMDYSNLETFSTGGNCLKMALKSTDYHSRGGWYTDDCSTLAHFVCKRPIGAVDCSGTPPPPTPTPPPINPPTCTTGDHWAPGTISSPNYPNSYRSGCAYTLTTYGSNKIRLIFDYKQIYVTDSVDIYDVNMKFIIYLFLFIAACHAASNNTICTNGFNLINNKCWKLFQEPANHTMAERTCTGYGGTLFMARTAIDNRAVGNYVNSFGIDRIWMGVFCIGNVKNQCYFDDQVGSTVIYDNFAPGFPNAGTGRCVYYSVPGSPSGLWINADCTEQLSYVCELPTTHSDYCDFNFNDHCYFRNDELPFSAAQLQCQNLCGNLVSIHSAEENRYITSIYSQFSYDFIRIGGIATSNDFVVWADGSVMDYSNLETFGTGGNCLKMALKSTDSHSRGAWYTDDCYSPGHFVCKRPIGAVDCSGTPPPPTAPPPPITPPTCTTGVYVAPGVISSPNYPSPYHSSCAYTLTTYGSNKIRLTFNYVYPYPNYDFVDIYDGGSTDTPKITSLTGYYGGSYTCTSTGNIMYINFRVTSSNSPGYIGFNATFYSVF*

>Cre|FL81_18956

MSCSNRRSNSSLATEFSQAKHDFIFNVVQNTTGFSPPYQYHVGLNYVSGLWVWTQPTGRQQVPLQKPFMWLSGNPQRSSTQSAVMNMQSGHGTGWQNIATMTLSANFICETYSCDTDNYCADYSPLSYVDRPCGTDLSNLWLDVIAVVDNSRGMTNKGLSNVASSILSVFGENTRIGSNSVEPRTTRLGLVTYNSVASQKADLNQYQSIADAYTGVFDALSTTVDTIQSYLATGLALAERMLVDQTVNSTRAHYKRVMIVYASEYNGNGESDPLPLAERLKLSNINIITVAYEQPGSVGLLQGLTQIASPGFSFSTNCFCPDWIQYRGSYSDPASSRYGVCLLPVGVPVVWAAAKIECSNRWNNSYLATEFNQAKHDFIFNAAQGSYFQQNPYQYHIGLNFVNGAWVWDQPAGQPQVNLKSWFNWGTGFPNSPASQSAVSNIQSALTTKWNNVGTFTTPATDSYSPLSYVDRPCGDDLTNLWLDVIAVVDNSRGMTVNGLNYIASNIASVFGFGTRIGLNASEPRTTRLGLVTYNSVATQMADLNQYQSLHDAFNRIFDDLSNTVDTTESYLSTGLTLAEKMFNDQSVNSTRAHYQKVVIVYASKYQTNGESNPESIADRLKLSGVKIITVAYGNAYGLMKSLSIIASPGFAFSNLVVSPGNLVGQVQTSLLESNCFCPDGWVQYRESYFDPASSRYGVCVQLVSLQANWLSAKMSCINRNSNLATEFNQDKHDFIFNVARNTKGFAPPYKYHIGLNYVSASWAWTQPTDRQQVPLQQPLMWLSGNPKPASTQSGVMNMQSGQGTGWISIAEMTGSANYVCETYSCDTDNYCCSADEVCGGSVTGIWLDIVIVADNSQRVHQYNFVSDVQNSIYYIFKGVNFPYTRVGFVTYNYVATVNADLNKFKSPSALSQGVYNSYNLDNISPEKTSFLGTGLTTAGDILTVQGSADGRVNNPKVIIVYASVLNGTGFVDPLLVANTLKSAGITIITIPLDTDHNGVIQKQLVSIASPGFAFDYLNPIYSDPVRNALRDVNCFCPPGWTQRRESYNNETRKFISCFQFIGAPTSWNTAKQGCRNLWPDNSYLAMENEPSKQDYIQGAVRNNSAFQQTPLQYHIGLNMMQGNWIWDQPYGLPRKILNWDDWMPNYPVVSSSMTAVMNVQNSTSSGWQNIDPIKVSAGYVCEAVACSVNNFCDANWNTKKQT*

>Cre|FL81_18957

NCFCPSNWIQYHTSYSDPASSRYGVCLQLVGVLANWVAAKNGCRMKSNTNLATEFNQAKHDFILEAVKDNSGFQRNPYQYHIGLNYINDDWVWDQPAGQPPVKLQSWFNWGTGFPNAPASQSAVSNIQSGVTTKWTNVAMYTGAANYVCETYSRRILSYVDRPCSTELSNLWLDVIIVVDNSQGMGIVRLTNVAANILDVFFNTSIGSNSSEPKTTCIGFITYNSNATLNVDLNKFQSSDSLFVDRVFNFLSNVAYSRDSFMGTGLPMAEQLLGRQGFGIGRDQYQNVIIVYASTYQNNKEFDPESIADRLKKTCSAQYSPQSYVDRQCGTDLNNLWLDVIAVVDNSHGMTNGGVQSVAANIASVFSSGTRIGSNSTEPRTTRVGLVTYNSXAKLDAXLNKFQDLDGLYNGVFKDLSDVVDTTDSFLATGLNAAEELLQSQSLNTTRDHYKKVIIVYASEYKGSGELDPVPVANXLKGSGVVIVTVAYDQGGDEGLLRDLANIASPGFAYSNAPNNAGNLVGQIQDSLLQTNCFCPNDWTQYRASYSDQKSFRYGVCFLPVNLPAVWNAAKMACRMKSNHAHLAAEFDQSKHDFIFNLAQDSNFKPNPFTYHIGLNFVNGAWVWDQPAGQSPVNLKSWTNWQSSYPKSPASLSGVSNIQTGLTTQWNNVALFVGAQDYVCETYSFSVYADSYSPLSYVDRPCGTDLSNLWLDVIAVVDNSRGMTVAGLNEVAANIASVFGSGTRIGLNASEPRTTRLGLVTYNSVATQKADLNQYQSIGDVFHGIFDALSNIVDTNESYLATGLELAERMLIDQSVNSTRAHYQKVVIVYASEYDGNGELDPLPIAERLKLSGVKIITVAYGNAYGLTKSFSNIASPGFAFSNSDNQGKLIGQIQGSLLQANCFCPSDWIQYRTSYSDPAAYRYGVCILPVTTPQVWRAAKMSCSHRWNNSNLATEFNQPKHDFILSAVRNTQGFSPPYQYHIGLNYASGSWVWTQPSGRQQVPLRQPLMWLSDYPQLASDKSVVMNQQNGHETGWINIASMNVVANSVSDYSPLSYVDRPCGEDLTNLWLDVIAVVDNSRGMTVDGLNEIASNIASVFGFGTRIGLNASEPRTTRLGLVTYNSVATQKADLNQYQSIGDVFHGIFYALSNTVDTTESYLATGLELAEKMFNDQSVNSIRAHYQKVVIVYAATYQTKGEMDPESIADRLKMSGVKIITVAYGDAYGLMKSLSVIASPRFALSNLADSPGNLIVQIQISLLEANCFCPDGWVQYRESYFDPASSRYGVCIQAVYSS*

>Cre|FL81_18958

MKHHSLLLALLGVYACSADYDPASYVDRRCGEDLSNLWLDVVAVVDNSQGMTNPGLINVASDIYSVFSSGTRIGSNSSEPRTTRVGLVTYNSNATQKADLNKYQSIDDVLNEIYDDISTVVNTADSYLATGLQLAEKMLIDQSENTNRAHYKRVVIVYASEYKGEGELDPLNVANRLKLSDINIITVAYEQKGDDGLFHDLSQIASPGFSFVNNASDTGNLVTNVQNALLQSNCFCPSDWIQYRTSYSDPASSRYGVCLQAVNIPSNWLGAKMSCSHRWIKSNLATEFNQAKHDFILSVAKITDGFAPPYQYHIGLNYASGSWVWAQPTGLQQVPLQQPLMWLSGYPQLASDKSAVMNQQSGLGTGWQNIATMTGNYNYFCETYSCDTDNYCFAQN*

>Cre|FL81_18959

MKLHTPLLALLGVYSCSADYDFASYVDRRCGEDLSNLWLDVVAVVDNSQGMTNEGLSNVAADIFSVFSSGTRIGSNSSEPRTTRLGLVTYNSAATQKADLNKFQSIGDVANGIGNALSTVVDTTDSYLATGLILAAKMFNEQSVNTNRGHYKRVVIVFASEYKGIGELDPLPVANRLKLSGVNIITVAYQQAGDDGLLQGLSQVASPGFSFVNNPLNLVTNVQNALLQSNCFCPSGWIQYRTSYSDPASYRSGVCLQLVGIPASWAAAKNGCRMKSNTNLATEFNQAKHDFIFNAVQDINSGFQRNPYQYHIGLNFINGDWVWDQPAGQPPVKLQSWFNWGTGYPNSPSSQSAVSNIQSGVTTKWTNVAMYMSAANYSCSYVLADHYTPYSSLSYVDRPCGTDLSNLWLDVVLVVDNSEEMGSQRLFDVAANIIDVFGANTRIGSNSSEPITTRVGLITYNFNATLNANLSQFQSYDDLSNGVFHSLSNVTNSTDSFIGTGLAMAEQLLRRQNFNTTRDHYKKVIIVYASAFQRNEDETPEWIADRLKGSGVKIITVGYGNSHGLIKSLSNIASPGLSFNSSGDGNLINQIQTSLLQANCYCPSTWIQYTSSYSNSSSYHYGLCIQPVPTLTTWRMAHYACIFLGNHSSLATEYNQDKHDFLLDAVKDTSGFSPPYQYHIGLMSNSYYWGWDPPTGSSGPTLQSWSNWILGFQGDTEARSVINIESENLKEGTTGWLNIDDTRTKAGYMKIANMKLHTLSLALIGVYACSADYDPASYVDRSCGTDLSNLWLDVIAVVDNSRGMTNKGLSYVASSIISVFGKNTRIGSSSAEPRTTRLGLVTYNSVATQNADLNQYQSIEDAYYGIYGALSTTVNTTESYLTTGLNAAVELFSRQSFRSNRQHYRKVIIVYASEYNGRGEFDPVPIANRLKASGVNIITIAYEQPGSAGLLQGLSQIASPGFSFSGDNIAGNLVKEIQTALLQSNCFCPNDWTQYRGSYSDPTSYRYGVCLLPVNLPKVWASAKMACSMKSNRTHLATEFTQAKHDFIFNLAQHSNFKQNPYTYHIGLNFVNGAWIWDQPAGQSPVNLKSWSNWQTGFPIGSPASQSVVSNIQNGVTTKWNNIAMYTAAADYVCETYSCDTDNYCDADFVYDN*

>Cre|FL81_18960

DTYSPLSYVDRPCGTDLSNLWLDVVLVVDNSQEMGSQRLHDVTSNILSVFGADTRIGSNSVEPRTTRVGLVTYNSAATLNADLNQFQSFSDLRNGVISFLKVAANTKDSYLATGLAMAAQVLNVQGLRDHYQKVIIVYASKYSGYGDLDPQPIADRLKGSGVKIITVAYGDETVLESLSSPRFGFNSASGYPQIQNALLESNCYCPHTWIQYRTDYSDRSSSPYGVCILPVNLAANWFAAKYQCSNSWNNSHLATEFNQAKHDFIFNVAKDYLRQNPYQYHIGLHYANGDWVWDQPAGQPQVNLQRWSNWQAGFPIGSPASQSGVSNIQNGVTTKWNNVPLYTMTADFFCETYSFRSADEECGGTVLDLWLDIVIVADNSQRVNQNNSVVDIQNSISNIFEIVPIPINRVGFVTYNSLATINADLNKFKSWGDLSQGVNDSYNNMNLSSENTSFIGTGLITAGELLQVQGSAIGRVYYPKVIIVYASAFNGTGLLDPLSVANTLKSAGITIITVAVDTDNNGVIQKQLASIASPGSAFSLDPDDDHWPVREALINANCFCPPGWTQRQEFYRNKFKKYASCFQFIGTPSAWNFAYFRCLILWSDGSYLAMENEPSKQKFILEAVRNNSAFQQTPLQYHIGLNMIGEKWVWETSDGPQQELGWNDWMDSYPVISSSMTSVMNVQNGASSGWQNIDPDEVMAGYVCEAKACSASRFCYIKTSNT*

>Cre|FL81_18975

MILRTLLLILIAEVTWCQIGQPGQAPGQVPLDPGYQQPLEPIKNILQGAYGSNVTLYFRNSPYRVMGDLTVEYGVTMDIETGTRIYFDTGVGLIVKGTLRAIGNEFAHIEMLPYQQQINYDSEMPKFRLVDGPTVRQGRLQAQFRDRWRSVCTMVTNWTSIDTGTACRSMGYSDGGFWKWYRRNNDTYPFVMPKPDCHGSAKNLWDCPAFSDPQKIRLSENLCQGEDDIGIYCWGPPTFTGWARHWKGIQILNSPFHYVNSDPDLVAVNRESNSRLEFVDILYAGYDGVNKNTTSALYIEGVPPIMNGLRIEHSARDGLQLLDTNGPAIIANSTFSFNRGHGIFVVNTTDARIFVNNTRIEGNWGDGIWYKQRTGVNLIDYGMREKRGVGSGRLDEEKPRIDMCSEHRIDDNHFFPHLISVNLKNRTFLDPSQPPSCWMTVSLPPRLPYTYSIQWLHIRDLNPVTSRTTLLICDSNNPDENSCSTQRFRIPIRNEIYPQSISLKSSGKPLYLALEHVLDGDQAGYVQGDVYLLFNIHASVLDKAYYGLNVTNCIIEKNTGNGVFANDIRERTALTNVTLDENQGYAGFMVRDGAADIWLNETRILRNWGDGMNISYAGGSIMVNGTRIEKNRWRGAAIHYNQTIPFFPLYNEVIFKGRPSNNKFYLPTIISENEWGGLLVGNYCAYGNESSWRWNSRNPPIQGLYPKLESKILISWVEFLKNQYHPAMEIFSCRDPHVTTNIVDITGNRVDGNLGFGMRISPAVNMHTLINSNQYLHNNDTALYVRNGQWPELVNLPAEVTISKNVFKFNHAKYIISIGMNEDGPRQFLTFNQQNEIRANTVFDPFPSLPPRSTPYAALVVSSSNVKIHRNCFNNERAKYEIATELERHAKWIDARENNWGFQEIPRFIDKFFDQFNRYSLASIDIDPYMAACNQRMPYISLLNGAFRQFKKTSEPNKLGGIIYENHDLLKGRYTVTEDLQVVPGAKLTIASGSVLEFNHGIGMIVQGDLIRNEYDQDEKVIFTSSPFTLAKRQNIRLVDVDGNDEVTEGRLEVLVDDQWGTVCNRSWTPQLTILACNQLGLVADIQYFENWRIFPEAGDLPMVMDNIRCEENEVDLTRCRHDGVERNCAAGCRSSEVVGLRCLEPRWAGVRYSLLANPPTVTGQTTMDNWRIEKGGLFNFRTSEFCAAFKIDWNYHTFHRLEVKNNFWDGVDVVYNDLVKKPAIRNSIISNNRNNGFHIRSAGITVENVTISFSGQSGMRYNPSVSALEQDDIVSWLSLKEQPELEANNIFRIPDQKLDLIEVMESNLNQRKFLVAAETDDCPDDPLQECVYNLMIRSVGYQYGLPSKMAIQIVNAPSNVSDEDAIFTEVSTGKSWSARKDQIYFPVVSTENAMRMRYTRSYGKPKLVILVLFLDTQEYVDRFIHLYQSRVEDNQYGFSAVHYSNLTFSDGRLSNRWNNEKIWLQKVNFTRNSEAVVWLHSPQHAVIPGTPIAEITYHFDNCSVVDNTGPIIESHRDLYASANVFHWILWSNTFANNSRSGIAVALPDTYDLLAKQTHSFWLTENRFERNDDFKILVDGYYAFANISSNNFTQNYAPKQFGMLELRGMEKKLICERNRFFFNWGHWMIKIDATSQYLKQVDVPSYVRYNYIEKNTFIRQRADYVDMWPRSYALGVFGSQKIDYTDIYDTMNATYNWWGTGNEAVISQRVFDFDDWNTYTRAQWSPFYVSNDLSINFWWNPYRDGQLANATYIEPTVHDLHGRVYEDKNLTLITERWYEFPHYYRPFRPYRITRDVTIMPGATLYIQENVEVHIWPNVRILVLGNLVAEGSYWQPIRFKPINTTEYNEIKGRIPTEYRKRRGIVFDGEPTNSEKPRPKRASDRSKPDLVFRDFPTLHRDDPYYQRFTVSLTANGSDYGRSGFLQIYNATTGETIPSCDRQFTIRNAQVVCRELGMETQNVYHWLTPRWDYNPQLKILKTYMEPRECRGDEPSLDRCNLRLSGNDSQWMCMDSENFNYIYCGTNRSLSREYIGNWGGITFAQPTLEHEYGEKRGTRKEKSILQNVEIVGGGAGHNDSWQSAGLQIFHRSPILDHVNVTNCSVHGVQVISPNDRITLLNLNVTFNQGQGVNIMTTFVQAPSTSQDAMKKPMSIPYYSQGMMDMCAAVKRFEVKNRILLYYKYDSFPVDCVKIFTSHGRRVAFRIVQYHLYSSPTDLGRSDALRLYSSESFTPMSLLADFRSDYQSVDPSVAVSSEEIAVHLRATAADGVYGFIAEVSALPSNSEQHTVGEVVIRGSRMDNNDRGAIEYSNLGEMSPNLVIESSSFSYNGIHLFGNISTSSQAIQLHLHNTVFFLFRSNSIAHNRGGLYILATSSSPVVRLGALVKNCMFVYNSNSTTVALSGNNYQSISLLNNVISHNFALYHDTVVAHDVAINMTRNTLFSNTGLHTLDIHANSKISADKNVFFYNHFYDNLALGHGHQYMEKFGYQPQKENNEFLNRPRREIGKSEVNRNRKKRQVLTQQGISFDWWTHVDNETTRYRSTIIAGSSQEIFKFNTFNDPLNDYELTTGRQSQYEIGSIDAKENYWGYPGTIGVASGKIRDHEDYPELVKVDYTPVMESNTSLIEGDCPAGWFQAGHEEFKSCFLFVPSAVTYTKAVEYCKELGAFVPYLRIDDILQTQLAQRIEKFSIDMITDQERLKAYGVEDDIHLWISSVNIPNTQCGWLSARTRRIGDVNCNILLPFVCEKGTHPYSEPILWRPGIIIPLVIFCIVLALLVLLVVCWCCKSRKRNEVLIERKQAARASLKLQKRHQEHQKKKMQTGSEHTHTSAHASLDGGSTISAYDWRAGRQPPRQVARSPTDTLSTATSDHTYSYAAYTPHGGPTTTTGTFQSRRNNRYVSTNPNGYSEITTPTVTTVPYTGATSSTGTTVKMRLHQLRSDTSDATSCSTCPSDSERTSTATDISSSYTSEASESTLQSTVVNNRRSPLPPAKSPIPALRKGNSNGFLNMTNNFQPLPTDPPRSQPPALQSQSSLNRFVELHAPPRTGGSLKHKKPVIETSM*

>Cre|FL81_20271

RQGAHLHSDSNDSSFGFSCFQLHWLHRLFTFDESVQYCNSIGGKSVSISSYSERDALVALTKTNILQPWLGARRNTTTNKFYNLDGTYFYTLMWSTNEPSVNGDCVTFKGASPSGLQVTQCYQLQPAFCKQTXALCNSAVIGGPNTWSGTFQSPGYPTQYYNNLDCRYLINSPNNTFITVTFYPFLIEEWYDSVDIYEGNSTSYANWIGQPSSYSSGRGFESSGNMMNVRFKTNYAITDKGWLATWKAKKDMPVITQSGTNGTMTSPNYPNNYDTYDEQVYQISVAYGMQVNLTIDDFRTXSKYDYLNIYNSSVQSNSTLVYTLSGTSVAPFNWISPRSYMSMKFVSDGXIQYKGWHAFWNATAADNNHCPSGWTFSTNTSYCYIQSSQYMTYSEADPYCQSIGGSQVFVFTTRELTWLTDFTSSSLVQPWIATTRNTTTNIWYNSDKTTPPRSYWTTGEPGVNGDCATFKGITTAGLKATQCYSLQPALCRQMPALCPTIKDYGGSSTRSGTIQSPGYPVQYYNNLDCWYTITSPKNTYITLLFNPYMVQDYVDYIDVYDGPNSSYPFLGTTDYWYFLRFDFESSNNSVSFKFHTDNIITDKGWQLTWNAKSNTPPISQSGQNGSFTSPNYPNDYDPYTEQLYYITAPLGFQVNVSITDFVTELNFDILEIYNSSYVSSNYLVANLSGPSIAPWSWVSPSRYVTMRFKSDYMVQKKGFSLLCPSGWTFANETSYCYIVSDRYMAYSETAGYCQSLGGSQVFVSLSKEFTFLNYFTAGLFAQPWLAITRNVTTNKWYNSDGTTPFSTWWSPGEPGPNGDCATLRGSDPSGMKATPCYSIQPAMCKQMPALCPTTTNYGGLYTRSGTIQSPGYPAQYYNNLDCWYTITAPNNTYITLQFSPYLVEKTFDYVTVYDGPNSTYPYLGKTDEYLNPRYDFESSSNYVSFKFHTDRTITKNGVYSAPINQTGINGTFTSPNYPNNYDPYTEQLYYITAPDGFHVNVTIDDFLTEARFDVLEIYNTSTVIANNLVANLSGNATAPWWWVSPDKFVTMRFKSDGSIQKRGFEGSWYIL*

>Cre|FL81_20821

MQLFLLFLFIIPAVYGLSCPPEYILVSGIKCLKMFSTPANHWEAEKNCTSVEGTLANIKNAIDNRVVTEFVRNASVNAVWIGLTCYGNNKYSCYWDDASGMPSLYDNFNYNEPSDKENGNCIVMSAETNWKWESKVCMDPAWPYVCEAPTTVEKSSCSQKYNYNNNCYTKSLQVANGSLAAYNCQNKSDTLVSIHSKMEVDYIRNLYKGTNTTSIYIGATVDANDKITWLDGTPFDFDYRNPLDTQKGDCVVMDVHGYGLWSRVDCSQNFEYLCKNPLHGGSGPEVLEDKPEIFPTQSGCNSSTVLAPGFISTFEYPLNGLNIFACYYDIVALGPYSVGIYFDFIDTYGELTVFDYRGLKIGDFGGIDNRDQVAVYAPYNTATVYYEPRHKNGTEKDRGFHAVILPL*

>Cre|FL81_21016

LFVISVELVVPEKQQTYPATAPTTVSTSAPSTVGPCNVGCQDGWVPYGGSCYKKMLDVLTQSTAEQECITLGAHLASFETNDEATAIKNLVLSAPLFSTDLLSYSSSSQESWIGLSKTSNGAWKWTDASEVEFTNLPTGTSVNGASCVSMNTSGIWKPNDCTSTVTSFICKRSSSTL*

>Cre|FL81_21460

MIRSILILSVVFVAIQAMMAPCIDKPVCPDGWKKFEDRTNGQWCMKVFPGNMTWWEAERECRCTTKGAHLSGIESSSEKQWVEGQGQEVLDKIQDKNGAIWIGAYRRKECPSGATSSDVNCHAEKLFQFTDQHTCKTFIFQNWADNQPTNNAGDDCGAILVSTESSGDNVDASGKTVAKNCLQTTGTTPIMTSVGYVCGVKPAYPGNDYGGGYNTGYGGGDGGYGFGGGEMVVIGAGKPEKKKN*

>Cre|FL81_21470

MRAVIIFFTIIVGSLTVENDMDDLLNRVNITKMFELSSGDAAILNELNMIRKLIAEGFMNNPFENIGKLIFDGAIQWIKNVYDKAFSQAEYIAEYGLKVFEFADLDFGAALIDTQEALTNLAGQALDGLEDLGNGAKDFFSNNFPSFGKRKKRYIVKFIEEKVTAFRETIEKPKNVFGAATNMNMLTWNRRLAQLAMVEKENIENGKRVVEYEGKRYRVNLYGTLATYVIANTGIQWIVRTGYLAAKLWFDKIDIPNQKEIDTKDKTHEMLFADRSEVGCFFTWPNTICIIGPLNATTGYLYQKGDDACLDCKYGCTEALCNPPPHYFFALREKYKKHLRQKIEKFEIVEGFDGQKPILGKNFMIDSANYSNNSLIPPESSKPSGLQDASQSGPVILSDTIGTAKCASGWIRWEGNGCCYKEMGSPMLSWYASEDWCWSQRAGAHLTSIHSQAEAVWLNYQYKLWWSKMDDWIGLRRNCDNTDWEWTDGTPVDFLWWQPGYPIYGGIEDSCTALWDSSVLRGMYGFLPGQFDDGRSCTGSVAWAVCNNYRTKVCQKRLYYYFNYNHHINNDNDNSHDNNNDNLEARKELAGNFLKDNQNLYRGAEDERWMFKFLNQNSLIPPETKSSSLSDSSSANLVVLSDAVETAACASGWKRYTVNGMCYKESTASMSWYAAEDWCWSQRAGAHLASIHTQAEAQWLNSQYKLWYAPMDDWIGLKKECDMTAYYWTDGTPVDFQWWQPGYPQAQYAEQSCTTIWNTALLFLVSGYTPGQFDDMKECGMATGSYALCKYDPNTYYYGNDDDTYNNYDNSNHNNNITYNDYHNSDHNYCNHYNNDSLHNSNDYNHNTYHNYDNSNHDNYNTNNYNNNHHNTYHNNNQVSTTTSKTTSKATTPFDTSKCANNCPTGWVSYNGLCYAKIKGPAKSSDFNTECVRLGGQMAEVSGSGVNEALRSFSTNTASTTLEEAWMNLSGGFTNMAPGYSAATGSCYTIVLSQSDNGVYTQRGLWRTQLCTSLKEFGICQKAS*

>Cre|FL81_21471

MCYKLSEYDMSWYKSEEYCWNQRAGAHLASIHSEAESRWLNAQFRDKYGQMDAWIGLRRDCDNVTYVWTDKSPTDFLWWQPEYPRSEFAEFSCVTLWEQSFLLDVDGYVPGQYDDMKECSSSGGSVALCKYDPNTSIIGEKYVKKTCSLIPETTSTTTSTTSTTTTTTTTTTPTTTASFYRLLKI*

>Cre|FL81_21528

MLWAVLLLVLAVTTPTQSQLVTDRSCGNDLSRLWLDIVFVVDNSKNMNMYKVFDTISSLFSPYVQIGTGYDDPRSTRVGFITYNWNATDVADFYKLQSYSDLSNQIQQLSTTPLSRRDESYIDTGLAAAIRMVNATQGLRDNYKKVVVFFTSQYNYYNTYPEDQAKLLKSWGATLITVNTGGDDNTQENLHDKIANKGMAFLMSDGNTTQELQRALLATNCFCRPDWYQYHYPLKSTDIFSNYGVCVYRPAVAMNRISAQNYCHQLTDTSYLVSELDEQKRAFNWEYLNSKGSDPTHAFYNGLTSFNGTWWWDQPNGMPMWPLSPYSGAAPQRAGCVADMKYSDGTYSWTPISCTNLFRFLCESVACDTDNYCEF*

>Cre|FL81_21530

MFKLVTLALTFTALVSAQTGWTDSWGTTGAGPTYGPSTPRPTPGGSNVDRECGGDLANLWLDIVVVVDNSKGMTNAGITQVAANIATVFGNGTRIGNQYTDPRSTRVGLVTYNKVATEVADLNHIQSIDDLYSVVFSTLTSVSSEDDSYLATGIGAAEKVFQNGRNGNVRSNYKRLVLVYASAYKGDGQNDPIPVSDRLKSSGVVISTIAFDQDGDEALLAGLAQIASPNYAFTSEDLNLANFDDENSYKYGVCIRAATISSSWTAAKFACQNLAQSGFLATEYDGQKHNFLFRIAQNNTAFSAPYIYHIGLSYVNGGWNWQQPAGYPLRPLSGYTAWNPSYPKSFSSNTGVVEQQFSSDLTVGWQNINGYSVAEYYMCEVASCDTEKYCP*

>Cre|FL81_21536

MKPYLLLLLLIPIVLFSYIDAEQVCRDGYTMVNNKCLAFMQTPQKYDDAEKTCRFNSGGVVVLSKNAVDNRALVNFLSVWNVANVFIGLKCSNGDTSTCQWDDLDGLNDYNNFAAGYPLSSQECVILDSQSGKWTSQSCDQPMQYVCELPPTERDCNSNCEVNYNNNCYIRIITPKSFADAENTCKKLNSHLTSVLSYLEFRLIAEMYRTPGQYWIGGLCASNDSPIEWLDQSQGEFSFGKTIIDGNCLQYGMKPLLILLPLLAYTSISTAEKVCASGFKMVNNRCLALVRQYKSYSNAEKWDDVDMLGGYNNFNGGIPSGDVCVQLNVGSGKWQSESCDKTLPFICELQETQKDCHSNCDYNYQNHCYTLVKQQKNFQDAENHCKSINAHMTSIHSFLEGRFVAQLYADWGLYWLGGTLTSADAKIKWLDGSSDDFETTKHHKDGNCLQYRVDSIGIGHDWFADNCGDESIFICKRPASC*

>Cre|FL81_21802

MTNLRWFLQVLSCLIVLGYCTDPRYNDSDMYDDCFQGAPGWYHLTATCIFPLTQFERNYSTAAQMCGAVNHSIGYRETNWLIAQQLLEMFTNEKSRDLMKLYWTGLSVRNGSIVVESEDATNDTVANVASLSSNELDGSCIALDLRNSSNFGWRSLPCSSQLPILCQNYACLPGTFRCADNSKCIPSSFQHDGFNDCLDGSDEMPVTQSIPLSSSTPNSLLSNVLRENPVYEWPMIVSSGGILSPTTVRYGRGECTHRWTVLSPNDQHFIIWIKWMSPTTSTAIYVEGKDQNGRIYLNSRNATSSFTLLSSSFVLTASDTDTRKIEFQVHYQEADKTICRITENNQLFFNSESIPMSCNYRFTTKIPSSYVAILIRKCEGPSPIVSTIQFNNSTVSLTRRQSKKLLIIPSNSLNIHVNSTWPGSETQLDFQFFELRPGEESVDVFMIDSDFEIEWIPKSSEICMEGQFRTLTVNMILTSSDESENSSQSSTWNVERFLKSCSEDNVQIVSKNQSAYVSNLGSVTGFGPTTVVIHQSAEPFEFYSIYALQGIARDGSGWAFDEGVSTTTISTTLSTTDIISTTTSSPSSGTKCKLPTINNGYIKAVSDHAYSVGTIVSVNCDEGYMLDSLPYNIQCSDNGTWIDGENRAYPPVHPIVKCLHINCPSETGYEIDNSMTPDTYETSYGTVRRYQNRSTYGYQPFCICGDDKKWLADWMCYNTEHLTKYEIPNGCIIPEVHDAIFVTQLPNDQYVAPSGYTIEMDCMVCPTVKKTYRCLDGLWRASNGSVQAYDSFECRCQDEPGWIDPCLPHGTYVQYQGYYSCQCENGWKYGDGTCVDIDECNETSSFCDPFAKCVNTNGSYYCECPINYHLFNASNFNPSQWGSIQRYLIDGYSCVETTCIYDIEWSQWNIQVIKPPTLSPYYKSGNSMGYLYATNMCDSDYKCLLQFKETCLNGDWLPRPNPLVACKALDQSVYSFQPVAPYFHVFQTIDLACNQESKIMI*

>Cre|FL81_21849

MRFATSLIFVSIILLVSGNRRFGFGVKTCPKGWLQFQRHCYIRQPDTLDFKGAMESCARQGATLFQFDSTFEFAAVRNLFPDYMFTWLQAEIEEELEWLYEPYEEKINGKNTVATCIAFYSSPTKSYNYYYPCTSRFHSICEKPLDAFHVWVA*

>Cre|FL81_22637

MHLCGYPEYPRWSSTKFTVASRNGVFEGSEGSEGTESFGESADDFISKYMQGVGVTRLWLGLYCDHSTPESCLWDYGQGNALLTNRFLSDSPDISKGKCVYMDGKSGNWSSVDCEEKMPYMCELPQTLEDPSCDHNYYGYCYIPHSTLLAYGDAQKVCTQNNADLISIHSEFENVFVSNIFKTPGAILIGGVALSKGTIIWTDFSLSSGYNNIQSFNTGNCLFMNVYTDNDTDGFWYVDQTVPITLTCTATTITVNLCHSYVQAIQEINKDYIWLGGFAASKNFVSWMDGTPMNYNVFNSRKDLLQDKESCNDK*

>Cre|FL81_22643

MKLNTELFCFFLLISLFFDVISSTCTNGFSEIYKNCYKILPESLTKADAKTRCERDYGAAILTIHNAADDFISKYMQGVGVTRLWLGLYCDHSTPESCLWDYGQGNALLTNRFLSDSPDISKGRCVYMDAKTGNWSSVDCGEKMPYMCELPQTLEDPSCDHNYYGYCYFPHSAPLAYGDAQKVCTQNNADLISIHSEFENVFVSIIFKTPGAVLIGGVALSRDDIIWTDFSLSSGYNNIQSFNTGHCLFMNVYTDNDTDGFWFVDTNHYNSSVSHPSTTQNFSKQRTLPARYNCY*

>Cre|FL81_22646

MISRIKNIYAEFQSRRGGIINNDSASVSSAPGTNEEPTTVEIIDTNPPSEFGLVDKQRHFGILRYTVTNRFRKLLLIALVNLVVVFAALIIFVFFLILHLNKKHEDIHISSTSHNHLETTTTSTPTTSAQTTKTDTTSHSYSCVTTTELFDNTAAKSVDDTGCSPNQKSTFFFAYSYDLTIDQVLNTWQSISNNSNFFFEKYALGIFDMMNSNNNITGIYDTLLSGLPNSTESLKDPSEGGGVLSIIDIFFFEDITHCGATLFIITKRLPTDTSISELVSMLQKYHTYVTFVVSNNSFGGLSPESLYRLASETNGLCIFTDDDRIQEFRSTSFWHQTPTWLPSIWPSYLVYSFNANVTNSGSVTLPIFNSPLVGCYYICMTLQDHGSLDTFRMVKLTWYNAKSSKSGYFEETVESHADYGNTTYVKKGPYTLDAVPYNMTLGFEYSDDKIKILQIRIYSVSIMTMPYRGEFVLAKTFGKTKIPDFSESPPEEPKNVLRKYWKAILFGFMCFLMICLIFFLILFLVYWKQTDSITSSTVLPTSGTTTDSNATTEVSTTPLPIIINTTSKPHPAPFLEGSTTVGFSTGSQTTNRLITELNSVPQSTVTELSTPFALRSSYSTSGTTKSVTTAPAIVTTPEQSTTFGLTTTLEDSTTSKPLTTKSISTTPLTTPKYSSSSTQKTTMSSTTLSTSELTTSQVTESTEVSEPSLSSTVSSTETTIFTNPSTVTIISTTAKPSESPTTSKSRTTANPERSTTSETLKSSTTVLKDLTTIKHIPSSLFTTSETPTKPSEGSSTSKSQTTADLTTIKYLPTTETTSVQYMLFTTSTTSKPSERSSTSKSRVTSDSKITTETTEVSTTVSQGTSRSWATVTTPSVLTTPTSPFSGIQIETSTGSSTTFTTTPGLKSTKPASTLPQSTSSSLKTTEGSASTVSQSTSTTAMKTSTTPKPELTSDVRTSSAPETTTETSTVTTTESTTPKSTTVEITTVTNPSFSSTTASTSTVPLASTSSVSSSTTPPVTVTTTVTSQSVGSTTGSTTAVPTTPDKSTVSTIRAESTTARTSDRSTSSAYKTTGVSTNTPVESTLSSTTTGLWRTTTPKYLSTTVTTPSSTLPEFTTTKTKQYSTTSQHIPTSGYQSSGTIRTTEFSTTRVTTPSTSVITTTASWGTTETSEHSTTSDESSSTSTVSAKSISTTPSTSTQETTMSSTAINTSKLTTPQSTTTTTVSEPSLSTVSSTETTIPTSEKTQTLSTTSETSNVLTTVLKDLTTIKHLPTTETTILTSPSIMSSNVITTTVPLSNTTIEKTTTYKSSPISSSSTAETTKSTSTSATSQQSTTVSTDGTTTTSPLETTTFLKYTSTSEILKSTSGTTIPEATTTTPWLSSISTTTVPLTTTEKTTSEPPENDQVESNTNFILKHWKSILLVILGLVLIALVIFLIVFFVILKPADQSTSSTVVSSGTTIGSTTVTKLATTTKPSSKSTVPSVTTVTEKRLTNPLHTNGPITNTAASEFSTKPSSITTAKLVPTNSHITTKSTGNPWTTVATSKTATTPVELKTSTQILTTVSSTEKSTMSSSTTFKKISTTEATTPTSTTVEITTSTVPLASTSSPTTVINTKSSAPSTATSTPSTTEHVSTSGATTTIATSKPPPTTREQTTSESPEPPARSSNFIMNHWKTILLVLMGLLILSIAIFLIIFFVILKPAGHTTVSPSTTRSSIGTTKLLTAVSSTTSKSTNGLTTVIKSTSTVPPINSTTRNKTTETPPIASVQKQKSPDPVFKYCKIVFVVLLGLLTLGLIVFLILFFVVLKPADQSASSTVVPSISTTGSTTVTTKLATTTKPAICSPITNTTFLFAYSNDLTSTLIKKALGDVKKYIVNPKITTFANNRFDTSKEDPIHFHGSKEDFDNSVNALLPDSKLKLPSTNDGSNVLNLLRKFLEQPTCGAIVYILMKRLPNTVDVTDLIQELRENHISVFPIIDSAYIGSKDQSIMCKLAHTTNGFCDYQIASRLENEVYETMWIVSRVHVFVSQSYQVSGKGSIKVPSFVRPPQGNEKGSMGLVFTYQNHVRDTNFKALSFTIKTDTNTVVLKGQTNQNNGNALLAHPSLNNNVTYLIDINYEYEKTNTLEKIEPARHMDSISNLSKCFNYQCLDSLFDVIAGSEVTRDTESSREAVLVYQNVGFEEEESELPGSQLEVITTSALASASLRESDQSTPILQSTGQVEEESEVPGSQSQDATTSGPASASSRELDQSTPILGRIGLGQVLSSIREDIEPNEQTKPSEPPIENLPRYENLENLELSNEAISSSESCQVRASTSSRESPILESIGHVEEESEVPGSQSQDVSSSGPASASSRESDQSTSILGSVGQVDQEPNEPTRTSEPPAENLPRYENLEHLESFRVIRQLVPVYQYMDCRAEEPVRFRPQPEEIPIETPCLDFTVNFVLDYWKFILIGFFILGIVIFLIVFFTTSKHGSVAPTFGTTTSFATVTTTVDTVTTTEAEPMQFTISTTSMNTTVADNFTSPSVDGCSPITNTTFLFAYSNDLPSSLIQKSLGDIRRFIVNPKITTFANNRLDTTKEDPIHFHASKDDFDKSVNALLPDSKLKLPNTKEGSNVLNMLKKFLEQPTCGAIVYILMKRLPDTVDVTDLIQELRENHISVFPIIDSAYIGSKDQSIMCKLAHSTNGFCDYQIASRLENEVYETMWIVSRVHVFVSQSYQVSGKGSIKVPSFVRPPQRNEQGSMGLVVTYQNHVRDTNFEALSFTIKNDQNTVVLKGQTNQNNGNALLEHPVLKNNVPYQIDINYEYEKTNTXEKIEPTRHMDSISNLSKCFNYQGLDSLFDVIAGSEVTRNTESSREAILVYQNVGSPGEGESELPIPQSQVFSTPGAASGSSRESDTPILRSIAQVEEESEVPGSQSQDVTTSLAASGCSRESDQSTPILGSVGQVDMEPIEPTRPSAENLPRYENLENLESFRVIRQLVPVYQYVDCRAEEPVRFRPQPEEIPIETPCLDFTVNFVLDYWKFILIGFFILGIVIFLIVFFTTSKHGSVAPTFGTTTSFATVTTTVDTVTTTEAEPMQFTISTTVASKLTSPPVDVNSQYPMSSVEINIEVVPSEDSDESIFTYCRVFFFVMLAILIICIVLFVVFMSSKPAESSTISTRSKTTITTTSLPVQNCTPKTDTTFLFAYSNNLSPSVVEKGRDLIVQYFQTPKIVNFANIRFDTTTDDLIYFHSSKEEFQKSVTNNLPDSKLSFPSTNTGSDVLNVLRKFLANQESPLCGAIVYILMKRWPNTQDVSELISQLRQRHIFVYTVSDTSQSGGENQSLICEVTHGTNGFCDFQSTGYMKDDMYQSLQCISNTFQIISQSYEVTGKGVIQVPAFVRPYQNNGLEPMALVITYQNHKRDGNLKSVGYRILDDKNENVWMNSLKSWDGNVFLDHPVLKNDVTYQIVINYEYGGVREEKIEVRRSGVLVAVFLLYAFGNRPSVSRIVDVPTYNDDPTTEMEPINKQNYFTLLNQVGDSQFQKVILIGLLNIVIIGVFVAILFSLFFLNHKDPHTPPDNLSTAVSLGSTVSAHTTNTTLLIPTTSAPTTFATTISAPTTRTDTTTHSPSSDTTTYPFDHTDCSPNHKSTFFFAYSNDLTADQVLNTWTSISNNTNFFFETYALGRFDNMYSRLNETFSTFDSSDSFDDITDALLSNLPNPADSFNDPSRGGTVLGIIDSFFCSDVIHCGATLFILTKRFPTETSSYIDYLVSLLKKYHSYITFVVSENSLGGLSPESMYKLASETNGLCIFTGDDKIQEVWITVLVSKYLFFASDTLLATIHLAFIFVNAEVTRSGTLTLPVFNAPLLGKYHISMTLQDHGPLDTFRMVHLTWYNAGTPNSGSFEETLESHAGYGNTTYIMKGPFTLYADPYNMALEFEYSDNKINILQIRIYSVSIPDTCDYNYNNYCYTKLNFQFTFTDAQDACQRDCSSLSIHSKLENRLVVSMFNMTRSLLLGGVAPSENLGTRADRTTEDYNNLKLFDMELSCIHMHFQSDDWNSALCNKPSWIVYAFGNRPASSNEDPTTEMEEIPKQKHFALLNRIVDSQFRKIVLIGLFNIIILGVFAVALFFLLFLNHKDSHTPSDSVSTAVSTGTTASGPTKSSSLTTNTVSSGTTVSAHPTNTKPTGTTTTITRNPSDCTPTTPATLLFAYSNDISTSLIQHGRSWITDYFTSLKIVYFANVRFDTTTGDPIYFNTNKSDFTDSVSANLPDSTLSFPSQRTESNVLNVIRKFLSHQEYPLCGSILYILMKRLPNTKDVSDLIQQLRELHIFVYTVTDTNSFGGNDQGLMCEITHATNGFCDFQSTDMRNEIYQTVLEISRTYQFVSQSYLVSGSGVINVPSFVRPVQGNQSGTMSLVITFQNHGTDDNLKSVSFSMLDDQNNEVLSDHYKSTNGNSFLRHPVLKNDVTYNIVINYEYGEERKEIIEVRRMKYFITITVLLVFLFNLMESLTICPNGFDPSTQFMKMESCWKYMPDLLDQEAADAECLKNNGAALVMISSCETTHALQQYLRDKGVSRIWTGLTCNQTGVPTSCYWSDGRPSTSVLQADPGANDFAPGSPNVTVGECISYEESSRHWISTNCGVSMTFVCEKNDSNSLHNKSTNGNSFLNHPVLKNDVTYNIVINYEYEEERKEIIEVWFYSFSRMKSFFFTITCVFIFFVFNPTESLEVCPEGFNATTQYKKVHTCWKYIPHELDQAAANAECLKNNGAALVMINSCTTTNALQKYLRERGVSRIWIGLTCNETAVKTSCYWSDGRSTSSIQADPGYNEFAPGSPNVTVGKCISYEESSRHWIRSQFHVTLEREASAKSENVEDSHRVELEEENYQHVMNPDDPDESYNHPIRFVRNPRLSREARGEPPEYELGRETELEEEEPVRFRQPRQEEEQEPANPPEEPPEDPQNTPFLDVAVNFILDYWKMISLGLLALLILGLVIFLIVFFVTLKPAGHATVAPNSTTSTVTGSTVTTVTKPTAVTIPTVSTTTTTTTVTQAPTTTTKTTTATTAVTSLPTTTTTLSPAPTPTADPNSICTDGYTLFYNKCLKVITQPATQASAYEICSKTGASLVVIKSGGENREIGEFLKLQGLRKIWIGLQCNENDKSSCLWDFGQGDLTSYSNFVTGSPNIGYGRCVFYLYNESLPDAGQWGNGNCDVDQLSYLCEVPPTINDYCDFNYNKHCYTRIDYGFTFTDAQDACQRICSNLISIHSELENRFVTSMFNITGYLMLGGVAPAKDLVMWIDGTPRDYNNLKLFIADQNCMYMNYITGDWSSVDCASAAWPVCKRKAGARC*

>Cre|FL81_23076

LSPNFITRCYSFPRFQLNFQDARTYCHSQNKYLAVIHDTTQSSFLATIVRTRSTNANAKFWIGLTRSSANSSYVWDDGTPLLWPNFNPNAPQDGRYVVENSDNSQWQTETACVLLDFYFNFAQLVAASLNNASDFSGYIDTFGYNAGLSDHDYFSPYSYNEFKSTPFPIDSTDDEIDLDLKDVDSTLTTATWVPPTNDQTCLIFFSAACHFNPRTN*

>Cre|FL81_24087

MKPNVGRCRKRRLLEFPVVFILILAALVKPSTSSSKTPESVVTDVELKCPDEWIRLGTKCYLPFSIHQSWPFALTTCQRYGSTLAKIQTGSENQFIASLLSKPAKSSQEVKEYWIGLTVELYIWSDGTPTSRYVGFWRQDQPNFLNGTCAMGRVERKDLEWRLETCNLLRKFVCERPACVQGSYFCSSGACISESKKCNGYADCDDGSDEHNCPSAFHPTCRTSEKAENGQLSSPNYPNSYEPNLNCRHVLEGPINSRIELTIEHFETEPDFDVLTVLSEDICERSRVCVDGGNDARTSRLFNYA*

>Cre|FL81_24231

MMRKLSLFFLVLLFAVNALDFGSDSSSCEDDDGHGGGHGHGVFSGSNIEYDAASTACSNNNAVLSGIQNSEEKNYISRSASSHLSSSTGSLWIGAKRTATCARSKLTATCSKTTSFSWTDGSTTGTDGFSWLDGSQPDNALGGNQNCIVFFFARSNTVIARVNWFPGALDDVNCDAAQFNSIPQRKIQGYVCGKDASS*

>Cre|FL81_24443

MLIVTLILSAFLIYSVEGQCSSTEDSYIGDLCYTIYNQQLSFQNAQSYCYGLNKNLGVIHTTLQSNFLASLVRTKTGSNEALFWIGLSRPSMNSRFQWDDGTTMSWNNFDSNFPKDNLNVAESVLNGKWRTMNGQEALPFVCSYDPRKVTPGTAGPTTTGYYTDGSTTSTDWPASTQTQSSSSGSTSDYITDSTS*

>Cre|FL81_24444

MLRFILALSLIHIVAGQCNQNDRLIGDLCYSVSTQKVSAENAGSLCLLYNQYLAVIHTTLQANFLASIVRSQTGADKFWIGLRRASLNSRFQWSDGTTMTWSNFDSSLPKNDYYVAESTTNGKWQTVNGGQQLYYVCSYTPGSVTGIPVSYPPSETPAVSYPPSGAPVSYPASGAPVSYPPTGVPVSYPASEGPVSYPPSGAPVSYQPTGEPVSYPASGAPVSYPASGAPVSYPPSGAPVSYPASGAPVSYPASEMPVSYPASGAPVSYPASGAPVSYPASGAPVSYPPSGAPVSYPASGAPVSYPASGAPVSYPAYTPDNYPSYAPTVM*

>Cre|FL81_24900

MLRFILALSLIHSVIGQCNQNDRLIGDLCYTVSTSKQTPEESEGICLVSGRYLAVIHTSIQANFLATLVKSQTGADKFWIGLHRVSGTSNFRWTDGTPMTWSNFDSSRVFQRGLPKDEHYVAESTSNGKWQTVDEAHEFYYVCITRSPTTGKCFDNQDIEIQGECYKFVPLKLTFEDARKWCHYQNPVTASYLAYVPNQT*

>Cre|FL81_25024

MQQFQMIKQFILLFIIFIVGVHTTCLDPNEKELKGICFHYVKQKMTFENARYWCHYKNPVTQSNLAIVQNQFTANFLASYARSAFGINDGTFWIGLSRAHNWNQWSWDNGTLLDGWIMIRTVLQHSLQVHRLVPQPLQLQLLVNQQLQLSLIESGMRAFQLVLLVGALLVTSLAVPQRPAGEPASRRPPPPPSDSPKYLSNPSRRPPPRGTGTPPPPPTGEPQELIEDGNASRGPPPPRGTGTPPPPSADEPRELAAGNASRRPPPPPRGTGTPPPPPTGEPQQMIGDNNASRRPPLPPRGTGAPPPPPTGEPVE*

>Cre|FL81_25050

MRNLKQFIIIFHVFLFEIAFNTNRLNHVYKSKRLFQIVLYLVLIATLIISTFLIYSVEGQCSGTDDRYIGDLCYTIYNQQLSFQNAQNYCYGLNTNLAVIHTTLQANFLASIVRTETGSNEALFWIGLSRPSLNSRFQWDDGTTMSWSNFDSNFPKDNLTVAESVVNGKWRTL*

>Cre|FL81_25262

MLILLLALFPTVFSQCFLGDDAYIGGLCYTTSNYKTTYQTAQLTCHWKSQNLAVIQNSLQANYLASLVHRLTNEQDGKFWIGLSRQSVGSRYQWDDGTPLTWSNFDSKYTQNKLNVVESTTNGKWQTVDVQESHYFVCSYDPSKYSTTLEPPSTYYPDSSTVPPVSESTYYPWSTDYPDVSTSYPPYGSTDYPWGTTDYPWGTTDY*

>Cre|FL81_25310

MSCMNRWSNSSLATEFCQPKHDFILSAVQNTQGFSPPYQYHIGLIYASGSWVWTQPTGFQQVPLQQPLMWSSGYPQLASDKSAVMNQQSWLGTGWQNIATMNVVANYVCETYSCDTDNYCDENMNKKQ*

>Egra|EgrG_000677600

MLTRLVIWLSLLCCGIAHGSPDSNCIQSGSSRWCFTFYSEPMPFGDANRKCEEVNSSLATVPNRYVKEFVLNYLNKTNNRTETFWIGLHWSHSMLRWISGYPAVVDVFWNKSSEDLSFKCVVASADGLWDSRVCTEPHPFLCSSEVKDSTESVRFLPHFKYECPRNFHLVGRRCLRFFFDEMATRAEAESTCTGLGAYLPTIRSFRENDLLVAMLADHPSTFWLGLSYGANGHWWANSGETEVAFTNWMAGEPSGDISNNCTVIHTDLYFLGKWSEVNCLSTAHFVCEAEPKLVPAHADSHLHGGLFVQGRCSSGFYEYRSACYMIIDLPPEVTRTSSLSSDSVTMACQNTVLDVDCSPSNVGGVLCPMVASPRDQFDAAFLRSLVDKFAHPPNAAWVGLKADRSHVYSDIPKFLESTNLLNYSILNALSNSIVCFSLHADKTLLSNNPCDSHLAALCGYELDARPLLPSWPQIPDVKPEVLKCPGDAWKLFDTNCYRLEGAGNEGRSWHDAELACIQFGGHLTSIASMTEDNFVRSLLPTTQPLKPWIGLKVLIDAGEDGSTVVKWSDDSPVNHLPLYVDGHGEGDEECFQLGSVQGKQFWTRANGCEELAPFVCKTPAFPISVEWKPQGSDHCLPGSHPQFCFSVNHTAVKFADAVSFCQAAGKHVATILDDSQQDFVLRALRQLASLELGRNTIQRYWIGLLRSRGTLHWVSGYPAVPNVYWKRGAEDSKEICVYVDVDLGNMLSWGTANCEEVNPFLCSNTPPLPPLLPSPKRPPIVPLKTSCPLGFLYVNGKCYKVEGDSRNNFTFAEAIAKCSRSGGHLATISSMYEQDIISVLLAERSFPFWIGLNTTTEGRKWANGASITYTNWMEEYPKWLNPRNPLCTYVHNQPTRTDGQWAETACNTKMGFICETRPTEVQMQQMFTPRLFSQDDCASGFLHYRGACYMLLRPSSSLPGRSASQLYDDVSAACAATSPIPLDCEKARGLAGCPVTITPHSHTEAAFMRLLIGEVADGNYVTEVWTGLKILSNSSLDITQTEDRVPLGSLDIDFGHLNNAAGAGYTCFSLRRDHTFLSSRHCSLPAPAICGYYHDTHPLHPHLTNADDFACPTVTERVGRSCFYRVDPTMKMSWVEAEEYCMIAGHEMNAAADGSVVGHLPSVHDPFTLRFIGQLNGNGLVWLGLTSTQELQGSTNGSWIDSQFQWSDGSPVDYLVFDPTTKKNYNRAALIGNLKTCVALDPQSGYWVKVDCFKRLAFTCQFPIEAFPKTHLVSGSKYLPAVKQCPSSFKIETENACYVVVKTQLSAKKALAFCGTLHPYASLASLHSEKEERDLLARLVDQHLEKAYWFGLSQSDLQYAWADSSVVDHISKTGVTVESSHFWHFQDCFTFNPLRNGPNASLTAAWYETNCSAERPFICQVYRGVHGPPSLEPAHPPRSDLPPLQCPQGYRQHEDRCFRFFPTLLSFDDAEGVCRKAVEGTGFMGFLARISNSREQDFVAGLFAAEAPRQPSMAWIGLRQGEKRWTDGTEVTFFRGSLDFPVPVPRDEESMCTLLLYSSNVHFYGLWSRSVCSISDQMYFVCQAVPISPTAVTVDNTAKANTMESTALTCADGYTLGFFNRTALALGESLSPPHCLQLVSTTPMTWQDARSLCKEQSASLPSIDTLADLSFFRAWLQTPRSLGGAGFPSNAAVWLDLRVPACPQCYSNWRWHAGEWDESPVRLTDWFNAPPDPSGCYLFGVAPPARNSTIASHLRSIRPAISCTNISLPVVCQRNLRLPSNSIGFAVNRCVRNPTPELSNVTAFHTNHSVAISGRACIRWDLVQHDFNRSDAVLPRNWRIFTAEIAYSEHASPFWEEHCAHLAIQDAAGKTIYRYACYTSTDPDSGLEDCDMDSCVTSLIPLGWIIFIALTCLALSVGITLCVVRVWNRQRFFLPRGRWLSPIKMTASSSVFGGAVAVHRQQFQQHGVLYSNGDGDPAAPYVRMFDNPASLPPLHSLVPSLTFKHNVYRPLHDQNPLLLDEPDNADEFTGPQMVNPEATALLLVTLTMPRPLTVPFLHDHKFLAYDWLCWKRF*

>Egra|EgrG_000176400

MEELAPIFSVVGHLEPKKFDCSFRDGEVRYYHEGNPLLNMDTVRVTIFFFRNNHSIIQTADIPVDIMDLPEKGGEMDQTFPRPRIHGPLELNVKSIKAASQAISASVLKIDYNPEEEECCLSFTRPDILSPGEQRRKQNQLESQIGRPLVTPWWEANWGLWTPRLGRPSASGPAIGASRRWPLFGHIIAAAFNRTAVDHFEHECHEALLKGYRYLHRKSDSSEADYIPLQVNIWRKVPDGGREFVTREARYLRVSIAGARKLEPPVIRIFRQVNLTHIGGSFSVLPKDCIHVVNPDLEDLLEVNVTKVQGPLYAQLVNLRDPTRPVLSFRIAELRKGLIALQLLNYAEMIEKIFTLTLVAIDPFMQVSEPVNLRLVSRLQPVFPTRLLGSSMGHKPFVFQVFSLPLFSYTGALSIVQRYNLQVVGYIDESMVRFQVLPNREEEEYFKLFNTTGMGGGHLQLLGMPLVDGGATTPTTSASTASWTLNEIAGQQLLYAHPGSLHPTVDRFRLHPMLDIQRAGYHQWMRVARQRIRRNAGVSESELELPVRVVRLYENARESALKKDITINLHHGAAHCFTSEELITPSALEAVRSDFIDLKFHVKFHPSQGRLMRRSMLLRFAFNNSTQQRRRKPLISLTKEGVEAVDGDGGVGTADAKGISQISVISMIDLEAEEICYLGLRRDSRADMFGLQQTGQNNFPTVAVNINILPKPKYQLIRRTDPNQEPLEVAESVNFAVLESTNLCYVVRPTRQWLRSSAHNLIPLMPNTTGVLYSLTSQPRFLITPSAATLESSTSAVSGQHGAADAGRLVSLATMSASQMEGDMALRPGLTNLLRELEPTGLSHFTQAQIDAGDIVYVPPVKDLGPSDQIVEFRYTVSAPGITPFNEESFQFKVLAEDNQKPDLKLLKSLEVHRDGELMIDNSVLYLSDEDTYFDRLALRVTRAPKHGNLFQMMQHPMFNNTNANYKTTATTAPLANRRKISEGEEIPAGLMRAGKLSYKQDGSNVKEDSFNLTATDGRQDSEVVNLKVAIRPRVLLEPVWNLLVNNSIIVEENSTVMLHPSVFPPLTLPAMRGPRFFVVVPPTKGKLLLDKKRKTAQFSTSDVANSRISYRHGPAEIGTEDKVDLVRVWDFKTGKIFSLNFTLLPVNSQPPTITALAPLQVNEGSAVVLSHQIISVRDPDTVDSQIKVKLVTHPKWGHLELRPENNTSIGSTEFAFTGRDLITGRVFYANSRHQDGLESVSDIFSLRAYDEMFPSRESTPIHVSIHPVNDEVPSVRLVEYFAVPLNGRRVLTPYLFTISDRDVPRDILQISFPRLPRFGHLTVHWQHGEQYTITEASAPIAESYLGMMNIVYIQNGSVQLPARDSFTVSVSDGLHVVKKSAYVLLRQENRYAPEIRVTEEGGLVLEGLAWRQLTSVLTISDMDTPGEDLVIIVVRAPKLGQVERLQRQDVTGVPAQEDLIEAAMDQYEAEVDGEKRNTRNLKEGDRFTRRQLDTGRIYYTYTGEYTQAYVYDLITLSVSDGQFEAGPVDLPIRIRATKGRTNPMVQSQSSLYELLDKGRFDSDTLLSDAASELWETKEQKTEEPSIFLDNFKVTVAEKIEVAAGERRTLTVDEHIRIEPIGVDGSLENVKETLATANFQHLESEMSRRGCILLARANTSSNYALTEFTYKDVVHGQIMLNAESCFGKSPLEGAISLLLSIPLQKPVSFTIPIRIIGEVDHFPRPTVAIGQPISIIPGADIPLTLTHLNLFYTGMDPTKVYFYSSNGRFHWKHDCNVTAKVFSFFNIIHDDIVYHHTADATAPRLILVNLNHLEVDFNAIQIERFIESIIPLTESSPLLQYLRADSPDHITSEPITLRITTDDLYTHVISSGRIFIRAPGPEELTRVRAGEMGFFLTPKHLLASDASVVYSLDTKMRDKWVIIDVKSGEAMNRFTQEDVNRLRLALVVFSHPLQTKITSFGRQKTRIETRVNVGLSLFEPKSRATENITLSVYWAQVGFDRKRYRICVSSGVLALPLVRHGALGHHVEVYVDEVIKQVRMRVTLSSEMSSEKQFFTVAVKAPSGAVLDGNSEAHIVMLGKRKCRNSPYFTAFNTSNGVGTGDEVGHSRRVSLHDWLASNVIPDSNALNKAYLISMQRRKRAPAFSCDSSWTLYQSRCYRFFHHENLTWLEARDHCENERGYLASVPDASTSTWLKTLLGSYSSRLWIGLHKPVANGAWLWHSMEQTNFVNWESGYPKNLRGKLPRQTSHLRRRLFRRHPSPPLKTAEHTVWRSTSAYGYSRSPRSASNGWHQREALGQELGLQRLCVNLDSGRGMVWRNAACVRMPQLPFVCMKNPN*

>Egra|EgrG_000699700

MPPDIMKLIILLIYLNTVPPTLGLGQCSSVWKAFTTPSTLHTGVGEVKCVDLCYFKSYTKLTYAEGAALCNSLNTKMVYLKNEAENNALKSLVDEPIYLLTRVVGSAFFNDGDPVTYGKWDCDIFSKKDNRCVKRLKSGFMTTTSCNERLTVLCDLY*

>Egra|EgrG_001157000

MRILAVSRSEECPNGFFRISNSCYFVYNGTKELFSWSEARKQCKGLHSDADLMEIHDQSTQDSLNVVISLVAWPVWIGLQSSAAYSMDRSSYKWITSNRTPNYSNWAPDAFSYLEISSKQGCAFLSTANLRVGAWDHSDCDKTRIGFGCEVPLNSVSSLNSTIRLEGAQPAWTKENNAAGQICYLGTCYRLLPSFPMELGFDEAKELCGDAAVALPRNPLELIFLVDRLANASVSTRAWIGVEVTNTQDRLEFPRTSSVTPWLTAAIHRSHPAFLIPPSNQSETFCLQVHVNFTSTSPFLVMPCVGDDDEEAEEEEDATLVTAFCATPEPSYPGSCPDGEGSGESVEWHAFGDKCFAVMRDCEGDSVPASLKTPELDAFAAWLLPSNLRSDDEVLIGGIVNTTSPLTITWLDGSVTEGFNRLKPSFLQHPVSRDGLCLAIQPGSGWWVLRQCKGQSAPRLTLCSIPIKPQNHTTTIGPPVRESEPMCPEDYFPHHRNNGSVVCYRVVLGGARSHGHDWRAAERACRAVALGGDQKVRWRGNLASLPNKETSDDVIALLQQNAFTVGIWDGTNNLPAHMLRPSEFIWLGLSKSEDKLFWNNWTDGSTGNPTFYDNRTRQNQSFFSDSSGYFFDYGTCIALHLLSGYWYPLRCTLDLGYICQATLAPDSETEGGVDHFDIPSCLISSKSRNAGPSVETGFIHDPNVDCVVPGFTYFNGQCFRTFHDKFMTFAEAQAHCHDIGSPYSRNGKAGLAIFRSEADQLFVASQLSRLPRMPASPGFYGRGSAIYDRLEVIWRRYHWVGLFHYRHTFHSVDERVPCYIAHELNRPLASSAGGPLCVSITGVPDTEHFASLTFDIGCNESLPFVCSFEPSPTRLTTKSSTSVCPKGYATHNAATGDHCYRFMGEWMGSYEEATAMCAQTAAGARLAALTTPFTVAWMRAHLPSDYTATGMLPWESVPHWIGLKLRARDESSWRWANGLPVTHTRWSSQPVDVVNSATDMCFAFAKSISMTSYELDMITVNCSRRLSPLCEADPLVELPISMSKPDESYLAADQHDYIGRLAKSASGKACIRWDLISPTNTSEMDALQRAFHNFTLAAVAAALDVNSSTDGDIYSPAFQPVFSSVCNWCRNPASLRDHAFCYIDVDKWEFCTLPPPSISSSPSSPSTSQIRTTLPFSVIFSIVVIFLGVFFLFPLCLLFIFCLHRKSRTGWVSTSPVQEILLQRLPFSWRWRWSRTASQPCLLADNVSYSAGAVQEDTIRDPAA*

>Eve|EVEC_0000124501-mRNA-1

MASYAFYYDNDISIYILIMTTLLLTSTLLFYYEFEISIYMRTHRTQNVTKNKTSSFHLLLPSKMQIFIPVKILKFINVFSSVSIHGGSRIAKSFLCITIRFFTFSVAEQLAKNQFWLGLVKKSDNWYWSDGSDFSYTKFLPMPSVDEKTEMCISGSLSGWKLEESSVKVLSFLRFGVDYKYKNLYINVLQLPMKVELGIAEQICLARDSSLVWITSEAEQKFLNGELNL*

>Eve|EVEC_0000124701-mRNA-1

MPTFDPSEFSEEHHIVVFNKSRLFVVNSSIYALAICKFPKFDRIQCPTKWQEYNGNCYFREKNVHSREFAKTVCQQKGAHLVSITSPKERDFLYDLARKLPFHTGLTTRHTEVPVFLTTSRNLFNYVNSVKVLDDAKGKTYPIQYKKEFARMTTITHENQSHPLCEYRVYHCRHDWYPVGEYCFKTVSISNSFWLKNYRSIIVVIKVRTALFYDDAVKTCIANGAFLASFQSNTEEDIAKDLVPSRYYWIGAQMYNGKWVWQDGSEWRYGKVKLEDDYPHKLPVVADRKTKFWIGISPNSRFPALCKYHGA*

>Eve|EVEC_0000135401-mRNA-1

MIRTFFRFQLGFEVRAFLCFSLLLLFTEVYSNRTSGEALEEGLCNYGYQNFQDKCYYFSCLNCRKNFYDALQHCREEGGDLASIHSEEVYKFLKNQTNNFLIGMRYDSGKEQFYWSDNTTINFDKLRVDFSKWHPYFGSCVRVNDGVWENAVCERKYRWLQSPGYPSNYHVGDSACYFKLVVKVDELIVVVFNETDLFYNFDNTYLEEDVIEVFEGFEDNERLIARFENAFNTLQHVINSHKRFYFTYTTTNCPIVEL*

>Eve|EVEC_0000159201-mRNA-1

MVIGKAVTVKTCPHYPYANYLGIPRMIEGYKPLKSYCFYPVSARVANFYNVNPENEGKISYAITTLWAPQTFNQADEYCQTRFGGQLASFKDARERILLHKAVFPSFDERSHKSLGFLVGLKSYFHAYPIFTDGTDGSYGFNRSETESDKLPRCHGIVRSYKYPIDSMQAVKCTHFFKFVCKGRIFSIPKEVQKYMVDDTTKQADSNDICEEERELLYFFHATKVVDIRDGSSYCIHDFFVSRKVHITDFQEAEELCRTHLGGHLLSISNSEELRFLSGLLFPPLNGSMVELELRPLGLHYSHGDISNFTDGSNGLYAIKEGYKEKNSLDLLGKCFVFVHLYNENRSAIWRSNCKAETVYKRITCKVRNEPISNGKSKRFAASGIGRDSTQSKRNWEQFLIMGSIGAVSGLIIIALYISRSER*

>Eve|EVEC_0000169201-mRNA-1

MKELSSDLTARFSRQILAVQDRIEYLEKELDGLKKAVTYDWKRTDSGSLYNVFFINKNFDDAQIPKSLSLYFPSDILTDALNETEIEQEVWFGTKTKTSTMSSPDSTYSNFDEKQPLKGCTSITSDGKWHIKDCSELKPFICQRIFLR*

>Eve|EVEC_0000203301-mRNA-1

MQEVLKPMTFEEAERNCEILGSHLASFSSVQQFKNIRNRFPRTNWWIGLKQVNQEWAWTDAKNVDYGIWSKYGLSKIIIDVQKCMRATERGSWERSSCELDSSSLCKLDPLESCRPGFNEFQGFCYNIETKRVAYEVAQNFCLANGADIVWVTSSKEQGFLNGLASSHPFWLSLVLESGSWVWKAEESPSFLNWKHGEPNHCCPEEEASNAFSEPSGWSDTSYLSKYAVVCKYKKGILLNSEVDFESDSSLSRRSPEDQSRLIAILPLSPFYATGIVLLVMVAITITAILCRKKPVVIVEYAKVPEIFEDDSYSAKVYYHNPTIIREYDA*

>Eve|EVEC_0000203401-mRNA-1

MVYFQGPRCVDRSTAADFCEDHGGILPTIKTDEESADALRHSENGFWLGVERIGSRLTWLDGLNSSASLFVGPKKDSGNFAVMTTNGHWRVVEHENETCGKPVCFMRDVVTSTKCPWYSEEENYNIYDSVVVSESNGKWYYCLIDVGTKDGKTTTLASSQKYCHNAFKGRLLSFPPIREYIFLHGHFYSSIKRGKPRVTLGPVEKELVQHLADANINTLTMAHLADTFFVAMESKTDIKYLCRPLWTYLDGVCYKKGTKKLTYSQSSEYCRTMERSRLATLYDDNSLVKFLDIPNDKRQNYWIGLDYQDNNYFWGNGRQYTLKSLKKPQPNFRVAITPDGFELFHETSKFLPICEYKSSILLTDSGTVRRFACKKYFKPVEELSNESFTGLETRSLRGQAHYG*

>Eve|EVEC_0000203501-mRNA-1

MRLYQLLTFQIAQPVIFEDAERYCEVLGGNLASFSSVEQFKRVQTVFPGTKWWIGLKQVNEEWGWVDSQTVDYNTWGSYGLTQEIISTQKCICATERGKWERVSCELELASVCRLNRITNEVKHKQLIRKLQGVERVEYETARNYCIAKGAELVHVASSNEQEFLNDLSDFHPFWLSLILHSGEWVWQFGEQVTFLNWKKGQPDQCCPKKEATHAASESGVWSDTSPLSKAAVICKYRKSALYKNKIVLEPDKGAVAKRIVWSETEKLLAIDVSPMYIIVGVLAISVALISLAIFCKQRSLTMQYVQYAEVPRYDEVYFGRY*

>Eve|EVEC_0000203601-mRNA-1

MQHFNKRNKISCKAEQNLQPTVQHDKGLDTSRKCPWYAEQKIHEIYSSEVIAENYGKWHYCLIDVRVKNGETTSLDNSVKYCHNAFNGRLLSFTSIREYIFLHGYFYSPIKRGRPHVTTGPVEKELLYHLADSNITTLSVDPAVETFLVPLPPETTFFFLRDTPNFIMVFYLPDVKHLCRPLWTYFDGMCYKAGEENYHHYNAKKYCSFMDKSRLAILYEEKSLHKFLSLATPGKRYWIGLKYEGDRYVWEDGRPYTLKPLPPPSNSEQLVQLTSEGFQLANKGAIAQPLCEYKSSVSLVDKSSIQRFACKKYFKPVKEFEDETFSGLKSRSQRGQLHYG*

>Eve|EVEC_0000212101-mRNA-1

MGPPDGYPASDAWIGLTTYGDRRRYFIDGNACPFRKWYPGIDPENRPKSAHRNKNCTLIDSEGEWDVEDCNLNDTVRAFVCKKRAGFRKWQPPKRLHL*

>Eve|EVEC_0000212201-mRNA-1

MIDREDASNCHCVKLDLTSGKRFWLGLRKSKSNGNWSWDDGSEYSYKNWDIGEPNSCCPMDPIPIHSYVGGNGLWYDTGELHYPLPDGYVCKYKHQCAEDWNIVWNRCYKYVNATVNSFTVPETYTVASTICQGYNATLTSIGSTVEELYLKTLTGGVPFWVGLQRDSDRWFWTDGTDSASLPWKSKEFSNCCENDEVTVIYNQGLQVVTVEDWQTGFVCKIDGDADYSPPAEMHIAKTILLPKKLEQQYPSATITTDSKKISDNCSFEN*

>Eve|EVEC_0000217001-mRNA-1

MINRYYLRENLDRNFPLAQEINEPMAFEYARAYCRAWHRGQLASFLDSVQIETFKTLIREGMKAWIGLFYNGSDYVFDDGRKYWENSQLKSKVGTPDYQACFGVEKSGNLGATRSS*

>Eve|EVEC_0000217101-mRNA-1

MRHNVTKTVSPGRKCMSVDGIGNMIRENCEREMSFLCRFEQKLIIVIILFATFYQLCFCNCSEDKSPKAVEWNVARKFCFARGSELVWIDSEAEQKFLNTLAGMDPFWISFVKNGDKWEWLYMDNVPYTKWKSGEPDQCCPVGKANRAFSAHNGWSDVGPTFKTGVMCKYEIKLPYFCTYFKID*

>Eve|EVEC_0000217401-mRNA-1

MNASWFVTLALTLFLAIGKLRAACPPGKTGPDCDIPICDTPGSVEHHDGMEGYGDYIFWEVSFGCNQEYPVNIDEYVNDITVSAYGNGQRFSCGLYKDEKLIIPSDVSSTGTGWESGHYDGIGLAHGYGQYTVKLSTESTTQCEISVSAVTAVVVNGGFITDLSDSNVQQLAPRPHQGVGQYPKVNVSTYFAFEVSDALRRPGSATSVTTYMAEGFEHTELVSGYDTMYNQWDRVYPFMCEGGTSPTTGPWVPPTECYNGGILINGGTQNAECYCGYYYEGKSCETIRCFHDGYAWAADSCACAEGYSGTYCQDVNCRDKSYVDGLHYRAIVFVIRTGRSMIDHITQITTAAENIASYYALIDPTYIQQWVLVTVSQGTVKMTTYDSSIVFISAINTLPTTEDTICNDVLFEGISAYLSSSYAEAYKRSSIFVFSDGLPDDSEETRFGLYHQFSFFRGQIFFILADSSIGRCYIDKSAEEYRSLRSIAQFTHGHVTYVDINYISQSKLLEEKRIPSEMVEEDSLQFILFNLAILFEAENLVVIMLQIVKRSMKINVCSIGTLFGIAVDDGKAAYLLAATMHQAVQVMTNDFLDSCLKAPKYQSFFIDESLKSFYVTGVGDSLVATVITPDRTVLELDTLYSQGDLHIWKLENATVGGYLLSLKSAGAGPCSIRVYGSAETALWFGTTDNVNDDFELTLPTTTKEAHIVAYIKGVEIPDTFQTHAEATLWSNEGPEDKRVVKYASNGIYRDNCAYNLYFGAYQCERPFSFVYINIYMTSKSGSIVQRSATAYCSTTPPTSTPQTDCLNGGLPVGINNTCLCPAHYTGDKCQRIQCLNLGTAVGGTCTCVNGFTGKFCQTASCTRRNDEVYFIPNGRSLSLIIQDSISTRSAIKAISNQLPGLYQDLIHQHKDWISNYQLWRFNDTTFTNVMSSSNPNDFLNGMKTLKNSNAEHSDPSCRNLYVLPGLLNALLHTGLTYDGIMYLFISGYMKFSTTDMSASAAVYEMLQATRVKLNVVLMSPAPCGQDLNSPESTVLMNLAAFSGGEFTMVMPPRAGNVIRSIPTLYRGSYIYENYAVNCSQQQNFYFPVDSQMQSFTAYILGDLDQTSIAYYQPNGFAKAPYTLWSDIGMGSRMDQIIKECDDGWELLSNRCWLFVLRSQPWDVAQAMCHANNAVLITLFNEYEQSYVNQHTMKETYWIGLRRGSTGFVWDTINDKQLTLESTGYTNWAAGEPTSDSNKICVTDKAAVGWKMNDCSERHSFICVKHAYNADYEPDAAEIGERLARGIWKLSMRTLEGSCYVQIYSQSGIELYTKFTTNVHDDFGEAQPITNADDNRFVAHVTNQLSTSTGNAGELKYAHLYQENLKIIQAQTYSRRDQDKCSYEFISTSFKCPADNFLSMSSGIDRFGYLYQRIQPVACYSGAVAEDCINGVYHNGRCICEPYYSGDFCTIPSCQHGTLDSVLQHCTCSDEYTGPTEYEKEYTPTTDERSIMNAVSRVMESDFIRYRSLMWIVTAGVPEDANRLSLALDAVARRHVYVNFLIIGDKSGPANVQYNDSRLLACFDLVTETGGQFYQVSEEEGKLSKFWQGLLSELYDVYYISTKRLISCPAGIDEYIQVGSKTITLIVDVYGQGTMNVFSPNGTVASYTKAASSRTNLVVVVEADVPGLWRIVITGNKAFCGISVRGQSDFEVYVAFTWDMSTDNGLHSNDGEAYPVAHGMPNALVAHSSFGKLNFAQVFSNDERSLLWSSPLVARQNCVYQYISTNVFICPQHSLAVSVDGEDENGYPVRRMFHIHCVGTILTIAPLPTITTRQPVITTGYPTIQPPDCDKSGKFDMVAVMQELPSTVPIDQSHTRFILSTYTDAYHTVNPNFEMIKSSDDFLKYLDVLRRVEYEGTIGNNIQSIFDVANNLTWRSDAVKVIVFTSSMGWDKSNTGQPFPDPTASATAFKRNGGKIIPLGFGPNADQSQLVAIGNGCTKMSMNLDYFHSVIIPWLSGSICNVAGALFEILGIGLERISFRSTSRLP*

>Eve|EVEC_0000268101-mRNA-1

MKIRSYAKNECEKTWIATEDYCYSKELRRHNFDEAQQVCKKKGARLPVFLSEKEFADFRNMTKEPHDFWIGIRQQTRRMWRWLDGAPLHLSEISLPISFTDGFRCAVLSKRGVGFRSFDCPSYWQEFEENCYLLVCFKAFNEASIFSKEEEDFLKELVGDKTAYIGLDFMYLARDTYLPVEFELANEYERVFIKRAYFNVRYQECLQLDWKATMMAYQACEHTAEFTCQKESMVMWQPQFLLTEDISKSIYRERNEKVRK*

>Eve|EVEC_0000268301-mRNA-1

MLLGLQVTNKFRKESEILSTGNQTEMYIKADKFCINRLNGRLLYAENVDEFKYLYKIFYEHPEFSRIPVSMSISNNELNVYAETDLLNENIHYGAGVHQFLVSSENKGKYWIGATYKSGKWVWEDDEVLDPIKFSLTSGSSFECLAVVNGKLQYYRSCSENNEFVCEYSSNFNYVKSNEIKRVVCKGYYNLAELQAAIEDDTEDTVELEDTTDSGMLGYLKEYI*

>Eve|EVEC_0000275201-mRNA-1

MAVAFVSELKSVEQERSCPKLWFEFEGRCYYHIKSKLSAHKAWEKCRRHWGSDLVSIHDRYLNEFIANLEEDGLLIGLYYNEGKNAYQWTDETPVDFVSSGT*

>Eve|EVEC_0000280501-mRNA-1

MFRLLEVLALVLSLRGSGGTSDCVGNAIYNSHINKCIQFFSTAANWLDAERTCEKLDGRLVSIASTYEGNFINAEAQKNLPSNDYWTSGSNILHENFWTWIDQSRIIHTNWDSFYPTYNDTMMCVYAMKAGGSWRTDSCLKSQKAFACQIKPLTKVDLTEWTYYEETKSFYKPLSSGFGWNRYEEDCKTEGGHLASIHSELEATIVIQVGLGIPGCLQWAIGFKRVTPESNFTWTDNSDDDYTDWRGSNPQDNFQCTFVRYYDEGWMSYQCYSAILNSCAVCKKPAK*

>Eve|EVEC_0000290901-mRNA-1

MSFTIRLKSFKFHRYFQFELQAKMFFIFIILSIGTKLSRAQSCFNGGIYGQQFNKCYHFFYSPLIAEEAEAVCASIGGQLVSIASPAEQAFVAGSTLSVSTQLKSVILVPSVSNQKLKNGNKGSQQLEANNCVAATLVHGNWYNTNCDLATNYFVCQTEPIVVAPASKLLI*

>Eve|EVEC_0000291001-mRNA-1

MYSQVALVEGFLKAIPTMATKSWEDYEKECTAQGAHLASIHSGYENSFVYGIAFGDDLCQYAAIGLKSVSGNLNSFQWNDGTSLDFNYWDSGKPPYENKFCTFMRYHVKGPWYNFGCDDTAWSFCAVCKKPATQI*

>Eve|EVEC_0000292601-mRNA-1

MRQAVIFIALSVTFTQAMVCPVGWERYTGTADTCIAAYKKEMSWLDANNFCKSLRADLVSIHNSFQNLHAATIAEKKFEACRYWIGLYNIDKDKKFKWTDKSSVDYTNWLQGEPPSVAEATTVYMSLQSRFRWVTTTEVAENNCFICEKRFNEPSTNPESTTETFESPSTGAPTETIVETFKPNYSTEKKLTTENPESSSTGAAPVETNAGTSKP*

>Eve|EVEC_0000337201-mRNA-1

MREEMRQYYKSLIDEEKDATHFGYGGQCPTNRNAVLYSDGTQFICLGLVRDSDVSNFEDAEAFCEKHYNGHLLSIRNLKEHDFLLSILSQTRSITDDILQLIPLGLKGDPNMAVAFTDGTDASYVLEWYKPYNDSGNFDDYSDKPCYFLMFSTLAYWQGRQKPPAETFVIDWFSTVLVIVSGGVIVGSIIVVLYVTYVTRALPYVSKGVLSDYGVLVKPKVVRPYEEISDKSSRTYDIADSGN*

>Eve|EVEC_0000346901-mRNA-1

MGDVRFIELDYSRKCPQSFIQGELDVKQTRVVNERNGRWRYCLFDVNVKGNTVTSYESSRKYCSSTFHGRFLSTSSVREFIFLQGVLYNKSEDGRPNVVIAPVEKNFLRYLADAKVKRMSAEYGRFKFRVNIEEKWYFHDRFCRTWQDATLATFYDVEHLHRVASMQQVTRNYIGVTFNSTQNRYVFDDGRPYFSGGPDNVKHFCHLIRRTYTAITECKDFHYSVCEYKSQLLFTDEVSIQRFACKKYFDTEEAFEKQINQPKNFEDSQRYCETVDSTLASFSSEQQYKMLKERYAKAKYWIGLKQVNEEWEWLDATTVDYKVWYLYNVTKRKFGIEKCMSVDEAGDWRRKNCEEKLNFLCKFEPKLWCRPGFTEFNSFCYKFFNASVPWSTARVTCRGYGSSLVWITSGREQKFLNEFTESKPFWLLPLFYSGKWRWPFSKVPKFTNWKRNEPDQCCPKEEILAARSESGKWSDTSPLFKGTTVCKYEKGQLYVGSEEETYAAIVKKLETKLGLEEELPDEEVPTDTPVN*

>Eve|EVEC_0000350801-mRNA-1

MTTGKKNSGFLPPNSEFILGVARNSDHFYDGTDAKYARKNARRWNAAASPSDCLAVTRVSIGEGFISKNCPSQSGDRKTDKVEIRNIWYISAENGRSKYCICQDTEAIEKSHKEEWQKKDDKKKNSKNDNRNGSSGKITTTKAESSRRFDFEEAEETCITKYGGHLLSVRNAEEMMFIIDTLLPGGAEYEKQKYYIPLGFRLLSEYRISRFTDGLSAEYIVKRADNSADFRSSDISTKCYALVRDTKIKRSSVEIIPCEGEEIWKGIVCKTLESTLPDDVYSLFHPFLM*

>Eve|EVEC_0000353901-mRNA-1

MKYDKAYELCWRQHSVLAYIEDSAEDEYIKREFGNQTYWIGINCQKDRWLMTNSELAFHTNWIHRQAKGCPLNEKAVYETDYPGGWNITDKNSSHYYACKHKIVWISATLAILLSTAIFLLVLRLCLKEDDTETIMPKVIAGTAAHTCRAPVPDVTAPTTMIPTTASAHTITPASTSLSLITATTTIPTNATTSSISTTTMTTIPTTLDPNQSTSLVISLTSLSHSESSTSDTVSTTFASSELSTESSTIS*

>Eve|EVEC_0000358901-mRNA-1

MPNMKYWVGLQRSGNDWQWVDSSTSVYRNWANNVTPTRGCVVANAPGTVAGSWSGMACNQRYQITVAATVFNVYLFATTQSTTVI*

>Eve|EVEC_0000376501-mRNA-1

MVYSRIAIISAGAKATVVANLTRSSSELHKLIYQTNFCKADLFNFTDAVQLAERIFADDRTRSNVPKVVVLFSSRTITCLPTSRCYILRSAKNYEDTYGCSLVAKMSRTYGVTFIGLSVGNHGLNMDFVRGCSLIPFDGFHSYSRIYRTLCYELIVANALKNFKNSKDFSVNCNCETKSLSQLSFGGSDVCSRRNLCVESRNLRLSFDDAAEHCRSRGFSLAIPTDKHTSSFIEGQITSRNKAEEYWIGLRKHGDKLYYLTSYKVLSEVGSLQTDWCPDANIKNDGCVYRRRCQARSGYGWDVGPCGPLTPLRHFACETTACSTIKYCDDQPGREYCRKGESDALRC*

>Eve|EVEC_0000382001-mRNA-1

MSKTETGTGEILTSVSNRGFWSGHEKINGSWRWLDGTEDEGDRRCADTEGPSNERCYNELYKRYFLFEIVPASKNLRIFGEYPLHDDIFGAAVYFLLNCATVVVSKVKGKKSISFEDSQKYCYRHFRSRFLSISLVREFIFLHEQLYGSSRSEQPNVVIGSVERDFLRYLADANLKRTSVKYGELKFRLPHPKEGMNQTINHYIGFKKTKSGYLYDDERAAHYLSHSESRECHRFMMDQYIFSSCFTPANSICEYKSQVLFTDEKSVKRFACKKYFVSQETSGRQSVPTVFKINEKNETFKNVQRYCETMDSMLASFSSKTEFRTVQEAYAPANYWIGLKQVNEKWNWMDATTVNYTDWNKYNVTEEYDKKVDWSVAQGICIAYGSYLVWITSNDEQNFLNDFTESNPFWLFPLSYSGNWTLPTAEKSKFENWKAGEPDQRYLYGQLFFAYSEPGGWNATSPSFSAVIVCKYEKGQLHRRYPEEQYEVALNESRGSGRTSEVASKTKYTTQQSKESREPHASTVRQSEGGTLAFQALLFIAKD*

>Eve|EVEC_0000406201-mRNA-1

MEMALAALLPLLFTSVFCVSASNEAVKAGNSCPEGWTGNNCDAPICYEQKIIERHEGGSYGDYLFWEQSSRCSGEFYFYVDEFVQNITLYVEGGENFFGEVRKNEKRVQAHGTPPVVSPDIHVSEFDEIASFYGYGVYTIVVQSDGTESCTIYVRSATSLMIDGGFVQDRRDDSAQYVVPQINQGVGRYPIVNNNSYFAFQYPVALSHPGRANFVYINSGSGYGSRIAVKDRYGCGASYISNAVQCEEDLSLTYHLKVEGVDTKGNTWQRVYIYMCETYATPSPPPTEPPPEQCLNGGILVNAGTAQQECYCGNDYEGRMCETKRCFNNGQVGKYGRCTCAVGYTGESCEHVFCQNPTEGYGDSKYKAIVFIVRSGLSMAGHIEEIAGAARTIASYYQSDSYIQRWVLVTVFNGMVDVQSTTRADEFVNAIRAIKIEQDTICNDAIYTAISTAIDTPAVQMYKRSMFFIFTDGMPDDNTEERLTLLHQLSFFRGQMFFMLANSVSGRCNIDTASEGYRGLRYLAQFSFGQVTYVDLEDLGSASLLTLLFNLKMQWATVVLAITAHKADHIFSNDLMDSCKRAPKYQSFFVDHSLRNVFISATGSNLNLYLTDPSRNRTEATLFYSSSDHLYVWQIENLMYGGYLMELESQSYNPCSLRVFGYGDTSLWVGISTGLAEDATVTQPIYQQTSHMVARINDVNYPDPTQILAEATIWTNEGTSDKRTVLYASGGIYRDSCNFNLYFGEWECAYPNEFFYMNVYVTASNGAVIQRSYTGFCSIVETTQTPPGQCMNGGVETTLSNGTQTCICPAHYTGKKCEKIQCLNLGTAIGNKCECVSGFTGQFCETSVCHQRNEDFDFGPNKRSLTLIIQDSISTRTSMRSIKNQLPSLVQDLTHQHAEWISTYQLYRFNDSTFTKVASTPNANEFLQGMDALYSANLAASDPSCRDLRVLPGLLGVLMNHQLTLNGVVYLFINGYMKLDVTTSEVEAVYELLERTKVMFNVVLMSGSPCGRDLSENETLVLMNFAGYTGGQFIMVMPPRAGSVIRSTPTLYRANYIYETYKENCTEPEPIYFPIDSQTQSFTAYVVGDMEDNAIKYYRPDGSKKEPFTIWKDLGMGSRMDQIIRECDDGWELSGNRCWHFGIVPRTWSEARDLCHNNNAVLITIFNQAEQDYIDSLARGEPYWTGLKYDSTSSAYVWDTTEGTPLTVFISKDDFKLSYNKKTRHMALSSTGYNNFPGGNVPAFNLTTQCVFDKHPVGWSLAACSERRSYVCVKHACSKNICVSKYLEKNVVSSTSVANSRISLTSAEETNNADYEPDFQDLGDLLARGIWKMTVQTISGACSVQIYSQSAIQMFMEFSSDIHSDFGSQQPYVQASDNRIIAHVTKQTSTSAGLTGSLQYAHLYKISSNITIVQAQTFSKRDMATCAFEYLSTSFKCPSEHFLSLNTGIDRFGYLYQRIQPISCFKGPVPGDCVNGVYNQGHCVCDPYYEGELCETPICQKGTLDSLRENCKCFAGYAGVVCDIPVCNRGTSQNMPSINNRTFVLLLDGSKTGRMKNIASSLEDLLTSVFDESNEPEYRGWFTHYTGVVFRDSSVGEQSVSEVIESDDSTDFVKKMLAERDKSYTPSGESRSVMAAIVRAMQSTYVQPRSYVFVLTGADAEDYERKTAAIEALARTHCYVEFLIVGDEEAPGGGKNYTKPQLVAYYDIVRETSGQLYQVQDNEGNVDVFKTFWRSLFSTYYDTFFVTTVQKQDCSSGIDEYVQLGYNASTLIIDAVGSGQVSIFDPQNRQVNNINSIKGSRTNRVNTVSVNMTGLWKITLPEYKGFCRLQVKGKSNIEIFIGYVHDGTDGGLHSRDVDYRPLTDGVNAVVAKSDVAKINFVQIFSLDERKLLWSSPMIARRSCTYQYVSSRTFTCTEPSLSVAVEGEDDRGYPIRRMIHTHCVGNFATTAAPGTTATLLTTTPVVTLKPLQCDDNAKFDIILMFDGSDGFNANDFEDVVKAFESVPEKMNISQNGIRFAIGTYAESAEIFSTFENTDVTEFHNTLQRLVSFGHEGVTANNISDIFTKIENLNFRDSVPRVLVMFSSLRLDVGLGEAYPDPKQQAEEFRAKGKILPIGYGKYVDADLLKNLGNNCAKFAGDMLEMKALLPWVTSSLCRFVIGYLKKLPLLWLTG*

>Eve|EVEC_0000407001-mRNA-1

MDEQSRHYNRRIGQQRNFGKMSEKNPLKSNECTGYDTAADFCEDHGGKLPSILSKEENNILSCISTYGFWSGHEKVNGSWRWLDGNKSNLTFWKSGHPGINGRHIKVNKDGEWVSVSDGECAEAICIIRAEDEGERRCADPKGPSNERRATALLKSSKHTNYETKATASLNAAVHPQPFLSVEHSKRCLPSFSGETDATRSIAVAERYGRWHYCLFDVKVKGRKAASLEESRKYCYHTFHGRFLSIGSVREFIFLHGVLYKELEDRRPNVVIAPVERNFLRHLADANLKKTSVEKKGNWWSADKYCRTWHRGLLGTFYKVSDSARVLRTDNKEYYYLGASFNYSANDYYFDDGRHLLTSGLDSANNQSCLAQLKDSVVFVDCQILFTNEKSIERFACKKYFDTEEDYGGQIEELLSFEDSERHCETIESTLASFSSEQEFKMIQEKYASKKYWIGLKQINENWEWMDTTAVDYTVWKKYNLTDEEFSVQKCMFVDGTGSWGRENCDFHTEVSWIVAQKMCAEYGSNLAWITSNDEQAFLEEFTDLKPFWLSLVFNTDKWILPIAESPDFVNWKPGELDKCCSDEQISAAYNAPEGWRHAPPFFKAAAVCKYESEF*

>Eve|EVEC_0000410501-mRNA-1

MCKRTNFASLLTVDSQAEQSFAISCFIISSYVTRYETYNYQIQSLLGFTYHKYWIALRRSGSGWVWENGRPLTYSNFGGGGSGDCVMSINRRWTLMNCRYISPTTQPATRPPPLRLFLATPLTCQDRVGPNGRTDCSERRSFCNDRIYYHYISYQCPRTCNRCWLANTGAPSVHYC*

>Eve|EVEC_0000437301-mRNA-1

MLSDDYEDQNIPIIQQLPGLLTPGSMGSGSNAFSGSSLLEPGSSRWISHHMKRVFAPDVFDVHFEGQQNAQTHLSDSLSGQPIPETPPERPLEKLPHTPAEQLSYANLKSTQAKRFELQKRATITSWNKTKTSTPIISQQLAAFAEEVTYNEALLRCLKLSSNLVTIRSQQENDYVTGISKVKNVWTGLFRRKTGWFWSDYTVPNFFNWARGQPRGGSGPNVRCVGINALWTSGTWFDADCNSHFGYICKYDPQSKSSFLSHNLAITTKMHLHRPVITAVAPNLFAQKPLIYLRPPAATANLYAYYYYALMQQLAHQMYRNIAI*

>Eve|EVEC_0000461501-mRNA-1

MLTTQKLLSNDVEEGAELIFLLLLEKLACFEISSNITRKRKGRSCFLTFSFSEKVTWDEAQRHCEARRSYLATFDSALQYRDISKELARNPFWFGFRKNAKGWEWVDLQTPVYSEMPTESNDVLAEFHCAYGTQTGAWNTALCSERMEYICEFRR*

>Eve|EVEC_0000461601-mRNA-1

MKIRSYEGFIIYILLLLCHVFAEEPENECPEWWHRTENYCYSRIEDRYTYDDAEEHCKKKEARLPTFLNKKEFDALLRYLRAPRNYWFGAKLQDKLLWNWTDGTPLQLSEISLPIFGLGNFSCAVLSQKGVQTVECTTYQNAICKRPKRKLFNQLNNLEYPKLNCFPGWDELEQNCYLLSTQGFVHGAAVKFCENNEANLATVFSKEEEEFLKTLLGNKSAYIGLDHISTRKNAWIPNVPDRLDRIYINLTKKRNPNGKCLQLYYATKQMDSQICDEIAELTCQQESSVIY*

>Eve|EVEC_0000469601-mRNA-1

MLRSLASKGYDAKANIKVPYDYASSICSALNAHLVSIHSEIENSFINKVHNIRSIWLGLKLTKNFDWKWEDGSVYNYSSWDILQPNGCCGANVRCAKMNWIIKNGKWHDLSCQDESGVVCKYDPKNRNVNGQNMAEDETQGLPKRRIKGPLSQEATTTEAGVKAENEKQSKNNSQQKNDNRFSSKTKWPSNIIADYSFRRSVKQPKTVTTTSEETQTPNFEEAISDDISATNSAKKTSVNAKTLLATVLEIPANDTAPSKIEMKPKNESLEKSTISSASERNYGFDGTITEPSDVKESQSITRGYGFNSTTFDITKQISTNFAIDKESSSTAVPEENMLLTRPKDELTCSSEAPSTYNSIKKLATSHEDTSAKAIAVETSERRNGTMVDQQFVTQTDFKTSTTAITTAAEKSFSVSTIRNYVDKRIFLEKNSFTTPSIPLAESATTDFNTPSNSENNAFRLLSNTSTPKKNVDKRKRCVPKTVGDGNIVCVCNATYCDEFPSVGKLHSHQAVILQTTKSGKRFEKSSGNFCYKRRKDRASKEIIITVDARKKYQEFIGFGGSFTDAVGINLNALTQPTVDNLLKSYFTSDKGLHYTHLSIPIASNSFSTTPYSYDDEDGDFELKHFNLTEEDYELKIPYIKEAKKLAQNKLKLFATPWSAPGWMKVSENMLINSPLKNDTKYYQTWAKYLSRFLEEYSKNGLNVWALAVQNRPYSNATKSWQQMRYSTQTKRDFIINYLKPMLQKSKSGKNVELLIFNDIYQFLPSAVDQVLKDSQTSNFTSGIAVQCDQNSFNEDSYTVFSEVHKKHPEKFILLTETAAGRLNPTHDDWSNAEIYANSMIGSLLNYVSGWTDFNLCLDQEGGPNLLDLSVSAPILVNTAADEFYKQPLYYFLAHFRYRLIFELSKIPSSPSVNFYTYIAIKITA*

>Eve|EVEC_0000475201-mRNA-1

MILTFESKAEYENFRAIKTDFKKKIWLGNVKHSSFLEWDYDTPRFINDESRSYECYSFNATTINPTDCKEQLPFVCKKSTVKQDEKIWVGRRKIKQLVHQSPDPQELRKPITPSNLKSCPAVVGEELLRRKCSKTYDVYFEEYMGKDKCPPYTMKYGEYCYFAIFSLAIFSKMPKRKPKNLESLVRLYQAKVKKFSRVEKFWVSELQTDEWERVKIFYYGSLVRYETAVQICANEGGFLASFVDEKEERVLEKLAVTRYFWLGLSQNDNTWEWHDGLNGDYIEKQIENNRFHTMRGNEENTRVFADRETMAWYETGSDQDEFSVLCKFHEVDQNKCPTYFSLQDYFLQPPESVSVNDGERHYCLYDFKLSKEMFGETEVINETVTFETAENFCIDKMNSEFLRPESADEFVFLHQSRICYPSWEKGDSTCYRIYSTYKNKTSAEKICESMANGYLAFKELTRDQTSVKEYIRDDCYYVGIYKDPYQKLKWIFPDGTEVPQSVMSSQRANYDCACIKATDTIKYFHSKCDGMRPFICEYKSRFYAQNYHLTLIILHNSFHRLGDRTH*

>Eve|EVEC_0000552301-mRNA-1

MFRIVLLAAIQLAFVTAKCPTGWTNLPGSLKCYKGFAERKTFAEADTYCRENFATNAGKIANELDKSCDFWIGLHYNEREGVQMWTDGSYLGFQMWDSGYPTGDIQKRVTYLPQDSRSHKWRSTTVADDRKCFFCQQRPAENDVRISENAAQAFCSAWKANLVSVHSAEENAFLNKLSQGKQYWLGLKYSTKWNWIDGTAMDYMNWNGQVTSGSKDTVCADANEPDFYGKWSKQGCYKRLRSICKQTRMLVAIIL*

>Eve|EVEC_0000564001-mRNA-1

LLIERFLVKLECQTGYERFREACYHIFNYTLNFIEAEQFCVNDGGHLASVHDNEEAAFLVQMIKEKSALMIGYNGRNWTDGSSTSYIANQSLLLNYLIPSPVFEDRSVAQQLYLVADSNNFFFSYSVVEHPSVPFACERPAYYNTDRSCPQNNIFAGFSTVTSPGYPKKYHAGYNLNCTYYFAAESGHLGHIQFKDVDLNGDYVKVYDGWNPLERRAIGIVTGNNKNTATSFKTTMTSLISIEFIGNYTGKWLANFTSEKALSGGTKNGISSESDERAETSDEQSDSKQYEEETAFMSAEDNDDQVLTSPATLGSAENILSQEDVASEEQIPDYDYEKS*

>Eve|EVEC_0000712301-mRNA-1

MNSTLVTYLSEGENLVARAVGNSGDFWIGLKKTDGEWQWDDGYPLIYQNWLNDNVTNLKRKKLDCVQDKFYKIWVDRPCSEKASFICQYEAQFTCPEGWREYNGDCYIFERTAIALPEAEKYCALNGAEVTDVVSKEERLFLKEVSEQNPHWVQFRTQGGNQKNAKRLDNRRSENDSLTENNREIKHVIATSEGWKIPHKYTLTAAVICKYKSGHLPSGETQL*

>Eve|EVEC_0000769101-mRNA-1

MNKKCFQSHLAKIENDADRNFLSQFFQVEVSNLTAAFRNIGIWIGGHSVLNYTEATYVWKPENYLITKFYWKNDEPNIYIDYTNVCVYIDDRSLYLDWATTLCTDEKYYICQRDVTDTEKKEMFAQCYCPPEYSGTFCEKAVETGNAKELQQVICSNEKLNFSCSSGEIIQVDFATFGNIGEVPSHCKSLTSEMLLVPQSDFCANPESLQRITALCEGLRECTIPSIPSHFPKSPCFGTPTSLTYRFQCVEESQSKCPGDGIYYNGRCYRAYFATAENERLTWEQAQQSCLKKSGILASITDETLHELVVNSARTYSDSADYWVAVWTNDDSDIIVGNNKTLGFVPGGSTIVGKNKCVAYKITETEAIWMSEECNSTKNWICESPPERETRAVEEYKEREREGTVFIGTPP*

>Eve|EVEC_0000787601-mRNA-1

MDFDTAVDFCEDHNAELPSISNDKQNDILASTSIHGFWIGYEKVSGEWKWLDGSTSNFSLLSKDKSGGKHVMVNKKGIWTLANEDSCAESICIIKDLEHSEMCPVAIPTGRRDNIKVERYVVSERNGKWQYCLFDVNVARMSWEDSKEYCYHKFQGRILSITSAREFIFLHSILYSQKLDGIHNVVVAPVERDFLRHLADANLTRKSVEYGELKFRIMHPKEGHMKGTYATHHGHSSKEYATSWLDLKGFLYIYAEALADVTAIGSRDN*

>Eve|EVEC_0000800001-mRNA-1

MEQSDVFEITFTVYSHQGPLENSKADAVCGTYAAFLTSVTSDDERAFFLERSVSKKFWVGLYWIPHNVWESVDGNRVVYISKQKDLIATEGGFRCLLRTNLQNETFSSKKFLQFTRTNAQNHGFGLASTVTQLLVFGHKSLEGRMLDVATFEEAVEICAEEGAFLPSIPGKYEEDVIFAMSPTRYLWLGLLSDTAAQMMWMDGSNTSYALSKLQSSRSSKVRSEFSKIVGDRNKKNWIDVEGTAHKVSVICQYDSIDINKCPQHPNLEPYYMQPPKSISVDGGKHYYCIYDFQVHKTLFSGLPVRNGEASFIAVEKFCLDKVHGTQIQPRNEDEFIFFHELFEKNVSLIALSQRTDHLWRYGETELRTNNNRKFLAFFDSRKPKSLRNTWYWMGFQKYEDAWYADAGNELNVSFQTVRTIDKKGAVCAVYNRLQNSVLGVECEEEHQFFCEYTSMVKWSRKRLKRFVCKQPYIPENEMITSEKFKELLFFAEQSVDAPSKNLNSTLPTALEHPFQFSHSVPWDESADFCQVEHGTLAVFSSYKTYLLVLGNKINIS*

>Eve|EVEC_0000800101-mRNA-1

MVQWGDNKIKRVLCKHYYDSKKYLLSSKDVEDFLDEREKLILHYREYLCPYEWISIHDLCYKYVSFSIPKPWNDAAEQCQMEHGTLAVLRDYETYRHILQEIKKSYFWVGKRRWKLEWEWYDFAPAGPIKILGEIDENDDCAHARNEDGLGATECFNELPFLCQFDPKKYCRAGFTEYEGFCYMFQRTKQPLRIAEEVCDARGSRIVWIDSEEELQFLENLYWGGKRVGGDKTILFRKERLQRVFMEENAINKIPQISEAVFKLDNYPDLGKFKPNVVTLREEVRGLSSKRTFWIALRYIHGSWSFPDQEKPKLLKWATNEPDGCCASQVMSHAAHEPGKGWSDRNEQQVENFICKYKKGFRDLPSSVFEGSPGGFQYEGNRIKLRRASPRSSPGVLLGGLDSRDNVVKMTFIMGMLTSTLVICSVGSVILYYSTRRAKLDSNILALYDMVTKSDAARYNMALYDQTYCEGYARHLREVNIPKQRSDLEVTEWVGKVQGAQFQGKQEARGKEEAMEKTEEKREGPKGKSKSQGVQELPAIDPVATEEVCHEAYIKQAVPAEESFDAGSVWRKCPL*

>Eve|EVEC_0000819401-mRNA-1

MCLYKVYSNLEELGSCFNGDGRLTVFNTSIGVYFCYYLRKLHIAEFEVAENLCEENHAHIVSVKDAVEEEFLKATFLQEVTPNGSEFWLLLGLQLNNSIPRWTDGSGISFIANRLQLYSHQYQSNCFALVRILTNSGFSDNYVSISCDEIPIKEVLCKADIRTLVLSRKSRTLQGKLPLAALKKIPDYEFLENYFSTGPLMEAVTESSFMGGDHKSDTNDEETEPKCDILRVIEDLNYLVYSERETIAASGGSAKSQSESLPLLPGCAEHLLKLVEAAYEEEFRKVSSCVLFSAAVKFGEIRFFIGNWLDDFNGLTFIPFLGLIVTILVVIIMAIQMCTRDRKTPLSEDSYCVPSIQSSPSEAVDTPSNKFQVRCDQTRYALVATRDSE*

>Eve|EVEC_0000835001-mRNA-1

MKFQFSAEVERGGLNYGLDIQSNSAVKATTVFTNVAEVEMTDTCDEGWKAFGDSCYLVVSESLEECLSYSSTLVSILSKEEEEFLERLVENEKFWLGAQKSENEWIWLDSGKTVDSKYWSESFSSSDSVLNEGGLCLGYPEPDVTMCNRTSDMIKSFLHAFMISLNLNGSDVGTRPESKVKCLAKKQSQLTTVLSDAEGKFLTDLRCSLFNFIVGNANQVVIGCLEITGGKEVWTGLTYSAGQWAWDSGQEQLNYTNWAEKTESEAIEISGVAFMYLANDKWQVTTMESEIRGYVCKRLGSRVRRALVPKETKVNK*

>Eve|EVEC_0000839701-mRNA-1

MRDICSCQLQPSEAAPDDFATKKVESFEEAESACVMQGSMSYESHLLSISNKNEHAILTELITGSILGKALLGLHKKVEGAVEFTDGSDVNYVRKNLQIFKDDCFYIASKTGNLSNSACESGVQDVFCQARPRGNYEEESPKTYGCTSNKCQRLWESIEEKLSVLDIVRSRRQAEKSKEKLKESINLASFQNAVIQISLLENVLGGHCSCTGNEESDDFRGGEALLGIIVGIFGVGILMILRVAKTTVRKLRPTYDIVSTRAYYSKTGSGEEYYSRVSRQFDDAPIYTM*

>Eve|EVEC_0000856001-mRNA-1

MFAQYFYPILLAILWLTSPNYGYRGHVCTLSNLELDVVFVLDASAGVDQHGYAGEKAAIYGLFNELTIGQRTEMTRVVTLNMGSQTNNEKVSLGMYRAGDQAKKDLLELPYKGGEVNLQSGLAAAYGLFKTESDRPSKPNVVVLFSSTAVNCANGGLTQSGACKQAFRMKNESIILISVDLRYHEDPNEETMNLGEYSLQNNETLFDKLKEIFCEVSPTRPPTLTPNACPCVLHGVWLDIVVMFDSSDGASTVGFAGQRGAITGLLHRLFIKQKGEQFSRIGFVNVGSTATLVSDLNKYSHGTEAIMDMMAMKPMKGEYNFGNGIVQAKTIFQKKMSRRERQNVKDLLIVFTNTIVKCETGKEGCQVAKWLTENGVTILTIDISVYGVPNGDPNQGSSFGDRCFRLSNNKYFLENLEDLICKANCFCKPPTIQFKTDECGIYGQCVYLEESPAASESAKVGCGLVGMTLVDVYSDEKDKFLTDIAKAEKTDKYIVGLQYINDELIWSSGETMDKDDYNIYAPGEPDARRGECTYADLYSSKNSIWKTMKCSYTSPAMRYFCQKDTCDSEHYCQ*

>Eve|EVEC_0000885401-mRNA-1

MILLQLSYGDNSDWSCDHDFTQIGSWCYKLVDTSATFDTAVNLCKTQYGANLASIHDDKENVGLRHLGDSFLIGLRYYEVASDLKWADESEIYSPGWPREYHLSNKTCKYNLAAKPNSTIDATIQLFSFGTTDECRDTLQVYEGWNLISIPDAQLVYLTLLFNFKL*

>Eve|EVEC_0000915701-mRNA-1

MTNLSENFKTPNVSETVKKPISLETVKKISKVDVIPKSSKSPKVDTVLKKIRKNEVRLTPEKETLDYSTSTIATDTVRIFFIDLSKCGSDKKFGKKKLMANATFTLVLKVEYCDNCVWGDTNNCKLASIHNEQEDICIGRLVRDTKTNAWLGLRRPKFTWENRAAVNFENWSDGKPNFLGGHCTMIRYDLLWDNYFCTMKNPAICQWKRTPTNGCGNYSNIYSGDHWCYHVTKSRMSWLRASSYCKRRFPNGRLASISTKDEMKHFRKMKQNFSTMFLWIGYNRLKLYWSDHSPVDYQDFNNRQRYVGNFDYCAFMPPSTLGLYGRTLWSQKPCNHTLQARVCQRTKL*

>Eve|EVEC_0000962501-mRNA-1

MIRKKLFGIDTQNQCPVCPNIRRFYLQPPKSISVDNGKQHYCIYDVQVNRKYFDLDNSLKRLASFESGEKFCITKMHGSVVFPENEEEYWFLRESFSANISRIGFSLRTPRAWFYGELPLRGSKETDTDLIPKFKVFDSQGKREEINFYYDYMYEEALMFCESLLDTHLSTTLHQLNSTQQKEVLYPYIGDYWLGLQKSEGHWRWSDGVIANLGDNIFESLRNVNNTQEHTFYNSRNGHFYTSEKIKRYPFVCEYKSKIHWFKNKLGRFLCKRPYDPSKKLIKSDDVDQLLSISQDALRYRSKSFAQYQLDTDC*

>Eve|EVEC_0000971501-mRNA-1

MQYDVAADFCEDHGGKLPSIISEKENTLLSRSSHYGFWNGFERSYSSSKWGWIDGINGNVSFWKNGQPSEIVGHVKVDQRGRWEVVSDETCAEAVCMIRDIENSIKCPQYFREGQMEVRRTTVVNVRDGKWHYCLFDIQTKYNKRTTLDESRKYCYRTFHGQFLSTSSVREFIFLHSELYKVSDEKRPNVVIAPVERSFLRHLADANLTRTSAEYGHLKFRLPHPEEDQKYLCRPGWTFVGGICYQSISRTGDWYALDGYCRSWNYGLLATFYTKDDLEQLKTKWKYPASSDELYIGCKREGAKTCVFEDKRLAPFLNCKDTKNYCTFGSNGVYLSDGRKSIRGICEYKSQIFFTDEKSIQRFACKKYFDKKIIFENQGQSTSGNKTYYGQINQRLNFENAQRHCEAEDSMLASFSTVEEFKTIKRKTSSLLGNAKKYKSASYWIGLKQVNEKWQWMDTTTVDYIAWNSFNVTAETFGVLKCMFVDEAGSWNRENCDVEHDFLCKYEYFTESKPFWLSLVLYSNDWLWPFRDRPTFGNWKSGEPDQCCPSSKIYAAYSEKGEWSDTSPSFKATTVCKYRKGQLYKDVEKPYAAAYNEVEEEMREAEKLRTEDDYMGEDGKLRQRKQTEQEAMKHTYMLYTAVAFLMFATIFSILIAIIRTRLKTVFDVKPPLYDEFYEPFVATPLTQESTQYEDVKDRSQDYQLKLLSMSH*

>Eve|EVEC_0000973001-mRNA-1

MHPKCNMFLLLLIGLYICLLKATAILAQQCQTVPVSLTAKDSLQSFTTPDYFVGSYPNNADCFWLLTARSVDRRIYLRVDDSQLDDALFYTCDDFCAIYSGKTNTSTKLKQWCGDEKPYAIISPSDSLFVHFHSDARFQRHGVKMSFTDYEIPGCPPGWVSNTALGTCYTLIYSGGGLTWAEAQNECLNQRSNLLTLSSQEEYSFIIDTFTEDEAMFWLGYTDAAVEGTFKSIDPNEKIWPDQFPTFFKNHDFEDCVCINAHSYEGIAYEVTNCMSRFPFVCKMSRDGQTIPYAPDLNAIRKGLAEEHTVRFNWALWILIILLLIAAILAILYFCYKCQQKRSKQRVGSADDNNRLTHTSTQLSAATGNEHTNVRYVQQISHRSSQPIENYPMNEGLSPTDRRRAAEHVNRFVETFERDLDRNFDTPGTANAAQNPQRLDAPLPRRPLEQMCPTVLTVVPSRDRFPHDFVNENIEPVNGSIRNEAFLKNRRDDLFERPHVTLLDNVSAISLDDFWNKSAPSGSR*

>Eve|EVEC_0001035701-mRNA-1

MLQLLLLSIFLPIFSGQQLSLCSSATWTTYNGNEYCAFTGYATYAAATVLCKKFNSNVVSLLSQAEQNFIDSRYTSPKKAYWIGLEKSGSTWEWADRNAYSYNNFRTTQATPDEQCVMVGANNPVDSFWDIKNCNFHRMDQIVVCKKSGSAPKGCTSDSQCSVYELCQNGQCLTHGNKQCQADTDCSTGETCRYGYCGFPAGKACTADGECGITELCQNGQCLPYGSTPGSSPQQQKCADDGECDPTTLCQSGTCLPPTAECPNGNECQATEECRFGFCALREQFLTVAAAGGGGGGGGAGGNPGP*

>Eve|EVEC_0001043201-mRNA-1

MSWKLNVVVYLLSLIGSTLSNLCDINNCYEIFPEDNIPGATVNEAMEFCAAGVGGFLASIHNAEVNSMIAKHLSENEENGSALIGIRYYPSEKKFFWLGDNSMVKYTNFDENFVTSAPTKGHCAVITAAGLWINIHCNNTRNYPALCAIPKRKRSEKPCTYLLAVGQEYRIEIQVIDAAFSENSYINLYRGWDPSEDYLVERQ*

>Eve|EVEC_0001054601-mRNA-1

MNSQKCYALLIEKPKTFEESERYCETIGGTLVSFSSENEFKILKGRHSKKKFWIGLKQVNEDWEWMDTTAIVYSQWKERNVTRDVFKDQKCMFVSGTGDWHRENCEKKLYFLCKFNRMRLT*

>Eve|EVEC_0001077901-mRNA-1

MPLSCRSVNYEEAEAACATLGAKITTARTEAENTRLNKIANGKKFWLGLKRIKSSRWKWVDEGPLIFNSWSGGKPSTTHDCALAANYGLWHTYPCQKKIENSIGQDKKNRREAFLSCSGKSATLLQLEDLEEENFIKDFLVGQSFSNKDKKDKDGPVAVWLGAEKKDNNWVWDDNQDFKYTHWEMGQPDGCCGSNVIYATLNVTKQGSFWDDTDVEVQHNFICKYTIQEPDEPTPFITDQTIVISVTKPSTTAFATVTIDEKAQKGQVGLGTLIMIIAGILLAITIAMTACFSRREIIVAPQPFYYEIPLEADEGPMLVNEPLHSAELFTQSTQPNIQIAPEDRLYDQYAKRPV*

>Eve|EVEC_0001112401-mRNA-1

MRSIWIALRRASVDNHTIWKWSDGSPFVYENWQLFQPNDCCGDDVTCAKMNWLLLSGAWFDTSCLEKLGVVCKYDPSMRTVPITTTEANSVLRENPNPLDGESIKFTCDAVTQNLAVLR*

>Eve|EVEC_0001112501-mRNA-1

MSSSVGRQTLAGAQPIWLGLKAARGGWLWADGTEVKYVNWYTTQPDKCCGEDVSCATTNWIFDNGKWFDTSCQALSGVVCKYNPKSRRTPLPAEMIEYKTTIRADSGLAEYNPIWIGLNRIKNRWFWNDLTDMKHKNWQQFQPDMCCGSHVNCAELRWIHNKAKWFDTCYFDVNSPASTPVPIWCRCPRNWVVYNEHCYQIYKSSAGPLTTPIAAAKCRRLGAELAEACTDTEFSLFEKMVEPFETQNPLFGMMVDKLFGGCSVSPAHGKTVCCHINWLYGTPAFGALYSTTCPTSNQDVYLCHTMSTSIEAPVFCSKTASWLGNKFYQVDTRLDSSHHIAPQK*

>Eve|EVEC_0001114301-mRNA-1

MYFSPLTKTCRNTLRAQHENEELEPPQVDLCDPGWSRLENKCYKVHLCGGCTWTETNECPLASIHSDKENMFVTDLVRRSGVDSWIGAIRLTFGIDDITLTSYFNWDRGEPSSKHNLAAYCVAMKTNGKWASYPCHTRMSIICKFRTKNVETAIRPFTKYWIGINRNSVRWSDETPVDYQAFSKYQDLPGDVRFCTYIKADLNRYAPPIWMQKPCKETLRAKICSRPAQQRVDLPELDSHFPRAKHPSRDKYYNIGETSLSLSKVSEIDEFKHSEERY*

>Eve|EVEC_0001176101-mRNA-1

MLHSWVLPLPQMTVAFKGRNINISLVFVSECPLGWHQYESTRSCFQVNKKTLSWSEAERECNQEGGHLASIVDEFENVQVFDIVKQANLSVLTVWLGRLVKLSETGEYEWNDGSVGRHSEGFKGGEPLSAKFYFDSPPSGTNKCLTMWLDSGRAEGSWLEWSCTYRPGYASLCKKPYKSVPSSAPSIHQSSRCCVTARCTTKCPPRQRCIPDDLNCWTKYCPDNGPGWCLPNP*

>Eve|EVEC_0001176701-mRNA-1

MLEDDIKQAKSFFFEVFEVGLKYEEVIDKCLLHNSTPVSILSSEEDDFVRGIAKSKDYWLGAYRTEHDWAWLDPPTAVAYTNWYGIGEPKSFDKCISAPIDCEPEWQKLSPYCYFYGNDVTSRDGAKAKCMAKDGSYLTSLLTEKEEQFVKNMTGAKPFWIGLMYSNGRWMWDDDIQRLKYANWANILDVDAAGKSSTAYVYANPKWRVTTLGKENYGYVCKKLGFRPRPPEEASRAQRSRSGFSQWMIDVDKESSGMVLITTIVIVAVLFSGVTLVCLLTTLFKAEKMVPIYAEVRDGNVYYNRPYTERPHQYYVSVPPEYEEVIVEKTADLKTLPDDIPPLTRTPYPAPL*

>Eve|EVEC_0000017301-mRNA-1

MTSRLQVCFAKSLRCYVFCKALCSHHPHDAAALLLDSFGCRSVHFKNIGRLLERNLQCHNRNKAVFQVVFAIPVNVTVVTASAIVMYYEKLQNYSECRSTCKSIGAVPLVPFDEPSLSAFGTFGYEEEIFWVGVSAERCHWQWDNGPFLCNYDGNFSKHINQCASFRENKITETPCNSTLRFVCKKSRIYATLSLLDFSLDLLKFIKAIESAVLLTSVFRNKAGNITGLLDLVCPEGWKYFNGDCYIFYPDKTNYYNARMKCDNGKGYLTTAVTKMEREYLENISEGNEIWLGMHESKKVLWKGEDGNLLVYHLDGARFKDAQQCLIVQQKQYFNYNCSEEAKCICSRLG*

>Eve|EVEC_0001190401-mRNA-1

MTWDDAQRHCETQRSHLATFDTEQEYLDVSNALEGDSFWFGLRKIAGLWKWVDLQTVSYGKLPSESEGALRNSDCVHGKRFERWSATVCSEKMGYICEFLR*

>Eve|EVEC_0000019701-mRNA-1

MQCSILTVVLLYFFLMTIHSSNQYSISPLQKKPNHFPSQLHPDFENSLQKLLTKSITEVYETTPNCCNTTATPTATSTTTATTPGEHSLVKHLNCGCEDESLTRGVNYWLDIVFVVDSTDAVRDLDFSDVKSFVSLFGNLQIGTASNGDPRYSRIAVVNTGAEAEIIADLNRFKSGTDLQNTVAQKLVKKGGTKFNLYAGLTAAQDILTSEDNASRRINVPKVVIVFSTIPTNCGLLARNSSTTGDRKVDRLCSLATYLQQQATLITIGLKMAGATSYPHIDIANNCNRFQNSFDMTMNVLRALCRANCHCPKSYDQFATGDQCDSSGECVTVHQLSVPYASAVEICLQDGAVVADAATHRKELFLQSLYKEAYFSPFWIGLNFIDGRYIWSNNQSISEYNYNNWCTADHQPKISGGNCVYEDRCGEYSTGWFTAECGMMLPTFYFACQKDACESSGFKN*

>Eve|EVEC_0000019801-mRNA-1

NPTTRDQWWKSTTKSPWWNPTTTGQTGQWWKSTTRDQWWNPTKTGPWQNPTTAYPTKSTTTAPYPGIKSSTCGCEDESLIRGVNYWLDVVFVVDSTDVVRDEDFYDIKTFISLFGNLQIGTASYGDPRYSRIAIVNAGAEAETIADLDRFESGTDLKNAVAQELNKKGGTKFNIQAGKVNLTRSEKKVDGMCSLAAYLQETTTLITIGLKMAGTKNFPQIDIADNCNRFENSVDMMMNVLRAFCRANCHCPTSYKQFATEDRCEPSGECVAVHQLSLPYRSATEICHQDGAVVADAATYKKERFLQSLHATATFSPFWIGLNYINGKYVWSNNETLNYYDYNNWCTADYQPKISSGKNCVYEDRCGEYATGWFTAECGMMLPTYYFACQKNACSVNSYCE*

>Eve|EVEC_0001202201-mRNA-1

MILITDGEDSTNVGVEHNLAWENDIDVYALGVTNDPPEPVPVSTTCGCDKIGQMIYNNYWLDMIFVVDTSDGVARQDFASTKVLAKLEERTTKEMQSIISNFKQTGGQGVDVQKGLELAQDMITKEQEAKRRLNVRKVVVLITTESIDCRRQQTKFLKNKATDTETHVICQLVSIMETNCYCLDDYTQLTKNTDNCAKYAECYRVNSFSLPQNMAQTTCGEVGGTLPNIVGSEKEEFINELHKTAGFFPFWIDGTCTSADTCRYSDNSLVKYKNWCTAKKQPNFGNGNCVYEDQCVGFNVGWFTGSCDDFMTDRFFTCQVPACSVNHYCDYNTMGKKINKKPKLHLKRH*

>Eve|EVEC_0000020501-mRNA-1

MSRSCLTGWTMIDEMCLLQSTRCLDYDRAADFCEDNGGKLPSVLSKRENDIMFHFSKHGFWTGYEKINGSWQWLDGNKSNFTFWGVGHPRNNRAHVMVNETGEWISVDNGKCAYAVCTNRGINPFNASTEARKGDLMHHNSSKNLLSAHSRLEF*

>Eve|EVEC_0000033201-mRNA-1

MKAVILLIVYAVTLVRGSSCPPGWEPYAGTSDRCIAAFRNESSWFDSLRNCQRYGASLVSIHNSFQNLHVATLAEKTFESCKYWIGLYNIDADKKFRWTDNSPTNYSNWLQGQPPQVSEATTVYMSLQSRFKWATTTEVAENSCFVCEITLNSSTVGTFAPPTTKRTTVAPCSQQENRDVCGESDHLGKCEQIGNSCDYHCCCTDGTYSWHCE*

>Eve|EVEC_0001272501-mRNA-1

MYICHITWARTYPCSTFGRNWAAEIRCLRNLLSHCEKSLKDSSRRTSRDLEEVPEGPEKMIDSNSHSYTTQRCVITDTPSFDCNHCCTKRLLELEIQFNKKINELNKKLDEAALRHNRKFAKEILSLNKTMRLMTSRIYSNGGNEFIVVDEHVSWYHAEENCIKWGGHLASISNEEENEFIKGLIRGQSWIGVNDVAKENKFVWTDGSRYAYSKWRKAKNYAVFLSQAVIKLKRSFGTEVSTAPKILQHLCRFNMRTVQNQPEQTEALSPTGT*

>Eve|EVEC_0000036601-mRNA-1

MPLPETSEFEEMLNSYNFTVVPGNITNGKNEPSSGEPFFNYKTAVELCRTYGADAVSLNSADEEDFVKKLAFYTSISKLNPNELETPRRVLLGLHGSSTKNLVWSDSSSTDYIVQRLAEEKITLSEDRSCLELQRRYLAQQIYYGITVL*

>Eve|EVEC_0001284501-mRNA-1

MELLTIHNDLCYTASRLETLHWKAAQGRCLSLGGTLPIRVTHDTNTVLRYALSHSNLKSSFYWFGLMKTGYQWRWADGDVIDPDQQDWAETPNDDNGEAMAAVFSRPESWHWISMSQSVYNAWICQTKPKFCTSPGVSEFGRVTFSSQSYAVGTTCYYSCEEGFELSGESRRECLSSGRWSHTIPICKRVDCGPLEDWSNGTVLLLNGTTTFGSLAEYRCKKGMVISESSPSYRVCEFDSRWSSVIPFCTDIDCGKPPELSNGKVDFEGTTQNSTATYSCNNNFKLIGHKKIYCSENGQWQPSSPVCYDLETLREMKESSGESNVILAIVVVLLLLLLAFLSFRLTRRGFSPLAIHTFNEGKRTSPRPLVYSTPSQQQDSVIYYASTGASMTHVEVPPELLQLHQLSNGNIHITLPSLKPIVRPALSLSMTQPIEVAENNSRNVPETSTSPTPSQLLYSFDDDPVYDSPSEDNVYEELSQAHDVK*

>Eve|EVEC_0000038301-mRNA-1

KSAKLYFNQTGRSRRQDDLLSAIQFKKNFWQQKLLFKEFWIGLEKQSNERWSWTDNSPFDFKQWGFMQPDNCCGTNVKWHWFDVSCGLTHGFLCKYNPLCRRYCAYQYRQVL*

>Eve|EVEC_0000069701-mRNA-1

MDGKAIVYRTLVIGIILQNLFVVAEYDCHKLTSQWNNHCYFKVNGAYVTYPKAQFLCKAVNASIVSIESAEENNFVRNLSDPYSTYWIDIKPNINANKNAQQTADNSGYSNWDISKTMDAGNGNCTIFNSVTGFWESVNCTNYASVICEKDFVPIIDEQKPSEIYLSSTASTPSPPSPSAALISQSSEILTKKLSPAMDRYCSQRKRPKTVLQLSTNRRFILGPLIPTVQLFGNKFFAF*

>Eve|EVEC_0001369501-mRNA-1

MRWERGMEEEGKSVYPSTKLLSQYGSQLARIESQRENSFVSRLINRPLRSASLVQKTQFWIGMIARRTEDEDALFLWSDGSIVSRYIGFWEIGQPDYKSGTCTRVDAHSLYFIYLFIFLITYIRLI*

>Eve|EVEC_0000092501-mRNA-1

LLPSFSVCSADRCDEGWRYSPHSGKCYKFYPKDSSWALAEFKCFFNGGHHVSLHSVYDNQFARELAKGAKTIWLGNAQFGKSVDYVWTDHSPYNYESWPHGIRPPRISGRQCTKMNIVTGEWFQSCCKVPSPYICQKDARLGSQYDSHHQKLHNIVDNSNVPLS*

>Eve|EVEC_0000096601-mRNA-1

KQQAANCSYPHISVPEIPACYFEVKETGNYSEVSNRCQEHGMHVASVLSKKENDFLLTFLMGKYGNSFEHFLGLTEQGTWEDGSPVSFINGSSRCNVALTRFGWKCRMYTEHSAICKRIKVD*

>Eve|EVEC_0000098101-mRNA-1

MCNLYNGTVVSILSAAEQNFIDSKAVAGKYANPRKAYWLGLTRNGSTWQWTDGSPFSYSNFRAMQTFPNEQCVMVGASEPTDSFWDIRHCNFSKVQQLVVCKRRNPGN*

>Eve|EVEC_0000099201-mRNA-1

KQQATNCSHPHISAPEIPACYFEVEKNLNYSEISKICQQHGMHVTSVLSKKENDFLTTFLTRKYGDSFKHFLGLTEQGTWEDGSPISFINGSFEHPVGITMFGWENLSNGPYYGMCKRFSCD*

>Llo|EN70_10316

MRNILLSTIWFHVLEVIPRCNGCLATSTVTTPKAPLKVTTYNPRTPTTTVAPNVQCFPIARQKRDFPSSASSASDSLSTTCLPTDYLETAVAEMKAGLIKSDSSNSERKVEENALFPLAISKHPAIAIEHRTINDHSNNGELSSSNHPAKKLCQDFEWDGPIYLYNQSLGVKIPYCYKYVASIDEETDELTRLTQKSAREKCRSYSGQSDGPSDLVSIHSSDENYDLQSTLSFFGWESVWIGLAYDNVRSTWRWIDGTTLSFSKLSLRDPSQHCAILLTNGSWVSEDCKSSAVSHFVCKKRAIT*

>Llo|EN70_11733

MPIAGGTSKSPEIVRWCGVKHPDVIISTTDSLYVYFHSDEAFQRKGINMSFIEFDMPGCPPNWISSSIGYCYILTEATHGLTWLEAQEECSLVRSNLLTLTSAIEYSSVAAIYTKSKTTPWIGYMDYSGEGRFTPVDPNSQPWPEEFVDGHLSNTENKKFLSGTPSAVLISAVMFSATSSRGTAKTDLIGTTVQITTSSKLLHSQANKLRQSIHLPKLIGSRSGQECAYIDWRNRKGEILAVDDCRNRHEYICKRRQDGTTIPYLPQKEMMRNGLAKSPISFNIWLFIILLVLLTLILLFVCYRKCKKQRALSRIGSSDANQRLVTGIDVQRAATSATVLHIGSSEIQKTNSVAINNNGARNDARVPTVTENASSVVQYRDSSRQAKEQNTPSQNSHSIRQLNLERIEPTVLTIEPRFNARQASEERLSVPSRSKQNLPFGIRESTMSSTTREETLLKIRRGELFERPRVSVLDHTSAISLDEFWNKNEMSTINRN*

>Llo|EN70_11756

MDIDIVINIYNASGNGTYWIGLLKDINGIFKWQSGESLNYTNWNKGEPEPRIGCVIASIMECNGKWLIINCNEMLYPDQGFVCEKDIRRS*

>Llo|EN70_1372

MFRSPKDQFPNDGVIVAEEEVKVAENENNELTEEFPCGKDPLFCPLKTNQGTVCYRLQTEPFYWEQAVKECDAKHQSDLISIHSKEEANFIYEMVQLQPSISGEKKYWIGLHRRNARSQYEWSDGSSLDYLVERLSNEDPDEERGVCVAIQFNISNKLLQKQSLYNLFGIPIQMSRKVPAYFWTHQRCDNRHLSICRKPGFDYHARSELSAKEERRLIKQNNWECSNGYKLFRGMCYKLYGTNANGVTFSEAIQRCKIDKANLVSLTDIYENGFVTSMLQNLNSNAWIGMDMTDGRVRWLDGEPLKLIRFGPDNRVIRIGGDRHIYQNVGQAGFSNEACVALDATNMIGYWDIIFNKTSKLYLFSA*

>Llo|EN70_325

MYVTNSTNHMKVENVSLFCAKENATLTTVNSNAERDFLYDLISHSTYYPSIASYKVLIDVQNDKWVDSNILLYTDCETKYPSSSECTAIRVESNGIMAKIGKWTRMNCDESVFAFLCKTKANKLPVLHRQCLKGYVYRPRTGLCYAIKSPKTNTTAYETAAISCQDELGMLVSVHSTEETEFITRELVPQRSNVTRIYLALIYNFDKNGGYSWADGTIFDYQNWNPTKARQNAENSCVEMDPKTGFWDQIPCIRKSKHDNTHTADNAGIICQTLPIFH*

>Llo|EN70_3335

MYLVSLLCSSLLLCLFPIVDSLSPCPPGFSYHVNSEKCFMVVKWLDEPMNQTNARLTCAKENAFLASIESDDENNFIKDLLVDKNAEIAYIGLQSVPYNVAHRYFSDGTTLGYTNWAPGKPDNDNLMDDGCVVIDAAEGQWDDWTCSANMVTGPWATVCQTRYLEKEDQKEEANCPTGFAYDSISTKCYKVIIKAEGITAWNAKIECANYNSQLVAINSKEENQFISNLAMKHPLSAMRVFIGLESDRTNLDARNRYWIDDTWVEQRYVNWEYDKPDNVPYYLNDCVVMIPERNGVWDDWACNLPRYLEHAVYGAVCEFRNE*

>Llo|EN70_4319

MFSEKCRTNNDCGSGRCEWRRCVGKLGKVISDRMEIENCYVDDDCPIKQVCRGGSCQERLPLISDVVNVRPDHCYTDANCPLGFKCGFYKKCYNMSDGRYVVESFMHIPCKHEAPSFCAEATGFENAICREIYFEHENVRSYICQFPPPFMYAEHKSCDEKGGCSRYGTCIRGTCYQPFWVTVRQRCKSNDECTFRATCNSDGGCEEGRWALRYRTCRVSSECGNGGSGSVMRYVCRRFECTYRRSVPAECDACRFDEFCDLYENCLRVQRFSELNCDGDNWFYWYSAFYCSSTTEKTYKDALLECMELKAKLVYSPDRGSETSLSPENALLSYLAIRAGWVKEENDEIEKNFVNWNFDSEKMKKSRFFCKKEVPRSSVPNNATAYIGQKCQTSFVYRHDYCYLAGFTNFMPRFAIRTICNLMDSEIVKIKNKEEAHTVYGLAGGKSFWLGLRKGKRDWYWDTSTYGTRAHFFFWRTGQPDITDNKNCAYAAYAGEWMSADCDSFRSPKIFVCQSPSYKN*

>Llo|EN70_4739

MLYGLVYFFFLSTIQATTSPCSDEFSATKCSNEQSDMICQKAFPVNEAFPIINAHCSNSKAHGVEECRKQCRSCCQHPDFMCTDKKDMFHSELNCRMIAEKGHCTTTDVTFKAILAVECASSCGLCRHGGCTDNNYLLCSMLQNLCELSDYHELMVKKCKRTCNLCTVDMQYPGWIYNKETNAHYKFINAISQSENISAVKYERMCAEEDAHLVSIHSEAENTFVYGLASCERVFIGLRGQTSNRFEWLDGSVVDFKNFFPGFPLGTHYCAYMTGDSMFWYTDSCYTKGCAVCKKIVKSLD*

>Llo|EN70_7414

MNTETYYWVQVIVRETNGGSKLGPCPEKWLPLIPTNNYALLEKCIAHVNVTLLQSKVTEVNGAINACSHVFGNESTIATIFRPNNKRELKNFLTYASEEALEGMIDIFVEGSAPYDHFGNDLRQLGDAVLNPLSTALDKQCAWNLQKYRTTARNARDVCKEKKAELVTISNIGEWSYVTRLANTLAPNTRDRSLLLGYTRCSTDPPEWRTMEGGISSGIFPFPNATTANDAECCLEMNIEHKINCKEYFYR*

>Llo|EN70_9690

MRWSDAERACINLGGHLASIMDEYENMFAFNVAKEANLSTPTLWLGRLVKLTQTGAYEWNDGAVGRHINGFRGELPSGTDLCLTMWLDFDRPEGSWNEWDCNYASGYSALCKRSLKRIPITTTVKPNIRSGTGSILLYHSRRCCLISSLCHNTSQSCTSDERCIPDDLDCWTNICRDGGIGWCLPLPKSHLYPE*

>Mar|M.Arenaria_Scaff10221g073326

MRYNLHIRIFHPKKVLGPPYGFSIFNRLYLGSKIFPRANFDATPVSDDTRTDLTTLIGTEGTTSTPNTSCPPDYTFFKEGNCCIQISSPEKKPLTWKEASEEAKKNGARMASIHFKKEDDFIRDLVAKQPKVEGKDNPVYFTGLNVNATNGKINNFAWSDNTRVNYGSPKDIKPNTDPWGPKTTFNGNTGFGGISKDPSGKASWGLYDPNQKGGYVWKIDLSKKVPEPGNPTGGSPEEGIVKGLPAGYKCTTGCNPSSIKLKAEKGQPSPVDEGPTEKNGCQTDVLSCTTNQPIATITFGTQGSLKSNQRDKRNVRAQVFCANEEATGKKGWVRNGLDNNPKLIFVEEAHCEQQSS*

>Mar|M.Arenaria_Scaff1022g018209

MKIPSFLVCLSIILFFNSLTAQKVPNCPARSYSCDTDWQLFTDPNSGVEYGYKAFIQDSINFYEADIFCRDRCAEVVSIHSQQEQDFVTSIAGPLLTKCQFTDACSERMEHTNKTAANFDRMLRGFWIGMNRVWGTLLNHNTIDTNITCIWSDRSKCDYGAVQDGSVTPDTVSPPWARGNPNGANPGGDPLIVQACVQMVEGRHGGWNDISCTYRLGGLICKRKCNSYCAAQDV*

>Mar|M.Arenaria_Scaff1022g018210

MHLSSILIILPILFSSIQTQQIPNCQTRIFQCDADWDTRTDPNTGDVYGYKAFIQNKMNFYEADVFCRARCAEVVSIHSQEEQNFVRRIAAPVLNRCQFTSACTPVVQSTNATFDRQLRSFWIGLNRVTGTLKDAHSVDSSVYQLWSDRTEVDFGSFPNNSVNTGTNSPPWTRGNPNGVNSADPQIIEDCTQMVESRQGGWNDFPCNWRISGVICKRKCTSYCAAQDV*

>Mar|M.Arenaria_Scaff103g003034

MKNIHTGHKKPRNRKIKLYNSYFNLLINIFVITTLFVNINGTESDESSTSVDDEEPVFSQSAQHLFKNENSNEDSGKHPSLEASSPSKNHRQHRKNPPPFVLADVQLSCAEGWERFEGKCYKLVSIEKSWPQALAFCSRFGAKLVRLESSEENKFLAKYLMRPHLTSGPTTSPSEYWIGLLYRPLMMDSSISSSSSHSKSLMDGSFLWSDGSQTSRYVGFWSYGQPDPANGSCTKILVDGLQVEGPTWQLDVCNQLRPFICEQNACIKGSFFCQNGVCLPERAHCNGIDECGDSSDEFNCPSAHSEMSCQRYEKGESGRIETPNFPASYRQGSNCRWVIEGPLNSKIQLNFDSFETEERHDLVTVLDGGPSENSTFALSTISGTPRNTEKLSFESSTNRMIVQFRADQSIQARGFQASWRTAYIIRSYTTVKNSGSQXEKLSFESSTNRMIVQFRADQSIQARGFQASWRTVPISCGNQQLKASSIGQQFHSPEWPRNYPKGLECVWRIEAHSGQLISLSIDEFNTEAETDFLTIYDGPSPSEPILAKFSGQMKEPQLIISTQSQVHIYFFSSETVPQKGFTITYKKGCDNSIRRSHGVLTSPGNAHLPYAPSQICRWSIELPSQQIENFEAAAEIPSLSLVLNSWDVADLGDKLQIYEGGEDGSSNGRPLHESDGFTVNNAPPKTIYAKQGRVELVWRSNVLNSGTGWNISFSTSCPPLSLPSRRVLLSTKNTAYGTHVTISCERGFEFSTGLGRHFETECELGGLWSNYAPVPDCQPVYCSAIPQIANGFAVSATNVSYAGMARYQCYEGFSFASGKQHEEIFCTDEGRWTQAPKCKTDACPALPSFVSGERILQFGDGIGFGSVFQFHCAKGYFIEGPLSIVCRPNGEWSSPQPLCKKLTCTDIPIVENGELHLLPSNNMAMDEKSNKDKKPKDQTKNFGSRQKMEFKTKRNTVLRELQFGDSLRVECHSGFQSVGAETLKCLANQTLSGIPKCRDIDECELQSAGNCAGKSTTCVNMPGGFHCQCQSGYKPKLDCSGPLTVIPTKIQTSHGVPIPVQQLNSKNGWCADTTITNAQMLEKRIMPFNESTTSSSPSTILTLIFTFPVPKLQFGDSLRVECHSGFQSVGAETLKCLANQTLSGIPKCRDIDECELQSAGNCAGKSTTCVNMPGGFHCQCQSGYKPKLDCSGPLTVIPTKIQTSHGVPIPVQQLNSKNGWCADTTITNAQMLEKRIMPFNESTTSSSPSTILTLIFTFPVPKIIEKLHLEKVVVPNASAPSGIAGAIAATPEAWPQRFTLSYSTEEGMPFEVYNGGLAEIGGNNNITSSNENKKSNERKLNTPNTKEIRTRALGAIGSEILVLAKPIEARTLQIEFLEFHGGVPCMKFEFLGCQRTSCEDINECEDGRNGGCEQHCHNTQGGHRCSCEEGFDLFVEPGQSGVRLRECETGYGEFDSLRFNHSCIPRHCAPLMTPENGQLIAQNLYKNFKNEVNNTNNKNEFFLSSFAFPSIVEFRCTFGYQMRGPSHLKCLADGTWNGTVPSCIPATCSGVKNSTAVGLFVQPETVSIPFGQNLSFVCSQTNRPPKHSALGEIRQCIYDPRTDGLEYWLSGPEVDCPLVDCGPPPALSGAYYEGDEGHHGGNFKVGSVYLFQCRAPYSLVGKSSYDDRMVRCNVDGTWDLGDLRCEGPVCVDPGHPDDGQTFLDSVEEGAVASFSCNRPGFKPFPAETISCSLGTPCVLSEDVGISSGFIPDGAFSDNSDKVIWGYEPHKSRMSSSGWCGSKDAFIFLSVDLQRIYTLTTLRLTGVAGNGHLSGHVTKMQLFYKVQFSQNYDNYPMVIWGYEPHKSRMSSSGWCGSKDAFIFLSVDLQRIYTLTTLRLTGVAGNGHLSGHVTKMQLFYKVQFSQNYDNYPMEFSTPSGNHRKIYQFTLNPPLRARYILLGITEYEKNPCLRFDMHGCLAPLSTTHEVPAHLQVGWNASIPQCLDAEPPTFKNCPQSPIIVQTDENGQLFPANYVIPEATDNSGRITYMLTKPEDFHPPYPVSQDTDIIYQAFDDAGNMAECPVRLRIPDTVPPILKCPDSYAIWAQENQTELHMHFNESSVRLVVQDQSPITQISYDPPEARIKLDSHVTVEASVLDAHSNRNKCKFQVALLPEPCSPWSLRIDEATVQKQCQRHASGTVCQVQCRKGYRFLESFPQQKSEVKKQQNSTNTLPQRYSCSMEQQSGKWLPSPTPPACVPMAMEPARYEMRVHMNYSLTSPLPSDCAKSYELLVGSLFDSIDQVLSQRCSSTVQIYVRFLDAKFSQMGEKTMGANFTVQILPTVLQQVFYELCSLTLRTIFDLRIPGATIPIRSLLTLSGDSVPAILNLGCPPINASSISVSQGFSCSQGELLKLQTPNVGSDDFLSAAPGLPECFPCPKGTAFVNNSCIQCPMGSFQDQEGQIRCKPCIENTYTLQSGAQSNESCLDVCGNGMFSATGMIPCQLCPRHTFAGPPPIGGYKECEPCPEGTYTARLGSVGPSHCKQPCAPGHFSVTGLEPCSPCPINFYQPNIGQQRCLQCSNDSFTAETGRSADEHCKKLDCQTLKCQNRGQCVVANHKEVCECRPGFMGSHCEQQIPLCDSHPCLNGGTCELHNGAFRCICPQNYTGSRCQFGPDECISSVHCPNGGVCQDLPGLGTTKCICRTGFTGPDCSQISDPCQSDQPCKNGAQCIPLQLGRYKCKCLPGWEGTNCDKNIDDCTENPCALGAKCHDLINDFECECPHGFSGKRCQIKDNLCDPSPCLNGGQCVDTLFDRHCICKRGWNGTFCEQEVNECSQKQCQNGATCRDQEDDYSCECAPGFHGYQCQYMIDHCAVKPCRNNGTCINRGPIYECQCPLGYEGDHCEHNVDECEMMTPCDAVGTGRCEDLVNGFKCHCHPGYEGTFCEQHVSQCEEEPCMNNGTCTDLGAGFQCECQLGWKGDRCQEMETQCDRKPCMNEGKCVPLVDDYFCVCPEGVSGKNCELAPNRCLGEPCHNGGVCGDFGSRSDCQCPKGYSGNGCQFRFDGCHEGLCKNGGTCVNNNDLSKHQMSKIENAVAVEQNSEAIGFKCICAPGFFGNECEIDINECQPSPCPLASHCVDLVNGYYCKCPFNMTGANCEKRIDPDYDLRFLESSSQPASASLGIPFNFVSSALTLNIWVKFEKNHQTDVLKEQRKSPPIFFTLYGSSSANQPTNLTELLTISSEAINIRLFPELDKQPLVLHFPIHQRPDTQLAWNNIIFMWDSQQQGSYSLLWNAVRLYSDKGYAPDRRLDINAWINLGDPKGIEQQQNQHLMTNKIISTEPKVNEKIIGTKFVGSITRVNMWNRMLDFETEIPSIVQRCQGSPDLYEGLSLRFANYDKLQGKVERIAKSTCGRIDLLSPTCHSSGDENKNLKDHQCRSRIPSNMKDYFKEELVEVEGCPMEPINVQTPLKELNISWKEPKFYETSSRVQIAKIEQNLKPGQVFTWGQYSAIYLALDNQSSPLATCQFKINVVREFCPDPEIPVNGVQRCEQWGPGLRYKACSVHCQNGYGFSRPPAAFYSCGEDGKWRPNEGKQRPFRYPQCTRQSPAEHLARIQLSYPQLALCNPAGKSTVTEKIIERINQINNKWHICANNEESSDCSGVQVQINCQDIFAASRLKRQSTLQQFDVIIDVPIREIQRMDPVEIIRDEAMAHGLFSLEQVIPNGRPDIGAFRVENAFRCPIGYLLNNGSCVPCAPGTFYFVPISECKLCPIGQYQPEEAQNQCIECPTDSPMTVGMGSIKQNECRIRCLPGHHLNISTGQCEPCSYGFFQPDSGAFDCIPCGIGKTTLERTAINEDQCRDECPDGQQLTASGSCQPCPQGMYRTRGQDKQCVECPSGTTTEGVGAGNKALCNTPKCGAGQFLLADIKRCQFCPRGTFQDQQLQFECKKCPPSFNTAEEGATRESQCYSTDQCALGQDNCSWNAQCIDLPDDNDVASWRCVCNPGFRGNGINCTDACLNFCLNDGVCRKNKLGHVECSCKENFSGERCEIRFQPRSQKLVYWTGAIVAVVFLLIVIVVVIWMISLRFSRSSDNSFLSSPLDKPALSFTQSTTDSPLASNFLYGRPPPLLGRSHSSSLGGGGSNSIIHPIGFYYEDEQSPYDGGVGSAVGRHGRENSQEMKSIFLASTVPDNNNDVVGGSSSASHSPGQQNNNGGIKVTGTTNTTNSSGGSPGSATRALEQRLRHIQQHMYRPMGDG*

>Mar|M.Arenaria_Scaff11047g075792

MKKRESYINEEENNGKTKLSSNSIEKRLDRLEANQQNLRRATLADWPAFSVDKRVRIFDHTRSTWIDAQRECSEHAGTLLEIDSESENERINEMLTNSGSSNRPNDHYWIGVQIMLQFSNGSSIIGNYSNFEEQNNSENTQNQKLSKGLVMKRCAAISASQTTNSEGRWLSLECSEKHGFICQL*

>Mar|M.Arenaria_Scaff1136g019637

MLGTSLRCLVASLLVLQAYANKMDPAYQTDDDRNQPVESVSSEQAPWISGPQNQKYQFHIGKQSWLSAREKCLAQNADLVSIGSIEEMQWLLSHYKPQFNHLRERQVQIGLLLDTIEGEAGSNSDSGLTSREWRWVNGKPLNLEITKWTMGEPFDHAKGKERCALLNINERRLDDVDCDLGSGAGFNYRFVCQRSHEKHIEHESLNNPLWKKLEDILTFFGISDREDDKKNCSLPIGAKEEGYWERAFNKGEGNSETQHKEKTIEKTDSITEKSELENPKGVEKNSKTNKEDRDSVLEDKTIIEKKVITNKNDETTNKLSSPPEQKLVKDVEKVVEEKKVSKVGGVTQHATSKIIGGEDIQSPNELENTRPTGGAVVLRRVESHVSASSNVNNKKNNQEKLKEGNEEAKSTKLEEEQSKILKVAGTEEANLRDKEASDSSDSMREQSGLSVREKSQIINLNTRENLQSSLNPKKPLIISNDPSDLTEDRNTISNKNTSDNEKENKSIQNLSESVQITEKKLGEDEISEIKQYTLENKYIVNTPKVDKKNNEKIKEDKLNAEKIGEEKISEKEEKKEEKNEDKIKNGDKDDRKGENEKITVVDERKTKKENKEIKEIEENNKEDKEVKRDGERKEENEKKSDELKKDEVTRGEEIKDEKKVIKEKDIKEEKLKSEETKTRKEKHDEKEEKEDRKNLLPKSNNIRDESKKLDLEDKEEKLEANLIKNKINNEITDVKKNEEVLAQNKVEEKIREEQSLEFRNKDKKDDNKSVKKSENVEAIQEKTAEIKEAKQDSNQESKKANNEAKDGLNNETTHELKNEATNSKNNEVSQETKKEAKLKENKEGKDEAKHEAKLEAKHKTKIEATEANEKESVNGAKSEAQSEARHEANKVSSSNKGTNNEAKSKENNEAETNINEDKTIKTNKPTKHPDPLSVVAETLERSLSSKEETIEKKKDEAENLIKERRQKPDGVRDRIQHLEKIITEVQHMMGIGEKGGEGELIEKTNKVLEEDEEEKKYEIKKEEGRKNMKEVKKEKEEKIRVEEKEESKEGSGSSEKLTEATPIAEVEELSSPEVEPKSHSPSVSSSDESEESEDSLKHVKTKNIKDNKAQREQPVNVNNEATRKSGAVNKSHSIISDSLDEQRKSMAAMEFDFEEAALQAKAHPGINSGDLVKNIILIGKDEELNGCKEGKECKDKKAKSKQNRRYAPRNEEKWEIINKMTDDINAKKGSRIEKYGRKSIGNKKRFEGDVVEGKDGEELNLVKNNHDEGLLNSIHSETIEATPTGHILDQLIIFHPNDGKRSRESQIISQMLLPDGGFHGKVTGDGKIEHRPADDDWVIPEVETQTMLLNSLNSSSNSSHSSTPLSIFPAPDFSIIKPGDQVPPQKIIQRLEEERKRIDAVYEKLNKGDNIYDLIKRIKASDARRQPLTVLQSKMTPN*

>Mar|M.Arenaria_Scaff1141g019702

MKLNFVFLISFFIFLFFSYTTEAHFGCNCQYPNNHYVPWYLSGNVENRLTFLERQTNQKINMLIARIEALERELGLIHRISMQEWNNSGAGNIYKIFNTSKTWEEAKNTCMTFGARLASIDSDYKNAFVRSERTLIENKYVNFTIKDMIERSFGKDESAEVWIGLKTRAELTNNPNSHFTNFGEEEKIDGCAVMGIKGKWKIRSCSNLKPFVCEQILM*

>Mar|M.Arenaria_Scaff114g003306

MLHQQQQQQQQQKPKFRLQLEDVGNPQPCPDNPIRLLASRTPQYIYSPYDENKLYPPDTDCQFLIEATDKFHRIHLTIIESDLEEALFTDCNDYVSVRDREGNETSIKEVARWCGQDYPAAIASASDSLLVHFHSDSIIQKRGFNISFVQFDIDTCPPDWISDGISSPYCYKQFVLPHILPWYEAQKECNFERANLATFQNEADYAFIVESYSQTHSFPWVGYSDANVEGIYESIDRNVPLWPENFPLKHENEMKDCVYLDWNKRDQIVVYEIDDCRNRRPFLCKKRRDGTEVPVILPAGMIRRGFRDFTMDYTLLVVVVIFALLALVVGCVLFHKYKERNNRIINIDMNQRLVQQQQGQQPGNKDKAAAALARQKAKERERRELREQKYAETSNNNSNRGGGRGGGGGIAATTKSPFESQDSNASFPLQTFQSTFAQRMTTTTTGNVEPLDTAAALAEASASASQQMREHEATKLHPQQLSAEILSVDSPPEDDLNSSPRIRMSPNQPSFVEEGRKISEKKIRKGWMGAGVSGGGTTDREEEEEEKDRHNMGYEPDSEEEEHEENERQKIRRASKINGEGEGEGEENFLMELNDEGGRKNTGENEELEQYTQHNHVEEELEQYSGGEQFKQYKHKNVIETEQYINSQQFKQLESADGGEIQQKVVQVELHGENGEEGGLGQFKDKRTKEERINNFDEKMMDSYAIGDHFKAAKVATSAIASAAAIAATEHPIRPPIELMRTRTTSTDKAKSPPPLKEAKESEKIGESGKSSREATREGKEETREEKETFPEKVSGKVNIPSSTATNIVKTEGEGTGTRIRIKRKTFDRPPPVGPLDNVSAISLDEFWQQQK*

>Mar|M.Arenaria_Scaff12243g079028

MKKRESYINEENIGKTKLSSNSIEKRLVRLEANQQNLRRATLADWPAFSVDKRVRIFDHTRSTWIDAQRECSEHAGTLLEIDSESENERINEMLTNSGSNNRPNDHYWIGVQIMLQFSNGSSIIGNYSNFEEQNNSENTQNQKLPKGLVMKRCAAISASQTTKSEGRWLSLECSEKHGFICQL*

>Mar|M.Arenaria_Scaff14882g085346

MKLNFVFLISLFIFLFFSYTTEAYFGCNCQYPNNHYVPWYLSGNVENRLTFLERQTSQKINMLIARIEALERELGLIHRISMQEWNNSGAGNIYKIFNTSKTWEEAKNTCMTFGARLASIDSDYKNAFVRNMIERSFGKDESAEVWIGLKTRAELTNNPNSHFTNFGEEEKIDGCAAMGIKGKWKIRSCSNLKPFVCEQILM*

>Mar|M.Arenaria_Scaff14g000640

MKLSVFKTLILTYLSIQCIHFAFINADCPAGWNSRSDSSGIEYGYQVVLQKNITFSQAQSICSGLESDIVSIHSKEENEFIYGLIKTSGVVLYIGMQQKSDQYSPTCAWPDKSDCNFGNFNGVKDPKRQEYPWVRNPSITTDGNTVHCVGIADTTYNFGKPNFKWNDVGCNDGLDGTICKKKCIGGNSGGSASTSQTPEASKAPTILPSNTEAPVVSTKSSIGGGSSIGPVVTSKPSGAPVVSTKSAIGGGSTIGPVVTAQSPKTLATLLPGFPGSTPKPVNGGGNNGSCGNCNCNDGYKCGSDDWQVKHGKDGSFAYKYFPAPNSSYFTAKAICAKYHALPCSINSEEENEFLLENVANNQLKSNKRAKRETQDPACIWTGVHIVVSDNDKEENECYCDDGKECSYGHKKENNEDDDDDTKEDGHDDKKKKEKEEKDKKKKEKEDKKKKEKEDKDKKKEEKDKKKEDKDDKNKKEKEEKDKKKKEKEDKDKKKEEKEDKKKQEKEEKDKKKKEKEDKKKQEKEEKDKKKKEKEDKKKKEKEEKDKKKKDKKDKSKDDSDEDDHKKCSCKDKKKPPSPWAPGCPSKPGGGDKKHCVGIGNDGKWMDVNCEKPGTGIICKRPCSP*

>Mar|M.Arenaria_Scaff14g000646

MKLSFSKINLLAVAVLISFSFVNCGCPDGWGTIDNVNGFKVFTSTDPINLFMAVPVCKANGGIIASLYSSDESAFIAQMLGNDTPVWVGAVMKNNGDGSFSCKMLDGGDCPSEIQNSFQNQPTDTETPIYGTQLISGKWNVVDCAERVYAVACKMSCDQ*

>Mar|M.Arenaria_Scaff1513g024059

MAVPVCKANGGIIASLYSSDESAFIAQMVGNDTPVWVGAVMKNNGDGSFSCKMLDGGDCPSEIQNSFQNQQTDTETPIYGTQLISGKWNVVNCTERVYAVACKMSCDQ*

>Mar|M.Arenaria_Scaff1555g024489

MTLIGLIIAFCIIPSAVNADCLSGNWTTRVATDGNTYGYQVLARDWLNFYEARALCLGVGGDVASIHSNAENEFVRQLAAPYIIGCQTNKTTCGARATTSLDTILSVVWLGMHRCQYFPSYNATVDCVNSDGTPCDYAATNAVILTGPSPFPWAWGNPSGSDSGGGAGLIEDCVSMYNATSGEWNDLSCFQKLGGVVCKRNCTGTCGSSSIKTALITSPSDITDTSCTSGNWTKRAGEDGNTYGYQIVMQDWLNFYEVKSKKYTRHLIKFFILKANAKCLALGAEVVSVHSVAENEFIRQLSAPYITACQTNTSVCATIFSSLWVGLHRSAFYPSYNSTVDCINSDGTICDYFNITGGPSGTETGGASGQQEACAAMYSATTGQWNDIACFNKLGGVICKKNCSNACGVASTTTTQLTTTTTQPTTTTTLPTTTTTLPTTTTTLPTTTTTEPSTTTTAEPTTTTTEPSTTTTAEPTTTTTEPSTTTTFNCAQPPVTSLLSYNTSDPKIQTGSNATQGVCECPADPGNSNVFFIPVTTITTGSSASNSSVIMKCSKMQDFCICDEDDVCWKVINAYSLVVINSFCDPTCHNYARLQNAAPFNQTFESECGRTITLADELTPIPNTNRNTFKPLGSSADYYIKAASIRCLQAGQTCTPIKCSGTRKPSPCPTTTTTMPTTTTTLPTTTTTLPTTTTTLPTTTTTLPTTTTTLPTTTTTLPTTTTTLPTTTTTLPTTTTTLPTTTTTLPTTTTTLPTTTTKLQTTTTTLPTTTLAPLNCGNCATGQSRVIYDKNTLSSNQIVKNNAASQCIFNCKDPDVSQICYTTDAVSVARINCTDPSQFCVCASNVKKGCYTVTNTALLADYSIFANASYTFLVINSVSSSIANSQTGASYKFTAGPSFVVGNSKTLNAPVLGISCNGCAPIHNPGADVIKPCTPGGG*

>Mar|M.Arenaria_Scaff17036g089683

MYAIRSYYGRMCFLSPAEVKCRSMGAEILAAPLLEECQNNPLICAERVPHIDKAHELLDHILRGFWIGLHRNQFYPFYNPAVVQVWSDRSECDYGCYEGEVYCDMNIEPWGNCDPSGTNSSHGHGQSEDCTGIYLATSSGFICKKPCEGEHKHDVCGKDGWPYVDGKAFHAFPLHSPGNYWQALSICHKHDAQVASIHSNEENKITSALAIGQAGGNISACSWIGLHSAKGGSKERFWDDGSNVDYGKHVEPGTLPLSDDISSDDKSKDTHHCHECAAINGNGHWKDLNCGANCGAVICKKGCRNPE*

>Mar|M.Arenaria_Scaff17036g089684

SSDGQSEDCTQIYLATSSGIAKWNDLACNQKIGGFICKKPCDGERKHDVCGKDGWPHVDGKAFHAFPLHSPGNYWQILSHPLVFVFKTARQLDPSPSLHYINSLCGKDGWPHVDGKAFHAFPLHSPGNYWQALSICHKHDAQVASIHSNEENKITSALAIGQAGGNISACSWIGLHSAKGGSKERFWDDGSNVDYGKHVEPGTLPLSDDISSDDKSKDTHHCHECAAINGNGHWKDLNCGANCGAVICKKGCRNPE*

>Mar|M.Arenaria_Scaff171g004614

MFPNWALLLPIFGLFAYSSAQSRCKFICEADWQTRVEPTDGKTYGYKVFFKSALNFFEVNIRDYIILNFSNIFQFKAQRICRDNCAEVATIHSRDEYDFVFLTFRDLLDQCQTNASVCPIRDPNPDFNRILNSFWLGMHRIQQYTSICQGCSVDSEIVYVNSDGTPYNFGTYNADRAVLEDCTQLLDSETGKWNDIPCHYQQAAVVCKRDCASFCQAQVPPITTTEATTTILSPGGQPGGPGLPGPNGPNGADATTSAPDNGNNPGPGGPGGNGPPGQNGPNGPNGADATTSAPDNGNNPGPGGPGGNGPPGQNGPNGPNGADATTSAPDNGNNPGPGGPGGNGPPGQNGPNGPNGADATTSAPDNGNNPGPGGPGGNGPNGTPGQNGTNGPNGADASTSAPDNGSYNFV*

>Mar|M.Arenaria_Scaff171g004615

MKPAPLYFTIIFSFPIVLFFLNFNIITAQKSTCVPFECNDTWDLFQDNDGKVYAYKAFVQESINFFEANALCREQCAEVVSIHSKEENEFVRAIAQPLLDDCQNEIICKSRPGETSPDFNRILHSFFIGMHRIQFGRLGQCTRDPNIYCIWSDGTICDFGNYTGVIGPHETVPPWATGNPNGIEQYGNEDCVEFYDSVNGYWNDIGCQMRLSGVICKRLCNSYCAEMDTQLK*

>Mar|M.Arenaria_Scaff17210g089981

MQEWNNSGAGNIYKIFNTSKTWEEAKNTCMTFGARLASIDSDYKNAFVRSERTLIENKYVNFTIKDMIERSFGKDESAEVWIGLKTRAELTNNPNSHFTNFGEEEKIDGCAVMGIKGKWKIRSCSNLKPFVCEQILM*

>Mar|M.Arenaria_Scaff1802g027090

MMLVILTSVDDEEPVFSQSAQHLFKNENSNEDAEKHPSLEASSPSKNHRQHRKNPPPSVLADVQLSCAEGWERFEGKCYKLVSIEKSWPQALAFCSRFGAKLVRLESSEENKFLAKYLMRPHLTSGSTSSPSEYWIGLLYRPLMMDSSISSSSSHSKSLMDGSFLWSDGSQTSRYIGFWSYGQPDPANGSCTKILVDGMQVEGPTWQLDVCNQLRPFICEQNACIKGSFFCQNGACLSERAHCNGIDECGDSSDEFNCPSAHSEMSCQRYEKGESGRIETPNFPASYRQGANCRWVIEGPLNSKIQLNFDSFETEERHDLVTVLDGGPSENSTFALSTISGTPRNTEKLSFESSTNRMIVQFRADQSIQARGFQASWRTVPISCGNQMLKASSIGQQFHSPEWPRNYPKGLECVWRIEAPSGQLISLFIDEFNTEAETDFLTIYDGPSPSEPILAKFSGQMKEPQLIISTQSQVHIYFFSSETVSQKGFTITYKKGCDNSIRRSHGVLTSPGNAHLPYAPSQICRWSIELPSQQIENFEAAAEIPSLSLVLNSWDVADLGDKLQIYEGGEDGSSNGRPLHDGDGFTVNNAPSKTIYAKQGRVELVWRSNVLNSGTGWNISFSTSCPPLSLPSRRVLLSTKNTAYGTHVTISCERGFEFSTGLGRHFETECELGGLWSNYAPVPDCQPVYCSAIPQIANGFAVSATNVSYAGMARYQCYEGFSFASGKQHEEIFCTDEGRWTQSPKCKTDACPALPSFISGERILQFGDGIGFGSVFQFHCAKGYFIEGPLSIVCRPNGEWSSPQPICKNLLKSSKFSPAAQKRKEFVSFAFQNPQNLLPGLKMKDFWLKDLPKLKKFSPAALKIEEIKIYESSATKMQ*

>Mar|M.Arenaria_Scaff1808g027149

MINYFQAELRCRSMGAEIVSIHSAEENSFVALLAECQNNPLICAERVPHIDKAHELLDHILRGFWIGLHRSQFYPFYNPAVVQVWSDGKQCDYGCYEGEVYCDMKIEPWGNCNPSGTNSSHGHGQSEDCTGIYLSTSSGIAKWNDLACNQKIGGFICKKPCDGEHKHVCGKDGWPYVDGKAFHAFPLHSPGNYWQPLLTKRSEEIRTKRLSHKKYRAGEGLTPIHNRMHLWTQRCKEQGIQTEELPNFFKSHL*

>Mar|M.Arenaria_Scaff1830g027373

MWLFTWKFKQKNHEAARALCLGVGGDVVSVHSNAENEFVRQLAAPYIAACQTNKTVCGSRATTSLDFYLSVVWMGMTRCQYFPSYNSTVDCVYSDGTKCDYATSSSVYPWGVGSPSGSDSGGGAGLIEDCVSMYNGTSGEWNDVSCFQKLGGVVCKRNCTGTCGSTSIKTALITSPSDITDTSCSSGNWTKRAGEDGNTYGYQVVMKDWLNFYEANAQCLALGAEVVSIHTVAENEFIRQLAGPYITACQTNTSVCVSRVSTTQDIQWRSFWLGLHRCAFYPTYNATVDCINSDGTVCDYLNITGGPSGTETGTASGQQEACAAMYSATTGQWNDIACFNKLGGVICKKNCSKACGVASTTTTQLTTTTTQPSTTTTAKPTTTTTLPTTTTTLATTTTTLPTTTTTEPSTTTTQPTTTTTEPSTTTSFNCAQPPVTSLLSYNTSDPNIQTNSNATEGVCECPADPGNNNVFFIPVTTITTGSSASNSSVIMKCSKMQDFCICDEDDICWKVINAYSLVVINSFCDPTCHNYARLQNAAPFNQTFESDCGRTITLADELTPIPNTNRNTFKPLGSSADYYIKKAFPLSYDHNYNANNYYYPTNYHNNLANYHDNPTNYHDNIANYHNNFTLNHNCFANYHNNPTNYHNNLANYHNNPTNYHDNLANYHNNTTNYHDNLANYHNNTTNYHDNLANYHNNPTNYHNKLTHNHNCFXTTLPTTTTTLPTTTTTLPTTTTTLPTTTTTLPTTTTTLPTTTTTLPTTTTTLPTTTTTLPTTTTTLPTTTTTLPTTTTTLPTTTTTLPTTTTNLPTTTTVLPTTTTPPINCADCSAGQSKVIYEKNTLLSNQTINNLAATECIYNCKDKDVSQACYNPSPISVVRIRCTDSTKFCVCTSDNKGCFTVTQSAPLYQADYLIYANASYTFLALNTGASQIKNSGTGKTYDFQTSMTFIPNDNTFLNAQYLASMLHYFVRYYCPIANNNVSCNGCNLIHNNGMDVQKPC*

>Mar|M.Arenaria_Scaff18594g092419

MKPAPLYFTIIFSFPIVLFFLNFNIITAQKSACVPFECNDTWDLFQDNDGKVYAYKAFVQESINFFEANALCREQCAEVVSIHSKEENEFVRAIAQPLLDDCQNEIICKSRPGETSPDFNRILHSFFIGMHRIQFGRLGQCTRDPNIYCIWSDGTICDFGNYTGVIGPHETVPPWATGNPNGIEQYGNEDCVEFYDSVNGYWNDIGCQMRLRWGYMQTSMQLILC*

>Mar|M.Arenaria_Scaff20685g095772

XERSLLDAPPINVKSKGNLDNDDRNKIKNVLDWNKQRAPVICGDEEWHYFDGFCYKLINGKFTWEEALGECKKQNSDLGSIQSEQENDFVGSLSTKFDQNFDNFCHGFWIGMTRKYIEETDSFKSEWSDGTLVNYGNVPIRVAFHTPPWMGGQPDFAGGVEECVHSYPKLGCDNWQQIFFNQWNGRKIREEKIFKFISVDARCSIKLSGAVCKKRQSIQKI*

>Mar|M.Arenaria_Scaff20871g096061

MHLSSILIILPILFSSILTQQIPNCQTRIFQCDADWDTRTDPNTGDVYGYKAFIVNKMNFYEADVFCRARCAEVVSIHSQEEQNFVRRIAAPVLNRCQFTSACAPVVQSTNATFDRQLRSFWIGLNRVTGTLKDAHSVDSSVYQLWSDRTEVDFGSFPNNSVNPSTNSPPWTRGNPNGVNSADPQIIEDCTQMVESRQGGWNDFPCNWRISGVICKRKCSSYCAAQDV*

>Mar|M.Arenaria_Scaff20891g096089

MKILYFVVCLSIILFFNSLIAQKIPNCPARNYNCDTDWQLFTDPNSGVEYGYKAFIQDSINFYEADIFCRARCAEVVSIHSQQEQDFVTKIAGPLLTKCQFTDACSERMEHTNKSAANFDRMLRGFWIGMNRVYGTLLNHNTIDTNVTCIWSDRSKCDYGSVQDGSVTPDTVIPPWARGNPNGANPGGDPLIVQACVQMVEGRHGGWNDISCTYRLGGLICKRKCSSYCAAQDV*

>Mar|M.Arenaria_Scaff21037g096304

GNCCIQISSPEKKPLTWKEASEEAKKNGARMASIHFKKEDDFIRDLVAKQPKGEGKDNPVYFTGLQVNATNGKIGNFAWSDNTRVNYGSPKDIKVICFKK*

>Mar|M.Arenaria_Scaff21711g097276

MKLSVSFLNLLTLVFLLQCIHFVYVNGDCPAGWSSFTDSNGTEYGYQVVLKNNVSYFEARRLCLDINSEIVDIHSREENEYMYSLASNTSAQNLFIGMHYKLNETSRTSEVYCTWTPDVKCDFAYFQNQSDSRRQQYPWVPIPKAQNNGNPVECVEITDTSLGIGTPAFMYNDIACADRLDGAVCKMTCSNGNNPATTFGSSTIINGQNVVTISTTGSGGNNPGPRPTPPTRTAGTGAPGLPGSPGLPGLPGSGGNNPGPRPTPPTRTAGTGAPGLPGSPGLPGLPGSGGNNPGPRPTPPTRTAGTGAPGLP*

>Mar|M.Arenaria_Scaff249g006304

MFPNWALLLPIFGLFAYSSAQSRCNFICEADWQTRVEPTDGKTYGYKVFFKSALNFFEAQRICRDNCAEVATIHSRDEYDFVFLTFRDLLDQCQTNASVCPIRDPNPDFNRILNSFWLGMHRIQQYTSICPGCSVDSEIVYVNSDGTPYNFGTYNADRAVIEDCTQLLDSATGKWNDIPCHYQQAGVVCKRDCASFCQAQVPPITTTEATTTISSPGGQPGGPGQPGPNGQNGADATTSAPGGGDNPGPGGNGSNGVPGQNGTNGQPGQNGTSFTNSPGEGGNGNGDLTTTPEPTTIEPVTITSTMRAPLPPPAPRRRSSRSPRSTQETTTSNPNIVS*

>Mar|M.Arenaria_Scaff249g006305

MKPAPLYFIIIFSFPIVLFFLKFNIITAQKSTCEPFECNDTWDLFQDNDGKVYAYKAFVQESINFFEANALCREQCAEVVSIHSKEENEFVRAIAQPLLDDCQNEVICKSRPGETNPDFDRILHSFFIGMHRIQFGRLGQCTRDPNIYCLWSDGTPCDFGNYTGVIGPHETVPPWATGNPNGIEQYGNEDCVEFYDSVNGYWNDIGCQMRLSGVICKRLCNSYCAEMDTQLK*

>Mar|M.Arenaria_Scaff24g000943

MSITSLIPLILFLLILPYYAHCSCSSKWTTRVDSDGSTYGYLAIISDLITIFEGNTICKQQGGEVVSIHSPEENNFIGQIAGPLMKLCQETNACQQRVDHNNNRTAFLDMLYRSFWIGLSRVQFAHPLDNPTVHSAWLDGTPVDYGRYDGLPIPNRDVRPWAYGSPSSLNATTSGEIEGCTQMFLNDNDPQWNDISCYSLLGGVICKRNCSANCPNEDNSTTTTSSTTTAVGLTTQTTEETTTEVQYDEATTEDIETSTPNSCGNNSNICGADGWKWKCFNNGKCYSYHKYSVKGSYWKALAKCRKHHASVCSIETEEENEHVCKHYCGCNGKDVWIDMHRMVTEDGTDIVCLDGEDNKCYYETGLTYGRQALEALTRELLKDSTSKNPWANGCPANASSMDDGNYGKKNCCVISKGKWKDVGCTRPISKVLCKKECVKP*

>Mar|M.Arenaria_Scaff24g000947

MIAFSSAKLCLFIFSVALIAIYCILPTNGQATCPSDWISRVDPIDGITYGYKVITRDWLNYYEAQGYCVRAGGQVASIHSAAENAFIANISAAQLKQCQTNDSVCATRIAHTSAGWAFLDSLMRSFWFGMNRVQYQPSYNNTLDCSYPDGTPCDYGRFDGVANANTNSPPWSPACPSGSNSSNGAGDLEYCTMFFNATTTLVWNDMSCYQKLGGVVCKMNCSATKTTQTCASPSVVTTNVAVTTKAAVVTSGSGSAGGSTAKSLTTKSSGTSNGSCSCNGGSCGGGGYKCGSDDWQWKINKKNGKSYAYKAFNYTGCYWQGLAICKKYKAVPCSIQSNDDNDFIVKTVCSGFLSSSSSNSSRKRRATSDTCIYTGIHRQVTNPSSGGVSCTCSDGSSCDYGNDDAGLAICKKYKAVPLCSGFLSSSSSNSSRKRRATSDTCIYTGIHRQVTNPSSGGVSCTCSDGSSCDYGNDDAHGDTTTTSFTSTTSRATTLKTSTTQKAATTTQKSTTKLATSFISTSTASPSSPWAPGCPANSTTGQRDNGGYGSRNCIGINGDGKWVDQSCDRPATAIICMKECDQP*

>Mar|M.Arenaria_Scaff250g006344

MLHSITILLFIFHLAIAQNGDNLNDYEDIIMTTANYNAGLNADNANAKPSTASTTTGDNIMVLENLNNATGNPADQADDSSDKPLYPNSNPNENGGANQCPYGWATRQDKDGNVYGYKVIMQDLVNYYQARLLCIKEGGELVSIHSEEEEQFVVQLATPLLDKCQTDTSVCKQRVPIPSLDKMYRSMYIGLNRAAIEPYYEPTVVQAWSDGTPVDYASVPTPGVPTTIIPPWGAGCPSGLNDTGNTPQKGSPEDCVCMFKYDEVNWNDISCYHKLGGGICKRKCNGGGGGGGGQPCGCGKVNCGTDGWSQSANGKGYKIFQLPSPGNYWQALAMCASNGAKVASYHSDQQKSAITNICQSSNCAWIGLHSATDGNKYWDDGSQYDYKNLLEVSLCQKKPKSPSCGGGNPPSGDNCPKDCTAVSSSGQTTDQSCESSCGAVICEKDCN*

>Mar|M.Arenaria_Scaff25127g101748

ARNLCNEEGGELVSIHSDEEEQFVAQLALNLLDQCQTNNSLCRQRVPIPSLDSMFRSFYIGLNRAAIEPYYDPTVVQTWADGSSVDYASVPTPGVPTIQIQPWGPGSPSGLNDTGSNPQKGSPEDCVCMYKGADGNINWNDISCYHKLAGGICKLTCNGGNFGKMCGINGWQVANDKEYKQLPISYPGNYWQARKISIK*

>Mar|M.Arenaria_Scaff25773g102522

MYIFPENYIYFRKDYVYFRKIIFSSGTYIYYFNNIFPETIFRKFYFYGRNETSIKEVARWCGQDYPAAIASASDSLLVHFHSDSIIQKRGFNISFVQFDIDTCPPDWISDGISSPYCYKQFVLPHILPWYEAQKECNFERANLATFQNEADYAFIVESYSQTHSFPWVGYSDANVEGIYESIDRNVPLWPENFPLKHENEMKDCVYLDWNKRDQIVVYEIDDCRNRRPFLCKKRRDGTEVPVILPAGMIRRGFRDFTMELYTFGCSCYICFVSISSWMCFISQIQSYNFV*

>Mar|M.Arenaria_Scaff2581g034484

MKLNFVFLISFLIFLFLSYTTEAHFGCNCQYPNNHYVPWYLSGNVENRLTFLERQTNQKINMLIARIEALERELGLIHRISMQEWNNSGAGNIYKIFNTSKTWEEAKNTCMTFGARLASIDSDYKNAFVRSERTLIENKYVNFTIKDMIERSFGKDESAEVWIGLKTRAELTNNPNSHFTNFGEEEKIDGCAVMGIKGKWKIRSCSNLKPFVCEQILM*

>Mar|M.Arenaria_Scaff2628g034872

MHLSSILIILPILFSSILTQQIPNCQTRIFQCDADWDTRTDPNTGDVYGYKAFIARCAEVVSIHSQEEQNFVRRIAAPVLNRCQFTSACAPVVQSSNGTFDRQLRSFWIGLNRVTGTLKDAHSVDSSVYQLWSDRTEVDFGAYPNNSVNSNTNSPPWTRGNPNGVNSADPQIIEDCTQMVESRQGGWNDFPCNWRISGVICKRKCTSYCAAQDV*

>Mar|M.Arenaria_Scaff2637g034970

MMLVILTSVDDEEPVFSQSAQNLFKNENSNEVSGKHPSLEASSPSKNHRQHRKNPPPFVLADVQLLCAEGWERFEGKCYKLVSIEKSWPQALAFCSRFGAKLVRLESSEENKFLAKYLIRPHLTSGPTTSPSEYWIGLLYRPLMMDSSISSSLSHSKSLMDGSFLWSDGSQTSRYVGFWSYGQPDPANGSCTKILVDGLQVEGPTWQLDVCNQLRPFICEQNACIKGSFFCHYGACLPERAHCNGIDECGDSSDEFNCPSAHSEMSCQRYEKGESGRIETPNFPASYRQGANCRWVIEGPLNFKIQLNFDSFETEERHDLVTVLDGGPSENSTFALSTISGTPRNTEKLSFESSTNRMIVQFRADQSIQARGFQASWRTVPISCGNQQLKASSIGQQFHSPEWPRNYPKGLECVWRIEAPSGQLISLFIDEFNTEAETDFLTIYDGLSPSEPILAKFSGHMKEPQLIISTQSQVHIYFFSSETVSQKGFTITYKKGCDNSIRRSHGVLTSPGNAHLPYAPSQICRWSIELPSQQIENFEVAAGIPSLSLVLNSWDVADFGDKLQIYEGGEDGSSNGRPLHESDGFTVNNAPPKTIYAKQGRVELVWRSNVLNSGTGWNISFSTSCPPLSLPSHRVLLSTKNTAYGTHVTISCERGFEFSTGLGRHFETECELGGLWSNNAPVPDCQPVYCSAIPQIANGFAVSATNVSYAGMARYQCYEGFSFASGKQHEEIFCTDEGRWTQSPKCKTDACPALPSFISGERILQFGDGIGFGSVFQFHCAKGYFIEGPLSIVCRPNGEWSSPQPLCKKLTCTDIPVVENGELHLLPSNNVVMDEKSNKDKKQKEQTKSFGSKQKMEFKTKRSMVIRELQFGDSLRVECHSGFQSVGAETLKCLANQTLSGTPKCRDIDECELQSAGNCAAKSTTCVNMPGGFHCQCQPGYKPKLDCSGPMTIIPTKIQTSHGVPIPVQQLNSKNGWCADSAITNAQMLEKTIMPFNESITGSSPSTILTLIFTFPVPKIIEKLHLEKVVVPNASAPSGIAGAIAATPEAWPQRFTLSYSIEEGMPFEVYNGGLAELGGNNNITSNNENKKSNEKKLNTPNTKEIRTRALGAIGSEILVLAKPIEARTLQIEFLEFHGGVPCMKFEFLGCQRTSCEDINECEDGRNGGCEQHCHNTQGGHRCSCEEGFDLFVEPGQSGVRLREGETGYGEFDSLRFNHSCIPRHCAPLTTPENGQLIAQNLYKTFKTEVNNTNNKNEFFSSSFAFPSIVEFRCTFGYQMRGPSHLKCLADGTWNGTVPSCIPATCSGVKNSTAVGLFVQPETVSIPFGQNLSFVCSQTNRPPKHSALGEIRQCIYDPRTDGLEYWLSGPEVDCPLVDCGPPPALSGAYYEGDEGHHGGNFKVGSVYLFQCRAPYSLVGKSSYDDRMVRCNVDGTWDLGDLRCEGPVCVDPGHPDDGQTFLDSVEEGAVASFSCNRPGFKPFPAETISCSLGTPCVLSEDVGISSGFIPDGAFSDNSDKVIWGYEPHKSRMSSSGWCGSKDAFIFLSVDLQRIYTLTTLRLTGVAGNGHLSGHVTKMQLFYKVQFSQNYDNYPMEFSTPSGNHRKIYQFTLNPPLRARYILLGITEYEKNPCLRFDMHGCLAPLSTTHEVPAHLQVIHSLSLPSNLSLSFQVGWNASIPQCLDAEPPTFKNCPQSPIIVQTDENGQLFPANYVIPEATDNSGRITYMLTKPEDFHPPYPVSQDTDIIYQAFDDAGNMAECPVRLRIPGKTWIPPILKCPDSYAIWAQENQTELHINFNESSVRLVVQDQSPITQISYDPPEARIKLDSHVTVEASVLDAHSNRNKCKFQVALLPEPCSPWSLRIDEATVQKQCQRHASGTVCQVQCRKGYRFLESFTQQKSEVKKQQNSTNTLPQRYSCSMEQQSGKWLPSPTPPACVPMAMEPARYEMRVHMNYNLTSPLPSDCAKSYELLVGSLFDSIDQVLSQRCSSTVQIYVRFLDAKFSQMGEKTMSANFTVQILPTVLQQVFYELCSLTLRTIFDLRIPGATIPIRSLLTLSGDSVPAILNLGCPPINASSISVSQGFSCSQGELLKLQTPNVGSDDFLSAAPGLPECFPCPKGTAFVNNSCIQCPMGSFQDQEGQIRCKPCIENTYTLQSGAQSNESCLDVCGNGMFSATGMIPCQLCPRHTFAGPPPIGGYKECEPCPEGTYTARLGSVGPSHCKQPCAPGHFSVTGLEPCSPCPINFYQPNIGQQRCLQCSNDSFTAETGRSADEHCKKLDCQTLKCQNRGQCVVANHKEVCECRPGFMGSHCEQQIPLCDSHPCLNGGTCELHNGAFRCICPQNYTGSRCQFGPDECISSVHCPNGGVCQDLPGLGTTKCICRTGFTGPDCSQISDPCQSDQPCKNGAQCIPLQLGRYKCKCLPGWEGTNCDKNIDDCTENPCALGAKCHDLINDFECECPHGFSGKRCQIKDNLCTPSPCLNGGQCVDTLFDRHCICKRGWNGTFCEQEVNECSQKPCQNGATCRDQEDDYSCECAPGFHGYQCQYMIDHCAVKPCRNNGTCINRGPIYECQCPLGYEGDHCEHNVDECKMMTPCDAVGTGRCEDMINGFKCHCHPGYEGTFCEQHVSQCEDEPCMNNGTCTDLGAGFQCECQLGWKGDRCQEMETQCDRKPCMNEGKCVPLVDDYFCVCPEGVSGKNCELAPNRCLGEPCHNGGVCGDFGSRSDCQCPKGYSGNGCQFRFDGCHEGLCKNGGTCVNNNEHQMSKIENTVAVEQNSEATGFKCICAPGFSGNECEIDINECQPSPCPLASHCVDLVNGYYCKCPFNTSHCVDLVNGYYCKCPFNMTGANCEKRIDPDYDLRFLESSSQPASASLGIPFNFVSSALTLNIWVKFEKNHQTDVLKEQRKSPPIFFTLYGSSSANQPSNLTELLTISSEAINIRLFPELDKQPLVLHFPLHQRPDTQLAWNNIIFMWDSQQQGSYSLLWNAVRLYSDKGYAPDRRLDINAWINLGDPKGIEQQQNQHLMTNKIISTDLPKGNEKIIGTKFVGSITRVNMWNRMLDFETEIPSIVQRCQGSPDLYEGLSLRFANYDMLQGKVERIAKSTCGRIDLLSPTCHSSGDENKNLKDHQCRSKILSTMNDYFKEELIEVEGCPLEPINVQTPLKELNISWKEPRFYETSSRVQIAKIEQNLKPGQVFTWGQYSAIYLALDNQSSPLATCQFKINVVREFCPDPEIPVNGVQRCEQWGPGLRYKACSVHCQNGYGFSRPPAAFYSCGEDGKWRPNEGKQRPFRYPQCTRQSPAEHLARIQLSYPQLALCNPAGKSTVTEKIIERINQINNKWHICANNEESSDCSGVQVQINCQDIFAASRLKRQTTLQQFDVIIDVPIREIQKMDPVEIIRDEAMSHGLFSLEQVIPNGRPDIGAFRVENAFRCPIGYLLNNGSCVPCAPGTFYFVPTSECKLCPIGQYQPEEAQNQCIECPIDSPMTVGMGSIKQNECRIRCLPGHHLNISTGQCEPCSYGFFQPDSGAFDCIPCGIGKTTLERTAINEDQCRDECPDGQQLTASGSCQPCPQGMYRTRGQDKQCVECPSGTTTEGVGAGNKALCNTPKCGAGQFLLTDIKRCQFCPRGTFQDQQLQFECKKCPPSFNTAEEGATRESQCYSTDQCALGQDNCSWNAQCIDLPDDNDVASWRCVCNPGFRGNGINCTDACLNFCLNDGICRKNKLGHVECSCKENFSGERCEIRFQPRSQKLVYWTGAIVAVVFLLIVIVVVIWMISLRFSRSSDNSFLSSPLDKPALSFTQSTTDSPLASNFLYGRPPPILDRSHSSSLGGGGSNSIIHPIGFYYEDEQSPYDGGVVGAVGRHGRENSQEMKSIFLASTVPDNNNEVVEGSSSASHSPCQQINGGIKVTGTTNTTNSSGGSPGSATRALEQRLRHIQQHMYRPIGDG*

>Mar|M.Arenaria_Scaff2835g036573

MKKRESYINEEENNGKTKLSSNSIEKRLDRLEANQQNLRRATLADWPAFSVDKRVRIFDHTRSTWIDAQRECSEHAGTLLEIDSESENERINEMLTNSGNSNRPNDNYWIGVQIMLQFSNGSSIIGNYSNFEEQNSNSENTENQKLSKGLVMKRCAAISASQTTKSEGRWLSLECSEKHGFICQL*

>Mar|M.Arenaria_Scaff2868g036863

MSIKIKYFLNKIFFFLFFNFVKNSKYNNQKYLVSERENNFCENTWEYWPERHLCIKIFNEKIGWNVAEINCLYNQGHQISVKNIKENKYLEDIARRVGGLIWLGAAQFGNNYMWTDGTPFYFTFWQNGIQPLFNPGKKCIKMNSLSGEWIQSCCRVAAPFVCQKPAFKINKKHLLRNEERKEEFIQR*

>Mar|M.Arenaria_Scaff2906g037202

MITLTYVSLFYLSLPALIAGQCSSDWQTNVGSDGFTYGYKAIVSDMVNFFEAHDVCAALGAQVASIHSAQENAFMISIASSLLDQCENNNAVCKQRVDRSTTSSLRLDRLLRSFWIGMHRVQYAPFYNTSVDCCWSDGTQCDYGRFDGVADPNQNIPPWSTGNPSGSSSSVTSAGWNVEDCVEITNDNGVLWNDIGCYHKLGGVVCKKNCTNTCPPVTTQAPAITSKPITTIPVVTTTTDDDTGGYICGSDDWQWKNNGNGKAYAYKWFNQSGSFWQLHNLCYDSCATPCSIESEEENQHIVNNICAGAFSSSSSGRKKRATDSECIYTGFHRYQESPPNGPIGCSCDDGKSCDYGNDDTTTTTSKTSTTKAATTTTKAITTPRPSTSTIRATTKATTIITSTTKPNNPWAPGCPANSTTGLGDSDGYGTKNCVGFDSNGKWRDQACERPGKGIVCKKPCIPPTGS*

>Mar|M.Arenaria_Scaff307g007449

MKNIHTGHKRPRNRKIKLYISYLNLLINIFVITTLFVNINGTESDEHLLGLKDNDASYSSSTSVDDEEPVFSQSAQHLFKNENSNEDAEKHPSLEASSPSKNHRQHRKNPPPFVLADVQLSCAEGWERFEGKCYKLVSIEKSWPQALAFCSRFGAKLVRLESSEENKFLAKYLMRPHLTSGPTTSPSEYWIGLLYRPLMMDSSISSSSSHSKSLMDGSFLWSDGSQTSRYVGFWSYGQPDPANGSCTKILVDGLQVEGPTWQLDVCNQLRPFTCEQNACIKGSFFCQNGVCLPERAHCNGIDECGDSSDEFNCPSAHSEMSCQRYEKGESGRIETPNFPASYRQGTNCRWVIEGPLNSKIQLNFDSFETEERHDLVTVLDGGPSENSTFALSTISGTPRNTEKLSFESSTNRMIVQFRADQSIQARGFQASWRTVPISCGNQQLKASSIGQQFHSPEWPRNYPKGLECVWRIEAPSGQLISLFIDEFNTEAETDFLTIYDGPSPSEPILAKFSGHMKEPQLIISTQSQVHIYFFSSETVSQKGFTITYKKGCDNSIRRSHGVLTSPGNAHLPYAPSQICRWSIELPSQQIENFEAAAEIPSLFLVLNSWDVADLGDKLQIYEGGEDGSSNGRPLHESDGFTVNNAPPKTIYAKQGRVELVWRSNVLNSGTGWNISFSTSCPPLSLPSRRVLLSTKNTAYGTHVTISCERGFEFSTGLGRHFETECELGGLWSNYAPVPDCQPVYCSAIPQIANGFAVSATNVSYAGMARYQCYEGFSFASGKQHEEIFCTDEGRWTQSPKCKTDACPALPSFVSGERILQFGDGIGFGSVFQFHCAKGYFIEGPLSIVCRPNGEWSSPQPLCKKLTCTDIPVVENGELHLLPSNNMVMDEKSNKDKKLKDQTKNFGSKQKMEFKTKRNMVVRELQFGDSLRVECHSGFQSVGAETLKCLANQTLSGIPKCRDIDECELQSAGNCAAKSTTCVNMPGGFHCQCQSGYKPKLDCSGPLTIIPTKILTSHGVPIPVQTLNSKNGWCADSAITNAQMLEKRIMPFNESITSSFPSTILTLIFTFPVPKIIEKLHLEKVVVPNAVAPSGIAGAIAATPEAWPQRFTLSYSIEEGMPFEVYNGGLAELGGNNNITSTNENKKSNEKKLNIPNTKEIRTRALGAIGSEILVLAKPIEARTLQIEFLEFHGGVPCMKFEFLGCQRTSCEDINECEDGRNGGCEQHCHNTQGGHRCSCEEGFDLFVEPGQSGVRLREGETGYGEFDSLRFNHSCIPRHCAPLMTPENGQLIAQNLYKNFKTEANNTNNKSEFFSSSFAFPSIVEFRCTFGYQMRGPSHLKCLADGTWNGTVPSCIPASCSGVKNSTAVGLFVQPETVSIPFGQNLSFVCSQTNRPPKHSALGEIRQCIYDPRTDGLEYWLSGPEVDCPLVDCGPPPALSGAYYEGDEGHHGGNFKVGSVYLFQCRAPYSLVGKSSYDDRMVRCNVDGTWDLGDLRCEGPVCVDPGHPDDGQTFLDSVEEGAVASFSCNRPGFKPFPAETISCSLGTPCVLSEDVGISSGFIPDGAFSDNSDKVIWGYEPHKSRMSSSGWCGSKDAFIFLSVDLQRIYTLTTLRLTGVAGNGHLSGHVTKMQLFYKVQFSQNYDNYPMEFSTPSGNHRKIYQFTLNPPLRARYILLGITEYEKNPCLRFDMHGCLAPLSTTHEVPAHLQVGWNASIPQCLDAEPPTFKNCPQSPIIVQTDENGQLFPANYVIPEATDNSGRITYMLTKPEDFHPPYPVSQDTDIIYQAFDDAGNMAECAVRLRIPDTVPPILKCPDSYAIWAQENQTELHMHFNESLVRLVVQDQSPITQISYDPPEARIKLDSHVTVEASVLDAHSNRNKCKFQVALLPEPCSPWSLRIDEATVQKQCQRHASGTVCQVQCRKGYRFLESFPQQKSEVKKQQNSTNTLPQRYSCSMEHQSGKWLPSPTPPACVPMAMEPARYEMRVHMNYSLTSPLPSDCAKSYELLVGSLFDSIDQVLSQRCSSTVQIYVRFLDAKFSQMGEKTVSFFKKFFYKHLLQMSANFTVQILPTVLQQVFYELCSLTLRTIFDLRIPGATIPIRSLLTLSGDSVPAILNLGCPPINASSISVSQGFSCSQGELLKLQTPNVGSDDFLSAAPGLPECFPCPKGTAFVNNSCIQCPMGSFQDQEGQIRCKPCIENTYTLQSGAQSNESCLDVCGNGMFSATGMIPCQLCPRHTFAGPPPIGGYKECEPCPEGTYTARLGSVGPSHCKQPCAPGHFSVTGLEPCSPCPINFYQPNIGQQRCLQCSNDSFTAETGRSADEHCKKLDCQTLKCQNRGQCVVANHKEVCECRPGFMGSHCEQQIPLCDSHPCLNGGTCELHNGAFRCICPQNYTGSRCQFGPDECISSVHCPNGGVCQDLPGLGTTKCICRTGFTGPDCSQISDPCQSDQPCKNGAQCIPLQLGRYKCKCLPGWEGTNCDKNIDDCTENPCALGAKCHDLINDFECECPHGFSGKRCQIKDNLCDPSPCLNGGQCVDTLFDRHCICKRGWNGTFCEQEVNECSQKPCQNGATCRDQEDDYSCECAPGFHGYQCQYMIDHCAVKPCRNNGTCINRGPIYDCQCPLGYEGDHCEHNVDECEMMTPCDAVGTGRCEDLVNGFKCHCHPGYEGTFCEQHVSQCEDEPCMNNGTCTDLGAGFQCECQLGWKGDRCQEMETQCDRKPCMNEGKCVPLVDDYFCVCPEGVSGKNCELAPNRCLGEPCHNGGVCGDFGSRSDCQCPKGYSGNGCQFRFDGCHEGLCKNGGTCVNNNDLSKHQMSKIENIAVEQNSEANGFKCICAPGFYGNECEIDINECQPSPCPLASHCVDLVNGYYCKCPFNMTGANCEKRIDPDYDLRFLESSSQPASASLGIPFNFVSSALTLNIWVKFEKNHQTDVLKEQRKSPPIFFTLYSSSSANQPTNLTELLTISSEAINIRLFPELDKQPLVLHFPIHQRPDTQLAWNNIIFMWDSQQQGSYSLLWNAVRLYSDKGYAPDRRLDINAWINLGDPKGIEQQQNQHLMTNKIISTDLPKGNEKIIGTKFVGSITRVNMWNRVLDFETEIPSIVQRCQGSPDLYEGLSLRFANYDRLQGKVERIAKSTCGRIDLLSPTCHSSGDENKNLKDHQCRSKIPSNMNDYFKEELVEVEGCPMEPINVQTPLKELNISWKEPKFYETSSRVQIAKIEQNLKPGQVFTWGQYSAIYLALDNQSSPLATCQFKINVVREFCPDPEIPVNGVQRCEQWGPGLRYKACSVHCQNGYGFSRPPAAFYSCVEDGKWRPNEGKQRPFRYPQCTRQSPAEHLARIQLSYPQLALCNPAGKSTVTEKIIERINQINNKWHICANNEESSDCSGVQVQINCQDIFALSRLKRQTTLQQFDVIIDVPIREVQRMDPVEIIRDEAMAHGLFSLEQVIPNGRPDIGAFRVENAFRCPIGYLLNNGSCVPCAPGTFYFVPTSECKLCPIGQYQPEEAQNQCIECPTDFPMTVGMGSIKQNECRKEAQNQCIECPTDFPMTVGMGSIKQNECRIRCLPGHHLNISTGQCEPCSYGFFQPDSGAFDCIPCGIGKTTLERTAINEDQCRDECPDGQQLTASGSCQPCPQGMYRTRGQDKQCVECPSGTTTEGVGAGNKALCNTPKCGAGQFLLADIKRCQFCPRGTFQDQQLQFVCKKCPPSFNTAEEGATRESQCYSTDQCALGQDNCSWNAQCIDLPDDNDVASWRCVCNPGFRGNGINCTDACLNFCLNDGICRKNKLGHVECSCKENFSGERCEIRFQPRSQKLVYWTGAIVAVVFLLIVIVVVIWMISLRFSRSSNNSFLSSPLDKPALSFTQSTTDSPLASNFLYGRPPPILDRSHSSSLGGGGSNSIIHPIGFYYEDEQSPYDGGVVGAVGRHGRENSQEMKSIFLASTVPDNNNEVVGGSSSASHSPGQQNNGGIKVTGTTNTTNSSGGSPGSATRALEQRLRHIQQHMYRPMGDG*

>Mar|M.Arenaria_Scaff3125g039029

MPLILFSLIFGLIPTLVSADCSVGNWTTRVDSDGNTYGYQVLMRDWLNFYEARALCLGVGGDVVSVHSNAENEFVRQLAAPYIAACQTNKTVCGSRATTSLDFYLSVVWMGMTRCQYFPSYNATVDCVYSDGTTCNYATSASVYPWGVGSPSGSDSGGGAGLIEDCVSMYNGTSGEWNDVSCFQKLGGVVCKRNCTGTCGSTSIKTALLTSPSDITDTSCTSGNWTKRAGEDGNTYGYQVVMKDWLNFYEANAQCLALGAEVVSIHTVAENEFIRQLAGPYITACQTNTSVCVSRVSTTQDIQWRSLWLGLHRCAFYPTYNATVDCINSDGTVCDYLNITGGPSGTETGTASGQQEACAAMYSATTGQWNDIACFNKLGGVICKKNCSKACGVASTTTTQLTTTTTQPSTTTTAEPTTTTTLPTTTTTLPTTTTTLPTTTTTEPSTTTTQPTTTTTEPSTTTTAEPTTTTTEPSTTTSFNCAQPPVTSLLSYNTSDPNIQTGPNATEGVCECPADPGNNNVFFIPVTTITTGSSASNSSVIMKCSKMQDFCICDEDDICWKVINAYSLVVINSFCDPTCHNYARLQNAAPFNQTFESDCGRTITLADELTPIPNTNRNTFKPLGSSADYYIKAASIRCLQAGQTCTPIKCSGTRNAFPLSYDHNYNANNYYYPTTTTTTCQLPRQPYQLPRQHCQLPQQLTL*

>Mar|M.Arenaria_Scaff314g007604

MKKRESYINEEENNGKTKLSSNSIEKRLDRLEANQQNLRRATLADWPAFSVDKRVRIFDHTRSTWIDAQRECSEHAGTLLEIDSESENERINEMLTNSGNSNRPNDHYWIGVQIMLQFSNGSSIIGNYSNFEEQNNSENTQNQKPSKGLVMKRCAAISASQTTKSEGRWLSLECSEKHGFICQL*

>Mar|M.Arenaria_Scaff318g007688

MLGTSIRCVVASLLVLQAYANKMDPAYQTDDDRNQPVESVPSEQAPWISGPQNQKYQFHIGKQSWLSAREKCLAQNADLVSIESIEEMQWLLSHYKPQFNHLRERQVQIGLLLDTIEGEAGSNSDSGLTSREWRWVNGKPLNLEITKWTMGEPFDHAKGKERCALLNINERRLDDVDCDLGSGAGFNYRFVCQRSHEKHIEHESLNNPLWKKLEDILTFFGISDREEDKKNCSLPIGAKEEGYWERAFNKGEGNSETQHKEKTTIEKTDSITEKSELEITKGVEKNSKPNKEDRETVLEDKTIIEKKVITNKNEEKTNKLSSPSEQKLVKDVEKVVEEKKVSKVGGVTQHATSKIIGGEDEQPPNELENTRPTGGAVVLRRVESHVSASSSVDNKKNNNQEKLKEGTEEAKSTKLEEEQSKIAGTEEANIREKEASDSSDSSRGQSGLSVREKSQIINLNSRENIQSSLNPKKPLIISNDPSDLKEDRNTISNKNTSDNEKENKNTHLSKSVQITEKKLGEDEISEIKQYTLENKYIVNTPKVDKKNDEKIKEDKLNAEKINEEKISEKEEKKEDKIRNGNKDDKKGENEEITIVDERKSKKEDKEIEKIEENNKEDREVKRDEGRKEENEKKSDELKKNEVTREKNIKEEKLKSEETKTGKEKHDEKEEKEDRKNLLPKSNNIRDDSKKLDLENIKEEKLEANLIKNKINNEIRDVKKNEEVLAQNKVEEKIREEQSLELKDKDKKDDDKSGKKSENAEEKTVEIKEAKEDSNQELMKANNKEKDGLNNETTHDVKNEATNSKINELDRETKKEVKMEENREAKDEAKLEAKHKMKIEATEANEKESVNDAKSEAQSETKGEARHEANKEISSNKGTNNEAKSKENNEAETKISEDKIVKTNKPTKHPDPLSVVAETLERSLSSKEETIEKKKDEAENLIKEKRQKPDGVRDRIQHLEKIITEVQHMMGIGEKGGEGELIEKTNKVLEEDEEEKKYEIKKEEGRKNIKEVKKEKEEKIRVEEKEESKEGSGSSEKLTEATPIAEVEELSSPEVEPKSHSPSVSSSDESEESEYSPKHIKTKNIKDNKAQREQPVNVNNEAINKSGAIKEFGTVNKSHSIISDSLDEQRKSMAAMEFDFEEAALQAKAHPGINSGDLVKNIILIGKDEELDGCREGKECKDKKAKSKQNRRYAPRNEEKWEIINKMTDDINAKKGSRIEKYGRKSIGNKKRTEGDLGEEKEVEDLNLIKNNHDQGLLNSIHSETIEATPTGHILDQLIIFHPNDGKRSRESQIISQMLLPDGGFHGKVTGDGKIEHRPADDDWVLPEVETQTMTTDRINSSSNSSQSSTPLSIFPAPDFSIIKPGDQVPPQKIIQRLEEERKRIDAVYEKLNKGDNIYDLIKRIKASDARRQPLTVLQSKMTPN*

>Mar|M.Arenaria_Scaff3282g040286

MGAEIVSIHSAEENSFVAKLAAPLLAECQHNPLICAERVPHIDKAHELLDHILSGFWIGLHRSQFYPFYNPAVVQVWSDRSECDYGCYEEEVDCNMKIEPWGNCDPSGTNSSHGHGQSEDCTQIYLATSSGIAKWNDLACNQKIGGFICKKPCDGERKHDVCGKDGWPHVDGKAFHAFPLHSPGNYWQALSICNKHDAQVASIHSNEENKITSALAIGQAGGNISACSWIGLHSANGGSKERFWDDGSNVDFGKHVEPGNLPVSDDISSDDKGKESHHCRECMAINGNGHWKDLNCGANCGAVICKKECRNPE*

>Mar|M.Arenaria_Scaff3501g041912

MLGTSIRCLVASLLVLQAYANKMDPAYQTDDDRNQPVESVPSEQAPWISGPQNQKYQFHIGKQSWLSAREKCLAQNADLVSIESIEEMQWLLSHYKPQFNHLRERQVQIGLLLDTIEGEAGSNSDSGLTSREWRWVNGKPLNLEITKWTMGEPFDHAKGKERCALLNINERRLDDVDCDLGSGAGFNYRFVCQRSHEKHIEHESLNNPLWKKLEDILTFFGISDREDDKKNCSLPIGAKEEGYWERAFNKGEGNSETQHKEKTIEKTDSITEKSELETPKGVEKNSKTNKEDRDSVLEDKTIIEKTCRYWFXLNINERRLDDVDCDLGSGAGFNYRLCANGVMKNILRETAKLNIKKTTIEKTDSITEKSELETPKGVEKNSKTNKEDRDSVLEDKTIIEKKVITNKNEETTNKLSSPPEQKLVKDVEKVVEEKKVSKVEGVTQHATSKIIGGEDEQPPNELENTRPTGGAVVLRRVESHVSASSSVDNKKNNQEKLKEGNDEAKSTKLEEEQSKILKVAREDEANLREKEASDSSDSNREQSGLSVREKSQIINLNSRENIQSSLNPKKPLIISNDASDLTEDRNTISNKNSSDNEKNNKNIPNLSKSVQITEKKFGENEISNIKQYTLENKYIVNTPKVDKKNDEKIKEDKLNVEKISEEKTSENEEKKEEKNEDKIKNGDKNDRKGENEEITIVDERKTKKEDKEIEKIGKNNKEDREVKRDEERKEENEKKSDGLKKNEVTREKDIKEEKKVIIEKDIKEEKLKSEETKTRKEKHDEKEEKDDRKNLLPKSNNIRDESKKLDLENKEEKLEANLIKNKINNEIRDVKKNEEILAQNKVEEKIREEQSLELKDKDKKDDNKSGKKSENTEEKTIEIKVAKEDSNQESMKANNEAKDGLNNETTRDVKNEETNSKNNEVNRETKKEAKLKENKEAKDEARHEAKHKTKIEAIEANEKESVNEAKSEAQSETRHEANKASSSNKGTKNEAKSKENNEAETKISEDKIVRTNKLTKHPDPLSVVAETLERSLSSKEETSEKKKDEVENLIKEKRQKPDGVRDRIQHLEKIITEVQHMMGIGEKGGEGELIEKTNKVLEEEEEEKKYEIKKEEGRKNMKEVKKEKEEKIRVEEKEESKEGSGSSEKAFLSSSSNSSSSIISSPSSTNPITTQPHNPVPQQIHGERNWRRRHFSSPLVHNSHSAVSRGGGDMVQTLMENAPELRIFDRAANLYGDLFRMMLDPEMLREQSSIPEGKHEEYD*

>Mar|M.Arenaria_Scaff3557g042312

MNIRPPNPPTSTPNTSCPPDYVFFKEGNCCIQISSPEKKPLTWKEASEEAKKNGARMASIHFKKEDDFIRDLVAKQPKGEGKDNPVYFTGLNVNATNGKINNFAWSDNTRVNYGSLKDIKPNTDPWGPKNTFNGNTGFGGISKDPSGKASWGLYDPNQKGGYVWKIDLSKKDGGTKVPEPGNPTGGGAPGGNNGLPAGYKCTTGCNPSSRINFV*

>Mar|M.Arenaria_Scaff3761g043744

MLHFITILLFIFHLAIAQNGDNLNDYDEDIITTTANYNAGLNADNANNAKPSTASTTTGDNIMVLENLNNATGNPADQADDSSDKPLYPNANPNENGGANQCPDGWATRQDKDGNVYGYKVIMQDMVNYYQTTANYNAGLNADNANNAKPSTASTTTGDNIMVLENLNNATGNPADQADDSSDKPLYPNANPNENGGANQCPDGWATRQDKDGNVYGYKVIMQDMVNYYQARLLCIKEGGELVSIHSDEEEQFVVQLATPLLDKCQTDTSVCKQRVQIPSLDKMYRSLYIGLNRAAIEPYYEPTVVQAWSDGTPVDYASVPTPGVPTTLIPPWGAGCPSGLNDTGNTPQKGSPEDCVCMFKYDSVNWNDISCYHKLGGGICKRKCNGGGGGGGQPCGCGKVNCGTDGWSQSANGKGYKIFQLPSPGNYWQALAMCANNGAKVASYHSDQQKSVITNICQSSNCAWIGLHSATDGNKYWDDGSQYDYKNLLEVSLCQKKPKSPSCGGGNPPSGDNCPKDCTAVSSSGQTTDQSCESSCGAVICEKDCN*

>Mar|M.Arenaria_Scaff3816g044124

MYFYFNKIILLFLFSIFVKCIPKYQEEFGDDILKTLNKETIENGEIEVKTGEDKLGDNKIRKWRKYVIKRKSNRGRNEEEKINEGERSLLDAPQINEKSKGNLDNNEKNKIKNVLDWNKQRAPVICGDEEWHYFDGFCYKLINGKLTWEEALGECKKQNSNLVSIQSEQENDFVGSLSTKFDQNFDNFCHGFWIGMTRKYIEETDSFKSEWSDGTIVNYGNVPIKVAFHTPPWMGGQPDFAGGVEECVHSYPKLGCDNWQQIFFNQWNDARCSIKLSGAVCKKRQSIQKI*

>Mar|M.Arenaria_Scaff3854g044370

MLHQQQQQQQQLKPKFRLQLEDVGNPQPCPDNPIRLLASRTPQYIYSPYDENKLYPPDTDCQFLIEATDKFHRIHLTIIESDLEEALFTDCNDYVSVRDREGNETSIKEVARWCGQDYPAAIASASDSLLVHFHSDSIIQKRGFNISFVQFGILESSKSPQNLKIIFLDIDTCPPDWISDGISSPYCYKQFVLPHILPWYEAQKECNFERANLATFQNEADYAFIVESYSQTHSFPWVGYSDANVEGIYESIDRNVPLWPENFPLKHENEMKDCVYLDWNKRDQIVVYEIDDCRNRRPFLCKKRRDGTEVPVILPAGMIRRGFRDFTMDYTLLVVVVIFALLALVVGCVLFHKYKERNNRIINIDMNQRLVQQQQGQQPGNKDKAAAALARQKAKERERRELREQKYAETSNNNSNRGGRGGGGIAATIKSPFESQDSNASFPLQTFQSTFAQRMTTTTTGSVEPLDTAAALAEASATASQQMREHEATKLHPQQLSAEIVSVDSPPEDDLNSSPRIRMSPNQPSFVEEGRKISEKKIRKGWMGAGVSGGGTTDREEEEEEKDRHNMGYEPDSEEEEHEENERQKIRRASKINGEGEGGEENFLMELNEEGGRKNNGENTGENTGENTGENEELEQYTQHNHMEEELEQYNGGEQFKQYKHKNVIESEQFKNSQQFKQLESDGGEVQQKVVQVELHEEIGEEGGLSQFKDKRTKEERITNFDEKMMDSYAIGDHFKAAKVATSAIASAAIAATEHPIRPPIELMRTTTRTTSTDKTKSPPPKEAKESEKGESGKSSREATREGKEETREEKETFPEKVSGKGNIPSSTATNIVKTEGSGTRIRIKRKTFDRPPPVGPLDNVSAISLDEFWQQQK*

>Mar|M.Arenaria_Scaff3976g045190

MGMSPILFSLIFGLIPTLVSADCSVGNWTTRVDSDGNTYGYQVLMRDWLNFYEARALCLGVGGDVVSVHSNAENEFVRQLAAPYIAACQTNKTVCGSRATTSLDFYLSVVWIGMTRCQYFPSYNSTVDCVYSDGTTCNYATSSSVYPWSVGSPSGSDSGGGAGLIEDCVSMYNGTSGEWNDVSCFQKLGGVVCKRNCTGTCGSTSIKTALLTSPSDITDTSCSSGNWTKRAGEDGNTYGYQVVMKDWLNFYEVEKYSRSMIKRAEVVSIHTVAENEFIRQLAAPYITACQTNTSVCVTQWRSLWLGLHRCAFYPTYNATVDCINSDGTVCDYLNITGGPSGTETGTASGQQEACAAMYSATTGQWNDIACFNKLGGVICKKNCSKACGVASTTTTRLTTTTTQLSTTTTAEPTTTTTLTTTTTTLPTTTTTLPTTTTTEPSTTTTLPTTTTTEPSTTTTAEPTTTTTEPSTTTSFNCAQPPVTSLLSYNTSDPNIQTGPNATGGVCECPADPGNNNVFFIPVTTITTGSSASNSSVIMKCSKMQDFCICDEDDICWKVINAYSLVVINSFCDPTCHMYARLQNAAPFNQTFESDCGRTITLADELTPIPNTNRNTFKPLGSSADYYIKAASIRCLQAGQTCTPIKCSGTRKPSPCPTTTTTMPSTTTTLPTTTTTLPTTTTTLPTTTTTLPTTTTTLPTTTTTLPTTTTTLPTTTTTLPTTTTTLPTTTTTLPTTTTTLPTTTTTLPTTTTTLPTTTTTYQLPRQHLPTTTTTLPTTTTTLQTTTTTLPTTTTTLPTTTTTLPTTTTTLPTTTTTLPTTTTTSPTTTTTLPTTTTTLPTTTTTLPTTTTTLPRTTTTLPSTTTVLPTTTTPPINCADCSAGQSKVIYEKNTLLSNQTINNLAATECIYNCKDKDVSQVFFHSCCVVGFVTD*

>Mar|M.Arenaria_Scaff4203g046670

MLHFITILLFIFHLAIAQNGDNLNDYEDIIATTASYNNLELNADNANAKPSTASTTTGDNIMVLENLNNATGNPADQADDSSDKPLYPNSNPNENGGANQCPDGWATRQDKDGNVYGYKVIMQDLVNYYQARLLCIKEGGELVSIHSEEEEQFVVQLATPLLDKCQTDTSVCKQRVPIPSLDKMYRSLYIGLNRAAIEPYYEPTVVQAWSDGTPVDYASVPTPGVPTTIIPPWGAGCPSGLNDTGNTPQSFFGIYPLKIHKIFKEGSPEDCVCMFKYDEVNWNDISCYHKLAWSDGTPVDYASVPTPGVPTTIIPPWGAGCPSGLNDTGNTPQKGSPEDCVCMFKYDEVNWNDISCYHKLGGGICKRKCNGGGGGGGGQPCGCGKVNCGTDGWSQSANGKGYKIFQLPSPGNYWQALAMCANNGAKVASYHSDQQKSAITNICQSSNCAWIGLHSATDGNKYWDDGSSYDYKNLLEVSLCQKKPKSPSCGGGNPPSGDNCPKDCTAVSSSGQTTDQSCESLCGAVICEKDYLTEIQTPKPQGHKIIKTKIYTNDSSFERARRAESNETKINQIRLDLTKL*

>Mar|M.Arenaria_Scaff435g009786

MLHFITILLFIFHLAIAQNGDNLNDYDEDIIMTTANYNAGLDADNANNAKPSTASTTTGDNIMVLENLNNATGNPADQTDDSSDKPLYPNANPNENGGANQCPDGWATRQDKDGNVYGYKVIMQDLVNYYQARLLCIKEGGELVSIHSDEEEQFVVQLATPLLDKCQTDTSVCKQRVPIPSLDKMYRSLYIGLNRAAIEPYYEPTVVQAWSDGTPVDYASVPTPGVPTTLIPPWGAGCPSGLNDTGNTPQKGSPEDCVCMFKYDSVNWNDISCYHKLGGGICKRKCNGGGGGGGQPCGCGKVNCGTDGWSQSANGKGYKIFQLPSPGNYWQALAMCANNGAKVASYHSDQQKSAITNICQSSNCAWIGLHSATDGNKYWDDGSHYDYKNLLEVSLCQKKPKSPSCGGGNPPSGDNCPKDCTAVSSSGQTTDQSCESSCGAVICEKDCN*

>Mar|M.Arenaria_Scaff4940g051102

MEIAYFLVCLSILLPYNFLIAQKVPNCPARSYNCDTDWQLFTDPDSGVEYGYKAFIQDSINFYEADIFCRARCAEVVSIHSQQEQDFVTKIAGPLLTKCQFTDACSERMEHTNKSAANFDRMLRGFWIGMNRVYGTLLNHNTIDTNVTCIWSDRSKCDYGSVDNGSVTPDTVTPPWARGNPNGANPGGDPLIVQACVQMVEGRHGGWNDISCTYRLGGLICKRKCSSYCAAQDA*

>Mar|M.Arenaria_Scaff4940g051104

MHLSSILIILPILFSSILTQQIPNCQTRIFQCDADWDTRTDPNTGDVYGYKAFIVNKMNFYEADVFCRARCAEVVSIHSQEEQNFVRRIAAPVLNRCQFTSACAPVVQSTNATFDRQLRSFWIGLNRVTGTLKDAHSVDSSVYQLWSDRTEVDFGSFPNNSVNTGTNSPPWTRGNPNGVNSAYFIIRKRHVLFSLLLISTGRHVIPPAYFTVRTADPQIIEDCTQMVESRQGGWNDFPCNWRISGVICKRKCTSYCATQDV*

>Mar|M.Arenaria_Scaff4981g051339

MLGTSIRCVVASLLVLQAYANKMDPAYQTDDDRNQPVESVPSEQAPWISGPQNQKYQFHIGKQSWLSAREKCLAQNADLVSIESIEEMQWLLSHYKPQFNHLRERQVQIGLLLDTIEGEAGSNSDSGLTSREWRWVNGKPLNLEITKWTMGEPFDHAKGKERCALLNINERRLDDVDCDLGSGAGFNYRFVCQRSHEKHIEHESLNNPLWKKLEDILTFFGISDREEDKKNCSLPIGAKEEGYWERAFNKGEGNSETQHKEKNIEKADSITEKSELETPKGIEKISKTNKEDRESVLEDKTIIEKKVITNKNEETTNKLSSPPEQKLVKDVEKVVEEKKVSKVGGVTQHATSKIIGGEDEQPPNELENTRPTGGAVVLRRVESHVSASSSVDNKKNNQEKLKEGNEEAKSTKLEEEQSKILKVAGTEEANLREKEASDSSDSNHGQSGLSVREKSQIINLNSRENIQSSLNPKKPLIISNDPSDLKEDRNTVSNKNTSDNEKENKNIQNLSKSVQITEKKLGEDEISEIKQYTLENKYIVNTPKVDKKNDEKIKEDKLNAEKISEEKISEKEEKKEDKIRNGSDKDEKKGGNEEITIVDERKTRKEDKESERIEENNKEDREVKRDEGRKEENENKSDELKKNEATREKDIKEEKKVINDNDIKEEKLKSEETKIRKEKHDEKEEKEDRKNLLPKSNNIRDESKKLDLENIKEEKLEANLIKNKINNEIRDEKKNEEVLAQNKVEEKIREEQSLELKNKDKKDDDKSGKKSENAEEKTVEIKEAKEDSNQESMKANNKEKDGLNNETTHDVKNEATNSKINELDRETKKEVKMEENREAKDEAKLEAKHKMKIEATEANEKESVNDAKSEAQSETKGEARHEAETKISEDKIIKTNKPTKHPDPLSVVAETLERSLSSKEETIEKKKDEAENLIKEKRQKPDGVRDRIQHLEKIITEVQHMMGIGEKGGEGELIEKTNKVLEEDEEEKKYEIKKEEGRKNIKEVKKEKEEKIRVEEKEESKEGSGSSEKLTEATPIAEVEELSSPEVEPKSHSPSVSSSDESEESEYSPKHIKTKNIKDNKAQREQPVKVNNEATRKSGTVKEFGTVNKSHSIISDSLDEQRKSMAAMEFDFEEAALQAKAHPGINSGDLVKNIILIGKDEELDGCREGKECKEKKAKSKQNRRYAPRNEEKWEIINKMTDDINAKKGSRIEKYGRKSIGNKKRFEGDVVEEKEGEELNLVKNNNDQGLLNSIHSETIEATPTGHILDQLIIFHPNDGKRSRESQIISQMLLPDGGFHGKVTGDGKIEHRPADDDWVLPEVETQTMTTDRINSSSNSSQSSTPLSIFPAPDFSIIKPGDQVPPQKIIQRLEEERKRIDAVYEKLNKGDNIYDLIKRIKASDARRQPLTVLQSKMTPN*

>Mar|M.Arenaria_Scaff5003g051475

MFPNWALLLPIFGLFAYSSAQSRCKFICEADWQTRVDPTDGITYGYKVFFKSGLNFFEVNIKNYIILNFSNIFKLKAQRICRDNCAEVATIHSRDEYDFVFLTFRDLLDQCQTNASVCPIRDPNPDFNRILNSFWLGMHRIQQYTSICPGCSVDSEIVYVNSDGTPYNFGTYNADRAVLEDCTQLLDSKTGKWNDIPCHYQQAAVVCKRDCASFCQAQVPSITTTEATTTILSPGGQPGGPGQPGGPGQPGSPGADATTSAPDNGNNPGPGGNGPNGPPGQNGPNGPNGADATTSAPDNGNNPGPGGNGPNGPPGQNGQNGSNGADATTSAPDNGNNPGPGGNGPNGTPGQNGTNGQPGQNGTSFTNSPGEGGNGNGDLTTTPEPTTIEPVTITSTMRAPLPPPAPRRRSSRSPRSTQETTTSNPNIVS*

>Mar|M.Arenaria_Scaff5003g051476

MKPAPLFFILIFSFPIVLLLLNFNIITAQKSTCVPFECNDTWDLFQDNDGKVYAYKAFVQEAINFFEANALCREQCAEVASIHSKEENEFVRAIAQPLLDDCQNEIICKSRPGETSPDFDRILHSFFIGMHRIQFGRLGQCTRDPNIYCLWSDGTPCDFGNYTSVIGPHETVPPWATGNPNGIEQYGNEDCVEFYDSVNGFWNDIGCQMRLSGVICKRLCNSYCAEMDTQLK*

>Mar|M.Arenaria_Scaff5343g053330

MMKAFLKKEIDHKNKHNENNQCTDDWTTFRNPKDGHLYGYRVFVEDMINYFQAELRCRSMGAEIVSIHSAEENSFVAKLAAPLLAECQNNPLICAERVPHIDKAHELLDHILRGFWTGLHRSQFYPFYNTAVVQVWSDRSECDYGCYEGEVYCDMKIEPWGNCDPSGTNSSHGHGQSEDCTGIYLATSSGIAKWNDLACNQKIGGFICKKSCDGEHKHDVCGKDGWPYVDGKAFHAFPLHSPGNYWQALSICHKHDAQVASIHSNEENKITSALAIGQAGGNISACSWIGLHSAHGGSKERFWDDGSTVDFGKTVEPGTLPLSDDISSDDKDKESHNCRECAVINGNGHWKDLNCGANCGAVICKKECRNPE*

>Mar|M.Arenaria_Scaff5658g055026

MSIKIKYFLNKIFLFLFFNFVKNSKYNNQEYLVSEKENNFCENNWEYWPERHLCIKIFNEKIGWNVAEINCLYNQGHQISVKNIKENKYLENIARRVGGLIWLGAAQFGQLNNYMWTDGTPFYFTFWQNGIRPLFNPGKKCIKMNSLTGEWIQSCCRVAAPFVCQKPAFKINKLRNEERKEEFIQR*

>Mar|M.Arenaria_Scaff583g012096

MKLSVSKTLLLTYLSIQCIHFAFINADCPAGWNSRTDSSGIEYGYQVVLQKNITFSQAQSICSGLESDIVSIHSKEENEFIYGLIKTSGVVLFIGMQQKSDQYSPTCSFTDKSECNFGNFNGVKDPKRQEYPWVRNPSITTDGNTVHCVGIADTTYNVGKPNFKWNDVGCNDGLDGTICKKKCIGGNSGGSASNSQTAEASKASTILPANTGAPVVSTKSAIGGGSSFGPVVTSKPSGAPVVSTKPAIGGGSSIGPVVTSKPSGAPVVSTKPAIVGGSSIGPVVTAQSPKTFATLLPGLPGSTPKPVNGGGNNGSCGNCNCNDGYKCGSDDWQVKHGKDGSFAYKYFPAPNSSYFTAKAICAKYHALPCSINSEEENEFLLENVANNQLKSKKRAKRETQDKCIWTGVHIVVSDNDKEENECYCDDGKECSYGHKKGDNEDDDDDNTKEDDHDDKKKKEKEEKDKKKKDKEDKKKEKEDKKKEEKYKKKEKEDRDKKKEEKEDKNKKEKEEKDKKKKEKEDKKKQEKEEKDKKKKEKEDKKKQEKENKDKKKKEKEDKKKREKEEKDKKKKDKKDKSKDDSDEDDHKKCSCKDKKKPPSPWAPGCPSKPGGGDKKHCVGIGNDGKWKDVNCEKPGTGIICKRPCSP*

>Mar|M.Arenaria_Scaff603g012386

MINLIFISFQKFCTILSIIFLINLKTSIGQTTSSLNLDYNYESPVNSNSNENGGLNQCPDGWASRLDSSGVVYGYKVIMQDMINYYQVFWFLEKNIFYVFSLNEIIHGLVKVIRLARNLCNKEGGELVSIHSDEEEQFVAQLASSLLDQCQTNNSLCRQRVPIPSLDSMFRSFYIGLNRAAIEPYYDPTVVQTWADGSSVDYASVPTPGVPTIQIQPWGPGCPSGLNDTGSNLQKGSPEDCVCMYKGADGNINWNDISCYHKLAGGICKRTCNGARFGKMCGINGWQAIAICQSVGAKVASIHSDLQQATVTNVSQTIQTNCSWIGLHRNAILGGQINNFWDDGSPSDYGVIVQLNGGNTPWSLGCPKSLGNTSCPTSNDCNAVVPPAGLWLDLDCVSTCGAVICEKECNPLTLTQNDLFFDELNSVEKEVVD*

>Mar|M.Arenaria_Scaff6289g058096

XEFGDDILKALNKETIENGEIEVKTGEDKIGDNKIRKWRKYVIKRKSNGGKNEEEKINEGERSLLDAPPVNEKSKGNLDNKEKNKIKNVLDWNKQRASVICGDEGWHYFDGFCYKLINGKFTWEEALGECKKQNSNLVSIQSEQENDFVGSLSTKFDQNFDNFCHGFWIGMTRKYIEETDSFKSEWSDGTLVNYGNVPIKIAFHTPPWMGGQPDFAGGVEECVHSYPKLGCDNWQQIFFNQWNDARCSIKLSGAVCKKRQSIKKFEKKK*

>Mar|M.Arenaria_Scaff649g013106

MKLNFAFLISFLIFLFFSYTTEAYFGCNCQYPNNHYVPWYLSGNVENRLTFLERQTSQKINMLIARIEALERELGLIHRISMQEWNNSGAGNIYKIFNTSKTWEEAKNTCMTFGARLASIDSDYKNAFVRNMIERSFGKDGSAEVWIGLKTRAELTNNPNSHFTNFGEEEKIDGCAVMGIKGKWKIRSCSNLQPFVCEQILM*

>Mar|M.Arenaria_Scaff6528g059216

MKLSVSFLNLLTIVFLLQCIHFVYVNGDCPAGWSSFTDSNGIEYGYQVVLKNNVSYFEARRLCLDINSEIVDIHTREENEYMYSLAANTSAQNLFIGMHYKLNETSRTSEVYCTWTPDVKCDFAYFQNQSDPRRQQYPWVPIPKAQNNGNPVECVEITDTSLGIGTPVFMYNDIACADRLDGAVCKMTCSNGNNPATTFGSSTTINGQNVATISTTGSRGNNPGPRPTPPTRTAGTGAPGLPGSPGLPGLPGSGGNNPGPRPTPPTRTAGTGAPGLPGSPGLPGLPGSGGNNPGPRPTPPTRTAGTGAPGLPGSPGLPGLPGSGGNNPGPRPTPPTRTAGTGAPGLPGSPGLPGLPGSGGNNPGPRPTPPTRTAGTGAPGLPGSPGLPGLPGSGGNNPGPRPTPPTRTAGTGAPGLPGSPGLPGLPGSGGNNPGPRPTPPTRTAGTGAPGLPGSPGLPGLPGSGGNNPGPRPTPPTRTAGTGAPGLPGSPGLPGLPGSGGNNPGPRPTPPTRTAGTGAPGLPGSPGLPGLPGSGGNNPGPRPTPPTRTAGTGAPGLPGSPGLPGLPGSGGNNPGPRPTPPTRTAETTLQPSTSIGKNTPIQNTPSSAKPINTITSRPSTLQPPTVEHNTPEQPTPTPEGPIITNGPTTSSQAPTTTIAKNTPSTPGCGNCNCSVEYKCGSDDWHWKTGKNGKSYAYKYFDSDGCYFDASAICAKHNATPCSVHSEEENSFLIDSFISKSERSKRDVSQTDKCVWIGLHLVHNEKENINNCYCDDSSDCSFGYKNVTKHEKKKNKKKKDKKCSCNKTKKPKTTTPFSLVTEPSEPSGPWAPGCPSKPGSDDGSYGKKHCVAIDNNGKWKDCSCERPGTGIVCKKECSP*

>Mar|M.Arenaria_Scaff656g013200

MSITSLIPLILFSLILPYAHCSCSSKWTTRVDSDGSTYGYLAIISDLITIFEGNTICKQQGGEVVSIHSAEENNFIGQIAGPLMKLCQETNACQQRVDHNNNRTAFLDMLYRSFWIGLSRVQFAHPLDNPTVHSAWLDGTPVDYGRYDGLPIPNRDVRPWAYGSPSSLNATTSGEIEGCTQMFLNDNDPQWNDISCYALLGGVICKRNCSADCPNEDNSTITTSSTTTAVGLTTQTTEETTTEVQYDEATTEDIETSTPNSCGNNSNICGADGWKWKCFKNGKCYSYHRYSVKGSYWKALAKCRKHHASVCSIETEEENEYVCKHYCGCKGKDVWIDMHRMVSEDGTNIVCLDGEDKKCYYETAALGFVPVRIRPARIRPMRIRPPEQKARIRPN*

>Mar|M.Arenaria_Scaff656g013207

MKLCQETNACQQRVDHNNNRTAFLDMLYRSFWIGLSRVQFAHPLDNPTVHSAWLDGTPVDYGRYDGLPIPNRDVRPWAYGSPSSLNATTSGEIEGCTQMFLNDNDPQWNDISCYALLGGVICKRNCSADCPNEDNSTTTTSSTTTAVGLTTQTTEETTTEVQYDEATTEDIETSTPNSCGNNSNICGADGWKWKCFKNGKCYSYHRYSVKGSYWKALAKCRKHHASVCSIETEEENEYVCKHYCGCKGKDVWIDMHRMVTEYGTNIVCLDGEDKKCYYETDSNSRNPWANGCPADASNMDDGKYGKKNCCVISKGKWKDVGCTRPISKVLCKKECVKPR*

>Mar|M.Arenaria_Scaff656g013211

MIAFPSAKLCSLIISVIILAIYCILPTNGQATCPSDWISRVDPIDGITYGYKVITRDWLNYYEAQGYCVRAGGQVASIHSAAENAFIANISAAQLKQCQTNDSVCATRIAHTSAGWAFLDSLMRSFWFGMNRVQYQPSYNNTLDCSYPDGTPCDYGRFDGVANANTNSPPWSPACPSGSNSSNGAGDLEYCTMFFNATTTLVWNDMSCYQKLGGVVCKMNCSATKTTQTCPSSSVVTANAAATTKAAVVTSGSGSAGGSTAKSLTTKSSGTSNGSCSCNGGSCGGGGYKCGSDDWQWKINKKNGKSYAYKAFNYTGCYWQGLAICKKYKAVPCSIQSSDDNDFIVKTVCSGLLSSSSSNSSRKRRATSDTCIYTGIHRQVTNPSSGGVSCTCSDGSSCDYGNDDAHGDITTISFTSTTSRATALKASTTQKAATTTTQKSTTKLATSPYISTSTAGPSNPWAPGCPANSTTGQRDNGGYGSRNCIGINGEGKWVDQSCDRPATAIICMKECDQP*

>Mar|M.Arenaria_Scaff6696g059989

MITLSFVSLFYLSLPALIAGQCSSDWQTNVGSDGFTYGYKAIVSDMVNFFEAHDVCAALGAQVASIHSAQENSFVISIASSLLDQCENNNAVCKQRVDRSTTSSLRLDRLLRSFWIGMHRVQYAPFYNTSVDCCWSDGTQCDYGRFDGVADPNQNIPPWSTGNPSGSSSSITSAGWNVEDCVEITNDNGVLWNDIGCYHKLGGVVCKKNCTNTCPPVTTQAPAVTSKPITTAPVVTTTTDDDTGGYICGSDDWQWKNNGNGKAYAYKWFNQSGSFWQLHDLCYDSCATPCSIESEEENQHIVNHVCAGAFSSSSSGRKKRATDSQCIYTA*

>Mar|M.Arenaria_Scaff682g013606

MIILSFVSLFYLSLPALIAGQCSSDWQTNVGSDGFTYGYKAVVSDMVNFFEAHDVCAALGAQVASIHSAQENAFVISIASSLLDQCENNNAVCKQRVDRSTTSSLRLDRLLRSFWIGMHRVQYAPFYNTSVDCCWSDGTQCDYGRFDGVADPNQNIPPWSTGNPSGSSSSITSAGWNVEDCVEITNDNGVLWNDIGCYHKLGGVVCKKNCTNTCPPVATQAPAVTSKPITTIPVVTTTTDDETGGYICGSDEWQWRNNGNGKAYAYKWFNQSGSFWQLHDLCYDSCATPCSIESEEENQHIVNHVCAGAFSSSSSGRKKRATDSQCIYTGFHRYQESPPNGPIGCSCDDGKSCDYGNDDTTTTTSTTSTTKAATTTAKAITTPRPPTSTIRATTKATTIITSTTKPNNPWAPGCPANSTTGLGDSGGYGTKNCVGFDSNGKWRDQACERPGKGIVCKKPCIPPAGS*

>Mar|M.Arenaria_Scaff7342g062916

MMEAFLKKEINHKNNEHNENNQCTDDWTTFRNPKDGHLYGYKVFVEDMINYFQAEVKCRSMGAEIVSIHSAEENSFVAKLAAPLLAECQNNPLICAERVPHIDKAHELLDHILRGFWIGLHRNQFYPFYNPAVVQVWSDRSECDYGCYEGEVYCDMNIEPWGNCDPSGTNSSHGHGQSEDCTGIYLATSSGIAKWNDLACNQKIGGFICKKPCEGEHKHDVCGKDGWPYVDGKAFHAFPLHSPGNYWQALSICHKHDAQVASIHSNEENKITSALAIGQAGGNISACSWIGLHSAKGGSKERFWDDGSNVDYGKHVEPGTLPLSDDISSDDKSKDTHHCHECAAINGNGHWKDLNCGANCGAVICKKGCRNPE*

>Mar|M.Arenaria_Scaff7585g063931

MIFLIFISFQKFRTILSIIFLINFKTSNGQTTSSLNLDYNYESPVNSNSNENGGLNQCPDGWASRLDSSGIVYGYKVIMQDMINYYQARNLCNKEGGELVSIHSDEEEHFVVQLASSLLDQCQTNNSLCRQRVPIPSLDSMFRSFYIGLNRAAIEPYYDPTVVQTWADGSSVDYASVPTPGVPTIQIQPWGPGSPSGLNDTGSNPQKGSPEDCVCMYKGADGNINWNDISCYHKLAGGICKRTCNGASFGKMCGINGWQAIATCQSMGAKVASIHSDLQQATVTNVSQTIQTNCSWIGLHRNAILGGQIHNFWDDGSPSDYGVIVQLNGGNTPWSLGCPKSLGNTSCPTSNDCTAVVPPAGLWLDLDCVSTCGAVICEKECNPLTLTQNDLFFDELNSVEKEVVD*

>Mar|M.Arenaria_Scaff7862g065029

MLHQQQQQQQQQQKPKFRLQLEDVGNPQPCPDNPIRLLASRTPQYIYSPYDENKLYPPDTDCQFLIEATDKFHRIHLTIIESDLEEALFTDCNDYVSVRDREGNETSIKEVARWCGQDYPAAIASASDSLLVHFHSDSIIQKRGFNISFVQFDIDTCPPDWISDGISSPYCYKQFVLPHILPWYEAQKECNFERANLATFQNEADYAFIVESYSQTHSFPWVGYSDANVEGIYESIDRNVPLWPENFPLKHENEMKDCVYLDWNKRDQIVVYEIDDCRNRRPFLCKKRRDGTEVPVILPAGMIRRGFRDFTMDYTLLVVVVIFALLALVVGCVLFHKYKERNNQIINIDMNQRLVQQQHGQQPGNKDKAAAALARQKAKERERRELREQKYAETSNNNRGGGRGGGGGIATTKSPFESQDSNASFPLQTFQSTFAQRMTSTTTGNVEPLDTAAALAEASATASQQMREHEATKLHPQQLSAEIVSVDSPPEDDLNSSPRIRMSPNQPSFVEEGRKISEKKIRKGWMGAGVSGGGTTDREEEEEEKDRHNMGYEPDSEEEEHEENERQKIRRASKINGEGEGGEENFLMELNDEGGRKNTGENNGENIGENEELEQYTQHNHMEEELEQYSGGGEQFKQYKHKNVIESEQYINSQQFKQLESDDGVQQTVVQVELHGENGEEGGLGQFKDKRTKEERINNFDEKMMDSYAIGDHFKAAKVATSAIASAAIAATEHPIRPPIELMRTRTTSTDKAKSPPPPKEAKESEKIGESGKSSREATREGKEETREEKETFPEKISGKVNISSSTATNIVKTEGEGTGTRIRIKRKTFDRPPPVGPLDNVSAISLDEFWQQQK*

>Mar|M.Arenaria_Scaff82g002512

MIAFSSAKLCSFIFSVAIFASYAILPTNGQATCPSDWISRVDPIDGITYGYKVITRDWLNYYEAQGYCVRAGGQVASIHSAAENVFIANISAAQLKQCQTNDSVCATRIAHTSAGWAFLDSLMRSFWFGMNRVQYQPSYNNTLDCSYPDGTPCDYGRFDGVANANTNSPPWSPACPSGSNSSNGAGDLEYCTMFFNATTTLLWNDMSCYQKLGGVVCKMNCSATKTTKTCPSSSVVTANAAATTKAAVVTSGSGSAGGSTAKSLTTKSSGTSNGSCSCNGGSCGGGGYKCGSDDWQWKINKKNGKSYAYKAFNYTGCYWQGLAICKKYKAVPCSIQSSDDNDFIVKTVCSGLLSSSSSNSSRKRRATSDTCIYTGIHRQVTNPSSGGVSCTCSDGSSCDYGNEDAHGDITTISFTSTTSRATALKASTTQKAATTTTQKSTTKLATSLRSPIWDYYTIFINVDGVRCVQCNNCSQQYPFPPNSTSANFRSHLESKHEKQFKEYLNKAKRNKQEYEGYTFSKILLKLIEIALIHLTFLFLRFTPNLNAWEFPK*

>Mar|M.Arenaria_Scaff897g016596

MTLIGLIIAFCIIPSAVNADCLSGNWTTRVATDGNTYGYQVLARDWLNFYEARALCLGVGGDVASIHSNAENEFVRQLAAPYIIGCQTNKTTCGARATTSLDTILSVVWLGMHRCQYFPSYNATVDCINSDGTPCDYAATNAVILTGPSPFPWAWGNPSGSDSGGGAGLIEDCVSMYNATSGEWNDLSCFQKLGGVVCKRNCTGTCGSTSIKTALITSPSGITDTSCTSGNWTKRAGEDGNTYGYQIVMNDWLNFYEANAKCLALGAEVVSVHSVAENEFIRQLAAPYITACQTNTSVCAQRTSTTQDIQWRSLWLGLHRCAFYPTYNATVDCINSDGTVCDYLNITGASGTETGTASGQQEACAAMYSATTGQWNDIACYNKLGGVICKKNCSNACGVATTTTTQLTTTTTQPTTTTTLPTTTTTLPTTTTTLPTTTTTLPTTTTTEPSTTTTAEPTTTTTEPSTTTTAEPTTTTTEPSTTTTFNCAQPPVTSLLSYNTSDPKIQTGSNATEGVCECPADPGNSNVFFIPVTTITTGSSASNSSVIMKCSKMQDFCICDEDDICWKVINAYSLVVINSFCDPTCHNYARLQNAAPFNQTFKSDCGRTITLADELTPIPNTNRNTFKPLGSSADYYIKAASIRCLQTGQTCTPIKCSGTRKPSPCPTTTTTLPTTTTTLPTTTTTLPTTTTTLPTTTTTLPTTTTTLPTTTTTLPTTTTTLPTTTTTLPTTTTTFTNYHYHPSNNHNDHTNNHNNLTNNHNNLTNNNYHPSNNHNNLTNYHYHPSNNHNDLTNNHNNLTNNHNNLTNNDNNLTNYHYHPSNNHNNLTNYHYHPSNNHNDLTNNHNNLTNNHNKVTINYYYPTYNNTCTVNWEELKFNIWLPIEIDCGNCATGQSRVIYDKNTLTSNQIVKNNAASQCIFNCKDPDVSQICYTTDAVSVARINCTDPSQFCVCASNVKKGCYQVTNTALLADYSIFANASYTFLVINSGSSNIANSQTGASYKFTAGPSFVVGNTKTLNAPVLGISCNGCAPIHNPGADVIKPCTPGVSCNGCAPIHNPGADVIKPCTPGGG*

>Mar|M.Arenaria_Scaff9049g069469

FCYKLINGKFTWEEALGECKKQNSNLVSIQSEQENDFVGSLSTKFDQNFDNFCHGFWIGMTRKYIEETDSFKSEWSDGTLVNYGNVPIKIAFHTPPWMGGQPDFAGGVEECVHSYPKLGCDNWQQIFFNQWNDARCSIKLSGAVCKKRQSIKKFEKKK*

>Mar|M.Arenaria_Scaff973g017615

MKLSVSKTLLLTYLSIQCIHFAFINADCPAGWNSRSDSSGIEYGYQVVLQKNTTFSQAQSICSGLESDIVSIHNKEENEFIYGLIKTSGVVLYIGMQQKSDQYSPTCSWPDKSDCNFGNFNGVKDPKRQEYPWVRNPSITTDGNTVHCVGIADTTYNFGKPNFKWNDVGCNDGLDGTICKKKCIGGNSGGSASTSQTPEASKASTILPSNTEAPVVSTKSSIGGGSSIGPVVTSKPSGAPVVSTKSAIGGGSTIGPVVTSKPSGAPVVSTKSAIGGGSTIGPVVTSKPSGAPVVSTKSAIGGGAPRVSTKSAIGGGSTIGPVVTSKPSGAPVVSTKSAIGGGSTIGPVVTSKPSGAPVVSTKSAIGGGSTIGPVVTAQSPKTFATLLPGFPGSTPKPVNGGGNNGSCGNCNCNDGYKCGSDGWQVKHGKDGSFAYKYFPAPNSSYFTAKAICAKYHALPCSINSEEENEFLLENVANNQLKSNKRAKRETQDPACIWTGVHIVVSDNDKEENECYCDDGKECSYGHKKENNEDDDDDNTKEDDHDDKKKKGKEEKDKKKKEKEDKKKEKEDKDKKKEEKDKKKEDKDDKNKKEKEEKDKKKKEKEDKDKKKEEKEDKKKQEKEEKDKKKKEKEDKKKQEKEEKDKKKKEKEDKKKKEKEEKDKKKKDKKNKSKDDSDEDDHKKCSCKDKKKPPSPWAPGCPSKPGGGHKKHCVGIGNDGKWKDVNCEKPGTGIICKRPCSP*

>Mar|M.Arenaria_Scaff989g017820

MIYLIFISFQKFCTILSIIFLINLKTSIGQTTSSLNFDNNYESPVNSNSNEEGGSNQCPDGWASRLDSSGIVYGYKVIMQDMINYYQARNLCNEEGGELVSIHSDEEEQFVAQLALNLLDQCQTNNSLCRQRVPIPSLDSMFRSFYIGLNRAAIEPYYDPTVVQTWADGSSVDYASVPTPGVPTIQIQPWGPGCPSGLNDTGSNPQKGSPEDCVCMYKGADGNINWNDISCYHKLAGGICKLTCNGGNFGKMCGINGWQAIATCQSLGAKVASIHSDLQQATVTNVSQTIQINCSWIGLHRNAILGGQIHNFWDDGSPSDYGVIVQLNGGNTPWSLGCPKSLGNTSCPTSNDCTAVVPPAGLWLDIDCVSTCGAVICEKECNPLTLTQNDLFFDELNSVEKEVVD*

>Mja|M.Javanica_Scaff10427g058767

MINLIFISFQKFCTILSIIFLINLKTSIGQTTSSLNLDYNYESPVNSNSNENGGLYQCPDGWASRLDSSGIVYGYKVIMQDMINYYQARNLCNEEGGELVSIHSDEEEQFVAQLALNLLDQCQTNNSLCRQRFPIPSLDSMFRSFYIGLNRAAIELSYDPTVVQTWADGSSVDYASVPTPGYXPTVVQTWADGSSVDYASVPTPGVLNYDPTVVQTWADGSSVDYASVPTPGVPTTQIQPWGPGCPSGLNDTGSNPQKGSPEDCVCMYKGADGNINWNDISCYHKLAGGICKLTCNGGNFGKNVWHKWMASGK*

>Mja|M.Javanica_Scaff10427g058768

MATCQSLGAKVASIHSDLQQATVTNVSQTIQINCSWIGLHRNAILGGQIHNFWDDGSPSDYGVIVQLNGGNTPWSLGCPKSLGNTSCPTSNDCTAVVPPAGLWLDIDCVSTCGAVICEKECNPLTLTQNDLFFDELNSVEKEVVD*

>Mja|M.Javanica_Scaff10949g060323

MKDWLNFYEANAQCLALGAEVVSIHTVAENEFIRQLAAPYITACQTNTSVCVSRVSTTQDTQWRSLWLGLHRCAFYPTYNATVDCINSDGTVCDYLNITGGPSGTETGTASGQQEACAAMYSATTGQWNDIACFNKLGGVICKKNCSKACGVASTTTTQFSTTTTQLSTTTTAEPTTTTTLTTTTTTLPTTTTTLPTTTTTEPSTTTTLPTTTTTEPSTTTTAEPTTTTTEPSTTTSFNCAQPPVTSLLSYNTSDPNIQTGPNATEGVCECPADPGNNNVFFIPVTTITTGSSASNSSVIMKCSKMQDFCICDEDDICWKVINAYSLVVINSYCDPTCHMYARLQNAAPFNQTFESDCGRTITLADELTPIPNTNRNTFKPLGSSADYYIKARLAPQSNVRGTRKPPPCPTTTTTMPTTTTTLPTTTTTLPTTTTTLPTTTTTLPTTTTTLPTTTTTLPTTTTTLPTTTTTLPTTTTTLPTTTTTLPTTTTNLPTTTTTLPTTTTTLPATTTTLPTTTTTLPTTTTNLPTTTTVLPTTTTPPINCADCSAGQSKVIYEKNTLLSNQTINNLAATECIYNCKDKDVSQACYNPSPISVVRIRCTDSTKFCVCTSDNKGCFTVTQSAPLYQADYLIYANASYTFLALNTGASQIKNSGTGKTYDFQTSMTFIPNDNTFLNAPYLAISCNGCNPIHNNGMDVQKPC*

>Mja|M.Javanica_Scaff10949g060324

MGMSPILFSLIFGLIPTLVSADCSVGNWTTRVDSDGNTYGYQVLMRDWLNFYEARALCLGVGGDVVSVHSNAENEFVRQLAAPYIAACQTNKTVCGSRATTSLDFYLSVVWIGMTRCQYFPSYNSTVDCVYSDGTTCNYATSSSVYPWGVGSPSGSDSGGGAGLIEDCVSMYNGTSGEWNDVSCFQKLGGVVCKRNCTGTCGSTSIKAALLTSPSDITDSYELFIGQLD*

>Mja|M.Javanica_Scaff11058g060678

MITLSFVSLFYLSLPALIAAQCSSDWQTNVGSDGFTYGYKAIVSDMVNFFEAHDVCAALGAQVASIHSAQENAFMISIASSLLDQCENNNAVCKQRVDRSTTSSLRLDRLLRSFWIGMHRVQYAPFYNNSVDCCWSDGTQCDYGRFDGVADPNQNIPPWSTGNPSGSSSSITSAGWNVEDCVEITNDNGVLWNDIGCYHKLGGVVCKKNCTNTCPPVTTQAPAVTSKPITTAPVVTTPTDDDTGGYICGSDDWQWKNNGNGKAYAYKWFNQSGSFWQLHDLCYDSCATPCSIESEEENQHIVNNICAGAFSSSSSGRKKRATDSECIYTGFHRYQESPPNGPIGCSCDDGKSCDYGNDDTTTTTSTTSTTKAATTTKAATTPRPSTSTSRATTKATTIITSTTKPNNPWAPGCPANSTTGLGDTDGYGTKNCVGFDSNGKWRDQACERPGKGIVCKKPCIPPTGS*

>Mja|M.Javanica_Scaff12399g064581

MKLSVSFLNLLTLVFLLQCIHFVYVNGDCPAGWSSFTDSNGTEYGYQVVLKNNVSYFEARRLCLDINSEIVDIHSREENEYMYSLASNTSAQNLFIGMHYKLNETSRTSEVYCTWTPDVKCDFAYFQNQSDPRRQQYPWVPIPKAQNNGNPVECVEITDTSLGIGTPAFMYNDIACADRLDGAVCKMTCSNGNNPATTFGSSTIINGQNVVTISTTGSGGNNPGPRPTPPTRTAGTGAPGLPGSPGLPGLPGSGGNNPGPRPTPPTRTAGTGAPGLPGTYT*

>Mja|M.Javanica_Scaff12412g064612

MKLSFSKINLLAVAVLISFSFVNCGCPDGWGTIDNVNGFKVFTSTDPINLFMAVPVCKANGGIIASLYSSDESAFIAQMLGNDTPVWVGAVMKNNGDGSFSCKMLDGGDCPSEIQNSFQNQPTDTETPIYGTQLISGKWNVVDCAERVYAVACKMSCDQ*

>Mja|M.Javanica_Scaff12459g064743

MFEAIYNAVPVCKANGGIIASLYSSDESALIAQMVGNDTPVWVGAVMKNNGDGSFSCKMLDGGDCPSEIQNSFQNQPSDTETPIYGTQLISGKWNVVDCAERVYAVACKISCDQ*

>Mja|M.Javanica_Scaff12780g065615

MKKRESYINEEENNGKTKLSSNSIEKRLDRLEANQQNLRRATLADWPAFSVDKRVRIFDHTRSTWIDAQRECSEHAGTLLEIDSESENERINEMLTNSGNSNRPNDHYWIGVQIMLQFSNGSSIIGNYSNFEEQNNSENTQNQKPSKGLVMKRCAAISASQTTKSEGRWLSLECSEKHGFICQL*

>Mja|M.Javanica_Scaff128g002342

MYFYLNKIILLFLFSIFVSCIPKYQEEFGDDILKALNKETIENGEIEVKTGEDKFGDNKIRKWRRYVIKRKNGGKNEEEKISEGELSLLEAPPINEKSKGNLDNNEKNRIKNVLDWNKQRAPVICGDEEWHYFDGFCYKLINGKFTWEEALGECKKQNSNLVSIQSEQENDFVGSLSTKFDQNFDNFCHGFWIGMTRKYIEETDSFKSEWSDGTLVNYGNVPIRVAFHTPPWMGGQPDFAGGVEECVHSYPKLGCDNWQQIFFNQWNGRKIREEKIFKFIFVDARCSIRLSGAVCKKRQSIQKI*

>Mja|M.Javanica_Scaff12960g066130

MLTTSSLNFDNNYESPVNSNSNEEGGSNQCPDGWASRLDSSGIVYGYKVIMQDMINYYQARNLCNEEGGELVSIHSDEEEQFVAQLALNLLDQCQTNNSLCRQRVPIPSPYDPTVVQTWADGSSVDYASVPTPGVPTIQIQPWGPGCPSGLNDTGSNPQKGSPEDCVCMYKGADGNINWNDISCYHKLAGGICKLTCNGGNFGKMCGINGWQVANDKEYKQLPISYPGNYWQARKISIK*

>Mja|M.Javanica_Scaff1320g014735

MEAFLKKEINHKNKNNENNQCTEDWTTFRNPKDGHLYGYRVFVEDMINYFQAELRCRSMGAEIVSIHSAEENSFVAKLAAPLLAECQHNPLICAERVPHIDKAHELLDHILRGFWVGLHRSQFFPFYNPAVVQVWSDGSECDYGCYEGEVYCDMKIEPWGNCDPSGTNSSHGHGQSEDCTGIYLATSSGIAKWNDLACNQKIGGFICKKPCDGEHKHDVCGKDGWPYVDGKAFHAFPLHSPGNYWQALSICHKHDAQVASIHSNEENKITSALAIGQAGGNISACSWIGLHSAKGGSKERFWDDGSSVDYGKHLEPGTLPLTDDISSDDKDNESQHCRECTAINGNGHWKDLNCGANCGAVICKKECRNPE*

>Mja|M.Javanica_Scaff13232g066838

MLHFITILLFIFHLAIAQNGDNLNDYDEDIIMTTANYNAGLNADNANNAKPSTASTTTGDNIMVLENLNNATGNPADQADDSSDKPLYPNANPDENGGANQCPDGWATRQDKDGNVYGYKVIMQDMVNYYQARLLCIKEGGELVSIHSDEEEQFVVQLATPLLDKCQTDTSVCKQRVQIPSLDKMYRSLYIGLNRAAIEP*

>Mja|M.Javanica_Scaff13232g066839

MKISSYEPTVVQAWSDGTPVDYASVPTPGVPTTLIPPWGAGCPSGLNDTGNTPQKGSPEDCVCMFKYDSVNWNDISCYHKLGGGICKRKCNGGGGGGGQPCGCGKVNCGTDGWSQSANGKGYKIFQLPSPGNYWQALAMCANNGAKVASYHSDQQKSAITNICQSSNCAWIGLHSATDGNKYWDDGSQYDYKNLLEVSLCQKKPKSPSCGGGNPPSGDNCPKDCTAVSSSGQTTDQSCESSCGAVICEKDCN*

>Mja|M.Javanica_Scaff13373g067183

MKVPSFLVCLSIILFFNSLTAQKVPNCPARSYNCDTDWQLFTDPNSGVEYGYKAFIQDSINFYEADIFCRNRCAEVVSIHSQQEQDFVTKIAGPLLTKCQFTDACSERMEHTNKSAANFDRMLRGFWIGMNRVWGTLLNHDTIDTNVTCIWSDRSKCDYGSVQDGSVTPDTVSPPWARGNPNGANPGGDPLIVQACVQMVEGRHGGWNDISCTYRLGGLICKRKCSSYCPAQDA*

>Mja|M.Javanica_Scaff14377g069743

MRYNLHIRIFHPKKVLGPPYGFSIFNRLYLDATPVSDDTRTDLTTLIGTEGTTSTPNTSCPPDYTFFKEGNCCIQISSPEKKPLTWKEASEEAKKNGARMASIHFKKEDDFIRDLVAKQPKVEGKDNPVYFTGLNVNATNGKINNFAWSDNTRVNYGSPKDIKPNTDPWGPKTTFNGNTGFGGISKDPSGKASWGLYDPNQKGGYVWKIDLSKKVPEPGNPTGGVPGGNNGLPAGYKCTTGCNPSSIKLKAEKGLPSPVDEGPTEKNGCQTDVLSCTTNQPIATITFGTKGSVQSDNPNKNNVRAKVICANEEASGKKGWLRVKANNDPNLFFFVDEAHCEKSS*

>Mja|M.Javanica_Scaff14378g069747

XYEPTVVQAWSDGTPVDYASVPTPGVPTTIIPPWGAGCPSGLNDTGNTPQKGSPEDCVCMFKYDEVNWNDISCYHKLGGGICKRKCNGGGGGGGGQPCGCGKVNCGTDGWSQSANGKGYKIFQLPSPGNYWQALAMCANNGAKVASYHSDQQKSAITNICQSSNCAWIGLHSATDGNKYWDDGSSYDYKNLLEVSLCQKKPKSPSCGGGNPPSGDNCPKDCTAVSSSGQTTDQSCESSCGAVICEKDCN*

>Mja|M.Javanica_Scaff15466g072394

MKLSVSFLNLLTIVFLLQCIHFVYVNGDCPAGWSSFTDSNGIEYGYQVVLKNNVSYFEARRLCLDINSEIVDIHTREENEYMYSLAANTSAQNLFIGMHYKLNETSRTSEVYCTWTPDVKCDFAYFQNQSDPRRQQYPWVPIPKAQNNGNPVECVEITDTSLGIGTPVFMYNDIACADRLDGAVCKMTCSNGNNPATTFGSSTTINGQNVATISTTGSRGNNPGPRPTPPTITAGTGAPGLPGSPGLPGLPGSGGNNPGPRPTPPTRTAGTGAPGLPGSPGLPGLPGSGGINPGPRPTPPTRTAGTGAPGLPGNNPGPRPTPPTRTAGTGAPGLPGSPGLPGLPGSGGNNPGPRPTPPTRTAGTGAPGLPGSPGLPGLPGSGGNNPGPRPTPPTRTAGTGAPGLPGSPGLPGLPGSGGNNPGPRPTPPTRTAGTGAPGLPGSPGLPGLPGSGGNNPGPRPTPPTRTAGTGAPGLPGSPGLPGLPGSGGNNPGPRPTPPTRTAGTGAPGLPGSPGLPGLPGSGGNNPGPRPTPPTRTAGTGAPGLPGSPGLPGLPGSGGNNPGPRPTPPTRTAGTGAPGLPGSPGLPGLPGSGGNNPGPRPTPPTRTAGTGAPGLPGSPGLPGLPGSGGNNPGPRPTPPTRTAGTGAPGLPGAPGLPGSPGLPGLPGSGGNNPGPRPTPPTRTAGTGAPGLPGSPGLPGLPGSGGNNPGPRPTPPTRTAGTGAPGLPGGYNSIN*

>Mja|M.Javanica_Scaff15493g072469

MHLPSILIILPILFSSILTQQIPNCQTRIFQCDADWDTRTDPNTGDVYGYKAFIGNKMNFYEADVFCRARCAEVVSIHSQEEQNFVRRIAAPVLNRCQFTSACAPVVQSSNGTFDRQLRSFWIGLNRVTGTLKDAHSVDSSVYQLWSDRTEVDFGAYPNNSVNSNTNSPPWTRGNPNGVNSADPQIIEDCTQMVESRQGGWNDFPCNWRISGVICKRKCTSYCAAQDV*

>Mja|M.Javanica_Scaff16387g074472

MPLILFSLIFGLIPTLVSADCSVGNWTTRVDSDGNTYGYQVLMRDWLNFYEARALCLGVGGDVVSVHSNAENEFVRQLAAPYIAACQTNKTVCGSRATTSLDFYLSVVWMGMTRCQYFPSYNATVDCVYSDGTTCNYATSASVYPWGVGSPSGSDSGGGAGLIEDCVSMYNGTSGEWNDVSCFQKLGGVVCKRNCTGTCGSTSIKTALLTSPSDITDTSCTSGNWTKRAGEDGNTYGYQVVMKDWLNFYEANAQCLALGAEVVSIHTVAENEFIRQLAGPYITACQTNTSVCVSRVSTTQDIQWRSLWLGLHRCAFYPTYNATVDCINSDGTVCDYLNITGGPSGTETGTASGQQEACAAMYSATTGQWNDIACFNKLGGVICKKNCSKACGVASTTTTQLTTTTTQPSTTTTAEPTTTTTLPTTTTTLPTTTTTLPTTTTTEPSTTTTQPTTTTTEPSTTTTAEPTTTTTEPSTTTSFNCAQPPVTSLLSYNTSDPNIQTGPNATEGVCECPADPGNNNVFFIPVTTITTGSSASNSSVIMKCSKMQDFCICDRPNATEGVCECPADPGNNNVFFIPVTTITTGSSASNSSVIMKCSKMQDFCICDEDDICWKVINAYSLVVINSFCDPTCHNYARLQNAAPFNQTFESDCGRTITLADELTPIPNTNRNTFKPLGSSADYYIKAASIRCLQAGQTCTPIKCSGTRKPSPCPTTTTTMPTTTTTLPTTTTTLPTTTTTLPTTTTTLPTTTT*

>Mja|M.Javanica_Scaff17071g075924

MKPAPLYFIIIFSFPIVLFFLKFNIITAQKSTCEPFECNDTWDLFQDNDGKVYAYKAFVQESINFFEANALCREQCAEVVSIHSKEENEFVRAIAQPLLDDCQNEVICKSRPGETNPDFDRILHSFFIGMHRIQFGRLGQCTRDPNIYCIWSDGTPCDFGNYTGVIGPHETVPPWATGNPNGIEQYGNEDCVEFYDSVNGYWNDIGCQMRLSGVICKRLCNSYCAEMDTQLK*

>Mja|M.Javanica_Scaff1720g017835

MLGTSIRCVVASLLVLQAYANKMDPAYQTDDDRNQPVESVPSEQAPWISGPQNQKYQFHIGKQSWLSAREKCLAQNADLVSIESIEEMQWLLSHYKPQFNHLRERQVQIGLLLDTIEGEAGSNSDSGLTSREWRWVNGKPLNLEITKWTMGEPFDHAKGKERCALLNINERRLDDVDCDLGSGAGFNYRFVCQRSHEKHIEHESLNNPLWKKLEDILTFFGISDREEDKKNCSLPIGAKEEGYWERAFNKGEGNSETQHKEKNIEKADSITEKSELETPKGIEKISKTNKEDRESVLEDKTIIEKKVITNKNEETTNKLSSPPEQKLVKDVEKVVEEKKVSKVGGVTQHATSKIIGGEDEQPPNELENTRPTGGAVVLRRVESHVSASSSVDNKKNNQEKLKEGNEEAKIESHVSASSSVDNKKNNQEKLKEGNEEAKSTKLEEEQSKILKVAGTEEANLREKEASDSSDSNHGQSGLSVREKSQIINLNSRENIQSSLNPKKPLIISNDPSDLKEDRNTVSNKNTSDNEKENKNIQNLSKSVQITEKKLGEDEISEIKQYTLENKYIVNTPKVDKKNDEKIKEDKLNAEKISEEKISEKEEKKEDKIRNGSDKDEKKGGNEEITIVDERKTRKEDKESERIEENNKEDREVKRDEGRKEENENKSDELKKNEATREKDIKEEKKVINDNDIKEEKLKSEETKIRKEKHDEKEEKEDRKNLLPKSNNIRDESKKLDLENIKEEKLEANLIKNKINNEIRDEKKNEEVLAQNKVEEKIREEQSLELKNKDKKDDDKSGKKSENAEEKTVEIKEAKEDSNQESMKANNKEKDGLNNETTHDVKNEATNSKINELDRETKKEVKMEENREAKDEAKLEAKHKMKIEATEANEKESVNDAKSEAQSETKGEARHEAETKISEDKIIKTNKPTKHPDPLSVVAETLERSLSSKEETIEKKKDEAENLIKEKRQKPDGVRDRIQHLEKIITEVQHMMGIGEKGGEGELIEKTNKVLEEDEEEKKYEIKKEEGRKNIKEVKKEKEEKIRVEEKEESKEGSGSSEKLTEATPIAEVEELSSPEVEPKSHSPSVSSSDESEESEYSPKHIKTKNIKDNKAQREQPVKVNNEATRKSGTVKEFGTVNKSHSIISDSLDEQRKSMAAMEFDFEEAALQAKAHPGINSGDLVKNIILIGKDEELDGCREGKECKEKKAKSKQNRRYAPRNEEKWEIINKMTDDINAKKGSRIEKYGRKSIGNKKRFEGDVVEEKEGEELNLVKNNNDQGLLNSIHSETIEATPTGHILDQLIIFHPNDGKRSRESQIISQMLLPDGGFHGKVTGDGKIEHRPADDDWVLPEVETQTMTTDRINSSSNSSQSSTPLSIFPAPDFSIIKPGDQVPPQKIIQRLEEERKRIDAVYEKLNKGDNIYDLIKRIKASDARRQPLTVLQSKMTPN*

>Mja|M.Javanica_Scaff17910g077673

MITLTYVSLFYLSLPALIAGQCSSDWQTNVGSDGFTYGYKAIVSDMVNFFEAHDVCAALGAQVASIHSAQENAFMISIASSLLDQCENNNAVCKQRVDRSTTSSLRLDRLLRSFWIGMHRVQYAPFYNTSVDCCWSDGTQCDYGRFDGVADPNQNIPPWSTGNPSGSSSSVTSAGWNVEDCVEITNDNGVLWNDIGCYHKLGGVVCKKNCTNTCPPVTTQAPAITSKPITTIPVVTTTTDDDTGGYICGSDDWQWKNNGNGKAYAYKWFNQSGSFWQLHNLCYDSCATPCSIESEEENQHIVNNICAGAFSSSSSGRKKRATDSECIYTGFHRYQESPPNGPIGCSCDDGKSCDYGNDDTTTTTSKTSTTKAATTTTKAITTPRPSTSTIRATTKATTIITSTTKPNNPWAPGCPANSTTGLGDSDGYGTKNCVGFDSNGKWRDQACERPGKGIVCKKPCIPPTGS*

>Mja|M.Javanica_Scaff17991g077850

MLHSITILLFIFHLAIAQNGDNLNDYEDIIMTTANYNAGLNADNANAKPSTASTTTGDNIMVLENLNNATGNPADQADDSSDKPLYPNSNPNENGGANQCPDGWATRQDKDGNVYGYKVIMQDLVNYYQARLLCIKEGGELVSIHSEEEEQFVVQLATPLLDKCQTDTSVCKQRVPIPSLDKMYRSMYIGLNRAAIEPYYEPTVVQAWSDGTPVDYASVPTPGVPTTIIPPWGAGCPSGLNDTGNTPQKGSPEDCVCMFKYDSVNWNDISCYHKLGGGICKRKCNGGGGGGGQPCGCGKVNCGTDGWSQSANGKGYKIFQLPSPGNYWQALAMCANNGAKVASYHSDQQKSVITNICQSSNCAWIGLHSATDGNKYWDDGSQYDYKNLLEVSLCQKKPKSPSCGGGNPPSGDNCPKDCTAVSSSGQTTDQSCESSCGAVICEKDCN*

>Mja|M.Javanica_Scaff1838g018739

MGTKLLFRIQLSFYEADIFCRARCAEVVSIHSQQEQDFVTKIAGPLLTKCQFTDACSERMEHTNKSAANFDRMLRGFWIGMNRVYGTLLNHNTIDTNVTCIWSDRSKCDYGSVQDGSVTPDTVIPPWARGNPNGANPGGDPLIVQACVQMVEGRHGGWNDISCTYRLGGLICKRKCSSYCAAQDV*

>Mja|M.Javanica_Scaff1838g018741

MHLSSILIILPILFSSIQTQQIPNCQTRIFQCDADWDTRTDPNTGDVYGYKAFIQNKMNFYEADVFCRARCAEVVSIHSQEEQNFVRRIAAPVLNRCQFTSACTPVVQSTNATFDRQLRSFWIGLNRVTGTLKDAHSVDSSVYQLWSDRTEVDFGSFPNNSVNTGTNSPPWTRGNPNGVNSADPQIIEDCTQMVESRQGGWNDFPCNWRISGVICKRKCTSYCAAQDV*

>Mja|M.Javanica_Scaff18737g079362

MKLSVSFLNLLTLVFLLQCIHFVYVNGDCPAGWSSFTDSNGTEYGYQVVLKNNVSYFEARRLCLDINSEIVDIHSREENEYMYSLASNTSAQNLFIGMHYKLNETSRTSEVYCTWTPDVKCDFAYFNNQSDPRRQQYPWVPIPKAQNNGNPVECVEITDTSLGIGRPAFMYNDIACADRLDGAVCKITCSNGNNAATIFGTSTTLNGQNVATISTTGSGGNNAGPRPTPPTRTAATGAAGLPGSPGLDGLPGKGGNNAGPRPTPPTRTAATGAAGLPGSPGLDGLPGKGGNNAGPRPTPPTRTAATGAAGLPGSPGLDGLPGKGGNNAGPRPTPPTRTAATGAAGLPGSPGLDGLPGKGGNNAGPRPTPPTRTAATGAAGLP*

>Mja|M.Javanica_Scaff19252g080420

MKLSVFKTLILTYLSIQCIHFAFINADCPAGWNSRSDSSGIEYGYQVVLQKNITFSQAQSICSGLESDIVSIHSKEENEFIYGLIKTSGVVLYIGMQQKSDQYSPTCAWPDKSDCNFGNFNGVKDPKRQEYPCVRNPSITTDGNTVHCVGIADTTYNFGKPNFKWNDVGCNDGLDGTICKKKCIGGNSGGSASTSQTPEASKAPTILPSNTEAPVVSTKSSIGGGSSIGPVVTSKPSGAPVVSTKSAIGGGSTIGPVVTAQSPKTLATLLPGFPGSTPKPVNGGGNNGSCGNCNCNDGYKCGSDDWQVKHGRMDHSPTSYNFV*

>Mja|M.Javanica_Scaff21624g084775

MIQLCSLRTQLPHQRIQLCRLKTQLPHQRIQLCRLMTQLPHQRIQLHRLRTQLRRPKKQLHRLRIQLRRLMTQLPHQRIQLHRLMTQPHRLRTQLRRPKKQLPHQRIQLRRLKTQLPHQRIQLRRLKKQQNQRDTTPPPEDTTSPPEDTTPPPEETTESTSEGTVGAQTITDGTDATPVSDDTRTDLTTLIGTEGTTPTPNTSCPPDYTFFKEGNCCIQISSPEKKPLTWKEASEEAKKNGARMASIHFKKEDDFIRDLVAKQPKGEGKDNPVYFTGLQVNATNGKIGNFAWSDNTRVNYGSPKDIKPNTDPWGPKTSFNGNTGFGGISKDPSGKASWGLYDPNQKGGYVWKIDLSKKDGGTKVPEPGNPTGGGAPGGNNGLPAGYKCTTGCNPSSIKLKAEKGQPSPVDEGPTEKNGCQIDVLSCTTKQPIATITFGTQGSLKSNQRDTRNVRAKVICANEEATGKKGWVRNGVDNNPKLIFVDEAHCEQTS*

>Mja|M.Javanica_Scaff2283g021893

MSIKIKYFLNKIFLFLFFNFVKNSKYNNQEYLVSEKENNFCENNWEYWPERHLCIKIFNEKIGWNVAEINCLYNQGHQISVKNIKENKYLENIARRVGGLIWLGAAQFGQLNNYMWTDGTPFYFTFWQNGIRPLFNPGKKCIKMNSLTGEWIQSCCRVAAPFVCQKPAFKINKLRNEERKEEFIQR*

>Mja|M.Javanica_Scaff23130g087316

MRREKNFFQKISERENNFCENTWEYWPERHLCIKIFNEKIGWNVAEINCLYNQGHQISVKNIKENKYLEDIARRVGGLIWLGAAQFGNNYMWTDGTPFYFTFWQNGIQPLFNPGKKCIKMNSLSGEWIQSCCRVAAPFVCQKPAFKIDKKHLLRNEERKEEFIQR*

>Mja|M.Javanica_Scaff2356g022390

MIYLIFISFQKFCTILSIIFLINLKTSIGQTTSSLNFDNNYESPVNSNSNEEGGSNQCPDGWASRLDSSGIVYGYKVIMQDMINYYQARNLCNEEGGELVSIHSDEEEQFVAQLALNLLDQCQTNNSLCRQRVPIPSLDSMFRSFYIGLNRAAIEPYYDPTVVQTWADGSSVDYASVPTPGVPTIQIQPWGPGCPSGLNDTGSNPQKGSPEDCVCMYKGADGNINWNDISCYHKLAGGICKLTCNGGNFGKMCGINGWQAIATCQSLGAKVASIHSDLQQATVTNVSQTIQINCSWIGLHRNAILGGQIHNFWDDGSPSDYGVIVQLNGGNTPWSLGCPKSLGNTSCPTSNDCTAVVPPAGLWLDIDCVSTCGAVICEKECNPLTLTQNDLFFDELNSVEKEVVD*

>Mja|M.Javanica_Scaff2360g022416

MIAFSSAKLCSFIFSVAIFASYAILPTNGQATCPSDWISRVDPIDGITYGYKVITRDWLNYYEAQGYCVRAGGQVASIHSAAENVFIANISAAQLKQCQTNDSVCATRIAHTSAGWAFLDSLMRSFWFGMNRVQYQPSYNNTLDCSYPDGTPCDYGRFDGVANANTNSPPWSPACPSGSNSSNGAGDLEYCTMFFNATTTLLWNDMSCYQKLGGVVCKMNCSATKTTKTCPSSSVVTANAAATTKAAVVTSGSGSAGGSTAKSLTTKSSGTSNGSCSCNGGSCGGGGYKCGSDDWQWKINKKNGKSYAYKAFNYTGCYWQGLAICKKYKAVPCSIQSSDDNDFIVKTVCSGLLSSSSSNSSRKRRATSDTCIYTGIHRQVTNPSSGGVSCTCSDGSSCDYGNEDAHGDITTISFTSTTSRATTLKASTTQKAATTTTQKSTTKLATSPYISTSTAGPSNPWAPGCPANSTTGQRDNGGYGSRNCIGINGEGKWVDQSCDRPATAIICMKECDQP*

>Mja|M.Javanica_Scaff2360g022419

MSITSLIPLILFSLILPYAHCSCSSKWTTRVDSDGSTYGYLAIISDLITIFEGNTICKQQGGEVVSIHSAEENNFIGQIAGPLMKLCQETNACQQRVDHNNNRTAFLDMLYRSFWIGLSRVQFAHPLDNPTVHSAWLDGTPVDYGRYDGLPIPNRDVRPWAYGSPSSLNATTSGEIEGCTQMFLNDNDPQWNDISCYALLGGVICKRNCSADCPNEDNSTTTTSSTTTAVGLTTQTTEETTTEVQYDEATTEDIETSTPNSCGNNSNICGADGWKWKCFKNGKCYSYHRYSVKGSYWKALAKCRKHHASVCSIETEEENEYVCKHYCGCKGKDVWIDMHRMVTEYGTNIVCLDGEDKKCYYETDSNSRNPWANGCPADASNMDDGKYGKKNCCVISKGKWKDVGCTRPISKVLCKKECVKPR*

>Mja|M.Javanica_Scaff2407g022725

MLHQQQQQQQQLKPKFRLQLEDVGNPQPCPDNPIRLLASRTPQYIYSPYDENKLYPPDTDCQFLIEATDKFHRIHLTIIESDLEEALFTDCNDYVSVRDREGNETSIKEVARWCGQDYPAAIASASDSLLVHFHSDSIIQKRGFNISFVQFDIDTCPPDWISDGISSPYCYKQFVLPHILPWYEAQKECNFERANLATFQNEADYAFIVESYSQTHSFPWVGYSDANVEGIYESIDRNVPLWPENFPLKHENEMKDCVYLDWNKRDQIVVYEIDDCRNRRPFLCKKRRDGTEVPVILPAGMIRRGFRDFTMDYTLLVVVVIFALLALVVGCVLFHKYKERNNRIINIDMNQRLVQQQQGQQPGNKDKAAAALARQKAKERERRELREQKYAETSNNNSNRGGRGGGGIAATIKSPFESQDSNASFPLQTFQSTFAQRMTTTTTGSVEPLDTAAALAEASATASQQMREHEATKLHPQQLSAEIVSVDSPPEDDLNSSPRIRMSPNQPSFVEEGRKISEKKIRKGWMGAGVSGGGTTDREEEEEEKDRHNMGYEPDSEEEEHEENERQKIRRASKINGEGEGGEENFLMELNEEGGRKNNGENTGENTGENTGENEELEQYTQHNHMEEELEQYNGGEQFKQYKHKNVIESEQFKNSQQFKQLESDGGEVQQKVVQVELHEEIGEEGGLSQFKDKRTKEERITNFDEKMMDSYAIGDHFKAAKVATSAIASAAIAATEHPIRPPIELMRTTTRTTSTDKTKSPPPKEAKESEKGESGKSSREATREGKEETREEKETFPEKVSGKGNIPSSTATNIVKTEGSGTRIRIKRKTFDRPPPVGPLDNVSAISLDEFWQQQK*

>Mja|M.Javanica_Scaff2439g022953

XRIPHFWRQKFKFFQKFGNRKSKISQNFENRKSKIERKKTSKIENRNRKSKFRNSSGDDILKALNKETIENGEIEVKTGEDKIGDNKIRKWRKYVIKRKSNGGKNEEEKINEGERSLLDAPPVNEKSKGNLDNKEKNKIKNVLDWNKQRASVICGDEGWHYFDGFCYKLINGKFTWEEALGECKKQNSNLVSIQSEQENDFVGSLSTKFDQNFDNFCHGFWIGMTRKYIEETDSFKSEWSDGTLVNYGNVPIKIAFHTPPWMGGQPDFAGGVEECVHSYPKLGCDNWQQIFFNQWNDARCSIKLSGAVCKKRQSIKKFEKKK*

>Mja|M.Javanica_Scaff24410g089305

MLGTSIRCVVASLLVLQAYANKMDPAYQTDDDRNQPVESVPSEQAPWISGPQNQKYQFHIGKQSWLSAREKCLAQNADLVSIESIEEMQWLLSHYKPQFNHLRERQVQIGLLLDTIEGEAGSNSDSGLTSREWRWVNGKPLNLEITKWTMGEPFDHAKGKERCALLNINERRLDDVDCDLGSGAGFNYRFVCQRSHEKHIE*

>Mja|M.Javanica_Scaff25863g091543

MINLIFISFQKFCTILSIIFLINLKTSIGQTTSSLNLDYNYESPVNSNSNENGGLNQCPDGWASRLDSSGVVYGYKVIMQDMINYYQVFWFLEKNIFYVFSLNEIIHGLVKVIRLARNLCNKEGGELVSIHSDEEEQFVAQLASSLLDQCQTNNSLCRQRVPIPSLDSMFRSFYIGLNRAAIEPYYDPTVVQTWADGSSVDYASVPTPGVPTIQIQPWGPGCPSGLNDTGSNLQKGSPEDCVCMYKGADGNINWNDISCYHKLAGGICKRTCNGARFGKMCGINGWQAIAICQSVGAKVASIHSDLQQATVTNVSQTIQTNCSWIGLHRNAILGGQINNFWDDGSPSDYGVIVQLNGGNTPWSLGCPKSLGNTSCPTSNDCNAVVPPAGLWLDLDCVSTCGAVICEKECNPLTLTQNDLFFDELNSVEKEVVD*

>Mja|M.Javanica_Scaff26996g093142

MKKRESYINEEENNGKTKLSSNSIEKRLDRLEANQQNLRRATLADWPAFSVDKRVRIFDHTRSTWIDAQRECSEHAGTLLEIDSESENERINEMLTNSGNSNRPNDNYWIGVQIMLQFSNGSSIIGNYSNFEEQNSNSENTENQKLSKGLVMKRCAAISASQTTKSEGRWLSLECSEKHGFICQL*

>Mja|M.Javanica_Scaff270g004217

MKNIHTGHKRPRNRKIKLYNSYFNLLINFFVITTLFVNINGTESDESSTSVDDEEPVFSQSAQHLFKNENSNEDSGKHSSLEASSPSKNHRQHRKNPPPFVLADLQLSCAEGWERFEGKCYKLVSIEKSWPQALAFCSRFGAKLVRLESSEENKFLAKYLMRPHLTSGPTNSPSEYWIGLLYRPLMMDSSTSSSSSHSKSLMDGSFLWSDGSQTSRYVGFWSYGQPDPANGSCTKILVDGLQVEGPTWQLDVCNQLRPFICEQNACIKGSFFCQNGVCLPERAHCNGIDECGDSSDEFNCPSAHSEMSCQRYEKGESGRIETPNFPASYRQGSNCRWVIEGPLTSKIQLNFDSFETEERHDLVTVLDGGPSENSTFALSTISGTPRNTEKLSFESSTNRMIVQFRADQSIQARGFQASWRTVPISCGNQQLKASSIDQQFHSPEWPRNYPKGLECVWRIEAPSGQLISLSIDEFNTEAETDFLTIYDGPSPSEPILAKFSGQMKEPQLIISTQSQVHIYFFSSETVSQKGFTITYKKGCDNSIRRSHGVLTSPGNAHLPYAPSQICRWSIELPSQQIENFEAAAEIPSLSLVLNSWDVADLGDKLQIYEGGEDGSSNGRPLHESDGFTVNNAPPKTIYAKQGRVELVWRSNVLNSGTGWNISFSTSCPPLSLPSRRVLLSTKNTAYGTHVTISCERGFEFSTGLGRHFETECELGGLWSNYAPVPDCQPVYCSAIPQIANGFAVSATNVSYAGMARYQCYEGFSFASGKQHEEIFCTDEGRWTQAPKCKTDACPALPSFVSGERILQFGDGIGFGSVFQFHCAKGYFIEGPLSIVCRPNGEWSSPQPLCKKLTCTDIPIVENGELHLLPSNNMAMDEKSNKDKKPKAQTKNFGSRQKMEFKTKRNTVLRELQFGDSLRVECHSGFQSVGAETLKCLANQTLSGIPKCRDIDECELQSAGNCAGKSTTCVNMPGGFHCQCQSGYKPKLDCSGPLTVIPTKIQTSHGVPIPVQQLNSKNGWCADTTITNAQMLEKRIMPFNESITSSSPSTILTLIFTFPVPKIIEKLHLEKVVVPNASAPSGIAGAIAATPEAWPQRFTLSYSTEEGMPFEVYNGGLAELGGNNNITSTNEKKKSNERKLNTPNTKEIRTRALGAIGSEILVLAKPIEARTLQIEFLEFHGGVPCMKFEFLGCQRTSCEDINECEDGRNGGCEQHCHNTQGGHRCSCEEGFDLFVEPGQSGVRLREGETGYGEFDSLRFNHSCIPRHCAPLMTPGNGQLIAQNLYKNFKNEVNNTNNKNEFFLSSFAFPSIVEFRCTFGYQMRGPSHLKCLADGTWNGTVPSCIPASCSGVKNSTAVGLFVQPETVSIPFSQNLSFVCSQTNRPPKHSALGEIRQCIYDPRTDGLEYWLSGPEVDCPLVDCGPPPALSGAYYEGDEGHHGGNFKVGSVYLFQCRAPYSLVGKSSYDDRMVRCNVDGTWDLGDLRCEGPVCVDPGHPDDGQTFLDSVEEGAVASFSCNRPGFKPFPAETISCSLGTPCVLSEDVGISSGFIPDGAFSDNSDKVIWGYEPHKSRMSSSGWCGSKDAFIFLSVDLQRIYTLTTLRLTGVAGNGHLSGHVTKMQLFYKVQFSQNYDNYPMEFSTPSGNHRKIYQFTLNPPLRARYILLGITEYEKNPCLRFDMHGCLAPLSTTHEVPAHLQVGWNASIPQCLDAEPPTFKNCPQSPIIVQTDENGQLFPANYVIPEATDNSGRITYMLTKPEDFHPPYPVSQDTDIIYQAFDDAGNMAECPVRLRIPDTVPPILKCPDSYAIWAQENQTELHMHFNESSVRLVVQDQSPITQISYDPPEARIKLDSHVTVEASVLDAHSNRNKCKFQVALLPEPCSPWSLRIDEATVQKQCQRHASGTVCQVQCRKGYRFLESFPQQKSEVKKQQNSTNTLPQRYSCSMEQQSGKWLPSPTPPACVPMAMEPARYEMRVHMNYSLTSPLPSDCAKSYELLVGSLFDSIDQVLSQRCSSTVQIYVRFLDAKFSQMGEKTMGANFTVQILPTVLQQVFYELCSLTLRTIFDLRIPGATIPIRSLLTLSGDSVPAILNLGCPPINASSISVSQGFSCSQGELLKLQTPNVGSDDFLSAAPGLPECFPCPKGTAFVNNSCIQCPMGSFQDQEGQIRCKPCIENTYTLQSGAQSNESCLDVCGNGMFSATGMIPCQLCPRHTFAGPPPIGGYKECEPCPEGTYTARLGSVGPSHCKQPCAPGHFSVTGLEPCSPCPINFYQPNIGQQRCLQCSNDSFTAETGRSADEHCKKLDCQTLKCQNRGQCVVANHKEVCECRPGFMGSHCEQQIPLCDSHPCLNGGTCELHNGAFRCICPQNYTDSRCQFGPDECISSVHCPNGGVCQDLPGLGTTKCICRTGFTGPDCSQISDPCQSDQPCKNGAQCIPLQLGRYKCKCLPGWEGTNCDKNIDDCTENPCALGAKCHDLINDFECECPHGFSGKRCQIKDNLCDPSPCLNGGQCVDTLFDRHCICKRGWNGTFCEQEVNECSQKPCQNGATCRDQEDDYSCECAPGFHGYQCQYMIDHCAVKPCRNNGTCINRGPIYECQCPLGYEGDHCEHNVDECEMMTPCDAVGTGRCEDLVNGFKCHCHPGYEGTFCEQHVSQCEEEPCMNNGTCTDLGAGFQCECQLGWKGDRCQEMETQCDRKPCMNEGKCVPLVDDYFCVCPEGVSGKNCELAPNRCLGEPCHNGGVCGDFGSRSDCQCPKGYSGNGCQFRFDGCHEGLCKNGGTCVNNDDLSKHQMSKIENAVAVEQNSEAIGFKCICAPGFSGNECEIDINECQPSPCPLASHCVDLVNGYYCKCPFNMTGANCEKRIDPDYDLRFLESSSQPASASLGIPFNFVSSALTLNIWVKFEKNHQTDVLKEQRKSPPIFFTLYGSSSANQPTNLTELLTISSEAINIRLFPELDKQPLVLHFPIHQRPDTQLAWNNIIFMWDSQQQGSYSLLWNAVRLYSDKGYAPDRRLDINAWINLGDPKGIEQQQNQNLMTNKIISTELPKVNEKIIGTKFVGSITRVNMWNRMLDFETEIPSIVQRCQGSPDLYEGLSLRFANYDKLQGKVERIAKSTCGRINLLSPTCHSSGDENKNLKDHQCRSRIPSNMNDYFKEELVEVEGCPMEPINVQTPLKELNISWKEPKFYETSSRVQIAKIEQNLKPGQVFTWGQYSAIYLALDNQSSPLATCQFKINVVREFCPDPEIPVNGVQRCEQWGPGLRYKACSVHCQNGYGFSRPPAAFYSCGEDGKWRPNEGKQRPFRYPQCTRQSPAEHLARIQLSYPQLALCNPAGKSTVTEKIIERINQSNNKWHICANNEESSDCSGVQVQINCQDIFAASRLKRQSTLQQFDVIIDVPIREIQRMDPVEIIRDEAMAHGLFSLEQVIPNGRPDIGAFRVENAFRCPIGYLLNNGSCVPCAPGTFYFVPTSECKLCPIGQYQPEEAQNQCIECPTDSPMTVGMGSIKQNECRIRCLPGHHLNISTGQCEPCSYGFFQPDSGAFDCIPCGIGKTTLERTAINEDQCRDECPDGQQLTASGSCQPCPQGMYRTRGQDKQCVECPSGTTTEGVGAGNKALCNTPKCGAGQFLLADIKRCQFCPRGTFQDQQLQFECKKCPPSFNTAEEGATRESQCYSTDQCALGQDNCSWNAQCIDLPDDNDVASWRCVCNPGFRGNGINCTDACLNFCLNDGVCRKNKLGHVECSCKENFSGERCEIRFQPRSQKLVYWTGAIVAVVFLLIVIVVVIWMISLRFSRSSDNSFLSSPLDKPALSFTQSTTDSPLASNFLYGRPPPLLGRSHSSSLGGGGSNSIIHPIGFYYEDEQSPYDGGVGSAVGRHGRENSQEVYTFFTCVQKARQSFKDEVHFSG*

>Mja|M.Javanica_Scaff27530g093864

MKLNFVFLISLFIFLFFSYTTEAYFGCNCQYPNNHYVPWYLSGNVENRLTFLERQTSQKINMLIARIEALERELGLIHRISMQEWNNSGAGNIYKIFNTSKTWEEAKNTCMTFGARLASIDSDYKNAFVRNMIERSFGKDGSAEVWIGLKTRAELTNNPNSHFTNFGEEEKIDGCAVMGIKGKWKIRSCSNLKTFCL*

>Mja|M.Javanica_Scaff2778g025155

MIAFSSAKLCLFIFSVALIAIYCILPTNGQATCPSDWISRVDPIDGITYGYKVITRDWLNYYEAQGYCVRAGGQVASIHSAAENAFIANISAAQLKQCQTNDSVCATRIAHTSAGWAFLDSLMRSFWFGMNRVQYQPSYNNTLDCSYPDGTPCDYGRFDGVANANTNSPPWSPACPSGSNSSNGAGDLEYCTMFFNATTTLVWNDMSCYQKLGGVVCKMNCSATKTTQTCASPSVVTTNVAVTTKAAVVTSGSGSAGGSTAKSLTTKSSGTSNGSCSCNGGSCGGGGYKCGSDDWQWKINKKNGKSYAYKAFNYTGCYWQGLAICKKYKAVPCSIQSNDDNDFIVKTVCSGFLSSSSSNSSRKRRATSDTCIYTGIHRQVTNPSSGGVSCTCSDGSSCDYGNDDAHGDTTTTSFTSTTSRATTLKTSTTQKAATTTQKSTTKLATSFISTSTASPSSPWAPGCPANSTTGQRDNGGYGSRNCIGINGDGKWVDQSCDRPATAIICMKECDQP*

>Mja|M.Javanica_Scaff2778g025158

MSITSLIPLILFLLILPYYAHCSCSSKWTTRVDSDGSTYGYLAIISDLITIFEGNTICKQQGGEVVSIHSPEENNFIGQIAGPLMKLCQETNACQQRVDHNNNRTAFLDMLYRSFWIGLSRVQFAHPLDNPTVHSAWLDGTPVDYGRYDGLPIPNRDVRPWAYGSPSSLNATTSGEIEGCTQMFLNDNDPQWNDISCYSLLGGVICKRNCSANCPNEDNSTTTTSSTTTAVGLTTQTTEETTTEVQYDEATTEDIETSTPNSCGNNSNICGADGWKWKCFNNGKCYSYHKYSVKGSYWKALAKCRKHHASVCSIETEEENEHVCKHYCGCNGKDVWIDMHRMVTEDGTDIVCLDGEDNKCYYETGLTYGRQALEALTRELLKDSTSKNPWANGCPANASSMDDGNYGKKNCCVISKGKWKDVGCTRPISKVLCKKECVKP*

>Mja|M.Javanica_Scaff29028g095842

MFPNWALLLPIFGLFAYSSAQSRCNFICEADWQTRVEPTDGKTYGYKVFFKSALNFFEAQRICRDNCAEVATIHSRDEYDFVFLTFRDLLDQCQTNASVCPIRDPNPDFNRISNSFWLGMHRIQQYTSICPGCSVDSEIVYVNSDGTPYNFGTYNADRAVIEDCTQLLDSATGKWNDIPCHYQQAGVVCKRDCASFCQAQVPPITTTEATTTISSPGGQPGGPGQPGPNGQNGADATTSAPGGGDNPPGGRNNFV*

>Mja|M.Javanica_Scaff2989g026481

MKNIHTGHKRPRNRKIKLYNSYFNLLINIFVITTLFVNINGTESDESSTSVDDEEPVFSQSAQHLFKNENSNEDSGKHPSLEASSPSKNHRQHRKNPPPFVLADVQLSCAEGWERFEGKCYKLVSIEKSWPQALAFCSRFGAKLVRLESSEENKFLAKYLMRPHLTSGPTTSPSEYWIGLLYRPLMMDSSISSSSSHSKSLMDGSFLWSDGSQTSRYVGFWSYGQPDPANGSCTKVRILVDGLQVEGPTWQLDVCNQLRPFICEQNACIKGSFFCQNGVCLPERAHCNGIDECGDSSDEFNCPSAHSEMSCQRYEKGESGRIETPNFPASYRQGSNCRWVIEGPLNSKIQLNFDSFETEERHDLVTVLDGGPSENSTFALSTISGTPRNTEKLSFESSTNRMIVQFRADQSIQARGFQASWRTVPISCGNQQLKASSIGQQFHSPEWPRNYPKGLECVWRIEAHSGQLISLSIDEFNTEAETDFLTIYDGPSPSEPILAKFSGQMKEPQLIISTQSQVHIYFFSSETVPQKGFTITYKKGCDNSIRRSHGVLTSPGNAHLPYAPSQICRWSIELPSQQIENFEAAAEIPSLSLVLNSWDVADLGDKLQIYEGGEDGSSNGRPLHESDGFTVNNAPPKTIYAKQGRVELVWRSNVLNSGTGWNISFSTSCPPLSLPSRRVLLSTKNTAYGTHVTISCERGFEFSTGLGRHFETECELGGLWSNYAPVPDCQPVYCSAIPQIANGFAVSATNVSYAGMARYQCYEGFSFASGKQHEEIFCTDEGRWTQAPKCKTDACPALPSFVSGERILQFGDGIGFGSVFQFHCAKGYFIEGPLSIVCRPNGEWSSPQPLCKKLTCTDIPIVENGELHLLPSNNMAMDEKSNKDKKPKDQTKNFGSRQKMEFKTKRNTVLRELQFGDSLRVECHSGFQSVGAETLKCLANQTLSGIPKCRDIDECELQSAGNCAGKSTTCIPKCRDIDECELQSAGNCAGKSTTCVNMPGGFHCQCQSGYKPKLDCSGPLTVIPTKIQTSHGVPIPVQQLNSKNGWCADTTITNAQMLEKRIMPFNESTTSSSPSTILTLIFTFPVPKIIEKLHLEKVVVPNASAPSGIAGAIAATPEAWPQRFTLSYSTEEGMPFEVYNGGLAEIGGNNNITSSNENKKSNERKLNTPNTKEIRTRALGAIGSEILVLAKPIEARTLQIEFLEFHGGVPCMKFEFLGCQRTSCEDINECEDGRNGGCEQHCHNTQGGHRCSCEEGFDLFVEPGQSGVRLRECETGYGEFDSLRFNHSCIPRHCAPLMTPENGQLIAQNLYKNFKNEVNNTNNKNEFFLSSFAFPSIVEFRCTFGYQMRGPSHLKCLADGTWNGTVPSCIPATCSGVKNSTAVGLFVQPETVSIPFGQNLSFVCSQTNRPPKHSALGEIRQCIYDPRTDGLEYWLSGPEVDCPLVDCGPPPALSGAYYEGDEGHHGGNFKVGSVYLFQCRAPYSLVGKSSYDDRMVRCNVDGTWDLGDLRCEGPVCVDPGHPDDGQTFLDSVEEGAVASFSCNRPGFKPFPAETISCSLGTPCVLSEDVGISSGFIPDGAFSDNSDKVIWGYEPHKSRMSSSGWCGSKDAFIFLSVDLQRIYTLTTLRLTGVAGNGHLSGHVTKMQLFYKVQFSQNYDNYPMEFSTPSGNHRKIYQFTLNPPLRARYILLGITEYEKNPCLRFDMHGCLAPLSTTHEVPAHLQVGWNASIPQCLDAEPPTFKNCPQSPIIVQTDENGQLFPANYVIPEATDNSGRITYMLTKPEDFHPPYPVSQDTDIIYQAFDDAGNMAECPVRLRIPDTVPPILKCPDSYAIWAQENQTELHMHFNESSVRLVVQDQSPITQISYDPPEARIKLDSHVTVRYCIF*

>Mja|M.Javanica_Scaff3056g026876

XTLKFNFKIFSHLNFKNKDYPAAIASASDSLLVHFHSDSIIQKRGFNISFVQFGILESSKSPQNLKIIFLDIDTCPPDWISDGISSPYCYKQFVLPHILPWYEAQKECNFERANLATFQNEADYAFIVESYSQTHSFPWVGYSDANVEGIYESIDRNVPLWPENFPLKHENEMKDCVYLDWNKRDQIVVYEIDDCRNRRPFLCKKRRDGTEVPVILPAGMIRRGFRDFTMDYTLLVVVVIFALLALVVGCVLFHKYKERNNRIINIDMNQRLVQQQQGQQPGNKDKAAAALARQKAKERERRELREQKYANTSNNNSNRGGGRGGGGGVATTKSPFESQDSNASFPLQTFQSTFAQRMTTTTTGSVEPLDTAAALAEASATASQQMREHEATKLHPQQLSAEIVSVDSPPEDDLNSSPRIRMSPNQPSFVVEEGRKISEKKIRKGWMGAGVSGGGTTDREEEEEEKDRHNMGYEPDSEEEEHEENERQKIRRASKINGEGEEGEENFLMELNKEGGRKNTGENTGENNGENEELEQYTQHNHMEEELEQYRGGEQFKQYKHKNVIESEQYVNSQQFKQLESDGGEVQQKVVQVELHGENGEEGGLSQFKDKRTKEERINNFDEKMMDSYAIGDHFKAAKVATSAIASAAIAATEHPIRPPIELMRTTRTTSTDKNKTPPPPKEVKESEKIGESGKSSREETREGKEETREEKETFPEKVSGKGNIPSSTATNIVKTEGEGGTRIRIKRKTFDRPPPVGPLDNVSAISLDEFWQQQK*

>Mja|M.Javanica_Scaff3225g027923

MMLVILTSVDDEEPVFSQSAQHLFKNENSNEDAEKHPSLEASSPSKNHRQHRKNPPPSVLADVQLSCAEGWERFEGKCYKLVSIEKSWPQALAFCSRFGAKLVRLESSEENKFLAKYLMRPHLTSGSTSSPSEYWIGLLYRPLMMDSSISSSSSHSKSLMDGSFLWSDGSQTSRYIGFWSYGQPDPANGSCTKILVDGMQVEGPTWQLDVCNQLRPFICEQNACIKGSFFCQNGACLSERAHCNGIDECGDSSDEFNCPSAHSEMSCQRYEKGESGRIETPNFPASYRQGANCRWVIEGPLNSKIQLNFDSFETEERHDLVTVLDGGPSENSTFALSTISGTPRNTEKLSFESSTNRMIVQFRADQSIQARGFQASWRTVPISCGNQMLKASSIGQQFHSPEWPRNYPKGLECVWRIEAPSGQLISLFIDEFNTEAETDFLTIYDGPSPSEPILAKFSGQMKEPQLIISTQSQVHIYFFSSETVSQKGFTITYKKGCDNSIRRSHGVLTSPGNAHLPYAPSQICRWSIELPSQQIENFEAAAEIPSLSLVLNSWDVADLGDKLQIYEGGEDGSSNGRPLHDGDGFTVNNAPSKTIYAKQGRVELVWRSNVLNSGTGWNISFSTSCPPLSLPSRRVLLSTKNTAYGTHVTISCERGFEFSTGLGRHFETECELGGLWSNYAPVPDCQPVYCSAIPQIANGFAVSATNVSYAGMARYQCYEGFSFASGKQHEEIFCTDEGRWTQSPKCKTDACPALPSFISGERILQFGDGIGFGSVFQFHCAKGYFIEGPLSIVCRPNGEWSSPQPICKKLTCTDIPVVENGELHLLPSNNMVMDEKSNKDKKLKDQTKNFGSKQKMEIKTKRNTVIRELQFGDSLRVECHSGFQSVGAETLKCLANQTLSGIPKCRDIDECELQSAGNCAAKSTTCVNMPGGFHCQCQSGYKPKLDCSGPLTIIPTRIQTSHGVPIPVQQLNSKNGWCADSAITNAQILEKRIMPFNESITSSSPSTILTLIFTFPVPKIIEKLHLEKVVVPNASAPSGIAGAIAATPEAWPQRFTLSYSIEEGMPFEVYNGGLAEIGGNNNITSTNENNKKSNERKLNTPNTKEIRTRALGAIGSEILVLAKPIEARTLQIEFLEFHGGVPCMKFEFLGCQRTSCEDINECEDGRNGGCEQHCHNTQGGHRCRCEEGFDLFVEPGQSGVRLREGETGYGEFDSLRFNHSCIPRHCAPLTTPENGQLIAQNLYKTFKNEVNNTINKNEFFSSSFAFPSIVEFRCTFGYQMRGPSHLKCLADGTWNGTVPSCIPATCSGVKNSTAVGLFVQPETVSIPFGQNLSFVCSQTNRPPKHSALGEIRQCIYDPRTDGLEYWLSGPEVDCPLVDCGPPPALSGAYYEGDEGHHGGNFKVGSVYLFQGGSLVEGPYPFERHILADCTFRVNTCRAPYSLVGKSSYDDRMVRCNVDGTWDLGDLRCEGVLNFFYLYSQNIFSGPVCVDPGHPDDGQTFLDSVEEGAVASFSCNRPGFKPFPAETISCSLGTPCVLSEDVGISSGFIPDGAFSDNSDKVIWGYEPHKSRMSSSGWCGSKDAFIFLSVDLQRIYTLTTLRLTGVAGNGHLSGHVTKMQLFYKVQFSQNYDNYPMEFSTPSGNHRKIYQFTLNPPLRARYILLGITEYEKNPCLRFDMHGCLAPLFGWNASIPQCLDAEPPTFKNCPQSPIIVQTDENGQLFPANYVIPEATDNSGRITYMLTKPEDFHPPYPVSQDTDIIYQAFDDAGNMAECAVRLRIPDTVPPILKCPDSYAIWAQENQTELHMHFNESSVRLVVQDQSPITQISYDPPEARIKLDSHVTVEASVLDAHSNRNKCKFQVALLPEPPEDFHPPYPVSQDTDIIYQAFDDAGNMAECAVRLRIPDTVPPILKCPDSYAIWAQENQTELHMHFNESSVRLVVQDQSPITQISYDPPEARIKLDSHVTVEASVLDAHSNRNKCKFQVALLPEPCSPWSLRIDEATVQKQCQRHASGTVCQVQCRKGYRFLESFPQQKSEVKKQQNSTNTLPQRYSCSMEQQSGKWLPSPTPPACVPMAMEPARYEMRVHMNYSLTSPLPSDCAKSYELLVGSLFDSIDQVLSQRCSSTVQIYVRFLDAKFSQMGEKTMGANFTVQILPTVLQQVFYELCSLTLRTIFDLRIPGATIPIRSLLTLSGDSVPAIL*

>Mja|M.Javanica_Scaff3387g028847

MIILSFVSLFYLSLPALIAGQCSSDWQTNVGSDGFTYGYKAVVSDMVNFFEAHDVCAALGAQVASIHSAQENAFVISIASSLLDQCENNNAVCKQRVDRSTTSSLRLDRLLRSFWIGMHRVQYAPFYNTSVDCCWSDGTQCDYGRFDGVADPNQNIPPWSTGNPSGSSSSITSAGWNVEDCVEITNDNGVLWNDIGCYHKLGGVVCKKNCTNTCPPVATQAPAVTSKPITTIPVVTTTTDDETGGYICGSDEWQWRNNGNGKAYAYKWFNQSGSFWQLHDLCYDSCATPCSIESEEENQHIVNHVCAGAFSSSSSGRKKRATDSQCIYTGFHRYQESPPNGPIGCSCDDGKSCDYGNDDTTTTTSTTSTTKAATTTAKAITTPRPPTSTIRATTKATTIITSTTKPNNPWAPGCPANSTTGLGDSGGYGTKNCVGFDSNGKWRDQACERPGKGIVCKKPCIPPAGS*

>Mja|M.Javanica_Scaff348g005187

MTLIGLIIAFCIIPSAVNADCLSGNWTTRVATDGNTYGYQVLARDWLNFYEARALCLGVGGDVASIHSNAENEFVRQLAAPYIIGCQTNKTTCGARATTSLDTILSVVWLGMHRCQYFPSYNATVDCVNSDGTPCDYAATNAVILTGPSPFPWAWGNPSGSDSGGGAGLIEDCVSMYNATSGEWNDLSCFQKLGGVVCKRNCTGTCGSSSIKTALITSPSDITDTSCTSGNWTKRAGEDGNTYGYQIVMQDWLNFYEVKSKKYTRHLIKFFILKANAKCLALGAEVVSVHSVAENEFIRQLSAPYITACQTNTSVCATIFSSLWVGLHRSAFYPSYNSTVDCINSDGTICDYFNITGGPSGTETGGASGQQEACAAMYSATTGQWNDIACFNKLGGVICKKNCSNACGVASTTTTQLTTTTTQPTTTTTLPTTTTTLPTTTTTLPTTTTTEPSTTTTAEPTTTTTEPSTTTTAEPTTTTTEPSTTTTFNCAQPPVTSLLSYNTSDPKIQTGSNATQGVCECPADPGNSNVFFIPVTTITTGSSASNSSVIMKCSKMQDFCICDEDDVCWKVINAYSLVVINSFCDPTCHNYARLQNAAPFNQTFESECGRTITLADELTPIPNTNRNTFKPLGSSADYYIKAASIRCLQAGQTCTPIKCSGTRKPSPCPTTTTTLPTTTTTLPTTTTTLPTTTTTLPTTTTTLPTTTTTLPTTTTTLPTTTTTLPTTTTTLPTTTTTLPTTTTKLQTTTTTLPTTTLAPLNCGNCATGQSRVIYDKNTLASNQIVKNNAASQCIFNCKDPDVSQICYTIDAVSVARINCTDPSQFCVCASNVKKGCYTVTNTALLADYSIFANASYTFLVINSGSSSIANSQTGASYKFTAGPSFVVGNSKTLNAPVLGISCNGCAPIHNPGADVIKPCTPGGG*

>Mja|M.Javanica_Scaff3646g030333

MNIRPPNPPTSTPNTSCPPDYVFFKEGNCCIQISSPEKKPLTWKEASEEAKKNGARMASIHFKKEDDFIRDLVAKQPKGEGKDNPVYFTGLNVNATNGKINNFAWSDNTRVNYGSLKDIKPNTDPWGPKITFNGNTGFGGISKDPSGKASWGLYDPNQKGGYVWKIDLSKKVPEPGNPTGGGGTGGNNGLPAGYKCTTGCNPSSIKLKAEKGQPSPVDEGPTEKNGCQIDVLSCTTKQPIATITEATGKKGWVRNGVDNNPKLFFVDEAHCEQTS*

>Mja|M.Javanica_Scaff385g005629

MDPAYQTDDDRNQPVESVSSEQAPWISGPQNQKYQFHIGKQSWLSAREKCLAQNADLVSIGSIEEMQWLLSHYKPQFNHLRERQVQIGLLLDTIEGEAGSNSDSGLTSREWRWVNGKPLNLEITKWTMGEPFDHAKGKERCALLNINERRLDDVDCDLGSGAGFNYRFVCQRSHEKHIEHESLNNPLWKKLEDILTFFGISDREDDKKNCSLPIGAKEEGYWERAFNKGEGNSETQHKEKTIEKTDSITEKSELENPKGVEKNSKTNKEDRDSVLEDKTIIEKKVITNKNDETTNKLSSPPEQKLVKDVEKVVEEKKVSKVGGVTQHATSKIIGGEDIQSPNELENTRPTGGAVVLRRVESHVSASSNVNNKKNNQEKLKEGNEEAKSTKLEEEQSKILKVAGTEEANLRDKEASDSSDSMREQSGLSVREKSQIINLNTRENLQSSLNPKKPLIISNDPSDLTEDRNTISNKNTSDNEKENKSIQNLSESVQITEKKLGEDEISEIKQYTLENKYIVNTPKVDKKNNEKIKEDKLNAEKIGEEKISEKEEKKEEKNEDKIKNGDKDDRKGENEKITVVDERKTKKENKEIKEIEENNKEDKEVKRDGERKEENEKKSDELKKDEVTRGEEIKDEKKVIKEKDIKEEKLKSEETKTRKEKHDEKEEKEDRKNLLPKSNNIRDESKKLDLEDKEEKLEANLIKNKINNEITDVKKNEEVLAQNKVEEKIREEQSLEFRNKDKKDDNKSVKKSENVEAIQEKTAEIKEAKQDSNQESKKANNEAKDGLNNETTHELKNEATNSKNNEVSQETKKEAKLKENKEGKDEAKHEAKLEAKHKTKIEATEANEKESVNGAKSEAQSEARHEANKVSSSNKGTNNEAKSKENNEAETNINEDKTIKTNKPTKHPDPLSVVAETLERSLSSKEETIEKKKDEAENLIKERRQKPDGVRDRIQHLEKIITEVQHMMGIGEKGGEGELIEKTNKVLEEDEEEKKYEIKKEEGRKNMKEVKKEKEEKIRVEEKEESKEGSGSSEKLTEATPIAEVEELSSPEVEPKSHSPSVSSSDESEESEDSPKHVKTKNIKDNKAQREQPVNVNNEATIKSGTVNKSHSIISDSLDEQRKSMAAMEFDFEEAALQAKAHPGINSGDLVKNIILIGKDEELDGCKEGKECKDKKAKSKQNRRYAPRNEEKWEIINKMSDDINAKKGSRIEKYGRKSVGNKKRFEGNVMEEREGEELNLVKNNHDQGLLNSIHSETIEATPTGHILDQLIIFHPNDGKRSRESQIISQMLLPDGGFHGKVTGDGKIEHRPADDDWVIPEVETQTMLLNSLNSSSNSSHSSTPLSIFPAPDFSIIKPGDQVPPQKIIQRLEEERKRIDAVYEKLNKGDNIYDLIKRIKASDARRQPLTVLQSKMTPN*

>Mja|M.Javanica_Scaff4147g033090

MLHQQQQQQQQKPKFRLQLEDVGNPQPCPDNPIRLLASRTPQYIYSPYDENKLYPPDTDCQFLIEATDKFHRIHLTIIESDLEEALFTDCNDYVSVRDREGNETSIKEVARWCGQDYPAAIASASDSLLVHFHSDSIIQKRGFNISFVQFDIDTCPPDWISDGISSPYCYKQFVLPHILPWYEAQKECNFERANLATFQNEADYAFIVESYSQTHSFPWVGYSDANVEGIYESIDRNVPLWPENFPLKHENEMKDCVYLDWNKRDQIVVYEIDDCRNRRPFLCKKRRDGTEVPVILPAGMIRRGFRDFTMDYTLLVVVVIFALLALVVGCVLFHKYKERNNQIINIDMNQRLVQQQQGQQPGNKDKAAAALARQKVKERERRELREQKYAETSNNNSNRGGGRGGGGGIAATTKSPFESQDSNASFPLQTFQSTFAQRMTSTTTGNVEPLDTAAALAEASATASQQMREHEATKLHPQQLSAEIVSVDSPPEDDLNSSPRIRMSPNQPSFVEEGRKISEKKIRKGWMGAGVSGGGTTDREEEEEEKDRHNMGYEPDSEEEEHEENERQKIRRASKINGEGEGGEENFLMELNDEGGRKNTGENNGENIGENEELEQYTQHNHMEEELEQYSGGGEQFKQYKHKNVIESEQYINSQQFKQLESDDGVQQTVVQVELHGENGEEGGLGQFKDKRTKEERINNFDEKMMDSYAIGDHFKAAKVATSAIASAAAIAATEHPIRPPIELMRTRTTSTDKAKSPPPPKEAKESEKIGESGKSSREATREGKEETREEKETFPEKVSGKVNISSSTATNIVKTEGEGTGTRIRIKRKTFDRPPPVGPLDNVSAISLDEFWQQQK*

>Mja|M.Javanica_Scaff4530g035059

MMLVILTSVDDEEPVFSQSAQHLFKNENSNEDAEKHPSLEASSPSKNHRQHRKNPPPFVLADVQLSCAEGWERFEGKCYKLVSIEKSWPQALAFCSRFGAKLVRLESSEENKFLAKYLMRPHLTSGPTTSPSEYWIGLLYRPLMMDSSISSSSSHSKSLMDGSFLWSDGSQTSRYVGFWSYGQPDPANGSCTKILVDGLQVEGPTWQLDVCNQLRPFTCEQNACIKGSFFCQNGVCLPERAHCNGIDECGDSSDEFNCPSAHSEMSCQRYEKGESGRIETPNFPASYRQGTNCRWVIEGPLNSKIQLNFDSFETEERHDLVTVLDGGPSENSTFALSTISGTPRNTEKLSFESSTNRMIVQFRADQSIQARGFQASWRTVPISCGNQQLKASSIGQQFHSPEWPRNYPKGLECVWRIEAPSGQLISLFIDEFNTEAETDFLTIYDGPSPSEPILAKFSGHMKEPQLIISTQSQVHIYFFSSETVSQKGFTITYKKGCDNSIRRLHGVLTSPGNAHLPYAPSQICRWSIELPSQQIENFEAAAEIPSLFLVLNSWDVADLGDKLQIYEGGEDGSSNGRPLHESDGFTVNNAPPKTIYAKQGRVELVWRSNVLNSGTGWNISFSTSCPPLSLPSRRVLLSTKNTAYGTHVTISCERGFEFSTGLGRHFETECELGGLWSNYAPVPDCQPVYCSAIPQIANGFAVSATNVSYAGMARYQCYEGFSFASGKQHEEIFCTDEGRWTQSPKCKTDACPALPSFVSGERILQFGDGIGFGSVFQFHCAKGYFIEGPLSIVCRPNGEWSSPQPLCKKLTCTDIPVVENGELHLLPSNNMVMDEKSNKDKKLKDQTKNFGSKQKMEFKTKRNMVVRELQFGDSLRVECHSGFQSVGAETLKCLANQTLSGIPKCRDIDECELQSAGNCAAKSTTCVNMPGGFHCQCQSGYKPKLDCSGPLTIIPTKILTSHGVPIPVQTLNSKNGWCADSAITNAQMLEKRIMPFNESITSSFPSTILTLIFTFPVPKIIEKLHLEKVVVPNAVAPSGIAGAIAATPEAWPQRFTLSYSIEEGMPFEVYNGGLAELGGNNNITSTNENKKSNEKKLNIPNTKEIRTRALGAIGSEILVLAKPIEARTLQIEFLEFHGGVPCMKFEFLGCQRTSCEDINECEDGRNGGCEQHCHNTQGGHRCSCEEGFDLFVEPGQSGVRLREGETGYGEFDSLRFNHSCIPRHCAPLMTPENGQLIAQNLYKNFKTEANNTNNKSEFFSSSFAFPSIVEFRCTFGYQMRGPSHLKCLADGTWNGTVPSCIPASCSGVKNSTAVGLFVQPETVSIPFGQNLSFVCSQTNRPPKHSALGEIRQCIYDPRTDGLEYWLSGPEVDCPLVDCGPPPALSGAYYEGDEGHHGGNFKVGSVYLFQCRAPYSLVGKSSYDDRMVRCNVDGTWDLGDLRCEGPVCVDPGHPDDGQTFLDSVEEGAVASFSCNRPGFKPFPAETISCSLGTPCVLSEDVGISSGFIPDGAFSDNSDKVIWGYEPHKSRMSSSGWCGSKDAFIFLSVDLQRIYTLTTLRLTGVAGNGHLSGHVTKMQLFYKVQFSQNYDNYPMEFSTPSGNHRKIYQFTLNPPLRARYILLGITEYEKNPCLRFDMHGCLAPLSTTHEVPAHLQVGWNASIPQCLDAEPPTFKNCPQSPIIVQTDENGQLFPANYVIPEATDNSGRITYMLTKPEDFHPPYPVSQDTDIIYQAFDDAGNMAECAVRLRIPDTVPPILKCPDSYAIWAQENQTELHMHFNESLVRLVVQDQSPITQISYDPPEARIKLDSHVTVEASVLDAHSNRNKCKFQVALLPEPCSPWSLRIDEATVQKQCQRHASGTVCQVQCRKGYRFLESFPQQKSEVKKQQNSTNTLPQRYSCSMEHQSGKWLPSPTPPACVPMAMEPARYEMRVHMNYSLTSPLPSDCAKSYELLVGSLFDSIDQVLSQRCSSTVQIYVRFLDAKFSQMGEKTVSFFKKFFYKHLLQMSANFTVQILPTVLQQVFYELCSLTLRTIFDLRIPGATIPIRSLLTLSGDSVPAILNLGCPPINASSISVSQGFSCSQGELLKLQTPNVGSDDFLSAAPGLPECFPCPKGTAFVNNSCIQCPMGSFQDQEGQIRCKPCIENTYTLQSGAQSNESCLDVCGNGMFSATGMIPCQLCPRHTFAGPPPIGGYKECEPCPEGTYTARLGSVGPSHCKQPCAPGHFSVTGLEPCSPCPINFYQPNIGQQRCLQCSNDSFTAETGRSADEHCKKLDCQTLKCQNRGQCVVANHKEVCECRPGFMGSHCEQQIPLCDSHPCLNGGTCELHNGAFRCICPQNYTGSRCQFGPDECISSVHCPNGGVCQDLPGLGTTKCICRTGFTGPDCSQISDPCQSDQPCKNGAQCIPLQLGRYKCKCLPGWEGTNCDKNIDDCTENPCALGAKCHDLINDFECECPHGFSGKRCQIKDNLCDPSPCLNGGQCVDTLFDRHCICKRGWNGTFCEQEVNECSQKPCQNGATCRDQEDDYSCECAPGFHGYQCQYMIDHCAVKPCRNNGTCINRGPIYDCQCPLGYEGDHCEHNVDECEMMTPCDAVGTGRCEDLVNGFKCHCHPGYEGTFCEQHVSQCEDEPCMNNGTCT*

>Mja|M.Javanica_Scaff4571g035264

MLHQQQQQQQQQQKPKFRLQLEDVGNPQPCPDNPIRLLASRTPQYIYSPYDENKLYPPDTDCQFLIEATDKFHRIHLTIIESDLEEALFTDCNDYVSVRDREGNETSIKEVARWCGQDYPAAIASASDSLLILDTCPPDWISDGISSPYCYKQFVLPHILPWYEAQKECNFERANLATFQNEADYAFIVESYSQTHSFPWVGYSDANVEGIYESIDRNVPLWPENFPLKHENEMKDCVYLDWNKRDQIVVYEIDDCRNRRPFLCKKRRDGTEVPVILPAGMIRRGFRDFTMDYTLLVVVVIFALLALVVGCVLFHKYKERNNQIINIDMNQRLVQQQHGQQPGNKDKAAAALARQKAKERERRELREQKYAETSNNNRGGGRGGGGGIATTKSPFESQDSNASFPLQTFQSTFAQRMTSTTTGNVEPLDTAAALAEASATASQQMREHEATKLHPQQLSAEIVSVDSPPEDDLNSSPRIRMSPNQPSFVEEGRKISEKKN*

>Mja|M.Javanica_Scaff457g006494

MKLNFVFLISFFIFLFFSYTTEAHFGCNCQYPNNHYVPWYLSGNVENRLTFLERQTNQKINMLIARIEALERELGLIHRISMQEWNNSGAGNIYKIFNTSKTWEEAKNTCMTFGARLASIDSDYKNAFVRNMIERSFGKDGSVEVWIGLKTRAELTNNPNSHFTNFGEEEKIDGCAVMGIKGKWKIRSCSNLNPFVCEQILM*

>Mja|M.Javanica_Scaff465g006610

MKLSVSKTLLLTYLSIQCIHFAFINADCPAGWNSRSDSSGIEYGYQVVLQKNTTFSQAQSICSGLESDIVSIHNKEENEFIYGLIKTSGVVLYIGMQQKSDQYSPTCSWPDKSDCNFGNFNGVKDPKRQEYPWVRNPSITTDGNTVHCVGIADTTYNFGKPNFKWNDVGCNDGLDGTICKKKCIGGNSGGSASTSQTPEASKASTILPSNTEAPVVSTKSSIGGGSSIGPVVTSKPSGAPVVSTKSAIGGGSTIGPVVTSKPSGAPVVSTKSAIGGGSTIGPVVTAQSPKTFCYLIYQVFSRDLLPKTCKTEVGNNGSCGNCNCNDGYKCGSDGWQVKHGKDGSFAYKYFPAPNSSYFTAKAICAKYHALPCSINSEEENEFLLENVANNQLKSNKRAKRETQDPACIWTGVHIVVSDNDKEENECYCDDGKECSYGHKKENNEDDDDDNTKEDDHDDKKKKGKEEKDKKKKEKEDKKKKEKEDKDKKKEEKDKKKEDKDDKNKKEKEEKDKKKKEKEDKDKKKEEKEDKKKQEKEEKDKKKKEKEDKKKQEKEEKDKKKKEKEDKKKEKEEKDKKKKDKKNKSKDDSDEDDHKKCSCKDKKKPPSPWAPGCPSKPGGGHKKHCVGIGNDGKWKDVNCEKPGTGIICKRPCSP*

>Mja|M.Javanica_Scaff5270g038643

MKKRESYINEENIGKTKLSSNSIEKRLVRLEANQQNLRRATLADWPAFSVDKRVRIFDHTRSTWIDAQRECSEHAGTLLEIDSESENERINEMLTNSGSNNRPNDHYWIGVQIMLQFSNGSSIIGNYSNFEEQNNSENTQNQKLPKGLVMKRCAAISASQTTKSEGRWLSLECSEKHGFICQL*

>Mja|M.Javanica_Scaff5339g039008

MKLSVSKTLILSYLSIQCIHFAFINADCPAGWNSRSDSSGIEYGYQVVLQKNITFSQAQSICSGLESDIVSIHSKEENEFIYGLIKTNGVVLYIGMQQKSDQYSPTCSWPDKSDCNFGNFNGVKDPKRQEYPWVRNPSITTDGNTVHCVGIADTTYNFGKPNFKWNDVGCNDGLDGTICKKKCIGGNSGGSASTSQTPEASKASTILPSNTEAPLVSTKSSIGGGSSIGPVVTSKPSGAPVVSTKSAIGGGSTIGPVVTAQSPKTFATLLPGFPGSTPKPINGGGNNGSCGNCNCNDGYKCGSDDWQVKHGKDGSFAYKYFPAPNSSYFTAKAICAKYHALPCSINSEEENEFLLENVANNQIKSNKRAKRETQDPACIWTGVHIVVLDNDKEENECYCDDGKECSYGHKKENNEDDDDDTKEDGHDDKKKKEKEEKDKKKKEKEDKKNKEKEDKDKKKEEKDKKKEDKDDKNKKEKEEKDKKKKEKEDKDKKKEEKEDKKKQEKEEKDKKKKEKEDKKKQEKEEKDKKKKEKEDKKKKEKEEKDKKKKDKKDKSKDDSDEDDHKKCSCKDKKKPPSPWAPGCPSKPGGGDKKHCVGIGNDGKWMDVNCEKPGTGIICKRPCSP*

>Mja|M.Javanica_Scaff5629g040312

MYFYFNKIILLFLFSIFVSLYSKISKTIENGEIEVKTGEDKIGDNKIRKWRKYVIKRKSNGGKNEEEKINEGERSLLDAPPVNEKSKGNLDNKEKNKIKNVLDWNKQRAPVICGDEGWHYFDGFCYKLINGKFTWEEALGECKKQNSNLVSIQSEQENDFVGSLSTKFDQNFDNFCHGFWIGMTRKYIEETDSFKSEWSDGTLVNYGNVPIKIAFHTPPWMGGQPDFAGGVEECVHSYPKLGCDNWQQIFFNQWNDARCSIKLSGAVCKKRQSIKKFEKKK*

>Mja|M.Javanica_Scaff6404g043785

MIERNFGKDGSAEVWIGLKTRAELTNNPNSHFTNFGEEEKIDGCAVMGIKGKWKIRSCSNLQPFVCEQILM*

>Mja|M.Javanica_Scaff6528g044354

MKLSFSKINLLAVAVLISFSFVNCGCPEGWGTIDNVSGFKVFTSTDPINLFMALPVCKSNGGIIASLYSSDESAFIAQMIGNDTPVWVGAVMKNNGDGSFSCKMIDGGDCPSEIQNSFQNQPTDTETPIYGTQLISGKWNVVDCADRVYAVACKMSCDQ

>Mja|M.Javanica_Scaff6859g045763

MHLSSILIILPILFSSILTQQIPNCQTRIFQCDADWDTRTDPNTGDVYGYKAFIVNKMNFYEADVFCRARCAEVVSIHSQEEQNFVRRIAAPVLNRCQFTSACAPVVQSTNATFDRQLRSFWIGLNRVTGTLKDAHSVDSSVYQLWSDRTEVDFGSFPNNSVNTGTNSPPWTRGNPNGVNSAYFIIRKRHVLFSLLLISTGRHVIPPAYFTVRTADPQIIEDCTQMVESRQGGWNDFPCNWRISGVICKRKCTSYCAAQDV

>Mja|M.Javanica_Scaff6859g045765

MEIAYFLVCLSILLPYNFLIAQKVPNCPARSYNCDTDWQLFTDPDSGVEYGYKAFIQDSINFYEADIFCRARCAEVVSIHSQQEQDFVTKIAGPLLTKCQFTDACSERMEHTNKSAANFDRMLRGFWIGMNRVYGTLLNHNTIDTNVTCIWSDRSKCDYGSVDNGSVTPDTVTPPWARGNPNGANPGGDPLIVQACVQMVEGRHGGWNDISCTYRLGGLICKRKCSSYCAAQDA

>Mja|M.Javanica_Scaff6885g045867

MGMSPILFSLIFGVIPTLVSADCSVGNWTTRVDSDGNTYGYQVLMRDWLNFYEARALCLGVGGDVVSIHSNAENEFVRQLAAPYIAACQTNKTVCGSRATTSLDFYLSVVWMGMTRCQYFPSYNATVDCVYSDGTKCDYATSSSVYPWGVGSPSGSDSGGGAGLIEDCVSMYNGTSGEWNDVSCFQKLGGVVCKRNCTGTCGSTSIKTALITSPSDITDTSCTSGNWTKRAGEDGNTYGYQVVMKDWLNFYEANAQCLALGAEVVSIHTVAENEFIRQLAGPYITACQTNTSVCVTQWRSLWLGLHRCAFYPTYNATVDCINSDGTVCDYLNITGGPSGTETGTASGQQEACAAMYSATTGQWNDIACFNKLGGVICKKNCSKACGVASTTTTQLTTTTTQPSTTTTAEPTTTTTLPTTTTTLPTTTTPLPTTTTTEPSTTTTQPTTTTTEPSTTTTAEPTTTTTEPSTTTSFNCAQPPVTSLLSYNTSDPNIQTGPNATEGVCECPADPGNNNVFFIPVTTITTGSSASNSSVIMKCSKMQDFCICDEDDICWKVINAYSLVVINSFCDPTCHNYARLQNAAPFNQTFESDCGRTITLADELTPIPNTNRNTFKPLGSSADYYIKAASIRCLQAGQTCTPIKCSGTRKPSPCPTTTTTMPTTTTTLPTTTTTLPTTTTTLPTTTTTLPTTTTTLPTTTTTLPSTTTVLPTTTTTLPTTTTTLPTTTTTLLTTTTTLPSTTTVLPTTTTNLPTTTTTLPTTTTTLPTTTTTLPTTTTTLPTTTTTLPTTTTPPLNCADCSAGQSKVIYEKNTLLSNQTINNLAATECIYNCKDKDVSQACYNPSPISVVRIRCTDSTKFCVCTSDNKGCFTVTQSAPLYQADYLIYANASYTFLALNTGASQIKNSGTGKTYDFQTSMTFIPNDNTFLNAQYLASMLHYFVRYYCPIANNNVSCNGCNLIHNNGMDVQKPC

>Mja|M.Javanica_Scaff6956g046145

MYNDGKVYAYKAFVQESINFFEANALCREQCAEVVSIHSKEENEFVRAIAQPLLDDCQNEIICKSRPGETSPDFNRILHSFFIGMHRIQFGRLGQCTRDPNIYCIWSDGTICDFGNYTGVIGPHETVPPWATGNPNGIEQYGNEDCVEFYDSVNGYWNDIGCQMRLSGVICKRLCNSYCAEMDTQLK

>Mja|M.Javanica_Scaff6956g046146

MFPNWALLLPIFGLFAYSSAQSRCKFICEADWQTRVEPTDGKTYGYKVFFKSALNFFEVNIRDYIILNFSNIFQLKAQRICRDNCAEVATIHSRDEYDFVFLTFRDLLDQCQTNASVCPIRDPNPDFNRILNSFWLGMHRIQQYTSICQGCSVDSEIVYVNSDGTPYNFGTYNADRAVLEDCTQLLDSETGKWNDIPCHYQQAAVVCKRDCASFCQAQVPPITTTEATTTILSPGGQPGGPGLPGPNGPNGADATTSAPDNGNNPGPGGPGGNGPPGQNGPNGPNGADATTSAPDNGNNPGPGGPGGNGPPEHKDRMXX

>Mja|M.Javanica_Scaff725g009324

MKLNFVFLISFLIFLFLSYTTEAHFGCNCQYPNNHYVPWYLSGNVENRLTFLERQTNQKINMLIARIEALERELGLIHRISMQEWNNSGAGNIYKIFNTSKTWEEAKNTCMTFGARLASIDSDYKNAFVRSERTLIENKYVNFTIKDMIERSFGKDESAEVWIGLKTRAELTNNPNSHFTNFGEEEKIDGCAVMGIKGKWKIRSCSNLKPFVCEQILM

>Mja|M.Javanica_Scaff7378g047848

MKKIGWNVAEINCLYNQGHQISVKNIKENKYLEDIARRVGGLIWLGAAQFGNNYMWTDGTPFYFTFWQNGIQPLFNPGKKCIKMNSLSGEWIQSCCRVAAPFVCQKPAFKIDKKHLLRNEERKEEFIQR

>Mja|M.Javanica_Scaff765g009691

MGMSPILFSLIFGLIPTLVSADCSVGNWTTRVDSDGNTYGYQVLMRDWLNFYEARALCLGVGGDVVSVHSNAENEFVRQLAAPYIAACQTNKTVCGSRATTSLDFYLSVVWIGMTRCQYFPSYNSTVDCVYSDGTTCNYATSSSVYPWSVGSPSGSDSGGGAGLIEDCVSMYNGTSGEWNDVSCFQKLGGVVCKRNCTGTCGSTSIKTALLTSPSDITDTSCSSGNWTKRAGEDGNTYGYQVVMKDWLNFYEANAQCLALGAEVVSIHTVAENEFIRQLAAPYITACQTNTSVCVTQWRSLWLGLHRCAFYPTYNATVDCINSDGTVCDYLNITGGPSGTETGTASGQQEACAAMYSATTGQWNDIACFNKLGGVICKKNCSKACGVASTTTTRLTTTTTQLSTTTTAEPTTTTTLTTTTTTLPTTTTTLPTTTTTEPSTTTTLPTTTTTEPSTTTTAEPTTTTTEPSTTTSFNCAQPPVTSLLSYNTSDPNIQTGPNATGGVCECPADPGNNNVFFIPVTTITTGSSASNSSVIMKCSKMQDFCICDEDDICWKVINAYSLVVINSFCDPTCHMYARLQNAAPFNQTFESDCGRTITLADELTPIPNTNRNTFKPLGSSADYYIKAASIRCLQAGQTCTPIKCSGTRKPSPCPTTTTTMPSTTTTLPTTTTTLPTTTTTLPTTTTTLPTTTTTLPTTTTTLPTTTTTLPTTTTTLPTTTTTLPTTTTTLPTTTTTLPTTTTTLPTTTTTLPTTTTTLPTTTTTLPTTTTTLPTTTTTLPTTTTTLPTTTTTSPTTTTTLPTTTTTLPTTTTTLPTTTTTLPRTTTTLPSTTTVLPTTTTPPINCADCSAGQSKVIYEKNTLLSNQTINNLAATECIYNCKDKDVSQACYNPSPISVVRIRCTDSTKFCVCTSDNKGCFTVTQSAPLYQADYLIYANASYTFLALNTGASQIKNSGTGKTYDFQTSMTFIPNDNTFLNAQYLAISCNGCNPIHNNGMDVQKPC

>Mja|M.Javanica_Scaff784g009870

MKPAPLYFTIIFSFPIVLFFLNFNIITAQKSTCVPFECNDTWDLFQDNDGKVYAYKAFVQESINFFEANALCREQCAEVVSIHSKEENEFVRAIAQPLLDDCQNEIICKSRPGETSPDFNRILHSFFIGMHRIQFGRLGQCTRDPNIYCIWSDGTICDFGNYTGVIGPHETVPPWATGNPNGIEQYGNEDCVEFYDSVNGYWNDIGCQMRLSGVICKRLCNSYCAEMDTQLK

>Mja|M.Javanica_Scaff784g009871

MFPNWALLLPIFGLFAYSSAQSRCKFICEADWQTRVEPTDGKTYGYKVFFKSALNFFEVNIRDYIILNFSNIFQFKAQRICRDNCAEVATIHSRDEYDFVFLTFRDLLDQCQTNASVCPIRDPNPDFNRILNSFWLGMHRIQQYTSICQGCSVDSEIVYVNSDGTPYNFGTYNADRAVLEDCTQLLDSETGKWNDIPCHYQQAAVVCKRDCASFCQAQVPPITTTEVV

>Mja|M.Javanica_Scaff7939g050057

MHLSSILIILPILFSSIQTQQIPNCQTRIFQCDADWDTRTDPNTGDVYGYKAFIVNKMNFYEADVFCRARCAEVVSIHSQEEQNFVRRIAAPVLNRCQFTSACAPVVQSSNGTFDRQLRSFWIGLNRVTGTLKDAHSVDSSVYQLWSDRTEVDFGSFPNNSVNSNTNSPPWTRGNPNGVNSADPQIIEDCTQMVESRQGGWNDFPCNWRISGVICKRKCTSYCAAQDV

>Mja|M.Javanica_Scaff8031g050410

MFYVFFPILNSSDGQSEDCTQIYLATSSGIAKWNDLACNQKIGGFICKKPCDGERKHDVCGKDGWPHVDGKAFHAFPLHSPGNYWQALSICHKHDAQVASIHSDEENKITSALAIGQAGGNISACSWIGLHSAHGGSKERFWDDGSSVDFGKHVEPGNLPVSDDISSDDKSKDTHHCHECAAINGNGHWKDLNCGANCGAVICKKGCRNPE

>Mja|M.Javanica_Scaff8031g050411

MEAFLKKEINHKNKHNENNQCTEDWTTFRNPKDGHLYGYKVFVEDMINYFQAELRCRSMGAEIVSIHSAEENSFVAKLAAPLLEECQNNPLICAERVPHIDKAHELLDHILRGFWIGLHRNQFYPFYNPAVVQVWSDRSECDYGCYEGEVYCDMNIEPWGNCDPSGTNSSHGHGQSEDCTGIYLATSSGIAKWNDLACNQKIGGFICKKPCEGEHKHDVCGKDGWPYVDGKAFHAFPLHSPGNYWQALSICHKHDAQVASIHSNEENKITSALAIGQAGGNISACSWIGLHSAKGGSKERFWDDGSNVDYGKHVEPGTLPLSDDISSDDKSKDTHHCHECAAINGNGHWKDLNCGANCGAVICKKGCRNPE

>Mja|M.Javanica_Scaff8044g050467

MKKRESYINEEENNGKTKLSSNSIEKRLDRLEANQQNLRRATLADWPAFSVDKRVRIFDHTRSTWIDAQRECSEHAGTLLEIDSESENERINEMLTNSGSSNRPNDHYWIGVQIMLQFSNGSSIIGNYSNFEEQNNSENTQNQKLSKGLVMKRCAAISASQTTNSEGRWLSLECSEKHGFICQL

>Mja|M.Javanica_Scaff8853g053509

MLHFITILLFIFHLAIAQNGDNLNDYEDIIATTASYNNLELNADNANAKPSTASTTTGDNIMVLENLNNATGNPADQADDSSDKPLYPNSNPNENGGANQCPDGWATRQDKDGNVYGYKVIMQDLVNYYQARLLCIKEGGELVSIHSEEEEQFVVQLATPLLDKCQTDTSVCKQRVPIPSLDKMYRSLYIGLNRAAIEPYYEPTVVQAWSDGTPVDYASVPTPGVPTTIIPPWGAGCPSGLNDTGNTPQKGSPEDCVCMFKYDEVNWNDISCYHKLGGGICKRKCNGGGGGGGGQPCGCGKVNCGTDGWSQSANGKGYKIFQLPSPGNYWQALAMCASNGAKVASYHSDQQKSAITNICQSSNCAWIGLHSATDGNKYWDDGSQYDYKNLLEVSLCQKKPKSPSCGGGNPPSGDNCPKDCTAVSSSGQTTDQSCESLCGAVICEKDYLTEIQTPKPQGHKIIKTKIYTNDSSFERARRAKSNETKINQIRLDLTKL

>Mja|M.Javanica_Scaff910g011117

MKLSVSKTLLLTYLSIQCIHFAFINADCPAGWNSRTDSSGIEYGYQVVLQKNITFSQAQSICSGLESDIVSIHSKEENEFIYGLIKTSGVVLFIGMQQKSDQYSPTCSFTDKSECNFGNFNGVKDPKRQEYPWVRNPSITTDGNTVHCVGIADTTYNVGKPNFKWNDVGCNDGLDGTICKKKCIGGNSGGSASNSQTAEASKASTILPANTGAPVVSTKSAIGGGSSFGPVVTSKPSGAPVVSTKPAIGGGSSIGPVVTSKPSGAPVVSTKPAIVGGSSIGPVVTAQSPKTFATLLPGLPGSTPKPVNGGGNNGSCGNCNCNDGYKCGSDDWQVKHGKDGSFAYKYFPAPNSSYFTAKAICAKYHALPCSINSEEENEFLLENVANNQLKSKKRAKRETQDKCIWTGVHIVVSDNDKEENECYCDDGKECSYGHKKGDNEDDDDDNTKEDDHDDKKKKEKEEKDKKKKDKEDKKKEKEDKKKEEKYKKKEKEDRDKKKEEKEDKNKKEKEEKDKKKKEKEDKKKQEKEEKDKKKKEKEDKKKQEKENKDKKKKEKEDKKKREKEEKDKKKKDKKDKSKDDSDEDDHKKCSCKDKKKPPSPWAPGCPSKPGGGDKKHCVGIGNDGKWKDVNCEKPGTGIICKRPCSP

>Mja|M.Javanica_Scaff97g001858

MMEAFLKKEINHKNNEHNENNQCTDDWTTFRNPKDGHLYGYKVFVEDMINYFQAEVKCRSMGAEIVSIHSAEENSFVAKLAAPLLAECQNNPLICAERVPHIDKAHELLDHILRGFWIGLHRNQFYPFYNPAVVQVWSDRSECDYGCYEGEVYCDMNIEPWGNCDPSGTNSSHGHGQSEDCTGIYLATSSGIAKWNDLACNQKIGGFICKKPCEGEHKHDVCGKDGWPYVDGKAFHAFPLHSPGNYWQALSICHKHDAQVASIHSNEENKITSALAIGQAGGNISACSWIGLHSAKGGSKERFWDDGSNVDYGKHVEPGTLPLSDDISSDDKSKDTHHCHECAAINGNGHWKDLNCGANCGAVICKKGCRNPE

>Ppa|PPA00415

MSEDDLHYSHCSFCIASACRFSACPLTECAVCYAVLHECKRDDHQEICRMEMVPCLNSSFGCPLSIRRSLLSRHLSVCPASVISCSHERARAPRDKPAKKELKAIGKMRKEKEHDCPLLIIEGYTYSRADRVRRRDEINIREPQLPLRPIVDGSLSDGLKPSSPLPEQDLNVDSSDDERKIEEARIKKLRGIFADCYMCQVDPCVQHFHTLGRSDVRWDRLMYLRSRPSYYHMDPFYAERNLMVSLHVEKIPEAARRSDNILKGHRGGTVYTRRCLAAVPRREVDEHHESQHVTGGDVDIESLIVRCPLWWRGCRFHTARIRPRGGQIRFIQELGAFSFRPEVFPSPILTDSSPLLDAPEWLICEVARFLSGVALNALTQTCKHLRNTLFSSVAVDRGVVSLKWTKQDRCRWTSLPVWSFPLTESSPHFDVNPLSDLCYHIGKCPYNEPNPLPEFTKTEQVAPVNGAVTCLKEAVRKAMHEEIRKRLTNVISSAIVIHLLLKFPLPSTKSLPNRLMTSTEMSLRSVALIVLSSVKPRVPLLIGSKTKMTYKAAVNHCEEQGGSLVNICSFFENQFVRGITRGADTWIEPKTECDYKNYLTGNEPTSVKECRVITDDWDGQWTAADCESEKHHAICEMKAPRPTPIAFRFRRFFGF

>Ppa|PPA01109

MLLIGLLLLRLYVGSAELTCPYDYTLFNGKCYTFVDEPLDIAGAMMICEKIGAELVSIHSEEEAAFIVDSHGGSESWIGLFCNGWDENWEWTDQTAVDYVNFEDRTSNTENCKEVPESQRFGLRDDLLWWKREWNELHNFTCMRDPINCPDGFEFLTNSYCYTSNATVKMDYGNATQYCANLDAYLPSVESPEVSNALTVTLKMDGADYAWIGLECQQATTSWYWSDHVPYARSQANFADGSGSDTCYPFDQNKYALDTKGKWKGIPNTNQLSYVICTAAALPMPTEAPDIAHCPEGYTKFRGKSPDDDWCFAMNHPLPLGNMATADFATATDFCKRRGEMLPVVESSAMTIFLRSIRASFDVVRKSPFWIALECGDNGSWVWSDGTPLDPNYTQWYGNTDPDPCEPDFRVVFNGDGKWEQVAATAGQHLIICAVRASTSTEDPFMPTQPESEDCPSGMHELTEKWCGVYSQPGSWTWSAAANYCAINGYGSLPSLAAALQLQRQNYVGTDQEAFWIGLECVPAGDKFPRYFHAPFKIFNIYACIAIGNTTPSPANNMKRALNNAFEDQDYGDTGAWSWLDGTPWNDDYVMFLYSKTNCTVDNTRFVFNSIERWAGVDGEENALSFVVCTAPKLGYFTTVTTEAPVPIFRCDLDWLELYPGLCYSISNVVAAYNTAQLSCASKDAVLPSIQSPKTNDAFMDVLKHLGYESTGFWIGLGCDEEGGFCWDDGAVFDYKRFKDIHEQCSTDNSTAYYFEIHGDWIETPIDDERYIVCERPAYEEVSPPQPVLCNKPFAEIDGQCYSFSTKLAVQLTYQKASDSCDSMQSRLPSIPDQTTNAKILFEANGVMFWLGVLCESGILEWDDGTPMLYDNFDAALITNRRCDKLGDNLRYIFASDGKWRPVEKSSTEALLVCMKEGTPYTPTLPATTTMTTTTVATTKPTTKKSVDPSSSEETDDPDEPSTKTTHKTTITTTSSPPAPIQWWMFVVIGAAVILGILVIVLIVFFVRYYCKKKSSAPFAKKIYEEAYEAARKKTSRYAMFPRKEDDWEIDRRFVLIDYDKRLGQGAFGSVHEGRVLHKNLPPGASRSIIEMSALKKGNDLVAVKMLHESADKAAEMEFRDEIDLMKTIGYHERLVNMLACVTDSEPVLLIIEYCPHGDLLQFMRERRMYMLEHEDDPCVDGTLIITQRKQLMFAMQIAYGLEYLSSRGFVHRDIAARNILVDHNESCKGGRLPLKWMSPEAMAKYEFSVASDVWSFGVLLFEIITLGGTPYPDWPASELLQRLKRGERMDRPDNCTDAMHALLHECWRNDPADRPVFSTLRKKLAMQLEQVSPDEYYLKLNAQANYYAASSQHEPRGGTWGNNTCISGFQPLTDGWCFATLERGTYTFQDAEDACYSKFEAHLPTLSTAELNDAVVGVRNNLFKANSYIWIGLNCSEDGDWYWLDGTPYDAAQANFDPGSLLVFDALRCDPTLTTTFTLTVFGYWQALAAESQVSHVFCTARSNESGQTQSPEELICPEGYTLVDDDGEKWCFVWNKPLPPESSVNFEEVKSLCEERLGETLPILRSSKMVDALDAIRSEKDAGTLWIGIVCNHETLDWEWEDGVPLGYPDSFTNFLSAPPFPCTDDKRFVQTIFQKKWIEDNTESRLISYVSSTTSPPLPTLPPSSLCPPEMTLLHDWCYSFDLSPIGDSDYEVNSEENGETEEEHLHHYCEKRYGGFLPSVSSEQESDDLMFLRRFHVGFDAENLWMALECGDKNQWIWTNGELWSGYTKFASAVSCNYNSLDNGHLFDATGKWIDGYEQKARYVACSRRPEGYASASTVATTTTKSALVCLDGYKKYTVNGEEWCFVMNYPIAIGMSFEAAKKMCDYREGETLPLLKSRQMVDALGTFRESLGTNDLWIGIVCNFSTLQWEWEDGVPVDYNNFMDEQTGECAQQDHFVQSALTEKWIKMNTYALTDEYVCMRRAMESIPPLPTLPPSPYCPDNMYPLGEWCYGMTKPMTATNFKTAEYFCERDFHAFLPSMSSDKELENLIFVRRYFLGSDSADLWIGVQCGTDDKWKWSNGEPWSGYSKFKDPEDVNSCAHEGKHSSFLFDKNGLWIDGTDIKTEYIVCAVRYSEITFITTIIPTTQSEPERCPPNFLEILNGTCISKAWKEAVTFDTNIKKCNDSGATLPSISSEEENNEYVDLMNGTTGISSIWIGLLCVPNEDFDGSGSWAWQDGTPFTYSHFIDTFTFHPTYCYPDRNEAFTLEIGGGWNPQNRESAQKHGICVTPADLSVSTPVPAICDDGWQSIDGACFIFDATKKKLSRFNAQQECKKLDANLPSITSRDQNERIYYATYHMTTFANAQFWLGLQCGKEVPGTFSWVDGTPTTTATTTTDTTTVSTATATTTTARPTTTTAQQPTTTATATAPTPPSGFPTWAIILICVGSVLIIAICAFLGYMARRRITRLEAEIVNIERHDSRAHSNKIEHSLYPGNPIHRYYNLPSRTDEWEIERKFVGIDYTNKLGEGAFGSVFLGRVLAKNIPLAAGKSIVEMTTLRNDNDSVAVKMLHENADTMAETDFKAEIDLMKKIGYHERLVNLLACVTQSEPVLLITEYCSNGDLLEFMRERRKYMIEHTSNYDEDKIITVKKQVMFAIQVAYGLEYLATRGFLHRDIAARNIMIDHQETCKIGDFGLCRAIGRDNENYQAQGGKLPLKWMSPEAIDKYFFSTASDVWSYGILLFEIVTLGGTPYAGWPASDLLNKLKRGERMDKPDNCSEKLKRLGKLLEEVNQDDYYLKLNAQANYYVFESDERPMPGCGRRAQEIFSNMRTLNQ

>Ppa|PPA01120

MHLSTLQDVLRDIDFKFSMCFVKTNKIVNAKSLSMVLIIILTVGTSHEMITKFAIWADRPRRAFGLEHVRRSPLSWLIFLPLLLGSLLAAAGIVAKRGELLVPLTVMMLLYMPLLFHSILTADQLHIFDPRLLVLFSIGSLLVVLHCIFILLAAKNEIDREVDEVFSVGKSMEFSETELSRTLRTIKSFRHNFVESSCPPGLEPVRDGQCRGKYADSMNTHFDEGYAAVVAKCAEIQGQPIIIHSEEDQSYWVNWNDENGADLGNAPIAIVSSLCPDGFELVKDGECRGKYVDETMYFDTAYDRIVSKCNEINATNHTGLTGRKRMARSRLSCNPITMQWQWADGSVVDFKPPIHSAELDQDCKAGCFWFLDTDGRWLVGCSSTLSLRANIHCTTQLVQPIPSEDGCYGFAGYDTDGVCYQIGKTVETWQEAQADCRRVGGNLASIHNQQENSFIRRLAVSNGAVNGLYLGAIMTGKGNQYGFPMDGVGNCLGMDTSTTSGQWMNINCVTNLNAACIRQPIFPEPPACGTGKENEIIYSPGFPFDSSIPCEYFLTVPMGKIVELEVLFLEANSCCDRLIVSDNFLGGTVIANLSGEVMDKILTTASTNVIRVSWQPHGGVNVRGVMMRYRAVTPDENGELSDVVDIPSRVDYCFLIDSLHYCFRYELICGDSRELFFLILTSLGQFLIISCHLIKIIFWLTITKYDRYYMYSIPIFRIAQPLNDLGGFLTDVNNFLLVIERTIASSQLERYEQSRMNWPVLLSCEVFCICVSLGVAYLVHFVRIVTECSILVIAMALLTIIVLVVCHFHNTRKYATAVGVSKKYQMKEVEYLTRALIPACIVNALMRILTSFMAVFSANLGFTSYVSFMLTYHTIQTINAVQWGVLVVARHEGMRRRTRDLLRALLGRRQLSARANIGEINLRKDNTKDYFDYLNSHWEGGCYFGRQSNLPSNRKREKPSSTSRVLRFLTPQSSRSLIRIRVKEKRRRVENDATGQPILTTMASRGFDPVRTHSEKIAMSEPRLKKCFLCLESAPQTRRFPQSSKPDEQLEWLLRQNRDEEGFQQLLNRHRTVKEPRWCLRHFTSPTDSLPTDNSSMPPSPSFSALDTMPQQPPWEPPTSPIRFDDDVLWNYADEVLTKRFKKEVLNARPTLNVKPKDPPKPPQPHRPRSSSFHATLPAPHYTVAVAVEEFFNEEHCIEIPILLPPPFLVGSIESVFHCLEDVQDIEETRFDRKFFGARAKIALLFTLINVFVVLCVKDIGIANDNLIRLI

>Ppa|PPA01181

MLVPLLASLFSLEIALSETKHKCPDNYRPGNGDGRCYNISYLPAGFEFAVPYDQQINICHDNGGDLVSITTVQEQFSLQWFRDIDQIKFETVFYIGLECENDHWKWTDNRTYNPFAANFADPEATNYCGRMEWTSLTSTFTSYKLFSLLFMCTLGIVFASIIGVIIVAAALIAIYICWRRRKNRQMNKVIQELTAKANYYDQEKAHNVENIRKADWEIDRLFVTVDWTNRLGNGAFGTVFLGHLSTDNLPAMAIESSIAVAALERNNGRVAVKMLSDSSNNADIAFRVEIDLMKNLGFHERLGETDYQTAVNMLACVTESSPTLLIVEFCAKGDLLGHMRKCREYMMSINSHSSLDYQQIITEKQQYIFAVQIACGLEYLSTRGYVHRDIAARNVLVDQNDAAKIGDFGLCRKLENEDGLYLTRGGRLPLKWMAPEALRNFEMSTASDVWAYGVFLFEIVTLGGSPYAGWTHSEVLPRLEAGERMARPDCCPETTDELMTACWMQRPCDRPSFTMIRCHLEDTLERMNCDTNYLLLDSQQEYYHIEPEHDQNVPENVIFSHIRTNQIVRRDHLSLFDGPLPLSVEQLKTTSAETPDGYQLPTSSPLKSPNPFMSPNQKARDDNGYQVPRPSPR

>Ppa|PPA01392

MKTRAPLLLTILVGVSAAVSCPPQYQLMEGRCIRPITLSRFDYLVNLMPRCQEACNKDGTHLPIIRNDQENDFFNNITNSFAETEGENVFLVLGLICNETTTRLQWEDGTRINYVPSGSGDMKYNCINSPYMVTSAVFAGNWNRNAINGLYPYTCLCVIEANEEPIEESTQQPPNQIHCGEYSLIEDAEDAEKPCFKVFTEPLSWDDAQIKCASEFGSLITINNDEENGFFWRTAVNNGMLGGMHIGARHSSKDTKNWSWIDGNDSIIGKSYDNFVGSFPIPGAGECASMATESVVAEWVNEDCKDNKLPFICRRGVLPAASPGCPKNAPKDGDDFFPPGYPNSGIACEYFLNVDSGKLVELEILAMAAAENVDFLEIYEGSSGLNLLANLTGAIDTPTKFTTTKSNVLRVNWKPNGSGDGKGFRIRYNEIAAQERKDDAIITTTNKAIGSAFRLTSVLVFLIMLTM

>Ppa|PPA02713

MGNFWQVQALITLLLIARYIDASVYSCEELRYKLINTTFTQNTNYACIITQEGFTHWNQLEKVYAQVDKYYTSFHDIATGAGSCIENVRMEHPWDFFADSGVVLDCSQEFTLILTSDKPTSSASACTADTCSPCPKQACGCTRGDVWDREPCDALPDWLFSFDNVITIDSDHSVEMTVECSSVLELDAMTMVLPGDRIAILTSGRSDDLQNMEGSDKAAFVLIGEWESHDVTVDMQLSFDPTNTGSIVLQKSWGGPEFKYYNGSHTEKFTTSFFEIHYAPKLKQPSEIWNSQDNIVIEIAIDSKAPSTEPTAAPPNKDPYCDCSVDASGAPVGWSYRGIWLDIVIVIDVSEAMGQESIDRASTLTESLVSTLVTDTTAPLYTRVGVIAMGTDAKVLYNLNMTKADKVKATVDNGVREINIVYAFEAAQGMFSDGLTSKPDRANARQVIYFMTDSDYKNDVNSINAFKDASGVIVVSNFLKNGEIEKSELNHLASLGYFFSDNTSNTALLQAFCKANCYCQSDKIPFGGSDPALKAAGGCYKPAPDERDPFSKAKSACSINGGMLATIHDDEKNHFVQKIMESSKSDFFWIGYSKSDDGAWHWDDESTDPYTNWAVDEPSTAAVSNCAYVDTTSSVLTWGAGNCQIGFPYACEYEPCSVGSMDC

>Ppa|PPA03201

MRFFIVTSILVGLCGTAPAQPASTCGDFSTYTNPQSGKSTCYLIETSWASWNEAEADCVQRGGHLASIHDDNLNSFLRNAARDAGIYDSFHIGLTDARFGSGFAWSDDSVLNYSNFGPGIPNINYGHCMYLQIQNLPGLSSGLWFNEQCDDYNLPYVCTKN

>Ppa|PPA03617

MFPLLLLLLFSTSLVIADYPCPGNYTWYQGPDNYHDGYCVTFSDDFLLDGPQAIAYCEGMGAFAPSIHNQKQLNYWADMTVRAGVGHYWLGTNCPHESDPYTWTDKTRTDYFGPNKELLSCKKGNGFHMHGDGFVLFALVDDPTSSTEQFSAAPALCAYAMTFYTTVQPETTIAMSDDQTTSNDGVQTVETTVKTTPKATTLPPTTIPTTTAYSPPLPDLNEAYCTCNPEEPSLYLDLVFVVDNSADMTGQMLGAAVAAIQTVMFAIPFGDQMWQTQVGVVSFAGDVKTVVNFGDLKSVDDIFSWNIPLSKAKSTMMTSAIDTATEMIDNKARNYTRGVIILLSNSYNQLDDTNIAEASESFQDDGGIFMTIDYSSKGNLAGLKKIASDGYYFNSKTELDNLVVDLIYGLCDANCFCSDGLTPYDSQNERGTPQGCYHAAISAAVFDAADNNCRNEKGFVATAHDSAKSFFLLSLFDKSKTYWIGYQRKNGAFKWEDNSKDTYTNWAASNPVSNKDCVYSKQTNGFNSAWGSMRFALVLAAVAAAVAAQSSDTIDTSFSDETQPAFTDEPTTDSGYETATPSPDDPRCRSGFRYYNGWCMFTSDPSSYTYQQAVSVCSSMGALAPSIHNKYELDFWSKSTETSVTGHFWLDAYCPSSGKPYVWRDGTPTDYMGPRGELQQCIMNSGYHIHPDGFEFYDYSTPAPAICVYPVDPSPEPSPVDVTEEYCACTPKQIYLDIVFVVDASADMTSRTVGDATATIQSTLLGLTLGTAMYQSNVAVVAFGDKVQTVRNFGTLRSENDIFSLNIPFLGGKSTKMTDAILQASSMISSNERDFTRGVIVLLSKSFNQLDATNIREASDAFQYDGGIFITIDYAKGQGIKGLKDISTTGYYINDATERETLNANIIYAFCDANCFCPDGLYPYNVADDTTGREVPKGCYHVADVAAVYNAAEQNCLAQNGFVATVHDNNKNFFMISLFPPKSRYWLGLKQPNGRDFEWADGADVDFTYWAPGNPIAGDACVYGQQSTGFNSLWYSAPCTDPLKYSMSYACQLRPCDTDSMRLALALLALVAVASAQTNDYDFGGYSTDDYNGVSASPSPIDNYCACTPKQIYLDIVFIVDSSADMTSRTVGDATATIQSTLLGLTFGTQLYQSQVAVLAFGDKVQTVKNFGDLRNENDIFTMNIPYLGGKATKMTDAIQQASSMISTNEREFTRGVIVLLSKSFNQLDATNLKEASDAFQYDGGIFITIDYSKGGIRGLADIATTGYYINDASSKPDSLNADIIYAFCDANCFCADGLYPYNLADRTTGREVPMGCYHVADVAAVYNAAEQNCKGQKGFVATIHDNDKSFFMMSIFPPKARFWLGLKRVGSDFVWADGVSEGQEYWAPGNPVAAQDCVYGQQQQGFNASWFSAPCTDPLKNSMGYACQLRPCDTEYDCSIPE

>Ppa|PPA03637

MIASAPSRSARQLIYIIAASDSSEIGRYANQCEKRHDATCDVEKFKRNGGIIAVMEASSSLNVGWFAELASPGYYDTSFADNINYYLNMLCFANCFCPRGLIPFSRPDALNRNAPDGDCFFSQSPAPYSTAKKVCRASGARLATIHDLYKQFFLMQASGGKPFWIGGVLNGEQLEPIDDSTAQYTNWAAGELNASRGRCAYSMQSAEYASEWHSADCSREVKYICALAPCSVDNFCK

>Ppa|PPA04072

MWRLLLAFAIMGTAHCSCPRGFDLIANGQCRGFVGFANQTSEQGPMIASQKCSGVQSGHAVSIHNQEQQSYWQSKAPSANEGLLVLGLHCNKLAKKWEWTDGSAMDFKPSNYQSDLDIVCSFDVTWYIQHDGYWTYAAAGSKLHAAISCNADLPHRTFDEGCENFGDDGGNGFCYQIGVKAKSWQDAQSICQEAGANLASIHNDKENAFVHRLALGNGAMQGVYIGASTRNDDEPFRWVDGTSVDYELYGPGSPKPNGGHCVGMETATSGDGFWTNVDCDWTMAYACKRRKRDQVEQPTCYAEDWKENTLITSPGFPSSAATPCDYFLSVDAGKRVSVEIELEANACCDSLVLYEGGSVIATLTGDLHNVTYTTTTSNIMKVSWQPNGGVNVNGLAMTFRSV

>Ppa|PPA04265

MHGLLLLSAAIGLSYSMVKHSDSTLYDVYDFEEAASIYLTNCDGGCYIYASTVGNTDPNDHDTRDPYSRNLMIRENNKGRDLIDIADLSDKVNEDLQKTPMIIEGPASISVVNRNPTGTKPSTPLVLWVVNDEIASRVSHQVFDVDEVGLIMPILDTITFISPRGYRLQADPGDYATNVTVRLAGYDNALDNNLDGCPIAFKSRNSTTFAPFPGFVIQPDSPLVTLTFAGSNLFWLRLSFEFEHERNLAEDGFVSSPGWNGCKNPIIGDYQSFRSPYYQPTDSFLLSTDEYYGSNEQDNYYVNAKSVNFTFVDLSGNQGFLALYYSVTPTVWTAPESYCNCAIKNSQPVDWKPSQIWLDIVLVIDTSASMAERLEEAKAMVTSFITGLNLPPWSSSGQYDEFYSRIGVVAASDRAEQVYFMNMTATDNLDMVKSTAAASMNYELAFIAALKEFKYIEQRPRVRQVVYFLTNSVPPANITGVHSFKENGGVIIVNEFVSKGASPTPFLSSLASDNYFFTDLSTDYTANWGVLCEGGCYQAFNDNLPQSSSYDMCHLKGYNGAGALVSIHDQQKEFFVNSVASSFGPKTKYWLGLRYNGKAWAWDDKSTNAFTDWDANQPNLNGGENQCAYAVQTTGLNVKWTAADCTEHFIGYVSLLSSTMYDVYDFDQKEVHLNKCDDGCIIYAATMRDTFDQYLDKIDPYARNLFIYDNNKGANVMSITELASQVNPSTKVKQRLNIQGPANISVINKNAPGTEKFTVVLYVVANAHGGILPHALYDIADVAGAVLTTATRNVTFISAQPYRLEAVQSDQPNNVSVVLAGFDSWWSSDFCPVAFESRNSTASNPFPGFVIQPNTPIVSLFLGMSNPIELHPSYGFEHNRPLSEDGFVTSPGWNGCTKPNRGGIQTFRSPSDKPTDNYLLTSDSGLDVDIRVAPNLDYDHELSVIIDDGSRKLHTIFDGTSPHENIWSSLIGINISYTDMSADQGFIATYYSGFHVEPTPPPVWQSPERYCSCDMKDGLADDWKTTDINLDIVLVIDTSAAMAERLDEAKQMITSFISGISTDPEAEFYTKIGVIAASDTIDVIYNMTMKSTDNLDAVQSKNVDTVDYDTAFLTAYALIGRGGDVTRNRAARPVVYFLTSTEPSKDVLGASRFKFIGGNIIVNEFISAGASPSEALRDLASENYYFSDLSEDYTNNWSQVVSAISTTPKFIRSMTIPRIRVLGQIVAVSKLSTISYRIQLLRTNAGRITSTSREPSFPYTIRRKIVSEFGPKTKYWIGLQNNGTSWNWADKSTNSFSDWDTNQPNANGGKNQCAYAAQATGLNVKWTAADCDMDGVLYVCETAPCSNAIVAALLCSYCNCDLVDDWFDGWNPAQIWIDIVIIIDTTGSMGNSLESAKAMLTSFISLMSTNTNRQFFSRIGVIAGSDTTEVVYNLNMSYSDNLDSVKPSESDKMNFDIAFQAAVVMLNDGRKLPSYSDNRREIIYILTKAAPPGAFVRGVDSFKTRGGTVIVNDYVMDGDFPVQSLSKIASENYYFSNLTSYVSNLCFAKPIASVIQPTHCTRGCFHTSSLSAPLSRAREVCQDIRSALVSIHDAEKEFFVNSVVSGLGKNKKYWIALQYNGSEWNWDDQSTDPFNDWDVGQPYTDDGQLECAYATQAMGLNVKWTATNCSLGGYVTVCESAPCSLQTATVMPVFDDTDPKYIHCCGCHIKRLARILATTEAVIVGILVLILLFDPSAFDPVIVLSLIFTGIIVYSSYYEHRNGVIAYLVSVIIFSVIDIYEIFNHPIAQNSSSNTALTISLVIFLAIEISYFRVFYLFQDYLREKHGECALLMTVQEATNPLVPHTDNHTSPPAYEEPSSSKSANHSRNLSVVL

>Ppa|PPA04644

MSSNAMCSSKLQQIRDLLGATNTLPGLVPCTCHFLRDTHVLLVRVQSVAYTGISIILFATAVSHYYFAARSQRTLAITILGTSGASAVLACIGVTVFEMLMTGILGFCGSSIASYGVSICSLIFLCLSIAVNALLFVASLLNWRQQMQIEQTLKVPTKSPYPTHLKMSSTSSCTSKVQQVKELLGANTGRHLLLHLASSIAHATISLARYFSGALSLFYTGFAATQFGTAIAHYYFAPKSQQTLAIIILATSGASAVLGFTGIVIIMIIASDSSFFYYDEALCVCTMVFLGGSIIMNSLLFAASLFHWRQQKQIEETFKVALSSFYVVIFAILFFTACAHFFFEKKSMDKLTHTKITTSFASAVLGAAGVIAVGIIMKSGGCYYSPSGALFVCTMIFLAGSVVINVTIFVLVRLHRIQLRVIEETLQGLTIMECEAKVKESTQFEASAINEVSQSSFPPVVPTKTTLVPPSALLYPIHQLGSIASMNGEYCTRPSMPVVFGVISIACTTFSVVQFSMAITHYFFVKRSQKAITITILAISGASAMLACIGIIVTVIIVNSIVGYCYYDVVRYPLFICTMTFLGGCVVLNVLLFATSLFHWRQQMPIEQTLKCSTSSGYASWGKEPSQFEDIDLNELAKTAPVPVAPKTALVPPSAPLYPTHQLGSIASFESLTPLRSRFLSLTVTTTPMLRQLYDGQNTYASNGAMQAIDFCSDYGAIPVNIVNQEDHDYWVSVSRSDKKNGNQYGNIILGIYCDSTNHWMWADGREVYYEWKPEDYDEDLHERCGENGQYCMWTINPTTNNWQKWCNTYQTTDKYCIIPPASKSEEPDRDCTDFDHDDDNDVCYQVGKYPANWTEANTICHSFGANVASVHNDLENSFIRRLSVSKGLFNGMWLGGALNAKKNAYKWADGTKWDYDNFVPGFPMNGLGECVAMQTNNVAGQWVNIDCATELPFACIRAPDIQDHVCDGGLRKENEIITNPGFPYDASVPCDFMLTVDPGMLVEVEILMLEANSCCDRLVLAEGLLGGPEIAILTGALYNGWTFRTTSQNAMRVSWQPNGGVNVKGMMFGDCNISTYSTLYTYAIICCFLPIFILSTMCLLIFILHTVQTELTERKKSSQSESTPPMPRPSVIIPRPRCIPHINSRILQSLNHLNLAMFLLSFLQQSSNIHQPRWSHVCTVFCSSSASSASVVLGASGNLVLVILLQFGECDIASYSTLCTCIFISCTLSLVLVPLVCLLMVVLHEINQKQLALTERKSRANYGDKEIANKSEEIEIRATNESAQSEFPSPAPLPSSIVPSSASLYPSHQLDDEIAMTEASKSVAPPPTVIYPLSSSLYPTDQLGSISSFESLSEMIEKVNCSKLQKEILMINIAAIASAIMDCVSGCAFAIIITSNEDDSDTDVLMYWTITFFLVAIVLDIGLLAASYLYWPPKKRRFPTETAQSVIE

>Ppa|PPA04648

MLRLSLLASLCYSAWAVCPSGFELVRNGECHRQLYDGQTTYAPNGALQAIDFCNDYGAIPVNIVNQEDHDYWVSVSRKDKKNGNQYGNIILGIYCDNTNHWMWADGREVYYEWKPEDYDEDLHERCGENGQYCMWTINPTTNNWQKWCNTYQTTDNYCLILPTSKPEEPDRDCTNFDHDDDNDVCYQVGKYPANWTEANTICHSFGANVASIHNDLENSFIRRLSVSKGLINGMLLGGSLNAKKNAYKWADGTKWDYDNFAPGFPLNGFGECVAMETNNVGGQWINVDCTTELPFACIRAPDDSDPVCDGSVKKENDIIYNPGFPSDASIPCDFMLKVDPGMLVEVEILMLEANSCCDRLVLSEGTLGGQEIAILTGELYDGWTFRTTSQNVMRASWQPKGGVNVKGMMITFLHSSPSSRLTRSSRPRAEWSLLLLLRSLSTLPHLLASTTLGVMGPQLLLHCIVALNCLQTGWELFLIYRQRRKHESTVKRPAGVESIISEEDYTKARSYSIDRLSFKVFRMVIECALMITMLYSGYYHYLWTTATSTWAPLSVFLILHNLVSFLLDLPLSLYENFVIEELHGFNKYTGSFYIVDAIKKLVLSTAITIPLASGAVWLIENGGDLFFIYLWVFISIVILLAMTIYPAYIAPLFDQYSPLPDGDLKKSIEELAAKLEYPLKKIFVVDGSTRSGHSNAYLFGFWKNKQIVLYDTLLSGEEKRKVYEALGKKVDEDKEEKEGKKKDDDKGMGVEEVVAVVGHELGHWALSHTVRQLGVAELNILLTLFCFSHFYTNETLGAAFGFTGGAPTVISLLVVMQYVMGVYNEIFGLISVSLTRRMEFEADAFAASLGLGSKLTTALIRLSKDNLSVPVNDSLYSLCRHTHPPVTERIEALKKKQ

>Ppa|PPA04669

MPIVFNTLENPKTHIVYGCKESAGIPVAAGFLSLFETCGRIMKRTLLSLLLIVPSISAGPCPDGYKQAPNGGDCFQTIKYKDFFSGNYLPQSYDAAETDCNNNYWGGLLASLHSSEENDFIGSNCGLSGATLNIGLKCKGRLCKWDDGSPITYTNFAGEGPSDSDGDACYAVKKDCGQQKADCWICRVKARKIECTDEEYERNGGCVSIHLAPLDQKSAQESCPNGGHLVSIHSKNEIIWFTSIALESGVNGSIYIGGQYTNGVFEWADGSYKNYANWANGFPNTIFGACVQMLLDSAFGVQGQWTNVDCTTKMPYFCFRDGATYGPPVAPQPKANPKCLSLHDIYSPNYPLSIPGQQTCEYVIDVNEGSQASIKFTTYDCKSGTTLSFFDGLNSNQPYLTFTSDSPILNQPYSATTNVMKIVFSANDTTIPVGTGWEAEFAEL

>Ppa|PPA04672

MRDATIFIILLFPIVNCDSCPKGYTQAPNGGDCFKTINFYDSFSGNYFPESFDEADTLCNDNYAGGLLASIHSEEENSLIQQENDPNLSCNAPLATKNIGLKCTGKLCTWYDGTPLTYTNFDGDGPSDKDGESCYGLMGNEGKWYKKKNCGKQNSDCWICRVKARTIDCTDDEISHNSGCVSVHKTPMDQQSAAVSCPGGGHLAAIHGNFERDFYTKVALDAGVVGSVYIGGQFSNGLFEWSDGTFKNFENWANGFPNTIFGSCVQILLDSEFGVQGQWTNIDCGTKQAYICYREAPSDFPTVAPRPKANAKCPSVQYYTVNGNIYSPNYPLSIPSGQSCEYVIGTNEGTRASINFSSYDCQTGTTLSLYDGLDSKHPFLTFADTQTPNATYSTSSNVMKIVFTADETSVGTGWEANFVGI

>Ppa|PPA04770

MNQWWDEAATMADAKCKEIGGKPMIIHSEEENSYWTKWVAGAGGPQMVLPLVCNENTKKWEWTDGSPVDYKPAVYYKELDADCPTGYTWDFHADGSWGVGHAHVEIVPDICCKTQLPPLPVQQDDECDGFDDDSDDAVCYKIAAPAENFKEAQKICRSFGAGLASVHSLQENSFIRRLAVSNGHVKGMFLGATISGKGDKFGWMDGHDLYFSADYEKCFPIKGLAMDTEGTSGYWVNTDCSSSLSVACMRQQNYVAPACTSGIYSEGEIVLLLEANSCCDHLILFDNYVAGNVIANLTGEISVSIYHTSPSNMMRVSWQPNGGVNVRGMMLKRTKEMKKQSNEKDFKKCFSNNVIIVSAMPMMGV

>Ppa|PPA04790

MHGGGFEPTTTSPEKQSTIRTGKIEWINLQAFGGIYIHADVWCVTQPSREPFPYNDGCDGFDEDREDGIGEKAESWVEAQMNCGKLGANLASIHNTQENSFVRRVAVSGRFNVEGLEKVRVGRRSFPMPGVGDCLAKDTSTVNGQWMRILIVRENCMWRAFAINNRSLISTGPWKEKTNITSPGFPYSSYTPCDFFLTVDEGKKIEMEVVYLHANASDSLTIFDGFLGGNVIATLTGEVKSTTYTSISNLMRVSWQHNSKKNASELVYCSCPDGYELVKDGECRGFSAKIASTYEESRDVALAHCGEVQGKPVIIHTEEQQQYWLKKREAYTSSYLVLGLKCNGTTNKAEWVDGSPIDYIPKDGGIDDMNYCYTDCSWYLWNDGHYHSLCTKIDVKVLDIFCTTQLQQPVPTGDSCSSIDEDDKDAGCYQVSKIAENWEDAQKICKTLGADLASVHSQQENNFIRRLAVTNGAVQGVSLGGRSGGNGGQWADGSTWDYDNFAAGQPVAGQGECLTMDTSTSAGQWKNTDCSAAMPIACKRQPSNAVCSSGPWTEGQFITSPGYPFNASTPCEYQFSVAAGKRVEVEIIILEANSCCDQLVLYDAFTGGNIVTNVTGEVNNKVYKTKSSSKMRVSWQPSGGVNVKGMMFVDTKHMIKMAPNSKIEYHKCLHCSCPPLFDLAHAGQCRGLVAEETVYRDEAYASAVAKCSEIQAQPVIIHDDEAQHYWQTLQQRFDGKIILGLTCNTDTKKWMWADGSVLDYLPQEGHDAALDEPCTSTGCSVYMSTVGHWHVWCDHAAEYVTVCCTTQLTLPTPSGDGCESFEDDSEDGVCYQIIATAEDEQYAETICESFGAELASIHNQQENSFIRRLAVSKGAVNGLFLGGTLPIGSKNSFWTDGSKWDYDNYKAGYPNQQLGQCVYMDTQDTAGQWMNRECSDKLPAACIRKPGYIAPACSSSSYKEGDIITSPGFPFNSSSPCDYSLLVKGGKRVQIEILMLEANSCCDFLYLYDIDDSVIAKYTGKQHDKMAPTSSSNYVRVSWQPNGAVNVRGLMILALAAVLATLGMSIASFITINNQQSIIQSIQDTLNAQFSSVAPPAVTSTVPAAVKTTTALPDDDKTTPFEDTIPEAEPITKDDPRYGAYSGMSQLLSTWMNRSVNPCDDFYAYTCGAGVQGQGMSFDVSDDAITDTLVGVLRQPANSFNNDPLPVRQLKWFYDSCMTKVSPTNAEKAARSKQIFDDLRAANPGIVNGIFNTINSVGLLLGKDVDQVQAQKDAKDIAQLDYDLAITYSTDETTRRQFARSYNPYSVDGLQKLAPFLNWKTFFNKALTPISKTVDGSFRSIAMEVDKLAMLSADVASGKISSRTLNNYVYLRVLNSNYLPQKGDLITTGYLKNFLRDKRPINRKIRREPKKRDPMEIASDYTKQESSCASATTNYLMWANTRVYVDANYPKEADKKAVREQTNSIIRSILVAFRAQVDLLDWMSPASKKGAYQKIDNLVVNIAFPDWVLDDAKLTDYYKNLDIKQNEAYQAQLDKLNAFGLYEAFLPLINGAPTDRTDFTGPSAITNAWYQPEMNSITFPGGILHAPFYDASYPAAINYGGLGVIAGHELTHGFDDQGVQWEGTGILNGWMDDNSTKAFTSMAQCVIDEYSQFCPLGAGHPCVDGAQTQGENIADNGGIQAAYKAFKAYEALHGPDPLLPGFASLFNADQLFFLGFAQVWCQYPPSANSLQNQILNDPHSPSLYRVLGTVQNIPAFQKAFNCPAGSTYAPVNHCNVWTSEPTSGAPLNDKGEPIVPDNDINIAPIDRISPQDMAKYNAYQQVQGIYKNTMSDLNLANDRIINDKLNDADYQATIQASTALTKLKTLYTTCKAEWEHSTIGTTDYLQPKVLKFRNAINQDIPIIGGTGAIDVAPADYGNALGYLSFQLGIDTLVSTGVDTNWMDPQGALGGTNGYQLFVDQATTYHVRAFYEDENWYKQKPGYAAQLKTLIEAYVKQMEKIDDIFQLQDTTAKLPADYVDMIDKVLELEKKIAITYSGTDDERRHYERQWQPKKLADLPKTVDWSLYFQQAPQVVQDWVAAKDIIVNEFDYTTKMFTDLGNTGDDTVVNYLFFRLLLDNSGLISTFNFADQAARRALAVKRGVPEHTGKSRIPGRRHPLPSFAIRSDDEDYEGEGCADDTAVMADAQGRVYIDARFPTEADRNQIRDKTQGVMNNIVNAMKGMIIELDWMTEASKQSALKKASNIQVNAAFPDFILDNAKLDAKYADLIFDQADSYYAMLDKVLLYSINEQFKLLTAAKADRTDFLGQTAIVNAWYDPELNSITFPAGILQQPYFDVNFPAGLNYGGIGIVAGHELTHGFDDEGVQWDYDGRLNSWMDKTSQDGFNAMAQCVIDEYSQFCPLPDDRSPHCTDGVRTQGENIADNGGVHSAWRAYEAHIELDGPDPMFMDRVFSQYTENQLYFMNFAQGWCMAKSYMTESYVSNRLMTDPHSLGPYRVLGTFQNIPAFQANFNCKLGSTYAPNQHCSQQTYWTEKQKEWGSRVVLGLVCNTSTLKRQWSDGSPVDYKPPSSLNNPVCTNELNHFDFFCTVQLPHQSPSGDGCESFADDSDDGVCYQVANVAQNWKDAQTICRTFGANTASVHNAQENSFLRRLAVSKGAVNGMFLGANTSGKGNIFGWIDGSEWDYDQFYPGFPVNVLGDCVIMDTGDTSGDWANKISLRKAAPLSFGQKTGKYNDPL

>Ppa|PPA04810

MDVEVERKILLVQHASGCSTSRSKVQLVRVEAKCNNFLPPDLEWVKDRRTIIFLLQEDSNANYATYVANPIVGVFSFLILAMLRQLVSEVKRGMHDSSEATRRYQQIPDRSLIASIAGHHGNAYAGQFVDYDILFLFISTKSCRMERIIATANPEVYHRTKLAMSVLIPLTIFLIDFTFLIGRLAYQWNEFEIAGTFLLFCDIMTIVTNALSVKYNRQRFQEAFDNLNAKYQAKEAFQLSSAMQPIYIVIFCMKFVIVISSIICLGMNDPFFNGYNEAFYTTVTAIYASIVAIMLIKKHPQLLKQARLILGIRISVAPLPPRNVCYTQDHFNILNFRLDSAHVPLSMQKLIIFVAILKIAYGSCPAGLELVRDGECRGLYATQSIDDFSQASAVAIAKCAEIHGKPVIIHNEEHQDYYADRMPKYQDYNYLILGLVCNYTSKRWIWADGSLIDYRKPAEGHNQALDKECQQGETWMLTDDGYWGYGDHWMGTSTIYIYCTTQLEQPKGDGCESFDDDVEDGECYQVTEKAQTWKEAQTTCRNLGANVASIHNLQENSFIRRLAVSKGAVNGMYIGGTPAGKGNQFGWIDGTDWDYENWYPGYPVNGGGDCLAMDTLSSAGQWMNQDCSSSLAVACARDANPRPTCHPGPWKEGEVIYSPGYPFDASLGCDYFLTVVAGKRVQVEVILLEANTCCDHLLMEDDVLGGNLVANLTGEVSNKIFTTDKSNLMRVSWHPNGGVNVRGMMMTFQHVLSSCPAGYDLIRDGECRGFQGTISATFAQAVLNVTDTCTAIQGKPVIIRNDEHQSYWKKMATPTDKGYLIIGLVCNSTTKRWDWADGSLASSADFRPTDGYDDVFDQYCDSGYSWYMDNTGYWKKGNSKNMFTNIQAYCTTQLQQPSGDGCDSFADDGADGFCYQVIPTTQTWQDAVETCQNIASTIASIHNSQENSFIRKLAVSNGALNGVYIGAKGTGNQYSWMDGSEWDYKNFYPGFPINGRGNCLAMDTFAPTGEWMNMDCNSKLAVACQRRAIPRPTCSAGPWMEGETIYSPGYPYDSSEPCDYLLSVERGKNVQIEIQVLEANSCCDYLVIEDQLLGGNIIAKSILYSKRLCKPFSLTGEISNKKYTTMLSNFARVSWQPNGGVNVRDDLPRSVICKSGAFIMIIENIITFFNMKFLLLAVSLLHFAYSSCPSGFDLIRDGECRGQQGTISAWFSQASSKVVDKCKTISGQPGIIHNDEHQLYWKKQAPPSNKGYFILGLVCNANTKRWEWADGSADDYRPSDGYHPALDDVCKPNWSWYIDNFGYWDLGNSNSTFTVQVFCTVQLQQPVGDGCDSFGDDSGDHMCYQVSATSQSWQDAQAACQDQGATVASIHNKQENTFIRNLAVANGAMNGVYIGAKGVGNQFSWIDGSAWDYKNFYPGFPVNGKGDCLAMDTFAPTGEWLNMDCNSKMAVACAKIGELSRYFWRGELMPLPRPTCTGGPWKEGETIYSPGYPYDASASCDYQFSVAAGKMVQVEIQQLEANTCCDFLVLEDKMMGGNIVANLTGEIYNKMYTTSSSNFMRVSWLPNGGVNVRGVMMTFRGV

>Ppa|PPA04842

MKLIILFAVASLIWACPPDYRLLARGRCFKTLQNRTKDTPANLLKQALTDCGKDGAYPPIIFSEEDNDMFNAVASTVETPEGAAPAKLLLGLVCNQKSRRLEWADDSKVDYRKNGLSVDFDCTKDETVVSEPSKNDWLVVSTTRTDYTYTIMCTTQPPEIPDDPCGDYDRMPDPTGYESVFTPCYKLHLESKSWNAAQSVCNDEKAALARIETDEENSYLWRTAVQSSAVNGVHIGLYQDAAQNWIWVDDDSDAYYDPYVVSGFPIRMSPPGKCGALQTENSKKQWINIDCDNEPLPFFCRRNETAESNVCNTDDLEQEKEIIAPGFPHPNISCEYSLSVSIGQIVELQIDFFEANECCDSLEILEGPIGPHPIATLTGTDLETTVYHTTSSNVMRVNWKPNGAINVRGFKMRYRGIGGTSVASLQPVEATTTEEVTESTSASEASMEHTTSPRIRTTEFVETSTTAITETSTMPSTSTTEEASTLAHSSSLQLRPRRNRLP

>Ppa|PPA04844

MLLRLLIAAAVATLASTESCPAGFDLMGGGRCIQALDFEIPGKLSTLLLDRVAKCEALNGHLPIVKSAQDNTDYINIVAGFDELKGKNVMLVLGLICNTTTARLNWEDHSAITYIQPPAPGQLNVDLTYDCVKRTDRVVSRITPKDWFVVPDTTSYPYTFLCETDPIEANLDAHCGEYEEMTVSTDFNNPCIKIYADKLVCLQAQERCHKEDFGSLATIHNDQENLYIWRTAIASNVTAGGVHIGAYLPDGETTSWKWIDGDVPITGSVYNHFESFPPPGVTTGMACSVMIVDASGKAFWETEDCDTQLAPSVCRRSRFPPPTFLCPTTPPPAGIPFFPPGFPNSNITCEYNLRVEAGMLVELEVMNLEANKGADFLVVYEGVDGNPLKNLTGSTPVPPDNIITTVSKNTMRVSWEPHGAVNVRGFRLQYRPVPP

>Ppa|PPA04853

MRALLLLSFLIVLLAAKNCPPQYQLIQDRCIRLLELEKVFVIDDALPAYRAECAKDGGHLPVIKNDQDNAQFSSIVYALNGNAWKDFYLMMDLFCDSTTKRLQWMDGTPVTYSRRDSIALDVDCYDSQLTAISEVEPQHDEWSYVSTQQKLYGYTVICDYDGPEIAGPCGDYAEMDTSSSAEKKCFKIFTEPMSWNDASKQCAADSASLITINSDEENRYIWRTAISSYILDGLHIGAHASPADASVWTWSDGDVPITGNVYNNFVNGFPLPGGGECSSMLTGTTAAYWINDNCDSVKRPFMCRRAKNLP

>Ppa|PPA04854

MRVAVFCLGVIISSVSGYCPSLEYTLTGETCIRALQLWTNDTIINLQPQSQRDCGLDGASLPIIKNAEDNDFFNQVANNIDEIAGWNNYLMLGLVCNPWTHELRWQDGSAVDYTPGLNMNFDCTKYTAVSRTKFGDWKLVSVADTWSYTVFCVTDPKYIN

>Ppa|PPA05204

MITFCHIYLLLNQPCFSWLYSSPFSIFPKPFAPKDSTLFEMENATNTFSAPEDAKKACGILSALPPMIKNQEDHDYWLSVSKETHEKGVNDGYIILGLECNRQSKWQWMDGTKVTFIPDGVYLPDLDWCDNTNDPWGWKCMWIMDPKSNNWNQECFTKHDVDLYCVIIPTPNIEEPDPSCDNFSHDGDDDICYQVDKTATNYTEAATICHSFGANVASVHNDRANNFVRRLAVSKGMVTALMLGGRLDGSQKTITWEDGSKDDYRNFAPGFPQSGSGDCIALQTNNVNGQWMNVNCDADYSFACVRPTNAPVPSCDGSLHNEGDIIYSPGFPSSASESCEFVLKVDSGKLVEVEILLIEANSCCDHLSLFEGSLGGNLIANLTGDQYNGYKFRTTSENVMRASWEPNGAVNVRGMMITFRGVGKYRKDESKGYVEYPNATIKCEGKSFKLYEEKIQYPIEIRCSANASFFCGEDSIVIVKPNKENLTRTAPDIINNIQSSLSCPDGKPHVIAHKKLYRNARFECAIYGDKGHWECSDHWKISASSVLKGTLSNENLPECFEGGTLLVDEKNVTGLRCDEKTGEYKYTTDGLEITVEKETKFECNYPAVVALEINEQNTNVLYRTLYISIGISLFLGIAIIIFGITYSNYRIKERKKVALEKLAHDELAWNQYVLNEDEREELKYAKKMERMKLKNGSDDSEKEQSLPIMNLSKWNDMQLNAFSVSTRLIFKDATTTSPPVHLKHMDVLPKPDDELIKSPGFVNEKLGNVPDTATKHRMREAFAERLAKHIEISILSIAFLLLVPALAQEGGDLPRINWTNCAKNGTQVNELCYLRKACSNAPGRPKYMELFPKEGIILKEEYCDVGIHIRFFTSEVALIQMQVIGIPNNSPNAELVIAHTSHKEKYFGCKYSDKEKKIIKIGDKELISKNRIKLFEHRSFFKSGQFLCAITIEFRDDLTEFKILRSISGTNTTDYTADRVNYFTNDLKKIKCDAYAIKSRKVSDHQIQDVINTDRDVINCENYGPLFSFKYFYETEEPTDIKELLCKEYKDGDEEKSKFYVVELNGNESAVEDISRSYFACAAPINKLCTNPNATCTDLSYCPIFTPGNETAPATLTCTGKKWIHKGEYQELTEEPVCKADGNDAVFHIDNEKIQGGICFTDCEIYYHFLLKCILDCSDNCKNHSKLDYPDCKDGGCEDDLDYSDDMMKCKNDKLHIRVYIRSNNTFDRATQFYCNKATGYWEWSNKTVLPEGTQVLCVGNATKPEAVANMTQLQLAFAVSGFIFLCILLTVLTVIGLHCYSNPPACLRVTGKMRWKRMDEPAKIVYCHQILGKTAEPKCWEAMYPFLMRVQGTLRKLDSNVALMFSRYLYLEAKKIIDKHGWNSTTKMTNKTKHKKDLIRVVAIKLLGFALEEKANGDAQKLLQSGFRQLGFNAVHQYPHCGVLWSVISHCRNPGYKDKKTGRLAQFDRMKMAQQLNEVAFKTMYPMATDDVIPEFGSPSEQAAFALGGFAAYHFQATHSKEILTFTDGAAALSYFKGMGASLMRNPAITIIASLKASDGLDEAIAESMLLMAPEKDWIVIFEDIMKTMVYYGRGLDSKKLQGLFLPNAASLVQLYDQLKVDQQFFEEAAECQLEEMRKWFASNTDMDGIKKKPEYIKDDRAVEVANPKPKPKKESNDKSKKGVDKRNDKKEKAAPNEAKPLLNK

>Ppa|PPA05209

MWRLFLCFALLQLASCTCPGTFDLVKDGECRGLVSIETLTFDQAAKTAIEKCNTVPGQPVIIHNEEHQTYWKGKAPATSEGFLILGIICNTTTRKYVWADGSAIDYKPSNGYNEELDSNYCQPDYCWDIHPDGFWSYGGPGAFIKASIYCTMQLEQPPLNTDGCDNFEDDTDDGVCYQVAATAQNYQDAQVLCKKAGADLASIHNSQENAFVRRLAVSRGAVNGVFLGASADKNGRFGWADGSKMDFVNYYPGFPKKNFGECLAMDTSLSAGQWMNVDCAAPLPAACVRDQRPGQAPVCSGDDYDEGQIIVSPGFPNSAATPCDYFLAVPSGKKVELTIILLEANTCCDSLVLYDGTLGATVIAKLSGSIQNQVYTSKTSNIMRVSWQPNGGVNVKGMVASDQEIVHYPSHKLALTICPPNFDLVRDGECRGFVSVEKLTFDQAAPTAIELCAALPSKVQGQPVIIRNQEQQQYWKDRAPATTTDGYLILGIVCNPATKKFEWADGSPIDFRPKEGYNGGELDALCYTEYGWYMRADGYWNFATRGAYVQADIFCTVQLLQPPLNPDGCDNYEDDMGDGVCYHVGAYAEDWQDAYMVCKKEGAELASIHNSQENSFVRRLAVSAGAVNGVYLGASSFFAQEEGAFGWADGTPMDYQNYYNGFPKKDFGDCIAMDTSSTAGQWMNNDCTKAIPAACIRRQKVDPAPICSGQDYYEGQIIVSPGFPNSAATPCDWFLEVPAGKRVQLQVIMLEANSCCDSLVLYDGSLGSKPLATLSGSQHNMTYTTKDSNIMKVSWEPNGGVNVRGMAKMWRLHLAFAILGLAHCSCPKGFDLVADEQCRGFVGKFYLPFELAAPYALEKCVSVQAQAVSIHNEEQQSYWSSKAPATTDGFLDWSAIPSRRSGSGPTGPHWTTNRPPTFAIRAAPKAIEKCADVQAQAVIIHNEEQQSYWSSKAPATSDGFLVLGLVCNSKSKKWEWSDGSALDYKPPTNLRNPELDKPCTPNYTWDIHPDGYWSYGGPGNYIQAYIFCTTDLPPLPTFTDGCENFGQDAGNGQCYQVGVTAASWQDAENICQQAGAHLASLHNKDENAFLRRMAVANGAVNGVFLGASARDNEPFRWVDGTNMDYEPYAPGFPKPNIGYCVAMDTSSANGQWMNVNCAAKMPFACRRKQRNDVEQPTCYDEEWKENTIITSPGFPSSAVTPCDYLLSVAPGKKVSVEIQLEANSCCDSLTIYDGYMGGSVIAILTGDINNGTYTTKTSNIMKVSWQPKGGVNVKGLAMTFRANEHRHLCCLRLSHGSNDQARAVPDRRATPFALAQPITPDQNDENRNETAVTPTRSVPSEVDEAFRKFATSPPSALSTVDSTPSAFHAPRLGLQGLQGFGHALPDALMMTPTSPLSPSPTTVFAPLPATPMLPRVPPLTNNAFWQQMLYNNLVLSLAANQQRLAALAQSSSVLLTPSCPSTPKQTTSPYSFSSAESLARSDAVPSPPSFFSTPKRRASSADHSTPVTGKEVRCPTCGKAFTRPWLLQNHMRVHTGEKPYACDTCGKAFADKSNLRAHTQTHSGLKPYGCTRCGKSFALKAYLSKHEESAYAVTGFSVSSPKPAVSGSPLKTSPAPVRPSGSPVGSTGFFYPTTKGFGNPSVKPVTGGPVSGSPKPAASTGFFYPTTRGFGNPSVKPVTGGPVSGSPKPQGFTGFFYPTTRGFGNPSVKPVTGGPVSGSPKPQGSTGFFYPTTRGFGSPSVKPVTGSPVSGSPKPQGSTGFFYPTTRGFGNPSFKPWWPCLGNHPRMVRTPQNLSDMPVESDSFFAIVNERVSFLFILYFVTREMGVFSTSTSASRNSLPSPLSSFESKTTTEAPVTSEVPVTDGVTPSEAPVEPSESATAADAPAASSPLPDAPVVQSEAPLTSDPVTDSPAPSDAPVVQSEAPLTCDPVTDSPAPSDAPVVQSEAPLTCDPVTNSPAPSDAPVVQSEAPLTSDPVTDSPAPSDAPVVHEEIMLKHLVLFAILQNIRSSCPTGFELVKNGECRGKAPSYYTLSWNTAMSDVGAKCALVCNTATSRWEWSDGSVLDYKPQSYDKVCSAGSANFEIGCTTHLAQPKPAGDGCENFADDNEDGVCYQIGNTTASSEDAKVTCRSFGADVASIHNDKENSFVRRLAVSRGAFNGVYLGATGVGNEFSWIDGSTWDYSNFYNTFPLDGYGKCLAMDTSGTSGQWVNVDCSSKLAVACMGQQNYSAPLCSPGPWKEGQIIYSPGFPDDASIPCDYLFSVNTGKKIEVEVLFVEANSCCDRLVLIENYIGGTVIAKVSWQPDGGVNVRGFMNNITILSDIVNATCRSGFELFRNGECRGNYTRLTPYWDASPNPSIASCKQIQAQPIIIRDAMDQSYWSSKATGGYALLGLVCNSTSKQWNWADGTVLNYKTPSGYATDLDKDCVPSMNWAIQSNGYWYSGAAHNTFTADVFCSIPDSQPVPSPNGCESFDDDSDDAICYQIATTAENFRNAQMVCKNVGGDLASIHNDRENSFVRRLAVSRQALNGVLIGGLLSGNDKAWTDGSAWDYDNFYPGFPINGLGQCLVLDTQGTSGEWMNVDCNNTVAVACERQQNFTTPFCPTEPPKERQLVVSPGFPFDASTPCDYMLMVDAGKKVQMEILMLEANTCCDRLIVYEDYFAGKVIANVTGEFKERVYTTSTSNFMKVSWQPNGGVNVRGMMITFRGVVVSVVSVFESIALIRVDKNRRSLRIELVFRAESIPGYSTSTMLLLILFSILRKHLSLRVPKALNWFAMASAGGFTLLYILVATKNLTSPSQNAQKSMAHQSYLTRHVHGGAYSKILLGLMCNSSSTKWEWVDKSPSDYKPPGDYDAALNKACKLGCVWYLTTNGVWACENSTPQLDVFCTTQLQQPVPAGEGCEGFDDDVEDGACYEVGDSAESWQDAQMNCAKLGANPASIHNSQENSFVRRLAVSKGAVNGLYLGATMSGKGKDFGWVDGTEWDYANFHQGFPMDGLGECLAMDTSSSAGLWMNIDCSVQLPVACVRQQNAIIHPNCSSGPFVEGALITSPGFPYSASTFCDFFLTVEAGKKVEAEILFLEANTCCDSLVLYDGYLGGQVIATLSGEMRNATFTTTTSNVMRVNWQPQGGVNVMGLAVNDLPWSVIPLGVYIREIVLGACPDGFKLTAEGQCSQITPTQVYAGYSQATDTAISKCKEKAAEPIIIHYAEQQSYWDSQYRTNYNLPLGLICNTSSLKWQWTDGSALDYKPSEYDAALNSNCKTGCVWHMASTNGSWNYACSNSGVYFNVFCTIQLPQPIFSDDGCENFNDDSEDGGNEFGWIDGSQWDYDNFFPGFPIAGLGDCLVIDTDGTSGQWANVDCSAQLSVACTKSQNYSPPTCTTGPWKEGQIIISLEANPCCDYVTIIENYFCGNFLANLTGEIERKLITTSSSNFMRVSWQPNGGVNVRGMMSGLDCGEEEKIEETFIRGACPDGFKLYAGGQCELITPLYYTGVRYDQAVNTAISKCKDKHAQPIIIHYAEQNSYFAGKYSSYAQPLGKCIQILSMKLRQAVLVCGLVCNTSSLKWQWSDHSALDYKPSAGYSSGIVTFYDILSALNSNCYTGAVWYIARTDGYWSVASGSSTSSFNVYCTIQLPQPIYSEDGCEYFYDDSEDGVCYRVGEDAETWQEAQLNCKKVGANLASVHNQQENNYLRRLSVSQNAITGLFLGATISGKENNFGWIDGTPWDYQNFYPGFPLPGFGDCVAMDTSSTAGLWMNFDCNSKLPVACIRDKMTNLVANYTCSAGPWEEDTLITSPGFPYNASTPCEFFLMADDGKHVELEILPLEANPCCDNLVLYDGYMSGSKIATVTGIHTNVTYTTTQTNFMRVSWEPKGGYNVMGLANIEKVGNDFYAVLSFCPPGLDLVRGGQCRGFYTTVTVPDDVAENIAANKCKEINGLPIIIHDDEEQAYWKSRATGAYDLVLGLVCSSSRWMWADGSQVDYHPPSFIPRCGDDQDTFQVYCTTQMPPLPVPSPDGCDGFKDNGDDGVCYQVSTTAESWNDAELICRKLGANLASIHNDHENSFIRHLAVSKGEVRGVFIGASSGGRGEKEADFAWVDGSPWDYSLFYNGFELTEFACENAHIRSGFPKDGFGACLTMDTSTTSGQWMNIDCAAKLPAACVRGRTSFSEIVLLNIDIFRKNRACVQFRNMEGARACMFILFQSFFIDITSPGFPYNSATPCEYFLMVESGKRVNVEILLLEANSCYDSLTIYDGYLGGDVLVSLTGEVYNVNYTTTTSNIMKVAWQPNGGINVLGLAMTFPVIHCSCPDGFELIAAGECRMLKTISLNAHDDNATDTFTSQCKNYQAQIVIIHNDEIWILIARMGVLGCDSNAHNFDVYCTAQLQQPIPSGGGCESFEDDSDDEVCYEVANAAQNWKEAQTICRSFGADVASIHNDQENSFLRRLAVSKGAVNGMYLGASPSGKGNQFGWIDGSEWDYEHFYPDDYISSGCYTGAWKEEEIAYSPSYPFDASVPCDFILSVDPGKRVELEVIVLEANSCCDYLIVHESYVGSNIIANLTGALDNTFYTTTSSNSMRVSWQPNGGVNVRGVMSNNIFSVHIQKRCRMLTPINMKIRDDQATDNVISQCKALQAQPVIIHNDEQQSYWTGKRKISSSYVPLGLVCNTSSLEWQWSDGSAVDYKPSASEGGYYEGFDKDCTNGCVALIYNSTGYWARWCGTDVEPFDVYCTVQLQQPTPSVDGCESFEDDSEDGVCYQVGATAENWQDAQILCLKLGANLASIHSSQENSFIRRIAVSRGAVSGVYLGGTISGKGSDFGWIDGSAWDFTNFHSGFPITGAGDCLAMDTSTSSGQWMNIACTEKLPIVCIRDQKPVVEPTCTDGPWEEGHIITSPGFPYNASIPCDFFLIVDEGKKVEVDVVFLEANSCCDSLIIYDGYLGGHVIATLTGEIRNSTYTATSNTMRVSWAPNGGVNVKGMANYSITTCSFMRTPVTTIKFIVFRVHIFNDKMLTLFLLLFAFSIIHCSCPDGFELVAAGECRMTTPISINIRDDQATDTTISQCKALQAQPIIIHNDEDWYATQVRSNGNGLMDRHGDSVRINTFFQENSFIRRIAVSQGAVSGVYLGGTISGKGSDFGWIDGSAWDYTNFHSGAN

>Ppa|PPA05500

MYSHWLIVGGRIVFTNSAILDEFDFKAQSKVTIDGLCRSTCLIYASITDESKKLAQNLLIQTSKGFTTIAEIADLRDPTTNQKLFLENTASLSIVNGNAGNAAGPLVLYVVSRAAEGFDFAEVYEADGLGRVPKWSQSITVMSARPFTLKEANYTAVAGPIGVSAILTGFDARSEGCKTVYSLNRDPFPGFTLTVNGPIVTLFYEPTQSHYPIGELSATTGMGESFPMEPKGFVNSPGFHECPGQPLFRSSLYDFTTPIVRSFHSFSYVPRLKRMRCGKKRSNEWMSLCALLLSKRDGSDKICATLCAFHVSYADREEYEKNVLDAFQTIVMKILLVLALFLFAMGTIEAAVMGLFGTKMINWAASQHPGLDSPDVIRIGNQCGDFSQVYTRDNLRPKTGSGAYLVWKKEKECVDECALLLSKRDGSDKICTSLCKFHFSYADREEYQKNVMDAFRVFMEGSQLRLLDSTMPECKHKNSRMAAKVSCQSQFKGFLPILLDKKTNNVLRIASTRSWIGLDCSSSNEWRWQNNELLKFTSFDRPLGSNKHQQKKRRRAGLLP

>Ppa|PPA05781

MKLILFLTVLPALGAACPFNGFETWLDGRCFRQNYQTSVPYSESSYTCREAVFSSNEAHLPSIFTQLDNDQFYLSLDHQFGYTFWLGLSCDGEKFVWADGSEANYTNFAGDYKCTKAAVDFKYYYGNDKLWYESANGKDSGVNNVVCEAKTRSASPCDSYDEVETVGQTKTCYNLYKATSGWENADAMCTLDFAHLAVIHDQTLNDFIRRTSVSAGLMDGIHIGIQLDRPSGNFSWVDGTDVDYTNFQTGLSTSAASVCGYMDTKSSSGQWGLTNCETIPLPFICTKPAFYVSNPHPAGCPVKTQYAPGDDVYSPSYPGAPGEGICDYLLLEADPNKRVKLEVTFLESNACCDTLTIYDGLSGSTVLTTVSGWHPDPFDIIANSNAVRLHWNASSGVHVRGFHVKMLNSVRK

>Ppa|PPA05905

MSWSHSFFCLLSLYSTMRCAIIVVAFLAIAVNSAEIRIDPPSAQCQGEIDRVWLDVIFLVDSSSGMTQHGLRKADAFLESVMRQMTIGEDTYQESRVGIITYAAEAHVVRDLSRSDNSSSTLPYAGDDEANLFGAFTAAVNMFDNSTSVHRKKVIVIAASTYEEEGPHDAVQIAKQFKISGGSIVTIDYRVSHSGLSPLADLASNGFAFTNNDLEVANLFNSFLHVNCFCPFDRHGASSTTYGSPDGGCFDDENIQSDWMAASDLCHEEGGFLVKIENEQKQRTMDNVASSSPYWIGLRFNSAHDGFYWQDWSEMNFTRWASSQPDLSIGECAFSKGSHWYSAPCNGDKKGLADHVFFCEIQPCSANADCYHPVAPNVTEPAFPEVSTLRPEEPSTVLPIEVSTLKPEEPSTVLPIEVSTLKPEEPSTVLPIEVSTLKPEEPSTILPIEVSTLKPEEPSTILPIEVSTLKPEEPSTILPIQVSTLKPEEPSTILPIEVSTLKPEQPSTVLPIEVSTLKPEEPSTILPIEVSTLKPEEHSTILPIEVSTLKPEEPSTILPIQVSTLKPEEPSTILPIEVSTLKPEEPSTVLPIEVSTLKPEEPSTILPIEVSTMKPEEPSTILPIEVSTMKPEEPSTILPIEVSTMKPEEPSTILPIEVSTLKPEEPSTVLPIEVSTLKPEEPSTVLPIEVSTMKPEEPSTILPIEVSTLKPEEPSTVLPIEVSTLKPEEPSTILPIEVSTLKPEEPVTEGPASVKPEEPVTEGPASVKPEEPVTEGPASVKPEEPVTEGPVSVKPEEPVTEGPASVKPEEPVTEGPASVKPEEPVTEGPASVKPEKPVTEGPASVKPEETVTEGPASVKPEEPVTEGPASVKPEEPVTEGPASMKPEEPEQPDLPIDFSTAEPDHSNKYFPNGTSVELAWWQELKSTSTVRFANLIAEDGNGRTRYPIKLNSPITFKVSLKNEQNLFSHSFFRQTVKISNFDEESFKWTEIPTYGLLNNMAACVNGVLCPVKVKRGEISMTIDLSAHKLWLHTLQNDAPYQFEIMMTDMISNKFFSFVVQARAYIH

>Ppa|PPA05947

MDPSSMFGRASPSSKIARFALLGPFSYLLTFSGFDLNNSICGSRRPRSRALFSTLVYSLAVILFTARTILVGSRDAPEAQVIQTRKMLILGGCLGAVFIGITVVASVKRSVYRNSYENSTEPLIHHHFLSRYNIWGVEIVIGIWMALCTVLAIASYIFMHIAAKEECTKFKEDLDEAAKAEKLVSRLDQFSHRHIAILKLVSLITERTNTFVSFSTICVFIAHINVFYVISAVGAPNAFGTASCVLWGISTIALLPFILSPPGTIQIMLEKSMATLANHEQLIGHPEKLAMAQLMEDRNRETPTRMAIMKAIKVNAQTPHLGRLEWTPQMQASLLTATFYGSLATISFTGSIADRFGPKYIFSAACCVYVIVTMATPFLAELSFRAYFAARLIMGLAEGFIVPCIGSMAGRWFPPLERSTMAGIYTSGNQLGASSSSVISAALCGSPLGWPSIFYLFGAIGIIWIIAFMTLSSNSPSSNRFITEGEAKFLMAEITRKKAFLPTYFRDELMIPLSMNGLYTTIPFVVQIFMKTFCSIVADALKRKVSVFKDKNENDSWMNTQGILAPTTAAKVFQTMCALGIATSLISLAFLPSCDRPWIAALCLASYGISYSFGIPGYFTALMSIAPQYTGTLTSMTMLAATFANISSPMLTSLMMTLKTEHMWKVVFTITGLLNLTGGIIFLIFGDADIQEWARVEKKSSSVVEEKKKHKIDDEKMERIQVEFSCENYLIVLQVGTTDLATVVQAFYFSVRITRPTRLLLILVQTFSSVNVFEMYHLSVLFSSILVLVNSALFKDYGIPGWACEPQVMKRSAQVPSSVHSLRFADIDIIGALGDSLTAGNGAGALNQNVQAVLQTENRGLVFSVGGDSDLNQHVSLANILKKFNPKLYGFSLGSGPVYDFGSAQFNAAVPGAKAINLTSQAIDLVDIAKSWKLVHIFIGGNDACDWCANPQDETADLFERRIIDAIKILKENLPRTIVVITGMMDITILRDLGDKNEMCSVMHDKLCPCVQNKNFTTEQLRGLCKDYIIQNSGLFDGSDDFTVVVLPFLEDNNRAPLLVNGSVDLSLFAPDCFHFSAYGHAVVGKNLWNNLLQTIPHKPRTVDLTTVASPLYCPNPECPFIRTTKNSFNCGKYWTSITLSPLMKVPPRVDYGDEREKDQPAPPSLVKITWANSQGGKVEFISTARDLAPYHDNYSSTVLIAHSSVTNMRFADVCSISHRRNEMMAIVAMVDVRNFREFWNTVDFDPTVFNETVRVDIDGGYTGTLLWIPPMQAIIFSGTFYGSLLTVFFTGKAIERLGAKAILAASIFVSMAATVATPILAESSFVALFVARMFMGAAESFVMPSIGVMASRWISPSERFAMMEMISSGNQLAASFSAVVTAALCLSPMGWQSVYYFYGLIASLWLVAWILLAANSPDKCRIVSESEAEFLSKTIPVKPSSSERIPWRSILTSRPFITCLFCQFAFVYSGTIMQGFLPTFLRAQLRIPLKMNGLFTLIPFGTMLIMKTLFSLLTRRFNSTKLSTPTGRAKLFQSLSSIGCILAMISMVFFANPNQLWIAIISLAVFGVSLAAAIPGFALSTQSLSPQHSLAINQISAVTGVVAAICAPMIMWLIMTLKVNGVVESIWKTAFSVACALNLIAGVMFILFGDGFSVSLCPKKYDLVRNGECYRQKEKGLEVDAGNGPSNAVYECGNDNAMPVIIRNQEDQDYWWSVAKKDKEKYGDLILGIVCDKRHELHWADGSDIDFRPEKYDADLDEPCHDNGYHFCMFNIDAKTGAWKAQCNIDVTINIYCTIVPPSPSLQPDDDCAEFEHDDDDNVCYQVGASPANWTEANSWCKSFGSSVASIHNDQENSFIRRLAASKGLLNGIMLGGIATNNNNKWIDGSVWDYNNFAPATDRDPTCDGTVRKEGEIIYSPGFPFNSSAACDFILMADSGKLVEVEILLLEANSCCDHLILTEGTLGGAVLADLTGDKNDGMKIRTKTQNSMRVSWQPNGATNVRGMMLMAASSYTSREEGTT

>Ppa|PPA06670

MNCFCPPSTLCGSSDGSEPTLRCFKPATEPASFDRALLNCANESMEMATIHNKNDASLLILSTVKGLPRWFGLRHEYNGFHWSDEEQHSYSNWAAHEPELEKNQNCAYALKGTAKKIEWYFFSPCWIAGSKSHPLTLPPVQNKPFCPSFNHATTHTSNSQVDRPLIVDCSRSPATVIFLSTTHVLQPLVQQKSTAPKKNLPNIGVSQAKSDKSWDENTIVLDKYTSALRICPIWMQVEKSDISMKRTVWKALLMLHLPTRSGSLHNPHLFVTICSSESNDGDEKENNK

>Ppa|PPA07634

MRLSLVLSLILSFCVAVSSANLFRSTNTISGAATDAPQCRNDFDFYKGWCVYTYGWPGPYQSEVERCAEVDAHGPSLHSDEDLKFWSDAVLDLKYDYFWLDAYCPNRGNEPVVWLDGTPTDYYGPDDELAKCVYGSGYMLYSTGLTLFEYRYFGNTLCVYRPELPSTSTTSLLTTQRPITPRPTTAISPPSNETADSCPCNPGTVYLDLVFVVDASDEMTRSTVDDATATIQSTLYGLTFGTGNFQTNVAALAYAETVQKVTNFGDIISTKNISSFSIPFLGGNSAKIADAIKQASLMISVNGRNFARGVIAIFSHNLNGLEAKDMDVVNEFKDNGGVIITVDYSKNLLPSDIASPGYSIHSSSEVSGDLLYAFCDANCFCKDGLSPYNTENERGREVPDSCYHVADTFAVYDAAEANCERKNGFVATVHDDNKNFFLTSRELFELLDFDIFPTKSRYWLGLERNEESFEWADGSADGYTNWAPGNPIGGLDCVYSQQQTGFNAPWFSAPCTDPLKNSMTYACQIRPCSSEHLFEKMRNCQKTRMKIYNHWVPLYDQLVRNPLDYEGFKLIYERYLVEKRENSAWSKLSSAQPDSHQCLNMIGNENNENNDNNEWKLCNWRKNEARNLTKMSETTSEREDLYVWIQINEVPAVALIDSGFQGIPMMRYSFAAIAGLAGDIDTRHRRSFYGNYAAKTVGKIHSCEFEIDGIKYDTSADICFDEDEHDVIIGLNFLRTHNCVIDLPNNRLILNGTEIVDFLPDAEARKHRHAREDERCRNRPMSIKPNKNKRNHIMDMGFAKDDVVRALTESLNDVPGAVSSLYKCAEKEELKSGNKSKTVVDKAIVGISEAVKPKATPEDEKDNQCRKLFEEMKKPDNSFSEGFKNEYENFISAKKTKTDIMNDPESDIGKALAIREKKVQRFDNDLKKRQEKNSKLFVPNELFFIRIAVNEISTLALVETGAPTSFIPKQFAENAELESKIDIRFQNDIEGFGGKKKAAGILSCSINISKFLFDAQCIVMEGDRYAIIGLDFLRKYNSSVMTCPAPANNHTDFAKKEHLFPCLQEDSGIPTEPFLSAFQGLADFVGFMGTAFAPVKSDIAGNVTGWLKDPIGQDTLQKLIASDLKDNGGKLGIATEGLLWLKRGQEFMLLMLIFMVRDYRKDKASTESLVSYSKWPTMADPVVEDQSSSSQDEVLQIKTLTKKHIQVLYRLLILIVDNFGGGRKDLFGCEGGRREGTSTAEGRKEEREGGGWKERGVEECEEKRLEGPMGGEEKKKESEGITFNGSVHVGIVPEGKIRKWLCVQFPPSPSFEVDQQMLADLIALKFDGKDAERALKKTLNDLDGAVTMTITVSNEAKSIPNMGESSPSIDNVKYSGPGDKEQNKKCRRLFEKLRKCEKTRNKIFVHWKPLHDKFIDNHLDYDSFQTAYEAYLTVMRGKADWSKYSHLESQLIENVDEENIEWQQINRKEKKRRNKLPEKVLPRASRSLYIWIEVNGVSTIAFVDSGSGGTCMQYSFAVKVGLASDIDTIYRSTCCGLYTVKTVGDIHSCKFEIGGSTYESVVEVWTESEPDCDVLIGMDFLRRHNSIIDLPNNRLMLNGCKTVEFLPREQAREFRRERRRNKPVKINCNKEKINQIVDMGFEKDIAEKQLRKSMNDVLNAVHNLFLRAQKDTRRAELENRAKKELQINEVLIELNSLLDKEEELKCRQLYERMKKPLTKRTTYFNWPALLIQYINFPCDYDGFKQEYVAFLTMKKNRNDIMNDKSSSIGQALTLHKAKEDAFEKQSAQVRKPPLCQSRLVTFTGKIHVGGVPEGKIRRWNYRRKFTPPPSFKVDDEMLSELIAMKFDKKEAIEALEETLNDIELAVSDLYYKEHDREIMAQLLQMRLAFILVFFAAAASAHIPRPALNASALSGAAKKIVSSNSDVQCKSGFSLYNGWCLYNSAPTNYYYQDAVDICTGKGEFAPSVHNADQLDFWKTLASEVEHFWLDTYCPSAGNTYLWRDGTPTDYYGPSNELLNCKEEVGYHMHNTGFASYGYTESGASAVCAYDPQRYVDVTSDYCSCSPNTIYLDIVFVVDTSDDMTIGLVGDATATILSTLYGLTFGTGMFQANVAIVAFAETVQTVVNFGGFRTSKDITGFSLLFMGGNSDKMAEALKQASSMINSNGRDFTRGAIVLLTNSFDQRHASNINEVARAFQDDNGIIITIDYSVGTGIQGLEDITSPGYYVNDTSTNPDLDADVLYALCDANCFCKDGLSVYNVANERGRSLPKGCYHVSGFSAVYDAADDNCHNMDGFVATVHDDDKNFYMISLFPPKSRYWLGYRKNGTNYEWADHSNDRTTYWAAGNPISGQDCVYGQQQSGFNSLWYSAPCTDPLKNSMPYACQLRPYDEPAPALCVYDPQKPISSTQKGIHKQVHCFVFHINAAHITATIKSTLSGLTLGTGLYQSNIAVFAYSETVQIVVDFGSFKSSEALAVSSLQYFLSPVLGSIKQASSMISTNGRAFTKGVIVLLTNSYDEHQTVDIAEAVNAYKNHGGIFVSIVYRLMMIHILKKSFPLVKLILAACLAIVSIHLFNYFALILFFKCQSSARMRLALVLSSVVATAAAAVFNRSAARIESGVAAGNPQCREPFFAYSTGCVYIENGNKHTYQQAVDLCSSLGSFAPSLHSKQDLTFWSDMMETWNVYPYHFWLDAYCPSAGEKYKWRDGTETDYYGPENELKSCDPNIGFHVEWNGVLGNEELDQLAPALCVYDPSLTTLPPTIRPTTPKSSDVPSEFCSCNPSTVYLDIVFVVDASDSMTKDLVGDVTATIKSTLYGLTFGTGHSQSNVAAIAYGETVQTVVDFGGFQSSKDISSFSLPFLGGKSDKTAEAIKQASAMLSANGRSFTKGVIVLLTNSYDQRQAVDIAEAANLFENNGGILVSIDYSEGSGLAGLQDVASAGYYINDTASNPSLDSDMLYAFCDANCFCKDGLTPYTVTNERGRNAPDGCYRVAGFSAVYQAAKENCDEEGGIVSTVHDDFKNFFLMSLFPSKTKYWLGLQRTADGFTWADGSTDAYVNFAPGNPAPGADCVYAQQQTGFNTRWFSAPCTDPLKNSLSYACQLRPCGSDYDCSA

>Ppa|PPA09328

MSHAMRCLAVFVVLFGLAHALVKFDYSVVLQAADLDPFSNTVLFDCYTSCKVYVDRKSDKLLISHNGRVIADFTKIIGDSAFNPAGFEIAAGTYKLENRGEINPVFVFYIVDSEAANVNTQVYVPTETIGISFKGHDRYVTVLSSFDALEFSAFQGTFPAGYPRIYSTGFDAAGDSRCHPVYQARSQDNAEISLPRVASAVITVDFGFVGAHNVSVNQKSLDNPRKGQGASTVYMSPGYVGCSFNAGHNYYANVSEVYDYYQSFMNEFNLVAVYDSLTYNEPLQITMGGVEQALTGSSRYEVHNPSDALVPSYTVLWSRTTPSSSFAVQIEWNIDEAQPTIPTTTTRRAVQTTTTRAVPKTTPVPWQTTTFGAPSTRNPAIGDSYCSCAVDKFGMPHGWLSTEIWLDVVIVLDTSEAMGAESLEDAATLIESLIGDDDYDVLITDTRSQFYTRIGVVVMSDTAEVLFNLNMTKSDTITDKVHVAKGVTKIDVMHAFVTATDMFANGLITKPDRANTRQVIYYLTDSDAKLNATQIKDFKERGTIIVEQFAKKGENDKDLPQLASSGYYFKTNEESPIQNFCKANCFCAPDRSPYAGPWSDAAVIAGGGCFHAPTIGLQFGKAQKECSNNRGSGELASVHDKHKALFLNELVSDSTNKPYYFWIGYSKNDDGVWKWEDGSSDPYTNWDYNEPSSASVSKCAYVDQEQENLPWGAGNCQIVFPYRLPMAMMTDDLRMVKLDEGLS

>Ppa|PPA10019

MKDLEVVETIKTRLSFFRNCRLFKSSVHRFKNGLLILGFLVEGRRSDCLAMDTLSTSGQWMNMNCSASLAAACAGQENPKPVCSPGPWVEGQTIFSPGYPYDASVPCDYFLTSSLLMKGREWRWSDFEIHQILLLEANSCCDRLILTDDVLVGKIVANLTGEISDKTYTTSTSNLMRVSWQPNGGVNVRGMMNLSLVHALPGSNWGLYATMTMHFDDSIDPVIAKCAEIQGTPVIIHDEEHNTYYQMRVADSHHLAIFDIYCTTQLQQPTSPDFGCYDFEEDNEDGICYQVDANIESFKDAQKLCGQYGANLASIHNLQENSFIRRLAVSAGAVNGVFLGATKSGKGDSFGWTDGTTWDYSNFYPGFPVEDRGDCLAMDTLSTSGQWMNMDCSASLAAACARKENPKPVCTSGPWAEGQTIFSPGYPYDASVPCDYFLTVADGKRVQVEILLLEANTCCDRLILTDDVLGGKIVANLTGEISDKTYTTSSSNLMRVSWQPNGGVNVRGMMVDTVQCSCPPGFELASQGECRGVTARTVVVYTDDALNTVKAYCDTVNGQVPIIHNEEQNSYWLSQQNDRFLLGLKCNSSTKKWEWMDGTDVDFKPEKYSQVSGHSKTTTDVYCTTQLQQPIPANDGCQNFEDDEEDGVCYHATLQILIMKTTTLLQEAVENWQVAQMSCRNFGGNLASIHSLKENSFVRRLAVSKGAVNGLFLGASVSGKGNSFAWIDGSEWDYDYFYPYFPLEGQGDCVAMDTLAPAGERINKNCSASLAFACVRQRNTKSVCPSGPFKEGQIITSPGFPFDASTPCEFILTVSPGKNIEVEIHVLEANSCCDRVILTDDFVGGNIIANLTGEISDKTYTTSTSNLMRVSWQPNGGVNVRGMMMTFREIAHCACPDGFDLVSNGECRGKLKTMDIFWDTWMATTITECAAIQAQPIIIHNEEQQSYWASVAGQSSTKHNNVILGLVCNSTTKQWTWSDGSRLDYKPPNYTSDLDKNCCDGCTWVVEPGNFWGYACGHSGTMNRDIFCTKQLQQVDPDANEGCLYFDSDSDDGVCYQVGENADTWQDAQLNCKKLGANLASIHNTQENSFLRRLAVSNGAVNGLFLGGTYSGKGDNMGWIDGSVWDYENFSPGFPKAGFGECLAMDTFSTAGQWVNIDCGAKLPVACIRNELTIVVPNATCSSNTWKEGQIITSPGFPFSASMPCDFYLTVDSGKRVEVEIILLEANSCCDNLFLFDGYLGGSLLANVTGQVSNVTYTTTSSNIMRVVWEPNGGVNVLGLVMTFRVLPEQTKNLYNGQSQISETITVEEVDLEAALNYSCPAGFNLVRAGQCISYGQPFQTRSEDLVQRASKTCIDMGGQLIVIHDNEDQLYWIAQYKTNEILLGLACNSASSKWEWVDGSLVDYKPHSYYDGTLDKSCVKNCAWTLLPTGKWEIRCGEHDYLTVNLYCTAQLTPPPVPSGDGCEDFEVDNDDGVCYQVFPTIIIKWKYAQLLCQSVGANLASIHNSNENAFVRELAVSKGAVNGVFLGANRSIALPHFQWNDRTGWVYENFYPGFPQPDFGDYLVMDVPTGQWMNMNCGSEMPFTCIREKRSLPPPSCRSESYERAGMISSPGYPFNASMPCDYFLKVDAGRKIITLEANSCCDYLTLYDGYVSGKVIANLTDVVRDETFTTTSTNFMRVSWKPNPGKYGFLESYWDEQEKVAMMKCMELGAMPVIIHNDEQQKYWQLHVDRKGDEFVLGIVCNTATMRWEWADGSQIDYRPPQHDPNLERNCITDASWRQQLNEAGHIDDGYTDIFCIKNLMQPIPSAEGCDSFADDSTDGVCYQIGGIAMSWPDAQKICRGVGANVASIHTVSENSFVRRLAVSKGAVNGVFLGATTVGKGNDFGWIDGTAWDYENFYPGFPVAGAGDCIAMDTSSTTGQWQNFDCSSTSLPVACIREQKPVVEPMCAGPWNEGDIIILLEANTCCDSLVIYNGNLSGNVIANLTGEKMNATYTSSSNSMKVSWQPMGGVNVRGVMENEMINHIANSMDELKERDMNLVLDLNCKNSTSRLEWADGSSLSYLPDSRLEVDFDCVHAKRTLFSKPRDGQWYHVLTDYNMGWTFTESDQILGGGRDL

>Ppa|PPA10028

MGMKKLVLLLFSILQIIHASCPGGFALVRDGQCRGQYSTITKPWDDILTTSISKCSEIQGQPVIIRNEEEQSYWSAQKSENQYFVLALTCNSKSKQWEWTDGSALGYKPPKGQYHRALDDRCSTGCSWLLTTDGSGYWMQGCGRFSTLTVDVFCTTQLQQPTPADDGCESFEDDKEDGVCYQIGEIAQNWRDAQRICRNFGADLATIHRKKFQANSFVRRLAVSRGQVSGLFLGAMSGSDNKFGWIDGSDMDYNNFYPGFPIAGLGTCLAMDTQGTSGQWVNTDCSTKQSVACIRPQNFPTPACTSGPWNEGDIIFSPGFPFDASTPCDFLLMVDEGKRILLLEANSCCDNLVLFDNYISGKVVANVTGEINDKTYTTDSSNFVRVSWQPNGGVNVRGINVCAHFALASCDMLGLLVFLSVLQLDLADVRACPQGFDVVANGQCRGYYKTLNLYWDERAVNTAISSCGEIDALPVTIHNAEQNQYWAARGWLVIGLVCAKGETKWRWTDGTPLDYRPPKYHAALDKQCKGGCSWDILEDGSWDFICNGQQTYKTNIYCTTQLDQPFPSPDGCDAFLDDRDDGICFEVAKTPTNFQEAQKICRSFGGFVASIHNDRENSFIRRVAVSKGATNGVYLGATVAPNAQSVKWLDGSVWNYKNFYSGFPLANAGQCVVMDTQGTSGQWVNTDCNAKQAVACERRQYYNDTTCNAGPFKEGDIIYSPGFPMTSNIPCDFLLSVDAGRRIAVEVLFLEANSCCDKLVLTENYIGGPVLATLTGEVSNKVFTTSSSNFMRVSWQPRGGYNVMGMMEQAHVVEYVTSRDLYWYERAVNTAISTCGAMGDDVDAVTIHNAEQQQYWTERGWLVIALICANGETKWRWTDGTPLDYKPPKGMYHSALDQQCKGGCSWDIKEDGYWNFICNGQETFTTKIYCTMQLEQPKPNPDGCDAFQDDRDDGICFEVAKAPSAFNEAQRICRSFGGFVASIHNDKENSFVRRIAVSKGATSGVYLGASVAANAQSVKWLDGSVWNYGNFYSGFPLPGLGECVAMDTQGTSGQWVNVDCKATQAVACERRQNYSDLSCPTGPFTEGDIIYSPGFPMTSNIPCDYLLSVEAGKRIAVEVLFLEANSCCDRLVLTENYIGGQVLATLTGEVSNQIYTTSSSNFMRVSWQPQGGWNVMGMMVTFRAV

>Ppa|PPA10030

MLTLLALASVLNFALVDANLCPDGFDLVANGQCRGLYKTLSLYWDERAVNTAIHTCEEIDADPIMIHNEEQQQYWTSLSQKGLELVIAIVCAEGETKWRWTDGTKLDYKPPKGMYHSVCNGSQKFTTKIYCTTQVEQPKPSPDGCDAFQDDKDDGICYEVAKVPTDFQEAQRICRSFGGFVASIHNDKENSFIRRVAVSKGATNGVYIGASVAPNGQSVKWLDGSIWNYGNFYSGFPLPGAGECVAMDTQGTSGQWVNVDCNATQAVACERRQNYTDLSCPTGTFTEGDIIYSPGFPFSSNIPCDYLLSVAAGKRISVEVIYLEANSCCDKLVLTENYIGGQVLATLTGEVSNKVYTTSSSNFMRVSWQPQGGWNVMGMMVTFRAV

>Ppa|PPA11288

MVQHKPSIHYIILVYSKELCPKDLDSNRDDIMAHGTCEEVPLKRELVLKDAYSKSDFFWIGFSKSDGVWRWEDKSTDSYTNWEVDEPNSAAVARCAYVDSSTKALAWGSGNCNVAFPFVCEDVPCSVGNKDC

>Ppa|PPA11506

MLQTTLLLSLVIIHATLGCTPPYNTWIDGNCYYSYSSYTTISWPAAAENCKGRDATLASIHGSQENADFLKLAKEDTLQETKRGYWIGLRCDGKKFYWEDGSFVDYTNFGDSTYKCNTTSTDLHFYMNWDDGKWYRDVNWDWYGRAYVCKKNYRPEDSICEEYELVSDTKTCLSLRSDSMDSKDAENSCTYTGGHLAAIHDNTVNDYIRRSAVSNNLLNGVLIGLKQSGNDLTWNDNTTVNYTNFAKNFPNSSLGSCFAMQTSSLAGEWVNVQCGGTSATPTKLPYACTMPAYDLPDEMETSECPIDTYYSDGDMIYSPSFPSPNNTNSCEYLIVGPEGAKNMQVEVVFFETNRCCDSLTIYEGIAGAQKIATLAGSTYNGNIYKSANGPAMRLVYNVQSGAHVRGWQLKVKAIK

>Ppa|PPA11619

MGKWIHTTPRMRLILLLSCLPALTNACAAGYNTVYDGLCFKNFGSGVHYDVAKNTCDSDDGHLPRLYSQAEQDQYWSVFHGYYAWTGLICDGERFIWDDGTVATYTNFDGTVTCNSGNTLNTYINPSYGKWYQSDGSQDTNNAVCAANTRSWICDTYYLLQMGKSDDWCYLFITSLTAQPAAESNCLTQRAHIAAIHDKDFNDYLKRTAVGYGITNGLLIGLKFVNGKYEWNDGSEVDYTNFAPGFPDESFGECVTMGINLKMDELGLCHSTSIHIFSPTWGSATGPALCDYALMDLDKTKKVTVEIVFFESNSCCDTLTIYDGLVGSTVLKTLSGYYGFTSIKVTASANAIRMEWNAKSGAHVRGWHAKVTSA

>Ppa|PPA11856

MARNAEKAMTTLARWRRLKEEEEKGPVAKRPHDTKYKLRDMNDDINKMLRIKMAWEWRIKELGGPDYRKIAPRQLDREGREVSSNRGYKYFGAAKDLPGVRELFEKNTEMEGVRKTRAELMKNVDADYYGYMDDDDGLLVPLEKVAEEKAKLAIKKKFDEQGGERLKKEHEDDIDIDFYKVEEDSEENGMETRESIVVGEDGRKMTIRHVMVPSQQDIEEMILERKKQALIDKPDCFEREMAPAWIAPRNANHVKVSSVQECLAICASAHSILQWECTRIVYDNINQGCLLFGSSPGEGEKMGVDPSFDAYNNLCWMEETTKNEVNFPDKDDEEEEISLGNVQTVEDLKKNLVSMKQIDSIPTTVPSIPSIVPTEQTRKCFRRIPQGNIPGKADDIYQNISSTECLEKCMECNDCLGDDLPCMSVIYYEHVSECILLRFKEENGLSRISPIQETSSLFWRDLTCGGTNKCNPHMDLIIILDGSDSTGIKDFEKGLKVIRTVMNTIGKLTSDARVKVFQIIGGRSLLGATVSQVIKNTQSDTSIFILISDLFIEDNPKLIEIELNKYDRQMITLSLTEVFDTKIGDRLTMGRTGKMIHSIGEEEAEEKLLRLLCEEREKRGKKQTNLLETRRVWIGLRRTVEGELLWTDQSTVNDLSRLSDIPTDDKKCVSSEIEWSYTECEKEFPFVCTFTPLEKVVMVKNDKKGAGPLHQKGPVTVSALQNDHITEIARKNWAPNTKDKDADFSKDLISKIYNEEVKASKFNPRKIVLLEFSQYLECYLWPNFTPERDSNEFIMSIVVMVNEKFRERIPAWQSFNKNPKHFNFFLHRVLNLALDETQLSMIEHCSILQFLVNCFNSVEVDLVREQITRLVHIRIWNNILPKQREDMLSKNKKLRKFWNQTEAKAKTAKLTEEAAKMQEMEQVFLWKMIQRFKRTLDRIDDENSEIDVDDIRYCERFVQLMIDLEALLPTRRFFNAVLHSSHLLVHCLLSSFIKSEAGSLFCQLVDMLKFYARFEIDEITGLPLTHTEVATRHTDHVIQLQKAAFKYFKESMKDFYLLNVSGVDSRKALTKQFTSMSKSELYRFAEFLHLVPSVEEGDEKKTMDGMEKEYLMEALILHCERRVNQLQQLNEQPLYPTETVIWDENIVPYEHYSGEGVLALNKLNLQFLTLHDYLLRNFNLFQLESTYEIRQDLQDVLFRMKPWLHETNGNCVWGGWARMALTIDEFTIVEVAKPLVGEKSPAVVKAHLQLNIGKRMDIRGEWESLRKHDVLFLVTCRPKSAIGTRFNPRMPFKDQIEVAYVRGAEVEGMLDPHGNVIEEFEAYEKKPTVQGDVRRWRITLDPNQYRLDMENTVEKKTEDVYYTFNLVVRRDPKTNNFKAVLGTMRELLNTECVVPDWLHNLILGYGEPDQANYKKMTNVVDTVDMNDTFLDFDHVKESFDGYKVVGSEKDEKMLPPFSLSFKDLTTVEGMRSKERTVEVRPLSRVPVTPYSHKTNKNKIRFTPAQVEAIKSGMQPGLTMVVGPPGTGKTDVAVQIISNIYHNWPNQRTLIVTHSNQALNQLFEKIIALDVDERHLLRMGHGEEGLETEKDFSRYGRVNHVLKERIRLLGEVEKLARAMGEVGDVGYTCETAGHFFRYRVCRAWDEFLSSSKLDEGEGRDIVKNSFPFTAFFADIQPLFSGVNESDSRIAASCWEHISRIFSQLEEFRAFELLRNGRDRTEYLLVKEAKIIAMTCTHAALRRAQLVQLGFRYDNILMEEAAQILEVETFMPLLLQNPQDGRNRLKRWIMIGDHHQLPPVVQNVAFQKYCNMEQSLFGRFVRLGVPYVLLDRQGRARSDIASLYAWRYRALGNLPHVEAIPAFQRANAGFAFTFQLIDVPEFNKVGESQPSPYFYQNLGEAEYAVALFTYMRILGYPSEKISIITTYNGQAQLLRDVVQRRCADNPLIGMPHKISTVDKYQGQQNEYVILSLVRTNNIGHVRDVRRLVVALSRSRLGLFVLGRASVFENCFELTPAFEQLSRRPKKLLIIPSESYPTERKVDEKSSDKVIRIDDTAHMCNFVHEFYNSNVEMLAANYQAAMDEQERVRMRFLPPPEEEPMEEDEDVKKAEEKKIKEEKKKQEEAEDIAFEEMDFQRLENMGDNNLPVEVNGLSVKENGDAPEENGDIVTPWDVSSGSATGVDYDKLIVKFGCRKLESDLITRLEALTGKKAHPMLRRGMFFAHRDFASILDRYEKGKPFYLYTGRGPSSGSLHLGHLIPFMFTKYLQELFDVPLVVQMTDDEKFLWKNMKVEEAKKMAVENMKDIISVGFDPEKTFMFQDFDAPFYENIVKIWKCVTGNQARAIFGFVGEDSMGKAAFPAVEAAPCFPSSFPHIFGKKTDIACLIPCAIDQDPYFRMCRDVSHKLKFPKPALIYSTFLPALQGAQTKMAASDDSSCIYLSDTPNQIKNKIKKYAFSGGRQTVEEHREFGGNCDVDISFQFLKYFMESDERLEEIRQQYTCGEMLTGDLKAIAIAEVSRVVMEMQERRKNVNDETVKQFTTMAPDLERKSSQESSSSEKGDIITPWDVSAEAPTGVDYDKLIVRFGVKKVNNDLIDRLERLTGKKAHPMLRRGMFFAHRDFGLILDRYEKGEPFFLYTGRGPSSRSLHLGHLLPFFFTKYLQESLDVPLIIQMTDDEKFIWKDLTVEESKRMTKENIKDIISVGFDPEKTFIFQDFEYIPPFYENIVQIWKYVTGNQARSIFGFIGEDSMGKAAFPAIEAAPCFASSFPHIFKGRKDIACLIPCGIDQDPYFRMCRDVASKLGSPKPSLIYSKFLPALQGAQTKMAASDANSCIYLSDTPKQIKNKINKYAFSGGQPTIDEHREKGGDCEIDISYQYLTYFMEDDQRLEEIKKSYSSGELLTGELKAIAIEEVTRVITAMQSRRALISDSVLAEFMSVRPLKYSF

>Ppa|PPA12964

MSTTSRLATVSALLIAYVAANCDPGWRYLPSTQSCYKLIDDQLPWSVAEIRCLYQGGHHVSIASYEENQFVHETARHQEVWLGAAFFGSDYSYVYSDHTPFGYFEGWEFGGARPSMNRARRCIKINDRGDWFQSCCKKLAATVCKKAATPAWGNSNDLRA

>Ppa|PPA13083

MINIIQITYPIIHCSCPDGFELVAEECRMLEPITMSIRDDQATDSAISQCNAYLAQPVTIHNDEQLKYWIEKKGSNYMPLELLNDCKSGCVWFINNSTGYWGEQCNTDVRAFDVYCMAHLRQHVPSGDGCESFEDDSDDGVCYQVENMAIKNEHLPLSPSPRVPGVNIFKFGALAENWKEAQLLCGSFGAQVASIHNEKENSFIRRLAVSKGAVSGMYLGASVSGKADNFSWIDGSAWDFNNFYSGFPINGLGDCIIMDTESTGGEWANVDCSTKMSVACERKREFIELKVILLLPVQQMYSPGFPFDASTPCDFILSVDSGKKIELEPKTGEVHDKKYTTSSSNFMRVLWQPNGGNNVRGAMVSEIASMNRTALKALQGKNSDYATIKKDLSLALRVGMNNCDNYNEQIQLTTSKVLYTITDLLLAGVENIEAGKQFDLTYKLVDAYSTMNKCQNPKDPHQVCLFKFARCFILFMDNLHGKKLLKGEEAPEKKLIPSVWVDSEVAKRNNPVKQLPIRSSKSTSVGIVRNIGAQQIVIINDRPGPSGCSQTEPAPHLPVPVVVTKRRLVPAKRVMPPAPSLVKDSPALFEDPETVKKGETEEEGVAWKDMGQQPSTSAHNDAPPSFLSHPPKEEEVEDRIIPSTTYHNAAPSTSSQSIKQEVEDNYDYGDVKMEMEIKEEDEEEMDGPIADTVKLSELCSSTNGFHDPTSSTSQPSLSSSSRRKQKMTLSPPPPECAGFATAEAQKLFFQSPVNCPYCGVEIPDGKKLEQHMKINHRDHWLKYVQKCPVNICDFRSSDPSVVQRHHVMVHNRNYNTRMGNAAVNFKFVATCPFCPDPLRGLAGFVQHMEKKHPRLCTYEAKILACAECRYSTSNVYMLLTHWLKTVPMCTQGLRFNYEIAANTKINDAIDHEETVFLLCNAKFDAALSFSRDQTVKMAIQLCDELPALPQNQYYMGELPPPESTPRPIVLGLMCNPSTRKYEWADGSPTDYHPPGFSFDEACNPDCAQYMADEDGRWSTWCGPDMERVTIYCIYQLPEPAPPPSGCANFDDDTDDGSCYEVINAVEDWQDAQVTCRNIGSDLASIHNERVTELRCFFCFRKYTDCEKLEENSFIRRLAVSQGVVGWIFIGGAVTGKGNNFGWIDGSEWDYENFYPGYPVDGNGECLAMDTRVNGRIWTAQQIWLLCAGGEIVSPGYPYDASVPCDYILSVETGKRVKLELQILEANSCCDRLILTDNLIGGNIIANLTGALSNRTYTTDSSNLMTVSWQPQGGLNVRGMMRIFIFKSLLVLGNYNSDLAHSSCPSGFELVRDGECRGRYTAVNSNWNDISRTAVTRCKEIQGKAVIIHNDEHQAYWRNRVPSETLFMGIVCNSSSMRWEWGDGSSVDYRPKEGYMEELDAECKSGQSWDMHDNGYWHIGEPFHTDLLLMSRFLAYGTDMEYSLSIFCTTQLQQPTDYGCDSFEDTEDGMCYNILPSVQNWQDAQNSCRNRGSTVASIHNLQENSFIRRSAVSQGAVNGLYLGASFYSGRFSWIDGSNWDYENFFPGFPVSGQGDCLAMDTFSGSGEWMNVQCSSRLAVACARKALRIRYDFVMNRHKNRFSRPEARVHRWAVIYSPGYPYDASVPCDWFLTVPAGRRVRVQIMLLEANSCCDRLVLQDATLGGNIVANLTGEITDRVFTTSSSNLMRVSWQPQGGVNVRGAMFTFNAV

>Ppa|PPA13091

MSKLALIACFFLAAHAVSGFSVSSPKPAVSGAPLKTSPAPVRPSGSPVGSTGFFNPTTRGFGNPSVKPVTGGPVSGSPKPAGSTGFFYPTTRGFGNPSVKPVSGGPVSATTRGWLVSEANLRFSSVSTEMRRLIVFLLLLCIVNATCRSGFELYRNGECRGNYTRMTPYWDASPNPSIASCKQSQALSIIIRDAMDQTYWSSKATGGYALLGLVCNTTSKQWNWADGTVLNYKPPSGYATDLDKDCVPSMNWAIQSNGYWYSGAAHNTFTADVFCSIPAPQLVPSPNGCESFDDDSDDVICYQIATAAENFRNAQMVCKNVGGDLALIHNDRENSFVRRLAVSRQAINGVLIGGLLSGNDKAWTDGSTWDYDNFYPGFPISGLGQCLVLDTQGTSGEWMNVDCNNTVAVACERQQNFTTPVCPTEPPKEGQFVVSPGFPFDASTPCDYMLMVDEGKKVQMEILMLEANTCCDRLVIYEDYFVTGEFKERVYTTSTSNFMKVSWQPNGGVNVRGMMITFRESILSSCPEGFELVRDGECRGFYSTVTGIPPSQQLDIAHQTYLTRHVHDGSYSKIILGLMCNTSSTKWEWIDKSPSDYRPPADFVSALDEDCELGYTWFLTDNGAWDYYAHSTVSMDIFCTTQLQPPVPSGDGCDGFDNDIEDGVCYEQENSFVRRLAVSKGATNGLFLGATMSGKGKDLGWVDGTDWDYDNFHSGFPMPGLGDFLAMDTSISAGQWMNIDCAAQLPVACIREGERAIEQPTCSSGPFVEGALITSPGFPFSASTFCDFFLTVEAGKRVEAEIIVLEANTCCDSLVLYDGYLGAPVLATLSGELSNVTFTTTSSNIMRVNWQPQGGVNVMGLATRSSIVSSRLRVDMRRLLLLFFILQFVRGACPDGFELTAAGQCSLLTPITVSNVAYTAATNTAISKCKEMQAQPIIIHYAEQNSYWASQFNKNLVQPLGLVCNTSSLKWQWTDGSAVDYKPAAGYFAELNSNCKTASLWYMASTSGYWYYTESTATNTFNIFCTTQLPHPIPSGDGCESFEDDNEDGVCYQVGASAENWQEAQTICRSFNANVASIHNLKENSFLRRLAVSKGAVSGMYLGATMIGKGNDFGWIDGSEWNYDNFFNGFPMQGLGDCLIMDTEDTSGQWVNVDCSSKLAVACMRQQHYSTPSCTSGPWQEEQIIYSPAFPLNASSPCEFILTVDAGKRVEVEILLLEANSCCDNLIIYENYFGGTIIANLTGEVGDKKYTTSSSNYMRVSWQPTDGKKCEGSDVHIPKPVILPTAFTSNCPDGFILTAEGQCSQTSPIVVSSREDEAIDLVISMCNEKHAYPVIIHYAEVSTLFYPPVRRDLLMFSNCELENKIRLYQLTLPCKNGCIWFVNRDGGWDSWCFATIHDFDVYCSIQLPHPEISEDGCENFEDDTEDGVCYLVGPNAETWSDAQLNCKKLGANLASIHNQQENNFLRRVAVSRGAVDGLFLGATISGKDDHFGWIDGTDWDYQNFYPGFPLPDFGDCVAMDTSTPVGLWMNFDCSSKLPVACIREKQLNVPADYTCSAGPWAEKRLITSPGFPYDASTPCEFFLKAEDGMKVELEIVHLEANSCCDFLLLYDGYLSSTLIGNITGILTNVTYTTSLTNYMRVSWQPKGGVNVMGLAVTVMKTIIVSKYHSDEFPKSMAHCACPDGFKLTAAGQCSQITPTLISNVRADQALDKAISTCKEIHTQPVIIHYEDQQQYWITQFKSTNIVLGIVCDTSSLKWQWTDNSPINYRPTTHYDSALDKNCRTGCTWGLWDKQWEYYCDGTTTSYNVYCSVQMPQPIYSEDGCEYFDDDTADGVCYKIGEDAESWQDAQLNCKKLGGNIASVHNQQENNFLRRLAVTQNAVTALFLGATISGKEDNFGWIDGSSWDYQNFYPGFPLPGFGDCVVMDTSTTAGLWMNYDCSAKVPAACARDKRTNLVANYTCSAGPWEENTIITSPGFPYNASTPCEFFLMADGGKHVEMEILHMEANPCCDNLVLYDGYVSGSVIANVTGIQNNVTYTTTQSNYMKVSWQPKGGYNVMGLAMTFRSVGAREHAIFGEEINGKTYIPNADVAISFCPPGFDLVRGGQCRGFYTTVTVPDDVAENIAANKCKEINGLPIIIHDDEEQAYWKSRATGAYDLVLALLDECGLTARWLIIIRLLSFPVRPVSFVQSSLDEECSSGCVWMQGKDGMWREWCDDDQDTFQVYCTTQMPPLPVPSPDGCDGFEDDGDDGACYQVSTTAESWNDAELICRKLGANLASIHNDHENSFIRHLAVSKGEVRGVFIGASSGGLGEKEADFAWVDGSPWDYSLFYNGFPKDGFGDCLTMDTSTTSGQVDAEGNKAGMSC

>Ppa|PPA13095

MQNSYRAHSSIVSARVRVAMRGLLLLFFILPIVHSSCPDGFELYSGGQCLELQPTQWSGYRADQAVSPAIAKCKEKQAQPVIIHNSWASFVLISVMLTLQHNYWENQYSSHSNTVVILGIVCNTISLKYQWSDHSAIDFKPSSYNSVCDSITYTYNLYCTAQLPVPIYSEDGCEYFDDDSDDGVCYKIGAAAETWQDAQLNCKKVGANLASIHNQQENNFLRRLAVSNLAVDGLFLGATISGKEDHFGWIDGTDWDYQNFYAERQNLPTNSECGTDPYLEDTLITSPGFPYNASIPCEYFLMVDEGKNVELEIVTLEANSCCDYLLIYDGYLGSSMIANVTGAVQNVTYTTTQTSYMRVVWQPNGDVAISFCPPGFDLVRGGQCRGFYTTVTVPDDVAENIAANKCKEIMDCRLLFTMMKNKPTGKAAPPALTTSTSMQFLSSVDMGRRLVGRLSSAFVHSQTYFDTHFGESIGKDLSGSHWCDTDVEPLDVYCTAPSRQPIPSGHGCESFEDDSDDGVCYQVGAAAENWKEAQIICGSFGAQVASIHNEKGNSFLRRLAVSKGAVSGMYLGASVSGKADNFSWIDGTTWNYDNFYSGFPITGLGDCIIMDTGSTGGEWANVDCSTKIRLYCSCLHNRSLGGRADNVFSGISIRFSIATTPCDFILYVDSGKKIELEVLLVEANPLTGEVNDKKYTTSSSNFMRVSWQPTDGKNVRGAMFTYKAV

>Ppa|PPA13470

MLLIVLLSFFPLIAAQSRRECAEGAKYCDSAGGACSLVENDEWTYFAQCECYDGFDGPRCDKRIAPGDSGQRYHCEGPFHAECPKGQTLRMDYASFGTNFASSTCAFAKHTCTDDRSLAVMRQECDGRQFCSIENLDYAFPNNNCTGKKSLIYRYRCTSDALPSACPEPNSIRGSDRCFLFDDTTGKIDYREAYEVCRRKGGFLASPITARDHQQFIDAGIMNEKRSRAWIGAISNNKAIPSWNDGSNLTFVPRDSQLEWANACLSYTTSAAIPNSFFWVSENCDQLRFGLCSFVPGTTPTQPTVPIPGKSKGKVEHDLALSFTNTIYFFCPTRAKSMTKRGSLAPVANRCDEKSPSLTATVDRVSIGDSVPRRPMPFARLFESAAHCEETTWRGVIFPRTRACDTVIVDCPNPDLIEGREQSVVRASAESSRMGVREMRNAVSRGREAEIVSGETANHLKTTLSTQLYGGDITGSVDLTRSMLSLARGQYNSLDDRVVRQTRAQNFTQNLGDCGDSLLEGRALPVWEQLPSGARINQASDLMQSLEESAILLADYSYIEKQAIDYDHWAMEVEVRRTVQPQFGGGGGEGAAGAFEAAAAAPPAAVFAPTSPFAANADVMDDSGFTARGMAGRQPVSSNDVPLPKLPEYQEQKPSDPVSFSTIRKSPIISLPSTSTLSSSAGPMFSPADSPPAGPSFFSPASSSGASFRVLNFSYFVFSSLGAILSNETTRVNSQVIGATVNDASRSVQLPDSEPVTLTFFHLHTSGVANPRCVFWDTFVSDWSTRGCQLLMSNLTTSVCQCNHLTSFAILMDITGELSQLENTALDVVTVVGCAISIVCLLLSFLVFSCFRSLRGLRSTIHANLCLTLLLAELVFVLGVSRTKNTVACSVTALLLHLLFLSAFCWMLLEGVQLYMMLVQVFEPSSTHIALLYLFAYGLPAVIVGVAAGVDWKNYGTENYCWIDTTTPTIWAFAGPVAAVVAINIFFLLIALRVVLSISSRDRSRADRLRGWLKGSATLLCLLGITWVFGFLTAVHGASVVFAWIFTLLNCTQGIFIFVLHVLMNEKARTAVSRWLRHGWCCFSNAESAANYNSKEYVTSRQRFLNMIRSTAADTGDNADETSGADKRQKRMRKEDTTSSTGPSTASTDTKEKGPLTPTSKTSAWLSQIPSQDDGNDYDLPPQSPVEKEEIKDHKEIKDQREIKDQDEEDRPPPLPKSPPPPLTPPTSPRLQYLEVPPLPPPDYDDDFIVPPLSRSTRSLDLRETDNRTPVRRKKFPLGATDEERGAAAAPLHLEQDQEALSRL

>Ppa|PPA14572

MRIALGLAVLAVLACAASGAFRGQFADLFNTWRAGEEAHYVKDFDHFSAAYHPEVHSEEHTRKKRNAEEAAKFAAGNPITKACDRPGYTGQYCEFPICQEFNPFINPEQYLRDDGYVIDLTDLGNCTRKHEIIVDETMFDIHIEVQSLEDVSPVLSIYDSDGYLGTPDETVAESDRFVATFKALKPGYYTLVPSAASIESRCILTTTAQTFMTISGGFQTDDRDRNDFPSDNAGAHQFNSIMLHLNGGRSPAELKTVSVIGPENQVLRPRMLDKRYGCQYEYYFDSLFCYGKGSYAMIVEGVDFYGLPFRRTAPFQCVYVPAPPTSPGPSTTPVPTNPPACANGGVMMFEGLRSSCVCQDHWTGYDCSQPLCINGGTLIEGKCFCTNGFEGVHCETVRCEPNSNHGFGVDKPTLIFVVRVREEMNAVMQQVQQAVDETFIICTSCSLLISYSFSETQQGKSGADRDISKGGRKMRSHRDKQHNVITPEDMFDALNATLQLRATSVFLEELVENSVSSTKIKKITELSYFEYHGSDARVWKFHGIGDGEVIKDLKHTNATLDIKKQGGKLAPAAVNIEDRKRILASFDKNPGQYEEPTFWLLPHEVAPMLDIEPNARDDDIVTPNRPDPSNPAGAAKQSLFYCRDCGSSFILYKNLLKHIEKGKHFIRPEHVKLLDKVLGLFMRAIEDTLVPEPLSPVSEVVKAFKRASDPELPQGWAIKHGRKVGRYSEATKAFVKAKFDEYAKRGAKLKADEAERLMRADRFIEPKDWMTKSQLRNYINSLKSQLPKMRAWRRQVEHEDMDDEHFEVEVEPSDEDIVITEEDFHRHLTPTMLKKFFSDVDKPVVANLQFDPAYLTRFQVVFFNDHNIFMSQHYTSIEAFDIGFYKATISQNTEGGCTDGVLGAVTTGLTNIALTQGSTIYVITDALADDYDTAFEALLQFNSYWRATINFIYVEPTAESQCDSDLSDPGFRAFDEVANRFGGLAWHVDDRSKVYDVLYGHMNSIIYKSQLMLTLDRDECGNGLGKVIQMEKSSDTLVFIAKGRDFTLEIIAPDGNTLQREHIVDQGLFTIQRTADPIAGAYMIRTHTKAPTASCSVRAYQASYQSYSTDSPTEAFWAITTDVDADAWLYQPLVGIDNHPVFHIENYGESEDYDHAFAFLNMYAVREGVEKEVYASNGLFRGGCSFHFYFPAFRCRPNENLHYEFNLRTEEGFYIQRAGVMTCFNSFPTPAPPSDCQNGGVMYNETCLCQPHYEGAHCESVICENGGTPYFGICQCAAGWLGPFCNTAQCSESGPVPNYGYHVDMAFLVEVTKSGVKQIQQLITSLPEIIRDINSQHPDWINRLVLIGYDSDKVIGMVDTPMDNTKKFFDTLNQWGNSNPVDDGCIVKVWPAIDQLLNGRLDGNQQHELPYRSVVNIFETGQPSDLANTVNLIQTSEEFLERKTLTNVFQAKDEATGGWRCNGKNDDFVYIEQLARRGDGKMYTLSNADLGKAVRMIPTLFSSAIVYKYHTEDCTTVPHNIYFPVDAYTQTVSAVIAGYNAEVKLFKYDGSQFTSDGRIDILSDDRNQVVEFRNPCDADWDSVSQYCMYFNSALVKNFVAGNELCQHMNGFLADDLSNEKNEWLKSAMNGQKAWLGLTFIQGTWYFQHDAGSSLAVPGNINFWVDGKIPDGSSGTCAYFYNGLWYPASCDEKHLVVCQKHMFDSTNEPSNIDDDDLAPGKYYLTVQTGLEGPWKGCDVENRVVSSIAIGKEETQTSILQHVMLRSDDDSSLLLEAATYSYRFGCRQPLSCEQTNGEDFSVVMIGEDDTGNTFQRYSTSLCYKWYVCANGGVYSNGQCICPDYFTGDDCRTPMCQNGGVPAVTGKKCHCQPGFGGDACQFVQCDADSGASFTNDGKALILVIEKSDNTADAIQNIADNFHAIQRDAWDRHQEWFKYFLLMSFTVDGTIEDLTLYYEPDDLGAHLNQLAIEARGKPGACQGPIWSALGRLFATNMVQYLGGAEVLLISAAAPLDADLDSIHATMENFDVHTPVIDYIHVETPQCAVDDWAKGLSTFANFLTTTGGTVFRVGQKNAGEAIDAFLPTRYAPQRLSYSDPLNCQNNEIFVQLDKYMSEVYIMVGGKSTVQVEDPMQQVHAIVQIWQSDYQSLWRLYKDTPGIYKVSVNSQDRACFPVVYGNGGAQVFFGYVQDYNYQDTPKPYPVFGVVNFPVFFLYDPTTGNSSETETLYMAHMDRQTIGGKFETPYDSDIDSRVGCSYNYIGKKFTCTEENDVITMSASGVDAYNQPFTRQSTAYCKKDGYLTTTTKAAKTTSTTVTTAPTTTALTPATLNFDVLFIIDETEEEGFIHDVAEPFIEKTMSLYTTTQRFARVGLITMPQKQNKSMPVAFLSSVDSFDALDQNLMSLEDFNIPGGDEYLVQALQFANDPLKYRMEEHGYRYGISNHLIVILTAKNKFADKKDAIINEIQKISAAQSYGVIGVGYGGQADWSDLTDIAGGDCVSIAADSASLLDKTTSFIQEKIWNAAFNGGTYCTPSNIAMSARNH

>Ppa|PPA15131

MKSSTSSSTRVSNFLLATLVSFWKVALWALRIYTCIERPNEVSWSIVSEGNVDLNCSKELSLIFTSTPPNVNEASQISPWQDFKMKNATVDENHEQIFVVPQAGIRIHAMQCLGDGNITASAGAGSGEAESRCSDLPTWIISFDNVITLSADKVTWLAVEYVWIGDNGMDVTVALSDRVAVMSSGLSDNVQNLPSAASLTEAADITMETQEGFGIKYYSALDTPENVGSRQDNFVVELTFTDIVPPILEKDPYCGCALAEDQQGPDGWDPRNIWLDVVIVLDTSEAMTQDDLDIASLVMMEIFAGPGEKYLTTNTGATFFIRVGLRTVAEEPQVRYNLNMTIDNLYHVLAQFKVTKGLDKMNFDDALNAAETMLTVSRSKLRIVKQLDKSGQLSTADRFKNSGGIIIVNNYIKEGEVHPGLFNLASPGGCFQAVPASSQFLNARKNCDQLGGGLISSIHDYHKGQFVQQLLTSASKSGFYWIGYTRTDSSTNPYTNWDEHQPSKLSHTKCAYADASSNLWGAGNCNIAYPTSCASSPQVSTQQVKSFLENYIDEMKLKREEDRKLKREEETVVNEKVYTSAMEICIFVALLALFTGGTNGYVYTCEEVKYKMINSDLEVSTDFACLVAAGNWSTEDSYRVDPLSSVYVSLGNATTSFMDIYNSTCIERPEKYSPWKIVADPAFDLNCTSAQFALIFTSSSTNINHDWINIDFRLKPAVVAELDEQVFAVPFWGQNLVMGQCLAPIGFTESDSNRLAIIASGLSDNPQNNPRFNNRADNRISIDLNFTSDGQITMDTDRYYSVYYKSAPVWPQEIRTRNDNFVFELAFTVIKPTLPPPPPPTPAPGPEDPYCSCALNRKSSGAVGWDSQEIWLDIVFVLDTSEAMSENDLYRAYILIPHILGNKGGNIGAYLTTNIESYFYSRVGLIAMSDKAEVAYNLNMTYTSSMKMYHVTKGVPKINIEDAFNAAENMLKQGHIAHRASARKIIYFVTDSDLIGSLPAADQFKANDGIIIVNNFIKAGEVRPGLFALASPGYYLTDAPFQQKEWLSLQLLCKANCYCRKEAGFTRYANYNKDPTKPASVLVDSKPEYPYFWIGYTRTDGHWNWEDQSTDTYTRWNRAFGEPSMPSLATTPALSFANSCPVKPAIRSAEAIEI

>Ppa|PPA15390

MVPMNLIFLCLLVPSVYSTFPCAQYGSDLYGEINCLRRLLNQCTSEKQNRETRTVPTVPPPTTTSTTTTTTTPRPTITTSTSTTTSTITPVPATDKWEDAVIESSDDWPTPNSALIIDALKYELSREIEEKIREANQKFDAKLASYQVQMEVEMKKMNKEMRMIKLKVHRRANYEYILSEERESWYTASEVCIQWGGHLVSIKDSKENAFVAAFLPAGESAWIGLNDIQREEVFVNHNGEKTTYRKWEEGQPDNLYHNENCVEMMGGGEGRWRDSLCLLTKRFVCRRA

>Ppa|PPA16060

MRSLLSFIVVFLLTLAIVHSFDYNYYGSRAFGEMDKRNPGMAMRNVGPQENLAGFLNQFKPSFGKRSRPYSFYPDIRRKKEEVKICPIPSNLNPHLILSSMAFVVTLSGFASATFRGPIADIYSTWDLSKETIYVKDFDHLAPLYVEKEVHEEEHSRKKRNAEEAARFIASANPITKTCDRPGYTGQYCEFPICQQTNLMIDPTQFQTGDGYLVDVADLGNCTRSQEIIVDETMYDIRILIQSADNVNPTFTVTDSNGYYGQPDGELKESDRYEAHFNELKPGFYRVTGKADSLNSRCLLQTTSQTIMTISGGFSTDERDRNDFPNPNAQVHQFNSIMVHLNGARSPAELKTISVIGTNNYVFRPRILDKRYGCGYEYYFDSMFCMVKGSYAMIVEGVDFNGNPFRRAATFQCADGPPQTTQPPTSTVTTPMPTACDNGGVFLFNGLQSSCVCQDHWAGGHCEQPLCINGGTLIEGKCFCPTGFEGVHCEDVRCEPNSDHGFGVDRPTLVLVVRVRQQMNDVMAQVLRAVDEISDNLQFEPNYFTRFQVVYFNDYTNFKSQSYKNIYEFNADFFKATISDHTDGGCTDAVIGAAATALTNLALTSNSFIYVITDALADDSPAMTDALLQWNSYFRATINFIYVEPTTDSGCQSDLSDPGFRKFDDIANTFSGLAMHVSDRTKVYDVFYNHLNSIVYKSQLMLTVDREECGNGLVKTVMIENKNENLILMSKGKGFFPVITNPMGENLGEDTLLTVANYQTPGPNQAEAFWSITTDIDQDGQLYQPLAGIDNHPVFHVDNLQDQDDWDHAFSFLNIYSWRNNQEMEIYASNGLYRDGCSYKFYFPSFRCRANEKLHYEFFLRNDYGFYIQRAGVMDCYLYIPTPVPPTDCQNGGVMFNETCLCLAHFTGDKCQTQICENGGTPGVNYQCICPNGWGGAFCQFAVCSEPGLPPTFGYHVDMAFLVEVTKSGVNQIKELIDQLPGLIRDITSQHGDWIDRLVLIGYDSKDVIRMVDAPISSPGKVFDALTAWGNSNPTDDNCVVRVWEAVFQLMRNRMDGPNRRNLPRRSIVNIFQSSLPDNQGDAIQALSTSEELLETNALTNVFQWLDTTSDSHWRCNGKQDDFQYIEQLARRGDGKMYTIENSDIKNILRMIPTLFSSSIVYKYHNEECHSSTNLVYFPIDAYTQTISAIVAGYKSTVQLFKYTGDQFTDDGRIPIHKNDMEQIVEFRNHNSAGQKIWLGLSWSATGGWVFQHDDGTTLAVPANMKKNWDGGVEPNGANGKTCAYFNPTAKNGYWFAEDCTKKYLTVCQKHMFDSSNEPSSIVDDDLAPGKYYLKVQTETPTNGWGGCDVEVRVQSDLNVEFGFVDGLRKDSPHPVANIDSNSNRVVSSISIGQAKTDLSVLEHVLLRDDSNQNVLLEAATYSYRFGCAYEYYSQELNCDLTQGKDFNVIHIGEDDTGNTFQRYSTSLCYKWNVCSNGGVYSNGACLCTDYWTGDNCRTPICQNNGVLNKDGKSCTCQPGYGGNACEFVQCDADSQTKFSNDGKVLALILEKSENTADSIRDIANNFKKIMDGINAKADKWINTFILHTFTSTGSVDDTVVLRDIDDVIAHLNQYASDADKLIGSCQQPLWDAINGLFTAFTPFLKGSEVLIITASAPLDADLSSVQSTMELFDEGAPIVDFIHIESVCQTEDWMRGLESFYWFFQTLGGTMFRVQPGIVADGLIGFLPTRYAAQSLTIQDAGACQQNTMYIQVDTRMREVYVLVGGSSGSVSVISPLGEQVIPTTVYNADVQKLWKVDIPYPGVYAVTISSNSKACFPTVYGSGGAQVFVGFVQDYSTSDKPLPYAVYGKVNFPVFHIMDRPDAPGVETLYMANMYVQSVWGKQGKMYDTDLDSRTGCSFEYIGKGFTCSNKDEVITVISSGVDDYNQPFSRESVAWCKAGTVPPPSSTTSKPTTTGPTVTSTLPPIPSSSTSTVTPPVDNPTVITSVLPSLSSTSPVTVTSTVTTTTTRKTTTKYPTTTHTNPPTSTTTLPPLSIPPTTTTTLPPTTTSASSLSALWGLTIVFLLTLYSI

>Ppa|PPA18549

MTDKIVASYRENARQIIYYLTNSAPGANMNGVDDFKTGGGIIIVNDYILEGEVADPGLQKLASDNFFFTDLSENYINSLGVFCEANCFCSPDLHPFNDDDNSPRTQANRGCFHPVNNGIPQQKARETCQKEGAALVSIHDALKEFFVNGVVSIFGPKKKFWLGYQNDGTQWIWDDKSTDPYTDWDNKQPNTNGGKNMCAYATSTFYMHITFHYCSPLQCTQLDQPTTQFVMKIHSTRCSQVYKDYSSPRYC

>Ppa|PPA18563

MKTHPYFSVVLFFCIIQGASSIVYSCEEIGFRLINAGYLTGTKYACIFLEEGLSPSTSYLNEIFIYNQGDSTNHSLSSIASSPSHCVEGRGNWQILSDHRDDLKCDLEITLLMTSDSDTEYVLATSSEVQYRTGNGRVTFVSPHSGMKISVNNIAADLTVYTGAGISNEMLYAYKTWTASEIPHYFASFDNVLTFDTKAKDAIYYVTADYRNLSTLDVGEKAAILTSGKSDNPMDKHPDENYLRYNLLEAATANVHGNLYLDPTYHGTINFTVKGDYMNEERSFTDASIDWKFYASYFEVKYLTSINPEDVWLNQDNFLIEIEMSELPTDITPIPGIRTTEAPDVKSIDNYCNCAITDGWFDNDWDPANIWVDVIIILDTSKSMGASLEEAKSVISSFVGIMSTDVTVEFYSRIGVIAVSDTVEVIYNLNMTSSDDLDNIQQHKIDKIDVGAAFQAALKMFADGTKMTSYRENARQIIYYLTNSAPGANMNGVDDFKTGGGIIIVNDYILEGEVADPGLQKLASDNFFFTDLSENYINSLGVFCEANCFCSPDLHPFNDEDNSPRTQANRGCFHPVNNGIPQQKARETCQKEGAALVSIHDAQKEFFVNGVVSIFGPKKKFWLGYQNDGTQWIWDDKSTDPYTDWDNKQPNTNGGKNMCAYAQQGTGFNTPWTAANCGMGGVVYVCESAPCAAGNKKC

>Ppa|PPA18776

MSGLRAEPSLSPVIRVLRLILLANYASSLNLPCSHSAVYHQMKFNEAVDIKTPGFDNNQTYPANMSCEYMFQGSNTSAKIQVDFISADFEQPIFSGCADYIMIKDGSSTTSKELIKFCGSDPLKSIVSTNDMIHISIVSDQTVQMRGAHMVVREFFEGSCGDDWDTKSSFPICFKFIKKRKTWLEAQRSCFAEMSNLMTTTNQEEYDFIIDTYGDNSSFNYPWFGIFDAATEGTYQSIQYKEVLWPHPQPVVANNSPIRDCVILDFTIKEGMTHVAEDCLARHPFICKKNKDGSTSAVMGPRQLIRRSQIAEKVDLTIWILIIIALILLIVLLCLFCKDCIKRKCCPNRVEPENRLMRGLEQDYRNPVPVAPPRNLQPSTNRARFVDDVDARAKESTIEAQRRNNETTANNNSTRPTEITIGSVSLPANTSPQPSFNHPLPPLIDRTPPHNQTVHVMPVDAVVHRSEEPEEVPVLLPVVHEIDEHRPLSPQELPLNVPSLRTDHQRGETIDEREPGPSNGNGYRAETAFRSGPTIKESTFISTREESFLRSKKNEGLFEKPKTKVLDNVSAISLDEFWNATKK

>Ppa|PPA18937

MLPFAMNHAFFFLFLSFYGSNAFTFTCQQVKKLIQDGTRFPTTDACISLDQFVPTSSGFTTEVFFDDGSGKGKWSFAQTASIGCVSQYSSMGAWRIVTDRPDKGEGIDCTYEFTVFFSSIQTNLVTAKNSGLHHQSYKAYNITVVAPRGGILIDFSCKDTQEPLNFYTGLGNGAAEDMYYYGSSLCDDRLSVFAWDTAITINVPNDGYIMITYTGTGAYLSLGSVSYDFILLGSGKSNAIVQHGEEDKFVWLSPSMNDDLKFHIDGFAEFDPRNGNSLVLSAQCKENNCTGSINPVPSDEIHLQLEADFLAIDYFTHVPDDQWKNSDMFFIRIASAPLNSTEPPPTIPPPKPNTDYSCGCELTNGKAEFASSIWMDIIFIVDVSKAMGNDGIAKASSMIDSIVSSLTIGNVSPSSIQFSRVGVVAVSDKAEVIYGLSNAVQSPLKLTPSTSAEADIGIGIESSLNLFLRSHRSSTRQFIYVIAASDSSNTDRNQPRSNQFYDATFDVQKFKYNGGIIAVTEADPAQTVTWFNNLASPGYFNPKFNDNVNLDLQLLCDANCMCPSGLSAFTREESERETALGGCYHSPSSTDPYALAKEACQAEGGILTTIHDQDKQLFLLQLISLYGAKVKFWIGNEYNGKEWEWIDDSNSTFSNWASGQPVSSLGRCSISQQTVGFNSGWFSEDCSDDHLYICERPPCSVDRFSVNGFVYSCNEVKQSILKKTTISASNACVALGDYSVLAGFVQDIFVQDGTGKNQWNLIEVHEAGGCVNSTVDSWRIVTNRTDGGEGIGCDEEFTLLFSSIQSTIVTARDNGRAHYSNTAGNITVVVSRGGILLEFTCNNTLQKLDFYTGLGNGEEEEMFYLGSAQCDDQLSLWALDTAITVSIPDDGYIRMINTGTGGYLSMGYYEFDMIVMGSGKSNNLIQHGDNDKFLWLDADDRVSFAIDGFYEMDQKVGNNIVLTAATDCERDPCTTKSVPLNSSSVHMTMVADLLSFDYYTKVGNADYWRDDDAFFFRIISSPLTTESPTTIPPVKGDTDYSCGCDLTNGIASFSSDLWLDVIFLVDVSKSMDSVNLNKIIADLSSPFPNSINFNPSNSPEANIGNGIDTSIDMFSRASNRTSRQLIYIITASDSSSTSFKIKFTQASALQSSPGDSFKNSGGIIAVTDVSPSKVPSLVDLSSPGYYNLGFRNNINLDLQMICDANCFCPQYQTGFSDDSDERETPSRGCYISQPITATFSLAMDYCSSSGSLLSTVHDDQKEYFLIGVISQFGAKTPFWIGYENNGKDWEWIDNSNSTYTKWASGQPNIGSCAYEIQTTGFNSAWYSDNCSNEHNFICEKSPCSVSNFCN

>Ppa|PPA21045

MGVPKFYRWLSERYPCLSEVITDAQIPEFDNLYLDMNGIIHNCSHPNDDDIHFRISEEQIFNDIFKYIENLFNIIKPQKVFFMAVDGVAPRAKMNQQRARRFMSAKNADALIEKAKRAGEEIPTEKRFDSNCITPGTQFMVELQRRLDEWVQKKVNTDGRWRGMRVYLSGHDCPGEGEHKIMDFIRSERAKEGYDPNTRHCMYGLDADLIMLGMCSHEPHFSLLREEVKFTRPSKPGGGKKKAPPPKKTESDTICFHLLHLSILREYLSWEFIKVKDSIKFDYDMERIIDDWVLMGFLIGNDFIPHLPNVHIHDDALPLLYKTYMDVLPTLDGYINEDGHLNLHRFQEFLTAFSRNDRNSFLQVMEDEEYLASKMGATGIDDDVEGTLVQFHDDDMSTASDEEGANSGDTEAAFQSSDDDDSSPALPSSRGGPSQGVLADGGIGGATDLLAQLKDDPVEDEFADQLAALQFRDMDDAEFENNVDTAKCWSRSINNDFKRHKKRYYNEKLKMGNISKGQLREQAEGYVRAIQWNLHYYYHGCCSWNWYFRHHYAPYISDVLDFTEMSMGFEMSTPFLPFEQLLAVLPAASSECLPRPLREPTLAAEDRARNVHGPHLLFTTKEAPENGNWCSRVEIEKDTFRIPITKVVHGLLPNVKLDVFFPGFPTMKHLPHKGELKEAHVKVFNMASRKLSMVLSILDRPDLNRDVESISAQLLGEEVCIDWPVLKVGLVEEIWTETHAFRRCYDETTREEVRGKVVAEKLDEDQQSQFALWRRNTKDRLMERYAIEPGTVKAMARVRRLLGTSMVVEGDKVVPRRQWTPVDIAVPVPLQLVCIGVLVNENISRVPLSIKEAFPVETKVFVMDPSWAGYGYPAIVKEIIDEGTTNCRLTVQYALPQQEMDLRPIRSEPDKFSLQWCDGYTSQRQTGLDRGLIARLTGTVFIMDCTQDELTADASFRPPKLNVGLNLKLSKRNEETPDYAKRLPEGYWIYSILTVRALAEYKNKFPEIFKYLEKNNSMDDNYYSGDIWPKEEQRKERMDELRQWLENQPSFSEQKQTGGEEYADRNAVAYIESVLAATNKDCVFRKSIVKPSLIYRPSLTSSSKKVRPDPDANFLLFDRVTFAQDNQSVPFGLTGTVIGLHGTDHVDVLFDREFPTGTKMRSTSASCARVLIAALVNTTFAKERKTAGIMKPPVPKKKEQYGSAPQKNAWEDRSKKGGDEKRGGGGGKEREKKDRKDSSGSKGKEKESPPATVLKLLKNTNAAKTVSGVAVSAPPLPNDNQKRNKAEKTAEAQQLLDMLIGGGPAPTNKKGGKKEPKKEMAKIEKKRSTDEKDEEERNKRGAKAGAAILASLMTATSSKTSSRKGDTESVQSTPSTSREERPPNRKERRMEKKEKQKQLAAQEGVAAVWSPRPKTPPVSEEVSEDGEKMKKKKNIMENTVCMQLLAMLGVNPEKEKEEEKKEERKEDKKEETAVEKENEEAIEPLSVSTPPVQQVQQQSQQARPSPRGGRAANQFWGPTRGGNSQRGGGPPHNQQHHQQQHPHVNHHMMGYGGRPGLPPMNGRGRGGGGGGGQRGYGAPPMGMPSPQEVMMHVARQQVVHGGHSLGPPPPPPHIVQQIMQQQQQQRMNNERGGFNNQDQNGRGGRRGRGGGEREAMKPEEYVRDKVTDLTPASVRGFGKGRGGGRGAQRGGGRGGGARGGRTSRTGPSPSLDAARSPVVSPTPPTAAAAAAPAASAAPAATQQQPKPRKARLAVKLAAKSDLASFSSLTPQPCATGDGPWRVVTDSPSSVDCTYEITLLFSSVPTNLIVVQPHTYEYIRGPGSDLTFVSPRGGISLNWHTQGEVTGYEEITFYTGVGYGPEEDLYPIGSLFTNDMAEGRDTDIFDPVVTVKIPSNISIEIGYDTFADKASIHRNAIKTMSVNVFGYPGYSVNVLSSGLATTFQEQNTMKVVQASYGRRASVHVTASITFDPSSDHKLKLQAYCGDDICGEKIVQQSTTVDWLLNAEKFRVNYITGLTASQIGKNVDNVFISVVSSTHNQVDGSKKCIDFLEGVRCDDDYLQLGDHCYRSRRTNTFINQLASGAPIGVFIGMKQDQGVFKWTDGSAVDYNNGHLDSFGGECVIMGSSTETWSNADCDFAFKYICEKNANE

>Ppa|PPA21410

MCFSQDTQMTVMFSGSFDARYDNYVDVAGNSSNGMQWARTFRNETSSMSTVATNFTALYVSTPVDAPDLWDSQDNFVVVMDLTDPYCGCELDKKFGMPIDWDPNGIWLDMVIILDCSEAMGKNSLVEASNLIQSFFGEGDYDVLITDPKAQLYNRIGLIAMSDVAEVFNQISVNIFAKIIWNVKTIYNLNMTKGDKVNVSIKTGVKQINVLDAFAAAQQMLQDGQTPGRSNARHVIYYMTDSDPKVDLTSLDSFKEEGVIIVNDFIDSHVIERPGLKDLASDGYYYTDIQDNYMSSIQLFCKANCFCSAEADHVPYAGHNKDEAGEAAGGCFHVAPAGVPYNSMIQICLELGGGQMVSIHDDNKAIFVNNLYKAAKLKPDYYWIGYAKSDDLWSWTDKLFYAKHTMLSVYESVHARIRLTTVLTSTRPRRIGEESVSSLGVLSLVPLTVEKARGMKRLRPDSRAGKIIDHVEKKMHRILFRGAANCNVGYPSVCEFKPCARKRSTRHSCLSACVVVISSMGLFGSVFLFFLFIGGTQAFVFSCEEVLNKLINSNLQEPTQYVCLVTQEGFTNEEALKDIYVQNDKVSTSFYTMWKEMPCVARPGSAPWRVVADLPLSLDCTQEFSLIFTSVPGDIFPVYDHENWLKAGVNALYVAPKTGIVLHKDTCEGDGQLNVYTGAGKGVGEFRFHLKSWSCADFPDWIISFENVITILPDAGVSYSVYYRTHDHTTGIDVSPSDHVAVMSSGYSDNLQNLFQERNVVVFNEKEDVEVTVNRTASFDDRYQGSVIVDYTNAGEGLEWFETIHNETGGSTTTVAQRVQVEYHTIPFAPQDIWDSYDHFVVELFFTALEKPTTAAPTIDADPYCGCALDKKFGMPDGWESTKIWLDVVIILDTSEAMGAVALTDASSLIESFIGTDDGDVLVTDKTATFYTRVGLIAMSDKPQVLYNLNMTKADKVVGKVQINSGVSYLDVVEAFNAAVQMLNDGMQEDRAYTRQVIYYMTDTDPKFDSRVLDSFKASRGKIIVNNFLEPNEVERPGLKELASPGYYYSNIQYNYMSTVQLFCKANCFCRPDIDRFPYPGHNTDPALLASGGCYRAVPAGVPFSKMRGNCEGGLIASIHDADKANFVAQQFTIAAPNSDYFWIGYSRGDAGWAWEDKARQLFNKPVHELGQSERRAQHSFGRQMGAGNCNVGFPSVCEYAPCEVLLSFVIICFYPLICLAFLRAVSRSFQVRENVEVMTFMCKIYLPAAVVSTIAFGGFGWKLYVPTEWRLSRNIAYACFDLGLELCFVVFFSCAILGNERIHREFSFASFAYLNLGSTTLTATLS

>Ppa|PPA21927

MPRRQPVKKERQSRPTSCVVCGSDAAGYNYDAPSCASCKAFFRRVVIKKQVFPACDRNALCDIDVRRCRACRYERCIKGGMNPLIVACPDNNESKTDSSDSSSVNGSEPSTSSAHAEYSPHDQSFQEDPEKIETIIEICRIKKSSPRSEPSPQSTEFDPCDPDNEAYYMNEHELSLVENPIRIEKRLDQMMHHLLLLEEACQKLRISSYVIKLVPGLSIDDFYLTQSKMGSNFEPMDSSYEPVSIVLMPVQVILDKRQVVDHSKVEQTSRRIWPIQDVAYSIEYIKALPFYNMLDDVSKKILIGSALICSNFTSAFFSYSKGADRTTYPDGSCMTWDRQVEEQSPEVARFHTVLIVAMRQVELDDREYSLLKLIIVCNPNAPKHFFRMYLRDEECGPAYFAKILSLIEVATKLTSWQKSTHILGLAMGLYKNITPDWNMRVCLILLLAFAIGEERNIVNISSHGREQTGDDNRCRSGFFYREGWCTALSPKFLSFSGAVDYCAGIGAYSPSIHSKVDLDYWAGSAFDLSPRFWLDASCPEEGEPNQWRDKTPTDYMGPLGELQMCTPNYGFIMSSKGFVNETYSDMNQAVCVYPVDPMPEPPLINVDDQYCQCNKSAIELDIVFAADTSEGMSGTDVSDITSTIQSMLCSFALGIGAHQSNVAVLSFGNEVKLVKNFGDINSNDDISKINVPVLGGENPNFEEVIDQASSMLASTGRKLTRKAIVILSKSSSSIDYTNGISRAQQFQNTGGVVLTLDLAINSNALNGLSSRGYNIKLSSNVVADSLEAFCDANCFCQDGLVPYLVPNEKDRDLPMGCYKVGQKVAIFDDAQKECRKLKGFVITIHDDAKNFFLISLFPPKAPHWIGLKEYGSEFVWTDGSNDGYTWWAPGNPVKGLACVYAQQTSGFNTGWFSAPCKDYTYSLNYACQLRPFDAGYSGIQ

>Ppa|PPA22158

MTDLSLAENNHFSMPTSAVLQSLLLLCLAVGINGYVYSCEEVRYKLINSNIAQNTTFACVFTQEGEFTLVLTSDVPTFQTPTKQPRQRAIRGRETFIQPHTYMWFHTESCLGDGDITFSTGAGVGEQEERYVLRTFQCWNMPDWIFSFDNVNTIYVDDGIVLSVEYSSEPGGSKNPTKVAKGDNIVVLTSGRADDVQNLKGRTDNRAYIQIGNKDNADVSMDMMINLDKDNSGSVAVQKAYAGTTYNYYNGTRTENFNTGYFETTTPSPTSAAATTKAPKPAGDDPYCKCGVDKFGFPAGWKYNDIWLDVVVILDTSEAMDASSLADATGLVESFIGDANDNVDFLITDTTAPFYSRVGVIAMADTAQVLYNLNMTKTDKVQAAIKKGVAQINVVDAFNAAQKMLSDNPSKVGARQVIYYLTDTDPTNNLSPLYQFKTTGVVIVNNFLQAGELEKDGLKELASYGYYYSDTNYMLTLQSFCKANCFCNPGKDALAGTDPATPSSGGCYHPSSAAVPFSKAKSTCAADGGIIAVIHDDDKGRFVQQQMAKTGTKSDYYWIGYEKSGDNIWRWQDQISIFSSRTEFDGPVHELGRGSQFQFVYGDARWASRTCASSRPAPWATRAAEPFLFSFMKSLLESEQPWSMPSYKTMVVVAVVIWMRRELYRWAVMASRSLYHLWDLKGPTNYPIVGSVYDFRWNVFALGMFKAWLGPVPIVILLRARHVKMILESNSLITKPWFYDIISEWIGRGLLTSTSEKWYSRRKIITPTFHFNILKGYREVFVAQGRVMVDELEASADTGREIDVFPIVKKCALDIIAETAMGTQLNTQTGGNADYCDAVVRMSNRMFEYMLFPLLWFKPIWLASGRGIEFYRLVKKSQEFTLQVIRERKKLMQDEGLLGGDTAVEDSKTLSKKSVFIDMLLLQQAANALTDEDIREEVDTFMFEGHDTTASAMGFTIWFLGQYPACQKLVHEEIDAVFGDDVARDPTEEDLRKLPYLERCIKESLRLMPSVPFIARVCSHDVEIEGVTLPKDLAVIVAPWIMQRDPEHWERPDEFFPDHFLPEKVAARDPYAYVPFSAGPRNCVGQKFAIAEEKTVLSWFFRRYSAESVEPFPGSPIVPELIMKPYNGFNIHRSLSSDPSITYGISKDLQTTPFWARFPVKNWTLTRNRQSVGRALPGCILAALTTHQGFRLIQHIIISPDGPGDTSDKWFSRRKIITPTFHFAVLKGYQEVFVTQGKIMVDQLESTADTGREVDVFRYIKNSTVMGTKLNSQTGESAEYSDAVVRISVNAFDYSRFPHLWLKPIWYGSGRGFEFDRLAKLTREFTLKSMKEEGLLDVDSSETLVASKKSVFIDMLLLQQAANNFTDEDIREEVDTFVFEGHDTTASAMGYTIWFLGQYPEYQCLVQEMDAVFGDDSVRDPTENDLRKLSYLERCIKESPRMVPLVPQFGRVLSHDLEIEGVTLPKGLTVFISPMATQRDVCKLGRVCKLAH

>Ppa|PPA22164

MRFIAALLQLLLLTCILERAGAQAISCTDVGAKLINSHIKPNGKVFCVIPQEGFSNFEQLKNIHVQSLFGDKSSFFDLATNNTCVEQQLALEWTITADKEFSLDCSQHFDVIIGLIQVEFVKPIKQIQEKTIAESEYRQFTVPQTGAWIRIKEANCKDASNKVTLIMGSGKDATGYNDYNKPHQWKCASVPDWIVTFDNYFTLSSDTNLLIEYSSDIESELQVSPGDKIFMFTNGINFGGKSSRALFEIGHEKRHNISVSVEMPEAPDNNPSIKLRKGQNDLLSYNAGEHKSSITGKKFEVHFNASNIPSSQLQKIEHCIRININIDNEAAPDLPDSTTTESPEETSTPEEDDEETTDSDVDGATTTGPDSETTTPEEDDEETTDSDVDGATTTGPDSETTTPEEDDEETTDSDVDGATTTGPDSETTTPEEDDEETTDSDVDGATTTGPDSETTTPEEDDEETTDSDVDGATTTGPDSETTTPEEDDEETTDSDVDGATTTGPDSETTTPEEDDEETTDSDVDGATTTGPDSETTTPEEDDEETTDSDVDGATTTGPDSETTTPEEDDEETTDSDVDGATTTGPDSETTTPEEDDEETTDSDVDGATTTGLDSETTTPEEDDEETTDSDVDGATTTGPDSETTTPEEDDEETTDSDVDGATTTGLDSETTTPEEDDEENTDSDVDGATTTGLDSETTTPEEDDEETTDSDVDGATTTGPDSETTTPEEDDEETTDSDVDGATTTGPDSETTTPEEDDEETTDSDVDGATTTGLDSETTTPEEDDEETTDSDVDGATTTGLDSETTTPEEDDEENTDSDVDGATTTGLDSETTTPEEDDEETTDSDVDGATTTGPDSETTTPEEDDEETTDSDVDGATTTGLDSETTTPEEDDEETTDSDVDGATTTNPDSETTTPEEDDEETTDSDVDGATTTGPDSETTTPEEDDEETTDSDVDGATTTGLDSETTTPEEDDEETTDSDVDGATTTGPDSETTTPEEDDEETTDSDVDGATTTGLDSETTTPEEDDEETTDSDVDGATTTNPDSETTTPEEDDEETTDSDVDGATTTGPDSETTTPEEDDEETTDSDVDGATTTGPDSETTTPEEDDEETTDSDVDGATTTGPDSETTTPEEDDEETTDSDVDGATTTGPDSETTTPEEDDEETTDSDVDGATTTGPDSETTTPEEDDEETTDSDVDGATTTGPDSETTTPEEDDEETTDSDVDGATTTGPDSETTTPEEDDEETTDSDVDGATTTGPDSETTTPEEDDEETTDSDVDGATTTGPDSETTTPEEDDEETTDSDVDGATTTGPDSETTTPEEDDEETTDSDVDGATTTGLDSETTTPEEDDEENTDSDVDGATTTGPDSETTTPEEDDEETTDSDVDGATTTGLDSETTTPEEDDEENTDSDVDGATTTGPDSETTTPEEDDEETTDSDVDGATTTGLDSETTTPEEDDEETTDSDVDGATTTGPDSETTTPEEDDEETTDSDVDGATTTGLDSETTTPEEDDEENTDSDVDGATTTGLDSETTTPEEDDEETTDSDVDGATTTGPDSETTTPEEDDEETTDSDVDGATTTGLDSETTTPEEDDEENTDSDVDGATTTGPDSETTTPEEDDEETTDSDVDGATTTGLDSETTTQEEDDEENTDSDVDGATTTGPDSETTTPEEDDEETTDSDVDGATTTGLDSETTTPEEDDEENTDSDVDGATTTGPDSETTTPEEDDEETTSPTDLTTRFLSTLLTTAFTTTFPLETTTTALNPWNPSSKDPYCNCAVDKFGFPIRWKHSDIWLDIVVILDTSEAMGRAALDDAAALIESFISDGVNDFLVTDESADFHTRVGVISMADTAEVLFDLNMTKTDRVRGRAAIKEGVLKINVIDAFEAALLMFNHGLKARPDREQTRQVIYYMTNSDPKNNLNPLNQFKASKGVIIVNNFLQEGAVEQPGLVHLATDGYYFSNHQYMNALQHLCKANCFCKRDRNAYAGADKAFRAAGGCYHYAPSGVPFNKAKTTCAQEGGILATIHDENKAHFLHHLTSKAPQGQFWMGYQKSAEGDWQWLDQSTDPYTNWDGLEPSEEAVAKCAYVSTANATLPWSAGNCQLGLPYICQYTPCSVGYKDC

>Ppa|PPA22169

MLLRSLLFSLLIVHSAHSFAYSCLEVADEFVGSSFDESLTYICLIAQEGYSNWQQLEKIHLTADITTVSLYDIVTSSSHCVAKGSPSARWTVQSGLFDLTCNQEFTLIFSSQKPDFIGVYHKAAQQFVARNTLVVPQTGLWIRSKGCLDGDVGSVTFYTGVGTDENEHIFEMKSWPCSSLPDLIVSFDNVVTIEVAQNVYEMECSSDIDRNIANLKPFMVSPGDRIAVLTSGRSDNLQNFKGAINAIALQLNDGQMYDVTASMDLTFDWSDTGAVWLQEYYGGPLVEFFGGKYIEYFHTNYLQVQYVPNGLSAAQIDASGDNIIVEITIGGGAPTIKPTTTAQSKTTSRAPTTKAAPTTNAQSKTTPRVTTSAPARTTALPIASTTASSSPPSESPYCNCAVDKFGFPVGWNYNDIWLDVIFILDTSEAMGEDSLGDATSLIESFISDGVNDFLITDPTAPYYTRVGVISMADTATVLFDLNMTKSDSLSGKAAINKGVSEIDVVDAFDVALRMFSDGNSADRVNTRKVIYYMTDSNPGANLNPINQFKTSQGIIIVDNFLEEGEIELPRLKELASVGYYFANSNYMEGLQAFCKANCFCKPNRDVYQGSDPAIVASGGCYHPSPAGVPFNKAKTNCMNDNGIIATVHDDDKGRYLQQLMAKSSSKSDYFWIGYEKSDAGVWQWEDQSMSSFTNWDVREPSTAAVSKCAYVDTTNAALPWGAGNCQIGFPYVCQYTPCSAGNKNCYRHDAARRKNQIKMLLQSLLLLWFSVQGVKSLAYSCLEVQDEFVGASYHKSLTYMCVVPQEDFSNWKQLQGIRVSAGTQTASLSDIANTPTRCLEKTSPEAEWTFQSTPFDLDCSQEFTLIFSKIAIDILTPVAAHAELGVGRATLVVPQTGMWIRTKSCVAQTSGNVTFYTGVGSDDNDHKFEMKSWPCTSVPNLIVSFDQVITIEADWSVTYTMEHSSYIDDFFWVYPFDEIAVLTSGRSDNLQNLQCTENLAKFWLGDNQKYEVVANLDLTFDWADDETGTVILTDYYTGQEVPFGEGKYIEYFSSDFFTIRYAPQPLLPAHIDACADNVVIQFSIGGRVQTGSTKPAPQTTTVQRKTTTRATTAALPTTTVQAKTTARATTAAPTKTTTRAAISSTTSSTKPPSEGPYCNCAVDKFGFPVGWNYNDIWLDVIFILDTSEAMGEDSLGDATSLIESFISDGVNDFLITDPTAPYYTRVGVISMADTATVLFDLNMTKSDSLSGKAAINKGVSEIDVVDAFDVALRMFSDGNSADRVNTRKVIYYMTDSNPGANLNPINQFKTSQGIIIVDNFLEEGEIELPRLKELASVGYYFANSNYMEGLQAFCKANCFCKPNRDVYQGSDPAIVASGGCYHPSPAGVPFNKAKTNCMNDNGIIATVHDDDKGRYLQQLMAKSSSKSDYFWIGYEKSDAGVWQWEDQSMSSFTNWDVREPSTAAVSKCAYVDTTNAALPWGAGNCQIGFPYVCQYTPCSAGNKNCYRHDAARRKNQIKMLLQSLLLLWFSVQGVKSLAYSCLEVQDEFVGASYHKSLTYMCVVPQEDFSNWKQLQGIRVSAGTQTASLSDIANTPTRCLEKTSPEAEWTFQSTPFDLDCSQEFTLIFSKIAIDILTPVAAHAELGVGRATLVVPQTGMWIRTKSCVAQTSGNVTFYTGVGSDDNDHKFEMKSWPCTSVPNLIVSFDQVITIEADWSVTYTMEHSSYIDDFFWVYPFDEIAVLTSGRSDNLQNLQCTENLAKFWLGDNQKYEVVANLDLTFDWADDETGTVILTDYYTGQEVPFGEGKYIEYFSSDFFTIRYAPQPLLPAHIDACADNVVIQFSIGGRVQTGSTKPAPQTTTVQRKTTTRATTAAPTKTTTRAAISSTTSSTKPPSEGPYCNCAVDKFGFPVGWNYNDIWLDVIFILDTSEAMGEDSLGDATSLIESFISDGVNDFLITDPTAPYYTRVGVISMADTATVLFDLNMTKSDSLSGKAAINKGVSEIDVVDAFDVALRMFSDGNSADRVNTRKVIYYMTDSNPGANLNPINQFKTSQGIIIVDNFLEEGEIELPRLKELASVGYYFANSNYMEGLQAFCKANCFCKPNRDVYQGSDPAIVASGGCYHPSPAGVPFNKAKTNCMNDNGIIATVHDDDKGRYLQQLMAKSSSKSDYFWIGYEKSDAGVWQWEDQSMSSFTNWDVREPSTAAVSKCAYVDTTNAALPWGAGNCQIGFPYVCQYTPCSAGNKNCYRHDAARRKNQIKMLLQSLLLLWFSVQGVKSLAYSCLEVQDEFVGASYHKSLTYMCVVPQEDFSNWKQLQGIRVSAGTQTASLSDIANTPTRCLEKTSPEAEWTFQSTPFDLDCSQEFTLIFSKIAIDILTPVAAHAELGVGRATLVVPQTGMWIRTKSCVAQTSGNVTFYTGVGSDDNDHKFEMKSWPCTSVPNLIVSFDQVITIEADWSVTYTMEHSIFWVYPFDEIAVLTSGRSDNLQNLQCTENLAKFWLGDNQKYEVVANLDLTFDWADDETGTVILTDYYTGQEVPFGEGKYIEYFSSDFFTIRYAPQPLLPAHIDACADNVVIQFSIGGRVQTGSTKPAPQTTTVQRKTTTRATTAALPTTTVQAKTTARATTAAPTKTTTRAAISSTTSSTKPPSEGPYCNCAVDKFGFPVGWNYNDIWLDVIFILDTSEAMGEDSLGDATSLIESFISDGVNDFLITDPTSPYYTRVGVISMADTATVLFDLNMTKSDSLSGKAAINKGVSEIDVVDAFDVALRMFSDGNSADRVNTRKVIYYMTDSNPCANLNPINQFKTSQGIIIVDNFLEEGEIELPRLKELASVGYYFANSNYMKGLQAFCKANCFCKPNRDVYRGSDPAIAASGGCYHPSPAGVPFNKATTNCMNDNGIIATVHDDDKGRYLQQLMAKSSSKSDYFWIGYEKSDAGVWQWEDQSMSSFTNWDVSEPSTAAVSKCAYVDTTNAALPWGAGNCQIGFPYVCQYTPCSAGNKNFQGVKSLAYSCLEVQDEGFRPSARVFRSLFWLCRKVTIPANCFCKPNRDIYRGSDPAIATSGTNKKLLGGCYHASPAGVPFNKAKTNCMNDNGIIATVHDDDKGRFLQQCGKNRSIGVLMSLLFSPLTLTPTGMGAGNCQIGFPYVCQYTPCSVGNKNC

>Ppa|PPA22639

MLPKTFLFLFFLPFIISAPRNGLRSQDEFESLSSPPSLPTNDPPSSSLTELEAKLNERMRIIYSDLSSRMDTIEEAIHQVHNSNEKGEWLERDDKSSYRLHEERRKWKEAQSICQSEGASLVTVDSEGENGFVTQIVKTKPHIDFVWLKMKNRPKVTPIDVQFSNITDKDQHNQCTVICSDSSWALRSCDQLRPFICKRDPPLPPKSDSDEEDEKKDEVSIDPTLKQPIQPWKNNQMHSALSGAESLNAQWFISPVVNIRAFKETAEEELQFTNSRMGVSDEYEPEQETLSSFLGCSSRTTSRILKDISLEKLLEPGKRSLNDDLEALDQPSLILPETLKTHRACMDRMKRFSDHSTKSCYFQKMRYYKWETFGNKMCPSDFGTYIEEGKELEKPDPKYFNISLYPSSFIFIHDTFYIDTSRPECQDISAEIREFMANKSCFGPTKVESMTGVTVMDLTLRLGQPYLFLHSGNCEHLIQFTDLRLMTKDDEQDESKYPIMTFERGGEVRCMACKKMEAGMGAYFNNRPLELRHTPMNPYTSCLAAERKMEGKLTSGTLHMAIRVKRKKKDPSVVKAECIGLVGTQFKFDSLCDFQYLPIRGKPQSDVFDDLIPRLIPTDLPSALCWWDKPELNGEETPLFLPPFQFSRYNTPSNKILCRETVFGEKTRKKALGHGHNLRVERKALSVNVHANDDFPLAPTADALADANFRCKNEGPHRMLFELFEERPLWTRVAVAYRTKLEETLLKSLLQKYAFYINNGPWGRLWCKFGYDPRKDPNSVLYQTLMVSFRQHTKIPERQRLKVSTSERKLGDEGEGTNSVNYVNFRSAQDLLRKNQSLEFVPDSGFISVATLDQIRDLIKEDVKKTSSEMEVDESDGMEITIDEEW

>Ppa|PPA22928

MLLQASLLLAFTSAVTGASPTPFVPPSTTPVPLPRECPSGARVFTPTNACYWIVRDQMTWSEAERACVRKGGKLSSVEAEDENEFLYDFTRSANLSSPTFWLGMLNKNKHSGQYVWHDGVQPRNAGAFKNFITREAGTASEMCVSMWSDYTQADGSWHPWSCAFQGYAGVCKRKFRSLNPSDVRSTISPLEDSIQANRCCTHCGKACASNERCIPDDLDCLIKECPGSEGYGWCLPAKDKN

>Ppa|PPA23636

MMNLTPLAILLAQVILLSQAASFKINPLQRLTNVQLSCTKVDPGYECFNGMCPESDRTCVKLDKGDTCCLNKNIVQQDDPPPPPTETPEQPSNNEVPPVTDKPVDDTPVVTKKPEATGSFCTCELDESGLAKEWAEQLWMDIVIILDTSAAMGQSGVIETTSIIESFISRMNLNDFSTPKYSRISLITASQWPKARNTMIFTTSSICHLTVISLPSKPNPSSSSISQSERILLAFKAAQYLFNFYTPAELHRGQVPRVIYLITNTRPPAKIAAADQFKQDGGTIIVTDFVQEGAVPQPLLANLASPGYFFDDLTLDYTLNLKVFCGLNCFCPSSTGSTHGGKKPTLGCFQPITEPASFDRAVQTCAMVDMGLATIHSEEEEEYLIRVKGLPHWFGLRQDESNQFSWTDKTDHSFSNWAAQEPELEDNQKCAYALKGTSKKIEWFTGNCHLPLYYSCSSAPCSAERYCSEEEIFGLKKMIAHL

>Ppa|PPA25240

MGPPLDFPKELDKVADELDSLNNLVTVLVVVFILFVIIQILQTVLKVIKPKQKNVERHCKPNAVIVQGKSIIRTSKRESVSQKSSSSRHKSLPSKKINTILSLSTESECEKSMKSNKEELGSREECKTKNAIQSIPSREMKKEIPKNTKMLNCSDVMNTPLPSTAPLALPLTSSKSSKELIDNSNKEKKMEATNKIPPTQSLSSIQTAPSTDKTPPSPPTPSQAPPTQITSKKSPTVDTTQRGSVVEILTPNKNSFVTTKEEVKTTPISVPMPPSSILLLLFLPFTSSHVSLTFPSSRYPPLDFLDSLRTKGPCGVPVPRKQYLTNLIINKPYNITWRMGYPHQGGYRLTLIDGHGDTVAQLTPTTNASYVGTMDQTEMKETVVFPKECSNCSILLQRQAIEWGKAYTFHSCADVNIVSEETEDTQCNGRGRMKEGKCDCDPGFGGKECKSIVHCSSDEDCGKGGKCIEERGTLVVKTCFCPYGQFGRNCDTREFNKHGMFNEECYMSKQLNSNDTVYYRHLKSEDEVEVILDFDTTSWVGLGWRPDGLDPSCRLFPDLEGARYRRQSPSFPSESTEMESFPTTSLRDSTAPTRTSQDGPIPVMPKSNGLCKSLPPPSLLSLSLFILLFFSSVSSALHAPLHPMDCIDMIIGAVRNGRSRIQDLYSRDRSTPIEDHWYDGLDSLSAAYGIEVDGRTIIMFRRNRQEIEPTDHPLGGTRQFIVWAKGQTKGGYSHSAPSAFDKGSHSISADSFYADDVVNYHGDKNRGVFKGDLAQSIPSQDVQKKLRDPPPPSSSSSSLPPLVIHHSNSITPSSSQSQTISPSVEVIDDSIPPSQSSSFVISSLLSIVLLRLLTPLILLLLIPSLSFTQGPNGFNDANTSGGGSPTFETFPSNRSEATKYYAYNIGGKTDPHYIYSYIKGLSFLPPPHLHRLILKKIIMMELPELRETVTELVEALSQMDNALVVQVKRIEATEKAFSDLEKKVDQQSGLISQVLDLKKQIAIMNGKLEALSKIVIAEWEQVGSSRVKPHTVAKSWDDAFHHCQVYSASLVRINDEDSNRKISDLIQYEPSSIHWIGVEGREHVDRLPFHRFAKPNRVCIALEKGGSWVSRDCSEKLPFICGYEDGSTSIH

>Ppa|PPA25718

MQTLSLLAVLASVAAACEWGWEKYKGSCYRLQSRTNTWTIHNFECVYEGGTLASIKSEGENEFIRRLAHGRDVYIGGASFGYSWEWADHSSFYGYSNWADESESYPPRGKPCISMNSYGQWEAHCCANPPRAAVCVRRW

>Ppa|PPA27057

MHFYAFLLLIALVGVSLSKITDYHQRSCDPGWTQVGSKCYKLIGSQLASISSAAENEAIRSIAGGREIYTGGLSYALGPWSWADHTPFGVYRNWRNGYEPSPSPYKPCIKMNPSGQWFDNCCRVPLFPVSARGVCSIF

>Ppa|PPA27097

MADNHCVNMENETRPWTIVADNPSLSCDQQFTLILKSDETKLVIPRLNAINKFFDGSGVFVVPQVGMLLERMSMSGEGIISFYSGAGTGEEEERYLLQSWKCSMLPNRIFSFESVITIVADDSCSYMLQYISNFDRNVNIFRNDRVAILSSGRSNNLQNRNGTKIIAYIEIGEWEESNVTVNMNLNFDSENTGSVGLMEQFAGPIDPYFNGTHKVEFVTDYFEIRWNPKELEPFEISNNQDIINIDIQVSHSNDEFECPDGYELISVDDERWCFTIFGPIPPSRGFSFLEAAAQCYIAGDSMPIVTSQEMNLRLAKFIYDFEVIEEAWIGLSCDPENGTWLWQDGQPLDYNALEGGTDKCSIAENVAIDKKSWRVHDHSDLMGKVVCSQRAHAAASSTTPELTTLHEFIGSTETTLAAATDQTTSVTSFAADTSTANTLTSTSTFSTITIISTLTSSNPPTTSTVDNSNGPPAWEIVLIVIGILLIIGCGIVLFLIVRRRRGLKDQILEEKLEIRKISQQVHMHYGIASDSEDEWEIDRRFVGIDYMNKLGEGAFGCVFLENADSQVERDFMSEIELMKRIGYHERLVNMLACVTSSRPILLICEYCANGDLLGFLRRRRKFMLERPEETESEEVITVKKQLMFAIQIAYGLEFLSSRGFIHRDVAARNVMVDQNEGCKIGDFGLGRSVGRENEHYHAQGGKLPLKWMSPEALDKYYFSTAADVWSYGVLLYEIVTLGGVPYAGWPAVELLSRLKRGERMDRPDNCSEMLFEIMCECWSEDPSDRPTFERLRKELGDLLENVHRDDYYLKLNAHAHYYVMESQASE

>Ppa|PPA27600

VYRIPPARMSRRRAPSLRLVLHATVCLLLLTTTVAPASGDDNSILSIDDVKLACAEGWTRQGAKCLRAIAREASWRRAEEFCKSRGATLAHIESPSENEALGAEVLRDPEVAGRANFWIGLSSDNSDRGVYHWSDGRAVSQYVGFWAIGQPEETDGDCVSVSLASSPLWSRSPCAQLLPFICQQPACVEGSNFCRNGKCSSISDICNGRDECGDGSDEENCPGTPCRAHLTAITGTIVSPGHPNHYGPAESCRWIVEVPINMRVELTFDNFDTEPSADVVTVSDGLPSSSSSNSSLLLATLSGNKQQERKFTSTGNVLTVQFRSDRSVHAHGFSASWKAVPFACGGSLTAQSFGQVLSSPAHPSLAPRGIECAWTISAPAGQLLTLSLEDLDVGSSAVDIYDGETASAALLVRFGQGNETSLEQAVSSGLEHLVSTTSRVYVVFRASRAVDVGRGFSLSYKRGCDIVVRRPFGALLSPGASGRLPYPAGADCSYTIELPDASPDHALSLAVEHFDLAGNDYLQIFEGSSKGSPLHDGEGFTQSARPPRQMTCRQSRAQLLFRSSGVRHSKHTGFNVTFSTNCPSLIKPPSVSLSTRATTHGTKVTVSCPKGFEFVTGRGVHFSTTCERAGRWSEPVVPECQPVYCSPVPQIANGFAVAATNTTFGGVARYACYEGFSFASGKKSEEIHCGDEGKWGELPVCRAASCPPLVPFMHGDRSLAFGDGSGYGSVYRYECAPGFRRMGAAALLCQNDGKWSVAQPTCARLECPSLPRISNGRLLVQKPFLFDQSAQLICNDGFEPDGVAEVHCLASQTLSHVAKCVDIDECARGDAKCDSASTRCVNRPGGYSCECLKGYQPELGCSIPSSLSTARISVSSKAPGQQLSSASSWCASPDDDDRLMRLHFSTPMVVEKLHFERENGGFPTEIALAFSSVDGMPLKAYGEGNKTVFTIKNAASSSGGYVVLPKPIEAVTLEIELLSYTGTPCLKVEVLGCHRLACADVNECKENNGGCEQICVNSLGGHTCSCRDGFDLFTEDGQGGFHIREGESGKQAGDVYRLHQSCIPRVCPPLSSPSHGLLLSTKKSFSFPDVVRFQCEFGHQMFGAEFLRCGEEGNWNGTAPRCEPALCAGVANNTEIGLFVSPSDEVVAFGESVHVHCTQQSRPIRKQPLSDSRQCIYDPREGQADYWLAGPPVDCPLVECGAPPALAGVVYDGNTNNRKVGAQLTFACRPPYQLVGRSSHEDRQIRCTMDGSWDLGDLRCEGPVCVDPGFPDDGSVEMTSVEEGAVAKFACERPGFRPFPTSAIACQLGTPCLLAEDIGISNGYVHDGAFSDNSDKPNYGYEPHKARLSSTGWCGNKDAFIFLSVDLQRIHTLTTLRLAGVAETARLRGHVTKLQLFHKIQFSHNYDTYPVEFETPSGNHNALHQFELDPPLRARFILLGVSEYEEHPCLRFDVQGCLAPSHDSPSHLQVGWNSSVPQCIDAEPPSFIDCPEEPIQVQTDSFGQLAPADYAVPKAIDNSGRVAWVRVEPEHFEPPHAISADTDVVYTAFDDAGNSAECVIKLRIPDMQPPLLRCPDSYTVHAEKGESVQLVFNSSLVPLHVQDVSNISSLVFSPTHANLKMGEHATITVTATDAAGNRAECRFQVAHKAPPCSPHSLSAGNTTLRTCTEKGEGKLECAISCAEGHRFVDEAEVVTQFTCEDGEWSPAVVAPSCAPLAEEPARYELSVRVEX

>Ppa|PPA27669

MLSRRSDRDHLSLPVISRAPLYCPGHVTFVSLRITRPTMLRFLISASLILGCAFAACPQNFSLVRNGECYRQTDAQYNAYAPQAVELVTGKCDDLKAQPVIVRNQEDHDYWLNVANRDNKEQKVYGNVFIGLVCNTDTRVWEWSDGSELTFMPANHVSSLDSRCDTNCMWAIDPSTQNWREWCNGLYVYIDIYCVVTPPTAPSHPDEDCTDFDHDEDDDVCYQVGNSLANWTEANTVCRSFGSNVASIHNSQENNFIRRLAVSKGLVNGLMLGGAPTGKENTFGWIDGSDFDYENFIPGFPIEGFGDCLAMETNNVNGPWMNIDCTTQLPFACSRKTTDQEPVCDGAMRKEGDIIYSPGFPYNSSLPCDFLLKVDTGMLVEIEILMLEANSCCDHLVLTEGTLGGAVIADLTGEISTGKTFRTTSQNIMRASWQPHGGVNVKGMMIQFKGMLLLFSLLLLPSFIDASAEQLAKQYLFNFSTFNQQKYIEGIMAASLNDQVDMLNFACKTFFCHLQEENTKCIAQCKDFAKLNVDEKLVDTLLTADFPKPTTSEAACNNRFNSLFHMTYDFASLCKFHYKCEKHELYEKEFGRFVRPHLPFFRQQKTFKKHLDSLKSVNKTVPEEKPKWSLLLLLNSLSMRCHLLTSISRMVEEMGPQLLLHCIVALNCLETVWELFLVYRQRRKHESAVSRPPGVESIISEEDYDKARSYSIDRLSFAVVRMVIECALMITMLYSGYYYHLWNIATSTVAPQCAFLILHYATTYLLDLPLSLYENFVIEEAHGFNKYTGSFYMLDSIKKLVLTTFITIPVASGAVWLIDNSGDLFFINLWVFISIVILLAMTIYPAYIAPLFDQYSPLPDGDLKRSIEELAAKLKYPLKKIFVVDGSTRSGHSNAYLFGFWKNKQIVLYDTLLSGEEKRKVYEALGKVDEDKEGKEKSDDDKGMGVEEVVAVVGHELGHWALSHTVWNLCVAELNILLTLFCFSHFYTNETLGAAFGFTGGAPTLISLLVIMQHVMGVYNEMFGLLSVSLTRRMEFEADAFAASLGLGSKLTTALIRLSKDNLSVPVNDSLYSLCRHTHPPVTERIDALKKMM

>Ppa|PPA27907

MRSLLALTLVVSAALAAGAPRHTLTPNFYDHLEAQSPNEHHRAKRAIGGFRSPCPEGWTGQFCENPICGNEMKVDPYETDLDKTQDILYLPSGCDGEYYVPYDSTTDLLTITVTTDAMTKATAVLTGPTGDTPNAISSDYAGRTATYRFKAKPNPYVLTIKMDSHTEECLVHIQARSSLSFNGGWVASPMIDNSPFGESLYNSKPQYFIVHPFNMAAPAEVKTVTIRQAQLTQPDYRSLLTRRYDCAFEYYAGQFQCKGQDSRYLYQIEGVDSKGYAFRRSRPFLCLQDQTPTVGPPTGVPTIPTTECFNGGSLLYANETDTSFCFCPELMTGKQCEEVLCMNGGIQVDGHKDECDCIPGFVGKNCDSVVCDPDVGKLLTDTKTLAVVVRNTASMKDHIQKISAAIDTEVLHHQIDGRTVYDGFILSYVRDGVVTAKSYAMQKFAQFQADLENLAAKYGSDTACTESLRIMDQSPIFIFTDATADPGDSDQFKDLMRLNAANRMQLHTMFMNTQANCKVNPNLEFYNRLAKYTAGLVHKPPADKLEDTFQMSMRATTYHMNLVESGDLKQCSMKQKSLLVDFESATLILIATGTDLSVTVTDPDWQVSMKTTVYDDGWTHIFNIDRPTGGEWIYAIQGDDLISPCSYRFYSQSDFDLFLGTTNAVDVDERTFEPVVGQAAHLIAQVNGFYGGYVTDQFRLFAEILITSPDEDDKHQPLYYSNGIFRSSCGFHLYFGVANFCQSEDQIFYATVFVDDANGFPVQRAAVGYCNAADIDPPLPDDCQNGGVRYNETACWCPSHFAGDLCQTPECLNGGRLNNDQNQCICPGGVTGRFCEMMECTTKADEWEATFDHRTLNFVVSTRSSMKDTVKKLADGAAAFAGYYNLNHKDWIDHFAIVTVNQTSADLHLTNSPQEFADMFREISNNFDSYTNYDDDNCTVLINAGINSALAASYENSNIFIFADSQAEKLEFPNILSLLDRADELHSTVTLFGTSYDMCDGSKSFGEQEQNVVDFTGGKTFYTDNIDRVFDYVDTFYYSGVVLEQTYDKCNEGVSIDFPLEASAHSVVVTVEGKGSGIVMSTPDNFDGLYHDQLLSGNEFFIQQYIQACPDQWDTTDNQCYFWDVTEVQWITSLVACGNMDPNKGASLVTIFNDAKHDQVQAELKTAAQGVWIALYRDSQSNKWQWMQAGAQPVPLDPSLEKYFAAGQDMNDMSKRYVYMKNDGKWYIDDTTGRHWTLCQRDRYGGTYNPESQEPDLPPGFWNLQVTSSSTTCTVNVRTQSDVQIAFGFSQNPHDDFKRGTANVDSDSNYVVAKAIGLDTFDFNPDIPEGKVDYATIAADGKDLYPLVMTPRAQSCTFDTISQIPFTCPSQQGLSEIVVKFQGIDQFGYSFERSLVTRCDTYAVRCKNDGFVNNGECVCPPHYNGDDCGVPICENGGTALWDGSCKCPPLYSGKFCSYIQCDPAYPDTFKRNERTLVIALETSFTTSTPIFYLSNQLVDSLDILTQKRPDWFDTFVVVPFDSTSNKDKWYKPITTYNYKDLDDYLNDIPTATCPGDDPDANPPISCPDQNCPRPIGSVLGDILDRPDVSRPDSVVIVITNSAIEDHNNIYDIVPKLINSKAQVHFVVAPSATPCNLGWDNEMNQAMTYMASFSSGTVSSVVPKNIGKYFSDFLPSMYNAQEVVIDSQYVKDCSAKENVFQIDHSANEFNMQFFGVEADTISITGPLGDVTVPPNLLPGSSAYFGVFEVDNNVIVPGTYIVRTKSAGTNCDLKVRSSTKILVDVGFTQVHDTIGGNSQDDAHYAGVYGVPNKVMFHVEGLEEGGALMYAQVISPRGDVVATKPVLARVSDCTYAYVMDGTFSCDYTDLEIVVYGVDNQNSPFHRNFKAHCTDDRPQPIPPMPTCDLGAMKADIAFLVDTSVEQGYVNWFQLFITQMMSGKFTLGREQTQFAVMSISDKPEQGGFFSFRFGSDKSTMTQMLGSIHSDGHVGQNITSSIPAMMADIFTKNNGFRGDDKSVKKLLIYLSSTNPTDDEPDNAFFALTSNKLTGVAVATFRLANPDKKMKSLVEPHCFWDSGPDGNAFAVYGPRFMQNLLCATQPICGIPPPHNNCSAPSPTCALGSIKTDLAFLLDTSVDAASVERFRNMISAAIKEYSISSSYSQIAVMSLSDQPMQGGSFSFANGNTKTNVNSNLQTIKSDGHKGQDLTSAVPVLMREFYSKNGGYRAGNNDVKKLLVYVTATEPTDNDPTNAILTMTLSETTGFAVITSDIASPSKKLRGLADPSCTYNTASYDDLLKNAPNFINNILCAQPSASLPSPLWHFANNNSAARESQRRL

>Ppa|PPA27964

MTLLALSIGVCCLAATAAGGALHLGLAGRQQADADPCPLGYSAVNGTDDCFKVYTMAKAYADAEDFCRQDGGHLASIHSADERRNLNDLLGPNSPLIGIKCSTTTSCTWSDGTPMDYQNFIYGQPTLEYGSCAHLFADDDKWYSWNCATPMNTFICRLPATAPQCHLASIHDLTTNYGLSNLISNTTLTCDNQMQLNCNVHIGARYSQSAGYWWTDGTPYDFRNWATPEFPDTHFGQCSQMLNTDEFGAPGQWTNIPCDTKLPYVCMKAPGVSPTYAPLACPRMQYFEDQGTVYSPGFPVVIPSKLYCEYLLAGDLDTTLSITFPFFNIGDDSKLSIYDGMSAAYPNYVLSGSDFAGSTFRSTQNIMKLVFETPYNTDGEGWEAAFTTIRPYTTARPMTTAVSSTTTTLPIILSSAAGTTVNSNCPPVTAYADAQISSPGWPLGYPRNANCWFYLSTQPGKRLMIDFEYIDTKRNKDYVSIRDGPFDYSTEIARVSGKTSENTLRYVSTSNYLTLQFVSDSAKGGTGWTATVDGCVGYALANMIGLLALVSLLALSQGCPDASWTTIDGGDCFKIYEISKTYYDAEDYCVGQGGHLASIHSQYELYDLHPLIGSRAPLIGLKCSTTADCVWTDGTPYDYANFIYGIPTLEYGSCVHLYGTDDQFYSWNCATPMNSFMCKIANITDVCSPGYTPYLGSCLTVKTIYKTADDAERECAVEGGHLASIHDPAANNFLTNLLLDANLTKNAHIGIRDNADVITWTDYSAYNYRNWATYYPNTYFGYCGQLLGTEEFGYIGQWSNIPCDTKLPYICMKAQGVNPMYAPLQCPSMQYFEDAGTVYSPGFPQTVPPNSNCEYLLASQVGTTISVSFPYFAIDSSSKLKLYDGMSESYPIAVLSGALPTGTEFNTTQNILKMISSPGYPLGYPQNADCVYYVSGNPGKKLVIDFTYVNTKKDKDYISIRDGPYENSLEIGRLSGDTLQNQLRYVTSTNYVTIIFHTDGTKGGAGWVTTIYTEKFAKSECKSLNVFSRSVVYNECEEKEEPGCGDYCGRFAQTTDAICKNSKNRRGVIIKTLTSRFPRLHCVPENVHDIAKTNRADEKDDELNVASKIKTTLEKSALTTLAGSFFSIIARWMFSPSIRPTRAHTINCTVEKRTAEV

>Ppa|PPA29746

MIRPCLLLAALSISRAQGDECPYRYDYTQSVDGGCFKLVSLEESLNFTEAQKYCENNIGSLVTILSNEELYFITDQAIALGWGDDPYWLGLSCKNGLLKWTEHCWYSYNNFYNYDDTTCPEGDENAHFIGSANDKYWRKAPDGMVTNKIVCSISSIWNGCLPSDPDNLGRMFATEGIILGVTALTVFVLTCAVCGCVCMLGRRYRKKKAKEEEEKQWRQAQLEAAQLSIQTNGEAVTPKALERTASMRYTPFPRAEGAGKEFDEWEIDRRHVSIDYTTKLGQGAFGSVYLGIVDNSNIPTTTGKSIIEQSALKKDNNAVAIKMLHESADKMQQMQFFEEIDLMKRLGYHERLVNMLACATQSEPALLIIEYCAHGDLLNYMRERRQIMLGSPEAISSIDRSKIITQKQQLMFCVQIAYGMEYLSQRGFVHRDIAARNILVDQHESCKIGDFGLCREVEKEDEHYHSRGGRLPLKWMSPEAIEKYDFTIASDVWSFGVLLFEIITLGGNPYPDWPSAEILNRLKRGRRMERPDNCTDHMFTVSEEDYYLRLNAQAMYYTDQSIFEQDVDIDDEETDETLRSMNAAAHR

>Ppa|PPA30435

MQSTVLVLAAIAGVALSACGSGWTQSGNKCFKLISALNTWSALNMACTYEGGRLASITSSAENEAVKSEATVPYRILTFPSGIAAGREIYIGGLSYQRGPWTWADNTPFGAYRNWAAGSEPAPTPARPCIKMNPSGQWFNNCCRTPPAAGVCSKSA

>Ppa|PPA30817

MKLVLLLAALPVLALSCADGFNTWLDGRCFRMNYYASAPYVTAFDQCARDNTRLPAINSQEENDQFFSALKQQNAFIDFSFWLSLTCDGSKFVWADGTEAEYTNFAANYTCTKSSMDRRYYVDTDRLWIEADQQSFYYAVNVIVCEALQRSSSPCDTYDALDATDTCYKLQKDEMSWKQAEETCNESGGHLAVIHNEIFNDFILKTAEVANLHDGVHIGIKRDDESSNYTWADGSDIDYNNFAPGFPIDDNGPCVAMQTGFIPGEWMNTDCYFTRLPFVCTKPAYSVTSQQPSGCPVKKQYAPGDEIFSPSYPLPPGVGGCQYLLLERDQNKRVAIEIDFFESNSCCDSLTIYDGLFGSNILQTITGYLSKPVTIRATSNAMRLVWNAKSGAHVRGFHAKMSTLSCGYGFDTWLDGRCFLVIDFSSAPYDTAFDRCARDDARLPAIRSHEENEQIFAALKKINAFADFSFWLSLSCDGSKFVWADGTEAEYTNFAANYTCTESSKDRLYYVDNHRLWQEAEEDFTLIYYYAVNYIVCEGLHRSSSPCDTYNLRDSTDTCYKLMRDGMTWMQAEEMCNESGGHLAVIHDQTLNDFIVQTADAANLLDRLHIGMKRDDKSGNFTWVDGSVIDYNDFVPGFPKDYNGPCVTMQTGFMPGMWMNTDCYYTRLPYVCTKNTYLTSIPQPAGCPVKTQYAPGDEAVVEIDFFESNSCCDSLTIYDGLAGSNILQTITGYLSKPVTIRGVSNAMRLVWNATSGTNVRGFHAKMSTLSCADGNETWLDGRCFRIFYPISVGYGTALDQCEGDNSRLPAIKNQQENEQIISALKKYNAFADFSFWLSLTCDGSKFVWADVIEAEYTNFAANCTCSKISKGHRYYIDKDGRWYGADGHSFDYPVNSVVCERLGFKQDLSIDNV

>Ppa|PPA31391

MQYQRKPLSLLQFISCRLALYKPERPFPLPPGPFVMSSLTLCLVLPYLIFIPTVSPGVIVVDHVLPSTLEDSFSSKPGERASNLDANLRLLTVLALQQNGWQLFRRHFYRFYELDLHWVPAENFCRSLGGHLVSIADEAENAFVHQLRKKSNIWIGLNKLNDSASQVYGWSDEADADFLNWDSSQPNEPKVDCAYMAYQQDERFGTWFDYSCDGALPNFFVCKTRAP

>Ppa|PPA33774

MPRPPHFGRPHLVYLLLLMMKMCNGLPSTTISQYTTTQEPASTDRSDYSTQYHVDDNTDQPFEPNIRPGPNEIDTTALTTEFATTEDGIERYACPEEFLQLTIDWCFHFRYNIDDRLDFTESQDYCSIKDAILPAITSEDVTKALMLERTTVMGISPFEGFYIGLRCNTKKHRWEWMGGVEYDSSLAVFARARTNPKYCVHWNDTVEYGSEFQFDQFGTWSLPNQMSKLASLVVCATRPKGYSPPNPIDAPCTDSWTQLPGGMCYRLYTIQYPDTGLDVYDAEMSCRSMGGHLPAIRSQDQSNLIQAYLTGKGKQNSQFWIGLYCNFGEDYMPGDTGVRAWIDGTPYTNDFTNFLEDSDDTVTCNVAVVDTFLYNGALKGKWQKENYTQLLDTIMCSRPDPEPMPGTSTATTRRTIPPPKTTTNAPEDTTDDGTTSSSDEPEKPEHTTTHAPTTSGSPDKPRGLTILELVLIIILVIIIIFVLVVILVYCIKRCRRAKVQEIFYNVQKRFSRSNTNAVVRTDEWEVKRQFVGIDYSRQLGRGAFGSVYLGRVFSGNIPEMAVKTLLQLNTLKKEDDFVAVKTLHETADRQAASEFLDEINIMKKIGFHERLVNLLACVTETEPLLLVVEYCSNGDLLKFMRERRMFMLKLKDTVGHDIAGRHSVITQKQQLMFGIQIAYGMEYLSQRGFVHRDLAARNILVDANETCKIGDFGLCRQISGESEQYISRGGRLPWKWMAPEALERFYFSVESDVWSFGVLLFEIITLGGNPYPEWPAVELLHRLKTGERMPRPEGCSNGFYEIMLDCWRCVPMQRPSFEKIRRKLAHELEESSSDDYYLKLDAAAKYYQGPASISVVNLNDKLQSSPIAVYVVQKAAADKITYGIYDVINMNPESSDYPQRESNLQPNSVTARLVGFDNALDGNVDECPVAYSTTKQALSFTVYVDGPIVSLSFAEKVGITARMQFIPSFDLAYPGFITSGGWNGCKKPASGGLQSFRSLFVNTDAVYLLTSQTDKYNVSMDIEPNLDKKNAITVYDLINRRDEEVFGYEKKALQFDNTSNFNIIFSGLKGDQGFLLRYSAVVLPRPITTAAPTTTSQTSTSTALTPTTTSGSSRLESMLLTLAALVVAGLI

>Ppa|PPA34500

LINSVLEESTRFVCLVPQDGYTGLNQLEKIFAQTDKVSTSFAILLSAQCIERPNESPWRIVAGLPVDLDCTQEFSLIFTSSQPNINVIREQANSYSVSDEEAIFVSSATGMELNTNACSGDGNVTIYTGAGSGVAEFRYPMVSWSISRGTRASRGDVKRSHLQNYHAERNFVKLFTCQDTQMTVMFSGSFDARYDNYVDVAGNSSNGMQWARTFRNETSSMSTVATNFTALYVSTPVDAPDLWDSQDNFVVVMDLTDPYCGCELDKKFGMPIDWDPNGIWLDMVIILDCSEAMGKKLAGRGNAFAAAQQMLQDGQTPGRSNARHVIYYMTDSDPKVDLTSLDSFKEEGVIIVNDFIDSHVIERPGLKDLASDGYYYTDIQDNYMSSIQLFCKGIVRLKCSANEMCWLASYRLSGREAGGRKGGGGHLIYRLLPVSSFLRPLERSSKVSYEWNGNILTIHDFIDRECLWLANCFCSAEADHVPYAGHNKDEAGEAAVLRRLLPRGACRVPYNSMIQICLELGGGQMVSIHDDNKAIFVNNLYKAAKLKPDYYWIGYAKSDDLWSWTDKNTVNNCAYVDSTSTYWGAANCNVGYPSVCEFKPCAVGHKKKKA

>Ppa|PPA34980

MVYTLKYYLTVVTVVLGGSAQFYSYGVVNPAQRLIMDWINQTYYDRNGDALGVTELNLFWSFVVSSIAVGAIVGALLTRVISERCGRRNGLILNGAVNVVGAVLELLAKPTRSPELLLVGRFVLGVNMGLTSGLVPMYLMEITPASSRGVAGTLHQVAVAFSDWFSLLIGLPEVLGSAVLWPFAFAFPGLFALLLVLVLPFSPESPKYLMSTKKDRMGALDACRSLVGHDAAQHMYEELVKEHIQEQDQSSATFRELFTSPQLRVPLAAAGIVMLAQQFTGCTAVFAYSTDMFVNAKLDQCVSCRLFHRVCSFCSFRKYRNTYTTNLRVTARFATLAIGIAYFVFTCSAPLLIERVGRRPLLLFQLSACFVSLLALTIFTGLQTYSKVSWASYGMIGALVVYMCVYGVGSPIPWMITGELFNQRFRSCAVTVSVFIAWFLAFLTSTAYLPFSQLVGVTFSYLPFLVGLLVSTTVMFFLLPETRHRAPSEILEEVRERSSSLASGRPWEAVTHTRPTSRQEMQSAHVNISKFNVLGNAMRFAYLLLLLLCAGGVRTYTFTCEEMLYHFNRGTIDDEITHACVILPDGNRNVEQLKKFHLTPHMTFADIFNAPGHCVKRANGVAWQPRVDDGAVCTPEFILIEAKQQPAIITPGAEPDQHVLDGQSVVLVVPQTGMWIGKSRCVGQGNVTFSTGADIEEPGLDMQYRSWPCHSIPVRIDVFDSVVTVKVDANVQFTMQTSSYIGSYNSARSGNHFAVFSSGRSNDRQNMEGYYNHANFKMDKEDEVTVNMDLTFDSANTGSVILKKSAGGVLENFYNGQYSENFNTKYFEIQWTPKPLKPNEIYYNQDKIVVDIFIGPQATAPPPVASKNEYCNCAVDKFGLPDGWKYNDIWLDVVIVLDTSEAMGERSLVDASALIESLISDGVDDLLITDPAGAFYTRIGVIAVADTAEILYNLNMTKTDKVTASVKQGVKEMDFNAAYSSALSMFSDGLTSQPNRAGTRQVVYFLTNSDPDCKRFAKPIASATCTMTPIQAPIPRFRLREVAIVPRPRAFPSPKLRVPVQLTEESLQPFMMMRRECFCTMMTKSASKSDYYWIGYEKSNQGEWEWEDQSTNPYTNWGPHEPSLASVAKCAYVDATTKNLTWGAGNCQIGFPYVCEFVPCSVGRKNCRANSGFRPIELGAYAFAVLDCSRYLAVLSLLEFASDRGEKKGKCPYYFEL

>Ppa|PPA35254

MNTTELDPIFKAFLVNYHHVSVVRGVIVFIANSLVSIVIIMDKDSRATTYRSYLLALQITSMTTDSLLDAYTPFILLNSHLVFSDSFLAQHLDIVSFFTIALFFFSTALSSYFYCVFYRRCVVLPAGSRFCWVGCSRYSLFIGLQVNVAVVTFMLVIVIISARVPAKDVPEDLSWLCSMPSFGLLNASPYFYTAAACSLNLNIFSQWYLKKFRNGSHNSDIILPSDSFPHIRMPRNEVETELLDCPADISHLSIRWQIKHPCSDAGSIAVFVFGSLVLMIWDVQRELRRGIKHASGATQRIQKRAITALTLQAWNDSECLFRHSCNRSPSTLSALLFAVLPMHTFMHSLTILACSPSYRRTIRDAVCRNKNARQRKSTTLFISPCCVFVVNSLVSIIIILDKDSRATSYRFYLLALQLTSMSMDILIDAYTPIFIINGRLIFSDSLLAKHVDIVPFFTIAVVFIGGAISSYFYCVFYRRCVVVPIGSRFRWFGWRRYSLFLAQQANVVIVALIVGVVASSARDVPAYLSWLHSMPSYILFNTSPYFYSAAAYAAASAIITFGAVVLMIWDVQNKLRRGIKHASGATQRVQKRAITALTLQGIIPSVCFVVPEVGLSLVYFRSLSQGLEVAAHDRRASTLSALLFAVLPLHTFMHSLTILACSPSYLPSMIMDLLIHLYNPILQMNSRVVYSDSMIANHVDIVVFFSFAAVFFCQSVTAYFYCVYYRRCAVLPRGNRFCWEGWRRNSLFLALHVYVWIVLAVIYISLYSVRAPVEEVPTALNWLRYTSSFIFVREQPAFYVAVGFVLFWGFSMASVVILMIIEVNRELRTGMINASSSTRKYQKLAVTALTLQGKIPRAFYTLPIVGEVLIYLTSLSMGLEAASENATAISGGLLLSMFPVRTFVHSLTIVACTPSYRAICFLFNVLVFTVILIDKDSRAKHYRIYLASLQLSSIPMDLLIDSFTPMLLINCRAIYYNTFIAKYVDILTDIAVGTRNGRNAFALSSTRTEKWHAACKSCNEKGVIPSAFYMIPATSVDTAGAKFGSTMSVLFFSMLSLHTFAHSITILACSPAYPRKTIRNMAPWCFKGESSISATAVFLVFQSASYELSLIYTDMRFIESISILLLLVGICEAFVFSCNEVKYKLINQNLEENTRYVCLVPANGYTNLEQLKKIYAQADKVSTSFFDLLDSQCIERPNEGSWRIVADNPVSLDCTREFSLIFTSSKPAIYTTYDESAVSLSGTEVIVVNAEAGIMMRMEECVGEGNVTVYTGAGSGVQEYRYEMASWRCSDVPTWIVSFDNVITVATDSGNLIVTPKSSLYHHYNITRVSPLDRITVMSSGRSDNLQNMTPANNFIDFNMGGTVPITMNCTVTFDKRYYGSVLAYTIKPENDHSFDYDINTWNTYGQAIEVKYLTDPVAPADLWDSSDNFVCDIRFGDYIEQPTTDAPTTLTPTTRAQTTPPAPTADADPYCTCTLDPKFENPLDFDPKQIWLDVVIILDITEEMGSDSIDEATILVEQFIGDGDFDVLITDPKAPFYTRVGLIAMSDRAEVIYNLNMTKADSVSDKVAMKKGVKQIDVVAAFDAAQQMLIDGEKPERSNTKQVIYYMTDGDPKFNPTTIDVFKQYGTIIVNDFIDSGVLEHPGLKNLSSPGYYRTDIQNSYTRTIQLFCKANCFCRQDIDRKPYAGHNEDIAVQAAGGCFHAAPAGVSYSKMVSTCNNLEGGGKIVSIHDMDKAQFVNTVYNNASIAKDYYWIGYAKDDAGKWNWEDASTNPWTNWDTANGEPSLNSVSKCAYVDRTTGNLLWGAGNCQTGFPSVCEFKPCMVGNANC

>Ppa|PPA36768

MLVLLLALIGTCAALCPDGYLQISRTDCYTIYFTNQTTFVAAEADCVLDGGHLASLHSYDEQHSLAGMTYGMNPLIGMKCDDEAIANCTWTDGSVVDYQNFPGGVPVLSYGGCVRLGSNDEMWYSWNCAAPTDSFLCKVPAYDDIVTIPVEMTTAEPVPTTPIDHIKGCPKQQYTADTYVFSPNWPSPYQAGVDCLYFITAKNSRKLTIQFEYVETNDMIIVYDGSDMQSRVIANVTGKNGAAVWQYYSTGSTLTLDFIANSTTGGKGWSAAVYNV

>Ppa|PPA37151

MTSSLQLFIILSAILNSSVAVVNFTKSILYDEIDFATQVVYVPNFCSEGCRIYVSVQEASASIAKNIVVHDYIHDQFSLYDISQQVESDGQKGYSAVDVGNAQVNIINTNLGYATAPLAVWIVRKDATNFGYSRVFEADNLSTAPSSLQVVSIMSAVPFTLRTKTQGSTELISTLTGFDAVNTSEDECIQVITEMDFDTFLDVQTFVQSPLITLFFNDEKRPTTSLSTETGYGNDLDLSGVSFVASPGFIGCKGNQTFRSSLYDDYTSVNYSNHQVTIKDTTNDKEYKLISPGGTSATNITISQTNILDISWTLQDSLSSSNNSFLLRLTPSKETMITPISTTATKPVQHSTTPVSVPSTTTKPAQGSALHSCPSPLILYRDEQCRGYYASDMEYMDDSAGQVISCCIKINAHPVSIHTDEQQDYWSAQKHERYFILGLTCNSRSKHWEWADGSVLDYKPPTFDKEMDSDCNTGCNWYMESDGAWRINCTHVVQETDFFCIAQLSQPTPTTDCGSFIDGSDDSQCYEQKRRPGTKLARSVTVSELNSLPFTVNSSKIIQQNSYIRRLAVSRGAINGVLLGGTVSGKGKTFGWVDGSEWDYANFHPGFPIDGLGDCLSMDTITTAGQWMNTDCNFKMPVVCSRPFYDTPELAGCSAGSWNEGQIIYSPGFPSDASVPCEYFLTVSAGKRVQLESNRRGQRSNVHYEGHKHNEGNLATEWRRECERIDDVVHYILNISFSYHSYSFCCNKEDCARCVLRCLATIACICIIKSARHYHKAAKLLAEQAIDVEMLKAIVFLQLLILTFHSVFGGCPSQLPLYRAGQCRGWKKNDDPYMDADALNRVTSECNSIQSVPVSIHDDEQLSYWVGKKTGYKFVLGLTCNTGSKKWAWSDGSILDFKPPSSQYATDCTHNWEYTDYFCIKQLVQPTQSNDCGNFDEDSNDGTCYEIAKIAETWNDAQSICHSFGAEVASVHSEQENSYIRRLAVSQGSTNGVLLGGKVAGKGNDFGWIDNSDWDYSNFYPSFPIDGLGECLTMDTLTTAGQWMNTDCASKLPVVCKRPFYNSPELAGCTGGPWKEGQIIYSPGFPDDASVPCDYFLTVPTGKLVKLEILLLEANGCCDHLLLTDGYTGGAIIANLTGDGLGGKYYYTTVSNTMRVSWQPNGGVGVRGLMCQLLRDIEMLMVFFVALLQGVHASCPSGFELIRNGECRGTVGRQNYYYDEAMSNAVAECKSIAGQIPIIHDDEDQSYWNNKEYFYIPLGLVCNTQSKLWEWIDGSHVDYKPPAGNYDAGGRPHMRPFAVNGVYLGATLSGKAKGFGWIDGSEWDYENFYPGFPVDGLGDCLSMDTFGVAGQWMNMDCSAKLPVACVRKPNTSYADSCSAGPWKEGQIIYSPGYPYDASIPSDYFLTVDMGKRVEVKVHLLEANSCCDRLLLNDSYLAGNIVANLTGEISEKTYTTKSSNLVRVSWQPNGGVNVRGFMMSYRAV

>Ppa|PPA37162

MLIQIDSLTPGQQYRVYAAQSAIVLAGEYVGNVVIKGVDGKQFDVQSLTKPIPNTGYVLAETNVLTAPITIADSMVPGANDKLVPFVIYIVSTKIPASPVISAQLAGSHVDSSIAKMSADSCTVLSNAIRMELSELNISPNGELMVKSAGYDATSHDYTVLSVNQNAVGCKLTVEGPIASLHNPLGSSANFTFTLAFDTPPHTILAPGASAVFLSQGWLSKHESCETLHGIISSIGHPCTTVNVTIDQGLIREAPRFMVQIESVDLLDILTTTERPATNMKPDPYCNCTVDKSGAPNGWRYNDIWLDVVVIVDATAAMGVDDVANAEYLVDSLISDGVSDFLITDPTKTYSTRIGVIEMTDSARVLYNLNMTKTDKIQGKITMKMGVEGINVIDAFDAAMNMFNGASTPDRTYTRQVIFYITDSNPTQDLASLNKFKQKGIIIVNSAVHRTGLWNLASEGYFFIESSTKHVLRAFCKANCFCKPGKQAYGGSDPAITAGGGCYSSYPKGSSFSNAQSTCASEGGIATDHDGEKGAFLNRGEFAILRNHRMDDDVSALKDASSKSDYFWIGFSKSDDGSWKWEDKAGFTTEINSTDSYTNWNEGEPNSASVSKCAYVDSTTPGLSWGSGNCNVAFPFVCEDIEVDSLSPGQQYRVYATQSAQNSAGEYVGNVVITGADGTHFELSLAKPIPNTGYVLAETNILTAPITIADTTEPAKNVKLVPFVIYIVSTKISASPVISALLAGSHVDSSITKVGFLGELTVSTAGYDSFSQDYTVLSVNKYQTGSKLTVEGPIVTLNNPAASPANFTFDLSFDAAPHGHLAPGAMTVFLSQGWLSEQASYDSDYPLDTAQFSSCTLDGIIFVLEPPCTSINLTVDKALINGAPPRFMVQIETIDFLALQTTTERTPMSKPADPYCNCAVDKQWAPDGWRYNDIWLDIVIIVDASEAMDEEPLANAIYLVDTLISDGYSDFLITDTTNPFSTRIAVIEMTDTAKVLYNLNMTKADKVQGKIATKKGRLQRSHGHVQRRFNTGQGQHSPNCILPHRHGTELHFAVCFRKQDLTPLNQFKQSKGVIIVNSGLQSSSLLSLASKGYYFLNLNNKHALRAFCKANCFCKPGKQAYGGSDPAITAGGGCYSSYPKGFSFSNAQTTCASEGGIIAVDHDGKKGEFLNRGEFTTLRNNRMKDGIAVLIDSSSKSDYFWIGFSKSDDGSWKREDKSIDSYTNWNVGEPNSATVSKCVYVDTTSSNLAWGAGNCNVAFPFVCEDLPCSLSNGQPIVNQKVFPALSTHLPNLRLQEMLSSQTWNNKLTTEAEKVAAHCEPTPSSPGQNTLYFPGTVHLFSKSSMVSYSAKYWCVEMVYRAWRSMNRTVPIEDSGVTEAIQMARPETREVGCAVSFCGVDDESALVAAATPPIPRDSPSATLSRRAQVREESSRPTTKTRKEPSLIKSTDSYTNWEADEPNSAAVAKCAYVDSSTKDLACQNAVGSKLTIEGPIATLHNSLGSIANFTFTLEHNVASDRDLTPGALTSILSQGWLTLNKSYDSDYPHDPAPFGRRYNFGRPARIRFSTENNFPLGISCELWCERGKEAPIGPYTYNDIWLDVAVIVDASEAMNDAALKSAGALVDSLISDGISDFLITDTKKKFSTRIGVIEMTDSARVLYNLNMTNADKIQGKIAIKKGVKEINVIDAFNAATNMFNGAPISDRAYTRQVVFYITETNPHNSKKKIFLKVYCTSPRKIE

>Ppa|PPA37170

MRVSWQPTDGKNVRGAMVSELAMSMNRTALNALQGMNNDYATIKKDLSLALRVGTNNYDNYNEQIQLMTSKVLYAITDLLLAGFENIELGKQFDLTYKLVDAYSTMNKCQNPKDPLQICLFKFARCFILFMDNLHGKSLLKGEEAHEKRLMPSVWVDNEIAKRNAPVKQPPIRSSKSTSIGVVRNIGAQQVVVINDRPGPSGCSQIEPAPYLPVPVVVTKRRLVPAKRVMPPAPSLEKDSPSFFEDPETVKKVKTGEEDEAWMRMAEQPSTSARNDAPPSFLAHPPKEEAEDGRINQSTSYHSAAPSTSTQPIKEEVEDNYNYGDVKMEIEIKEEEEEEMDGPIADTVKLSELCSSTNGFYDPTSSTSQPVVSSSSRRKQKITLSPPQPECSGFATAEAQKLFFQSPVNCPYCGVEIPDGKKLEQHMKINHRDHWLKYVQKCPVNICDFRSSDPSVVQRHHVMVHSRNYNTRMGNVAVNFKFVATCPFCPDPLRGLAGFVNHMEKKHPRLCTYEAKILAYAECRYAASNVHMLLSHWLKKVPMCTQGLRFNYDIAANTKINDAVRERDERARMSTIVLDNHNVIQHFYLAVAEHRNAHIHDIDFYSGLMCNTNTSKFERLDGSATDFHAENFHFDTGNCNPACSMIIDENAYWFGWCGSDKAEADVYCTYQLPEPAPPPSGCANFDDDTEDGTCYEVINTVEDWQDAQLTCRSIGADLASIHNERENSFIRRLAVSQGAINGVFIGATVTGKGKQFGWIDGSDWDYENFYPGFPVDGIGECLAMDTLSKSGEWMNMDCSTNLAVVCARKEDPKPICPPGPWTEGQVISSPGYPYDASVPCDYFLSVDAGKRVQVEVGVVLCADWIRIQVLEANSCCDYVLLTDNLLGGNIVVNLTGALSNQTYTTHSSNFMRVSWQPDGGLNVRGMMITFKGVIVEPLMHYYD

>Ppa|PPA37173

MFHIHLTADDLYECFRDHSQKATRSTLSVMKRWFVLFALLHLALSSCPNPGYDLVRDGECRGLEDETTVSFNKARLFLPDLYSKGAAIEAVETCANARATPVIIHNEEHQSYWRSQAAATKNFFLILGLVCNTTTKRYTWADGSQVDFKPSEGYAKDLEGDCSPLYSWYLQSNGVWGIKGGFATGNPALVSIYCTTQLTPPVRSADGCDAFGDDGEDGVCYFVSKFASHNVQAAQSGCMMAGGNLASVHNAQENAFIRRMAVNQRANYGVFIGGSAGKDGNFSWVDGSPMDYENYKPGKFNLSFTYFLLYIGFPKAGAGDCLSLDTSAASGQWSNVDCLSGLPAACMRDQRPVTQPTCVSEGYKENTIIASPGFPFDASTPCDFLLTVDQGRKVQVNILQIEANSCCDHLVLYDGAEGGNVIANLTGGEIHGETFATRQSNVMKVSWQPNGGVNVMGFT

>Ppa|PPA37343

MRVPLLLLVHVGSVLSTLVCPPKYFSIEGRCIRPYTLNANDYLDIIMDYAQAACALDGATLPIIRSDEDNDMFTRIAQTFLQPSGTRIYLVLGVVCDEETDMTRWPENQWKRLLLGDAHLFTSLCVLEEQIATTDEVTEKPDNKCGDYERMEEGMDDETPCFKVFTEPQSWENAQKKCEADFGSLIAISSAEENSFFWNVATTHKFTGGMHIGAHQCPDDSTTWTWIDGEIPITSNTYNNFIRSFPIAGSGKCASMVTESVAAAWVNVDCYEDELPFICQRGDFSLIPSSCPNAAPKAGEEIFSPSYPKSDISCEYFLTVSEDKLVEVEIISLISEKNIDYLEIREGTSGMKLLANLTGTLEKPTKFTTSKSNVLRVNWKPVGTADGRGFKIRYTEVDKVDVGQPATVPVQDEVTTTKSATPQGIFAVIATLLFLHFVSTTAVTQLSCPPNYFFFEDKCIRPFAVMADDTLLNLLPLARESCAQDGAHLPMIRSHEENESYARLVNLLTAPPKGRMIRTNLMDLPNEVLVNIFDFCRHPQSIQIEIKHATRSGSTLRFSGNMYSNGADLPHNPPDMNAFVQDYTYFNRLRFGLQRLAQNTRVQDLRIQMQPMMTDAVNYIIRVLLSFEITNTILWCPESTWNQEIPTITKGPAHGTTMWGGHIQKAVQLWSADSQAHSCPKAAYSLTGQRGERVCEMKRRRPVFRTLHCTDDSIEDVAESCTLDLEISSDCDRGLRFHYCTMTLHKPSGELDWTRTGKWRFLLSSSPIFTLPCLARNGTSVTVVCQERHCNDMNLDANIELCTKELLRRNEAGNQHSSTCDANSASDNQTLSCLCVEGLLIVFLLQLLQYVHAKRNAAKLEKMRHKLNVSSRANGNSENYEEYDSASELHCNKEIVCGDQKYVLSEEDCNRGLVNQYCTKTLIRSSNDNQTNNVDLKFTTKELLPDYSYVWQTEKGTLYYAKFVVNWVLYVMFEGKKVTTKLPAEFGMESVSSLGVRKIIYIGQIDQEVAVEQFNADILIIKIPMTPKYKWSFGHDSSPYFYITNGESLFTINTDTRKILTTLKFRGVNSLVYIAGVFGSEISLMVNQDGWKLISAQLPDGYFVDVSIAGDNNGAIAYTEDRCASTGWKRTNGWELLLSSEPIANVPCLVMNETTMIIFCDSKQCKASHYYFFTRTFNVFKDSRLDMDIKNCADKCKERMTKMGNDEAIRAGRSIQHEDNIDVVCPLNSLHSSTHLLNAHRDAARVEKMQKEIFLNTALPEIPRSSSRQFIDNDDPNSEMVDY

>Ppa|PPA37365

MAVFLLPSQSFQCRRHSHQSTMSTNTIFLLVFTAIYPLSAQSVGTTQSSGTTQCPDQFTSYWDGACLAFKKPDTAKDFIGAQAVCQNIKGNLVTILTKPENDFIKDKVNKTFGASAVFWLGLQCSKGELEWTEDCPMNGYANFAEPDKTICNNNDAHFFVSNALDVIWRTAEDGFVTDAIVCSYRTDQGCKQPDPHTPSADDLTTLHVNELIIISTSLVVVIVLLGAVCACFWQIGRRHRLEKRKAEDDRLKKECVEYLREANIDLNIEAGKFKFQVGKAAAAAGAADKAIERTASMRYTPFPRAEGAGKDFDEWEIDRRHVSIDYTTKLGQGAFGNVYLGIVDSSNIPTTSEKSIIEQSALRKDNNAQMQFFEEIDLMKRLGYHERLVNMIACATQSEPALLIIEYCVHGDLLNYMRERRQFMLGSPEGISSIDRSKIITQKQQLMFCVQIAYGMEYLSQRGFVHRDIAARNILVDQHSSCKIGDFGLCREVERQDEHYHSRGGRLPLKWMSPEAIERYDFSIASDVWAFGVLLFEIITLGGNPYPDWPAAEILTRLKRGRRMDRPDNCTDHMFTVMNTCWQYKPENRPNFSDLRQKMGVALEEVSEDDYYLKLNARALYYCTQKTSPSDSECPDAFMQLTPDWCFHSHFDPDQIMGFKEAQNYCSQKNAALPAITSAEVTLALIRERSPAMGLSPFDVFFIDLGCDLMNHRWTWIDGPVFNSSYTHFQETLTNPQMCSLDRPFFFDHDGKWVSTSRYAGDFPTLLVVCVVRPASYMEPAFTDAPTTDAIQSTVDTDISVTSNTFSSTADGTISKHMPNTSTASISTTCTGLWTELPGGLCYRLVTLAAPVNIHDAELSCRSFGGHLPSITSNEQSNLIEAYLAGKGKQHSRIWIGLYCQVSVDLPSETDGYRAWIDNTPYSDEYTNFIDEDDDSKFCNISQTTTFIYNGVLQGKWQKAHADTELHVLMCTREYAFFSIPPSPFRILTFDYRSMCGVRKRPGGGKNPQHVSTSSTPWATNSQTTDDNDILYFTVAPQSTTNPTTTAVELSTTSASQRGAYSLHLLIAMASVSKLNSLI

>Ppa|PPA37381

MRALLLFAVLAGSALATLVCPPKYFSIEDRCIRPYTLNANDYLDIIMDYAQAACALDGAHLPIIRSDEDNDMFTRIAQTFLQPSGTWIYLVLGVVCDDETEMTRWVDGSPIEYQGPPGSDPALFNCSLSPTMPTSMLPENQWKRLLLGDAHLFTSLCVLEEQIATTEEVVTEAPDNKCGDYERMEDGMDDDTPCFKVFTEPLSWENAQKKCEADFGSLIAISSAEENSFFWNVATTHKFTGGMHIGAHQCPDDSTTWTWIDGEIPITSNTYNNFIRSFPIAGSGKCASMVTESVAAAWVNVDCYEDELPFICQRGDFSLIPSSCPNAAPKAGEEIFSPSYPKSDISCEYFLTVSEDKLVEVEIISLISEKNVDYLEIREGTSGMKSLANLTGTLQNPTKFTTSKSNVLRVNWKPVGTTDGRGFKIRYIEVDKTDIVEPATVPVHDEETTTKSATPQGIFAVIVTLLYSLTTRFAKRLPRAAPIKAFTCERIRMRALLLVALHFVSTTALTQLSWPLNYFFFEDKCIRPFAVMADDTLLNLLPLARESCAQDGAHLPMIRSHEENESYARLVNLLTAPPKGRMIRVPLDLVCDANTTLMTWADGTSVDYVPYTSDGMKFDCTQYNHSVYTEPMYVTSTVYRWTRVELDTVMSYTSLCVHDSEVVQDVTTSSATSATTLLALLVAILRYDSSRNGAPSLHTLSSVRTMIESLSMRSVFFLFGFAAIGAATPPHLSNHSSAKILPIDSVSAASDDVQCPSGFTDYLGWCAYLFHESFMYSEAAQFCSNKGTYAPSIHSESDYNFWNLFAHINEFWFDAYCPNQGEPYAWLDKTPTNYYGPKNELGNCQMDKGMQMLPSGLHAEKLTIGAYALCSAPTATSGPTTGDDVQCASGFSNFRGWCVYQSPEKMTYPEAVDVCASMGTFAPSIHSESEYNFWNLFGYTHDYWFDAYCPSEGQPYAWLDKTPTNYLGEHNELSNCQKDWGIQMMVFGLAAFPYDFDASPLCVYEASNPPANLATTTLQTVQPTATPPATSGFTTESYCDCNVDKFGLPDGWNPSEIWLDIVIVLDISMAMGQDQLDDAEVLIESLLSDGLTDFLTTDVQAKFFTRLGVITMPDNKSGATPLPDLINIIASDGYYFMDSNNMQGLQAFCKANCFCGSPDMIVNGDSASGGCYHASLSGVPFNKAKAACSNNFHGFLATVHNEDKGRFLQKLLNSSKSDYFWIGYEKTDDGVWQWEDQSSDSYTNWDEDEPSSSSVAKCAYIDSSKTTLPWGAGNCQVGFPYVCQSIPCSAGHIDCM

>Ppa|PPA37386

MWKLLLFCAIAQVVHGACPTGFDLIGNECRGTYAKTLLGLNNVTETTKKMCSGILGQPVIIHNEEEQSYWAKRVQISPSKPGDPMLVLGLTCEGNEYRWADGSPMDYLPWNYDTMLKSYPCKSNIRFYIDSDGSWLSWGGSAYTVDVSCTTKLNQPVPSGNGCDSFEDDSEDGVCYQIFETAERWEVAQNICKKYGAKVASIHNSQENSFIRRLAVSKGALNGVFLGATISENGKDFGWVDGSNVTYENYYPGFPIPGSGNCLAMDTSKSAGQWMNIDCSTSLPVACIREQKEVTEPTCNGGLSIEGTVINSPGFPYNASTPCDYFLQVNPQQRVEVEISLEANPCCDFLVIHAGFLGGEMIANITGEQRNLTFYSTSNLMRVSWEPNSGENVRGLAVVHGACPTGFDLIGNECRGTYAKTLLGLNNVTETTKKMCSGILGQPVIIHNEEEQSYWAKRVQISPSKPGDPMLVLGLTCEGTKYRWADGSPMDYLPCNYDTMLKSYPCKSNIRFYIDSDGSWLSWGGSAYTVDVSCTTKLNQPVPSGNGCDSFEDDSEDGVCYQIFETAERWEVAQNICKKYGAKVASIHNSQENSFIRRLAVSKGALNGVFLGATISENGKDFGWVDGSNVTYENYYPGFPIPGIGNCLAMDTSKSAGQWMNIDCSTSLPVACIREQKEVTEPTCNGGLSIEGTVINSPGFPYNASTPCDYFLQVNPQQRVEVEISLEANPCCDFLVIHAGFLGGEMIANITGEQRNLTFYSTSNLMRVSWEPNSGENVRGLAMKFRGV

>Ppa|PPA37408

MRYVIFSLLILSFAHCDPCPDGFFQAPNGGDCFKTIKFYDSWANYYTPTSFEEAEEACSNWNSGGLLASIHNFDENEAIQSDAMRTCGVSYATLNIGLKCKGKDCKWDDGSPVIYTNFAGDGPSENDGESCYAIYGNQGEWIRKTCGTQKADCWICRVKARTIDCVDGEIEYKGGCVSIQTSPLDQKSAEESCPGGGHPVSIHSANENKFYVNAAVDAGITGTIYIGGQYSKEMFEWADSTLQGFMKWANGFPNPVFGSCVQILLDSEFGVQGQWTNIDCSTKQPFICYRDGAVYNPADIYPKEGPSCPFGAYYEEKGEYKIGNLYSPNFPLSLQSSQDCTYVLNTVSVTLASINFTTFDCQSGVTLELRDPGDPIDPNTPFITFNSTSPPVIGQYYSSGSKEMEIQFTTDSSPVGTGWAAKYTGIYAGDN

>Ppa|PPA38230

MKVILLLAALPVLVLSCADNYDTWLDGRCFRMNYYSSVGYSTAADQCGKDSARLPAIKSQEEQEQFFAALKQYNNFGAYSFWLALSCNGSKFVWADGSEADRLQLHFHLNRSSLLHRQRSSVAGIRRTELLLWTSPCDSYELLQTGKSTDTCYKLQQQQTTWNQAETYCKGQGAHLSVIHDQTIAFPFSDASDNYTWVDGSDIDYNNFVAGFPSDSYGNCVAMETGFLPGQWMNVDCYNTRLPYMCTKPAFYATNPQPAGCPEKMQYAPGDEIFSPAYPQAPGGTGCDYLLLEPNQNKRAEITIDFFESNTCCDSLTVYDGLFGSNILKTARKLSFEKCRDQHVYKIISTITGMNAFRIAAFLGDDRLLLEPGDYQGQFKCDQTGVECDEWRTCERIPCQV

>Ppa|PPA38315

MVFYLLAVNRMLSSKTKKARFYTYDLIYSSSSVACHEQAFSARSIRSYAWWDSPGTAMPVVSDFAAVSADCPNDFDIVHDGKCAGKVPRGRCNWADPSCMAQSCNSTDLRAFNLKPVIIHSEEEQKYWTEQATSNKNGIFLDLGSCTLPPPEYYFLIWWIEKDTGRWTAERSDGSYDVFCIGQLPPLPSHDFCDDGFVSDKMKDQCYKIGSPATDFTRAQMACDDANADLASIHSTQENSFVRQLAFSSGATNGIFLGGATDENQTEFTWTDGTAWDYDNTYAGYPKEGNGECLLMDTFSSSGKWINADCSSKLPYVCVRPETLPVCAPGPWKEGDKANNYIFILKILVSLQITSPGYPYDASTACDYILSVDTGKYVEVTIEELQANSCCDQLLLTDAKFGGALVAKLSGQIITGQPKTFISASNFMRVSWQPNGGVNVKGFKMSYRAVDTREPTTTTTTTRTPKPATQPEKTTETTSYNYSDYSETTVKSTSTSETTVKSTSTTVLPTTTSGTSFVGSALMVMPLLTICLF

>Ppa|PPA38318

MRAFLLLFSAIGSTYAIVHFSNSTLIGYYDALHYSVALDQCNYGCLIYAAGSSGFHNEYPNNTDPYLSNVFVHNTRINSNIISIAKLASQVDSARRKVPLTIQNKNEKLTIVDLNGPHDSSPGFVIYVVDLATANAIADFEVYDVADVRDAELKPQSEIVTFISADPFTLSAPASEQTNQVVVRLGGLDNALANHRDDCPIALQISDDSAFPGFSIQPNTPIISMVVTKKNRLILQPSANFMKDRDLSSDGFITSPGWNGCRNPNSGGIQSFRSPKYMPSDSYRLTAKLVEHAKFSTRRVSNVYSHPFLFFFMFVHIQLRAVHSHQCGPNLDQYHPLMIQEKGDQRIDVTGTEVVAFDFDVSDDKFVDVWYENMVGDQGFLLTHTSKSTISASTALPPASTVITADSYCNCAITNGWFDHWDPSEIWVDVVFILDTSMDDKLEEAKTLVLSTVSLLSTNTSAEFYSRVGVIAASDIFEVIYALDMNATDNLDSVKQHNIDSIDIEAAFTAATQLFGTGSDQPSYRPKAKQIVLFLTHSSPTAANCAMGGVMYVCESAPCSCSGYCGPMWRMTQGTGHWRACEAFKISTGGDFSFVTFCSGQLPPLPAYDGCGDAFAWNKETEKCYKIGTPSTDFTRAHCTDANAELASIHSSQENTFVRQLAFSSGATNGVFLGGSTDENQTEFAWADGSIWDYDNTYPGYPKAGNGGCLVMDSFSSSGRWINSECSSKLPRKSASRVLIWTMESRRHHELIITSAGYPYNASIACDYILSVDAGKYVEVTIEDLQANSCCDNLLLTDAKIGGALVAKLSGQIPPSQPKTFVSASNFMRVSWQPNGGVNVKGFRMSYRAVDTRDTPTTSTTSTTANPTTEKITTDTTTITTTMSSTSTVKPTSTVVSPTTRGTSFIGTSAIIVPTLLIICLF

>Ppa|PPA38321

MQFPIVTFLPSSLIISTMWRLLLFSALFVTVSSDCPDDFDIVHDGKCAGMVPKRSCNWADPLCMAGACNSTDLRAFNLKPVIIHSVEEQTYWTEQAISNKKGIFLGLVCNNLTNLWGWSDGSFIDYKPYNNFDKDLGSCTLPPARNCAGYCGPIWWIEKDTGRWTAAAVSADCPNDFDIVHDGKCAGKVPRGRCNWADPSCMAQSCNSTDLRAFNLKPVIIHSEEEQKYWTEQATSNKNGIFLGLICNNLTNLWGWSDGSFIDYKPYNNFDKKDTGRWTAERSDHGSYDVFCIGQLPPLPSHDFCDDGFVSDKMKDQCYKIGSPATDFTRAQMACDDANADLAPIHSTQENSFVRQLAFNSGATNGIFLGGATDENQTEFTWTDGTAWDYDNTYAGYPKEGNGECLLMDTFSSSGKWINADCSSKLPYITSPGYPYDASTACDYILSVDTGKYVEVTIEELQANSCCDQLLLTDAKFGGALVAKLSGQIITGQPKTFISASNFMRVSWQPNGGVNVKRFKMSYRAVDTRELTTTTTTTTTTLKPTAQPEKKTHPTSSSSTAVKSTSTTIEELQANSCCDQLLLTDAKFGGALVAKLSGQIITGQPKTFISASNFMRVSWQPNGGVNVKRFKMSYRAVDARELTTTTTTTTTTLKPTAQPEKKTHPTSSSSTAVKSTSTTVLPTTTSGTSFVGSALMVMPLLTTCLF

>Ppa|PPA38859

MLIIDYISGMKTLVSIALLLSTIQEVSSYVFSCEEIKSKLINSPRIENTLFACVFLEEGLDVNTPYLNNIFIQDQQDKTSYSLSSIAASPSHCIRGSGPWQVVSDKPGDLRCDLEISLLFTSDEKTEYVLAIDDDTVHRTGNGRVTFVSPRSGMKITINNIDADLTVYTGAGKGPSEFMYELKTWSVWDIPRYFASFDNVLTFDTQAKDVIYYVTAEYRNGSFHPSLVSNLGVGEKAVILTSGKSDNLMNKHPDENYVRYNLWEAATANVHGNLAFDPNFGGSVNFTVRGDFMNEEQSFTDATIDWKFYASYFEVKYLTSVKPEDVWDNEDTFVIEIEMSELPTDITPIPGIRTTPQQEEITTQEEGMTTMKPSTRPPSTLAPTIIPVAAVDNYCNCAITDGWFDNDWDPANIWVDVIIILDTSKSMGASLEEAKSVITSFVGIMSTDVTVEFYSRIGVIAVSDTVEVIYNLNMTSSDDLDNIQQHKIDKIDVGAAFQAALKMFADGTKMTSYRENARQIIYYLTNSAPGANMNGVDDFKTGGGIIIVNDYILEGEVADPGLQKLASDNFFFTDLSENYINSLGVFCEANCFCSPDLHPFNDDDNSPRTQANRGCFHPVNNGIPQQKARETCQKEGAALVSIHDAQKEFFVNGVVSIFGPKKKFWLGYQNDGTQWIWDDKSTDPYTDWDKANNQPNTNGGKNMCAYAQQGTGFNTPWTAANCGMGGVVYVCESAPCSAGNKKC

>Ppa|PPA38981

MIYFQGGVVLLLLGSLHSVNSEIEKSPVPLCGIEWSCITTDTCMGGLSSMDLTTFSTQLITGDCKMIMKIRAYSAKKYRVSLESRDPNVNKLVVNKENTVLFSCEEGLQPKYKGAHQKSINHTEKPKLLMCEFIAEEKDEQTIVRSDEQTNIAIFANDVQVAKGKEFDLYSKIKRDSYCNPEKIAYDPAIERLIIGLTDLSCLPPYYIETRLKDNAGKLEPKHIKTTSIDCVKTGDTYEYVIMRENNKMEFTETLGRCAARSCTLCDWTPEVEGIDFTIFEESDGCKKLKCKTGKLNGDSMGVATCKMTGDKSAWDLNGKTIEKDDKVTCDTKLECRSPVPFSCPNDETAILDSKNVAISCDYNTGNWTYYQEGTVVTNPDPAKLSCLSKESKGSNDTGSQTKSASGTTIGVIVGGILILIALIAGIAIYLNRRRNAQNNNIGKTTKSQEPFSIQPTSKSKKSKKEPNICMGVDMSTVPTPSVPPVSPEPYKRPPPPQQVMLNAADIPSNATTGTDGIKRKKVTPDGGTNKGGDTTGAETGPFNIDETAINKKGFRRVCLTIVSIRHLFTCHVDRSMLLLFLLALIGTVNAVCPTGYQQINGGDCFKLYTTTQNYDNAEAECVKDGAHLASVHSLNEQNALMAIMGMTTPLIGMKCMDAVAAHCTWADGSIVDFSKFPGGAPLLAYGNCVHLASTDTNWYSWNCAAVITGFLCRVPLNAPPVAECTNGYVAYNGGCAALKTTSRVQSDAEASCALEGGHLASIHTEADNTFYAQLGTNAGLTNNIYVGLAWNTAANNYKWTDDSTYNFKKFANQFPNTVFGECVYSPNFPVSIPAGQTCEWVLATAVGTKVSMQFPIFNTDAGTTLSLYNGLDDTTPTNRLSGNAINPLTAYESTTNVMKMIFTPSAAPTGTGFQANFIPTGGVIPDPQPTFDPITQCPQQQYTADTYVYSPNWPEPYPPMSDCLYYIHSRDGKKMSIEFGYVDTEQCCDIVVVYDAPDNKDESKIIAYISGQSAVTPKTYYSTGTTLTLQFFSDSNNQGEGWIATVRNL

>Ppa|PPA39269

MLFLLIFLVIDIASSDCPTNFELFGSRCYFVSSENKNYHDASSFCRSLKSHLPSITNDEEQDSLIQIRSSNVFFLGLEFDFNNGYKMCSNMDARTRFKLSDTGLWEIIWDSADIQLVCATDEDGGNIDGDDFCCWWLLYYVLPIGSSIFGCLFLSLCCIALRRRAREQHETMMEYAQKNTQLESEIQRNAEWVNERKEYLEKFNELKKDEWEVERRFVDVDWSVELGNGAFGKVYKGTLPAEKLPSKSLESVIQVSELKKNDEKIAVKMLHESADRSTVLAFLDEIELMKNIGYHERLVNLLACVTESEPRMLIVELCIKGDLLRYMTQRREYMLSHRMDEVTDYNLVITHKHQFIFAVQIASGLLREFLSHRGFVHRDIAARNILVNRNDSVKIGDFGLCRKIVEDDGMYLSRGGRMPIKWMAPEALKDYEASSASDVWSFGVLLFEIVTLGGSPYAGWNIAEILLRLERGERMERPDECTDQMHEIMQSCWKFNPRERPDFTELRMKLGKALEKVTDDNYYLQLDGRKEYYLVSNERKEKMSRFFILLTVSAILFTSAFAAENNSSDPVLTRVRRQCGCFGQVFPPFFEFSLLWFDLFQLLRDDPRFLSIHSLVFSHLNRLIPLLLIFPPPSLSLLLTMSIYLGMTAAAAAGYYAITTKMSQISPLVDPRSQTTVLPDGSRVAANICGSPKAEYLYEDARTVYEAFARGRKMSGDRPMLGMRGGTADAPTDYSWITYNEFWKNGHWLAAGLMSLGLKQSTETIIGIFSKNRPEWIITELGTFANGMVNSPLYETLGLIETIYVCKLTTMPLVIVDSPSKAQLLLKHKTDLPDLKHIVVIDAKDLKSIESIATPLKVSIHSFSSLIEKGKTMKDFKPKFPQPDDLATICFTSGTTSMPKGVMLTHKNIISDTTTLLNVSTMRMIDNDDVMMSFLPLAHMFERVMEITLFSSGGAIGYFRGDIKGLADDIKTLRPTVFPVVPRVLNKIYDKIWAEASKSTVKSMLLSSAVAFKELEMKRFVVRNDSLVDTVVFKKIRDSLGGRVRLMVTGSAPLSTTVLTFMRAALGCVLVEGYGQTECVAGATLTIAGDSNPGHVGIPVPAIQIKLEDIPDMDYKADKNGGEVCIKGATVFTGYYKNAEETGKALDGEGWLHTGDIGRWTTEGTLKIVDRKKHIFKLSQGEYVAPEKVENVYVTSKFVGQVFVHGDSLKTCVIAIVVPDAEVNFTICHSNVRYAQVLLPHAKSIGMKGTLEELCKDAKIKKMIMDDMIAEGKKAKLNSIEQVKDIFLSHELFTIENDLLTPTMKSKRPNIKKRYTKELADLYSRLE

>Ppa|PPA39287

MYLRTWIFIVQSIIVVPPLILYILELKILLITRGNEFSSPFYTLFIACGITDIIGVILCQFFYALPMASDIGDAYISSLPVWFFTAANGLLFYLPIVEDFLNIAVALNRLFSPAIYIKLPVGRDDPPVNLLYPLTLVTFPGLFFFLNTSSPYAGYVVQSLPWILIFKCLLHPILLFLTNSSIRKRITCVNQSLNSSSEIAFVLLILPLLTLSDGICDDFDVYEGLLHTICLKVYRNEKNFYDAHTFCQSQKGNIAPLTISWYISFNNFVRRTSVANNITELIHIGFMHNRTDFYFQDDKYDEVYAWTDGTKRDYEHWNLNGYPSHLIANCTAMQTGTIEGRWINVPCENELPFVCKRKPSNQ

>Ppa|PPA39558

MLRLLSVLAAVLSLAVASKCPPFYDLKDGGKCIRALNLWVKGSLDTLLPQMNDQCGKDKATLPIIKSAQDQKMFNDIVASYPDIKDKSVFLVLGMVCNPSTHRLEWADHSAINYIQPPGNVNLNFDCVADAKHVVSRTWFNDWFVVDDDATLYTYTFLCEASPSDEDIDECGDYDVMSASKDWDKPCIKIFTDALSWRDAEAKCERDGGSLSTINSDEENSYIRRSANGLGVVGGVHIGAHESPVGSGKWSWIDGNGPIDKSVYSNFVNPFPISGRWSCGGMYTDSTAAPWIDVDCDSAKLPFVCRKQRK

>Ppa|PPA39562

MRALLFLPAIVAVTTALTCPSQYQLIYGRCIRLLELSKKFVLDDKLPDYKAQCAKDGAHLPVIKNDQENQNFNDIVLSLSGNLEESYYICMDLVCSKATRKLEWADGSPVSYSRKNSVNFNFDCMSSPKTAISRELFDDWSLVSTTQLENYWTILCDYEEPITTPKPTTTTPKPTTTTPKPTTTTPKPTTTTPKPTTTTPKPTTTTPKPTTTTPKPTTTTPKPTTTTPKLTTTKLTLPPSTTTAPKPTDKPTEQCGHYSTIVNPADNKSCFKVYTDSLSWSEAQNRCSSDFGALVTINSAEENKFFWRSAVTANLLNGIHIGAHQSPDLPSTWTWIDGEVPFGGKSYDNFVGSFPIPGLGACAAMMTDSTYDQWINEDCNYDPLPFICRRIDYTNLPKSCPVDAPAAGQDIFPPGFPSSDMPCSYLLSVEANMLVELEVITLVAEQNMDFLEIHEGPVGSTLLANLTGTILSPVKFTTAKSNVLRVNWKPNGSGEGRGYRVSLLSTVAEMRALLFLAITVATAPTLPCPSQYELIEGRCIRVLELFTSFIIDDKLPGYRSECAKYGGHFPVIKNDKENQDFADILYSLNGNQWENFFLVMDLVCNNTTRRLEWADGSPVTYSRKNSVDFNFDCVTTQQTAISREIGLDDWFLVSTTELYYYYTVLCEHDSAATTTKPTTTTVKTTTPTTTTSKTTPTGQCGDYSVYTDPISWNNASQKCASEFASLITINSADENKYLWRAALSNNILEGMHIGAHESPADSSVWTWSDGEIPFNGKTYDNFMSIFPIPGGGECSSMMTGTSAAYWINEHCDENDQPFMCRRADFSALPKSCPTDVPKAGDDIYSPGIPNSDIPCGYMLFVAANKLVELEVITLVADDNKDFLELLEGTSGPHLLANLTGKILAPTKFTTTKSNVLRMNWAPGGSGSGRGFRCSNAVVMRALLFVALFGINSCLSCPQNYDLVESGECIRKLFYGSDYLDHLLPDSRAKCVKDGARLPIIKNDKENEIFNRLVNAQSIGTYLVLDLTCNYTSRRLEWIDGTVVNYIPWKSNINLNFDCYTSSQTIITRPVVNNRTSWNQAQRKCSSDFASLITINSDEENKFFVRAAVSNNILEGMHIGTHISESTSVSLDSNVWIWTDSEMPLDGNTYNNFISGSEHNNNNKTLDTNLVFPIPGGGECTSMLTGSSEAYWINENCDQNMQAYMCRRADFSTLSKICPTDAPMAGEDFYPPGFPNSNIPCAYMLFVAANKRVELEVITLVADINKDFLEILEGTSGAHLLANLTGTILTPAKFMTVTSNVLRVNWAPGGSGSGRGFRAITSNFPVFEAANQRVEMRVIVLCLAVIFTTVWAGCPAQYQLLENRCIRPLHLWANDTIIDLQPQSQRDCGKDGASLPIIKSDEDNEAFIRITNSFDDIKGWNNYLMLGLVCNGWTQQLQWQDGSAVDYSPGLSLNFDCTKNTAVSRTKFHDWKLVSVEDTWSYTVLNYVTASKRSLFTVEEDTDKIHSAAIIGSISAQSCPPNYKLSEGRCVRALYLEVQQVLNNVLPQARTECAKDGATLPIIRSDEDYTVFNNIVNSFGLKGKNPYLVLDMVCNAATRKLQWVDGSTITYTPKGSSDISFDCVNVHNTTVSMTDANYWTNIQTDSSRMDTILCVTIDQEEQCGGYTLMDDAKIPKPCYKVYSEPLPWNAAQKQCDADYGSLITINNADENKFFWRTAVTNNFIEGMHIGAHQSSADPSVWTWVDGEVPFGGKTYDNFISSFPIPGAGACASMLTESAPALWINEDCANNKQPFICRREEAVVRIFIDVRIDPVSFYLTLYNLHNPSRFSDHQEELSRFFIVNADSSVRQRWRVVIEKSQIISVTEKKDVMNRAKSQPAIADKLSSNQNMRALLLLAALAGSVTSTLVCPPRYFYIEGRCIRPYTLYIADYLELIMKYGQEACAEDGANLPIIRSDEENDWYNRIALTFLQPKGMRIYLVLGIVCDEETQMTRWVDGSPIEYYGPAGSKYPAYYNCSESTTMPTSMVPENHWERLLKSDLFYFTSLCVIEEKAEPTEEVTEEPGNRCGDYERMDDGMDEETPCYKVFTEPLSWENAQRKCASDFGSLVAINTADENAFFWNVAASYNFTGGMHIGAHQSPDDSSKWTWIDGEMPITSKTYNNFIRSFPIAGSGRCASMATESVAAVWVNVDCDGVEQSFICQRGDFSKIPSSCPNAAPKSGVEIYSPSFPKSDISCEYFLTVDADKLVEIEIISLISERNSDYLELFEGSAGKTVVANLTGSLLKPTKFTTSKFNVLRVNWKPSGSADGRGFKIRYTEVGKVDAGGATTLEHFDTTTTMSATSYGIHSLVLTALIWFALSFYCFHNNNSDMSSEILLHRRQIIREYAQAACDEDGAHLPIIKSDEDNDLLNRIALAFIEPPGPENHWELLWNGDFMHFTSLCVIEKNVEEPTEAVATEEPDRNRQGFEYWLNKLRPFPLLQSLLSVRKLSKDEIYRCGDYEMAAEGMDEETPCITFLVFLKRRCQVFTEPQSWVNAQKKCAADFGSLVAINTAEENVFFWNIAAAHNFSGGMHIGAHQSPVDSSKWTWIDGEIPITSKTYNNFIRSFPISGSGNCASMATESIAAVWVNVDCDLVKQPFVCQREDFSKIPSSCPNAAPRAGVEIISIISERNNDYLEVLEGTAGVNIIANLTGTLLKPTKFMTSSSNVLRVNWKPSGSSDEGRGFKIRYTAVAKVDVSGATTTEPFEHFDTTTKTAMSKGIFTLIVAILHSFFASAAAITEQACPPDYLFFEEKCIRPFTIMADDYLVDLLPIVRDKCALDGAHLPMIKSHEENESYNRLTDLLTAPKGKMVRLVLDLVCDDNNELLTWSDGTDVEYKPLGSDNMSYNCSNSPSTVISAPLDHRWERVALNDKWLYTSLCVYDSQEQTTTEQPSTTEETTIDPAFEKCGYYDLMENTANGDQYCLKVHSEPLSWEDAQKKCSEDSGSLLTINSIEENNFFWSTAVSNNIHGGMHIGARQYQTNPSKWMWTNGEMPISCRAYNNFIESFPIPGVGECASMATESSLALWVNQDCNEAKLPFICQKGDFAAARNVCPKQVPSAGEAFYSPGYPDSDISCEYFLFVEANKRVEIEIQSLTADVYNDYLEILEGSSGSNILANLTGTIHAPTRFKTAKSNVLRVNWISNGEGIGRGFKIRYSEVGQEASITTARPITTTSSVASHGICGLIVALLFCLPK

>Ppa|PPA39563

MWRLLFLVTILQLAHCQSTCPKGYDLVRDGECRGHAKTATITLDLGSSIAVDTCAGVQPSSKPVIIHDDEHQDYWKAFAPENTSEGFLILGIVCDKKSGRYMWADGTRIDYKPSLVGWNPGGRGSPVSADFYCTYQIAPSPPGADGCDNFADDEDDGVCYQVSPATENWQDAHMTCKKLGAELASIHNQQENNFIRRLAVSKGAVNGVFLGASVQQDGSFAWIDGSKMDYENYYPGFPKKNFGDCIGMDSSTSAGQWMNVDCNSQLPVACIRQQGSTEGPVCTGDDYAEGTIITSPGFPNTASTECDWFLTVEPDKKVQMEIILLEANSCCDTLILYDGYLGGSVIEKLSGSIQNQTYTTKTSNIMRVSWQPNGGVNVRGLAVNHLSCCVIFIQGTSTLN

>Ppa|PPA39565

MRALLFLPAIVAVTTALTCPSQYQLIYGRCIRLLELSKKFVLDDKLPDYKAQCAKDGAHLPVIKNDQENQNFNDIVLSLSGNLEESYYICMDLVCSKATRKLEWADGSPVSYSRKNSVNFNFDCMSSPKTAISRELFDDWSLVSTTQLENYWTILFDYEEPITTPKPTT

>Ppa|PPA40555
[truncated: 213,321 more chars]
