# Supplementary material for: Parallel Evolution of C-Type Lectin Domain Gene Family Sizes in Insect-Vectored Nematodes
Source: Front Plant Sci. 2022 Apr 25;13:856826. doi: 10.3389/fpls.2022.856826 (PMC9085898; doi:10.3389/fpls.2022.856826)
Supplement: Supplementary File 3 — Amino acid sequences of CTL genes in vectors. [file Table_3.DOCX]

>MaCTL-1

MQFSSTGFVAIVLLATQCPFTNSANKFGNQFKDGFHLDLREIQRQNQITTQILQNYLEKRLFGQNRSSKPPSLWEILPRTRRKPSNNIQEVINGTQGEKETLQSVESERISAYEDYQDDEEFDENEDCPNCVDDSLPSANRWTMPLLKLGEKRYYLGIFFKANYFRATQYCRFHGMHLASITSQEENDKLEKYIKDFGFGNEHFWTSGTDLAEEGNFFWMSTGRPITFTNWNAGEPNNFEYENGEQENCLELWNRDGKGLKWNDSPCSFETFFVCEVQQ

>MaCTL-3

PDAHWVWLSTGKPVLFANWSPNQPDNSGNNESCIEAVYIHTKDALVWNDHVCTLPKHVICETLEVKCVTNUUHSUUCSUKYINTUILGNKNIGKLYILURRRKKNSYIGYPIKNGTQSSYRURTSKUINSATUUNATSYRDTKLUKGASTVUMAALFLYUKYVDVKTPVKEKSINISNLFVVEKNVIUFICTFPDALINUITFFLELTSMHTLEVVUUNNHKIMIUKVUTQFCTFLDQSQENERKSSDKTPCVSIRSEILHILLNCUVSMSYSYSCNNUAUSLMGHSVNLFSKMLFSRISRQRVDIRLLLWLRNIUKILFIUSHNRSPIECAFLYIAIVLDFIFLEKVLSQKTRMRKSUSEDAFFKGEFCISYLSHVHSSIILIVINNSNNNQFRIEPTNYIQIYCGHNIUULFSHSTQISFQSSGTCAFQLKQWIFNLMEALUICCACVGUFETVSNCCANRAIAIWPLLAIIQKUKRNSEKVQQPLGTKNVEDVSYFDLISWDLRCALTGKUEFELSFGKCKIYLUVKWCVPATRFUHFLRLRKSATKLMFTFTRRELUFELTFNRLWFSSGIUMQNKVIFKNGYTICUCWKUUTLVDFIWCSMFSYFADKVSUNUUTUPYLLGUDSLWENGKLVYUUNIFLURTPSSGQDVNRLCGISNWLUWKICLUUTRRQVRKYKLHWHEGFLCYSGSYYSSNVLYNCGUNDTKYNISIIFLLVDPFUCGINYLDSVHPFRSYIAVFYGGCNNGGSKSVLETEURIFTCMVVVVIIYGNNVVLLCLRUVCWFICCFFDRTDDRFRFMDYIGRYEUTRERYPEKFISKSPLFNNITCASLHHFFLYPLEGTKUEWUWGWILQFSISVPINRUUSSQCKHASPSSLWFYNNIKKPUDGRRLLTFSLSSLPRRDWSUTTTNYHLHSURUUQUVGNKKIQYRASRRCRDSTUWUPDKIGTYSNKTQPSFAQRTSSHNKETLPSHRVWRKRYGRCERRMADHCHGCKRRNPCDSSUUQIKIRSLLAVVHINYQWQTVTUMGLUTARGDLUSULEGSKWSVECRGKYFUETAQCUFUSVCTQLLGKVRUISRSNVSRKCWVKAKRRRNYISPMAVAHKLQRPIFLWVPIPNIFTRUSCYMVEUFGVYCDIFDCVHVURCQAKAWIYUIIFRFPUEEIGGMCMVILRMVVALCTILGNGUSVIFSSLFSSITFULNDYRYTNULLIGGDSEFVURTNFKTCLPYISWVNFVNYIIQFLFICSAGIWNERSFSKRSKFINEWSKMDGHLGVLIIFLUIFUDIIKKVKFYNURLKYSEHILYIFFIIUSFUYFLLYTVWPIKNUAKHPYFKKFLGCDFNEFIYVYUPSRCUSYYDDPTANPUTTIFFSALNFFENYFFYUKSGNVASLQHHLKFLWLKFHUFISKNNFFIEPUKLKIYUICDSKKKVVTVGNVIUILFLYLFHKLKCURKNNTVAVTQQITLNKVNEKNRNFEIFENKUKPLNNSINEWRWIFKFLFVYFGIFUNSTFHINKNHUNHGUTFFRRVFUTLVGYTICIYLSUKKLLUFYKIHDNKFVITNIMIYSKKMLLYLKQVLSIFNNFIKKYIYFUYKEFRLUILVMPEKLHKLEP

>MaCTL-4

RRQVYKGNDKNHNTQIFYYSFEAMDLLKYLLVICAYLGNIEAAATPPNKTNTGGWQNAFSNDSSLIPLVQFGDKSYYFGVYFTATYLEADQFCRDIHMELVRVKTSEENDQLYKHAQQSTNQDHFWTAGSKLLDKKNWIWMTTGEIINYTNWGSGQPDNPNTELCLELWLFKNKGLYFNDRDCNTKFPFICEREARRQYGSPYRGDSNWPSVFQSPTVSPNINLLNYEGKSYYFGTYFKATYLQAFQFCEIINMKLVSITSDAENNIIHKYLRDIGIVDSFWSSGSKAIDGTIWVWLSYGRKVNYTHWLSGQPDSVNEQCIELVQKKEEGLFWNDLTCTNQLYFICEKNDRIIPRIPLGPGPVIFPSRTTLAVQPTVQYPENPSLPVTTVNGRSYRCSKIKATQANASQICKQYGMELVSIKDKKKNDVLTDLIHQSGKSAAGDYWTSGKKQPDGKWVWGNLQPLTYTNWAKGEPNNEDKQEFCLELYPDGQWNDVACGREFYFICESPGSGNSHQCTSQPVINVYVNNNQCSNAKNGTVTETKKIASSKDGEGYNVEVNNNIGTCPNTVPHK

>MaCTL-5

MLRKITLLLMGAVMVIAQRRLALPDPRSCANRVRHATYRDARGVTHSYFFSWEHAPTRSLEVDWLDARNICRRHCMDAVSLETPQENEFIKQRIARGNVRYIWTSGRKCNFAGCDRPDLQPPNINGWFWSGSGAKIGPTTQRNSGDWSQTGGYGQPQPDNREAAQGNDESCLSILNNFYNDGIKWHDVACHHVKPFVCEDSEELLNFVASRNPGIRL

>MaCTL-6

MWSTKCSSQRPSYSALVIVFLGFINEIVCQDIKCGHPAVPVNARVSLSSPSLAPGTIATYQCDEGYETFGNTQTSCSPSGQWAGELPFCGVNIAFRKPANQSTTVRGGNALNANDGEKSTNHDGKRCSETQKEASPWWQVDLLRPYAVKVVRVTTRGCCGHQPLQDLEIRVGNSSSDLQRNPLCAWFPGTIDEGVTKTFTCARALTGQYVFLQLVGVEGSLSLCEVEVFTTDEFSVDRCAPRNAPEDAQLAAFSKMCYEFGVGRGGSFAEARAYCQSHNGDLVHDMSPGQTSFIYAELERRKPNLKTQLVWIGAQKEPGLTSRTWKWVNGDLVQRPAWGKDQPNNYNGEQNCVVLDGGRGWLWNDVGCNLDYLYWICQHTPSSCGSPDKLINTTIVGDNYDVGSNIEYQCPEGHMLTGDGKRSCGTNGFWTGIAPTCKYVDCGDLPDLEHGTVTLEDRRTTHGAKAVYTCHENYTLIGHEMRMCGDDAKWTNSTPECLFDWCPDPPAIHGGIVSITGHRAGDTATYSCQPGYIIFGQGILSCGLGGKWSGKAPSCKFIDCGAPPNIDNGRYELRNGTTTVESIVEYHCEDDYWLDGQKVQKCTREGKWSGDAPSCELITCEEPEVPPGSFVVGYDFNVHSTIKYDCEVGHILRGEDMHVCERDGEWSGTTPTCEYIDCGKVPTMPYGTVEYVNGTTYLNSIITYSCVKSYRLNGVPKRICLENNQWSDSSPKCEEIRCPEPILADHSILSVTGNDRMYGRTLIRTAESTNVGATSYKIGALVKYRCERGYRVVGEPLSTCEDTGKWSGDVPQCVYVDCGSPEIVENGKVVLPSNATYYGALALYACQPNFDLDGVSRRLCLENGTWSSDTPKCREIQCKEPDAIEGVNYKVSTLSVGGVAQYRCPRGHSMQGNSTRICLKVGSWSGVAPICTPVDCGHPGTIDNGRIIVMNGTTYNNAIEYHCIPNFERIGPYLRKCMENGEWSGEEPRCEMTTGEPQESSNLGSSIGIGAGIVLFLLLLLGATYLKLRKPVAVKNTENVEGAERKEDRNAAVMSYATLSDRNGYAHPNLESTNIYENIHEENLYDAPYEETSRDSGTYEPEPMERIYGNAITINGVPVR

>MaCTL-12

MWLVTVQVSCIIGITLAQVEPYNVNNFNTNSNPNYNVNNQLYNYNQNQYQGYQGGLNYGNVEELRCPEHWLQFQHSCYRFIKSPLKDYSGARRLCQTYSPDQDGSDLVSITTPEEHGFLIHQLNQFDRQHYRWYIGTHQQSANSYTNLDGTQLVTAEHAILEVNLPYGKEYLAYNFSKSLMHWSFEPVKGDELLLFICEAKIMAVQRLVTENRTYTYGIDVNDPERIPRGPYFINQPVDATFDTSKRKLYNDISVNCLAGGYPTPTYKWFREDYENDRLVAREIDPLKDGRYTVSGGMLIIHNPQQKLDYATYHCKATNKYGTIISETVQLNFGFILEFVLKRSPETGDQNWGKSIFCDPPHHFPSVKYSWSRDYFPNFVEEDKRVFVSNDGALYFSALEGIDRANYSCSVSSEFSDSGRNGPFFPLRVNPHSNYQQLKFPNNFPKAFPEAPIAGKDVRLECVAFGYPVPSYNWTRKGGSLPRSVYFTSYNRVLNIPRVQVEDEGEYVCRAFNDRASIENSVILNIQAEPNFTIPLTDKHLDSKGELVWTCEAFGIPDVNYTWWKNGRQLVMGYMEPEDQGRIKIQDNVLTISRLNDERDPGMYQCRAENSLKIRYSSAQLRVLAFKPSFKKHPLESETYAAEGGNVTIKCNPEAAPRPKFVWKKDGNVIGAGGHRRIYDSGNLFISPVSRDDEGLYTCTATNDLGLDESKGRLLVLRGPRLVEPLRSNIFTTVGNDINLHCYAEADEMLDIAYIWKHNGLTIRDVDVKNSYNRLKIDGGYLKIINSTFYDAGEYACVVKSAVGRIVSKSNVVIQGPPGRPGGLQVVTIQKKAVTLEWTDGAHHGSPIHSYVVSGRTNWNSTWVNISGNFRVTEVNRYTGRKEAVIENTLIPWSVYEFRVAAWNDLGMGPPSAPSPRHATPWDRPFIPPRNIGGGGGKIGDLTITWEPLRPEEQHAPGIHYKIFWKRKFGETEFQSQNLKHYGNTGMAVVHIPLEFYYTEYIVKVQAINDMGPGPISHEVVIFSAEDKPQVAPQLVYALSYNSTSLNVSWSPVQETREIIRGKLIGHRIKYWKKDSNEQDAVYYLSRSTKPWALIVGLQPDTYYVVKALVYNGAGEGPESQFFIERTYKKAPQKPPSSVQLFGINPSTVKVTWRYVQPSQEEEPLQGFKIRVWEVDQDMSTANDTIIPYGDKLEATISNLSPGKTYNMRVLAFSKGGDGRMSSPTKMFRMGDPEYYRSASENVQTSMFVVAIALTVNLLFRYF

>MaCTL-13

MKFACFILAACACACVVADPAINQIRPVSNRPGRFLSLPNPTKCANRPKQFFYRGHNYFFSEHVPELAGRKVDWLDGRNICREYCMDLVSMETQEENNMIFKLIQQNDVPYIWTSGRLCDFKGCENRADLEPKNVFGWFWSANREKIQPTNRTPAGWGYNPWSQTGHKKQRQPDNAEFDINGTSESCLSILNNVYNDGIAWHDVACYHEKPIVCEDSDELLNYVSSTNRGIRL

>MaCTL-16

MKTFLVLSVALFGAAVAQFPNGRILEPPVPALCAQRVIHDRTPDGKGYFFSWRDPATKGLEYDWLDGRNFCRKRCMDLISLETSAENEWVKKRIVDDKVKYIWTSGRLCDFKGCDRADLQPVQVNGWFWTAVLQKLAPTTQRDQNDWSPTGGIGKPQPDNREAQQGGATENCLAVLNQFYNDGVNWHDVACHHVKPWVCEENEDLLKYVRYTNPTLAI

>Asi|KFB34918.1

MALYLSRLILFCVTLPILSSYGQQSSNVLRGNSPFYEEADDYLKQQRSSSGIDDIFLRTGGDRKSIDDYFCPEYWTAFRSTCLRVHKSPRKSWFNAQKICQAYQGDLVSVDTIEKHSFVVKLLDQDVSKQNRYYISARQVSPGNWVNADKSQLIAIEDAINYEESIEASDEFQSYFEGSKNVIEKDDQDDPRRFYQSDRYRNRNYLLLGFNGNKEKWQFHPVSGEDQFLFICESRNLYSADNMKTLLEDQRQYDYGLEQTDLEKIPRGPYFIRQPVDTTYDTGKFKITKDVTMSCLAGGYPTPKYTWYKEEYVKDNLTVIPIDPLKNARHTVSGGNLIIHNPSQNLDQGTYHCTAQNIYGKIISESVQLNFGYILEFNLQRASEKGEENWGKALVCEEPQHYPDVKYYWSRNYFPNFVFEDQRVFVSHDGSLYFSSLEVMDRANYSCTVMSTVSDTGRNGPFFELWVSPSPHYQDLIFANSFPKAFPKVPLAGKDVRLECMTYGYPVASYNWTRRNGHLPRLSRLENFNRVLLIQNATVNDNGEYLCTAKNGKKSIMQSIFLNVQMEPNFTIPLRDRTKDFQSTVSFLCEAFAMPDVNYTWYKNGELMAEDGQDARFNKDKYTIQDNLLKINYLDPEEDNGMYQCKATNQLKGVYSAAQLRVLSMKPSFKKRPLESEIYSIANGNTTIHCEPEAAPTPKIVWKKDGNVIGAGGHRKIHPGTGTLFISPTSRDDEGTYTCVATNTQGMAESKARLIVLQELRFTEQLPSKLIKQIGELLFLRCEVTYDQLLDVAFIWTHNGQLLNDYGHEFEQVASGSVEGNRIRIHYNTLEVHNITLVDAGEYECIAKSSVNRIVSRSTVLIQGPPGAPGAVKVLDIKRTEALLEWTNGNDNGRPILYYNILGRTTWNRTWVNVTTNVVAQEADRYNGRRQATVTNLTPWCSYEFAVVAVNDLGIGTPSLPSPVYSTQKDRPYIAPRNVGGGGGKIGDLTITWDPLRPDEQNSIDVHYKVFYRLYGQREWASEELKRQGNTGKAVIHVSVDKYYTRYEVKVQAINDLGEGPISDPVEIFSAEDMPQVAPQQTIARSYNSTALNVTWQAVSQNRETIRGKLIGHRLKYWKKEHNEEDSVYYLSRTTRPWALIVGLEPDTYYYVKVMAYNAAGEGPESERYLERTYRKAPQKPPSSVDIFGINPSTIRVTWRYIAPSQDEEPVQGYKIRIWEKDQDMSSANDTVVPIGKKLEKDIDNLTPGKSYNLRVLAFSNGGDGRMSSPPIQFQMGITQSPLNNSNKMTIPIVLLVVLSVLCYVSTSQAYTL

>Asi|KFB35077.1

MKLFIVLAGGLAVLLHTLTFIACNVPRTAQDMLTQNLCICPCGNPKGGKLFQVSFDVVENWYDAVAHCNALGMSIAAIEDASQLVQLQQYLYRHRYNRNVDYWIGANNLVSGRKLRWGFTEREVHNANWDGAREPRFGPSRAFCATINGFSMLWSGSDCEAKSRQVICEY

>Asi|KFB35078.1

MSNKCLCPCKPFAVKEYYIPISRTNDWFGAVSYCHSSKMEMAEVLNEAEAELLRETIAEEDSDPDSEFYWIGANDLVRPGRYDWSLTTRPVTYTNWASGEPNNARIEGDDGPMERCVAIEKGTLEWNDFLCKQEKRFVCQRFRND

>Asi|KFB35079.1

MPLLKLGEKRYYLSIFFKANWFKALQYCRFHGMQLASIQTQEENDRLEKYVKDYGLATEHFWTSGTDLAEEGSFFWVSNGRPLSFTNWNAGEPNNFRYENGEEEHCLELWNRDGKGLKWNDTPCSFETYFICEV

>Asi|KFB38258.1

MKLATITSVADSQLIEAAIQRSSNTKGPWWIGGTDLGLEGTFVWISTNNLVGSPGGYFNFSPTQPDNKGGNEHCLEIGRWGGVAWNDVPCELSQRFICEFQS

>Asi|KFB38259.1

MLLLSISLFFTIILDHPPVYVHAGQCGIRQIKTRHLLTNGYDTQAGDYPWHTAVYQIIPTEQYICGGTLITSRAVFTSAHCAARPGLNTPRPSDELLVKLGKYTLDEDSPHVQPHSIDRIVLHDEFKMEEFRNDVALLVTKEKVTFGTYVQPVCLPANPSLRASLIGTVVGWGYTEGKKVANVLRAASAPIVSHRVCLESNLEAFGRTVDETVFCAGWRNGTNPCNGDSGGGLFIRSATSGTWTLLGVVAFAASDREDENFCSTSDYTVYVDVSKYVEWIRAKLKAHDRPPTGCNTNEGQKRYVVHNNRNVTFLEAWRLCQTLGFRLATITSQDDSQAIAEAIVSSSNTKGPWWIGGTDLGSEGLFVWISTNQVVGFRSGYLNYSPGQPDNAGGNENCLEIGRWGGVVWNDVPCDWKQRYICEHVV

>Asi|KFB39058.1

MRPTFVIMVPLLLIPCLSAVPICEQQAKRIVHRFYPLRHCQRSNRTVIGLANVKTVRECANLARHKQALAFNYAPEGRNRSNLFVVALEREQNSSNRPSPWKPTQLPSSDGVEEGFEDFYNCHVLDCPEYRNLSTIVNDTRFDYYSLYARNLQTKLNYSNAYNSCVSLGGSLAHIVSDTRTFYLAKYISTLPHANQSISNESSTDTSGSFYFVGLNETARDRFFTSSDERLDCFTFRAWAPGHPERNRHPGCAALTDEGSWKVFSCNRSLPYICELHTSGPALSEPKLKRKCSIKRPNNRFAPTKHITN

>Asi|KFB39648.1

MYVGHRLVAAPGTSSVGRPSAGGCQFPGAPAHGSVIFSDDALSNNTVCTYYCERGFELLGPSRRVCMDGQWIPEGIPFCVLNVAAGKAPMQISTEGSGIPQKAIDGSTSAFFSPDTCSLTKPERVPWWYVNLLEPYMVQLVRLDFGKSCCGNGKPATIVVRVGNNRPDLGTNPVCNRFTGTLEEGQPLFLPCNPPMPGAFVSVHLETSAPSQLSICEAFVYTDQALPIERCPAFRDQPPGASASYNGKCYIFYSRQPATLPNALAFCRARGGTLINESNPALQGFISWELWRRHRSDTSSQYWMGAVRDAQDRNTWKWIGGEEVSVSFWNLPGGDEDCARYDGSKGWLWSDTNCNTPLNFICQHQPKACGRPEQPPNSTMVAPKGFDVGAVVVYSCDEGHLLVGPQQRTCLETGFYNEFPPVCKYIECGLPASIPHGYYDLINGTVGYLSTVMYRCAEGYEMVGRAVLTCDIDERWNGPPPRCELIECDPLPTLFANGVIVAPNQTVYGSRAEVLCNRGFVPDGEPELLCTATGQWSHTLPKCIPDPTAAGPVTVRSTLAPASTTLIPQTTSAASRRPAPTAGRRPAPVVTATTATTTRPPTTSRPILSFSTTGSSQAQVPSESPISSIEIDDEVRPGSVREEAARPPYRPLQPSVVVLPNGASDGQRPKLPPASLPTSTSPVSVTPPSPLPPQTYRPTAGRRPTVTTGTSTTTTTALPPPAPSTTRGRTPPKPHDPLDIHPQDNEIAGSVNIQYDQAPKVNVPFAVDPSEGAAGESKNAKLNLGAIVALGAFGGFVFLAAVITTIVIVVRRNRSTNQHYRHRASPDCNTVASFSSSSSESRNGLNRYYRQAWENLHESASKSHSHSGHSGLKRKETMDAPSVNRSRSRENLDQAGGRPRDLDRSRENLSSARSRDYGRDSMALRDGSEMVVSDVCVKGEKKRHHHHHHKSSHRNDFREPNILGSNGNGRREHRHY

>Asi|KFB41552.1

MRARQVYVLPIVGMLAVCSLAASVPSSSSDEKASIRSEAAESDQRAFPVNHLPPLTYSSKKYTAYLEVVNFFQAWQLCRDKGKRLAAIESYDDHKAVREAILPYAGFEAAFWTAGTNLGAKPAETGTYYWITNDRPVGYLSGFENWLTGVTVTDSDQCIALFLGSALWIAGTCDTNAFYVCEESQDV

>Asi|KFB41553.1

MFVKNGSVPAVVLMLACIGLALALNVVREEQAVPVKETVSIGKLCLAKDVAEPHARFLEPANYGVRKKKFTIGTGGVGTFFRAWRNCIDEDKSLATTDSEEEQHAIERLVARQDTEYWIAATNLGSGTPELTWITTDLVVKTTPRNFNQVPDTCVSVDPDGIWKNNDCFNISIVLPYICEEYF

>Asi|KFB42151.1

MPVAPCQRITTIQLDGVQYFISRMNPYSPELNYFLAYQYCRSLGLQLASFETKEKVESMTEYLQNAGYGKYNFWTSGNRLGTGMFLWMSTGLPFNATFDYFEKSPETVGMDPLDHNSNTSPQRTARDSSSGLQKGCVHLKAPSLRWAPEDCSAVKDFICEQTRCYYYNYGSIPVSSAQGRPIQTSTSTSLYTTLAATTTTTTTSTTTEAATTAAEARQFTSSTGSTTAPTLSSEPTSPLASLLNGDILEQDRTRRPIVQQQQHDNTAEEEHDEEQDQAEHDDDDEQLHDELEEDDEENVVGRSSGNASGIDAIEDEHEEHEHHDEEEDHEEQDETDDEEEQAADLHHQQALAAGQDTDAVSSGLPEDIPVEQKLKQITKEIEQLSGGAGADRELRVDSRQSFLSLSDLIKNIRPAEKVVPQIDSSYANTMRVLGEPLSHAQR

>Asi|KFB42458.1

MLHGREDSVQLDVPSLGSLKDANSGCAQGRIIGYFVISAQNETVTSSDQLMRCDSRVEAKRYIVFNDVTRTFFEAWRFCASMGLRLATVSSLEESRLLEQAVDGSTGVTKGYTWWIGGTDLGREGTFVWISTNIPVGYKTGYSNFSPGQPDNTKKNEHCLEIGRFGKVLWNDMPCETKLRCICEDAN

>Asi|KFB42558.1

MTSYVVYELIYTMVLLEECSLFLKHGQGTIASHQLPSSFQDCSVTFPSQRTDEPEELPGLLVQLTRLNTPCRAGGFLRFIPDPSQRGVNQRTTPSNNAHQIISLCGKLEELPASERSFHFQPHRNTTLHLHNQPLFSLQYRLVDFCYNMTLQDQNGTVQLGPSRSSLDCYFKIHLRTNDWQLQEAAHNGSGAGARYEPIELNALETVLAGQQLTEPVRCSSAGGMLIELYEDSLRRWTSCINGSTTPARYSLLSSSNNLVIKVTKLPLLGMTPWDTPADINRGGRWRADPASSSSSSSPTSTTPSVEQLGSGPSSPSLLFEYRAHPIEPITSRCAFGWIATAQFCISSIERRLPWQEAELECNQLGGHLVSIRSSEDQQLIDQLLFNSPGYKDDNAYWIGASDLVVEGDFRWNDKFSFSYTNWFQGWVHQEHYNRQPNDDGLSGQDCVEIRRHFQIASSSGQTTPTMSPLTMSYMWNDRNCDARNYFICERMMDEELPERLWNEAECNVTVTLSSERSKTTIWSPGFPQMYPDSVDCYTLILAPPGYRVVLDFEEMVLENEPLCYYDYLQLVEPDGDGPYVSPKVSSSNTVQFRFRNSRHSGRNSVQHRRKSSKQGGTTALPTTGSGSNGDGASHPDRRGDINFNVPSIVLQPNDSRYTSSLPLPELSLGSVPRKICGDWSTKLKLLRYVTNGPTLGLRFVSDYSNSYGGYKAKISMENVTSECHDERFKAYNNSCYLIISYPDVDWTTAQQICRGIGAQLASISTTDEQRFITSNIRNSIDYTPRSLYWVGGEITTNGNLEWVDGVKLLFEVCRRWLYESMGWLPGQRPEPSDTLKLPSCLGLQWKVSPTPMISSGLHWTAQKCSMIGGYVCKKPRPKLDETMVKNQTLTGTEGHILSPGYPNPYPAQTDFWIRIVAPEHNRIIIQFQKLDLEHQDECLYDYISIQNYPIVPSSPAAFFGASESSGMALVAEVYHGAPNGSSGATPVNKKQTNFEAFPSTSNARPTEAPITTGRQIRGPRRRIRYVSSSGAEYGLSDQDGATREKANGMAPTLLASSNVNHNELHPSFLPYVRWCGSHDVNMARFNFISTGNEAIVRFHSDFSISGAGFSATWSTVDISGCPLQTITSKEGSIRSPNYPYFLLNNLDCTYVIQAPYGRRVWLEFLVTDLAYEATLQVDIADGPFEPFADESHVNDGVFVSNGERLVVRLKTGAMPRGKGFQAVFKTLAHVNEQRYLMLSNKTTGMLYSLNYPQTMPYGVDYTQHLIAPLGEVILLELYGVGFSQHGCHQTGFIEIYDNYTDSNGTLWHLCEHHVGGKPGSSPDDLEDAGRAENADGHYHPLGHGAAALRDATGSSHPALTKPAPIYITSYLNTIHIRQRNINGTGIRLNATVRLQEDAGYKMKLISNADEWVESCKPNPCQYGGKCITSHKRGLCQCYGHFTGRFCGLNICELEPCFFGKCELTPNSFKCNCQPGFVGQRCDQRQKPCAENPCESRGECFEKNGGFFCRCHAWWEGPRCEKRMMHIPYKPLSERMLQEPFWLGLITVFVVLAVIGLVWCAKRHFPEKIEKLLADETQINRSTFPPHHLNTALREQLQASAGTVPSSNATTPASHRTIFGRLGTPSPRKKRNNSTPTKKNVNEKKQILQQLVSPAQNAATKVISLGELIQLSETRLKAQSYADAEAAGLKETTFSENSLSVTSMVRQISDPKLEKKVTFARLLSKVSAEMSSGSEDLANGSDSLSSSELALHDFGLRSNARRQRPKVSSADSILAMFKNFAASSSALNTLPSSIVISPSSTPTASSPQDDVPGDDDSSTSSNQNTPVSYSSGGGASDSPVFYRQSTIEVPVLDALSAHKSTPTAMAGGGGGSGGGGGGSSGSGSSSGGSGQLHPPTILLEIPSNGINNKCLSPIREMPTPIPSPALTPIMPRPQRSIRSPQMLHDESMSVTFNASFDEYHKPHQLSIEIRPPSPPEDAGRCDYSSSSQSDTTVDGAPTGPTVSIDIHPPTPEHRSPERPRDLIIPELIIQQPSPTRERTMVVIFPPGSPPPQRANQHPFDTGLPSPYTTTDKQQYHQKRFLKQWEKPTSLDLPFDPPMITITSNSNEVVSDAEAPSPAHPMGHPKQAGLGPPGSGGAAGMCYLSPFSMCIRGDRAPSESNLSSSGYSSMASPGPSRCGSSNPLFPHESDEPGSGPAGGPGYSGFHSLINNRRQSSSNVRKKSADSGATGGGGGGGTTVGPATGFSHHHHSFRLRSDSETLSDEPLLESNDEGIGTDHLDEKIEEGEIRSAKELELYLGKELIQSGQDILSQENLSMSQLQLPSIVIQSDAGCDKLSPVSSRSDSPLSERNASLERFSTMFYGKKDQHLPFTDSDGLYDFPSSDGKGAGGVVGTSSHRKSAGRRKERRVARSTVSLQSPSKATSPLLELPGSAKFPSTAPTARKSPKRRVHRQPLASSSSSTESLTSMRENATRTIKGIHHYSGRDPCGVAHLSASKVSADVCDYGDDTGEVGRAMSNAKSKQGREP

>Asi|KFB45679.1

MSVLRTLTLLAIGATVALAQRRLALPDPRSCANRVRHATYRDARGVAHSYFFSWEHTPTRSLEVDWLDARNICRRHCMDAVSMETPQENEFIKQRIARGNVRYIWTSGRKCNFAGCDRPDLQPPNENGWFWSGSGVKIGPTTQRNTGDWSYTGGYGQPQPDNREAAQGNDESCLSILNNFYNDGLKWHDVACHHLKPFVCEDSDELLNFVRSRNPGIRL

>Asi|KFB45856.1

MQATVVAGPKYIPTSSFYINKRIGEALELDGAAPQHRPHRVAPPVGKVIASTGAGGNLFGDAGQGHAGATFTPMRQTFGGASPTELLASPREVSETDLYLLGAIEKLVYRVDYMENRLRRAEQIIYFLMAGNSQKQEPCPQNFTQVHDRCYHFDIERGLNWKSASTMCKSYGAHLAEFETIAEFQDVVAYILNNPVNRGKDFWLGGLNPGLLWIWANSAKPVNPNTNLSSITGTTKKSQPLTTPATPVDTTNGGGDDGDGAAKKQGTKIVNNASKHPTLEITGNGRCLRLSYNSALYTYGYRGEDCSAQFNYVCELKDKTLDNEISRIAKELKLNDIPSVNPSVN

>Asi|KFB51643.1

MFLVSIRNGEEREAVVRYLASINYIQSNKEFKLWISANDLAEEGVFHWGSTGEQLNYKNWRDGEPNEYVHDRCTCEDCVILEYIDGAGLNYNYTFDDRPCTRQFPFICETMLE

>Asi|KFB51644.1

MTKLHVFVVICVAVALQGQVSFQQGDNTFAWSRQKEYYFSSSFKLNWFKAVEYCRDRGMFLLSVRNAEEREAVIDYLASTGYTKTHKGLMAWISANDLGEEGEFYWASTGGRVNYPNWSDTEPNDYKTDECTGEDCAILEYWAEGGANYNYTFNDRACTKEFLFICETLPQ

>Asi|KFB51645.1

MTKLHVFVVTCVAVALQAQLSLQYGDNSFAWSRQKEYYFGSRFKLNWFKAVEYCRDRGMFLLSVRNAEEREAIINYLASIGYTKRHKGLWAWMSANDLGEEGEFHWASTGGPVDYQNWSETEPNNYKTNDCRGEDCAILEYWEEGGANFNFTFNDRDCYTKHLNWHKAVEYCRTRGMFLVSINNAEELDGVVDYIEKSGFSKTHGLLHMWTSANDLGEEGQFFLASTGQRLTFDRWTKNEPNNAKHDNCTFEHCVVLEYYLPLGINYTFDDRPCNAENFFMCETIYD

>Asi|KFB52201.1

MHSVCGPPAIPANAKVHTEKAEGASGGLKSARYDCDSGYELFGPETIRCDPVKGWDRELPFCGTNVAYRKPVNQSSATRSGPAGFANDGKPGNQNPDGQECSETQKEVSPWWRVDLLTPEAVRVVRLTTRGCCGHQPLQDLEIRVGNSSTDLQRNPLCAWYPGTVDEGTTKSFTCARPLIGQYVTVQLVGVESSLSLCEVEVFSNDEFSSDRCASPNLSVDTVLTTFAKTCYEFHITRGESFEKARAVCQSHGGDLIHDFRGITTDYIISELERRKSDLRTQLVWIGAQKEPGITSRTWKWVNGDTVIKPTWGKDQPNNYNGEQNCVVLDGGRSWLWNDVGCNLDYLNYICQHSPLACGSPDALVNTTVVGRNYSVGASITYRCPVGHSLIGTEVRTCQQNGVWSGGPPTCKYVDCGALPDIEHGGIILSEQRTSFGVQASYTCHENYTLIGNENRTCEATGWSGTQPKCMVDWCPEPPPIQGGAIKVSGRRAGSTALYTCDYGFVLIGEPVLSCGLGGNWTGKIPVCRYVDCGMPARPDRGNILLLNDSTTVGSVVRYFCDDDYWLVGPQELFCTKDGKWSGNAPACELITCETPHVPPGSYVIGYDYNIHSSIQYHCDPGHILRGEDTLTCLESGQWSGDAPDCVYVDCGPLTPIPFGSHRYLQNTTYLDSEVVYSCANSHRLSGVSRRICLDTGLWSETAPRCEEIRCTEPTLTPHSFVSVTGNDRMYGRTLIRTSDATASGAQTFKVGALAKYRCERGYKIVGEALITCEENGQWSGEIPECVYVNCETPAGIANGKVTLATNATYYGAAAMYECDGNYKLDGVSRRICLEDGTWGHEQPQCVEITCDELSFADAALLVNVGTRKVGVLAEFSCSKGRYMVGNGTRTCLPNGQWSGRNPVCKLIDCGRPADIENGRVIVVNESTVYGGSAEYHCVPHYNRIGPYLRKCMDDGKWSGEEPRCELIVNDAQETNSLGTGIAIGAAIIVILLILIGVLFLHRNKARPVKNTENVQAAEHKEDQNAAVMSYSSLENGRHNFDLTNRGGLVTFNTFHQSAGPHPPPPSQLTHGSSNNNNHLSSSINNNNVNSNNGSLRGGENIYDQIPSEQFYDAPYEMRTNEEVYEPEPTSRGNIITINGVSVR
