# Supplementary material for: Parallel Evolution of C-Type Lectin Domain Gene Family Sizes in Insect-Vectored Nematodes
Source: Front Plant Sci. 2022 Apr 25;13:856826. doi: 10.3389/fpls.2022.856826 (PMC9085898; doi:10.3389/fpls.2022.856826)
Supplement: Supplementary File 4 — Transcriptomic datasets used in comparative analyses. [file Table_4.DOCX]

Supporting file 4. Transcriptome datasets used in comparative analyses.

| **Species** | **RNA-Seq samples** | **SRA accession numbers** |
| --- | --- | --- |
| *Brugia malayi* | L3 | SRR3111547 |
| *Brugia malayi* | L3 | SRR3111569 |
| *Brugia malayi* | L4 | SRR3111628 |
| *Brugia malayi* | L4 | SRR3111637 |
| *Brugia malayi* | Male | SRR3111501 |
| *Brugia malayi* | Male | SRR3111497 |
| *Brugia malayi* | Famale | SRR3111490 |
| *Brugia malayi* | Famale | SRR3110748 |
| *Brugia malayi* | MF (Immature MF) | SRR5099146 |
| *Brugia malayi* | MF (Immature MF) | SRR5099147 |
| *Brugia malayi* | MF (Mature MF) | SRR3111644 |
| *Brugia malayi* | MF (Mature MF) | SRR3111645 |
| *Brugia malayi* | Embryos | SRR3111507 |
| *Brugia malayi* | Embryos | SRR3111512 |
| *Brugia malayi* | MF/L1 | SRR3111646 |
| *Brugia malayi* | MF/L1 | SRR3111656 |
| *Brugia malayi* | L2 | SRR3111730 |
| *Brugia malayi* | L2 | SRR3111732 |
| *Brugia malayi* | L3 | SRR3111735 |
| *Bursaphelenchus xylophilus* | aseptic nematodes | SRR11474054 |
| *Bursaphelenchus xylophilus* | aseptic nematodes | SRR11474053 |
| *Bursaphelenchus xylophilus* | aseptic nematodes | SRR11474052 |
| *Bursaphelenchus xylophilus* | *Stenotrophomonas maltophilia* | SRR11474051 |
| *Bursaphelenchus xylophilus* | *Stenotrophomonas maltophilia* | SRR11474050 |
| *Bursaphelenchus xylophilus* | *Stenotrophomonas maltophilia* | SRR11474049 |
| *Caenorhabditis elegans* | embryo | SRX1020634 |
| *Caenorhabditis elegans* | embryo | SRX1020635 |
| *Caenorhabditis elegans* | embryo | SRX1020636 |
| *Caenorhabditis elegans* | L1 | SRX145443 |
| *Caenorhabditis elegans* | L1 | SRX145446 |
| *Caenorhabditis elegans* | L1 | SRX004867 |
| *Caenorhabditis elegans* | L2 | SRX145661 |
| *Caenorhabditis elegans* | L2 | SRX151607 |
| *Caenorhabditis elegans* | L2 | SRX190370 |
| *Caenorhabditis elegans* | L3 | SRX001875 |
| *Caenorhabditis elegans* | L3 | SRX036881 |
| *Caenorhabditis elegans* | L4 | SRX001874 |
| *Caenorhabditis elegans* | L4 | SRX100631 |
| *Caenorhabditis elegans* | L4 | SRX100633 |
| *Caenorhabditis elegans* | dauer | SRX103983 |
| *Caenorhabditis elegans* | dauer | SRX103984 |
| *Caenorhabditis elegans* | dauer | SRX103985 |
| **Species** | **RNA-Seq samples** | **SRA accession numbers** |
| *Caenorhabditis elegans* | adult | SRX036967 |
| *Caenorhabditis elegans* | adult | SRX036969 |
| *Caenorhabditis elegans* | adult | SRX036970 |
| *Monochamus alternatus* | larva | SRX5385911 |
| *Monochamus alternatus* | larva | SRX5385912 |
| *Monochamus alternatus* | larva | SRX5385913 |
| *Monochamus alternatus* | Adult-Ep | SRX2563813 |
| *Monochamus alternatus* | Adult-Fb | SRX2563812 |
| *Monochamus alternatus* | Adult-Tr | SRX2563811 |
| *Monochamus alternatus* | Adult-Bx-Ep | SRX2563827 |
| *Monochamus alternatus* | Adult-Bx-Fb | SRX2563819 |
| *Monochamus alternatus* | Adult-Bx-Tr | SRX2563818 |
