# Supplementary material for: Parallel Evolution of C-Type Lectin Domain Gene Family Sizes in Insect-Vectored Nematodes
Source: Front Plant Sci. 2022 Apr 25;13:856826. doi: 10.3389/fpls.2022.856826 (PMC9085898; doi:10.3389/fpls.2022.856826)
Supplement: Supplementary Table 1 — Primers used in quantitative real-time PCR analysis. [file Table_5.DOCX]

Table S1 Primers used in quantitative real-time PCR analysis.

| **Primer name** | **Primer sequences** |
| --- | --- |
| Bxy\|Bu_1185F | CAGTGCCTCACAATGACA |
| Bxy\|Bu_1185R | GTGCTCCTTGACGAACAT |
| Bxy\|Bu_842F | CCAGAGGTACACAGTGATAC |
| Bxy\|Bu_842R | TTCGGTCGTAGTTCCAATAG |
| Bxy\|Bu_11124F | AGCTGCCTTCATTACTCAC |
| Bxy\|Bu_11124R | CCAATCCATTCCTCCACAA |
| Bxy\|Bu_18638F | GCAGCCATACGAACAGAA |
| Bxy\|Bu_18638R | GTGAAGAAGATGACCTCCTT |
| Bxy\|Bu_2162F | TACTTGGTCGGACGCTAA |
| Bxy\|Bu_2162R | TTGGAAGTTCTGGTATCGG |
| Bxy\|Bu_3303F | TGGATACAACGACGCTAAC |
| Bxy\|Bu_3303R | CAGTCATCTACGGTGTGAG |
| Bxy\|Bu_1991F | ATGATCGCATCTTCAGTAGA |
| Bxy\|Bu_1991R | TTGTCTGACCATTCCATCTT |
| Bxy\|Bu_6499F | GGAATCTGATTGGAACCATT |
| Bxy\|Bu_6499R | CTCGCTGAAGTTATCGTATT |
| Bxy\|Bu_7847F | AACACTGGATTGGTCTGAT |
| Bxy\|Bu_7847R | TTGACACCTGCTTGAACA |
| Bxy\|Bu_717F | ATACTGCTCAGCGAATACTT |
| Bxy\|Bu_717R | TGGACGGAGATGTGATGA |
| Bxy\|Bu_1341F | TGCTATCTTCTGTCTTCCATC |
| Bxy\|Bu_1341R | TCCATCAGACCATCTCCAA |
| Bxy\|Bu_4785F | AACAGACCAGACGAGGAT |
| Bxy\|Bu_4785R | GAGTTGAGGCTTGTAATGAC |
