# Supplementary figures and images for: Parallel Evolution of C-Type Lectin Domain Gene Family Sizes in Insect-Vectored Nematodes
Source: Front Plant Sci. 2022 Apr 25;13:856826. doi: 10.3389/fpls.2022.856826 (PMC9085898; doi:10.3389/fpls.2022.856826)

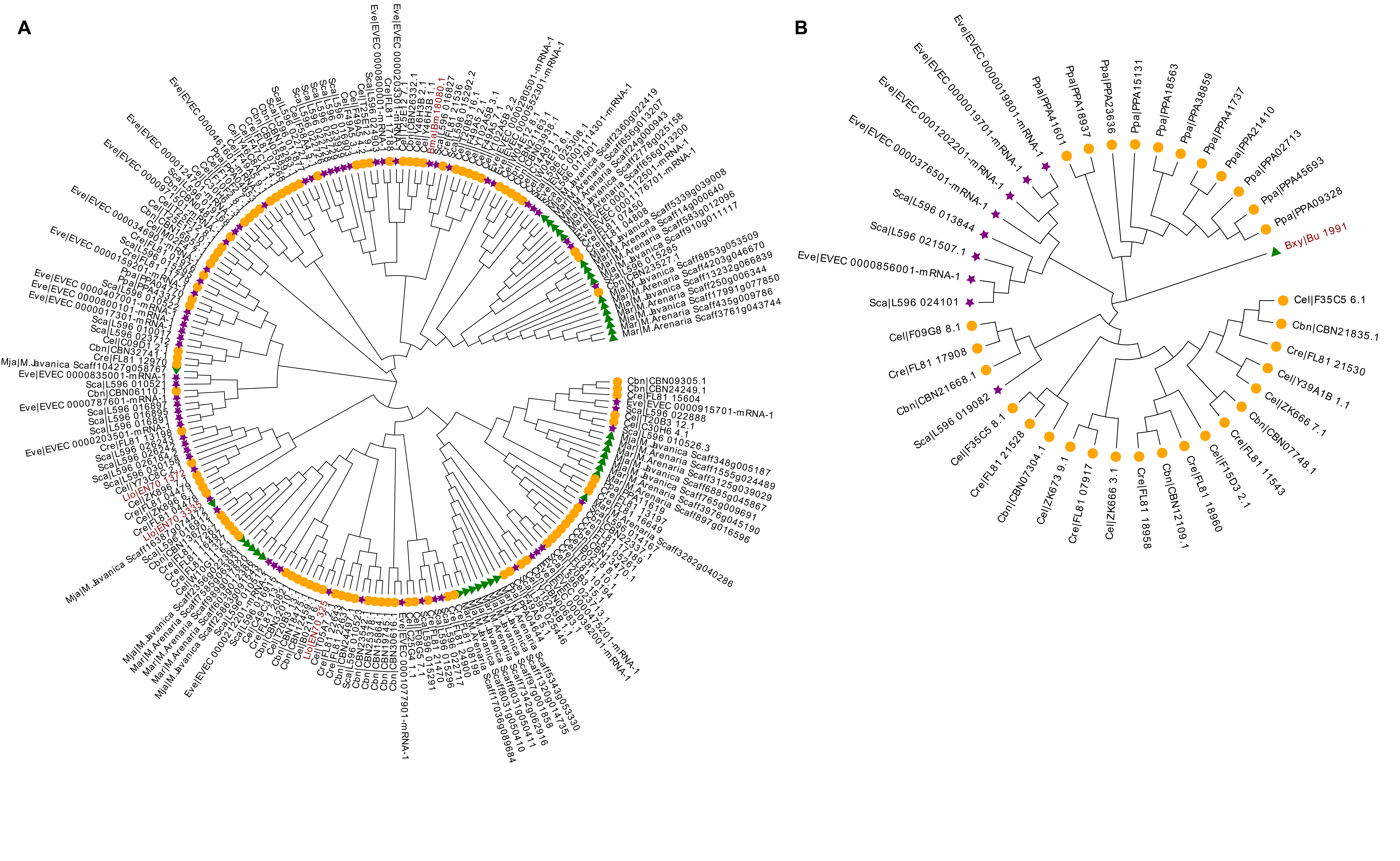

Supplement: Supplementary Figure 1 — Phylogenetic analysis of CTLs. (A) Phylogenetic analysis of type II CTLs. (B) Phylogenetic analysis of type IV CTLs. [file Image_1.JPG]

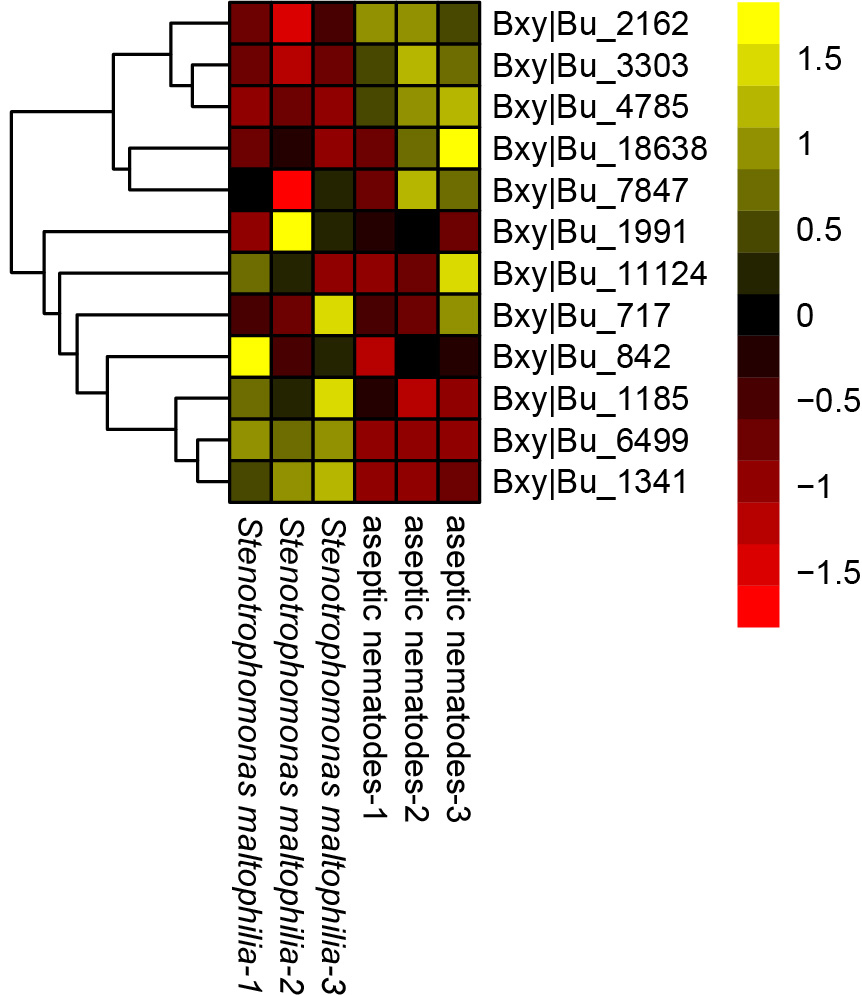

Supplement: Supplementary Figure 3 — Transcriptomic data of B. xylophilus CTL genes under S. maltophilia compared with aseptic nematodes. [file Image_3.JPG]
